# Cosmology and fundamental physics with the Euclid satellite

**DOI:** 10.1007/s41114-017-0010-3

**Published:** 2018-04-12

**Authors:** Luca Amendola, Stephen Appleby, Anastasios Avgoustidis, David Bacon, Tessa Baker, Marco Baldi, Nicola Bartolo, Alain Blanchard, Camille Bonvin, Stefano Borgani, Enzo Branchini, Clare Burrage, Stefano Camera, Carmelita Carbone, Luciano Casarini, Mark Cropper, Claudia de Rham, Jörg P. Dietrich, Cinzia Di Porto, Ruth Durrer, Anne Ealet, Pedro G. Ferreira, Fabio Finelli, Juan García-Bellido, Tommaso Giannantonio, Luigi Guzzo, Alan Heavens, Lavinia Heisenberg, Catherine Heymans, Henk Hoekstra, Lukas Hollenstein, Rory Holmes, Zhiqi Hwang, Knud Jahnke, Thomas D. Kitching, Tomi Koivisto, Martin Kunz, Giuseppe La Vacca, Eric Linder, Marisa March, Valerio Marra, Carlos Martins, Elisabetta Majerotto, Dida Markovic, David Marsh, Federico Marulli, Richard Massey, Yannick Mellier, Francesco Montanari, David F. Mota, Nelson J. Nunes, Will Percival, Valeria Pettorino, Cristiano Porciani, Claudia Quercellini, Justin Read, Massimiliano Rinaldi, Domenico Sapone, Ignacy Sawicki, Roberto Scaramella, Constantinos Skordis, Fergus Simpson, Andy Taylor, Shaun Thomas, Roberto Trotta, Licia Verde, Filippo Vernizzi, Adrian Vollmer, Yun Wang, Jochen Weller, Tom Zlosnik, The Euclid Theory Working Group

**Affiliations:** 1 University of Heidelberg, Heidelberg, Germany; 2 Korea Institute for Advanced Study (KIAS), Seoul, Korea; 3 University of Nottingham, Nottingham, UK; 4 Institute of Cosmology and Gravitation, University of Portsmouth, Portsmouth, UK; 5 University of Oxford, Oxford, UK; 6 Dipartimento di Fisica e Astronomia, Alma Mater Studiorum, University of Bologna, Via Piero Gobetti 93/2, 40129 Bologna, BO Italy; 7 INAF - Osservatorio di Astrofisica e Scienza dello Spazio di Bologna, Via Piero Gobetti 93/3, 40129 Bologna, BO Italy; 8 INFN - Istituto Nazionale di Fisica Nucleare, Sezione di Bologna, Viale Berti Pichat 6/2, 40127 Bologna, BO Italy; 9 Dipartimento di Fisica e Astronomia “G. Galilei”, Università degli Studi di Padova, via Marzolo 8, 5131 Padova, Italy; 10 INFN Sezione di Padova, via Marzolo 8, 35131 Padova, Italy; 11 INAF-Osservatorio Astronomico di Padova, Vicolo dell’Osservatorio 5, 35122 Padova, Italy; 12 IRAP, Université de Toulouse, CNRS, CNES, UPS, Toulouse, France; 13 Départment de Physique Théorique and Center for Astroparticle Physics, Université de Genève, Quai E. Ansermet 24, 1211 Genève 4, Switzerland; 14 Dipartimento di Fisica dell’ Università di Trieste, Sezione di Astronomia, Trieste, Italy; 15 INAF, Osservatorio Astronomico di Trieste, Trieste, Italy; 16 INFN, National Institute for Nuclear Physics, Trieste, Italy; 17 Dipartimento di Fisica, Università degli Studi Roma Tre, Via della Vasca Navale 84, 00146 Rome, Italy; 18 INFN Sezione di Roma 3, Via della Vasca Navale 84, 00146 Rome, Italy; 19 INAF, Osservatorio Astronomico di Roma, Monte Porzio Catone, Italy; 20 Dipartimento di Fisica, Università degli Studi di Torino, Torino, Italy; 21 INFN, Sezione di Torino, Torino, Italy; 22 INAF, Osservatorio Astrofisico di Torino, Pino Torinese, Italy; 23 Jodrell Bank Centre for Astrophysics, The University of Manchester, Manchester, UK; 24 Dipartimento di Fisica “Aldo Pontremoli”, Università degli Studi di Milano, via CeIoria 16, 20133 Milano, Italy; 25 INAF, Osservatorio Astronomico di Brera, via Brera 28, 20121 Milano, Italy; 26 INFN, Sezione di Milano, via Celoria 16, 2033 Milano, Italy; 27 Institute of Theoretical Physics, University of Oslo, Oslo, Norway; 28 International Institute of Physics, Federal University of Rio Grande do Norte, Natal, Brazil; 29 Mullard Space Science Laboratory, University College London, Holmbury St Mary, Dorking, Surrey RH5 6NT UK; 30 Imperial College London, London, UK; 31 Faculty of Physics, Ludwig-Maximilians-Universität München/Excellence Cluster Universe, Garching b. München, Germany; 32 Rome, Italy; 33 CPPM, Marseille, France; 34 Astrophysics, University of Oxford, Keble Road, Oxford, OX2 7LG UK; 35 INAF/IASF Bologna, via Gobetti 101, 40129 Bologna, Italy; 36 INFN, Sezione di Bologna, viale Berti Pichat 6/2, 40127 Bologna, Italy; 37 Instituto de Fisica Teorica, Universidad Autonoma de Madrid, Cantoblanco, 28049 Madrid, Spain; 38 Ludwig-Maximilians-Universität, Munich, Germany; 39 Dipartimento di Fisica, Università degli Studi di Milano, via G. Celoria 16, 20133 Milano, Italy; 40 INAF-Osservatorio Astronomico di Brera, Via E. Bianchi 46, 23807 Merate, Italy; 41 Institute for Theoretical Studies, ETH Zurich, Clausiusstrasse 47, 8092 Zurich, Switzerland; 42 Scottish Universities Physics Alliance, Institute for Astronomy, University of Edinburgh, Royal Observatory, Blackford Hill, Edinburgh, EH9 3HJ UK; 43 Leiden Observatory/Leiden University, Leiden, The Netherlands; 44 Wädenswil, Switzerland; 45 Stoke Mandeville, UK; 46 Sun Yat-Sen University, Zhuhai, China; 47 Max Planck Institute for Astronomy, Heidelberg, Germany; 48 Mullard Space Science Laboratory, University College London, Holmbury House, Holmbury Saint Mary, Dorking, RH6 6NT UK; 49 Nordita, KTH Royal Institute of Technology, Stockholm University, Roslagstullsbacken 23, 10691 Stockholm, Sweden; 50 University of Milano-Bicocca, Milano, Italy; 51 University of California, Berkeley, USA; 52 University of Pennsylvania, Philadelphia, USA; 53 Federal University of Espírito Santo, Vitória, Brazil; 54 Centro de Astrofísica da Universidade do Porto and IA-Porto, Rua das Estrelas, 4150-762 Porto, Portugal; 55 Départment de Physique Théorique, Université de Genève, Quai E. Ansermet 24, 1211 Genève 4, Switzerland; 56 Institute of Cosmology and Gravitation, Portsmouth, UK; 57 King’s College London, London, UK; 58 Dipartimento di Fisica e Astronomia, Università di Bologna, Via Gobetti 93/2, 40129 Bologna, Italy; 59 Institute for Computational Cosmology, Durham University, South Road, Durham, DH1 3LE UK; 60 Institut d’Astrophysique de Paris, Sorbonne Universite, 98 bis, Bd Arago, 75014 Paris, France; 61 Astrophysics Department, IRFU, CEA, Saclay, 91191 Gif-sur-Yvette, France; 62 Helsinki Institute of Physics, Helsinki, Finland; 63 Institute of Theoretical Astrophysics, University of Oslo, 0315 Oslo, Norway; 64 University of Lisbon, Lisbon, Portugal; 65 University of Portsmouth, Dennis Sciama Building, Portsmouth, PO1 3FX UK; 66 Astrophysics Department, IRFU, CEA, Université Paris-Saclay, 91191 Gif-sur-Yvette, France; 67 Université Paris-Diderot, AIM, Sorbonne Paris Cité, CEA, CNRS, 91191 Gif-sur-Yvette, France; 68 Argelander Institut für Astronomie, Auf dem Hügel 71, 53121 Bonn, Germany; 69 Department of Physics, University of Surrey, Guildford, GU2 7XH UK; 70 Department of Physics, University of Trento, Trento, Italy; 71 Departamento de Física, FCFM, Universidad de Chile, Blanco Encalada 2008, Santiago, Chile; 72 CEICO, Institute of Physics of the Czech Academy of Sciences, Na Slovance 1999/2, Praha, 182 21 Czechia; 73 I.N.A.F. - Osservatorio Astronomico di Roma, via Frascati 33, 00040 Monte Porzio Catone, Roma Italy; 74 Department of Physics, University of Cyprus, 1, Panepistimiou Street, 2109 Aglantzia, Cyprus; 75 CEICO, Institute of Physics of the Czech Academy of Sciences, Na Slovance 2, 18221 Praha 8, Czech Republic; 76 University of Barcelona, Barcelona, Spain; 77 Institute for Astronomy, University of Edinburgh, Royal Observatory, Blackford Hill, Edinburgh, EH9 3HJ Scotland; 78 London, UK; 79 Physics Department, Imperial College London, Astrophysics Group, Prince Consort Rd, London, SW7 2AZ UK; 80 Institut de Ciències del Cosmos (ICCUB), Universitat de Barcelona (IEEC-UB), Martí Franquès 1, E08028 Barcelona, Spain; 81 ICREA, Pg. Lluís Companys 23, 08010 Barcelona, Spain; 82 Institut de physique théorique, Université Paris Saclay CEA, CNRS, 91191 Gif-sur-Yvette, France; 83 Tübingen, Germany; 84 IPAC, California Institute of Technology, Pasadena, USA; 85 Perimeter Institute for Theoretical Physics, Waterloo, Canada

**Keywords:** Dark energy, Cosmology, Galaxy evolution

## Abstract

Euclid is a European Space Agency medium-class mission selected for launch in 2020 within the cosmic vision 2015–2025 program. The main goal of Euclid is to understand the origin of the accelerated expansion of the universe. Euclid will explore the expansion history of the universe and the evolution of cosmic structures by measuring shapes and red-shifts of galaxies as well as the distribution of clusters of galaxies over a large fraction of the sky. Although the main driver for Euclid is the nature of dark energy, Euclid science covers a vast range of topics, from cosmology to galaxy evolution to planetary research. In this review we focus on cosmology and fundamental physics, with a strong emphasis on science beyond the current standard models. We discuss five broad topics: dark energy and modified gravity, dark matter, initial conditions, basic assumptions and questions of methodology in the data analysis. This review has been planned and carried out within Euclid’s Theory Working Group and is meant to provide a guide to the scientific themes that will underlie the activity of the group during the preparation of the Euclid mission.

## Introduction

Euclid^1^ (Laureijs et al. 2011; Refregier 2009; Cimatti et al. 2009) is an ESA medium-class mission selected for the second launch slot (expected for 2020) of the cosmic vision 2015–2025 program. The main goal of Euclid is to understand the physical origin of the accelerated expansion of the universe. Euclid is a satellite equipped with a 1.2 m telescope and three imaging and spectroscopic instruments working in the visible and near-infrared wavelength domains. These instruments will explore the expansion history of the universe and the evolution of cosmic structures by measuring shapes and redshifts of galaxies over a large fraction of the sky. The satellite will be launched by a Soyuz ST-2.1B rocket and transferred to the L2 Lagrange point for a 6-year mission that will cover at least 15,000 square degrees of sky. Euclid plans to image a billion galaxies and measure nearly 100 million galaxy redshifts.

These impressive numbers will allow Euclid to realize a detailed reconstruction of the clustering of galaxies out to a redshift 2 and the pattern of light distortion from weak lensing to redshift 3. The two main probes, redshift clustering and weak lensing, are complemented by a number of additional cosmological probes: cross correlation between the cosmic microwave background and the large scale structure; abundance and properties of galaxy clusters and strong lensing and possible luminosity distance through supernovae Ia. To extract the maximum of information also in the nonlinear regime of perturbations, these probes will require accurate high-resolution numerical simulations. Besides cosmology, Euclid will provide an exceptional dataset for galaxy evolution, galaxy structure, and planetary searches. All Euclid data will be publicly released after a relatively short proprietary period and will constitute for many years the ultimate survey database for astrophysics.

A huge enterprise like Euclid requires highly considered planning in terms not only of technology but also for the scientific exploitation of future data. Many ideas and models that today seem to be abstract exercises for theorists will in fact finally become testable with the Euclid surveys. The main science driver of Euclid is clearly the nature of dark energy, the enigmatic substance that is driving the accelerated expansion of the universe. As we discuss in detail in Part I, under the label “dark energy” we include a wide variety of hypotheses, from extradimensional physics to higher-order gravity, from new fields and new forces to large violations of homogeneity and isotropy. The simplest explanation, Einstein’s famous cosmological constant, is still currently acceptable from the observational point of view, but is not the only one, nor necessarily the most satisfying, as we will argue. Therefore, it is important to identify the main observables that will help distinguish the cosmological constant from the alternatives and to forecast Euclid’s performance in testing the various models.

Since clustering and weak lensing also depend on the properties of dark matter, Euclid is a dark matter probe as well. In Part II we focus on the models of dark matter that can be tested with Euclid data, from massive neutrinos to ultra-light scalar fields. We show that Euclid can measure the neutrino mass to a very high precision, making it one of the most sensitive neutrino experiments of its time, and it can help identify new light fields in the cosmic fluid.

The evolution of perturbations depends not only on the fields and forces active during the cosmic eras, but also on the initial conditions. By reconstructing the initial conditions we open a window on the inflationary physics that created the perturbations, and allow ourselves the chance of determining whether a single inflaton drove the expansion or a mixture of fields. In Part III we review the choices of initial conditions and their impact on Euclid science. In particular we discuss deviations from simple scale invariance, mixed isocurvature-adiabatic initial conditions, non-Gaussianity, and the combined forecasts of Euclid and CMB experiments.

Practically all of cosmology is built on the copernican principle, a very fruitful idea postulating a homogeneous and isotropic background. Although this assumption has been confirmed time and again since the beginning of modern cosmology, Euclid’s capabilities can push the test to new levels. In Part IV we challenge some of the basic cosmological assumptions and predict how well Euclid can constrain them. We explore the basic relation between luminosity and angular diameter distance that holds in any metric theory of gravity if the universe is transparent to light, and the existence of large violations of homogeneity and isotropy, either due to local voids or to the cumulative stochastic effects of perturbations, or to intrinsically anisotropic vector fields or spacetime geometry.

Finally, in Part V we review some of the statistical methods that are used to forecast the performance of probes like Euclid, and we discuss some possible future developments.

This review has been planned and carried out within Euclid’s Theory Working Group and is meant to provide a guide to the scientific themes that will underlie the activity of the group during the preparation of the mission. At the same time, this review will help us and the community at large to identify the areas that deserve closer attention, to improve the development of Euclid science and to offer new scientific challenges and opportunities.

## Part I Dark energy

### Introduction

With the discovery of cosmic acceleration at the end of the 1990s, and its possible explanation in terms of a cosmological constant, cosmology has returned to its roots in Einstein’s famous 1917 paper that simultaneously inaugurated modern cosmology and the history of the constant $\varLambda$. Perhaps cosmology is approaching a robust and all-encompassing standard model, like its cousin, the very successful standard model of particle physics. In this scenario, the cosmological standard model could essentially close the search for a broad picture of cosmic evolution, leaving to future generations only the task of filling in a number of important, but not crucial, details.

The cosmological constant is still in remarkably good agreement with almost all cosmological data more than 10 years after the observational discovery of the accelerated expansion rate of the universe. However, our knowledge of the universe’s evolution is so incomplete that it would be premature to claim that we are close to understanding the ingredients of the cosmological standard model. If we ask ourselves what we know for certain about the expansion rate at redshifts larger than unity, or the growth rate of matter fluctuations, or about the properties of gravity on large scales and at early times, or about the influence of extra dimensions (or their absence) on our four dimensional world, the answer would be surprisingly disappointing.

Our present knowledge can be succinctly summarized as follows: we live in a universe that is consistent with the presence of a cosmological constant in the field equations of general relativity, and as of 2016, the value of this constant corresponds to a fractional energy density today of $\varOmega _{\varLambda }\approx 0.7$. However, far from being disheartening, this current lack of knowledge points to an exciting future. A decade of research on dark energy has taught many cosmologists that this ignorance can be overcome by the same tools that revealed it, together with many more that have been developed in recent years.

Why then is the cosmological constant not the end of the story as far as cosmic acceleration is concerned? There are at least three reasons. The first is that we have no simple way to explain its small but non-zero value. In fact, its value is unexpectedly small with respect to any physically meaningful scale, except the *current* horizon scale. The second reason is that this value is not only small, but also surprisingly close to another unrelated quantity, the *present* matter-energy density. That this happens just by coincidence is hard to accept, as the matter density is diluted rapidly with the expansion of space. Why is it that we happen to live at the precise, fleeting epoch when the energy densities of matter and the cosmological constant are of comparable magnitude? Finally, observations of coherent acoustic oscillations in the cosmic microwave background (CMB) have turned the notion of accelerated expansion in the very early universe (inflation) into an integral part of the cosmological standard model. Yet the simple truth that we exist as observers demonstrates that this early accelerated expansion was of a finite duration, and hence cannot be ascribable to a true, constant $\varLambda$; this sheds doubt on the nature of the current accelerated expansion. The very fact that we know so little about the past dynamics of the universe forces us to enlarge the theoretical parameter space and to consider phenomenology that a simple cosmological constant cannot accommodate.

These motivations have led many scientists to challenge one of the most basic tenets of physics: Einstein’s law of gravity. Einstein’s theory of general relativity (GR) is a supremely successful theory on scales ranging from the size of our solar system down to micrometers, the shortest distances at which GR has been probed in the laboratory so far. Although specific predictions about such diverse phenomena as the gravitational redshift of light, energy loss from binary pulsars, the rate of precession of the perihelia of bound orbits, and light deflection by the sun are not unique to GR, it must be regarded as highly significant that GR is consistent with each of these tests and more. We can securely state that GR has been tested to high accuracy *at these distance scales*.

The success of GR on larger scales is less clear. On astrophysical and cosmological scales, tests of GR are complicated by the existence of invisible components like dark matter and by the effects of spacetime geometry. We do not know whether the physics underlying the apparent cosmological constant originates from modifications to GR (i.e., an extended theory of gravity), or from a new fluid or field in our universe that we have not yet detected directly. The latter phenomena are generally referred to as ‘dark energy’ models.

If we only consider observations of the expansion rate of the universe we cannot discriminate between a theory of modified gravity and a dark-energy model. However, it is likely that these two alternatives will cause perturbations around the ‘background’ universe to behave differently. Only by improving our knowledge of the growth of structure in the universe can we hope to progress towards breaking the degeneracy between dark energy and modified gravity. Part I of this review is dedicated to this effort. We begin with a review of the background and linear perturbation equations in a general setting, defining quantities that will be employed throughout. We then explore the nonlinear effects of dark energy, making use of analytical tools such as the spherical collapse model, perturbation theory and numerical *N*-body simulations. We discuss a number of competing models proposed in literature and demonstrate what the Euclid survey will be able to tell us about them. For an updated review of present cosmological constraints on a variety of dark energy and modified gravity models, we refer to the Planck 2015 analysis (Planck Collaboration 2016c).

### Background evolution

Most of the calculations in this review are performed in the Friedmann–Lemaître–Robertson–Walker (FLRW) metric

I.2.1 $$\begin{aligned} \mathrm {d}s^2=-\,\mathrm {d}t^2+{a(t)^2}\left( \frac{\mathrm {d}r^2}{1-kr^2}+r^2 \, \mathrm {d}\theta ^2+r^2\sin ^2\theta \, \mathrm {d}\phi ^2\right) , \end{aligned}$$

where *a*(*t*) is the scale factor (normalized to $a=1$ today) and *k* the spatial curvature. The usual symbols for the Hubble function $H=\dot{a}/a$ and the density fractions $\varOmega _x$, where *x* stands for the component, are employed. We characterize the components with the subscript *M* or *m* for matter, $\gamma$ or *r* for radiation, *b* for baryons, *k* or *K* for curvature and $\varLambda$ for the cosmological constant. Whenever necessary for clarity, we append a subscript 0 to denote the present epoch, e.g., $\varOmega _{M,0}$. Sometimes the conformal time $\eta =\int \mathrm {d}t/a$ and the conformal Hubble function $\mathcal {H}=aH= \mathrm {d}a/(a\mathrm {d}\eta )$ are employed. Unless otherwise stated, we denote with a dot derivatives w.r.t. cosmic time *t* (and sometimes we employ the dot for derivatives w.r.t. conformal time $\eta$) while we use a prime for derivatives with respect to $\ln a$.

The energy density due to a cosmological constant with $p=-\,\rho$ is obviously constant over time. This can easily be seen from the covariant conservation equation $T_{\mu ;\nu }^\nu =0$ for the homogeneous and isotropic FLRW metric,

I.2.2 $$\begin{aligned} \dot{\rho } + 3 H (\rho +p) = 0. \end{aligned}$$

However, since we also observe radiation with $p=\rho /3$ and non-relativistic matter for which $p\approx 0$, it is natural to assume that the dark energy is not necessarily limited to a constant energy density, but that it could be dynamical instead.

One of the simplest models that explicitly realizes such a dynamical dark energy scenario is described by a minimally-coupled canonical scalar field evolving in a given potential. For this reason, the very concept of dynamical dark energy is often associated with this scenario, and in this context it is called ‘quintessence’ (Wetterich 1988; Ratra and Peebles 1988). In the following, the scalar field will be indicated with $\phi$. Although in this simplest framework the dark energy does not interact with other species and influences spacetime only through its energy density and pressure, this is not the only possibility and we will encounter more general models later on. The homogeneous energy density and pressure of the scalar field $\phi$ are defined as

I.2.3 $$\begin{aligned} \rho _{\phi } = \frac{{{\dot{\phi }}}^2}{2 } + V(\phi ), \quad p_{\phi } = \frac{{{\dot{\phi }}}^2}{2 } - V(\phi ), \quad w_{\phi } = \frac{p_{\phi }}{\rho _{\phi }}, \end{aligned}$$

and $w_\phi$ is called the equation-of-state parameter. Minimally-coupled dark-energy models can allow for attractor solutions (Copeland et al. 1998; Liddle and Scherrer 1999; Steinhardt et al. 1999): if an attractor exists, depending on the potential $V(\phi )$ in which dark energy rolls, the trajectory of the scalar field in the present regime converges to the path given by the attractor, though starting from a wide set of different initial conditions for $\phi$ and for its first derivative ${\dot{\phi }}$. Inverse power law and exponential potentials are typical examples of potential that can lead to attractor solutions. As constraints on $w_\phi$ become tighter (e.g., Komatsu et al. 2011), the allowed range of initial conditions to follow into the attractor solution shrinks, so that minimally-coupled quintessence is actually constrained to have very flat potentials. The flatter the potential, the more minimally-coupled quintessence mimics a cosmological constant, the more it suffers from the same fine-tuning and coincidence problems that affect a $\varLambda$CDM scenario (Matarrese et al. 2004).

However, when GR is modified or when an interaction with other species is active, dark energy may very well have a non-negligible contribution at early times. Therefore, it is important, already at the background level, to understand the best way to characterize the main features of the evolution of quintessence and dark energy in general, pointing out which parameterizations are more suitable and which ranges of parameters are of interest to disentangle quintessence or modified gravity from a cosmological constant scenario.

In the following we briefly discuss how to describe the cosmic expansion rate in terms of a small number of parameters. This will set the stage for the more detailed cases discussed in the subsequent sections. Even within specific physical models it is often convenient to reduce the information to a few phenomenological parameters.

Two important points are left for later: from Eq. (I.2.3) we can easily see that $w_\phi \ge -1$ as long as $\rho _\phi >0$, i.e., uncoupled canonical scalar field dark energy never crosses $w_\phi =-\,1$. However, this is not necessarily the case for non-canonical scalar fields or for cases where GR is modified. We postpone to Sect. I.3.3 the discussion of how to parametrize this ‘phantom crossing’ to avoid singularities, as it also requires the study of perturbations.

The second deferred part on the background expansion concerns a basic statistical question: what is a sensible precision target for a measurement of dark energy, e.g., of its equation of state? In other words, how close to $w_\phi =-\,1$ should we go before we can be satisfied and declare that dark energy is the cosmological constant? We will address this question in Sect. I.4.

#### Parametrization of the background evolution

If one wants to parametrize the equation of state of dark energy, two general approaches are possible. The first is to start from a set of dark-energy models given by the theory and to find parameters describing their $w_\phi$ as accurately as possible. Only later one can try and include as many theoretical models as possible in a single parametrization. In the context of scalar-field dark-energy models (to be discussed in Sect. I.5.1), Crittenden et al. (2007) parametrize the case of slow-rolling fields, Scherrer and Sen (2008) study thawing quintessence, Hrycyna and Szydlowski (2007) and Chiba et al. (2010) include non-minimally coupled fields, Setare and Saridakis (2009) quintom quintessence, Dutta and Scherrer (2008) parametrize hilltop quintessence, Chiba et al. (2009) extend the quintessence parametrization to a class of *k*-essence models, Huang et al. (2011) study a common parametrization for quintessence and phantom fields. Another convenient way to parametrize the presence of a non-negligible homogeneous dark energy component at early times (usually labeled as EDE) was presented in Wetterich (2004). We recall it here because we will refer to this example in Sect. I.6.1.1. In this case the equation of state is parametrized as:

I.2.4 $$\begin{aligned} {w}_X (z) = \frac{{ w}_0}{1+b \ln {(1+z)}}, \end{aligned}$$

where *b* is a constant related to the amount of dark energy at early times, i.e.,

I.2.5 $$\begin{aligned} b = -\, \frac{3 w_0}{\ln {\frac{1-\varOmega _{e}}{\varOmega _{e}}} + \ln {\frac{1-\varOmega _{m,0}}{\varOmega _{m,0}}}}. \end{aligned}$$

Here the subscripts ‘0’ and ‘*e*’ refer to quantities calculated today or early times, respectively. With regard to the latter parametrization, we note that concrete theoretical and realistic models involving a non-negligible energy component at early times are often accompanied by further important modifications (as in the case of interacting dark energy), not always included in a parametrization of the sole equation of state such as (I.2.4) (for further details see Sect. I.6 on nonlinear aspects of dark energy and modified gravity).

The second approach is to start from a simple expression of *w* without assuming any specific dark-energy model (but still checking afterwards whether known theoretical dark-energy models can be represented). This is what has been done by Huterer and Turner (2001), Maor et al. (2001), Weller and Albrecht (2001) (linear and logarithmic parametrization in *z*), Chevallier and Polarski (2001), Linder (2003) (linear and power law parametrization in *a*), Douspis et al. (2006), Bassett et al. (2004) (rapidly varying equation of state).

The most common parametrization, widely employed in this review, is the linear equation of state (Chevallier and Polarski 2001; Linder 2003)

I.2.6 $$\begin{aligned} w_X(a)=w_0+w_a (1-a), \end{aligned}$$

where the subscript *X* refers to the generic dark-energy constituent. While this parametrization is useful as a toy model in comparing the forecasts for different dark-energy projects, it should not be taken as all-encompassing. In general, a dark-energy model can introduce further significant terms in the effective $w_X(z)$ that cannot be mapped onto the simple form of Eq. (I.2.6).

An alternative to model-independent constraints is measuring the dark-energy density $\rho _X(z)$ (or the expansion history *H*(*z*)) as a free function of cosmic time (Wang and Garnavich 2001; Tegmark 2002; Daly and Djorgovski 2003). Measuring $\rho _X(z)$ has advantages over measuring the dark-energy equation of state $w_X(z)$ as a free function; $\rho _X(z)$ is more closely related to observables, hence is more tightly constrained for the same number of redshift bins used (Wang and Garnavich 2001; Wang and Freese 2006). Note that $\rho _X(z)$ is related to $w_X(z)$ as follows (Wang and Garnavich 2001):

I.2.7 $$\begin{aligned} \frac{\rho _X(z)}{\rho _X(0)} = \exp \left\{ \int _0^z \, \mathrm {d}z'\, \frac{3 \left[ 1+w_X\left( z'\right) \right] }{1+z'} \right\} {.} \end{aligned}$$

Hence, parametrizing dark energy with $w_X(z)$ implicitly assumes that $\rho _X(z)$ does not change sign in cosmic time. This precludes whole classes of dark-energy models in which $\rho _X(z)$ becomes negative in the future (“Big crunch” models, see Wang et al. 2004 for an example) (Wang and Tegmark 2004).

Note that the measurement of $\rho _X(z)$ is straightforward once *H*(*z*) is measured from baryon acoustic oscillations, and $\varOmega _m$ is constrained tightly by the combined data from galaxy clustering, weak lensing, and cosmic microwave background data—although strictly speaking this requires a choice of perturbation evolution for the dark energy as well, and in addition one that is not degenerate with the evolution of dark matter perturbations; see Kunz (2009).

Another useful possibility is to adopt the principal component approach (Huterer and Starkman 2003), which avoids any assumption about the form of *w* and assumes it to be constant or linear in redshift bins, then derives which combination of parameters is best constrained by each experiment.

For a cross-check of the results using more complicated parameterizations, one can use simple polynomial parameterizations of *w* and $\rho _{\mathrm {DE}}(z)/\rho _{\mathrm {DE}}(0)$ (Wang 2008b).

### Perturbations

This section is devoted to a discussion of linear perturbation theory in dark-energy models. Since we will discuss a number of non-standard models in later sections, we present here the main equations in a general form that can be adapted to various contexts. This section will identify which perturbation functions the Euclid survey (Laureijs et al. 2011) will try to measure and how they can help us to characterize the nature of dark energy and the properties of gravity.

#### Cosmological perturbation theory

Here we provide the perturbation equations in a dark-energy dominated universe for a general fluid, focusing on scalar perturbations.

For simplicity, we consider a flat universe containing only (cold dark) matter and dark energy, so that the Hubble parameter is given by

I.3.1 $$\begin{aligned} H^2 =\left( \frac{1}{a} \frac{\mathrm {d}a}{\mathrm {d}t} \right) ^2 = H_{0}^{2}\left[ \varOmega _{m_0} a^{-3}+\left( 1- \varOmega _{m_0} \right) \exp \left( -3\int _1^a\frac{1+w(a')}{a'} \, \mathrm {d}a \right) \right] .\nonumber \\ \end{aligned}$$

We will consider linear perturbations on a spatially-flat background model, defined by the line of element

I.3.2 $$\begin{aligned} \mathrm {d}s^{2} = a^{2} \left[ -\left( 1+2A\right) \, \mathrm {d}\eta ^{2}+2B_{i} \, \mathrm {d}\eta \, \mathrm {d}x^{i}+\left( \left( 1+2H_{L}\right) \delta _{ij}+2H_{Tij} \right) \, \mathrm {d}x_{i} \, \mathrm {d}x^{j} \right] , \nonumber \\ \end{aligned}$$

where *A* is the scalar potential; $B_{i}$ a vector shift; $H_{L}$ is the scalar perturbation to the spatial curvature; $H_{T}^{ij}$ is the trace-free distortion to the spatial metric; $\mathrm {d}\eta = \mathrm {d}t/a$ is the conformal time.

We will assume that the universe is filled with perfect fluids only, so that the energy momentum tensor takes the simple form

I.3.3 $$\begin{aligned} T^{\mu \nu }=\left( \rho +p\right) u^{\mu }u^{\nu } +p\ g^{\mu \nu }+\varPi ^{\mu \nu }, \end{aligned}$$

where $\rho$ and *p* are the density and the pressure of the fluid respectively, $u^{\mu }$ is the four-velocity and $\varPi ^{\mu \nu }$ is the anisotropic-stress perturbation tensor that represents the traceless component of the $T_{j}^{i}$.

The components of the perturbed energy momentum tensor can be written as:

I.3.4 $$\begin{aligned} T_{0}^{0}= & {} - \left( {\bar{\rho }} + \delta \rho \right) \end{aligned}$$

I.3.5 $$\begin{aligned} T_{j}^{0}= & {} \left( {\bar{\rho }} + \bar{p} \right) \left( v_{j} - B_{j} \right) \end{aligned}$$

I.3.6 $$\begin{aligned} T_{0}^{i}= & {} \left( {\bar{\rho }} + \bar{p} \right) v^{i} \end{aligned}$$

I.3.7 $$\begin{aligned} T_{j}^{i}= & {} \left( \bar{p} + \delta {p} \right) \delta _{j}^{i} + \bar{p}\ \varPi _{j}^{i}. \end{aligned}$$

Here ${\bar{\rho }}$ and ${\bar{p}}$ are the energy density and pressure of the homogeneous and isotropic background universe, $\delta \rho$ is the density perturbation, $\delta p$ is the pressure perturbation, $v^{i}$ is the velocity vector. Here we want to investigate only the scalar modes of the perturbation equations. So far the treatment of the matter and metric is fully general and applies to any form of matter and metric. We now choose the Newtonian gauge (also known as the longitudinal or Poisson gauge), characterized by zero non-diagonal metric terms (the shift vector $B_{i}=0$ and $H_{T}^{ij}=0$) and by two scalar potentials $\varPsi$ and $\varPhi$; the metric Eq. (I.3.2) then becomes

I.3.8 $$\begin{aligned} \mathrm {d}s^{2} = a^{2} \left[ -\left( 1+2\varPsi \right) \, \mathrm {d}\eta ^{2} + \left( 1-2\varPhi \right) \, \mathrm {d}x_{i} \, \mathrm {d}x^{i} \right] . \end{aligned}$$

The advantage of using the Newtonian gauge is that the metric tensor $g_{\mu \nu }$ is diagonal and this simplifies the calculations. This choice not only simplifies the calculations but is also the most intuitive one as the observers are attached to the points in the unperturbed frame; as a consequence, they will detect a velocity field of particles falling into the clumps of matter and will measure their gravitational potential, represented directly by $\varPsi$; $\varPhi$ corresponds to the perturbation to the spatial curvature. Moreover, as we will see later, the Newtonian gauge is the best choice for observational tests (i.e., for perturbations smaller than the horizon).

In the conformal Newtonian gauge, and in Fourier space, the first-order perturbed Einstein equations give (see Ma and Bertschinger 1995, for more details):

I.3.9 $$\begin{aligned}&k^2\varPhi + 3\frac{\dot{a}}{a} \left( \dot{\varPhi } + \frac{\dot{a}}{a}\varPsi \right) = -4\pi G a^2 \sum _{\alpha }\bar{\rho }_{\alpha }\delta _{\alpha }, \end{aligned}$$

I.3.10 $$\begin{aligned}&k^2 \left( \dot{\varPhi } + \frac{\dot{a}}{a}\varPsi \right) = 4\pi G a^2 \sum _{\alpha }(\bar{\rho }_{\alpha }+\bar{p}_{\alpha }) \theta _{\alpha }, \end{aligned}$$

I.3.11 $$\begin{aligned}&\ddot{\varPhi } + \frac{\dot{a}}{a} (\dot{\varPsi }+2\dot{\varPhi })+\left( 2\frac{\ddot{a}}{a} - \frac{\dot{a}^2}{a^2}\right) \varPsi + \frac{k^2}{3} (\varPhi -\varPsi ) = 4\pi G a^2 \sum _{\alpha }\delta p_{\alpha },\qquad \qquad \end{aligned}$$

I.3.12 $$\begin{aligned}&k^2(\varPhi -\varPsi ) = 12\pi G a^2 \sum _{\alpha }\left( \bar{\rho }_{\alpha }+\bar{p}_{\alpha }\right) \pi _{\alpha }, \end{aligned}$$

where a dot denotes $d/d\eta$, $\delta _\alpha =\delta \rho _\alpha /\bar{\rho }_\alpha$, the index $\alpha$ indicates a sum over all matter components in the universe and $\pi$ is related to $\varPi _{j}^{i}$ through:

I.3.13 $$\begin{aligned} \left( \bar{\rho }+\bar{p}\right) \pi = -\,\left( \hat{k}_i\hat{k}_j-\frac{1}{3}\delta _{ij}\right) \varPi _{j}^{i}. \end{aligned}$$

The energy–momentum tensor components in the Newtonian gauge become:

I.3.14 $$\begin{aligned} T_{0}^{0}= & {} -\left( {\bar{\rho }} + \delta \rho \right) \end{aligned}$$

I.3.15 $$\begin{aligned} ik_i T_{0}^{i}= & {} -ik_i T_{i}^{0} = \left( {\bar{\rho }} + \bar{p} \right) \theta \end{aligned}$$

I.3.16 $$\begin{aligned} T_{j}^{i}= & {} \left( {\bar{p}} + \delta p \right) \delta _{j}^{i} +\bar{p}\varPi _{j}^{i} \end{aligned}$$

where we have defined the variable $\theta =ik_j v^j$ that represents the divergence of the velocity field.

Perturbation equations for a single fluid are obtained taking the covariant derivative of the perturbed energy momentum tensor, i.e., $T_{\nu ;\mu }^{\mu }=0$. We have

I.3.17 $$\begin{aligned} {\dot{\delta }}= & {} -\left( 1+w \right) \left( \theta - 3{\dot{\varPhi }} \right) -3\frac{\dot{a}}{a} \left( \frac{\delta p}{{\bar{\rho }}} - w\delta \right) \qquad {\mathrm {for}}~~~~\nu =0 \end{aligned}$$

I.3.18 $$\begin{aligned} {\dot{\theta }}= & {} -\frac{\dot{a}}{a} \left( 1-3w \right) \theta - \frac{\dot{w}}{1+w}\theta +k^{2}\frac{\delta {p}/{\bar{\rho }}}{1+w} + k^{2}\varPsi - k^2\pi \quad {\mathrm {for}}\quad \nu =i .\qquad \qquad \end{aligned}$$

The equations above are valid for any fluid. The evolution of the perturbations depends on the characteristics of the fluids considered, i.e., we need to specify the equation of state parameter *w*, the pressure perturbation $\delta p$ and the anisotropic stress $\pi$. For instance, if we want to study how matter perturbations evolve, we simply substitute $w=\delta p = \pi = 0$ (matter is pressureless) in the above equations. However, Eqs. (I.3.17) and (I.3.18) depend on the gravitational potentials $\varPsi$ and $\varPhi$, which in turn depend on the evolution of the perturbations of the other fluids. For instance, if we assume that the universe is filled by dark matter and dark energy then we need to specify $\delta p$ and $\pi$ for the dark energy.

The problem here is not only to parameterize the pressure perturbation and the anisotropic stress for the dark energy (there is not a unique way to do it, see below, especially Sect. I.3.3 for what to do when *w* crosses $-\,1$) but rather that we need to run the perturbation equations for each model we assume, making predictions and compare the results with observations. Clearly, this approach takes too much time. In the following Sect. I.3.2 we show a general approach to understanding the observed late-time accelerated expansion of the universe through the evolution of the matter density contrast.

In the following, whenever there is no risk of confusion, we remove the overbars from the background quantities.

#### Modified growth parameters

Even if the expansion history, *H*(*z*), of the FLRW background has been measured (at least up to redshifts $\sim \, 1$ by supernova data, i.e., via the luminosity distance), it is not possible yet to identify the physics causing the recent acceleration of the expansion of the universe. Information on the growth of structure at different scales and different redshifts is needed to discriminate between models of dark energy (DE) and modified gravity (MG). A definition of what we mean by DE and MG will be postponed to Sect. I.5.

An alternative to testing predictions of specific theories is to parameterize the possible departures from a fiducial model. Two conceptually-different approaches are widely discussed in the literature:

- *Model parameters* capture the degrees of freedom of DE/MG and modify the evolution equations of the energy–momentum content of the fiducial model. They can be associated with physical meanings and have uniquely-predicted behavior in specific theories of DE and MG.
- *Trigger relations* are derived directly from observations and only hold in the fiducial model. They are constructed to break down if the fiducial model does not describe the growth of structure correctly.

As the current observations favor concordance cosmology, the fiducial model is typically taken to be spatially flat FLRW in GR with cold dark matter and a cosmological constant, hereafter referred to as $\varLambda$CDM.

For a large-scale structure and weak lensing survey the crucial quantities are the matter-density contrast and the gravitational potentials and we therefore focus on scalar perturbations in the Newtonian gauge with the metric (I.3.8).

We describe the matter perturbations using the gauge-invariant comoving density contrast $\varDelta _M\equiv \delta _M+3aH \theta _M/k^2$ where $\delta _M$ and $\theta _M$ are the matter density contrast and the divergence of the fluid velocity for matter, respectively. The discussion can be generalized to include multiple fluids.

In $\varLambda$CDM, after radiation-matter equality there is no anisotropic stress present and the Einstein constraint equations become

I.3.19 $$\begin{aligned} -k^2 \varPhi = 4\pi G a^2 \rho _M \varDelta _M, \qquad \varPhi =\varPsi . \end{aligned}$$

These can be used to reduce the energy–momentum conservation of matter simply to the second-order growth equation

I.3.20 $$\begin{aligned} \varDelta _M''+\left[ 2+(\ln H)'\right] \varDelta _M' = \frac{3}{2}\varOmega _M(a)\varDelta _M. \end{aligned}$$

Primes denote derivatives with respect to $\ln a$ and we define the time-dependent fractional matter density as $\varOmega _M(a)\equiv 8\pi G\rho _M(a)/(3H^2)$. Notice that the evolution of $\varDelta _M$ is driven by $\varOmega _M(a)$ and is scale-independent throughout (valid on sub- and super-Hubble scales after radiation-matter equality). We define the growth factor *G*(*a*) as $\varDelta =\varDelta _0G(a)$. This is very well approximated by the expression

I.3.21 $$\begin{aligned} G(a)\approx \exp \left\{ \int _1^a \frac{\mathrm {d}a'}{a'}\left[ \varOmega _M\left( a'\right) ^\gamma \right] \right\} \end{aligned}$$

and

I.3.22 $$\begin{aligned} f_g\equiv \frac{\mathrm {d}\log G}{\mathrm {d}\log a}\approx \varOmega _M(a)^\gamma \end{aligned}$$

defines the growth rate and the growth index $\gamma$ that is found to be $\gamma _{\varLambda }\simeq 0.545$ for the $\varLambda$CDM solution (see Wang and Steinhardt 1998; Linder 2005; Huterer and Linder 2007; Ferreira and Skordis 2010).

Clearly, if the actual theory of structure growth is not the $\varLambda$CDM scenario, the constraints (I.3.19) will be modified, the growth Eq. (I.3.20) will be different, and finally the growth factor (I.3.21) is changed, i.e., the growth index is different from $\gamma _\varLambda$ and may become time and scale dependent. Therefore, the inconsistency of these three points of view can be used to test the $\varLambda$CDM paradigm.

**I.3.2.1 Two new degrees of freedom**

Any generic modification of the dynamics of scalar perturbations with respect to the simple scenario of a smooth dark-energy component that only alters the background evolution of $\varLambda$CDM can be represented by introducing two new degrees of freedom in the Einstein constraint equations. We do this by replacing (I.3.19) with

I.3.23 $$\begin{aligned} -k^2 \varPhi = 4\pi G Q(a,k) a^2 \rho _M \varDelta _M, \qquad \varPhi =\eta (a,k)\varPsi . \end{aligned}$$

Non-trivial behavior of the two functions *Q* and $\eta$ can be due to a clustering dark-energy component or some modification to GR. In MG models the function *Q*(*a*, *k*) represents a mass screening effect due to local modifications of gravity and effectively modifies Newton’s constant. In dynamical DE models *Q* represents the additional clustering due to the perturbations in the DE. On the other hand, the function $\eta (a,k)$ parameterizes the effective anisotropic stress introduced by MG or DE, which is absent in $\varLambda$CDM.

Given an MG or DE theory, the scale- and time-dependence of the functions *Q* and $\eta$ can be derived and predictions projected into the $(Q,\eta )$ plane. This is also true for interacting dark sector models, although in this case the identification of the total matter density contrast (DM plus baryonic matter) and the galaxy bias become somewhat contrived (see, e.g., Song et al. 2010, for an overview of predictions for different MG/DE models).

Using the above-defined modified constraint Eq. (I.3.23), the conservation equations of matter perturbations can be expressed in the following form (see Pogosian et al. 2010)

I.3.24 $$\begin{aligned} \varDelta _M'= & {} -\frac{1/\eta -1+(\ln Q)'}{x_Q^2+\frac{9}{2}\varOmega _M}\, \frac{9}{2}\varOmega _M \varDelta _M - \frac{x_Q^2-3(\ln H)'/Q}{x_Q^2+\frac{9}{2}\varOmega _M}\, \frac{\theta _M}{aH} \nonumber \\ \theta _M'= & {} -\theta _M - \frac{3}{2}aH\varOmega _M \frac{Q}{\eta }\varDelta _M, \end{aligned}$$

where we define $x_Q\equiv k/(aH\sqrt{Q})$. Remember $\varOmega _M=\varOmega _M(a)$ as defined above. Notice that it is $Q/\eta$ that modifies the source term of the $\theta _M$ equation and therefore also the growth of $\varDelta _M$. Together with the modified Einstein constraints (I.3.23) these evolution equations form a closed system for $(\varDelta _M,\theta _M,\varPhi ,\varPsi )$ which can be solved for given $(Q,\eta )$.

The influence of the Hubble scale is modified by *Q*, such that now the size of $x_Q$ determines the behavior of $\varDelta _M$; on “sub-Hubble” scales, $x_Q\gg 1$, we find

I.3.25 $$\begin{aligned} \varDelta _M''+\left[ 2+(\ln H)'\right] \varDelta _M' = \frac{3}{2}\varOmega _M(a) \frac{Q}{\eta } \varDelta _M \, \end{aligned}$$

and $\theta _M=-\,aH\varDelta _M'$. The growth equation is only modified by the factor $Q/\eta$ on the RHS with respect to $\varLambda$CDM (I.3.20). On “super-Hubble” scales, $x_Q\ll 1$, we have

I.3.26 $$\begin{aligned} \varDelta _M'= & {} -\left[ 1/\eta -1+(\ln Q)'\right] \varDelta _M + \frac{2}{3\varOmega _M}\frac{(\ln H)'}{aH}\frac{1}{Q} \theta _M, \nonumber \\ \theta _M'= & {} -\theta _M - \frac{3}{2}\varOmega _M\,aH \frac{Q}{\eta }\varDelta _M. \end{aligned}$$

*Q* and $\eta$ now create an additional drag term in the $\varDelta _M$ equation, except if $\eta >1$ when the drag term could flip sign. Pogosian et al. (2010) also showed that the metric potentials evolve independently and scale-invariantly on super-Hubble scales as long as $x_Q\rightarrow 0$ for $k \rightarrow 0$. This is needed for the comoving curvature perturbation, $\zeta$, to be constant on super-Hubble scales.

Many different names and combinations of the above defined functions $(Q,\eta )$ have been used in the literature, some of which are more closely related to actual observables and are less correlated than others in certain situations (see, e.g., Amendola et al. 2008b; Mota et al. 2007; Song et al. 2010; Pogosian et al. 2010; Daniel et al. 2010; Daniel and Linder 2010; Ferreira and Skordis 2010).

For instance, as observed above, the combination $Q/\eta$ modifies the source term in the growth equation. Moreover, peculiar velocities are following gradients of the Newtonian potential, $\varPsi$, and therefore the comparison of peculiar velocities with the density field is also sensitive to $Q/\eta$. So we define

I.3.27 $$\begin{aligned} \mu \equiv Q\,/\,\eta \Rightarrow -k^2 \varPsi = 4\pi G a^2 \mu (a,k) \rho _M \varDelta _M. \end{aligned}$$

Weak lensing and the integrated Sachs–Wolfe (ISW) effect, on the other hand, are measuring $(\varPhi +\varPsi )/2$, which is related to the density field via

I.3.28 $$\begin{aligned} \varSigma \equiv \frac{1}{2}Q(1+1/\eta ) = \frac{1}{2}\mu (\eta +1) \Rightarrow -k^2 (\varPhi +\varPsi ) = 8\pi G a^2 \varSigma (a,k) \rho _M \varDelta _M. \end{aligned}$$

A summary of different other variables used was given by Daniel et al. (2010). For instance, the gravitational slip parameter introduced by Caldwell et al. (2007) and widely used is related through $\varpi \equiv 1/\eta -1$. Recently, Daniel and Linder (2010) used $\{{{\mathcal {G}}}\equiv \varSigma ,\ \mu \equiv Q,\ {{\mathcal {V}}}\equiv \mu \}$, while (Bean and Tangmatitham 2010) defined $R\equiv 1/\eta$. All these variables reflect the same two degrees of freedom additional to the linear growth of structure in $\varLambda$CDM.

Any combination of two variables out of $\{Q,\eta ,\mu ,\varSigma ,\ldots \}$ is a valid alternative to $(Q,\eta )$. It turns out that the pair $(\mu ,\varSigma )$ is particularly well suited when CMB, WL and LSS data are combined as it is less correlated than others (see Zhao et al. 2010; Daniel and Linder 2010; Axelsson et al. 2014).

**I.3.2.2 Parameterizations and non-parametric approaches**

So far we have defined two free functions that can encode any departure of the growth of linear perturbations from $\varLambda$CDM. However, these free functions are not measurable, but have to be inferred via their impact on the observables. Therefore, one needs to specify a parameterization of, e.g., $(Q,\eta )$ such that departures from $\varLambda$CDM can be quantified. Alternatively, one can use non-parametric approaches to infer the time and scale-dependence of the modified growth functions from the observations.

Ideally, such a parameterization should be able to capture all relevant physics with the least number of parameters. Useful parameterizations can be motivated by predictions for specific theories of MG/DE (see Song et al. 2010) and/or by pure simplicity and measurability (see Amendola et al. 2008b). For instance, Zhao et al. (2010) and Daniel et al. (2010) use scale-independent parameterizations that model one or two smooth transitions of the modified growth parameters as a function of redshift. Bean and Tangmatitham (2010) also adds a scale dependence to the parameterization, while keeping the time-dependence a simple power law:

I.3.29 $$\begin{aligned} Q(a,k)\equiv & {} 1 + \left[ Q_0e^{-k/k_c} + Q_\infty \left( 1-e^{-k/k_c}\right) -1\right] \,a^s, \nonumber \\ \eta (a,k)^{-1}\equiv & {} 1 + \left[ R_0e^{-k/k_c} + R_\infty \left( 1-e^{-k/k_c}\right) -1\right] \,a^s, \end{aligned}$$

with constant $Q_0$, $Q_\infty$, $R_0$, $R_\infty$, *s* and $k_c$. Generally, the problem with any kind of parameterization is that it is difficult—if not impossible—for it to be flexible enough to describe all possible modifications.

Daniel et al. (2010) and Daniel and Linder (2010) investigate the modified growth parameters binned in *z* and *k*. The functions are taken constant in each bin. This approach is simple and only mildly dependent on the size and number of the bins. However, the bins can be correlated and therefore the data might not be used in the most efficient way with fixed bins. Slightly more sophisticated than simple binning is a principal component analysis (PCA) of the binned (or pixelized) modified growth functions. In PCA uncorrelated linear combinations of the original pixels are constructed. In the limit of a large number of pixels the model dependence disappears. At the moment however, computational cost limits the number of pixels to only a few. Zhao et al. (2009a, 2010) employ a PCA in the $(\mu ,\eta )$ plane and find that the observables are more strongly sensitive to the scale-variation of the modified growth parameters rather than the time-dependence and their average values. This suggests that simple, monotonically or mildly-varying parameterizations as well as only time-dependent parameterizations are poorly suited to detect departures from $\varLambda$CDM.

**I.3.2.3 Trigger relations**

A useful and widely popular trigger relation is the value of the growth index $\gamma$ in $\varLambda$CDM. It turns out that the value of $\gamma$ can also be fitted also for simple DE models and sub-Hubble evolution in some MG models (see, e.g., Linder 2005, 2009; Huterer and Linder 2007; Linder and Cahn 2007; Nunes and Mota 2006; Ferreira and Skordis 2010). For example, for a non-clustering perfect fluid DE model with equation of state *w*(*z*) the growth factor *G*(*a*) given in (I.3.21) with the fitting formula

I.3.30 $$\begin{aligned} \gamma = 0.55 + 0.05\left[ 1+w(z=1)\right] \end{aligned}$$

is accurate to the $10^{-3}$ level compared with the actual solution of the growth Eq. (I.3.20). Generally, for a given solution of the growth equation the growth index can simply be computed using

I.3.31 $$\begin{aligned} \gamma (a,k) = \frac{\ln \left( \varDelta _M'\right) -\ln \varDelta _M}{\ln \varOmega _M(a)}. \end{aligned}$$

The other way round, the modified gravity function $\mu$ can be computed for a given $\gamma$ (Pogosian et al. 2010)

I.3.32 $$\begin{aligned} \mu = \frac{2}{3}\varOmega _M^{\gamma -1}(a) \left[ \varOmega _M^\gamma (a) +2 +(\ln H)' -3\gamma +\gamma '\ln \gamma \right] . \end{aligned}$$

The fact that the value of $\gamma$ is quite stable in most DE models but strongly differs in MG scenarios means that a large deviation from $\gamma _\varLambda$ signifies the breakdown of GR, a substantial DE clustering or a breakdown of another fundamental hypothesis like near-homogeneity. Furthermore, using the growth factor to describe the evolution of linear structure is a very simple and computationally cheap way to carry out forecasts and compare theory with data. However, several drawbacks of this approach can be identified:

- As only one additional parameter is introduced, a second parameter, such as $\eta$, is needed to close the system and be general enough to capture all possible modifications.
- The growth factor is a solution of the growth equation on sub-Hubble scales and, therefore, is not general enough to be consistent on all scales.
- The framework is designed to describe the evolution of the matter density contrast and is not easily extended to describe all other energy–momentum components and integrated into a CMB-Boltzmann code.

#### Phantom crossing

In this section, we pay attention to the evolution of the perturbations of a general dark-energy fluid with an evolving equation of state parameter *w*. Current limits on the equation of state parameter $w=p{/}\rho$ of the dark energy indicate that $p\approx -\rho$, and so do not exclude $p<-\rho$, a region of parameter space often called *phantom energy*. Even though the region for which $w<-\,1$ may be unphysical at the quantum level, it is still important to probe it, not least to test for coupled dark energy and alternative theories of gravity or higher dimensional models that can give rise to an effective or apparent phantom energy.

Although there is no problem in considering $w<-\,1$ for the background evolution, there are apparent divergences appearing in the perturbations when a model tries to cross the limit $w=-\,1$. This is a potential headache for experiments like Euclid that directly probe the perturbations through measurements of the galaxy clustering and weak lensing. To analyze the Euclid data, we need to be able to consider models that cross the phantom divide $w=-\,1$ at the level of first-order perturbations (since the only dark-energy model that has no perturbations at all is the cosmological constant).

However, at the level of cosmological first-order perturbation theory, there is no fundamental limitation that prevents an effective fluid from crossing the phantom divide.

As $w \rightarrow -1$ the terms in Eqs. (I.3.17) and (I.3.18) containing $1/(1+w)$ will generally diverge. This can be avoided by replacing $\theta$ with a new variable *V* defined via $V=\rho \left( 1+w \right) \theta$. This corresponds to rewriting the 0-*i* component of the energy momentum tensor as $ik_j T_{0}^{j}= V$, which avoids problems if $T_{0}^{j}\ne 0$ when $\bar{p}=-\,{\bar{\rho }}$. Replacing the time derivatives by a derivative with respect to the logarithm of the scale factor $\ln a$ (denoted by a prime), we obtain (Ma and Bertschinger 1995; Hu 2004; Kunz and Sapone 2006):

I.3.33 $$\begin{aligned} \delta '= & {} 3(1+w) \varPhi ' - \frac{V}{H a} - 3 \left( \frac{\delta p}{{\bar{\rho }}}-w \delta \right) \end{aligned}$$

I.3.34 $$\begin{aligned} V'= & {} -(1-3w) V+ \frac{k^2}{H a} \frac{\delta p}{{\bar{\rho }}} +(1+w) \frac{k^2}{H a}\left( \varPsi -\pi \right) . \end{aligned}$$

In order to solve Eqs. (I.3.33) and (I.3.34) we still need to specify the expressions for $\delta p$ and $\pi$, quantities that characterize the physical, intrinsic nature of the dark-energy fluid at first order in perturbation theory. While in general the anisotropic stress plays an important role as it gives a measure of how the gravitational potentials $\varPhi$ and $\varPsi$ differ, we will set it in this section to zero, $\pi =0$. Therefore, we will focus on the form of the pressure perturbation. There are two important special cases: barotropic fluids,^2^ which have no internal degrees of freedom and for which the pressure perturbation is fixed by the evolution of the average pressure, and non-adiabatic fluids like, e.g., scalar fields for which internal degrees of freedom can change the pressure perturbation.

**I.3.3.1 Parameterizing the pressure perturbation**

Barotropic fluids.

We define a fluid to be barotropic if the pressure *p* depends strictly only on the energy density $\rho$: $p=p(\rho )$. These fluids have only adiabatic perturbations, so that they are often called adiabatic. We can write their pressure as

I.3.35 $$\begin{aligned} p(\rho ) = p(\bar{\rho }+\delta \rho ) = p(\bar{\rho }) + \left. \frac{\mathrm {d}p}{{\mathrm {d}}\rho }\right| _{\bar{\rho }} \delta \rho + O\left[ (\delta \rho )^2\right] . \end{aligned}$$

Here $p({\bar{\rho }}) = \bar{p}$ is the pressure of the isotropic and homogeneous part of the fluid. The second term in the expansion (I.3.35) can be re-written as

I.3.36 $$\begin{aligned} \left. \frac{\mathrm {d}p}{\mathrm {d}\rho }\right| _{\bar{\rho }} = \frac{\dot{\bar{p}}}{\dot{\bar{\rho }}} = w - \frac{\dot{w}}{3 aH(1+w)} \equiv c_a^2, \end{aligned}$$

where we used the equation of state and the conservation equation for the dark-energy density in the background. We notice that the adiabatic sound speed $c_a^2$ will necessarily diverge for any fluid where *w* crosses $-\,1$.

However, for a perfect barotropic fluid the adiabatic sound speed $c_{a}^2$ turns out to be the physical propagation speed of perturbations. Therefore, it should never be negative ($c_{a}^{2}<0$)—otherwise classical, and possible quantum, instabilities appear (superluminal propagation, $c_{a}^{2}>1$, may be acceptable as the fluid is effectively a kind of ether that introduces a preferred frame, see Babichev et al. 2008). Even worse, the pressure perturbation

I.3.37 $$\begin{aligned} \delta p = c_{a}^{2} \delta \rho = \left( w - \frac{\dot{w}}{3 aH(1+w)} \right) \delta \rho \end{aligned}$$

will necessarily diverge if *w* crosses $-\,1$ and $\delta \rho \ne 0$. Even if we find a way to stabilize the pressure perturbation, for instance an equation of state parameter that crosses the $-\,1$ limit with zero slope ($\dot{w}$), there will always be the problem of a negative speed of sound that prevents these models from being viable dark-energy candidates (Vikman 2005; Kunz and Sapone 2006).

Non-adiabatic fluids

To construct a model that can cross the phantom divide, we therefore need to violate the constraint that *p* is a unique function of $\rho$. At the level of first-order perturbation theory, this amounts to changing the prescription for $\delta p$, which now becomes an arbitrary function of *k* and *t*. One way out of this problem is to choose an appropriate gauge where the equations are simple; one choice is, for instance, the rest frame of the fluid where the pressure perturbation reads (in this frame)

I.3.38 $$\begin{aligned} \hat{\delta p} = \hat{c}_{s}^{2}\hat{\delta \rho }, \end{aligned}$$

where now the $\hat{c}_{s}^{2}$ is the speed with which fluctuations in the fluid propagate, i.e., the sound speed. We can write Eq. (I.3.38), with an appropriate gauge transformation, in a form suitable for the Newtonian frame, i.e., for Eqs. (I.3.33) and (I.3.34). We find that the pressure perturbation is given by (Erickson et al. 2002; Bean and Dore 2004; Carturan and Finelli 2003)

I.3.39 $$\begin{aligned} \delta p = \hat{c}_s^2 \delta \rho + 3 a H\left( a\right) \left( \hat{c}_s^2 - c_{a}^{2}\right) \bar{\rho }\frac{V}{k^2}. \end{aligned}$$

The problem here is the presence of $c_{a}^2$, which goes to infinity at the crossing and it is impossible that this term stays finite except if $V\rightarrow 0$ fast enough or $\dot{w}=0$, but this is not, in general, the case.

This divergence appears because for $w=-\,1$ the energy momentum tensor Eq. (I.3.3) reads $T^{\mu \nu }=pg^{\mu \nu }$. Normally the four-velocity $u^{\mu }$ is the time-like eigenvector of the energy–momentum tensor, but now all vectors are eigenvectors. So the problem of fixing a unique rest-frame is no longer well posed. Then, even though the pressure perturbation looks fine for *the observer in the rest-frame*, because it does not diverge, the badly-defined gauge transformation to the Newtonian frame does, as it also contains $c_{a}^{2}$.

**I.3.3.2 Regularizing the divergences**

We have seen that neither barotropic fluids nor canonical scalar fields, for which the pressure perturbation is of the type (I.3.39), can cross the phantom divide. However, there is a simple model (called the quintom model Feng et al. 2005; Hu 2005) consisting of two fluids of the same type as in the previous Sect. I.3.3.1 but with a constant *w* on either side of $w=-\,1$.^3^ The combination of the two fluids then effectively crosses the phantom divide if we start with $w_{\mathrm {tot}}>-\,1$, as the energy density in the fluid with $w<-\,1$ will grow faster, so that this fluid will eventually dominate and we will end up with $w_{\mathrm {tot}}<-\,1$.

The perturbations in this scenario were analyzed in detail in Kunz and Sapone (2006), where it was shown that in addition to the rest-frame contribution, one also has relative and non-adiabatic perturbations. All these contributions apparently diverge at the crossing, but their sum stays finite. When parameterizing the perturbations in the Newtonian gauge as

I.3.40 $$\begin{aligned} \delta p(k,t) = \gamma (k,t)\, \delta \rho (k,t) \end{aligned}$$

the quantity $\gamma$ will, in general, have a complicated time and scale dependence. The conclusion of the analysis is that indeed single canonical scalar fields with pressure perturbations of the type (I.3.39) in the Newtonian frame cannot cross $w=-\,1$, but that this is not the most general case. More general models have a priori no problem crossing the phantom divide, at least not with the classical stability of the perturbations.

Kunz and Sapone (2006) found that a good approximation to the quintom model behavior can be found by regularizing the adiabatic sound speed in the gauge transformation with

I.3.41 $$\begin{aligned} c_a^2 = w - \frac{\dot{w}(1+w)}{3 H a [(1+w)^2 + \lambda ]} \end{aligned}$$

where $\lambda$ is a tunable parameter which determines how close to $w=-\,1$ the regularization kicks in. A value of $\lambda \approx 1/1000$ should work reasonably well. However, the final results are not too sensitive on the detailed regularization prescription.

This result appears also related to the behavior found for coupled dark-energy models (originally introduced to solve the coincidence problem) where dark matter and dark energy interact not only through gravity (Amendola 2000a). The effective dark energy in these models can also cross the phantom divide without divergences (Huey and Wandelt 2006; Das et al. 2006; Kunz 2009).

The idea is to insert (by hand) a term in the continuity equations of the two fluids

I.3.42 $$\begin{aligned}&\dot{\rho }_{M}+3H\rho _{M}=\lambda \end{aligned}$$

I.3.43 $$\begin{aligned}&\dot{\rho }_{x}+3H\left( 1+w_{x}\right) \rho _{x}=-\,\lambda , \end{aligned}$$

where the subscripts *m*, *x* refer to dark matter and dark energy, respectively. In this approximation, the adiabatic sound speed $c_{a}^{2}$ reads

I.3.44 $$\begin{aligned} c_{a,x}^{2}= \frac{\dot{p}_{x}}{\dot{\rho }_{x}} = w_{x}-\frac{\dot{w_{x}}}{3aH\left( 1+w_{x}\right) + \lambda /\rho _{x}}, \end{aligned}$$

which stays finite at crossing as long as $\lambda \ne 0$.

However in this class of models there are other instabilities arising at the perturbation level regardless of the coupling used, (cf. Väliviita et al. 2008).

*I.3.3.3 A word on perturbations when* $w=-\,1$

Although a cosmological constant has $w=-\,1$ and no perturbations, the converse is not automatically true: $w=-\,1$ does not necessarily imply that there are no perturbations. It is only when we set from the beginning (in the calculation):

I.3.45 $$\begin{aligned} p= & {} -\rho \end{aligned}$$

I.3.46 $$\begin{aligned} \delta p= & {} -\delta \rho \end{aligned}$$

I.3.47 $$\begin{aligned} \pi= & {} 0, \end{aligned}$$

i.e., $T^{\mu \nu } \propto g^{\mu \nu }$, that we have as a solution $\delta = V =0$.

For instance, if we set $w=-\,1$ and $\delta p = \gamma \delta \rho$ (where $\gamma$ can be a generic function) in Eqs. (I.3.33) and (I.3.34) we have $\delta \ne 0$ and $V\ne 0$. However, the solutions are decaying modes due to the $-\frac{1}{a}\left( 1-3w\right) V$ term so they are not important at late times; but it is interesting to notice that they are in general not zero.

As another example, if we have a non-zero anisotropic stress $\pi$ then the Eqs. (I.3.33) and (I.3.34) will have a source term that will influence the growth of $\delta$ and *V* in the same way as $\varPsi$ does (just because they appear in the same way). The $\left( 1+w\right)$ term in front of $\pi$ should not worry us as we can always define the anisotropic stress through

I.3.48 $$\begin{aligned} \rho \left( 1+w\right) \pi = -\,\left( \hat{k}_{i}\hat{k}_{j}-\frac{1}{3}\delta _{ij}\right) \varPi ^{i}_{\,j}, \end{aligned}$$

where $\varPi ^{i}_{\,j}\ne 0$ when $i\ne j$ is the *real* traceless part of the energy momentum tensor, probably the quantity we need to look at: as in the case of $V=(1+w) \theta$, there is no need for $\varPi \propto (1+w)\pi$ to vanish when $w=-\,1$.

It is also interesting to notice that when $w = -\,1$ the perturbation equations tell us that dark-energy perturbations are not influenced through $\varPsi$ and $\varPhi '$ [see Eqs. (I.3.33) and (I.3.34)]. Since $\varPhi$ and $\varPsi$ are the quantities directly entering the metric, they must remain finite, and even much smaller than 1 for perturbation theory to hold. Since, in the absence of direct couplings, the dark energy only feels the other constituents through the terms $(1+w)\varPsi$ and $(1+w)\varPhi '$, it decouples completely in the limit $w=-\,1$ and just evolves on its own. But its perturbations still enter the Poisson equation and so the dark matter perturbation will feel the effects of the dark-energy perturbations.

Although this situation may seem contrived, it might be that the acceleration of the universe is just an observed effect as a consequence of a modified theory of gravity. As was shown in Kunz and Sapone (2007), any modified gravity theory can be described as an effective fluid both at background and at perturbation level; in such a situation it is imperative to describe its perturbations properly as this effective fluid may manifest unexpected behavior.

### Generic properties of dark energy and modified gravity models

This section explores some generic issues that are not necessarily a feature of any particular model. We will recall the properties of particular classes of models as examples, leaving the details of the model description to Sect. I.5.

We begin by discussing the general implications of modelling dark energy as an extra degree of freedom, instead of the cosmological constant. We then discuss how the literature tends to categorize models into models of dark energy and models of modified gravity. We focus on the expansion of the cosmological background and ask what precision of measurement is necessary in order to make definite statements about large parts of the interesting model space. Then we address the issue of dark-energy perturbations, their impact on observables and how they can be used to distinguish between different classes of models. Finally, we present some general consistency relations among the perturbation variables that all models of modified gravity should fulfill.

#### Dark energy as a degree of freedom

De Sitter spacetime, filled with only a cosmological constant, is static, undergoes no evolution. It is also invariant under Lorentz transformations. When other sources of energy–momentum are added into this spacetime, the dynamics occurs on top of this static background, or better to say—vacuum. This is to say that the cosmological constant is a form of dark energy which has no dynamics of its own and the value of which is fixed for all frames and coordinate choices.

A dynamical model for acceleration implies the existence of some change of the configuration in space or time. It is no longer a gravitational vacuum. In the case of a perfectly homogeneous and isotropic universe, the evolution can only be a function of time. In reality, the universe has neither of these properties and therefore the configuration of any dynamical dark energy *must* also be inhomogeneous. Whether the inhomogeneities are small is a model-dependent statement.

It is important to stress that there exists *no* such thing as a modified gravity theory with no extra degrees of freedom beyond the metric. All models which seemingly involve just the metric degrees of freedom in some modified sense (say *f*(*R*) or *f*(*G*)), in fact can be shown to be equivalent to general relativity plus an extra scalar degree of freedom with some particular couplings to gravity (Chiba 2003; Kobayashi et al. 2011). Modifications such as massive gravity increase the number of polarisations.

In the context of $\varLambda$CDM, it has proven fruitful to consider the dynamics of the universe in terms of a perturbation theory: a separation into a background, linear and then higher-order fluctuations, each of increasingly small relevance (see Sect. I.3.1). These perturbations are thought to be seeded with a certain amplitude by an inflationary era at early times. Gravitational collapse then leads to a growth of the fluctuations, eventually leading to a breakdown of the perturbation theory; however, for dark matter in $\varLambda$CDM, this growth is only large enough to lead to non-linearity at smaller scales.

When dynamical DE is introduced, it must be described by at least one new (potentially more) degree of freedom. In principle, in order to make any statements about any such theory, one must specify the initial conditions on some space-like hypersurface and then the particular DE model will describe the subsequent evolutionary history. Within the framework of perturbation theory, initial conditions must be specified for *both* the background and the fluctuations. The model then provides a set of *related* evolution equations at each order.

We defer the discussion of the freedom allowed at particular orders to the appropriate sections below (Sect. I.4.3 for the background, Sect. I.4.4 for the perturbations). Here, let us just stress that since DE is a full degree of freedom, its initial conditions will contain both adiabatic and isocurvature modes, which may or may not be correlated, depending on their origin and which may or may not survive until today, depending on the particular model. Secondly, the non-linearity in the DE configuration is in principle independent of the non-linearity in the distribution of dark matter and will depend on both the particular model and the initial conditions. For example, the chameleon mechanism present in many non-minimally coupled models of dark energy acts to break down DE perturbation theory in higher-density environments (see Sect. I.5.8). This breakdown of linear theory is environment-dependent and only indirectly related to non-linearities in the distribution of dark matter.

Let us underline that the absolute and *unique* prediction of $\varLambda$CDM is that $\varLambda$ is constant in space and time and therefore does not contribute to fluctuations at any order. Any violation of this statement at any one order, if it cannot be explained by astrophysics, is sufficient evidence that the acceleration is not caused by vacuum energy.

#### A definition of modified gravity

In this review we often make reference to DE and MG models. Although in an increasing number of publications a similar dichotomy is employed, there is currently no consensus on where to draw the line between the two classes. Here we will introduce an operational definition for the purpose of this document.

Roughly speaking, what most people have in mind when talking about standard dark energy are models of minimally-coupled scalar fields with standard kinetic energy in 4-dimensional Einstein gravity, the only functional degree of freedom being the scalar potential. Often, this class of model is referred to simply as “quintessence”. However, when we depart from this picture a simple classification is not easy to draw. One problem is that, as we have seen in the previous sections, both at background and at the perturbation level, different models can have the same observational signatures (Kunz and Sapone 2007). This problem is not due to the use of perturbation theory: any modification to Einstein’s equations can be interpreted as standard Einstein gravity with a modified “matter” source, containing an arbitrary mixture of scalars, vectors and tensors (Hu and Sawicki 2007b; Kunz et al. 2008).

Therefore, we could simply abandon any attempt to distinguish between DE and MG, and just analyse different models, comparing their properties and phenomenology. However, there is a possible classification that helps us set targets for the observations, which is often useful in concisely communicating the results of complex arguments. In this review, we will use the following notation:

- *Standard dark energy* These are models in which dark energy lives in standard Einstein gravity *and* does not cluster appreciably on sub-horizon scales and does not carry anisotropic stress. As already noted, the prime example of a standard dark-energy model is a minimally-coupled scalar field with standard kinetic energy, for which the sound speed equals the speed of light.
- *Clustering dark energy* In clustering dark-energy models, there is an additional contribution to the Poisson equation due to the dark-energy perturbation, which induces $Q \ne 1$. However, in this class we require $\eta =1$, i.e., no extra effective anisotropic stress is induced by the extra dark component. A typical example is a k-essence model with a low sound speed, $c_s^2\ll 1$.
- *Modified gravity models* These are models where from the start the Einstein equations are modified, for example scalar–tensor and *f*(*R*) type theories, Dvali–Gabadadze–Porrati (DGP) as well as interacting dark energy, in which effectively a fifth force is introduced in addition to gravity. Generically they change the clustering and/or induce a non-zero anisotropic stress. Since our definitions are based on the phenomenological parameters, we also add dark-energy models that live in Einstein’s gravity but that have non-vanishing anisotropic stress into this class since they cannot be distinguished by cosmological observations.

Notice that both clustering dark energy and explicit modified gravity models lead to deviations from what is often called ‘general relativity’ (or, like here, standard dark energy) in the literature when constraining extra perturbation parameters like the growth index $\gamma$. For this reason we generically call both of these classes MG models. In other words, in this review we use the simple and by now extremely popular (although admittedly somewhat misleading) expression “modified gravity” to denote models in which gravity is modified and/or dark energy clusters or interacts with other fields. Whenever we feel useful, we will remind the reader of the actual meaning of the expression “modified gravity” in this review.

Therefore, on sub-horizon scales and at first order in perturbation theory our definition of MG is straightforward: models with $Q=\eta =1$ (see Eq. I.3.23) are standard DE, otherwise they are MG models. In this sense the definition above is rather convenient: we can use it to quantify, for instance, how well Euclid will distinguish between standard dynamical dark energy and modified gravity by forecasting the errors on $Q,\eta$ or on related quantities like the growth index $\gamma$.

On the other hand, it is clear that this definition is only a practical way to group different models and should not be taken as a fundamental one. We do not try to set a precise threshold on, for instance, how much dark energy should cluster before we call it modified gravity: the boundary between the classes is therefore left undetermined but we think this will not harm the understanding of this document.

#### The background: to what precision should we measure *w*?

The effect of dark energy on background expansion is to add a new source of energy density. The chosen model will have dynamics which will cause the energy density to evolve in a particular manner. On the simplest level, this evolution is a result of the existence of intrinsic hydrodynamical pressure of the dark energy fluid which can be described by the instantaneous equation of state. Alternatively, an interaction with other species can result in a non-conservation of the DE EMT and therefore change the manner in which energy density evolves (e.g. coupled dark energy). Taken together, all these effects add up to result in an *effective* equation of state for DE which drives the expansion history of the universe.

It is important to stress that all background observables are geometrical in nature and therefore can only be measurements from *curvatures*. It is not possible to disentangle the dark energy and dark matter in a model independent matter and therefore only the measurement of the Hubble parameter up to a normalization factor, $H(z)/H_0$, and the spatial curvature $\varOmega _{k0}$ can be obtained in a DE-model independent manner. In particular, the measurement of the dark-matter density, $\varOmega _{m0}$, becomes possible only on choosing some parameterization for $w_\text {eff}$ (e.g., a constant) (Amendola et al. 2013a). One must therefore always be mindful that extracting DE properties from background measurements is limited to constraining the coefficients of a chosen *parameterization* of the *effective* equation of state for the DE component, rather than being measurements of the actual effective *w* and definitely not the intrinsic *w* of the dark energy.

Given the above complications, two crucial questions are often asked in the context of dark-energy surveys:

- Since current measurements of the expansion history appear so consistent with $w=-\,1$, do we not already know that the dark energy is a cosmological constant?
- To which precision should we measure *w*? Or equivalently, why is the Euclid target precision of about 0.01 on $w_0$ and 0.1 on $w_a$ interesting?

We will now attempt to answer these questions at least partially. First, we address the question of what the measurement of *w* can tell us about the viable model space of DE. Then we examine whether we can draw useful lessons from inflation. Finally, we will look at what we can learn from arguments based on Bayesian model comparison.

In the first part, we will argue that whereas any detection of a deviation from $\varLambda$CDM expansion history immediately implies that acceleration is not driven by a cosmological constant, the converse is not true, even if $w=-\,1$ exactly. We will also argue that a detection of a phantom equation of state, $w<-\,1$, would reveal that gravity is not minimally coupled or that dark energy interacts and immediately eliminate the perfect-fluid models of dark energy, such as quintessence.

**Fig. 1:**
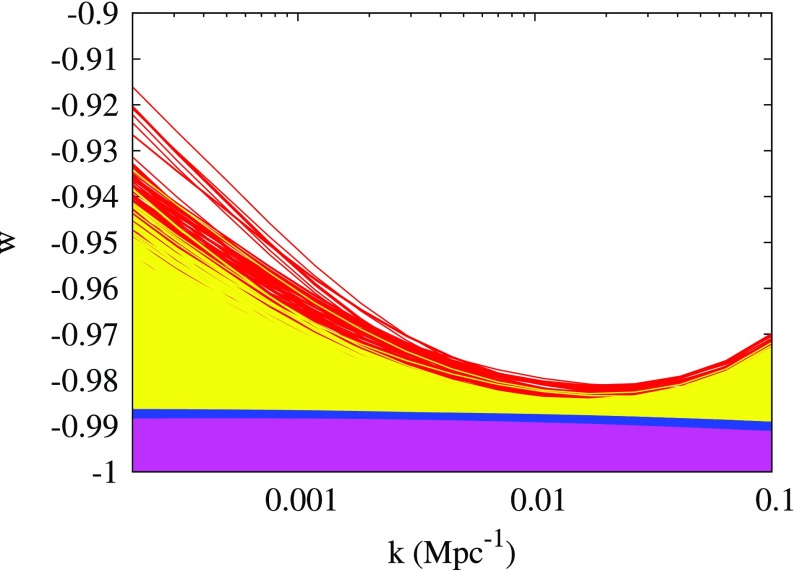
The evolution of *w* as a function of the comoving scale *k*, using only the 5-year WMAP CMB data. Red and yellow are the 95 and 68% confidence regions for the LV formalism. Blue and purple are the same for the flow-equation formalism. From the outside inward, the colored regions are red, yellow, blue, and purple. Image reproduced by permission from Ilić et al. (2010); copyright by APS

Then we will see that for single field slow-roll inflation models we effectively measure $w \sim -\,1$ with percent-level accuracy (see Fig. 1); however, the deviation from a scale-invariant spectrum means that we nonetheless observe a dynamical evolution and, thus, a deviation from an exact and constant equation of state of $w=-\,1$. Therefore, we know that inflation was not due to a cosmological constant; we also know that we can see no deviation from a de Sitter expansion for a precision smaller than the one Euclid will reach.

In the final part, we will consider the Bayesian evidence in favor of a true cosmological constant if we keep finding $w=-\,1$; we will see that for priors on $w_0$ and $w_a$ of order unity, a precision like the one for Euclid is necessary to favor a true cosmological constant decisively. We will also discuss how this conclusion changes depending on the choice of priors.

*I.4.3.1 What can a measurement of* *w* *tell us?*

The prediction of $\varLambda$CDM is that $w=-\,1$ exactly at all times. Any detection of a deviation from this result immediately disqualifies the cosmological constant as a model for dark energy.

The converse is not true, however. Simplest models of dynamical dark energy, such as quintessence (Sect. I.5.1) can approach the vacuum equation of state arbitrarily closely, given sufficiently flat potentials and appropriate initial conditions. An equation of state $w=-\,1$ at all times is inconsistent with these models, but this may never be detectable.

Moreover, there exist classes of models, e.g., shift-symmetric k-*essence* with de-Sitter attractors, which have equation of state $w=-\,1$ exactly, once the attractor is approached. Despite this, the acceleration is not at all driven by a cosmological constant, but by a perturbable fluid which has vanishing sound speed and can cluster. Such models can only be differentiated from a cosmological constant by the measurements of perturbations, if at all, see Sect. I.4.4.

Beyond eliminating the cosmological constant as a mechanism for acceleration, measuring $w>-\,1$ is not by itself very informative as to the nature of dark energy. Essentially all classes of models can evolve with such an equation of state given appropriate initial conditions (which is *not* to say that any evolution history can be produced by any class of models). On the other hand, the observation of a phantom equation of state, $w<-\,1$, at any one moment in time is hugely informative as to the nature of gravitational physics. It is well known that any such background made up of either a perfect fluid or a minimally coupled scalar field suffers from gradient instabilities, ghosts or both (Dubovsky et al. 2006). Therefore such an observation immediately implies that either gravity is non-minimally coupled and therefore there is a fifth force, that dark energy is not a perfect fluid, that dark energy interacts with other species, or that dynamical ghosts are not forbidden by nature, perhaps being stabilized by a mechanism such as ghost condensation (Arkani-Hamed et al. 2004b). Any of these would provide a discovery in itself as significant as excluding a cosmological constant.

In conclusion, we aim to measure *w* since it is the most direct way of disproving that acceleration is caused by a cosmological constant. However, if it turns out that no significant deviation can be detected this does not imply that the cosmological constant is the mechanism for dark energy. The clustering properties must then be verified and found to not disagree with $\varLambda$CDM predictions.

*I.4.3.2 Lessons from inflation*

In all probability the observed late-time acceleration of the universe is not the first period of accelerated expansion that occurred during its evolution: the current standard model of cosmology incorporates a much earlier phase with $\ddot{a}>0$, called inflation. Such a period provides a natural mechanism for generating several properties of the universe: spatial flatness, gross homogeneity and isotropy on scales beyond naive causal horizons and nearly scale-invariant initial fluctuations.

The first lesson to draw from inflation is that it cannot have been due to a pure cosmological constant. This is immediately clear since inflation actually ended and therefore there had to be some sort of time evolution. We can go even further: since de Sitter spacetime is static, no curvature perturbations are produced in this case (the fluctuations are just unphysical gauge modes) and therefore an exactly scale-invariant power spectrum would have necessitated an alternative mechanism.

The results obtained by the Planck collaboration from the first year of data imply that the initial spectrum of fluctuations is not scale invariant, but rather has a tilt given by $n_\text {s} = 0.9608\pm 0.0054$ and is consistent with no running and no tensor modes (Planck Collaboration 2014a). This is consistent with the final results from WMAP (Hinshaw et al. 2013). It is surprisingly difficult to create this observed fluctuation spectrum in alternative scenarios that are strictly causal and only act on sub-horizon scales (Spergel and Zaldarriaga 1997; Scodeller et al. 2009).

Let us now translate what the measured properties of the initial power spectrum of fluctuations imply for a observer existing during the inflationary period. We will assume that inflation was driven by one of the simple models (i.e., with sound speed $c_\text {s}=1$). Following the analysis in Ilić et al. (2010), we notice that

I.4.1 $$\begin{aligned} 1+w = - \frac{2}{3} \frac{\dot{H}}{H^2} = \frac{2}{3} \varepsilon _H , \end{aligned}$$

where $\epsilon _H \equiv 2 M^2_{\mathrm {Pl}}(H'{/}H)^2$ and where the prime denotes a derivative with respect to the inflaton field.

This equation of state is directly related to the tensor-to-scalar ratio through $r \sim 24(1+w)$. Since no tensor modes have been detected thus far, no deviation from $w=-\,1$ has been seen either. In fact the Planck 95% limit is $r\lesssim 0.1$ implying that $1+w\lesssim 0.04$. Moreover, the spectral tilt itself is also related to the rate of change of *w*,

I.4.2 $$\begin{aligned} \frac{d \ln (1+w)}{d N}&= 2 (\eta _H - \varepsilon _H) \nonumber \\ 2\eta _H&= (n_s-1)+4\varepsilon _H \end{aligned}$$

where $\eta _H \equiv 2 M^2_{\mathrm {Pl}} H''/H$. Thus, if $n_s\ne 1$ we have that either $\eta _H\ne 0$ or $\varepsilon _H\ne 0$, and consequently either $w\ne -1$ or *w* is not constant at the pivot scale.

We can rewrite Eq. (I.4.1) as

I.4.3 $$\begin{aligned} (1+w) = -\, \frac{1}{6} (n_s-1) + \frac{\eta _H}{3} \approx 0.007 + \frac{\eta _H}{3}. \end{aligned}$$

Without tuning, it is natural for $\eta _H\sim \mathcal {O}(\varepsilon _H^2)$. However, classes of models exist where $\eta _H\sim \varepsilon _H$. Thus, given the observations of the scale dependence of the initial curvature fluctuations, we can conclude that $1+w$ should lie between 0.005 and 0.04, which is well within the current experimental bounds on the DE equation of state and roughly at the limit of Euclid’s sensitivity. We have plotted the allowed values of *w* as a function of scale in Fig. 1.

**Fig. 2:**
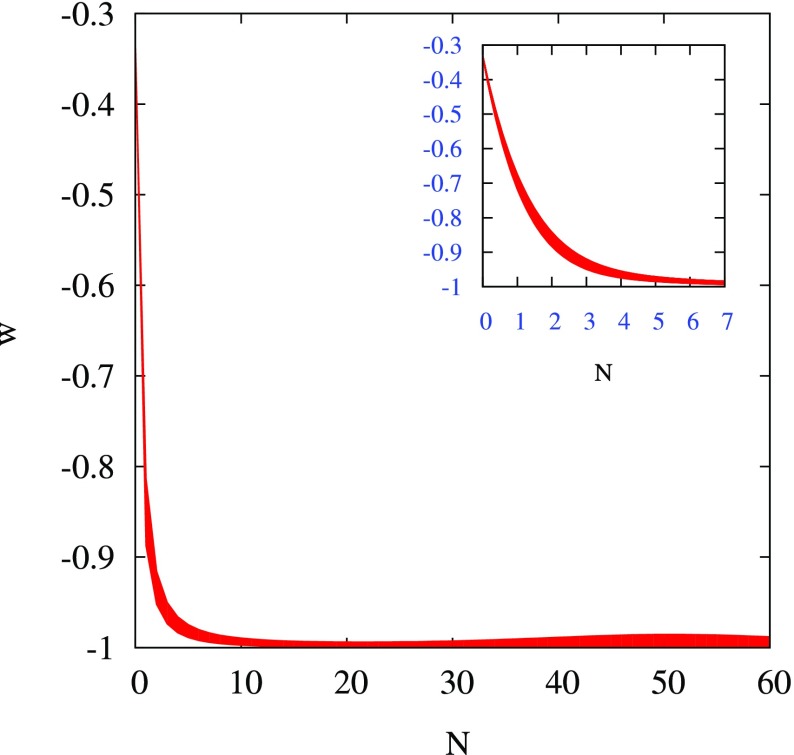
The complete evolution of *w*(*N*), from the flow-equation results accepted by the CMB likelihood. Inflation is made to end at $N=0$ where $w(N=0)=-\,1/3$ corresponding to $\epsilon _H(N=0)=1$. For our choice of priors on the slow-roll parameters at $N=0$, we find that *w* decreases rapidly towards $-\,1$ (see inset) and stays close to it during the period when the observable scales leave the horizon ($N\approx 40\textendash 60$). Image reproduced by permission from Ilić et al. (2010); copyright by APS

We should note that there are classes of models where the cancellation between $\eta _H$ and the tilt in Eq. (I.4.3) is indeed natural which is why one cannot give a lower limit for the amplitude of primordial gravitational waves and *w* lies arbitrarily close to $-\,1$. On the other hand, the observed period of inflation is probably in the middle of a long slow-roll phase. By Eq. (I.4.2), this cancellation would only happen at one moment in time. We have plotted the typical evolution of *w* in inflation in Fig. 2.

Despite being the only other physically motivated period of acceleration, inflation does occur at a very different energy scale, between 1 MeV and GUT scale $10^{16}$ GeV, while the energy scale for dark energy is $10^{-3}$ eV. We should therefore be wary about pushing the analogy too far.

*I.4.3.3 When should we stop? Bayesian model comparison*

In Sect. I.4.3.1, we explained that the measurement of the equation of state *w* can exclude some classes of models, including the cosmological constant of $\varLambda$CDM. However, most *classes* of models allow the equation of state to be arbitrarily close to that of vacuum energy, $w=-\,1$, while still representing completely different physics. Since precision cannot be infinite, we need to propose an algorithm to determine how well this property should be measured. As we showed in Sect. I.4.3.2 above, inflation provides an example of a period that acceleration that, if it occurred at late times would have been judged as consistent with $w=-\,1$ given today’s constraints. We therefore should require a better measurement, but how much better?

We approach the answer to this question from the perspective of Bayesian evidence: at what precision does the non-detection of a deviation of the background expansion history signifies that we should prefer the simpler null hypothesis that $w=-\,1$.

In our Bayesian framework, the first model, the null hypothesis $M_0$, posits that the background expansion is due to an extra component of energy density that has equation of state $w=-\,1$ at all times. The other models assume that the dark energy is dynamical in a way that is well parametrized either by an arbitrary constant *w* (model $M_1$) or by a linear fit $w(a)=w_0+(1-a) w_a$ (model $M_2$).

Here we are using the constant and linear parametrization of *w* because on the one hand we can consider the constant *w* to be an effective quantity, averaged over redshift with the appropriate weighting factor for the observable, see Simpson and Bridle (2006), and on the other hand because the precision targets for observations are conventionally phrased in terms of the figure of merit (FoM) given by $1\big /\sqrt{|{\mathrm {Cov}}(w_0,w_a)|}$. We will, therefore, find a direct link between the model probability and the FoM. It would be an interesting exercise to repeat the calculations with a more general model, using e.g. PCA, although we would expect to reach a similar conclusion.

Bayesian model comparison aims to compute the relative model probability

I.4.4 $$\begin{aligned} \frac{P(M_0|d)}{P(M_1|d)} = \frac{P(d|M_0)}{P(d|M_1)} \frac{P(M_0)}{P(M_1)} \end{aligned}$$

where we used Bayes formula and where $B_{01}\equiv P(d|M_0)/P(d|M_1)$ is called the Bayes factor. The Bayes factor is the amount by which our relative belief in the two models is modified by the data, with $\ln B_{01} > 0\ (<0)$ indicating a preference for model 0 (model 1). Since model $M_0$ is nested in $M_1$ at the point $w=-\,1$ and in model $M_2$ at $(w_0=-\,1,w_a=0)$, we can use the Savage–Dickey (SD) density ratio (e.g. Trotta 2007a). Based on SD, the Bayes factor between the two models is just the ratio of posterior to prior at $w=-\,1$ or at $(w_0=-\,1,w_a=0)$, marginalized over all other parameters.

Let us start by following Trotta et al. (2010) and consider the Bayes factor $B_{01}$ between a cosmological constant model $w=-\,1$ and a free but constant effective *w*. If we assume that the data are compatible with $w_{{\mathrm {eff}}}=-\,1$ with an uncertainty $\sigma$, then the Bayes factor in favor of a cosmological constant is given by

I.4.5 $$\begin{aligned} B = \sqrt{\frac{2}{\pi }}\frac{\varDelta _{+} + \varDelta _{-}}{\sigma } \left[ \text {erfc}\left( -\frac{\varDelta _+}{\sqrt{2}\sigma }\right) - \text {erfc}\left( \frac{\varDelta _-}{\sqrt{2}\sigma }\right) \right] ^{-1}, \end{aligned}$$

where for the evolving dark-energy model we have adopted a flat prior in the region $-\,1 - \varDelta _{-} \le w_{{\mathrm {eff}}}\le -1+\varDelta _+$ and we have made use of the Savage–Dickey density ratio formula (see Trotta 2007a). The prior, of total width $\varDelta = \varDelta _+ + \varDelta _-$, is best interpreted as a factor describing the predictivity of the dark-energy model under consideration. In what follows we will consider example benchmark three models as alternatives to $w=-\,1$:

- *Fluid-like*: we assume that the acceleration is driven by a fluid the background configuration of which satisfies both the strong energy condition and the null energy condition, i.e., we have that $\varDelta _+ = 2/3, \varDelta _- = 0$.
- *Phantom*: phantom models violate the null energy condition, i.e., are described by $\varDelta _+ = 0, \varDelta _- > 0$, with the latter being possibly rather large.
- *Small departures*: We assume that the equation of state is very close to that of vacuum energy, as seems to have been the case during inflation: $\varDelta _+ = \varDelta _- = 0.01$.

A model with a large $\varDelta$ will be more generic and less predictive, and therefore is disfavored by the Occam’s razor of Bayesian model selection, see Eq. (I.4.5). According to the Jeffreys’ scale for the strength of evidence, we have a moderate (strong) preference for the cosmological constant model for $2.5< \ln B_{01} < 5.0$ ($\ln B_{01}>5.0$), corresponding to posterior odds of 12:1–150:1 (above 150:1).

**Fig. 3:**
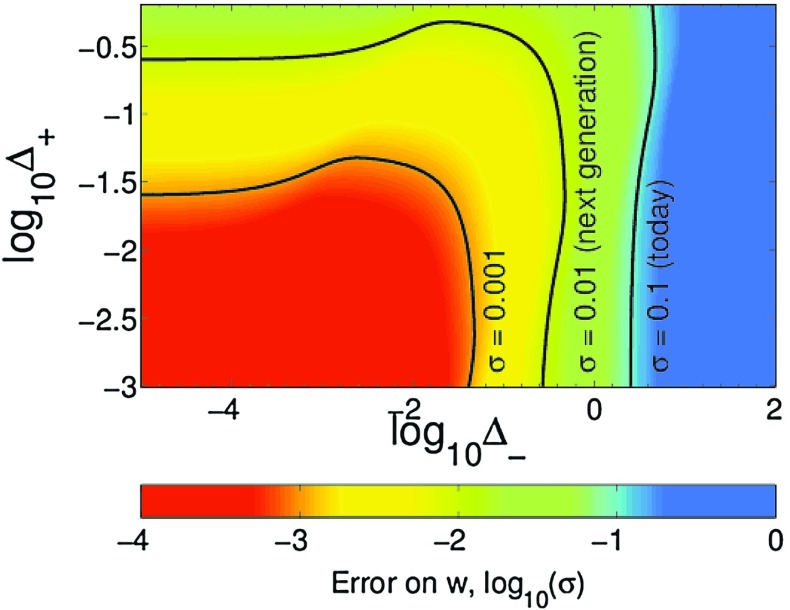
Required accuracy on $w_{{\mathrm {eff}}}= -\,1$ to obtain strong evidence against a model where $-\,1 - \varDelta _{-} \le w_{{\mathrm {eff}}}\le -1+\varDelta _+$ as compared to a cosmological constant model, $w=-\,1$. For a given $\sigma$, models to the right and above the contour are disfavored with odds of more than 20:1

**Table 1:** Strength of evidence disfavoring the three example benchmark models against a $\varLambda$CDM expansion history, using an indicative accuracy on $w=-\,1$ from present data, $\sigma \sim 0.1$

Model	$(\varDelta _+, \varDelta _-)$	$\ln B$ today ($\sigma = 0.1$)
Phantom	(0, 10)	4.4 (strongly disfavored)
Fluid-like	(2 / 3, 0)	1.7 (slightly disfavored)
Small departures	(0.01, 0.01)	0.0 (inconclusive)

**Table 2:** Required precision $\sigma$ of the value of *w* for future surveys in order to disfavor the three benchmark models against $w=-\,1$ for two different strengths of evidence

Model	$(\varDelta _+, \varDelta _-)$	Required $\sigma$ for odds	
		$>\,20:1$	$>\,150:1$
Phantom	(0, 10)	0.4	$5\times 10^{-2}$
Fluid-like	(2 / 3, 0)	$3\times 10^{-2}$	$3\times 10^{-3}$
Small departures	(0.01, 0.01)	$4\times 10^{-4}$	$5\times 10^{-5}$

We plot in Fig. 3 contours of constant observational accuracy $\sigma$ in the model predictivity space $(\varDelta _-,\varDelta _+)$ for $\ln B = 3.0$ from Eq. (I.4.5), corresponding to odds of 20–1 in favor of a cosmological constant (slightly above the “moderate” threshold. The figure can be interpreted as giving the space of extended models that can be significantly disfavored with respect to $w=-\,1$ at a given accuracy. The results for the 3 benchmark models mentioned above (fluid-like, phantom or small departures from $w=-\,1$) are summarized in Table 1. Instead, we can ask the question which precision needs to reached to support $\varLambda$CDM at a given level. This is shown in Table 2 for odds 20:1 and 150:1. We see that to rule out a fluid-like model, which also covers the parameter space expected for canonical scalar field dark energy, we need to reach a precision comparable to the one that the Euclid satellite is expected to attain.

By considering the model $M_2$ we can also provide a direct link with the target DETF FoM: Let us choose (fairly arbitrarily) a flat probability distribution for the prior, of width $\varDelta w_0$ and $\varDelta w_a$ in the dark-energy parameters, so that the value of the prior is $1/(\varDelta w_0 \varDelta w_a)$ everywhere. Let us assume that the likelihood is Gaussian in $w_0$ and $w_a$ and centered on $\varLambda$CDM (i.e., the data fully supports $\varLambda$ as the dark energy).

As above, we need to distinguish different cases depending on the width of the prior. If you accept the argument of the previous section that we expect only a small deviation from $w=-\,1$, and set a prior width of order 0.01 on both $w_0$ and $w_a$, then the posterior is dominated by the prior, and the ratio will be of order 1 if the future data is compatible with $w=-\,1$. Since the precision of the experiment is comparable to the expected deviation, both $\varLambda$CDM and evolving dark energy are equally probable (as argued above and shown for model $M_1$ in Table 1), and we have to wait for a detection of $w\ne -1$ or a significant further increase in precision (cf. the last row in Table 2).

However, one often considers a much wider range for *w*, for example the fluid-like model with $w_0\in [-\,1/3,-\,1]$ and $w_a \in [-\,1,1]$ with equal probability (and neglecting some subtleties near $w=-\,1$). If the likelihood is much narrower than the prior range, then the value of the normalized posterior at $w=-\,1$ will be $2/(2\pi \sqrt{|{\mathrm {Cov}}(w_0,w_a)|}={\mathrm {FoM}}/\pi$ (since we excluded $w<-\,1$, else it would half this value). The Bayes factor is then given by

I.4.6 $$\begin{aligned} B_{01} = \frac{\varDelta w_0 \varDelta w_a {\mathrm {FoM}}}{\pi }. \end{aligned}$$

For the prior given above, we end up with $B_{01} \approx 4 {\mathrm {FoM}}/(3\pi ) \approx 0.4 {\mathrm {FoM}}$. In order to reach a “decisive” Bayes factor, usually characterized as $\ln B > 5$ or $B > 150$, we thus need a figure of merit exceeding 375. Demanding that Euclid achieve a FoM $> \,500$ places us, therefore, on the safe side and allows to reach the same conclusions (the ability to favor the $\varLambda$CDM expansion history decisively *if* the data is in full agreement with $w=-\,1$) under small variations of the prior as well.

To summarize, the most direct effect of dynamical dark energy is the modification of the expansion history. We used inflation as a dark-energy prototype to show that the current experimental bounds of $w \approx -\,1.0 \pm 0.1$ are not yet sufficient to significantly favor a parameter-free $\varLambda$CDM expansion history: we showed that we need to reach a percent-level accuracy both to have any chance of observing a deviation of *w* from $-\,1$ if the dark energy is similar to inflation, and because it is at this point that a $w=-\,1$ expansion history beings to be favored decisively for prior widths of order 1.

We do not expect to be able to improve much our knowledge with a lower-precision measurement of *w*, unless dark energy is significantly different from $w=-\,1$ either at late times or, for example, owing to a significant early-dark-energy component (Pettorino et al. 2013). A large deviation would be the preferred situation for Euclid, as then we would be able to observe the evolution of dark energy rather than just a fixed state, which would be much more revealing. However, even if the expansion history matches that of $\varLambda$CDM to some arbitrary precision, this does not imply that the cosmological constant is accelerating the universe. Even on such configurations a large amount of freedom exists which can then only be tested by investigating the evolution of large-scale structure, to which we now turn.

#### Dark-energy: linear perturbations and growth rate

Without a given model for dark energy, the evolution of its perturbations is not determined by the background expansion history. As we have explained in Sect. I.4.1, the cosmological constant is the only form of dark energy which does not carry fluctuations at all, with all dynamical DE models clustering to a larger or smaller extent. Since both dark matter and dark energy must interact (at least) through gravity, the existence of these fluctuations would alter the geodesics on which pressureless dark matter moves and therefore also change the clustering history of the dark matter. This implies that the appropriate evolution history for perturbations is another consistency check that $\varLambda$CDM must satisfy over and above the matching background expansion history.

In order to meaningfully discuss dark-energy fluctuations, we must specify the following:

- the field content of the dark-energy sector
- the initial conditions for the fluctuations
- either the initial conditions for the DE background configuration and subsequent evolution or a measurement of the background expansion history as discussed in Sect. I.4.3
- the rules for evolving the fluctuations (i.e., the model of dark energy)

A scalar–vector–tensor decomposition of the perturbations on the FLRW background can be performed, where each of the spins evolves independently at linear order. Since general relativity only contains tensors as dynamical degrees of freedom, any dynamics in the scalar and vector modes is determined by the matter content. Therefore fluctuations of dark energy provide a source for metric degrees of freedom.

Typically, a vector or tensor degree of freedom will contain all the lower helicities and therefore source all the perturbations of lower spin. For example, a vector dark energy (e.g., Einstein-Aether or TeVeS), will in general source both vector and scalar perturbations. Higher-spin perturbations would affect polarization predictions for the CMB if the dark energy contributed a significant part of the energy density during recombination (Lim 2005), but otherwise are unconstrained and appear largely uninvestigated in the literature, where most attention is paid to scalar modes even in models containing higher-spin matter. If the dark energy itself contains multiple degrees of freedom, the perturbations will also feature internal modes which do not change any of the potential observables, such as the gravitational potentials, and only affect how they evolve in time.

Each of the new dynamical modes must be given appropriate initial conditions. Typically, they should be set during inflation, where the dark energy plays the role of a spectator field(s). In particular, the dark energy will contribute to the scalar adiabatic mode, which is constant on scales larger than the cosmological horizon. In addition, it will introduce new isocurvature modes with respect to matter and radiation. In general, these only decay if the dark energy interacts with the other matter components and equilibrates, in particular if the dark energy features a tracker in its evolution history (Malquarti and Liddle 2002). These isocurvature modes affect the CMB and are strongly constrained, but again only if the dark energy is a significant fraction of total energy density during recombination, such as in early dark energy models. Otherwise, the isocurvature modes do not become relevant until the late universe where they can affect structure formation or at least the magnitude of the ISW effect on the CMB (Gordon and Hu 2004). If the dark-energy is not coupled to the inflaton and is not involved in reheating, the isocurvature modes are likely to be statistically uncorrelated.

In practice, for the purpose of the late universe, the assumption is made that the isocurvature modes are not present and only the scalar adiabatic mode is considered. Let us take this point of view for the remainder of this section. Therefore what we discuss now are the possible rules for evolving the linear scalar perturbations.

As discussed in Sect. I.3.2, a “closure” relation between the dark matter density perturbation $\varDelta _M$ and the scalar gravitational potentials $\varPhi$ and $\varPsi$ is enough to describe the evolution of all these variables Sawicki et al. (2013). We can define two variables *Q* and $\eta$,

I.4.7 $$\begin{aligned} -k^2 \varPhi = 4\pi G Q(a,k) a^2 \rho _M \varDelta _M, \qquad \varPhi =\eta (a,k)\varPsi , \end{aligned}$$

which provide such closure relations. However, these two variables are actually just a recasting of the equations: they effectively parameterize the particular solutions that are realized by the universe, rather than necessarily saying anything in particular about the model: *Q* describes the energy density perturbations of dark energy in the realized configuration, while $\eta$ is a statement about the anisotropic stress carried by that configuration. It should be clear that at any *one* particular redshift it is always be possible to arrange the dark-energy configuration such that $Q=\eta =1$.

In $\varLambda$CDM, the dark-matter density perturbation $\varDelta _M$ and the gravitational potential $\varPhi$ are related through a constraint, if we ignore the other components in the universe, such as baryons and radiation. In dynamical dark-energy models with a single additional degree of freedom, the $\varDelta _M$ equation is one of two coupled second-order differential equations, with time- and scale-dependent coefficients determined by the model.

At this point, one often employs the quasi-static approximation, i.e. neglecting all time derivatives in the dynamical equation for $\varPhi$, which is equivalent to treating it as a constraint and therefore the dark energy purely as an effective modification of the constraint structure of general relativity, rather than as a full degree of freedom. This sort of approximation seems to be valid inside the sound horizon for the dark energy at least for some models, although it is not proven that it works in general. Under this quasi-static approximation, in any (single) scalar–tensor theory (Sawicki et al. 2013)

I.4.8 $$\begin{aligned} Q = h_1 \left( \frac{ a^2 H^2 + k^2 h_5}{a^2 H^2 + k^2 h_3} \right) , \quad \eta = h_2 \left( \frac{a^2 H^2 + k^2 h_4}{a^2 H^2 + k^2 h_5} \right) , \end{aligned}$$

where the $h_i$ are essentially arbitrary functions of time only, determined by the action of the model. In fact, a more general argument given in Silvestri et al. (2013), proves that simply requiring quasi-staticity and locality for the theory of dark energy implies that both the functions *Q* and $\eta$ are ratios of polynomials of $\left( k/aH\right) ^2$ with coefficients purely functions of time. Theories which break these assumptions, can have a different structure, e.g., DGP where contributions appear at order *k* / *aH* as a result of the existence of a singular brane source (Amin et al. 2008).

Let us now discuss under what circumstances the two functions *Q* and $\eta$ deviate from their $\varLambda$CDM values considerably and therefore would presumably significantly change observables.

*I.4.4.1 Anisotropic stress:* $$\eta \ne 1$$

A deviation of $\eta$ from 1 results from anisotropic stress at first order in perturbations of the fluid. This occurs in the early universe owing to relativistic neutrinos, but is negligible at late times. Note that at second order in perturbations anisotropic stress is always present (e.g., Ballesteros et al. 2012).

The existence of anisotropic stress is a frame-dependent question. Models such as *f*(*R*) gravity which exhibit anisotropic stress, can be redefined through a frame transformation to have none. In addition to specifying a model, we must therefore fix a frame in order to discuss this properly. The natural frame to pick is the Jordan frame of the baryons. This is defined as the frame related to the metric on the geodesics of which visible matter propagates when in free fall. In many modified-gravity models, this is also the Jordan frame for dark matter, i.e., gravity-like forces couple to just the EMT, irrespective of species. All observations of perturbations to be performed by Euclid are those of the motion of galaxies and light which directly probes the Jordan-frame metric for these species.

Given the fixed baryon Jordan frame, the anisotropic stress appears whenever the effective Planck mass is not constant, i.e., whenever the normalization of the kinetic term of gravitons is time dependent, or when the speed of tensor modes is different from the speed of light. Anisotropic stress therefore is a probe of the nature of the action for gravitational waves. This occurs whenever there are dynamical degrees of freedom which are coupled non-minimally to gravity. For example, the *f*(*R*) action, seemingly without any additional dynamical degrees of freedom, can be Legendre transformed into the equivalent (Chiba 2003)

I.4.9 $$\begin{aligned} S=\frac{1}{2\kappa ^2} \int \mathrm {d}^4x\sqrt{-g} \left[ \phi R + V(\phi ) + \mathcal {L}_m(\varPsi _m) \right] , \end{aligned}$$

with $V(\phi )\equiv f - R(\phi )f_R$, where the coupling between gravity and the scalar $\phi =f_R$ is explicit.

On the other hand, many coupled dark energy models are constructed to be very similar to *f*(*R*), but introduce a split between the dark matter and visible matter frames. When the visible matter is subdominant gravitationally, the growth of dark matter perturbations in these two classes of models should be very similar. However, all the measurements are always performed through the galaxies and weak lensing and therefore observations are different; in particular, there is no anisotropic stress in CDE models (Motta et al. 2013).

When dealing with multiple degrees of freedom, it is in principle possible to tune them in such a way that the time-variation of the Planck mass cancels out and therefore there would be no anisotropic stress despite non-minimal coupling to gravity in the baryon Jordan frame. However, if the action for the two degrees of freedom is of a different model class, it is not clear whether it is possible to perform this cancellation during more than one era of the evolution of the universe, e.g., matter domination (see Saltas and Kunz 2011 for the case of *f*(*R*, *G*) gravity^4^).

*I.4.4.2 Clustering:* $$Q\ne 1$$

The implication of DE clustering, $Q\ne 1$ is that the dark matter perturbations are dressed with dark energy perturbations. This means that the effect of some particular density of matter is to curve space differently than in $\varLambda$CDM. On scales where *Q* is a constant, the dark-energy distribution follows that of the dark matter precisely. $QG_\text {N}$ is an effective Newton’s constant for non-relativistic matter.

If the curvature of space sourced by the DM changes, then so does the gravitational force acting on the dark matter. This implies that given a fixed background expansion history, the growth rate of the perturbations is different, see Sect. I.3.2.

As discussed in Sect. I.4.1, only a cosmological constant is not perturbed at all. Therefore only in this case do we have $Q=1$ exactly up to relativistic corrections near the cosmological horizon, $k/aH\sim 1$.

However, when the dark energy comprises a single dynamical degree of freedom and has an EMT of perfect-fluid form (e.g., k-essence is the most general model of this type, with quintessence I.5.1 as a subclass) and the background expansion history is very close to $\varLambda$CDM, the exact equations for the DE/DM system coupled through gravity can be written as (Motta et al. 2013)

I.4.10 $$\begin{aligned} \varPhi ''&+ \left( 4 + \frac{H'}{H} + 3c_\text {a}^2 \right) \varPhi ' + \left( 3+ 2\frac{H'}{H}+3c_\text {a}^2 \right) \varPhi + \left( \frac{c_\text {s}k}{aH} \right) ^2 \varPhi = \end{aligned}$$

I.4.11 $$\begin{aligned}&- \frac{3}{2}\varOmega _\text {m} \left( c_\text {s}^2 \delta _\text {m} + 3(c_\text {a}^2-c_\text {s}^2) \frac{a^2 H}{k^2} \theta _\text {m}\right) \nonumber \\ \delta _\text {m}'&+ H^{-1}\theta _\text {m} = 3\varPhi ' \qquad \theta _\text {m}' + 2\theta _\text {m} = \frac{k^2}{a^2H}\varPhi \end{aligned}$$

with $c_\text {a}^2\equiv p'_\text {DE}/\rho _\text {DE}'$ the adiabatic sound speed and the prime denoting differentiation w.r.t. $\ln a$. Deep inside the sound horizon, $c_\text {s}k/aH\gg 1$, the standard Poisson constraint can be recovered from Eq. (I.4.10), i.e., $Q=1$. The growth rate of the dark matter perturbation is then fully determined by the background expansion and is only different from the $\varLambda$CDM one when the expansion history deviates significantly. This is the standard case of quintessence, for which the sound speed $c_\text {s}=1$.

In the opposite limit of clustering dark energy, $c_\text {s}=0$, which is typical of k-essence models such as the ghost-condensate (Arkani-Hamed et al. 2004b) or dusty dark energy (Lim et al. 2010), there is no sound horizon, just as for dark-matter dust, and in principle the dark energy clusters. If the background expansion is now very close to $\varLambda$CDM (i.e., $w\approx -1$ and $c_\text {a}^2\approx 0$), Eq. (I.4.10) reduces to the standard equation in $\varLambda$CDM. If the initial conditions are adiabatic, the evolution of both the potential and of the dark matter density is the same as in $\varLambda$CDM, i.e., $Q=1$ again. Any deviations are purely a result of a different background expansion history. The above implies that given a very similar expansion history to $\varLambda$CDM, dark-energy models comprising a single degree of freedom that is minimally coupled do not significantly cluster (Sapone and Kunz 2009) if the initial conditions are adiabatic and there is no anisotropic stress.

For significant clustering, a coupling of dark energy to gravity or dark matter is required, with a strength similar to that of gravity (or possibly to other species, with appropriately stronger couplings to compensate for the relatively smaller energy density). All models which exhibit significant anisotropic stress also cluster significantly, since the anisotropic stress is a sign of non-minimal coupling to gravity, see Sect. I.4.4.1. This implies that models such as coupled dark energy also cluster, since they are effectively scalar–tensor models with non-universal couplings to matter.

If the couplings are universal, the most general class of models where $Q\ne 1$ while there is no anisotropic stress are kinetic gravity braiding models of dark energy (Deffayet et al. 2010), which are a class of imperfect fluids (Pujolas et al. 2011). The effective Planck mass is constant in these models, however there is still non-minimal coupling to gravity on the level of the equations of motion. This implies that they cluster significantly (Kimura and Yamamoto 2011).

In summary, given a fixed background expansion history close to $\varLambda$CDM, the appearance of anisotropic stress is a sign of a modification of the action for gravitational waves in the Jordan frame of baryons: either a time-varying effective Planck mass, i.e., a normalization scale for graviton kinetic terms or a deviation of the speed of gravitational waves from that of light. On the other hand, a detection of significant clustering, resulting in a growth rate significantly deviating from the $\varLambda$CDM one, is a sign of coupling of the dark-energy to gravity or some of the species with a strength similar to that of gravity.

#### Parameterized frameworks for theories of modified gravity

As explained in earlier sections of this report, modified-gravity models cannot be distinguished from dark-energy models by using solely the FLRW background equations. But by comparing the background expansion rate of the universe with observables that depend on linear perturbations of an FRW spacetime we can hope to distinguish between these two categories of explanations. An efficient way to do this is via a parameterized, model-independent framework that describes cosmological perturbation theory in modified gravity. We present here one such framework, the parameterized post-Friedmann formalism (Baker et al. 2013)^5^ that implements possible extensions to the linearized gravitational field equations.

The parameterized post-Friedmann approach (PPF) is inspired by the parameterized post-Newtonian (PPN) formalism (Will and Nordtvedt 1972; Will 1971), which uses a set of parameters to summarize leading-order deviations from the metric of GR. PPN was developed in the 1970s for the purpose of testing of alternative gravity theories in the solar system or binary systems, and is valid in weak-field, low-velocity scenarios. PPN itself cannot be applied to cosmology, because we do not know the exact form of the linearized metric for our Hubble volume. Furthermore, PPN can only test for constant deviations from GR, whereas the cosmological data we collect contain inherent redshift dependence.

For these reasons the PPF framework is a parameterization of the gravitational field equations (instead of the metric) in terms of a set of functions of redshift. A theory of modified gravity can be analytically mapped onto these PPF functions, which in turn can be constrained by data.

We begin by writing the perturbed Einstein field equations for spin-0 (scalar) perturbations in the form:

I.4.12 $$\begin{aligned} \delta G_{\mu \nu } \;=\; 8\pi G\,\delta T_{\mu \nu }+\delta U_{\mu \nu }^{\mathrm {metric}}+\delta U_{\mu \nu }^{\mathrm {d.o.f}}+\mathrm {\ gauge\ invariance\ fixing\ terms}, \end{aligned}$$

where $\delta T_{\mu \nu }$ is the usual perturbed stress–energy tensor of all cosmologically-relevant fluids. The tensor $\delta U_{\mu \nu }^{\mathrm {metric}}$ holds new terms that may appear in a modified theory, containing perturbations of the metric (in GR such perturbations are entirely accounted for by $\delta G_{\mu \nu }$). $\delta U_{\mu \nu }^{\mathrm {d.o.f.}}$ holds perturbations of any new degrees of freedom that are introduced by modifications to gravity. A simple example of the latter is a new scalar field, such as introduced by scalar–tensor or Galileon theories. However, new degrees of freedom could also come from spin-0 perturbations of new tensor or vector fields, St$\ddot{\mathrm {u}}$ckelberg fields, effective fluids and actions based on curvature invariants (such as $f\left( R\right)$ gravity).

In principle there could also be new terms containing matter perturbations on the RHS of Eq. (I.4.12). However, for theories that maintain the weak equivalence principle—i.e., those with a Jordan frame where matter is uncoupled to any new fields—these matter terms can be eliminated in favor of additional contributions to $\delta U_{\mu \nu }^{\mathrm {metric}}$ and $\delta U_{\mu \nu }^{\mathrm {d.o.f.}}$.

The tensor $\delta U_{\mu \nu }^{\mathrm {metric}}$ is then expanded in terms of two gauge-invariant perturbation variables ${\hat{\varPhi }}$ and ${\hat{\varGamma }}$. ${\hat{\varPhi }}$ is one of the standard gauge-invariant Bardeen potentials, while ${\hat{\varGamma }}$ is the following combination of the Bardeen potentials: ${\hat{\varGamma }}=1/k (\dot{{\hat{\varPhi }}}+\mathcal{H}{\hat{\varPsi }})$. We use ${\hat{\varGamma }}$ instead of the usual Bardeen potential ${\hat{\varPsi }}$ because ${\hat{\varGamma }}$ has the same derivative order as ${\hat{\varPhi }}$ (whereas ${\hat{\varPsi }}$ does not). We then deduce that the only possible structure of $\delta U_{\mu \nu }^{\mathrm {metric}}$ that maintains the gauge-invariance of the field equations is a linear combination of ${\hat{\varPhi }}$, ${\hat{\varGamma }}$ and their derivatives, multiplied by functions of the cosmological background (see Eqs. (I.4.13)–(I.4.17) below).

$\delta U_{\mu \nu }^{\mathrm {d.o.f.}}$ is similarly expanded in a set of gauge-invariant potentials $\{{\hat{\chi _i\}}}$ that contain the new degrees of freedom. Baker et al. (2013) presented an algorithm for constructing the relevant gauge-invariant quantities in any theory.

For concreteness we will consider here a theory that contains only one new degree of freedom and is second-order in its equations of motion (a generic but not watertight requirement for stability, see Woodard 2007). Then the four components of Eq. (I.4.12) are:

I.4.13 $$\begin{aligned} -a^2\delta G^0_0= & {} 8\pi a^2 G\,\rho _M\delta _M+A_0 k^2{\hat{\varPhi }}+F_0k^2{\hat{\varGamma }}+\alpha _0k^2{\hat{\chi }}+\alpha _1k\dot{{\hat{\chi }}}+k^3 M_{\varDelta }({\dot{\nu }}+2\epsilon ) \end{aligned}$$

I.4.14 $$\begin{aligned} -a^2\delta G^0_i= & {} \nabla _i\left[ 8\pi a^2 G\,\rho _M (1+\omega _M)\theta _M+B_0 k{\hat{\varPhi }}+I_0k{\hat{\varGamma }}+\beta _0 k{\hat{\chi }}\right. \nonumber \\&\quad \left. +\,\beta _1\dot{{\hat{\chi }}}+k^2M_{\varTheta }({\dot{\nu }}+2\epsilon )\right] \end{aligned}$$

I.4.15 $$\begin{aligned} a^2\delta G^i_i= & {} 3\,8\pi a^2 G\,\rho _M\varPi _M+C_0 k^2{\hat{\varPhi }}+C_1 k\dot{{\hat{\varPhi }}}+J_0k^2{\hat{\varGamma }}+J_1 k\dot{{\hat{\varGamma }}}+\gamma _0 k^2{\hat{\chi }}\nonumber \\&\quad +\, \gamma _1 k \dot{{\hat{\chi }}}+\gamma _2 \ddot{{\hat{\chi }}} \end{aligned}$$

I.4.16 $$\begin{aligned}&\quad +\, k^3M_P ({\dot{\nu }}+2\epsilon ) \end{aligned}$$

I.4.17 $$\begin{aligned} a^2\delta \hat{G}^i_j= & {} 8\pi a^2 G\,\rho _M (1+\omega _M)\varSigma _M+ D_0{\hat{\varPhi }}+\frac{D_1}{k} \dot{{\hat{\varPhi }}}+K_0{\hat{\varGamma }}+\frac{K_1}{k}\dot{{\hat{\varGamma }}}\nonumber \\&\quad +\,\,\epsilon _0{\hat{\chi }}+\frac{\epsilon _1}{k}\dot{{\hat{\chi }}}+\frac{\epsilon _2}{k^2} \ddot{{\hat{\chi }}} \end{aligned}$$

where $\delta \hat{G}^i_j=\delta G^i_j-\frac{\delta ^i_j}{3}\delta G^k_k$. Each of the lettered coefficients in Eqs. (I.4.13)–(I.4.17) is a function of cosmological background quantities, i.e., functions of time or redshift; this dependence has been suppressed above for clarity. Potentially, the coefficients could also depend on scale, but this dependence is not arbitrary Silvestri et al. 2013). These PPF coefficients are the analogy of the PPN parameters; they are the objects that a particular theory of gravity ‘maps onto’, and the quantities to be constrained by data. Numerous examples of the PPF coefficients corresponding to well-known theories are given in Baker et al. (2013).

The final terms in Eqs. (I.4.13)–(I.4.16) are present to ensure the gauge invariance of the modified field equations, as is required for any theory governed by a covariant action. The quantities $M_\varDelta$, $M_\varTheta$ and $M_P$ are all pre-determined functions of the background. $\epsilon$ and $\nu$ are off-diagonal metric perturbations, so these terms vanish in the conformal Newtonian gauge. The gauge-fixing terms should be regarded as a piece of mathematical book-keeping; there is no constrainable freedom associated with them.

One can then calculate observable quantities—such as the weak lensing kernel or the growth rate of structure *f*(*z*)—using the parameterized field Eqs. (I.4.13)–(I.4.17). Similarly, they can be implemented in an Einstein–Boltzmann solver code such as camb (Lewis et al. 2000b) to utilize constraints from the CMB. If we take the divergence of the gravitational field equations [i.e., the unperturbed equivalent of Eq. (I.4.12)], the left-hand side vanishes due to the Bianchi identity, while the stress–energy tensor of matter obeys its standard conservation equations (since we are working in the Jordan frame). Hence the *U*-tensor must be separately conserved, and this provides the necessary evolution equation for the variable ${\hat{\chi }}$:

I.4.18 $$\begin{aligned} \delta \left( \nabla ^\mu \left[ U_{\mu \nu }^{\mathrm {metric}}+U_{\mu \nu }^{\mathrm {d.o.f.}}\right] \right)&=0. \end{aligned}$$

Equation (I.4.18) has two components. If one wishes to treat theories with more than two new degrees of freedom, further information is needed to supplement the PPF framework.

The full form of the parameterized Eqs. (I.4.13)–(I.4.17) can be simplified in the ‘quasistatic regime’, that is, significantly sub-horizon scales on which the time derivatives of perturbations can be neglected in comparison to their spatial derivatives (Hu and Sawicki 2007b). Quasistatic lengthscales are the relevant stage for weak lensing surveys and galaxy redshift surveys such as those of Euclid. A common parameterization used on these scales has the form:

I.4.19 $$\begin{aligned} 2\nabla ^2\varPhi&=8\pi a^2G\,\mu (a,k)\,{{\bar{\rho }}}_M\varDelta _M, \end{aligned}$$

I.4.20 $$\begin{aligned} \frac{\varPhi }{\varPsi }&=\gamma (a,k), \end{aligned}$$

where $\{\mu ,\gamma \}$ are two functions of time and scale to be constrained. This parameterization has been widely employed (Bertschinger and Zukin 2008; Daniel and Linder 2010; Linder and Cahn 2007; Bean and Tangmatitham 2010; Pogosian et al. 2010; Zhao et al. 2010; Dossett et al. 2011; Hojjati et al. 2011, 2012). It has the advantages of simplicity and somewhat greater physical transparency: $\mu (a,k)$ can be regarded as describing evolution of the effective gravitational constant, while $\gamma (a,k)$ can, to a certain extent, be thought of as acting like a source of anisotropic stress (see Sect. I.4.4).

Let us make a comment about the number of coefficient functions employed in the PPF formalism. One may justifiably question whether the number of unknown functions in Eqs. (I.4.13)–(I.4.17) could ever be constrained. In reality, the PPF coefficients are not all independent. The form shown above represents a fully agnostic description of the extended field equations. However, as one begins to impose restrictions in theory space (even the simple requirement that the modified field equations must originate from a covariant action), constraint relations between the PPF coefficients begin to emerge. These constraints remove freedom from the parameterization.

Even so, degeneracies will exist between the PPF coefficients. It is likely that a subset of them can be well-constrained, while another subset have relatively little impact on current observables and so cannot be tested. In this case it is justifiable to drop the untestable terms. Note that this realization, in itself, would be an interesting statement—that there are parts of the gravitational field equations that are essentially unknowable.

Finally, we note that there is also a completely different, complementary approach to parameterizing modifications to gravity. Instead of parameterizing the linearized field equations, one could choose to parameterize the perturbed gravitational action. This approach has been used recently to apply the standard techniques of effective field theory to modified gravity; see Battye and Pearson (2012), Bloomfield et al. (2013), Gubitosi et al. (2013) and references therein.

### Models of dark energy and modified gravity

In this section we review a number of popular models of dynamical DE and MG. This section is more technical than the rest and it is meant to provide a quick but self-contained review of the current research in the theoretical foundations of DE models. The selection of models is of course somewhat arbitrary but we have tried to cover the most well-studied cases and those that introduce new and interesting observable phenomena.

#### Quintessence

In this review, we refer to scalar field models with canonical kinetic energy in Einstein’s gravity as “quintessence models”. Scalar fields are obvious candidates for dark energy, as they are for the inflaton, for many reasons: they are the simplest fields since they lack internal degrees of freedom, do not introduce preferred directions, are typically weakly clustered (as discussed later on), and can easily drive an accelerated expansion. If the kinetic energy has a canonical form, the only degree of freedom is then provided by the field potential (and of course by the initial conditions). The typical requirement is that the potentials are flat enough to lead to the slow-roll inflation today with an energy scale $\rho _{\mathrm {DE}}\simeq 10^{-123}m_{\mathrm {pl}}^{4}$ and a mass scale $m_{\phi }\lesssim 10^{-33}\mathrm {\ eV}$.

Quintessence models are the prototypical DE models (Caldwell et al. 1998) and as such are the most studied ones. Since they have been explored in many reviews of DE, we limit ourselves here to a few remarks.^6^

The quintessence model is described by the action

I.5.1 $$\begin{aligned} S=\int {\mathrm {d}}^{4}x\sqrt{-g}\,\left[ \frac{1}{2\kappa ^{2}}R+\mathcal{L}_{\phi }\right] +S_{M},\quad \mathcal{L}_{\phi }=-\frac{1}{2}g^{\mu \nu }\partial _{\mu }\phi \partial _{\nu }\phi -V(\phi ),\qquad \quad \end{aligned}$$

where $\kappa ^{2}=8\pi G$ and *R* is the Ricci scalar and $S_{M}$ is the matter action. The fluid satisfies the continuity equation

I.5.2 $$\begin{aligned} \dot{\rho }_{M}+3H(\rho _{M}+p_{M})=0. \end{aligned}$$

The energy–momentum tensor of quintessence is

I.5.3 $$\begin{aligned} T_{\mu \nu }^{(\phi )}= & {} -\frac{2}{\sqrt{-g}}\frac{\delta \left( \sqrt{-g}{{\mathcal {L}}}_{\phi }\right) }{\delta g^{\mu \nu }} \end{aligned}$$

I.5.4 $$\begin{aligned}= & {} \partial _{\mu }\phi \partial _{\nu }\phi -g_{\mu \nu }\left[ \frac{1}{2}g^{\alpha \beta }\partial _{\alpha }\phi \partial _{\beta }\phi +V(\phi )\right] .\end{aligned}$$

As we have already seen, in a FLRW background, the energy density $\rho _{\phi }$ and the pressure $p_{\phi }$ of the field are

I.5.5 $$\begin{aligned} \rho _{\phi }=-{T_{0}^{0}}^{(\phi )}=\frac{1}{2}\dot{\phi }^{2}+V(\phi ),\quad p_{\phi }=\frac{1}{3}{T_{i}^{i}}^{(\phi )}=\frac{1}{2}\dot{\phi }^{2}-V(\phi ), \end{aligned}$$

which give the equation of state

I.5.6 $$\begin{aligned} w_{\phi }\equiv \frac{p_{\phi }}{\rho _{\phi }}=\frac{\dot{\phi }^{2}-2V(\phi )}{\dot{\phi }^{2}+2V(\phi )}. \end{aligned}$$

In the flat universe, Einstein’s equations give the following equations of motion:

I.5.7 $$\begin{aligned}&H^{2}=\frac{\kappa ^{2}}{3}\left[ \frac{1}{2}\dot{\phi }^{2}+V(\phi )+\rho _{M}\right] , \end{aligned}$$

I.5.8 $$\begin{aligned}&\dot{H}=-\frac{\kappa ^{2}}{2}\left( \dot{\phi }^{2}+\rho _{M}+p_{M}\right) , \end{aligned}$$

where $\kappa ^{2}=8\pi G$. The variation of the action (I.5.1) with respect to $\phi$ gives

I.5.9 $$\begin{aligned} \ddot{\phi }+3H\dot{\phi }+V_{,\phi }=0, \end{aligned}$$

where $$V_{,\phi }\equiv {\mathrm {d}}V/{\mathrm {d}}\phi $$.

During radiation or matter dominated epochs, the energy density $\rho _{M}$ of the fluid dominates over that of quintessence, i.e., $\rho _{M}\gg \rho _{\phi }$. If the potential is steep so that the condition $\dot{\phi }^{2}/2\gg V(\phi )$ is always satisfied, the field equation of state is given by $w_{\phi }\simeq 1$ from Eq. (I.5.6). In this case the energy density of the field evolves as $\rho _{\phi }\propto a^{-6}$, which decreases much faster than the background fluid density.

The condition $w_{\phi }<-\,1/3$ is required to realize the late-time cosmic acceleration, which translates into the condition $\dot{\phi }^{2}<V(\phi )$. Hence the scalar potential needs to be shallow enough for the field to evolve slowly along the potential. This situation is similar to that in inflationary cosmology and it is convenient to introduce the following slow-roll parameters (Bassett et al. 2006)

I.5.10 $$\begin{aligned} \epsilon _{s}\equiv \frac{1}{2\kappa ^{2}}\left( \frac{V_{,\phi }}{V}\right) ^{2} , \qquad \eta _{s}\equiv \frac{V_{,\phi \phi }}{\kappa ^{2}V}. \end{aligned}$$

If the conditions $\epsilon _{s}\ll 1$ and $|\eta _{s}|\ll 1$ are satisfied, the evolution of the field is sufficiently slow so that $\dot{\phi }^{2}\ll V(\phi )$ and $|\ddot{\phi }|\ll |3H\dot{\phi }|$ in Eqs. (I.5.7) and (I.5.9).

From Eq. (I.5.9) the deviation of $w_{\phi }$ from $-\,1$ is given by

I.5.11 $$\begin{aligned} 1+w_{\phi }=\frac{V_{,\phi }^{2}}{9H^{2}(\xi _{s}+1)^{2}\rho _{\phi }} , \end{aligned}$$

where $\xi _{s}\equiv \ddot{\phi }/(3H\dot{\phi })$. This shows that $w_{\phi }$ is always larger than $-\,1$ for a positive potential and energy density. In the slow-roll limit, $|\xi _{s}|\ll 1$ and $\dot{\phi }^{2}/2\ll V(\phi )$, we obtain $1+w_{\phi }\simeq 2\epsilon _{s}/3$ by neglecting the matter fluid in Eq. (I.5.7), i.e., $3H^{2}\simeq \kappa ^{2}V(\phi )$. The deviation of $w_{\phi }$ from $-\,1$ is characterized by the slow-roll parameter $\epsilon _{s}$. It is also possible to consider Eq. (I.5.11) as a prescription for the evolution of the potential given $w_\phi (z)$ and to reconstruct a potential that gives a desired evolution of the equation of state (subject to $w\in [-\,1,1]$). This was used, for example, in Bassett et al. (2002).

However, in order to study the evolution of the perturbations of a quintessence field it is not even necessary to compute the field evolution explicitly. Rewriting the perturbation equations of the field in terms of the perturbations of the density contrast $\delta _\phi$ and the velocity $\theta _\phi$ in the conformal Newtonian gauge, one finds (see, e.g., Kunz and Sapone 2006, “Appendix A” section) that they correspond precisely to those of a fluid, (I.3.17) and (I.3.18), with $\pi =0$ and $\delta p = c_s^2 \delta \rho + 3 a H (c_s^2-c_a^2) (1+w) \rho \theta /k^2$ with $c_s^2=1$. The adiabatic sound speed, $c_a$, is defined in Eq. (I.3.36). The large value of the sound speed $c_s^2$, equal to the speed of light, means that quintessence models do not cluster significantly inside the horizon (see Sapone and Kunz 2009; Sapone et al. 2010, and Sect. I.8.6 for a detailed analytical discussion of quintessence clustering and its detectability with future probes, for arbitrary $c_s^2$).

Many quintessence potentials have been proposed in the literature. A simple crude classification divides them into two classes, (i) “freezing” models and (ii) “thawing” models (Caldwell and Linder 2005). In class (i) the field was rolling along the potential in the past, but the movement gradually slows down after the system enters the phase of cosmic acceleration. The representative potentials that belong to this class are

**(i) Freezing models**

- $$V(\phi )=M^{4+n}\phi ^{-n}\quad (n>0)$$,
- $$V(\phi )=M^{4+n}\phi ^{-n}\exp \left( \alpha \phi ^{2}/m_{\mathrm {pl}}^{2}\right) $$.

The former potential does not possess a minimum and hence the field rolls down the potential toward infinity. This appears, for example, in the fermion condensate model as a dynamical supersymmetry breaking (Binétruy 1999). The latter potential has a minimum at which the field is eventually trapped (corresponding to $w_{\phi }=-\,1$). This potential can be constructed in the framework of supergravity (Brax and Martin 1999).

In thawing models (ii) the field (with mass $m_{\phi }$) has been frozen by Hubble friction [i.e., the term $H\dot{\phi }$ in Eq. (I.5.9)] until recently and then it begins to evolve once *H* drops below $m_{\phi }$. The equation of state of DE is $w_{\phi }\simeq -1$ at early times, which is followed by the growth of $w_{\phi }$. The representative potentials that belong to this class are

**(ii) Thawing models**

- $$V(\phi )=V_{0}+M^{4-n}\phi ^{n}\quad (n>0)$$,
- $$V(\phi )=M^{4}\cos ^{2}(\phi /f)$$.

The former potential is similar to that of chaotic inflation ($$n=2,4$$) used in the early universe (with $$V_{0}=0)$$ (Linde 1983), while the mass scale *M* is very different. The model with $$n=1$$ was proposed by Kallosh et al. (2003) in connection with the possibility to allow for negative values of $$V(\phi )$$. The universe will collapse in the future if the system enters the region with $$V(\phi )<0$$. The latter potential appears as a potential for the Pseudo-Nambu–Goldstone Boson (PNGB). This was introduced by Frieman et al. (1995) in response to the first tentative suggestions that the universe may be dominated by the cosmological constant. In this model the field is nearly frozen at the potential maximum during the period in which the field mass $$m_{\phi }$$ is smaller than *H*, but it begins to roll down around the present ($$m_{\phi }\simeq H_{0}$$).

Potentials can also be classified in several other ways, e.g., on the basis of the existence of special solutions. For instance, tracker solutions have approximately constant $w_{\phi }$ and $\varOmega _{\phi }$ along special attractors. A wide range of initial conditions converge to a common, cosmic evolutionary tracker. Early DE models contain instead solutions in which DE was not negligible even during the last scattering. While in the specific Euclid forecasts Sect. I.8 we will not explicitly consider these models, it is worthwhile to note that the combination of observations of the CMB and of large scale structure (such as Euclid) can dramatically constrain these models drastically improving the inverse area figure of merit compared to current constraints, as discussed in Huterer and Peiris (2007).

#### K-essence

In a quintessence model it is the potential energy of a scalar field that leads to the late-time acceleration of the expansion of the universe; the alternative, in which the kinetic energy of the scalar field which dominates, is known as k-essence. Models of k-essence are characterized by an action for the scalar field of the following form

I.5.12 $$\begin{aligned} S=\int \mathrm {d}^4 x\;\sqrt{-g}p(\phi ,X), \end{aligned}$$

where $X=(1/2)g^{\mu \nu }\nabla _{\mu }\phi \nabla _{\nu }\phi$. The energy density of the scalar field is given by

I.5.13 $$\begin{aligned} \rho _{\phi }=2X\frac{\mathrm {d}p}{\mathrm {d}X}-p, \end{aligned}$$

and the pressure is simply $p_{\phi }=p(\phi ,X)$. Treating the k-essence scalar as a perfect fluid, this means that k-essence has the equation of state

I.5.14 $$\begin{aligned} w_{\phi }=\frac{p_{\phi }}{\rho _{\phi }}=-\frac{p}{p-2X p,_{X}}, \end{aligned}$$

where the subscript, $_{X}$ indicates a derivative with respect to *X*. Clearly, with a suitably chosen *p* the scalar can have an appropriate equation of state to allow it to act as dark energy.

The dynamics of the k-essence field are given by a continuity equation

I.5.15 $$\begin{aligned} \dot{\rho }_{\phi }=-\,3H(\rho _{\phi }+p_{\phi }), \end{aligned}$$

or equivalently by the scalar equation of motion

I.5.16 $$\begin{aligned} G^{\mu \nu }\nabla _{\mu }\nabla _{\nu }\phi +2X\frac{\partial ^2p}{\partial X \partial \phi }-\frac{\partial p}{\partial \phi }=0, \end{aligned}$$

where

I.5.17 $$\begin{aligned} G^{\mu \nu }=\frac{\partial p}{\partial X}g^{\mu \nu }+\frac{\partial ^2 p}{\partial X^2}\nabla ^{\mu }\phi \nabla ^{\nu }\phi . \end{aligned}$$

For this second order equation of motion to be hyperbolic, and hence physically meaningful, we must impose

I.5.18 $$\begin{aligned} 1+2X\frac{p,_{XX}}{p,_X}>0. \end{aligned}$$

K-essence was first proposed by Armendariz-Picon et al. (2000, 2001), where it was also shown that tracking solutions to this equation of motion, which are attractors in the space of solutions, exist during the radiation and matter-dominated eras for k-essence in a similar manner to quintessence.

The speed of sound for k-essence fluctuation is

I.5.19 $$\begin{aligned} c_s^2 =\frac{p,_{X}}{p,_X+2Xp,_{XX}}. \end{aligned}$$

So that whenever the kinetic terms for the scalar field are not linear in *X*, the speed of sound of fluctuations differs from unity. It might appear concerning that superluminal fluctuations are allowed in k-essence models (and even necessarily arise in models where k-essence dark energy solves the coincidence problem Bonvin et al. 2006). However, it was shown in Babichev et al. (2008) that this does not lead to any causal paradoxes.

#### Coupled dark-energy models

A first class of models in which dark energy shows dynamics, in connection with the presence of a fifth force different from gravity, is the case of ‘interacting dark energy’: we consider the possibility that dark energy, seen as a dynamical scalar field, may interact with other components in the universe. This class of models effectively enters in the “explicit modified gravity models” in the classification above, because the gravitational attraction between dark matter particles is modified by the presence of a fifth force. However, we note that the anisotropic stress for DE is still zero in the Einstein frame, while it is, in general, non-zero in the Jordan frame. In some cases (when a universal coupling is present) such an interaction can be explicitly recast in a non-minimal coupling to gravity, after a redefinition of the metric and matter fields (Weyl scaling). We would like to identify whether interactions (couplings) of dark energy with matter fields, neutrinos or gravity itself can affect the universe in an observable way.

In this subsection, we give a general description of the following main interacting scenarios:

1. couplings between dark energy and baryons;
2. couplings between dark energy and dark matter (coupled quintessence);
3. couplings between dark energy and neutrinos (growing neutrinos, MaVaNs);
4. universal couplings with all species (scalar–tensor theories and *f*(*R*)).

In all these cosmologies the coupling introduces a fifth force, in addition to standard gravitational attraction. The presence of a new force, mediated by the DE scalar field (sometimes called the ‘cosmon’ Wetterich 1988, seen as the mediator of a cosmological interaction) has several implications and can significantly modify the process of structure formation. We will discuss cases (2) and (3) in Sect. II.

In these scenarios the presence of the additional interaction couples the evolution of components that in the standard $\varLambda$-FLRW would evolve independently. The stress–energy tensor ${T{^{\mu }}}_{\nu }$ of each species is, in general, not conserved—only the total stress–energy tensor is. Usually, at the level of the Lagrangian, the coupling is introduced by allowing the mass *m* of matter fields to depend on a scalar field $\phi$ via a function $m(\phi )$ whose choice specifies the interaction. This wide class of cosmological models can be described by the following action:

I.5.20 $$\begin{aligned} {{\mathcal {S}}} = \int {\mathrm {d}^4x \sqrt{-g} \left[ -\,\frac{1}{2}\partial ^\mu \phi \partial _\mu \phi - U(\phi ) - m(\phi )\bar{\psi }\psi + {{\mathcal {L}}}_{\mathrm {kin}}[\psi ]\right] }, \end{aligned}$$

where $U(\phi )$ is the potential in which the scalar field $\phi$ rolls, $\psi$ describes matter fields, and *g* is defined in the usual way as the determinant of the metric tensor, whose background expression is $g_{\mu \nu } = \mathrm {diag}[-\,a^2, a^2, a^2, a^2]$.

For a general treatment of background and perturbation equations we refer to Kodama and Sasaki (1984), Amendola (2000a, 2004) and Pettorino and Baccigalupi (2008). Here the coupling of the dark-energy scalar field to a generic matter component (denoted by index $\alpha$) is treated as an external source $Q_{(\alpha )\mu }$ in the Bianchi identities:

I.5.21 $$\begin{aligned} \nabla _{\nu }T_{(\alpha )\mu }^{\nu }=Q_{(\alpha )\mu }, \end{aligned}$$

with the constraint

I.5.22 $$\begin{aligned} \sum _{\alpha }Q_{(\alpha )\mu }=0 .\end{aligned}$$

The zero component of (I.5.21) gives the background conservation equations:

I.5.23 $$\begin{aligned} \frac{\mathrm {d}\rho _{\phi }}{\mathrm {d}\eta } = -\,3 {{\mathcal {H}}} (1 + w_\phi ) \rho _{\phi } + \beta (\phi ) \frac{\mathrm {d}\phi }{\mathrm {d}\eta } (1-3 w_{\alpha }) \rho _{\alpha }, \end{aligned}$$

I.5.24 $$\begin{aligned} \frac{\mathrm {d}\rho _{\alpha }}{\mathrm {d}\eta } = -\,3 {{\mathcal {H}}} (1 + w_{\alpha }) \rho _{\alpha } - \beta (\phi ) \frac{\mathrm {d}\phi }{\mathrm {d}\eta } (1-3 w_{\alpha }) \rho _{\alpha }, \end{aligned}$$

for a scalar field $\phi$ coupled to one single fluid $\alpha$ with a function $\beta (\phi )$, which in general may not be constant. The choice of the mass function $m(\phi )$ corresponds to a choice of $\beta (\phi )$ and equivalently to a choice of the source $Q_{(\alpha )\mu }$ and specifies the strength of the coupling according to the following relations:

I.5.25 $$\begin{aligned} Q_{(\phi )\mu }=\frac{\partial \ln {m(\phi )}}{\partial \phi } T_{\alpha }\,\partial _{\mu }\phi , \, m_{\alpha }=\bar{m}_\alpha ~ e^{-{\beta (\phi )}{\phi }}, \end{aligned}$$

where $\bar{m}_\alpha$ is the constant Jordan-frame bare mass. The evolution of dark energy is related to the trace $T_{\alpha }$ and, as a consequence, to density and pressure of the species $\alpha$. We note that a description of the coupling via an action such as (I.5.20) is originally motivated by the wish to modify GR with an extension such as scalar–tensor theories. In general, one of more couplings (Brookfield et al. 2008) can be active.

As for perturbation equations, it is possible to include the coupling in a modified Euler equation:

I.5.26 $$\begin{aligned}&\frac{\mathrm {d}\mathbf {v}_{\alpha }}{\mathrm {d}\eta } + \left( {{\mathcal {H}}} - {\beta (\phi )} \frac{\mathrm {d}\phi }{\mathrm {d}\eta } \right) \mathbf {v}_{\alpha } - \varvec{\nabla } \left[ \varPhi _\alpha + \beta \phi \right] = 0. \end{aligned}$$

The Euler equation in cosmic time ($\mathrm {d}t = a\, \mathrm {d}\tau$) can also be rewritten in the form of an acceleration equation for particles at position $\mathbf {r}$:

I.5.27 $$\begin{aligned} \dot{\mathbf {v}}_{\alpha } = -\,\tilde{H}\mathbf {v}_{\alpha } - \varvec{\nabla }\frac{\tilde{G}_{\alpha }{m}_{\alpha }}{r}. \end{aligned}$$

The latter expression explicitly contains all the main ingredients that affect dark-energy interactions:

1. a fifth force $\varvec{\nabla } \left[ \varPhi _\alpha + \beta \phi \right]$ with an effective $\tilde{G}_{\alpha } = G_{N}[1+2\beta ^2(\phi )]$;
2. a velocity dependent term $\tilde{H}\mathbf {v}_{\alpha } \equiv H \left( 1 - {\beta (\phi )} \frac{\dot{\phi }}{H}\right) \mathbf {v}_{\alpha }$
3. a time-dependent mass for each particle $\alpha$, evolving according to (I.5.25).

The relative significance of these key ingredients can lead to a variety of potentially observable effects, especially on structure formation. We will recall some of them in the following subsections as well as, in more detail, for two specific couplings in the dark matter Sects. II.10, II.8 of this report.

*I.5.3.1 Dark energy and baryons*

A coupling between dark energy and baryons is active when the baryon mass is a function of the dark-energy scalar field: $m_b = m_b(\phi )$. Such a coupling is constrained to be very small: main bounds come from tests of the equivalence principle and solar system constraints (Bertotti et al. 2003). More in general, depending on the coupling, bounds on the variation of fundamental constants over cosmological time-scales may have to be considered (Marra and Rosati 2005; Dent et al. 2008, 2009; Martins et al. 2010, and references therein). It is presumably very difficult to have significant cosmological effects due to a coupling to baryons only. However, uncoupled baryons can still play a role in the presence of a coupling to dark matter (see Sect. I.6 on nonlinear aspects).

*I.5.3.2 Dark energy and dark matter*

An interaction between dark energy and dark matter (CDM) is active when CDM mass is a function of the dark-energy scalar field: $m_c = m_c(\phi )$. In this case the coupling is not affected by tests on the equivalence principle and solar-system constraints and can therefore be stronger than the one with baryons. One may argue that dark-matter particles are themselves coupled to baryons, which leads, through quantum corrections, to direct coupling between dark energy and baryons. The strength of such couplings can still be small and was discussed in Dent et al. (2009) for the case of neutrino–dark-energy couplings. Also, quantum corrections are often recalled to spoil the flatness of a quintessence potential. However, it may be misleading to calculate quantum corrections up to a cutoff scale, as contributions above the cutoff can possibly compensate terms below the cutoff, as discussed in Wetterich (2008).

Typical values of $\beta$ presently allowed by observations (within current CMB data) are within the range $0< \beta < 0.06$ (at 95% CL for a constant coupling and an exponential potential) (Bean et al. 2008b; Amendola et al. 2003b; Amendola 2004; Amendola and Quercellini 2003), or possibly more (La Vacca et al. 2009; Kristiansen et al. 2010) if neutrinos are taken into account or for more realistic time-dependent choices of the coupling. This framework is generally referred to as ‘coupled quintessence’ (CQ). Various choices of couplings have been investigated in literature, including constant and varying $\beta (\phi )$ (Amendola 2000a; Mangano et al. 2003; Amendola 2004; Koivisto 2005; Guo et al. 2007; Quartin et al. 2008; Quercellini et al. 2008; Pettorino and Baccigalupi 2008; Gannouji et al. 2010; Pourtsidou et al. 2013) or within a PPF formalism (Skordis et al. 2015).

The presence of a coupling (and therefore, of a fifth force acting among dark-matter particles) modifies the background expansion and linear perturbations (Amendola 2000b, a, 2004), therefore, affecting CMB and cross-correlation of CMB and LSS (Amendola and Quercellini 2003; Amendola 2004; Amendola et al. 2003b; Amendola and Quercellini 2004; Bean et al. 2008b; La Vacca et al. 2009; Kristiansen et al. 2010; Xia 2009; Mainini and Mota 2012; Amendola et al. 2011).

Furthermore, structure formation itself is modified (Macciò et al. 2004; Manera and Mota 2006; Koivisto 2005; Mainini and Bonometto 2006; Sutter and Ricker 2008; Abdalla et al. 2009; Mota 2008; Bertolami et al. 2009; Wintergerst and Pettorino 2010; Baldi et al. 2010; Baldi 2011b, a; Baldi and Pettorino 2011; Baldi and Viel 2010; Li et al. 2011; Li and Barrow 2011b; Zhao et al. 2010; Marulli et al. 2012).

An alternative approach, also investigated in the literature (Mangano et al. 2003; Väliviita et al. 2008, 2010; Majerotto et al. 2010; Gavela et al. 2009, 2010; Caldera-Cabral et al. 2009b; Schaefer et al. 2008; Caldera-Cabral et al. 2009a), where the authors consider as a starting point Eq. (I.5.21): the coupling is then introduced by choosing directly a covariant stress–energy tensor on the RHS of the equation, treating dark energy as a fluid and in the absence of a starting action. The advantage of this approach is that a good parameterization allows us to investigate several models of dark energy at the same time. Problems connected to instabilities of some parameterizations or to the definition of a physically-motivated speed of sound for the density fluctuations can be found in Väliviita et al. (2008). It is also possible to both take a covariant form for the coupling and a quintessence dark-energy scalar field, starting again directly from Eq. (I.5.21). This has been done, e.g., in Boehmer et al. (2008) and Boehmer et al. (2010). At the background level only, Chimento et al. (2003), Chimento and Pavon (2006), del Campo et al. (2006), and Olivares et al. (2006) have also considered which background constraints can be obtained when starting from a fixed present ratio of dark energy and dark matter. The disadvantage of this approach is that it is not clear how to perturb a coupling that has been defined as a background quantity.

A Yukawa-like interaction was investigated (Farrar and Peebles 2004; Das et al. 2006), pointing out that coupled dark energy behaves as a fluid with an effective equation of state $w \lesssim -1$, though staying well defined and without the presence of ghosts (Das et al. 2006).

For an illustration of observable effects related to dark-energy–dark-matter interaction see also Sect. (II.10) of this report.

*I.5.3.3 Dark energy and neutrinos*

A coupling between dark energy and neutrinos can be even stronger than the one with dark matter and as compared to gravitational strength. Typical values of $\beta$ are order 50–100 or even more, such that even the small fraction of cosmic energy density in neutrinos can have a substantial influence on the time evolution of the quintessence field. In this scenario neutrino masses change in time, depending on the value of the dark-energy scalar field $\phi$. Such a coupling has been investigated within MaVaNs (Fardon et al. 2004; Peccei 2005; Bi et al. 2005; Afshordi et al. 2005; Weiner and Zurek 2006; Das and Weiner 2011; Takahashi and Tanimoto 2006; Spitzer 2006; Bjælde et al. 2008; Brookfield et al. 2006a, b) and more recently within growing neutrino cosmologies (Amendola et al. 2008a; Wetterich 2007; Mota et al. 2008; Wintergerst et al. 2010; Wintergerst and Pettorino 2010; Pettorino et al. 2010; Brouzakis et al. 2011; Baldi et al. 2011). In this latter case, DE properties are related to the neutrino mass and to a cosmological event, i.e., neutrinos becoming non-relativistic. This leads to the formation of stable neutrino lumps (Mota et al. 2008; Wintergerst et al. 2010; Baldi et al. 2011) at very large scales only ($\sim$ 100 Mpc and beyond) as well as to signatures in the CMB spectra (Pettorino et al. 2010). For an illustration of observable effects related to this case see Sect. II.8 of this report.

*I.5.3.4 Scalar–tensor theories*

Scalar–tensor theories (Wetterich 1988; Hwang 1990a, b; Damour et al. 1990; Casas et al. 1991, 1992; Wetterich 1995; Uzan 1999; Perrotta et al. 2000; Faraoni 2000; Boisseau et al. 2000; Riazuelo and Uzan 2002; Perrotta and Baccigalupi 2002; Schimd et al. 2005; Matarrese et al. 2004; Pettorino et al. 2005a, b; Capozziello et al. 2007; Appleby and Weller 2010) extend GR by introducing a non-minimal coupling between a scalar field (acting also as dark energy) and the metric tensor (gravity); they are also sometimes referred to as ‘extended quintessence’. We include scalar–tensor theories among ‘interacting cosmologies’ because, via a Weyl transformation, they are equivalent to a GR framework (minimal coupling to gravity) in which the dark-energy scalar field $\phi$ is coupled (universally) to all species (Wetterich 1988; Maeda 1989; Wands 1994; Esposito-Farèse and Polarski 2001; Pettorino and Baccigalupi 2008; Catena et al. 2007). In other words, these theories correspond to the case where, in action (I.5.20), the mass of all species (baryons, dark matter, ...) is a function $m=m(\phi )$ with the same coupling for every species $\alpha$. Indeed, a description of the coupling via an action such as (I.5.20) is originally motivated by extensions of GR such as scalar–tensor theories. Typically the strength of the scalar-mediated interaction is required to be orders of magnitude weaker than gravity (Lee 2010; Pettorino et al. 2005a and references therein for recent constraints). It is possible to tune this coupling to be as small as is required—for example by choosing a suitably flat potential $V(\phi )$ for the scalar field. However, this leads back to naturalness and fine-tuning problems.

In Sects. I.5.4 and I.5.5, we will discuss in more detail a number of ways in which new scalar degrees of freedom can naturally couple to standard model fields, while still being in agreement with observations. We mention here only that the presence of chameleon mechanisms (Brax et al. 2004, 2008, 2010a; Mota and Winther 2011; Mota and Shaw 2007; Hui et al. 2009; Davis et al. 2012b) can, for example, modify the coupling depending on the environment. In this way, a small (screened) coupling in high-density regions, in agreement with observations, is still compatible with a bigger coupling ($\beta \sim 1$) active in low density regions. In other words, a dynamical mechanism ensures that the effects of the coupling are screened in laboratory and solar system tests of gravity.

Typical effects of scalar–tensor theories on CMB and structure formation include:

- enhanced ISW (Pettorino et al. 2005a; Giannantonio 2009; Zhao et al. 2010);
- violation of the equivalence principle: extended objects such as galaxies do not all fall at the same rate (Amendola and Quercellini 2004; Hui et al. 2009).

However, it is important to remark that screening mechanisms are meant to protect the scalar field in high-density regions (and therefore allow for bigger couplings in low density environments) but they do not address problems related to self-acceleration of the DE scalar field, which still usually require some fine-tuning to match present observations on *w*. *f*(*R*) theories, which can be mapped into a subclass of scalar–tensor theories, will be discussed in more detail in Sect. I.5.4.

#### *f(R)* gravity

In parallel to models with extra degrees of freedom in the matter sector, such as interacting quintessence (and k-essence, not treated here), another promising approach to the late-time acceleration enigma is to modify the left-hand side of the Einstein equations and invoke new degrees of freedom, belonging this time to the gravitational sector itself. One of the simplest and most popular extensions of GR and a known example of modified gravity models is the *f*(*R*) gravity in which the 4-dimensional action is given by some generic function *f*(*R*) of the Ricci scalar *R* (for an introduction see, e.g., Amendola and Tsujikawa 2010):

I.5.28 $$\begin{aligned} S=\frac{1}{2\kappa ^{2}}\int {\mathrm {d}}^{4}x\sqrt{-g}f(R)+S_{m}(g_{\mu \nu },\varPsi _{m}) , \end{aligned}$$

where as usual $\kappa ^{2}=8\pi G$, and $S_{m}$ is a matter action with matter fields $\varPsi _{m}$. Here *G* is a *bare* gravitational constant: we will see that the observed value will in general be different. As mentioned in the previously, it is possible to show that *f*(*R*) theories can be mapped into a subset of scalar–tensor theories and, therefore, to a class of interacting scalar field dark-energy models universally coupled to all species. When seen in the Einstein frame (Wetterich 1988; Maeda 1989; Wands 1994; Esposito-Farèse and Polarski 2001; Pettorino and Baccigalupi 2008; Catena et al. 2007), action (I.5.28) can, therefore, be related to the action (I.5.20) shown previously. Here we describe *f*(*R*) in the Jordan frame: the matter fields in $S_{m}$ obey standard conservation equations and, therefore, the metric $g_{\mu \nu }$ corresponds to the physical frame (which here is the Jordan frame).

There are two approaches to deriving field equations from the action (I.5.28).

- **(I) The metric formalism**
  The first approach is the *metric formalism* in which the connections $\varGamma _{\beta \gamma }^{\alpha }$ are the usual connections defined in terms of the metric $g_{\mu \nu }$. The field equations can be obtained by varying the action (I.5.28) with respect to $g_{\mu \nu }$:
  I.5.29 $$\begin{aligned} F(R)R_{\mu \nu }(g)-\frac{1}{2}f(R)g_{\mu \nu }-\nabla _{\mu }\nabla _{\nu }F(R)+g_{ \mu \nu }\square F(R)=\kappa ^{2}T_{\mu \nu }, \end{aligned}$$
  where $F(R)\equiv \partial f/\partial R$ (we also use the notation $f_{,R}\equiv \partial f/\partial R,\, f_{,RR}\equiv \partial ^{2}f/\partial R^{2}$), and $T_{\mu \nu }$ is the matter energy–momentum tensor. The trace of Eq. (I.5.29) is given by
  I.5.30 $$\begin{aligned} 3\,\square F(R)+F(R)R-2f(R)=\kappa ^{2}T, \end{aligned}$$
  where $T=g^{\mu \nu }T_{\mu \nu }=-\,\rho +3P$. Here $\rho$ and *P* are the energy density and the pressure of the matter, respectively.
- **(II) The Palatini formalism**
  The second approach is the *Palatini formalism*, where $\varGamma _{\beta \gamma }^{\alpha }$ and $g_{\mu \nu }$ are treated as independent variables. Varying the action (I.5.28) with respect to $g_{\mu \nu }$ gives
  I.5.31 $$\begin{aligned} F(R)R_{\mu \nu }(\varGamma )-\frac{1}{2}f(R)g_{\mu \nu }=\kappa ^{2}T_{\mu \nu }, \end{aligned}$$
  where $R_{\mu \nu }(\varGamma )$ is the Ricci tensor corresponding to the connections $\varGamma _{\beta \gamma }^{\alpha }$. In general this is different from the Ricci tensor $R_{\mu \nu }(g)$ corresponding to the metric connections. Taking the trace of Eq. (I.5.31), we obtain
  I.5.32 $$\begin{aligned} F(R)R-2f(R)=\kappa ^{2}T, \end{aligned}$$
  where $R(T)=g^{\mu \nu }R_{\mu \nu }(\varGamma )$ is directly related to *T*. Taking the variation of the action (I.5.28) with respect to the connection, and using Eq. (I.5.31), we find
  I.5.33 $$\begin{aligned} R_{\mu \nu }(g)-\frac{1}{2}g_{\mu \nu }R(g)= & {} \frac{\kappa ^{2}T_{\mu \nu }}{F}-\frac{FR(T)-f}{2F}g_{\mu \nu } +\frac{1}{F}(\nabla _{\mu }\nabla _{\nu }F-g_{\mu \nu }\square F)\nonumber \\&-\frac{3}{2F^{2}}\left[ \partial _{\mu }F\partial _{\nu }F -\frac{1}{2}g_{\mu \nu }(\nabla F)^{2}\right] . \end{aligned}$$

In GR we have $f(R)=R-2\varLambda$ and $F(R)=1$, so that the term $\square F(R)$ in Eq. (I.5.30) vanishes. In this case both the metric and the Palatini formalisms give the relation $R=-\,\kappa ^{2}T=\kappa ^{2}(\rho -3P)$, which means that the Ricci scalar *R* is directly determined by the matter (the trace *T*).

In modified gravity models where *F*(*R*) is a function of *R*, the term $\square F(R)$ does not vanish in Eq. (I.5.30). This means that, in the metric formalism, there is a propagating scalar degree of freedom, $\psi \equiv F(R)$. The trace Eq. (I.5.30) governs the dynamics of the scalar field $\psi$—dubbed “scalaron” (Starobinsky 1980). In the Palatini formalism the kinetic term $\square F(R)$ is not present in Eq. (I.5.32), which means that the scalar-field degree of freedom does not propagate freely (Amarzguioui et al. 2006; Li et al. 2007, 2009, 2008b).

The de Sitter point corresponds to a vacuum solution at which the Ricci scalar is constant. Since $\square F(R)=0$ at this point, we get

I.5.34 $$\begin{aligned} F(R)R-2f(R)=0, \end{aligned}$$

which holds for both the metric and the Palatini formalisms. Since the model $f(R)=\alpha R^{2}$ satisfies this condition, it possesses an exact de Sitter solution (Starobinsky 1980).

It is important to realize that the dynamics of *f*(*R*) dark-energy models is different depending on the two formalisms. Here we confine ourselves to the metric case only; details of a viable model in unifying the metric and Palatini formalism can be found in Harko et al. (2012).

Already in the early 1980s, it was known that the model $f(R)=R+\alpha R^{2}$ can be responsible for inflation in the early universe (Starobinsky 1980). This comes from the fact that the presence of the quadratic term $\alpha R^{2}$ gives rise to an asymptotically exact de Sitter solution. Inflation ends when the term $\alpha R^{2}$ becomes smaller than the linear term *R*. Since the term $\alpha R^{2}$ is negligibly small relative to *R* at the present epoch, this model is not suitable to realizing the present cosmic acceleration.

Since a late-time acceleration requires modification for small *R*, models of the type $f(R)=R-\alpha /R^{n}$ ($\alpha>0,n>0$) were proposed as a candidate for dark energy (Capozziello 2002; Carroll et al. 2004; Nojiri and Odintsov 2003). While the late-time cosmic acceleration is possible in these models, it has become clear that they do not satisfy local gravity constraints because of the instability associated with negative values of $f_{,RR}$ (Chiba 2003; Dolgov and Kawasaki 2003; Soussa and Woodard 2004; Olmo 2005; Faraoni 2006). Moreover a standard matter epoch is not present because of a large coupling between the Ricci scalar and the non-relativistic matter (Amendola et al. 2007b).

Then, we can ask what are the conditions for the viability of *f*(*R*) dark-energy models in the metric formalism. In the following we first present such conditions and then explain step by step why they are required.

- (i) $f_{,R}>0$ for $R\ge R_{0}~(>0)$, where $R_{0}$ is the Ricci scalar at the present epoch. Strictly speaking, if the final attractor is a de Sitter point with the Ricci scalar $R_{1}~(>0)$, then the condition $f_{,R}>0$ needs to hold for $R\ge R_{1}$.
  This is required to avoid a negative effective gravitational constant.
- (ii) $$f_{,RR}>0$$ for $$R\ge R_{0}$$.
  This is required for consistency with local gravity tests (Dolgov and Kawasaki 2003; Olmo 2005; Faraoni 2006; Navarro and Van Acoleyen 2007), for the presence of the matter-dominated epoch (Amendola et al. 2007b, a), and for the stability of cosmological perturbations (Carroll et al. 2006; Song et al. 2007a; Bean et al. 2007; Faulkner et al. 2007).
- (iii) $$f(R)\rightarrow R-2\varLambda $$ for $$R\gg R_{0}$$.
  This is required for consistency with local gravity tests (Amendola and Tsujikawa 2008; Hu and Sawicki 2007a; Starobinsky 2007; Appleby and Battye 2007; Tsujikawa 2008) and for the presence of the matter-dominated epoch (Amendola et al. 2007a).
- (iv) $$0<\frac{Rf_{,RR}}{f_{,R}}(r=-\,2)<1$$ at $$r=-\,\frac{Rf_{,R}}{f}=-\,2$$.
  This is required for the stability of the late-time de Sitter point (Müller et al. 1988; Amendola et al. 2007a).

For example, the model $f(R)=R-\alpha /R^{n}$ ($\alpha >0$, $n>0$) does not satisfy the condition (ii).

Below we list some viable *f*(*R*) models that satisfy the above conditions.

I.5.35 $$\begin{aligned}&{\mathrm {(A)}}~f(R)=R-\mu R_{c}(R{/}R_{c})^{p}\qquad {\mathrm {with}}~~0<p<1,~~\mu ,R_{c}>0, \end{aligned}$$

I.5.36 $$\begin{aligned}&{\mathrm {(B)}}~f(R)=R-\mu R_{c}\frac{(R{/}R_{c})^{2n}}{(R{/}R_{c})^{2n}+1}\qquad {\mathrm {with}}~~n,\mu ,R_{c}>0, \end{aligned}$$

I.5.37 $$\begin{aligned}&{\mathrm {(C)}}~f(R)=R-\mu R_{c}\left[ 1-\left( 1+R^{2}/R_{c}^{2}\right) ^{-n}\right] \qquad {\mathrm {with}}~~n,\mu ,R_{c}>0,\qquad \quad \end{aligned}$$

I.5.38 $$\begin{aligned}&{\mathrm {(D)}}~f(R)=R-\mu R_{c}{\mathrm {tanh}}\,(R{/}R_{c})\qquad {\mathrm {with}}~~\mu ,R_{c}>0. \end{aligned}$$

The models (A), (B), (C), and (D) have been proposed in Amendola et al. (2007a), Hu and Sawicki (2007a), Starobinsky (2007), and Tsujikawa (2008), respectively. A model similar to (D) has been also proposed in Appleby and Battye (2007), while a generalized model encompassing (B) and (C) has been studied in Miranda et al. (2009). In model (A), the power *p* needs to be close to 0 to satisfy the condition (iii). In models (B) and (C) the function *f*(*R*) asymptotically behaves as $f(R)\rightarrow R-\mu R_{c}[1-(R^{2}/R_{c}^{2})^{-n}]$ for $R\gg R_{c}$ and hence the condition (iii) can be satisfied even for $n={{\mathcal {O}}}(1)$. In model (D) the function *f*(*R*) rapidly approaches $f(R)\rightarrow R-\mu R_{c}$ in the region $R\gg R_{c}$. These models satisfy $f(R=0)=0$, so the cosmological constant vanishes in the flat spacetime.

Let us consider the cosmological dynamics of *f*(*R*) gravity in the metric formalism. It is possible to carry out a general analysis without specifying the form of *f*(*R*). In the flat FLRW spacetime the Ricci scalar is given by

I.5.39 $$\begin{aligned} R=6\left( 2H^{2}+\dot{H}\right) , \end{aligned}$$

where *H* is the Hubble parameter. As a matter action $S_{m}$ we take into account non-relativistic matter and radiation, which satisfy the usual conservation equations $\dot{\rho }_{m}+3H\rho _{m}=0$ and $\dot{\rho }_{r}+4H\rho _{r}=0$ respectively. From Eqs. (I.5.29) and (I.5.30) we obtain the following equations

I.5.40 $$\begin{aligned} 3FH^{2}= & {} \kappa ^{2}\,(\rho _{m}+\rho _{r})+(FR-f)/2-3H\dot{F}, \end{aligned}$$

I.5.41 $$\begin{aligned} -2F\dot{H}= & {} \kappa ^{2}\left[ \rho _{m}+(4/3)\rho _{r}\right] +{\ddot{F}}-H\dot{F}. \end{aligned}$$

We introduce the dimensionless variables:

I.5.42 $$\begin{aligned} x_{1}\equiv -\frac{\dot{F}}{HF},\quad x_{2}\equiv -\frac{f}{6FH^{2}},\quad x_{3}\equiv \frac{R}{6H^{2}},\quad x_{4}\equiv \frac{\kappa ^{2}\rho _{r}}{3FH^{2}}, \end{aligned}$$

together with the following quantities

I.5.43 $$\begin{aligned} \varOmega _{m}\equiv \frac{\kappa ^{2}\rho _{m}}{3FH^{2}}=1-x_{1}-x_{2}-x_{3}-x_{4} , \quad \varOmega _{r}\equiv x_{4},\quad \varOmega _{\mathrm {DE}}\equiv x_{1}+x_{2}+x_{3}.\quad \end{aligned}$$

It is straightforward to derive the following differential equations (Amendola et al. 2007a):

I.5.44 $$\begin{aligned} x_{1}'= & {} -1-x_{3}-3x_{2}+x_{1}^{2}-x_{1}x_{3}+x_{4}, \end{aligned}$$

I.5.45 $$\begin{aligned} x_{2}'= & {} \frac{x_{1}x_{3}}{m}-x_{2}\left( 2x_{3}-4-x_{1}\right) , \end{aligned}$$

I.5.46 $$\begin{aligned} x_{3}'= & {} -\frac{x_{1}x_{3}}{m}-2x_{3}\left( x_{3}-2\right) , \end{aligned}$$

I.5.47 $$\begin{aligned} x_{4}'= & {} -2x_{3}x_{4}+x_{1}x_{4}, \end{aligned}$$

where the prime denotes $\mathrm {d}/\mathrm {d}\ln a$ and

I.5.48 $$\begin{aligned} m\equiv & {} \frac{\mathrm {d}\ln F}{\mathrm {d}\ln R}=\frac{Rf_{,RR}}{f_{,R}}, \end{aligned}$$

I.5.49 $$\begin{aligned} r\equiv & {} -\frac{\mathrm {d}\ln f}{\mathrm {d}\ln R}=-\frac{Rf_{,R}}{f}=\frac{x_{3}}{x_{2}}. \end{aligned}$$

From Eq. (I.5.49) one can express *R* as a function of $x_{3}/x_{2}$. Since *m* is a function of *R*, it follows that *m* is a function of *r*, i.e., $m=m(r)$. The $\varLambda$CDM model, $f(R)=R-2\varLambda$, corresponds to $m=0$. Hence the quantity *m* characterizes the deviation from the $\varLambda$CDM model. Note also that the model, $f(R)=\alpha R^{1+m}-2\varLambda$, gives a constant value of *m*. The analysis using Eqs. (I.5.44)–(I.5.47) is sufficiently general in the sense that the form of *f*(*R*) does not need to be specified.

The effective equation of state of the system (i.e., $p_{\mathrm {tot}}/\rho _{\mathrm {tot}}$) is

I.5.50 $$\begin{aligned} w_{\mathrm {eff}}=-\frac{1}{3}(2x_{3}-1). \end{aligned}$$

The dynamics of the full system can be investigated by analyzing the stability properties of the critical phase-space points as in, e.g., Amendola et al. (2007a). The general conclusions is that only models with a characteristic function *m*(*r*) positive and close to $\varLambda$CDM, i.e., $m\ge 0$, are cosmologically viable. That is, only for these models one finds a sequence of a long decelerated matter epoch followed by a stable accelerated attractor.

The perturbation equations have been derived in, e.g., Hwang and Noh (2002) and Tsujikawa et al. (2008b). Neglecting the contribution of radiation one has

I.5.51 $$\begin{aligned}&\delta _{m}'' +\left( x_{3}-\frac{1}{2}x_{1}\right) \delta _{m}'-\frac{3}{2}(1-x_{1}-x_{2}-x_{3})\delta _{m}\nonumber \\&\quad =\frac{1}{2}\biggl [\left\{ \frac{k^{2}}{x_{5}^{2}}-6+3x_{1}^{2}-3x_{1}'-3x_{1}(x_{3}-1)\right\} \delta \tilde{F}\nonumber \\&\quad \quad +3(-2x_{1}+x_{3}-1)\delta \tilde{F}'+3\delta \tilde{F}''\biggr ], \end{aligned}$$

I.5.52 $$\begin{aligned}&\delta \tilde{F}'' +(1-2x_{1}+x_{3})\delta \tilde{F}'\nonumber \\&\quad \quad +\left[ \frac{k^{2}}{x_{5}^{2}}-2x_{3}+\frac{2x_{3}}{m}-x_{1}(x_{3}+1)-x_{1}'+x_{1}^{2}\right] \delta \tilde{F}\nonumber \\&\quad =(1-x_{1}-x_{2}-x_{3})\delta _{m}-x_{1}\delta _{m}', \end{aligned}$$

where $\delta \tilde{F}\equiv \delta F{/}F$, and the new variable $x_{5}\equiv aH$ satisfies

I.5.53 $$\begin{aligned} x_{5}'=(x_{3}-1)\, x_{5}. \end{aligned}$$

The perturbation $\delta F$ can be written as $\delta F=f_{,RR}\delta R$ and, therefore, $\delta \tilde{F}=m\delta R{/}R$. These equations can be integrated numerically to derive the behavior of $\delta _m$ at all scales. However, at sub-Hubble scales they can be simplified and the following expression for the two MG functions $Q,\eta$ of Eq. (I.3.23) can be obtained:

I.5.54 $$\begin{aligned} Q= & {} 1-{ \frac{k^{2}}{ 3\left( a^2M^2 + k^{2}\right) } }\nonumber \\ \eta= & {} 1- {\frac{2k^{2} }{3a^2M^2 + 4k^{2}} } \end{aligned}$$

where

I.5.55 $$\begin{aligned} M^{2} = {\frac{1}{3 f_{,RR}} }. \end{aligned}$$

Note that in the $\varLambda$CDM limit $f_{,RR}\rightarrow 0$ and $Q,\eta \rightarrow 1$.

These relations can be straightforwardly generalized. In De Felice et al. (2010), the perturbation equations for the *f*(*R*) Lagrangian have been extended to include coupled scalar fields and their kinetic energy $X\equiv -\phi _{,\mu }\phi ^{\mu }/2$, resulting in a $f(R,\phi ,X)$-theory. In the slightly simplified case in which $f(R,\phi ,X)=f_1(R,\phi )+f_2(\phi ,X)$, with arbitrary functions $f_1,2$, one obtains

I.5.56 $$\begin{aligned} Q= & {} -\frac{1}{F} \frac{(1+2r_1)(f_{,X}+2r_2)+2F_{,\phi }^2{/}F}{(1+3r_1)(f_{,X}+2r_2)+3F_{,\phi }^2{/}F},\nonumber \\ \eta= & {} \frac{(1+2r_1)(f_{,X}+2r_2)+2F_{,\phi }^2{/}F}{(1+4r_1)(f_{,X}+2r_2)+4F_{,\phi }^2{/}F}, \end{aligned}$$

where the notation $f_{,X}$ or $F_{,\phi }$ denote differentiation wrt *X* or $\phi$, respectively, and where $r_1 \equiv \frac{k^2}{a^{2}} \frac{m}{R}$ and $r_2 \equiv \frac{a^2}{k^2}M_\phi ^2\,$, $M_{\phi }=-\,f_{,\phi \phi }/2$ being the scalar field effective mass. In the same paper (De Felice et al. 2010) an extra term proportional to $X\Box \phi$ in the Lagrangian is also taken into account.

Euclid forecasts for the *f*(*R*) models will be presented in Sect. I.8.7.

#### Massive gravity and higher-dimensional models

Instead of introducing new scalar degrees of freedom such as in *f*(*R*) theories, another philosophy in modifying gravity is to modify the graviton itself. In this case the new degrees of freedom belong to the gravitational sector itself; examples include massive gravity and higher-dimensional frameworks, such as the Dvali–Gabadadze–Porrati (DGP) model (Dvali et al. 2000) and its extensions. The new degrees of freedom can be responsible for a late-time acceleration of the universe, as is summarized below for a choice of selected models. We note here that while such self-accelerating solutions are interesting in their own right, they do not tackle the old cosmological constant problem: why the observed cosmological constant is so much smaller than expected in the first place. Instead of answering this question directly, an alternative approach is the idea of degravitation (see Dvali et al. 2002, 2003; Arkani-Hamed et al. 2002; Dvali et al. 2007), where the cosmological constant could be as large as expected from standard field theory, but would simply gravitate very little (see the paragraph in Sect. I.5.5.2 below).

*I.5.5.1 Infrared modifications of gravity*

Infrared modifications of gravity are of great interest for cosmology as they can affect the evolution of the Universe in two different ways, via self-acceleration and degravitation, as illustrated below.

Self-acceleration

The first interest in modifications of gravity is the possibility of self-acceleration where the late-time acceleration of the Universe is not sourced by a cosmological constant or dark energy but rather by the graviton itself. This interesting phenomenology was first encountered in the DGP model as is explained below and was later shown to be also present in the Galileon, massive gravity and bi-gravity. Technically speaking if the Galileon is considered as a scalar field in its own right then the acceleration of the Universe is due to a new scalar degree of freedom and lies in the category of dark energy. However massive gravity and higher-dimensional models of gravity often behave as a Galileon model in some limit, where the Galileon plays the role of one of the graviton’s own degree of freedom, in this sense Galileon models are often also thought of models of self-acceleration.

Degravitation

The idea behind degravitation is to modify gravity in the IR, such that the vacuum energy could have a weaker effect on the geometry, and therefore reconcile a natural value for the vacuum energy as expected from particle physics with the observed late-time acceleration. Such modifications of gravity typically arise in models of massive gravity (Dvali et al. 2002, 2003, 2007; Arkani-Hamed et al. 2002), i.e., where gravity is mediated by a massive spin-2 field. The extra-dimensional DGP scenario presented below, represents a specific model of soft mass gravity, where gravity weakens at large distance, with a force law going as 1 / *r*. Nevertheless, this weakening is too weak to achieve degravitation and tackle the cosmological constant problem. However, an obvious way out is to extend the DGP model to higher dimensions, thereby diluting gravity more efficiently at large distances. This is achieved in models of cascading gravity, as is presented below. An alternative to cascading gravity is to work directly with theories of constant mass gravity (hard mass graviton).

*I.5.5.2 Models*

Infrared modifications of gravity usually weaken the effect of gravity on cosmological scales, i.e., the propagation of gravitational waves is affected at distances and time-scales that are of the order of the size and age of the current Universe. These infrared modifications of general relativity are united by the common feature of invoking new degrees of freedom which could be used to either explain the recent acceleration of the Hubble expansion or tackle the cosmological constant problem. Below we will discuss different models which share these features.

DGP

The DGP model is one of the important infrared (IR) modified theories of gravity. From a four-dimensional point of view this corresponds effectively to a theory in which the graviton acquires a soft mass *m*. In this braneworld model our visible universe is confined to a brane of four dimensions embedded into a five-dimensional bulk. At small distances, the four-dimensional gravity is recovered due to an intrinsic Einstein–Hilbert term sourced by the brane curvature causing a gravitational force law that scales as $r^{-2}$. At large scales the gravitational force law asymptotes to an $r^{-3}$ behavior. The cross over scale $r_c=m^{-1}$ is given by the ratio of the Planck masses in four ($M_4$) and five ($M_5$) dimensions. One can study perturbations around flat spacetime and compute the gravitational exchange amplitude between two conserved sources, which does not reduce to the GR result even in the limit m$\rightarrow 0$. However, the successful implementation of the Vainshtein mechanism for decoupling the additional modes from gravitational dynamics at sub-cosmological scales makes these theories still very attractive (Vainshtein 1972). Hereby, the Vainshtein effect is realized through the nonlinear interactions of the helicity-0 mode $\pi$, as will be explained in further detail below. Thus, this vDVZ discontinuity does not appear close to an astrophysical source where the $\pi$ field becomes nonlinear and these nonlinear effects of $\pi$ restore predictions to those of GR. This is most easily understood in the limit where $M_4, M_5\rightarrow \infty$ and $m\rightarrow 0$ while keeping the strong coupling scale $\varLambda =(M_4m^2)^{1/3}$ fixed. This allows us to treat the usual helicity-2 mode of gravity linearly while treating the helicity-0 mode $\pi$ nonlinearly. The resulting effective action is then

I.5.57 $$\begin{aligned} \mathcal {L}_{\pi }=3 \pi \Box \pi -\frac{1}{\varLambda ^3}(\partial \pi )^2 \Box \pi , \end{aligned}$$

where interactions already become important at the scale $\varLambda \ll M_{\mathrm {Pl}}$ (Luty et al. 2003).

Furthermore, in this model, one can recover an interesting range of cosmologies, in particular a modified Friedmann equation with a self-accelerating solution. The Einstein equations thus obtained reduce to the following modified Friedmann equation in a homogeneous and isotropic metric (Deffayet et al. 2002a)

I.5.58 $$\begin{aligned} H^2\pm m H=\frac{8\pi G}{3}\rho , \end{aligned}$$

such that at higher energies one recovers the usual four-dimensional behavior, $H^2\sim \rho$, while at later time corrections from the extra dimensions kick in. As is clear in this Friedmann equation, this braneworld scenario holds two branches of cosmological solutions with distinct properties. The self-accelerating branch (minus sign) allows for a de Sitter behavior $H={\mathrm {const}}=m$ even in the absence of any cosmological constant $\rho _{\varLambda }=0$ and as such it has attracted a lot of attention. Unfortunately, this branch suffers from a ghost-like instability. The normal branch (the plus sign) instead slows the expansion rate but is stable. In this case a cosmological constant is still required for late-time acceleration, but it provides significant intuition for the study of degravitation.

The Galileon

Even though the DGP model is interesting for several reasons like giving the Vainshtein effect a chance to work, the self-acceleration solution unfortunately introduces extra ghost states as outlined above. However, it has been generalized to a “Galileon” model, which can be considered as an effective field theory for the helicity-0 field $\pi$. Galileon models are invariant under shifts of the field $\pi$ and shifts of the gradients of $\pi$ (known as the Galileon symmetry), meaning that a Galileon model is invariant under the transformation

I.5.59 $$\begin{aligned} \pi \rightarrow \pi + c +v_{\mu }x^{\mu }, \end{aligned}$$

for arbitrary constant *c* and $v_{\mu }$. In induced gravity braneworld models, this symmetry is naturally inherited from the five-dimensional Poincaré invariance (de Rham and Tolley 2010). The Galileon theory relies strongly on this symmetry to constrain the possible structure of the effective $\pi$ Lagrangian, and insisting that the effective field theory for $\pi$ bears no ghost-like instabilities further restricts the possibilities (Nicolis et al. 2009). It can be shown that there exist only five derivative interactions which preserve the Galilean symmetry in flat spacetime without introducing ghosts. In curved spacetimes the situation is more subtle, see Deffayet et al. (2009) for details. In flat spacetime, the interactions are symbolically of the form $\mathcal {L}_{\pi }^{(1)}=\pi$ and $\mathcal {L}_{\pi }^{(n)}=(\partial \pi )^2(\partial \partial \pi )^{n-2}$, for $n = 2,\ldots 5$. A general Galileon Lagrangian can be constructed as a linear combination of these Lagrangian operators. The effective action for the DGP scalar (I.5.57) can be seen to be a combination of $\mathcal {L}_{\pi }^{(2)}$ and $\mathcal {L}_{\pi }^{(3)}$. Such interactions have been shown to naturally arise from Lovelock invariants in the bulk of generalized braneworld models (de Rham and Tolley 2010). However, the Galileon does not necessarily require a higher-dimensional origin and can be consistently treated as a four-dimensional effective field theory.

As shown in Nicolis et al. (2009), such theories can allow for self-accelerating de Sitter solutions without any ghosts, unlike in the DGP model. In the presence of compact sources, these solutions can support spherically-symmetric, Vainshtein-like nonlinear perturbations that are also stable against small fluctuations. However, this is constrained to the subset of the third-order Galileon, which contains only $\mathcal {L}_{\pi }^{(1)}$, $\mathcal {L}_{\pi }^{(2)}$ and $\mathcal {L}_{\pi }^{(3)}$ (Mota et al. 2010).

The fact that they give rise to second order equations of motion, have a symmetry and allow for healthy self-accelerating solutions, have initiated a wealth of investigations in cosmology. Moreover the non-renormalization theorem makes them theoretically very interesting since once the parameters in the theory are tuned by observational constraints they are radiatively stable. This means that the coefficients governing the Galileon interactions are technically natural.

“Generalized galileons” and Horndeski interactions

The Galileon terms described above form a subset of the “generalized Galileons”. A generalized Galileon model allows nonlinear derivative interactions of the scalar field $\pi$ in the Lagrangian while insisting that the equations of motion remain at most second order in derivatives, thus removing any ghost-like instabilities. However, unlike the pure Galileon models, generalized Galileons do not impose the symmetry of Eq. (I.5.59). These theories were first written down by Horndeski (1974). They are a linear combination of Lagrangians constructed by multiplying the Galileon Lagrangians $\mathcal {L}_{\pi }^{(n)}$ by an arbitrary scalar function of the scalar $\pi$ and its first derivatives. Just like the Galileon, generalized Galileons can give rise to cosmological acceleration and to Vainshtein screening. However, as they lack the Galileon symmetry these theories are not protected from quantum corrections. The non-renormalization theorem is lost and hence the technical naturalness. Even if the naive covariantization of the Galileon interactions on non-flat backgrounds break the Galileon symmetry explicitly, one can successfully generalize the Galileon interactions to maximally symmetric backgrounds (Burrage et al. 2011; Trodden and Hinterbichler 2011). It is also worth mentioning that a given subclass of these Horndeski interactions can also be constructed within the context of massive gravity from covariantizing its decoupling limit (de Rham and Heisenberg 2011). Many other theories can also be found within the spectrum of generalized Galileon models, including k-essence. Recently, a new way to maintain a generalized Galileon symmetry on curved spacetimes was proposed in Gabadadze et al. (2012), Trodden (2012) by coupling massive gravity to a higher-dimensional DBI Galileon as in de Rham and Tolley (2010). In Hinterbichler et al. (2013), it was shown that such a generalized covariant Galileon model can lead to stable self-accelerating solutions.

Even if the scalar fields are by far the most extensively explored fields in cosmology, there are also motivations for the exploration of the role of vector fields or higher p-forms in general. Inspired by the Horndeski interactions of the scalar field, one can construct the most general vector-tensor interactions with non-minimal coupling giving rise to second order equations of motion (Jiménez et al. 2013).

Cascading gravity

Cascading gravity is an explicit realization of the idea of degravitation, where gravity behaves as a high-pass filter, allowing sources with characteristic wavelength (in space and in time) shorter than a characteristic scale $r_c$ to behave as expected from GR, but weakening the effect of sources with longer wavelengths. This could explain why a large cosmological constant does not backreact as much as anticipated from standard GR. Since the DGP model does not modify gravity enough in the IR, “cascading gravity” relies on the presence of at least two infinite extra dimensions, while our world is confined on a four-dimensional brane (de Rham et al. 2008b). Similarly as in DGP, four-dimensional gravity is recovered at short distances thanks to an induced Einstein–Hilbert term on the brane with associated Planck scale $M_4$. The brane we live in is then embedded in a five-dimensional brane, which bears a five-dimensional Planck scale $M_5$, itself embedded in six dimensions (with Planck scale $M_6$). From a four-dimensional perspective, the relevant scales are the 5d and 6d masses $m_4=M_5^3/M_4^2$ and $m_5=M_6^4/M_5^3$, which characterize the transition from the 4d–5d and 5d–6d behavior respectively.

Such theories embedded in more-than-one extra dimensions involve at least one additional scalar field that typically enters as a ghost. This ghost is independent of the ghost present in the self-accelerating branch of DGP but is completely generic to any codimension-two and higher framework with brane localized kinetic terms. However, there are two ways to cure the ghost, both of which are natural when considering a realistic higher codimensional scenario, namely smoothing out the brane, or including a brane tension (de Rham et al. 2008a, b, 2010).

When properly taking into account the issue associated with the ghost, such models give rise to a theory of massive gravity (soft mass graviton) composed of one helicity-2 mode, helicity-1 modes that decouple and 2 helicity-0 modes. In order for this theory to be consistent with standard GR in four dimensions, both helicity-0 modes should decouple from the theory. As in DGP, this decoupling does not happen in a trivial way, and relies on a phenomenon of strong coupling. Close enough to any source, both scalar modes are strongly coupled and therefore freeze.

The resulting theory appears as a theory of a massless spin-2 field in four-dimensions, in other words as GR. If $r\ll m_5$ and for $m_6\le m_5$, the respective Vainshtein scale or strong coupling scale, i.e., the distance from the source *M* within which each mode is strongly coupled is $r_{i}^3=M/m_i^2 M_4^2$, where $i=5,6$. Around a source *M*, one recovers four-dimensional gravity for $r\ll r_{5}$, five-dimensional gravity for $r_{5}\ll r \ll r_{6}$ and finally six-dimensional gravity at larger distances $r\gg r_{6}$.

The extension of Cascading gravity to higher dimensions also show the presence of solutions which allow for arbitrarily large cosmological constant without leading to any cosmic acceleration of the $3+1$ brane (de Rham et al. 2009), hence providing a first ingredient towards tackling the cosmological constant problem.

*I.5.5.3 Massive gravity and cosmological consequences*

While laboratory experiments, solar systems tests and cosmological observations have all been in complete agreement with GR for almost a century now, these bounds do not eliminate the possibility for the graviton to bear a small hard mass $m\lesssim 6.10^{-32}\mathrm {\ eV}$ (Goldhaber and Nieto 2010). The question of whether or not gravity could be mediated by a hard-mass graviton is not only a purely fundamental but could potentially have interesting observational implications and help with the late-time acceleration of the Universe and the original cosmological constant problem. Since the degravitation mechanism is also expected to be present if the graviton bears a hard mass, such models can play an important role for late-time cosmology, and more precisely when the age of the universe becomes on the order of the graviton Compton wavelength. See de Rham (2014) for a recent review on massive gravity and related models.

Lorentz invariant theories of hard massive gravity can be free of any ghost-like pathologies in the decoupling limit where $M_{\mathrm {Pl}}\rightarrow \infty$ and $m\rightarrow 0$ keeping the scale $\varLambda _{3}^3=M_{\mathrm {Pl}} m^2$ fixed (de Rham and Gabadadze 2010; de Rham et al. 2011b). The decoupling limit provides a good framework to understand the implications of a small graviton mass. Unlike a massless spin-2 field, which only bears two polarizations, a massive one bears five of them, namely two helicity-2 modes, two helicity-1 modes which decouple, and one helicity-0 mode (denoted as $\pi$). As in the braneworld models presented previously, this helicity-0 mode behaves as a scalar field with specific derivative interactions of the form

I.5.60 $$\begin{aligned} \mathcal {L}_{\pi }=h^{\mu \nu }\left( X^{(1)}_{\mu \nu }+\frac{1}{\varLambda _{3}^{3}} X^{(2)}_{\mu \nu }+\frac{1}{\varLambda _{3}^{6}} X^{(3)}_{\mu \nu }\right) . \end{aligned}$$

Here, $h_{\mu \nu }$ denotes the canonically-normalized (rescaled by $M_{\mathrm {pl}}$) tensor field perturbation (helicity-2 mode), while $X^{(1)}_{\mu \nu },X^{(2)}_{\mu \nu },$ and $X^{(3)}_{\mu \nu }$ are respectively, linear, quadratic and cubic in the helicity-0 mode $\pi$. Importantly, they are all transverse (for instance, $X^{(1)}_{\mu \nu }\propto \eta _ {\mu \nu }\square \pi - \partial _{\mu } \partial _{\nu } \pi$). Not only do these interactions automatically satisfy the Bianchi identity, as they should to preserve diffeomorphism invariance, but they are also at most second order in time derivatives. Hence, the interactions (I.5.60) are linear in the helicity-2 mode, and are free of any ghost-like pathologies. Therefore, such interactions are very similar in spirit to the Galileon ones, and bear the same internal symmetry (I.5.59), and present very similar physical properties. The stability of spherically symmetric configurations forces the $X^{(3)}_{\mu \nu }$ term to be absent (Berezhiani et al. 2013b). This represents a tuning of the parameters of the original theory but since these parameters are radiatively stable, this is not a self-tuning (de Rham et al. 2013a, b). In that case one recovers an Einstein frame picture for which the interactions are specific Galileon ones

I.5.61 $$\begin{aligned} \mathcal {L}= & {} \frac{M_{\mathrm {Pl}}^2}{2}\sqrt{-g}R +\frac{3}{2} \pi \Box \pi +\frac{3\beta }{2\varLambda _{3}^{3}}(\partial \pi )^{2} \Box \pi +\frac{\beta ^{2}}{2\varLambda _{3}^{6}}(\partial \pi )^{2}\left( (\partial _{\alpha }\partial _{\beta } \pi )^{2}-(\Box \pi )^{2} \right) \nonumber \\&+\mathcal {L}_{\mathrm {mat}}[\psi , {\tilde{g}}_{\mu \nu }], \end{aligned}$$

where $\beta$ is an arbitrary constant and matter fields $\psi$ do not couple to the metric $g_{\mu \nu }$ but to $\tilde{g}_{\mu \nu }=g_{\mu \nu }+\pi \eta _{\mu \nu }+\frac{\beta }{\varLambda _{3}^3} \partial _{\mu } \pi \partial _{\nu } \pi$. Here again, the recovery of GR in the UV is possible via a strong coupling phenomena, where the interactions for $\pi$ are already important at the scale $\varLambda _{3}\ll M_{\mathrm {Pl}}$, well before the interactions for the usual helicity-2 mode. This strong coupling, as well as the peculiar coupling to matter sources, have distinguishable features in cosmology as is explained below (Afshordi et al. 2009; Jain and Khoury 2010).

Spherically symmetric solutions in the decoupling limit were considered in Berezhiani et al. (2013a). Stability of this solutions requires the parameter $\beta$ to be positive definite which sets another constraint of the parameters of the original theory. Furthermore it was also shown that the solutions are asymptotic to a non-trivial FRW solution which is independent of the source at infinity. Notice however that these solutions are valid within the decoupling limit of massive gravity. At very large distances from the source, the decoupling limit is no longer valid, as the graviton mass takes over. At distances comparable to the graviton’s Compton wavelength one expects any solutions to reach a Yukawa-like type of behaviour and so the space–time to be asymptotically flat, although this has not been shown explicitly in any cosmological solution.

As in the studies of the spherically symmetric solutions mentioned above, a considerable amount of insight into the cosmological solutions can be gained from the decoupling limit analysis. Considering the de Sitter geometry as being a small perturbation about Minkowski space–time, one can construct self-accelerating solutions which are at leading order indistinguishable from a standard $\varLambda$CDM model. The helicity-0 degree of freedom of massive gravity forms a condensate whose energy density sources self-acceleration (de Rham et al. 2011a). However, as mentioned above, the solutions found in the decoupling limit could be considered just as a transient state of the full solution. In addition, the specific cosmological solution found in the decoupling limit suffers from pathologies since the vector fields lose their kinetic terms.

Beyond the decoupling limit, it has been shown that there is a no-go theorem against the existence of flat and closed FRW solutions, i.e. if the reference metric is chosen to be Minkowski then there is no flat/closed FRW solutions in the full theory beyond the decoupling limit (D’Amico et al. 2011b). The constraint needed for the absence of the Boulware–Deser ghost actually forbids the existence of homogeneous and isotropic cosmological solutions. Despite this no-go, there still exists non-FRW solutions that are approximately homogeneous and isotropic locally within domains of the size of inverse graviton mass. These solutions can be used to put constraints on the magnitude of the graviton mass coming from the consistency with known constraints on homogeneity and isotropy. This kind of solutions demands the successful implementation of the Vainshtein mechanism in the cosmological evolution which so far has not been investigated in detail in the literature.

The no-go theorem for the existence of flat/closed FRW solutions does not apply to the case of open FRW solutions (Gumrukcuoglu et al. 2011). Unfortunately, non-linear perturbations around this open FRW background are unstable making these solutions phenomenologically unviable.

A possible way out of these problems is to consider a more general reference metric. Indeed, if one takes the reference metric to be de Sitter, then one can construct FRW solutions. Nonetheless, these solutions bring other problems along due to the Higuchi bound, which imposes the mass of the graviton to be $m^2 > H^2$ which is in conflict with observational constraints in our cosmological past. Promoting the reference metric to a FRW metric leads to a generalized Higuchi bound and one encounters similar problems (Fasiello and Tolley 2013).

Finally another more natural possibility is the presence of inhomogeneous and even possibility anisotropies at large distance scales. Recently there has been a considerable amount of work devoted to this studies and it is beyond the scope of this review to detail them all. We simply refer to Volkov (2013) for a recent review and some of the most general solutions.

Such inhomogeneities/anisotropies are indeed to be expected on distance scales larger than the observable Universe. After all one of the main motivations of inflation is to ensure that such inhomogeneities/anisotropies are diluted in our observable Universe, but if inflation lasted a minimum number of e-folds such inhomogeneities/anisotropies would also be expected in General Relativity.

The first type of inhomogeneous solutions corresponds to the case where only the Stückelberg fields (or new degrees of freedom) carry order unity inhomogeneities while the metric remains isotropic and homogeneous. The inhomogeneities are then effectively unobservable since matter only couples to the metric and not directly to the Stückelberg fields.

Solutions where the metric itself carries explicit inhomogeneities while remaining isotropic have also been explored. These solutions can be constructed in such a way that the effective impact of the metric remains homogeneous and isotropic on short distance scales. In some of these cases, the mass term effectively plays the role of a cosmological constant leading to self-accelerating solutions.

Anisotropic solutions have been explored in Gumrukcuoglu et al. (2012) and subsequent litterature, for which the observed anisotropy remains small at short distance scales. The presence of the anisotropy also allow for stable self-accelerating solutions.

These represents special cases of exact solutions found in massive gravity although it is understood that the most general solution is likely to differ from these exact cases by carrying order one inhomogeneity or anisotropy or both at large distances which would requires numerical methods to be solved. This is still very much work in progress.

#### Beyond massive gravity: non-local models and multigravity

Different extensions of massive gravity have been introduced which could lead to an enriched phenomenology. First the mass can be promoted to a function of a new scalar field (Huang et al. 2012a). This allows for more interesting cosmology and some stable self-accelerating solutions. In this model the graviton mass could be effectively larger at earlier cosmological time, which implies that it can have an interesting phenomenology both at early and late times.

Another extension of massive gravity which also includes a new scalar field is the quasi-dilaton (D’Amico et al. 2013) and its extension (De Felice et al. 2013), where the extra scalar field satisfies a specific symmetry and its interactions are thus radiatively stable. In the original quasi-dilaton model the self-accelerating solution has a ghost and is unstable, however this issue is avoided in the extended quasi-dilaton proposed in De Felice et al. (2013). Moreover new types of stable self-accelerating solutions were recently found in Gabadadze et al. (2014). Similarly as in massive gravity, the decoupling limit solution must have a completion in the full theory although it might require some level of inhomogeneity at large distance scales, which are screened at small distance scales via the Vainshtein mechanism.

*I.5.6.1 Non-local models*

Different versions of massive gravity have been proposed in Maggiore (2014) and Maggiore and Mancarella (2014) (see also a previous model, Deser and Woodard 2007), based on a non-local modification of Einstein’s gravity that avoids the introduction of a second metric. In Maggiore and Mancarella (2014), in particular, the action has the form

I.5.62 $$\begin{aligned} \mathcal {L}=\frac{M_{\mathrm {pl}}^{2}}{2}R\left[ 1-\frac{m^{2}}{6}\left( \frac{1}{\Box }\right) ^{2}R\right] =\frac{M_{\mathrm {pl}}^{2}}{2}\left[ R-\frac{m^{2}}{6}\left( \frac{R}{\Box }\right) ^{2}\right] , \end{aligned}$$

This model can produce a nonlocal form of dark energy able to fit the background data while retaining a matter power spectrum compatible with observations (see, e.g., Foffa et al. 2014; Dirian et al. 2016). The $(R/\Box )^{2}$-correction to GR has indeed been obtained in an effective field theory for gravity at the second order curvature-expansion (Codello and Jain 2015).

*I.5.6.2 Bi- and multi-gravity*

Unlike DGP or cascading gravity, models of massive gravity require the presence of a reference metric. The dynamics of this reference metric can be included and leads to a model of bi-gravity where two metrics, say $g_{\mu \nu }$ and $f_{\mu \nu }$ with their own Einstein–Hilbert kinetic terms respectively $M_g^2\sqrt{-g}R[g_{\mu \nu }]$ and $M_f^2\sqrt{-f}R[f_{\mu \nu }]$ in addition to interactions between the two-metrics which takes precisely the same form as the potential term in massive gravity (Hassan and Rosen 2012). In this form bi-gravity was shown to be ghost free so long as different species of matter couple to either one of both metrics. The absence of ghost when some species couple to both metrics *f* and *g* at the same time has not been proven but is feasible.

Bi-gravity has two metrics and yet only one copy of diffeomorphism invariance. The second copy of diffeomorphism can be restored by introducing three Stückelberg fields similarly as in massive gravity and can be thought of as the three additional degrees of freedom in addition to the two degrees of freedom present in metric. This leads to a total of seven degrees of freedom: two in an effectively massless spin-2 field and five in an effectively massive spin-2 field. Notice that both the massive and the massless modes are a combination of $g_{\mu \nu }$ and $f_{\mu \nu }$.

Among these three additional degrees of freedom, one counts a helicity-0 mode which satisfies the same properties as in massive gravity. In particular this helicity-0 mode behaves as a Galileon in a similar decoupling limit and is screened via a Vainshtein mechanism.

The cosmology of bi-gravity was investigated for instance in Volkov (2012), von Strauss et al. (2012), Comelli et al. (2012a) and subsequent literature (see de Rham 2014 and Volkov 2013 for a review.) Unlike in massive gravity, both metrics can take a FLRW form and lead to an interesting new cosmology. For instance, in Akrami et al. (2013a, b) explicit self-accelerating solutions were provided in the absence of a cosmological constant. These solutions were tested against $\varLambda$CDM solutions using data from supernovae, CMB and large scale structure. For some parameters of the theory the best-fit chi-square is competitive to that of $\varLambda$CDM. The explicit Friedman equation for these parameters was derived in Fasiello and Tolley (2013)

I.5.63 $$\begin{aligned} H^2=\frac{1}{6M_g^2}\left( \rho +\sqrt{\rho ^2+\frac{12m^4 M_g^6}{M_f^2}}\right) , \end{aligned}$$

assuming that matter only couples to the metric $g_{\mu \nu }$, and has an effective energy density $\rho$. In this case the scale $M_g$ is essentially the Planck scale. The scale *m* governs the interactions between both metrics $g_{\mu \nu }$ and $f_{\mu \nu }$. In this case the self-accelerating solution can be shown to be stable.

Recently it has also been shown that a simple form of bi-gravity that depends on a single parameter (the minimal model) allows for stable self-accelerating solutions with distinguishable features from $\varLambda$CDM and an effective equation of state for small redshift $\omega (z)\approx -\,1.22 ^{+0.02}_{0.02} -0.64^{+0.05}_{-0.04}z/(1+z)$ (Könnig and Amendola 2014). At the linearly perturbed level, however, this model has been shown contain a gradient instability, ultimately due to the violation of the Higuchi bound. Linear perturbations in bimetric gravity have been studied extensively in Comelli et al. (2012b), Könnig and Amendola (2014), Solomon et al. (2014), Könnig et al. (2014), Lagos and Ferreira (2014), Cusin et al. (2015), Yamashita and Tanaka (2014), De Felice et al. (2014), Enander et al. (2015), Amendola et al. (2015), Johnson and Terrana (2015), and the models have been shown to contain either ghost or gradient instabilities. Cosmological solutions can be made stable back to arbitrarily early times by taking one Planck mass to be much smaller than the other (Akrami et al. 2015), or by reintroducing a cosmological constant which is much larger than the bimetric interaction parameter (Könnig and Amendola 2014). It is also possible that the gradient instability in bigravity is cured at the nonlinear level (Mortsell and Enander 2015) due to a version of the Vainshtein screening mechanism (Vainshtein 1972; Babichev and Deffayet 2013).

Bi-gravity was also shown to be extendable to an arbitrary number of interacting metrics in Hinterbichler and Rosen (2012), which would lead to multiple Galileon in its decoupling limit. In Lüben et al. (2016), several non-trivial cosmological solutions in a model with three metrics have been identified.

#### Effective field theory of dark energy

One of the most productive recent ideas in dark-energy cosmology has been the employing of effective field-theory methods originally developed for inflation (Creminelli et al. 2006, 2009; Cheung et al. 2008a) to limit the space of possible parameterisations of gravity to that obtainable from local actions with a fixed number of degrees of freedom and also to describe the perturbation evolution in different models of modified gravity using a common approach (Gubitosi et al. 2013; Bloomfield et al. 2013; Gleyzes et al. 2013).

We refer the reader to, e.g., the review by Gleyzes et al. (2015b) for details, here mentioning only the rough principles. The EFT of DE approach depends on choosing an FRW cosmological background as well as being able to pick one of the degrees of freedom of the model to be used as a clock on this background. This means that the approach is most directly applicable to models with at least one scalar degree of freedom, where the background configuration of the scalar field evolves monotonically (i.e., does not oscillate during the evolution). When this is possible, the scalar will play a role of a goldstone boson of the broken time symmetry in cosmology, its field value will define a time slicing (a unitary gauge). The symmetries of the FRW background must then also be the symmetries of the action which describes the evolution of fluctuations on the cosmological background: the action for perturbations must obey time-reparameterisation invariance and the remaining unbroken diffeomorphism invariance of the spatial slice.

The Arnowitt–Deser–Misner (ADM) $3+1$ split is the natural choice to employ in this approach. One forms from the full space–time metric $g_{\mu \nu }$ a three-dimensional spatial metric

$$\begin{aligned} h_{\mu \nu }=g_{\mu \nu }+u_{\mu }u_{\nu } \end{aligned}$$

by projecting out a time direction defined by the timelike scalar field gradient, $u_{\mu }\equiv -\partial _{\mu }\phi /\sqrt{-\partial _{\alpha }\phi \partial ^{\alpha }\phi }$. With this choice of slicing, one can then use the ADM coordinates, describing the metric through

$$\begin{aligned} \mathrm {d}s^{2}=-N^{2}\mathrm {d}t^{2}+h_{ij} \left( \mathrm {d}x^{i}+N^{i}\mathrm {d}t\right) \left( \mathrm {d}x^{j}+N^{j}\mathrm {d}t\right) , \end{aligned}$$

with *N* called the lapse and the vector $N^{i}$ the shift. In order to preserve the appropriate symmetries, the action for gravity and the goldstone boson must then be a scalar formed out of only these geometrical quantities and the spatial covariant derivative compatible with $h_{\mu \nu }$, $D_{i}$,

$$\begin{aligned} S_{g}=\int \mathrm {d}^{4}x\sqrt{-g}L \left( N,K_{ij},R_{ij},h_{ij},D_{i};t\right) , \end{aligned}$$

where $K_{ij}$ is the extrinsic curvature of the spatial slice and $R_{ij}$ its intrinsic curvature. If other degrees of freedom are present in the problem, then terms mixing these ADM geometrical quantities and the additional fields can also appear.

One then writes down various operators for the FRW background, the quadratic action for fluctuations and, in principle, higher-order actions, if non-linearities are of interest. The symmetry of the cosmological background is such that the coefficients of all these operators are allowed to be arbitrary functions of time *t*, but the resulting scale dependence is given by the particular operators in a fixed manner. Note that since the ADM curvatures do not contain time derivatives, but only spatial derivatives $D_{i}$, by using them, one does not introduce new degrees of freedom through higher time-derivatives. Thus one avoids this complication of the usual covariant approach for actions. However, higher-order spatial derivatives are generated through this approach.

The simplest application is to universally coupled scalar–tensor theories, modifications of gravity which contain no more than one extra scalar degree of freedom. For the background, the end result is that an arbitrary expansion history *H*(*t*) can be generated, and thus the background should be thought of as an input of the EFT approach (e.g., $w=-\,1$ or any other such choice). The question of determining the theory of gravity is then one of constraining the behaviour of perturbations and growth of structure on this chosen background. Linear structure formation is then determined by the quadratic action for fluctuations.

At quadratic order, one can write down multiple operators, thus a choice of basis for them must be made. A particularly useful one is the one introduced in Bellini and Sawicki (2014), so-called $\alpha$-functions, since it uses operators most closely related to physical properties of the dark energy. In this basis, the most general action for perturbations in a scalar–tensor theory which does not contain derivatives higher than second in the equation of motion for the propagating degrees of freedom can be written (Gleyzes et al. 2015b)

$$\begin{aligned} S_{2}&=\int \mathrm {d}t\mathrm {d}^{3}xa^{3}\frac{M_{*}^{2}}{2}\left[ \delta K_{ij}\delta K^{ij}-\delta K^{2}+\left( 1+\alpha _{\text {T}}\right) \delta _{2}R\right. \\&\quad \left. +\,\alpha _{\text {K}}H^{2}\delta N^{2}+4\alpha _{\text {B}}H\delta K\delta N+(1+\alpha _{\text {H}})\delta R\delta N\right] , \end{aligned}$$

where $M_{*}$ is the effective Planck mass and the $\alpha _{i}$ are all dimensionless functions of time that are in principle arbitrary and the $\delta$’s signify fluctuations of quantities away from their background value. The GR limit can be recovered by taking all the $\alpha _{i}\rightarrow 0$ and $M_{*}=\text {const}$. Other operators can be added to the the quadratic action, but they will invariably result in higher derivatives in the equations of motion.

The $\alpha$ functions play a role in modifying the properties of the perturbations and growth of structure. In particular they can be divided into two classes:

1. Non-minimal coupling of gravity. These functions modify both the scalar and the tensor propagation (Saltas et al. 2014):
  - $M_{*}^{2}(t)$*, the effective Planck mass. *$M_{*}^{2}$ is the normalisation of the kinetic term for gravitons. It encodes the strength of the gravitational force/space–time curvature produced by a fixed amount of energy. Large-scale structure is sensitive only to the time variation of the Planck mass,
    I.5.64 $$\begin{aligned} \alpha _{\text {M}}\equiv \frac{\mathrm {d}\ln M_{*}^{2}}{\mathrm {d}\ln a}, \end{aligned}$$
    or the *Planck-mass run rate*.
  - $\alpha _{\text {T}}(t)$, *tensor speed excess*. This parameter denotes the difference in the speed propagation of gravitational waves compared to the speed of light, i.e., $\alpha _{\text {T}}=c_{\text {T}}^{2}-1$. It applies to modes propagating on cosmological scales and is quite weakly constrained despite the recent detection from LIGO (Abbott et al. 2016; Blas et al. 2016; Creminelli and Vernizzi 2017; Ezquiaga and Zumalacárregui 2017; Crisostomi and Koyama 2017; Baker et al. 2017; Sakstein and Jain 2017).
2. Kinetic terms. The scalar mode is in addition affected by the following three functions:
  - $\alpha _{\text {K}}(t)$, *kineticity*. Coefficient of the kinetic term for the scalar d.o.f. before demixing (see Bellini and Sawicki 2014). Increasing this function leads to a relative increase of the kinetic terms compared to the gradient terms and thus a lower sound speed for the scalar field. This creates a sound horizon smaller than the cosmological horizon: super-sound-horizon the scalar does not have pressure support and clusters similarly to dust. Inside, it is arrested and eventually can enter a quasi-static configuration (Sawicki and Bellini 2015). When looking only at the quasi-static scales, inside the sound horizon, this function cannot be constrained (Gleyzes et al. 2016). This is the only term present in the simplest DE models, e.g. quintessence and in perfect-fluid dark energy.
  - $\alpha _{\text {B}}(t)$, *braiding*. This operator gives rise to a new mixing of the scalar field and the extrinsic curvature of the spatial metric, *K*. This leads to a modification of the coupling of matter to the curvature, independent and additional to any change in the Planck mass. This is typically interpreted as an additional fifth force between massive particles and can be approximated as a modification of the effective Newton’s constant for perturbations. It is present in archetypal modified gravity models such as *f*(*R*) gravity (see (Bellini and Sawicki 2014) for details). A purely conformal coupling of the scalar to gravity leads to the universal property $\alpha _{\text {M}}+\alpha _{\text {B}}=0$.
  - $\alpha _{\text {H}}(t)$, *beyond Horndeski*. This term is generated by a kinetic mixing of the scalar with the intrinsic curvature *R*. It results in third-order derivatives in the equations of motion, but which cancel once all the constraints are solved for. It produces a coupling of the gravitational field to the velocity of the matter.
  Note that either $\alpha _{\text {M}}$, $\alpha _{\text {T}}$ or $\alpha _{\text {H}}$ must not vanish in order for gravitational slip to be generated by perfect-fluid matter sources. such a case, the equation of motion for the propagation of gravitational waves is also modified.

In order for the chosen background *H*(*t*) to be stable in the model of gravity given by the choice of $\alpha$ functions, certain algebraic conditions on the $\alpha$ functions must be satisfied (the perturbations must not be ghosty and the sound speed squared needs to be positive, for both scalars and tensors; see e.g., Bellini and Sawicki (2014), Gleyzes et al. (2015b) for details).

In addition to providing a method for exploring new models of gravity, the EFT of DE approach allows one to describe linear perturbations in a very efficient and unified fashion. It is enough to obtain the appropriate functions *H*(*t*) and $\alpha _{i}$ for any particular model and structure formation in that model can be fully solved for. In particular, Horndeski models of gravity (or the generalised galileon models) determined by the four free functions of the scalar field $G_{2,3,4,5}$ can be encoded in the EFT language through (Bellini and Sawicki 2014)

I.5.65 $$\begin{aligned} M_{*}^{2}&\equiv 2\left( G_{4}-2XG_{4X}+XG_{5\phi }-\dot{\phi }HXG_{5X}\right) \end{aligned}$$

I.5.66 $$\begin{aligned} H^{2}M_{*}^{2}\alpha _{\text {K}}&\equiv 2X\left( K_{X}+2XK_{XX}-2G_{3\phi }-2XG_{3\phi X}\right) \end{aligned}$$

I.5.67 $$\begin{aligned}&\quad +\,12\dot{\phi }XH\left( G_{3X}+XG_{3XX}-3G_{4\phi X}-2XG_{4\phi XX}\right) \nonumber \\&\quad +\,12XH^{2}\left( G_{4X}+8XG_{4XX}+4X^{2}G_{4XXX}\right) \nonumber \\&\quad -\,12XH^{2}\left( G_{5\phi }+5XG_{5\phi X}+2X^{2}G_{5\phi XX}\right) \nonumber \\&\quad +\,4\dot{\phi }XH^{3}\left( 3G_{5X}+7XG_{5XX}+2X^{2}G_{5XXX}\right) \nonumber \\ HM_{*}^{2}\alpha _{\text {B}}&\equiv 2\dot{\phi }\left( XG_{3X}-G_{4\phi }-2XG_{4\phi X}\right) + \end{aligned}$$

I.5.68 $$\begin{aligned}&\quad +\,8XH\left( G_{4X}+2XG_{4XX}-G_{5\phi }-XG_{5\phi X}\right) \nonumber \\&\quad +\,2\dot{\phi }XH^{2}\left( 3G_{5X}+2XG_{5XX}\right) \nonumber \\ M_{*}^{2}\alpha _{\text {T}}&\equiv 2X\left( 2G_{4X}-2G_{5\phi }-\left( \ddot{\phi }-\dot{\phi }H\right) G_{5X}\right) \end{aligned}$$

I.5.69 $$\begin{aligned} \alpha _{\text {H}}&= 0 \end{aligned}$$

The operator $\alpha _{\text {H}}$ only appears in the so-called beyond Horndeski models introduced in Gleyzes et al. (2015a).

Since Horndeski theories include as subclasses the majority of the popular models of modified gravity, including perfect-fluid dark energy, linear structure formation in all these models can be solved for in a unified manner by obtaining these $\alpha$ functions. This method is now employed in the publicly available Boltzmann codes EFTCAMB (Hu et al. 2014), used also in the planck analysis on dark energy and modified gravity (Planck Collaboration 2016c), and hi_class (Zumalacárregui et al. 2017).

#### Observations and screening mechanisms

All models of modified gravity presented in this section have in common the presence of at least one additional helicity-0 degree of freedom that is not an arbitrary scalar, but descends from a full-fledged spin-two field. As such it has no potential and enters the Lagrangian via very specific derivative terms fixed by symmetries. However, tests of gravity severely constrain the presence of additional scalar degrees of freedom. Interestingly this degree of freedom would severly affect the behavior of voids and could potentially help reducing the tension between Planck and supernovae. Euclid could detect such an effect at the $5\sigma$ confidence level (Spolyar et al. 2013). Outside voids, as it is well known, in theories of massive gravity the helicity-0 mode can evade fifth-force constraints in the vicinity of matter if the helicity-0 mode interactions are important enough to freeze out the field fluctuations (Vainshtein 1972). This Vainshtein mechanism is similar in spirit but different in practice to the chameleon and symmetron mechanisms presented in detail below. One key difference relies on the presence of derivative interactions rather than a specific potential. So, rather than becoming massive in dense regions, in the Vainshtein mechanism the helicity-0 mode becomes weakly coupled to matter (and light, i.e., sources in general) at high energy. This screening of scalar mode can yet have distinct signatures in cosmology and in particular for structure formation.

Different classes of screening

While quintessence introduces a new degree of freedom to explain the late-time acceleration of the universe, the idea behind modified gravity is instead to tackle the core of the cosmological constant problem and its tuning issues as well as screening any fifth forces that would come from the introduction of extra degrees of freedom. As mentioned in Sect. I.5.3.1, the strength with which these new degrees of freedom can couple to the fields of the standard model is very tightly constrained by searches for fifth forces and violations of the weak equivalence principle. Typically the strength of the scalar mediated interaction is required to be orders of magnitude weaker than gravity. It is possible to tune this coupling to be as small as is required, leading however to additional naturalness problems. Here we discuss in more detail a number of ways in which new scalar degrees of freedom can naturally couple to standard model fields, whilst still being in agreement with observations, because a dynamical mechanism ensures that their effects are screened in laboratory and solar system tests of gravity. This is done by making some property of the field dependent on the background environment under consideration. These models typically fall into three classes; either the field becomes massive in a dense environment so that the scalar force is suppressed because the Compton wavelength of the interaction is small, or the coupling to matter becomes weaker in dense environments to ensure that the effects of the scalar are suppressed. The latter can be achieved either in regions of space–time where the coupling is dynamically driven to small values (the Damour–Polyakov mechanism, Damour and Polyakov 1994) or the wave function normalisation of the field becomes large (the K-mouflage, Babichev et al. 2009, or Vainshtein mechanisms). These types of behavior require the presence of nonlinearities. One can also see that the different types of screening mechanisms can be differentiated by a screening criterion (Khoury 2013) which requires the potential to be large (chameleon and Damour–Polyakov, Brax et al. 2012a), the gravitational acceleration (K-mouflage, Brax and Valageas 2014) or the local spatial curvature (Vainshtein).

Density dependent masses: the chameleon

The chameleon (Khoury and Weltman 2004) is the archetypal model of a scalar field with a mass that depends on its environment, becoming heavy in dense environments and light in diffuse ones. The ingredients for construction of a chameleon model are a conformal coupling between the scalar field and the matter fields of the standard model, and a potential for the scalar field, which includes relevant self-interaction terms.

In the presence of non-relativistic matter these two pieces conspire to give rise to an effective potential for the scalar field

I.5.70 $$\begin{aligned} V_{\mathrm {eff}}(\phi ) = V(\phi )+\rho A(\phi ), \end{aligned}$$

where $V(\phi )$ is the bare potential, $\rho$ the local energy density and $A(\phi )$ the conformal coupling function. For suitable choices of $A(\phi )$ and $V(\phi )$ the effective potential has a minimum and the position of the minimum depends on $\rho$. Self-interaction terms in $V(\phi )$ ensure that the mass of the field in this minimum also depends on $\rho$ so that the field becomes more massive in denser environments.

The environmental dependence of the mass of the field allows the chameleon to avoid the constraints of fifth-force experiments through what is known as the thin-shell effect. If a dense object is embedded in a diffuse background the chameleon is massive inside the object. There, its Compton wavelength is small. If the Compton wavelength is smaller than the size of the object, then the scalar mediated force felt by an observer at infinity is sourced, not by the entire object, but instead only by a thin shell of matter (of depth the Compton wavelength) at the surface. This leads to a natural suppression of the force without the need to fine tune the coupling constant.

The Vainshtein mechanism

In models such as DGP, the Galileon, Cascading gravity, massive gravity and bi- or multi-gravity, the effects of the scalar field(s) are screened by the Vainshtein mechanism (Vainshtein 1972; Deffayet et al. 2002b), see also Babichev and Deffayet (2013) for a recent review on the Vainshtein mechanism. This occurs when nonlinear, higher-derivative operators are present in the Lagrangian for a scalar field, arranged in such a way that the equations of motion for the field are still second order, such as the interactions presented in Eq. (I.5.57).

In the presence of a massive source the nonlinear terms force the suppression of the scalar force in the vicinity of a massive object. The radius within which the scalar force is suppressed is known as the Vainshtein radius. As an example in the DGP model the Vainshtein radius around a massive object of mass *M* is

I.5.71 $$\begin{aligned} r_{\star }\sim \left( \frac{M}{4\pi M_{\mathrm {Pl}}}\right) ^{1/3}\frac{1}{\varLambda }, \end{aligned}$$

where $\varLambda$ is the strong coupling scale introduced in Sect. I.5.5.2. For the Sun, if $m\sim 10^{-33}\mathrm {\ eV}$, or in other words, $\varLambda ^{-1}=1000\mathrm {\ km}$, then the Vainshtein radius is $r_{\star } \sim 10^2\mathrm {\ pc}$.

Inside the Vainshtein radius, when the nonlinear, higher-derivative terms become important they cause the kinetic terms for scalar fluctuations to become large. This can be interpreted as a relative weakening of the coupling between the scalar field and matter. In this way the strength of the interaction is suppressed in the vicinity of massive objects.

Related to the Vainshtein mechanism but slight more general is the screening via a disformal coupling between the scalar field and the stress–energy tensor $\partial _\mu \pi \partial _\nu \pi T^{\mu \nu }$ (Koivisto et al. 2012) as is present in DBI-braneworld types of models (de Rham and Tolley 2010) and massive gravity (de Rham and Gabadadze 2010).

The Symmetron

The symmetron model (Hinterbichler and Khoury 2010) is in many ways similar to the chameleon model discussed above. It requires a conformal coupling between the scalar field and the standard model and a potential of a certain form. In the presence of non-relativistic matter this leads to an effective potential for the scalar field

I.5.72 $$\begin{aligned} V_{\mathrm {eff}}(\phi ) =-\frac{1}{2}\left( \frac{\rho }{M^2}-\mu ^2\right) \phi ^2+\frac{1}{4}\lambda \phi ^4, \end{aligned}$$

where *M*, $\mu$ and $\lambda$ are parameters of the model, and $\rho$ is the local energy density.

In sufficiently dense environments, $\rho >\mu ^2M^2$, the field sits in a minimum at the origin. As the local density drops the symmetry of the field is spontaneously broken and the field falls into one of the two new minima with a non-zero vacuum expectation value. In high-density symmetry-restoring environments, the scalar field vacuum expectation value should be near zero and fluctuations of the field should not couple to matter. Thus, the symmetron force in the exterior of a massive object is suppressed because the field does not couple to the core of the object. This is an example of Damour–Polyakov mechanism.

The Olive–Pospelov model

The Olive–Pospelov model (Olive and Pospelov 2008) again uses a scalar conformally coupled to matter. In this construction both the coupling function and the scalar field potential are chosen to have quadratic minima. If the background field takes the value that minimizes the coupling function, then fluctuations of the scalar field decouple from matter. In non-relativistic environments the scalar field feels an effective potential, which is a combinations of these two functions. In high-density environments the field is very close to the value that minimizes the form of the coupling function. In low-density environments the field relaxes to the minimum of the bare potential. Thus, the interactions of the scalar field are suppressed in dense environments. This is another example of Damour–Polyakov mechanism.

#### Einstein Aether and its generalizations

Milgrom (1983) suggested that the emerging evidence for the presence of dark matter in galaxies could follow from a modification either to how ‘baryonic’ matter responded to the Newtonian gravitational field it created or to how the gravitational field was related to the baryonic matter density. Collectively these ideas are referred to as modified Newtonian dynamics (MOND). By way of illustration, MOND may be considered as a modification to the non-relativistic Poisson equation:

I.5.73 $$\begin{aligned} \nabla \cdot \left( \mu \left( \frac{|\nabla \varPsi |}{a_{0}}\right) \nabla \varPsi \right) = 4\pi G\rho , \end{aligned}$$

where $\varPsi$ is the gravitational potential, $a_{0}$ is a number with dimensions Length$^{-1}$ and $\rho$ is the baryonic matter density. The number $a_{0}$ is determined by looking at the dynamics of visible matter in galaxies (Sanders and McGaugh 2002). The function $\mu (x)$ would simply be equal to unity in Newtonian gravity. In MOND, the functional form is only fixed at its limits: $\mu \rightarrow 1$ as $x \rightarrow \infty$ and $\mu \rightarrow x$ as $x \rightarrow 0$.

We are naturally interested in a relativistic version of such a proposal. The building block is the perturbed spacetime metric already introduced in Eq. (I.3.8)

I.5.74 $$\begin{aligned} ds^{2}= -\,(1+2\varPsi )\, \mathrm {d}t^{2}+\left( 1-2\varPhi \right) a^{2}(t) \left( \mathrm {d}R^{2}+R^{2}\, \mathrm {d}\varOmega ^{2}\right) . \end{aligned}$$

A simple approach is to introduce a dynamical clock field, which we will call $A^{\mu }$. If it has solutions aligned with the time-like coordinate $t^{\mu }$ then it will be sensitive to $\varPsi$. The dynamical nature of the field implies that it should have an action that will contain gradients of the field and thus potentially scalars formed from gradients of $\varPsi$, as we seek. A family of covariant actions for the clock field is as follows (Zlosnik et al. 2007):

$$\begin{aligned} I \left[ g^{ab},A^{a},\lambda \right]= & {} \frac{1}{16\pi G} \int \mathrm {d}^4x \sqrt{-g} \left[ \frac{1}{\ell ^2} F(K) + \lambda \left( A^a A_a + 1 \right) \right] , \end{aligned}$$

where

I.5.75 $$\begin{aligned} K = \ell ^2 K^{\mu \nu \gamma \delta } \nabla _\mu A_\nu \nabla _\gamma A_\delta \end{aligned}$$

with

I.5.76 $$\begin{aligned} K^{\mu \nu \gamma \delta } = c_1 g^{\mu \gamma } g^{\nu \delta } + c_2 g^{\mu \nu } g^{\gamma \delta } + c_3 g^{\mu \delta } g^{\nu \delta }. \end{aligned}$$

The quantity $\ell$ is a number with dimensions of length, the $c_{A}$ are dimensionless constants, the Lagrange multiplier field $\lambda$ enforces the unit-timelike constraint on $A^{a}$, and *F* is a function. These models have been termed generalized Einstein-aether (GEA) theories, emphasizing the coexistence of general covariance and a ‘preferred’ state of rest in the model, i.e., keeping time with $A^{\mu }$.

Indeed, when the geometry is of the form (I.5.74), anisotropic stresses are negligible and $A^{\mu }$ is aligned with the flow of time $t^{\mu }$, then one can find appropriate values of the $c_{A}$ and $\ell$ such that *K* is dominated by a term equal to $|\nabla \varPsi |^{2}/a_{0}^{2}$. This influence then leads to a modification to the time-time component of Einstein’s equations: instead of reducing to Poisson’s equation, one recovers an equation of the form (I.5.73). Therefore the models are successful covariant realizations of MOND.

Interestingly, in the FLRW limit $\varPhi ,\varPsi \rightarrow 0$, the time-time component of Einstein’s equations in the GEA model becomes a modified Friedmann equation:

I.5.77 $$\begin{aligned} \beta \left( \frac{H^{2}}{a_{0}^{2}}\right) H^{2} = \frac{8\pi G\rho }{3}, \end{aligned}$$

where the function $\beta$ is related to *F* and its derivatives with respect to *K*. The dynamics in galaxies prefer a value $a_{0}$ on the order the Hubble parameter today $H_{0}$ (Sanders and McGaugh 2002) and so one typically gets a modification to the background expansion with a characteristic scale $H_{0}$, i.e., the scale associated with modified gravity models that produce dark-energy effects. Ultimately the GEA model is a phenomenological one and as such there currently lack deeper reasons to favor any particular form of *F*. However, one may gain insight into the possible solutions of (I.5.77) by looking at simple forms for *F*. In Zuntz et al. (2010), the monomial case $F\propto K^{n_{ae}}$ was considered where the kinetic index $n_{ae}$ was allowed to vary. Solutions with accelerated expansion were found that could mimic dark energy.

Returning to the original motivation behind the theory, the next step is to look at the theory on cosmological scales and see whether the GEA models are realistic alternatives to dark matter. As emphasized, the additional structure in spacetime is dynamical and so possesses independent degrees of freedom. As the model is assumed to be uncoupled to other matter, the gravitational field equations would regard the influence of these degrees of freedom as a type of dark matter (possibly coupled non-minimally to gravity, and not necessarily ‘cold’).

The possibility that the model may then be a viable alternative to the dark sector in background cosmology and linear cosmological perturbations has been explored in depth in Zlosnik et al. (2008), Li et al. (2008a) and Zuntz et al. (2010). As an alternative to dark matter, it was found that the GEA models could replicate some but not all of the following features of cold dark matter: influence on background dynamics of the universe; negligible sound speed of perturbations; growth rate of dark matter ‘overdensity’; absence of anisotropic stress and contribution to the cosmological Poisson equation; effective minimal coupling to the gravitational field. When compared to the data from large scale structure and the CMB, the model fared significantly less well than the concordance model and so is excluded. If one relaxes the requirement that the vector field be responsible for the effects of cosmological dark matter, one can look at the model as one responsible only for the effects of dark energy. It was found (Zuntz et al. 2010) that the current most stringent constraints on the model’s success as dark energy were from constraints on the size of large scale CMB anisotropy. Specifically, possible variation in *w*(*z*) of the ‘dark energy’ along with new degrees of freedom sourcing anisotropic stress in the perturbations was found to lead to new, non-standard time variation of the potentials $\varPhi$ and $\varPsi$. These time variations source large scale anisotropies via the integrated Sachs–Wolfe effect, and the parameter space of the model is constrained in avoiding the effect becoming too pronounced.

In spite of this, given the status of current experimental bounds it is conceivable that a more successful alternative to the dark sector may share some of these points of departure from the Concordance Model and yet fare significantly better at the level of the background and linear perturbations.

#### The tensor–vector–scalar theory of gravity

Another proposal for a theory of modified gravity arising from Milgrom’s observation is the tensor–vector–scalar theory of gravity, or TeVeS. TeVeS theory is *bimetric* with two frames: the “geometric frame” for the gravitational fields, and the “physical frame”, for the matter fields. The three gravitational fields are the metric $\tilde{g}_{ab}$ (with connection $\tilde{\nabla }_a$) that we refer to as the geometric metric, the vector field $A_a$ and the scalar field $\phi$. The action for all matter fields, uses a single physical metric $g_{ab}$ (with connection $\nabla _a$). The two metrics are related via an algebraic, disformal relation (Bekenstein 1993) as

I.5.78 $$\begin{aligned} g_{ab} = e^{-2\phi }\tilde{g}_{ab} - 2\sinh (2\phi )A_a A_b. \end{aligned}$$

Just like in the generalized Einstein-Aether theories, the vector field is further enforced to be unit-timelike with respect to the geometric metric, i.e.,

I.5.79 $$\begin{aligned} \tilde{g}^{ab} A_a A_b = A^a A_a = -\,1. \end{aligned}$$

The theory is based on an action *S*, which is split as $S = S_{\tilde{g}} + S_A + S_{\phi }+S_m$ where

I.5.80 $$\begin{aligned} S_{\tilde{g}} = \frac{1}{16\pi G}\int \mathrm {d}^4x \; \sqrt{-\tilde{g}}\; \tilde{R}, \end{aligned}$$

where $\tilde{g}$ and $\tilde{R}$ are the determinant and scalar curvature of $\tilde{g}_{\mu \nu }$ respectively and *G* is the bare gravitational constant,

I.5.81 $$\begin{aligned} S_A = -\frac{1}{32\pi G} \int \mathrm {d}^4x \; \sqrt{-\tilde{g}}\; \left[ K F^{ab}F_{ab} - 2\lambda \left( A_a A^a + 1\right) \right] , \end{aligned}$$

where $F_{ab} = \nabla _a A_b - \nabla _b A_a$ leads to a Maxwellian kinetic term and $\lambda$ is a Lagrange multiplier ensuring the unit-timelike constraint on $A_a$ and *K* is a dimensionless constant (note that indices on $F_{ab}$ are raised using the geometric metric, i.e., $F^a_{\;\;b} = \tilde{g}^{ac} F_{cb}$) and

I.5.82 $$\begin{aligned} S_{\phi } = -\frac{1}{16\pi G} \int \mathrm {d}^4x \sqrt{-\tilde{g}}\left[ \mu \; \hat{g}^{ab}\tilde{\nabla }_a\phi \tilde{\nabla }_b\phi + V(\mu ) \right] , \end{aligned}$$

where $\mu$ is a non-dynamical dimensionless scalar field, $\hat{g}^{ab} = \tilde{g}^{ab} - A^a A^b$ and $V(\mu )$ is an arbitrary function that typically depends on a scale $\ell _B$. The matter is coupled only to the physical metric $g_{ab}$ and defines the matter stress–energy tensor $T_{ab}$ through $\delta S_m = -\frac{1}{2} \int \mathrm {d}^4x \sqrt{-g}\; T_{ab} \; \delta g^{ab}$. The TeVeS action can be written entirely in the physical frame (Zlosnik et al. 2006; Skordis 2009) or in a diagonal frame (Skordis 2009) where the scalar and vector fields decouple.

In a Friedmann universe, the cosmological evolution is governed by the Friedmann equation

I.5.83 $$\begin{aligned} 3\tilde{H}^2 = 8\pi G e^{-2\phi } \left( \rho _\phi + \rho \right) , \end{aligned}$$

where $\tilde{H}$ is the Hubble rate in terms of the geometric scale factor, $\rho$ is the physical matter density that obeys the energy conservation equation with respect to the physical metric and where the scalar field energy density is

I.5.84 $$\begin{aligned} \rho _\phi = \frac{e^{2\phi }}{16\pi G}\left( \mu \frac{\mathrm {d}V}{\mathrm {d}\mu }+ V \right) \end{aligned}$$

Exact analytical and numerical solutions with the Bekenstein free function have been found in Skordis et al. (2006) and Dodelson and Liguori (2006). It turns out that energy density tracks the matter fluid energy density. The ratio of the energy density of the scalar field to that of ordinary matter is approximately constant, so that the scalar field exactly tracks the matter dynamics. In realistic situations, the radiation era tracker is almost never realized, as has been noted by Dodelson and Liguori, but rather $\rho _\phi$ is subdominant and slowly-rolling and $\phi \propto a^{4/5}$. Bourliot et al. (2007) studied more general free functions which have the Bekenstein function as a special case and found a whole range of behavior, from tracking and accelerated expansion to finite time singularities. Diaz-Rivera et al. (2006) have studied cases where the cosmological TeVeS equations lead to inflationary/accelerated expansion solutions.

Although no further studies of accelerated expansion in TeVeS have been performed, it is very plausible that certain choices of function will inevitably lead to acceleration. It is easy to see that the scalar field action has the same form as a k-essence/k-inflation (Armendariz-Picon et al. 2000) action which has been considered as a candidate theory for acceleration. It is unknown in general whether this has similar features as the uncoupled k-essence, although Zhao’s study indicates that this a promising research direction (Zhao 2008).

In TeVeS, cold dark matter is absent. Therefore, in order to get acceptable values for the physical Hubble constant today (i.e., around $H_0 \sim 70\mathrm {\ km/s/Mpc}$), we have to supplement the absence of CDM with something else. Possibilities include the scalar field itself, massive neutrinos (Skordis et al. 2006; Ferreira et al. 2008) and a cosmological constant. At the same time, one has to get the right angular diameter distance to recombination (Ferreira et al. 2008). These two requirements can place severe constraints on the allowed free functions.

**Fig. 4:**
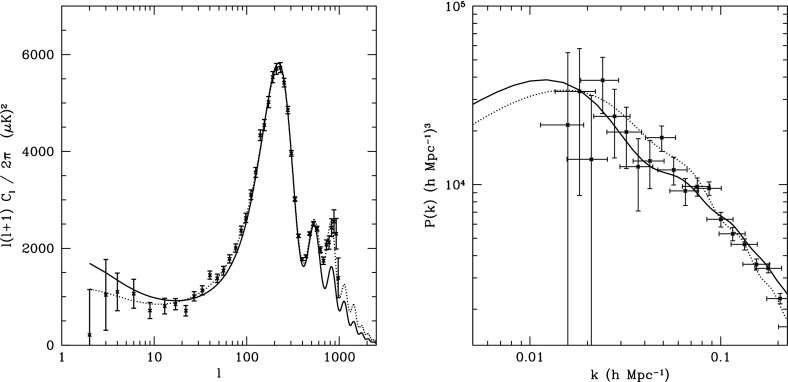
Left: the cosmic microwave background angular power spectrum $l(l+1)C_l/(2\pi )$ for TeVeS (solid) and $\varLambda$CDM (dotted) with WMAP 5-year data (Nolta et al. 2009). Right: the matter power spectrum *P*(*k*) for TeVeS (solid) and $\varLambda$CDM (dotted) plotted with SDSS data

Until TeVeS was proposed and studied in detail, MOND-type theories were assumed to be fatally flawed: their lack of a dark matter component would necessarily prevent the formation of large-scale structure compatible with current observational data. In the case of an Einstein universe, it is well known that, since baryons are coupled to photons before recombination they do not have enough time to grow into structures on their own. In particular, on scales smaller than the diffusion damping scale perturbations in such a universe are exponentially damped due to the Silk-damping effect. CDM solves all of these problems because it does not couple to photons and therefore can start creating potential wells early on, into which the baryons fall.

TeVeS contains two additional fields, which change the structure of the equations significantly. The first study of TeVeS predictions for large-scale structure observations was conducted in Skordis et al. (2006). They found that TeVeS can indeed form large-scale structure compatible with observations depending on the choice of TeVeS parameters in the free function. In fact the form of the matter power spectrum *P*(*k*) in TeVeS looks quite similar to that in $\varLambda$CDM. Thus TeVeS can produce matter power spectra that cannot be distinguished from $\varLambda$CDM by current observations. One would have to turn to other observables to distinguish the two models. The power spectra for TeVeS and $\varLambda$CDM are plotted on the right panel of Fig. 4. Dodelson and Liguori (2006) provided an analytical explanation of the growth of structure seen numerically by Skordis et al. (2006) and found that the growth in TeVeS is due to the vector field perturbation.

It is premature to claim (as in Slosar et al. 2005; Spergel et al. 2007) that only a theory with CDM can fit CMB observations; a prime example to the contrary is the EBI theory (Bañados et al. 2009). Nevertheless, in the case of TeVeS (Skordis et al. 2006) numerically solved the linear Boltzmann equation in the case of TeVeS and calculated the CMB angular power spectrum for TeVeS. By using initial conditions close to adiabatic the spectrum thus found provides very poor fit as compared to the $\varLambda$CDM model (see the left panel of Fig. 4). The CMB seems to put TeVeS into trouble, at least for the Bekenstein free function. The result of Dodelson and Liguori (2006) has a further direct consequence. The difference $\varPhi -\varPsi$, sometimes named the gravitational slip (see Sect. I.3.2), has additional contributions coming from the perturbed vector field $\alpha$. Since the vector field is required to grow in order to drive structure formation, it will inevitably lead to a growing $\varPhi - \varPsi$. The difference $\varPhi - \varPsi$ will be measured by Euclid, and therefore, when the data is available, one will be able to provide a substantial test that can distinguish TeVeS from $\varLambda$CDM.

#### Other models of interest

*I.5.11.1 Models of varying alpha*

Whenever a dynamical scalar field is added to a theory, the field will naturally couple to all other fields present, unless a (still unknown) symmetry is postulated to suppress such couplings (Carroll 1998). A coupling to the electromagnetic sector leads to spacetime variations of Nature’s fundamental constants, which are constrained both by local atomic clock experiments and by astrophysical observations (Uzan 2011). Joint constraints on dynamical dark energy model parametrizations and on the coupling with electromagnetism were obtained in Calabrese et al. (2014), combining weak lensing and supernova measurements from Euclid with high-resolution spectroscopy measurements from the European extremely large telescope (Martins 2015). These forecasts suggest that in the CPL parametrization of these models, the addition of spectroscopic data (which spans the range $0<z \lesssim 4$) improves constraints from Euclid observables by a factor of 2 for $w_0$ and by one order of magnitude for $w_a$.

*I.5.11.2* *f*(*T*) *gravity*

*f*(*T*) gravity is a generalization of teleparallel gravity, where the torsion scalar *T*, instead of curvature, is responsible for gravitational interactions. In this theory, spacetime is endowed with a curvature-free Weitzenbock connection. Thus, torsion acts as a force, allowing for the interpretation of gravity as a gauge theory of the translation group (Arcos and Pereira 2004). Teleparallel gravity and GR yield completely equivalent dynamics for $f(T)=T$, but differ for any other choice *f*(*T*) (Ferraro and Fiorini 2008; Fiorini and Ferraro 2009). Unlike analogous approach of *f*(*R*) theories, *f*(*T*) gravity yields equations that remain at second order in field derivatives; however, local Lorentz invariance is lost.

In *f*(*T*) cosmology, structure formation is modified because of a time dependent effective gravitational constant. Cardone et al. (2012) analysed two viable *f*(*T*) gravity models and showed that both are in very good agreement with a wide set of data, including SNIa and GRB Hubble diagrams, BAOs at different redshifts, Hubble expansion rate measurements and the WMAP7 distance priors. Yet, that wide dataset is unable to constrain the model parameters enough to discriminate among the considered *f*(*T*) models and the standard $\varLambda$CDM scenario. Therefore, Camera et al. (2014) investigated the imprints of *f*(*T*) gravity on galaxy clustering and weak gravitational lensing in the context of *Euclid*.

In particular, by studying weak lensing tomographic cosmic shear and both 2D and 3D clustering of *Euclid* H-$\alpha$ galaxies. They found that with such a combination of probes it will indeed be possible to tightly constrain *f*(*T*) model parameters. Again, it is the combination of clustering and lensing that yields the tighest constraints, thanks to the high complementarity of the two probes when it comes to tracking the different behaviour of the metric potentials. By such probe combination, bounds on the two modified *f*(*T*) models get more constraining by more than an order of magnitude, thus allowing us to rule out the models with more than 3$\sigma$ confidence.

### Nonlinear aspects

In this section, we discuss how the nonlinear evolution of cosmic structures in the context of different non-standard cosmological models can be studied by means of numerical simulations based on *N*-body algorithms and of analytical approaches based on the spherical collapse model.

#### *N*-body simulations of dark energy and modified gravity

Here we discuss the numerical methods presently available for this type of analyses, and we review the main results obtained so far for different classes of alternative cosmologies. These can be grouped into models where structure formation is affected only through a modified expansion history (such as quintessence and early dark-energy models, Sect. I.5.1) and models where particles experience modified gravitational forces, either for individual particle species (interacting dark-energy models and growing neutrino models, Sect. I.5.3 or for all types of particles in the universe (modified gravity models). A general overview on the recent developments in the field of dark energy and modified gravity *N*-body simulations can be found in Baldi (2012a).

*I.6.1.1 Quintessence and early dark-energy models*

In general, in the context of flat FLRW cosmologies, any dynamical evolution of the dark-energy density ($\rho _{\mathrm {DE}}\ne {\mathrm {const.}} = \rho _{\varLambda }$) determines a modification of the cosmic expansion history with respect to the standard $\varLambda$CDM cosmology. In other words, if the dark energy is a dynamical quantity, i.e., if its equation of state parameter $w\ne -1$ exactly, for any given set of cosmological parameters ($H_{0}$, $\varOmega _{\mathrm {CDM}}$, $\varOmega _{\mathrm {b}}$, $\varOmega _{\mathrm {DE}}$, $\varOmega _{\mathrm {rad}}$), the redshift evolution of the Hubble function *H*(*z*) will differ from the standard $\varLambda$CDM case $H_{\varLambda }(z)$.

Quintessence models of dark energy (Wetterich 1988; Ratra and Peebles 1988) based on a classical scalar field $\phi$ subject to a self-interaction potential $V(\phi )$ have an energy density $\rho _{\phi } \equiv \dot{\phi }^{2}/2 + V(\phi )$ that evolves in time according to the dynamical evolution of the scalar field, which is governed by the homogeneous Klein–Gordon equation:

I.6.1 $$\begin{aligned} \ddot{\phi } + 3H\dot{\phi } + \frac{\mathrm {d}V}{\mathrm {d}\phi } = 0. \end{aligned}$$

Here the dot denotes derivation w.r.t. ordinary time *t*.

For a canonical scalar field, the equation of state parameter $w_{\phi }\equiv \rho _{\phi }/p_{\phi }$, where $p_{\phi }\equiv \dot{\phi }^{2}/2 - V(\phi )$, will in general be larger than $-\,1$, and the density of dark energy $\rho _{\phi }$ will consequently be larger than $\rho _{\varLambda }$ at any redshift $z > 0$. Furthermore, for some simple choices of the potential function such as those discussed in Sect. I.5.1 (e.g., an exponential potential $V\propto \exp (-\alpha \phi /M_{\mathrm {Pl}})$ or an inverse-power potential $V\propto (\phi /M_{\mathrm {Pl}})^{-\alpha }$), scaling solutions for the evolution of the system can be analytically derived. In particular, for an exponential potential, a scaling solution exists where the dark energy scales as the dominant cosmic component, with a fractional energy density

I.6.2 $$\begin{aligned} \varOmega _{\phi }\equiv \frac{8\pi G \rho _{\phi }}{3H^{2}} = \frac{n}{\alpha ^{2}}, \end{aligned}$$

with $n=3$ for matter domination and $n=4$ for radiation domination. This corresponds to a relative fraction of dark energy at high redshifts, which is in general not negligible, whereas during matter and radiation domination $\varOmega _{\varLambda }\sim 0$ and, therefore, represents a phenomenon of an early emergence of dark energy as compared to $\varLambda$CDM where dark energy is for all purposes negligible until $z\sim 1$.

Early dark energy (EDE) is, therefore, a common prediction of scalar field models of dark energy, and observational constraints put firm bounds on the allowed range of $\varOmega _{\mathrm {DE}}$ at early times, and consequently on the potential slope $\alpha$.

As we have seen in Sect. I.2.1, a completely phenomenological parametrization of EDE, independent from any specific model of dynamical dark energy has been proposed by Wetterich (2004) as a function of the present dark-energy density $\varOmega _{\mathrm {DE}}$, its value at early times $\varOmega _{\mathrm {e}}$, and the present value of the equation of state parameter $w_{0}$. From Eq. I.2.4, the full expansion history of the corresponding EDE model can be derived.

A modification of the expansion history indirectly influences also the growth of density perturbations and ultimately the formation of cosmic structures. While this effect can be investigated analytically for the linear regime, *N*-body simulations are required to extend the analysis to the nonlinear stages of structure formation. For standard Quintessence and EDE models, the only modification that is necessary to implement into standard *N*-body algorithms is the computation of the correct Hubble function *H*(*z*) for the specific model under investigation, since this is the only way in which these non standard cosmological models can alter structure formation processes.

This has been done by the independent studies of Grossi and Springel (2009) and Francis et al. (2008a), where a modified expansion history consistent with EDE models described by the parametrization of Eq. I.2.4 has been implemented in the widely used *N*-body code Gadget-2 (Springel 2005) and the properties of nonlinear structures forming in these EDE cosmologies have been analyzed. Both studies have shown that the standard formalism for the computation of the halo mass function still holds for EDE models at $z=0$. In other words, both the standard fitting formulae for the number density of collapsed objects as a function of mass, and their key parameter $\delta _{c} = 1.686$ representing the linear overdensity at collapse for a spherical density perturbation, remain unchanged also for EDE cosmologies.

The work of Grossi and Springel (2009), however, investigated also the internal properties of collapsed halos in EDE models, finding a slight increase of halo concentrations due to the earlier onset of structure formation and most importantly a significant increment of the line-of-sight velocity dispersion of massive halos. The latter effect could mimic a higher $\sigma _{8}$ normalization for cluster mass estimates based on galaxy velocity dispersion measurements and, therefore, represents a potentially detectable signature of EDE models.

Besides determining a different expansion history with respect to the standard $\varLambda$CDM cosmology due to the presence of an EDE component, scalar-field DE cosmologies also predict the existence of spatial perturbations of the DE density, resulting in a modification of the shape of the matter power spectrum. Even though such density perturbations are suppressed by free-streaming at sub-horizon scales (thereby allowing to discard the effect of DE fluctuations on the dynamical evolution of cosmic structures), they remain frozen to a constant value at super-horizon scales. Therefore, as new large scales continuously enter the causal horizon, they will be affected by the presence of DE perturbations before these are eventually damped by free-streaming. Consequently, DE perturbations are expected to slightly change the large-scale shape of the linear power spectrum, thereby affecting the initial conditions of structure formation (Ma et al. 1999; Alimi et al. 2010). This has motivated the development of DE *N*-body simulations with extremely large volumes, comparable or larger to the comoving size of the cosmic horizon, in order to investigate the nonlinear signatures of the large-scale DE perturbations (Alimi et al. 2010, 2012; Rasera et al. 2010). Such studies have highlighted that the nonlinear regime of structure formation carries information on the initial conditions of the Universe and keeps memory of the growth history of density perturbations even for the case of perfectly degenerate linear matter power spectra and $\sigma _{8}$ values. Therefore, nonlinear structure formation processes represent a precious source of information for the highly demanding requirements of precision cosmology.

*I.6.1.2 Interacting dark-energy models*

Another interesting class of non standard dark-energy models, as introduced in Sect. I.5.3, is given by coupled dark energy where a direct interaction is present between a Quintessence scalar field $\phi$ and other cosmic components, in the form of a source term in the background continuity equations:

I.6.3 $$\begin{aligned} \frac{\mathrm {d}\rho _{\phi }}{\mathrm {d}\eta }= & {} -3 {{\mathcal {H}}} (1 + w_\phi ) \rho _{\phi } + \beta (\phi ) \frac{\mathrm {d}\phi }{\mathrm {d}\eta } (1-3 w_{\alpha }) \rho _{\alpha }, \end{aligned}$$

I.6.4 $$\begin{aligned} \frac{\mathrm {d}\rho _{\alpha }}{\mathrm {d}\eta }= & {} -3 {{\mathcal {H}}} (1 + w_{\alpha }) \rho _{\alpha } - \beta (\phi ) \frac{\mathrm {d}\phi }{\mathrm {d}\eta } (1-3 w_{\alpha }) \rho _{\alpha }, \end{aligned}$$

where $\alpha$ represents a single cosmic fluid coupled to $\phi$.

While such direct interaction with baryonic particles ($\alpha =b$) is tightly constrained by observational bounds, and while it is suppressed for relativistic particles ($\alpha =r$) by symmetry reasons ($1-3w_{r}=0$), a selective interaction with cold dark matter (CDM hereafter) or with massive neutrinos is still observationally viable (see Sect. I.5.3).

Since the details of interacting dark-energy models have been discussed in Sect. I.5.3, here we simply recall the main features of these models that have a direct relevance for nonlinear structure formation studies. For the case of interacting dark energy, in fact, the situation is much more complicated than for the simple EDE scenario discussed above. The mass of a coupled particle changes in time due to the energy exchange with the dark-energy scalar field $\phi$ according to the equation:

I.6.5 $$\begin{aligned} m(\phi ) = m_{0}e^{-\int \beta \left( \phi '\right) \, \mathrm {d}\phi '} \end{aligned}$$

where $m_{0}$ is the mass at $z=0$. Furthermore, the Newtonian acceleration of a coupled particle (subscript *c*) gets modified as:

I.6.6 $$\begin{aligned} \dot{\mathbf {v}}_{c} = -\tilde{H}\mathbf {v}_{c} - \varvec{\nabla }\tilde{\varPhi }_{c} - \varvec{\nabla }\varPhi _{nc}. \end{aligned}$$

where $\tilde{H}$ contains a new velocity-dependent acceleration:

I.6.7 $$\begin{aligned} \tilde{H}\mathbf {v}_{c} = H\left( 1-\beta _{\phi }\frac{\dot{\phi }}{H}\right) \mathbf {v}_{c}, \end{aligned}$$

and where a fifth-force acts only between coupled particles as

I.6.8 $$\begin{aligned} \tilde{\varPhi }_{c} = (1 + 2\beta ^{2})\varPhi _{c}, \end{aligned}$$

while $\varPhi _{nc}$ represents the gravitational potential due to all massive particles with no coupling to the dark energy that exert a standard gravitational pull.

As a consequence of these new terms in the Newtonian acceleration equation the growth of density perturbations will be affected, in interacting dark-energy models, not only by the different Hubble expansion due to the dynamical nature of dark energy, but also by a direct modification of the effective gravitational interactions at subhorizon scales. Therefore, linear perturbations of coupled species will grow with a higher rate in these cosmologies In particular, for the case of a coupling to CDM, a different amplitude of the matter power spectrum will be reached at $z=0$ with respect to $\varLambda$CDM if a normalization in accordance with CMB measurements at high redshifts is assumed.

Clearly, the new acceleration Eq. (I.6.6) will have an influence also on the formation and evolution of nonlinear structures, and a consistent implementation of all the above mentioned effects into an *N*-body algorithm is required in order to investigate this regime.

For the case of a coupling to CDM (a coupling with neutrinos will be discussed in the next section) this has been done, e.g., by Macciò et al. (2004) and Sutter and Ricker (2008) with 1D or 3D grid-based field solvers, and more recently by means of suitable modifications (by Baldi et al. 2010; Carlesi et al. 2014a) of the TreePM hydrodynamic *N*-body code Gadget-2 (Springel 2005), and similarly through a modified version (by Li and Barrow 2011a) of the adaptive mesh refinements code Ramses (Teyssier 2002).

Nonlinear evolution within coupled quintessence cosmologies has been addressed using various methods of investigation, such as spherical collapse (Mainini and Bonometto 2006; Wintergerst and Pettorino 2010; Manera and Mota 2006; Koivisto 2005; Sutter and Ricker 2008; Abdalla et al. 2009; Bertolami et al. 2009) and alternative semi-analytic methods (Saracco et al. 2010; Amendola and Quercellini 2004). *N*-body and hydro-simulations have also been done (Macciò et al. 2004; Baldi et al. 2010; Baldi 2011b; Baldi and Pettorino 2011; Baldi and Viel 2010; Li et al. 2011; Baldi 2011a; Li and Barrow 2011b; Zhao et al. 2010). We list here briefly the main observable features typical of this class of models:

- The suppression of power at small scales in the power spectrum of interacting dark-energy models as compared to $\varLambda$CDM (see, e.g., Baldi 2011a);
- An enhanced lensing power spectrum as compared to $\varLambda$CDM (see e.g. Beynon et al. 2012);
- The development of a gravitational bias in the amplitude of density perturbations of uncoupled baryons and coupled CDM particles defined as $P_{b}(k)/P_{c}(k)<1$, which determines a significant decrease of the baryonic content of massive halos at low redshifts in accordance with a large number of observations (Baldi et al. 2010; Baldi 2011a);
- The increase of the number density of high-mass objects at any redshift as compared to $\varLambda$CDM (see, e.g., Baldi and Pettorino 2011; Baldi 2012b; Cui et al. 2012);
- An enhanced ISW effect (Amendola 2000a, 2004; Mainini and Mota 2012); such effects may be partially reduced when taking into account nonlinearities, as described in Pettorino et al. (2010);
- A modification in the shape of *z*-space distortions (Marulli et al. 2012) and an enhanced pairwise infall velocity of colliding massive clusters (Lee and Baldi 2012);
- A less steep inner core halo profiles (depending on the interplay between fifth force and velocity-dependent terms) (Baldi et al. 2010; Baldi 2011a, b; Li et al. 2011; Li and Barrow 2011b);
- A lower concentration of the halos (Baldi et al. 2010; Baldi 2011b; Li and Barrow 2011b);
- CDM voids are larger and more underdense when a coupling is active (Baldi and Viel 2010; Sutter et al. 2015).
- A modified amplitude and time evolution of the halo bias (see, e.g., Marulli et al. 2012; Moresco et al. 2014), which might determine a counterintuitive behaviour in the connection between CDM and halo populations statistics in the context of interacting dark energy cosmologies.

The analysis has been extended to the case of non-constant coupling functions $\beta (\phi )$ by Baldi (2011b). As discussed in Sect. I.6.1, when a variable coupling $\beta (\phi )$ is active the relative balance of the fifth-force and other dynamical effects depends on the specific time evolution of the coupling strength. Under such conditions, some of the above mentioned results no longer hold. In particular, the CoDECS simulations series (Baldi 2012d) has provided evidence that for various combinations of coupling and self-interaction potential functions—$V(\phi )$ and $\beta {\phi }$—some of the following effects might arise:

- Small scale power can be both suppressed and enhanced when a growing coupling function is considered, depending on the magnitude of the coupling time derivative $\mathrm {d}\beta (\phi )/\mathrm {d}\phi$
- The inner overdensity of CDM halos, and consequently the halo concentrations, can both decrease (as always happens for the case of constant couplings) or increase, again depending on the rate of change of the coupling strength (Cui et al. 2012);
- The abundance of halo substructures (see Giocoli et al. 2013) as well as the CMB lensing power spectrum (see Carbone et al. 2013) might show both an enhancement or a suppression with respect to $\varLambda$CDM depending on the specific model under exam.

More recently, Carlesi et al. (2014a, b) have employed a specific suite of high-resolution *N*-body simulations to investigate how some of the above mentioned effects depend on the cosmic environment in which CDM halos reside, showing that most of the characteristic observational features of interacting dark energy are significantly enhanced for halo populations residing in underdense regions of the Universe. Furthermore, Carlesi et al. (2014a) also highlighted a positive correlation between the average spin parameter of CDM halos and the coupling constant.

All these effects represent characteristic features of interacting dark-energy models and could provide a direct way to observationally test these scenarios.

A slightly more complex realisation of the interacting dark energy scenario has been recently proposed by Baldi (2012c), and termed the “Multi-coupled dark energy” model. The latter is characterised by two distinct CDM particle species featuring an opposite constant coupling to a single classical dark energy scalar field, and represents the simplest possible realisation of the general multiple-interaction scenario proposed by Gubser and Peebles (2004a, b) and Brookfield et al. (2008). The most noticeable feature of such model is the dynamical screening that effectively suppresses the coupling at the level of the background and linear perturbations evolution, although leaving room for a possible interesting phenomenology at nonlinear scales (see, e.g., Piloyan et al. 2013, 2014). Some first *N*-body simulations of the multi-coupled dark energy scenario have been performed by Baldi (2013, 2014), showing for the first time the halo fragmentation process occurring in these cosmologies as a consequence of the repulsive long-range fifth-force between CDM particles of different types. Higher resolution simulations will be required in order to investigate possible observable effects of this new phenomenon on the shape and abundance of CDM halos at very small scales.

Alternatively, the coupling can be introduced by choosing directly a covariant stress–energy tensor, treating dark energy as a fluid in the absence of a starting action (Mangano et al. 2003; Väliviita et al. 2008, 2010; Caldera-Cabral et al. 2009b; Schaefer et al. 2008; Majerotto et al. 2010; Gavela et al. 2009, 2010; Caldera-Cabral et al. 2009a).

*I.6.1.3 Growing neutrinos*

In case of a coupling between the dark-energy scalar field $\phi$ and the relic fraction of massive neutrinos, all the above basic Eqs. (I.6.5)–(I.6.8) still hold. However, such models are found to be cosmologically viable only for large negative values of the coupling $\beta$ (as shown by Amendola et al. 2008a), that according to Eq. I.6.5 determines a neutrino mass that grows in time (from which these models have been dubbed “growing neutrinos”). An exponential growth of the neutrino mass implies that cosmological bounds on the neutrino mass are no longer applicable and that neutrinos remain relativistic much longer than in the standard scenario, which keeps them effectively uncoupled until recent epochs, according to Eqs. (I.6.3 and I.6.4). However, as soon as neutrinos become non-relativistic at redshift $z_{\mathrm {nr}}$ due to the exponential growth of their mass, the pressure terms $1-3w_{\nu }$ in Eqs. (I.6.3 and I.6.4) no longer vanish and the coupling with the DE scalar field $\phi$ becomes active.

Therefore, while before $z_{\mathrm {nr}}$ the model behaves as a standard $\varLambda$CDM scenario, after $z_{\mathrm {nr}}$ the non-relativistic massive neutrinos obey the modified Newtonian Eq. (I.6.6) and a fast growth of neutrino density perturbation takes place due to the strong fifth force described by Eq. (I.6.8).

The growth of neutrino overdensities in the context of growing neutrinos models has been studied in the linear regime by Mota et al. (2008), predicting the formation of very large neutrino lumps at the scale of superclusters and above (10–100 Mpc/h) at redshift $z\approx 1$.

The analysis has been extended to the nonlinear regime in Wintergerst et al. (2010) by following the spherical collapse of a neutrino lump in the context of growing neutrino cosmologies. This study has witnessed the onset of virialization processes in the nonlinear evolution of the neutrino halo at $z\approx 1.3$, and provided a first estimate of the associated gravitational potential at virialization being of the order of $\varPhi _{\nu }\approx 10^{-6}$ for a neutrino lump with radius $R \approx 15\mathrm {\ Mpc}$.

An estimate of the potential impact of such very large nonlinear structures onto the CMB angular power spectrum through the integrated Sachs–Wolfe effect has been attempted by Pettorino et al. (2010). This study has shown that the linear approximation fails in predicting the global impact of the model on CMB anisotropies at low multipoles, and that the effects under consideration are very sensitive to the details of the transition between the linear and nonlinear regimes and of the virialization processes of nonlinear neutrino lumps, and that also significantly depend on possible backreaction effects of the evolved neutrino density field onto the local scalar filed evolution.

A full nonlinear treatment by means of specifically designed *N*-body simulations is, therefore, required in order to follow in further detail the evolution of a cosmological sample of neutrino lumps beyond virialization, and to assess the impact of growing neutrinos models onto potentially observable quantities as the low-multipoles CMB power spectrum or the statistical properties of CDM large scale structures. Simulations of the growing neutrino scenario have been performed for the first time by Baldi et al. (2011) by means of a modified version of the Gadget-2 code which assumed the linearity of the scalar field spatial perturbations (and consequently of the neutrino mass) and no backreaction of the growth of neutrino lumps on the overall background cosmic expansions. Although such approximations are quite restrictive, the simulations performed by Baldi et al. (2011) allowed to follow the evolution of the formation of a few large neutrino structures down to $z\sim 1$, after which neutrino particles start to become relativistic thereby breaking the Newtonian implementation of gravitational dynamics implemented in standard *N*-body algorithms. Such restrictions and approximations have been subsequently removed by the specific *N*-body algorithm developed by Ayaita et al. (2012) which self-consistently implements both the relativistic evolution of neutrino particles and the backreaction effect on the background cosmology, and which employs a Newton–Gauss–Seidel relaxation scheme to solve for nonlinear spatial fluctuations of the scalar field. Nonetheless, even this more accurate numerical treatment has been so far successfully employed only down to $z\sim 1$ due to its high computational cost.

*I.6.1.4 Modified gravity*

Modified gravity models, presented in Sect. I.5, represent a different perspective to account for the nature of the dark components of the universe. Although most of the viable modifications of GR are constructed in order to provide an identical cosmic expansion history to the standard $\varLambda$CDM model, their effects on the growth of density perturbations could lead to observationally testable predictions capable of distinguishing modified gravity models from standard GR plus a cosmological constant.

Since a modification of the theory of gravity would affect all test masses in the universe, i.e., including the standard baryonic matter, an asymptotic recovery of GR for solar system environments, where deviations from GR are tightly constrained, is required for all viable modified gravity models. Such “screening mechanism” represents the main difference between modified gravity models and the interacting dark-energy scenarios discussed above, by determining a local dependence of the modified gravitational laws in the Newtonian limit. Different modifications of the GR Action integral might feature different types of screening mechanisms (see Sect. I.5 for an introduction to modified gravity theories and screening mechanisms)—as, e.g., the “Chameleon” (Khoury and Weltman 2004), the “Symmetron” (Hinterbichler and Khoury 2010), the “Dilaton” (Damour and Polyakov 1994) or the “Vainshtein” (Vainshtein 1972) mechanisms—which in turn might require different numerical implementations in order to solve for the fully nonlinear evolution of the additional degrees of freedom associated to the modifications of gravity.

While the linear growth of density perturbations in the context of modified gravity theories can be studied (see, e.g., Hu and Sawicki 2007a; Motohashi et al. 2010b; Amarzguioui et al. 2006; Appleby and Weller 2010) by parametrizing the scale dependence of the modified Poisson and Euler equations in Fourier space (see the discussion in Sect. I.3), the nonlinear evolution of the additional degrees of freedom of any viable modified gravity scenario makes the implementation of these theories into nonlinear *N*-body algorithms much more challenging. Nonetheless, enormous progress has been made over the past few years in the development of specific *N*-body codes for various classes of modified gravity cosmologies, such that the investigation of nonlinear structure formation for (at least some) alternative gravitational theories by means of dedicated *N*-body simulations is becoming a mature field of investigation in computational cosmology. The first simulations of modified gravity cosmologies, limited to the “Chameleon” screening mechanism featured by *f*(*R*) theories, have been performed by means of mesh-based iterative relaxation schemes (Oyaizu 2008; Oyaizu et al. 2008; Schmidt et al. 2009; Khoury and Wyman 2009; Zhao et al. 2011; Davis et al. 2012a; Winther et al. 2012) and showed an enhancement of the power spectrum amplitude at intermediate and small scales. These studies also showed that this nonlinear enhancement of small scale power cannot be accurately reproduced by applying the linear perturbed equations of each specific modified gravity theory to the standard nonlinear fitting formulae (as, e.g., Smith et al. 2003).

After these first pioneering studies, a very significant amount of work has been done in both extending the numerical implementation of modified gravity models within *N*-body algorithms and in using high-resolution *N*-body simulations to investigate the impact of various modifications of gravity on possible observable quantities.

Concerning the former aspect, the main advancements have been obtained by extending the range of possible screening mechanisms implemented in the modified gravity nonlinear solvers, in order to include “Symmetron” (see, e.g., Davis et al. 2012a), “Dilaton” (see, e.g., Brax et al. 2012b), and Vainshtein-like (see, e.g., Li et al. 2013a, b; Barreira et al. 2013, for the cases of general Vainshtein as well as for cubic and quartic Galileon models) mechanisms, and by optimising the nonlinear Poisson solvers. Presently, three main parallel codes for modified gravity models have been developed by independent groups, based on different underlying *N*-body numerical schemes and procedures, namely the ECOSMOG (Li et al. 2012c), the MG-GADGET (Puchwein et al. 2013), and the ISIS (Llinares et al. 2014) codes. The latter has also recently provided the first implementation of Chameleon and Symmetron modified gravity theories beyond the quasi-static approximation (Llinares and Mota 2013, 2014).

Concerning the latter aspect, a wide range of results about the impact of various modified gravity theories on several observable quantities have been obtained with the above-mentioned *N*-body codes, generally finding a good agreement between the different algorithms even though a properly controlled code-comparison study has yet to be performed. Among the most relevant results it is worth mentioning the identification of modified gravity signatures in the large-scale structure statistics (see, e.g., Li et al. 2012b; Lombriser et al. 2013, 2014; Arnold et al. 2014), in the environmental dependence of CDM halo properties (Winther et al. 2012), on the large-scale velocity field (see, e.g., Li et al. 2012a; Jennings et al. 2012; Hellwing et al. 2014), and on the ISW effect (see, e.g., Cai et al. 2013). Furthermore, a recent study (Baldi et al. 2014) performed with the MG-GADGET code has highlighted the issue of a severe observational degeneracy between the effects of an *f*(*R*) modification of gravity and a cosmological background of massive neutrinos

Despite the huge advancements in the field of nonlinear simulations of modified gravity models achieved in recent years,

higher resolution simulations and new numerical approaches will be necessary in order to extend these results to smaller scales and to accurately evaluate the deviations of specific models of modified gravity from the standard GR predictions to a potentially detectable precision level.

#### The spherical collapse model

A popular analytical approach to study nonlinear clustering of dark matter without recurring to *N*-body simulations is the spherical collapse model, first studied by Gunn and Gott (1972). In this approach, one studies the collapse of a spherical overdensity and determines its critical overdensity for collapse as a function of redshift. Combining this information with the extended Press–Schechter theory (Press and Schechter 1974; Bond et al. 1991; see Zentner et al. 2008 for a review) one can provide a statistical model for the formation of structures which allows to predict the abundance of virialized objects as a function of their mass. Although it fails to match the details of *N*-body simulations, this simple model works surprisingly well and can give useful insigths into the physics of structure formation. Improved models accounting for the complexity of the collapse exist in the literature and offer a better fit to numerical simulations. For instance, Sheth and Tormen (1999) showed that a significant improvement can be obtained by considering an ellipsoidal collapse model. Furthermore, recent theoretical developments and new improvements in the excursion set theory have been undertaken by Maggiore and Riotto (2010) and other authors (see, e.g., Shaw and Mota 2008).

The spherical collapse model has been generalized to include a cosmological constant by Peebles (1984) and Weinberg (1987). Lahav et al. (1991) have used it to study the observational consequences of a cosmological constant on the growth of perturbations. The case of standard quintessence, with speed of sound $c_s = 1$, have been studied by Wang and Steinhardt (1998). In this case, scalar fluctuations propagate at the speed of light and sound waves maintain quintessence homogeneous on scales smaller than the horizon scale. In the spherical collapse pressure gradients maintain the same energy density of quintessence between the inner and outer part of the spherical overdensity, so that the evolution of the overdensity radius is described by

I.6.9 $$\begin{aligned} \frac{\ddot{R}}{R} = -\frac{4 \pi G}{3} \left( \rho _m + {\bar{\rho }}_Q + 3 {\bar{p}}_Q\right) , \end{aligned}$$

where $\rho _m$ denotes the energy density of dark matter while $\bar{\rho }_Q$ and ${\bar{p}}_Q$ denote the homogeneous energy density and pressure of the quintessence field. Note that, although this equation looks like one of the Friedmann equations, the dynamics of *R* is not the same as for a FLRW universe. Indeed, $\rho _m$ evolves following the scale factor *R*, while the quintessence follows the external scale factor *a*, according to the continuity equation $\dot{{\bar{\rho }}}_Q + 3 (\dot{a}/a) ({\bar{\rho }}_Q + {\bar{p}}_Q) =0$.

In the following we will discuss the spherical collapse model in the contest of other dark energy and modified gravity models.

*I.6.2.1 Clustering dark energy*

In its standard version, quintessence is described by a minimally-coupled canonical field, with speed of sound $c_s=1$. As mentioned above, in this case clustering can only take place on scales larger than the horizon, where sound waves have no time to propagate. However, observations on such large scales are strongly limited by cosmic variance and this effect is difficult to observe. A minimally-coupled scalar field with fluctuations characterized by a practically zero speed of sound can cluster on all observable scales. There are several theoretical motivations to consider this case. In the limit of zero sound speed one recovers the Ghost Condensate theory proposed by Arkani-Hamed et al. (2004b) in the context of modification of gravity, which is invariant under shift symmetry of the field $\phi \rightarrow \phi + {\mathrm {constant}}$. Thus, there is no fine tuning in assuming that the speed of sound is very small: quintessence models with vanishing speed of sound should be thought of as deformations of this particular limit where shift symmetry is recovered. Moreover, it has been shown that minimally-coupled quintessence with an equation of state $w< -\,1$ can be free from ghosts and gradient instabilities only if the speed of sound is very tiny, $| c_s | \lesssim 10^{-15}$. Stability can be guaranteed by the presence of higher derivative operators, although their effect is absent on cosmologically relevant scales (Creminelli et al. 2006, 2009; Cheung et al. 2008c).

The fact that the speed of sound of quintessence may vanish opens up new observational consequences. Indeed, the absence of quintessence pressure gradients allows instabilities to develop on all scales, also on scales where dark matter perturbations become nonlinear. Thus, we expect quintessence to modify the growth history of dark matter not only through its different background evolution but also by actively participating to the structure formation mechanism, in the linear and nonlinear regime, and by contributing to the total mass of virialized halos.

Following Creminelli et al. (2010), in the limit of zero sound speed pressure gradients are negligible and, as long as the fluid approximation is valid, quintessence follows geodesics remaining comoving with the dark matter (see also Lim et al. 2010 for a more recent model with identical phenomenology). In particular, one can study the effect of quintessence with vanishing sound speed on the structure formation in the nonlinear regime, in the context of the spherical collapse model. The zero speed of sound limit represents the natural counterpart of the opposite case $c_s = 1$. Indeed, in both cases there are no characteristic length scales associated with the quintessence clustering and the spherical collapse remains independent of the size of the object (see Basse et al. 2011; Mota and van de Bruck 2004; Nunes and Mota 2006 for a study of the spherical collapse when $c_s$ of quintessence is small but finite).

Due to the absence of pressure gradients quintessence follows dark matter in the collapse and the evolution of the overdensity radius is described by

I.6.10 $$\begin{aligned} \frac{{\ddot{R}}}{R} = -\frac{4 \pi G}{3} \left( \rho _m + \rho _Q + {\bar{p}}_Q\right) , \end{aligned}$$

**Fig. 5:**
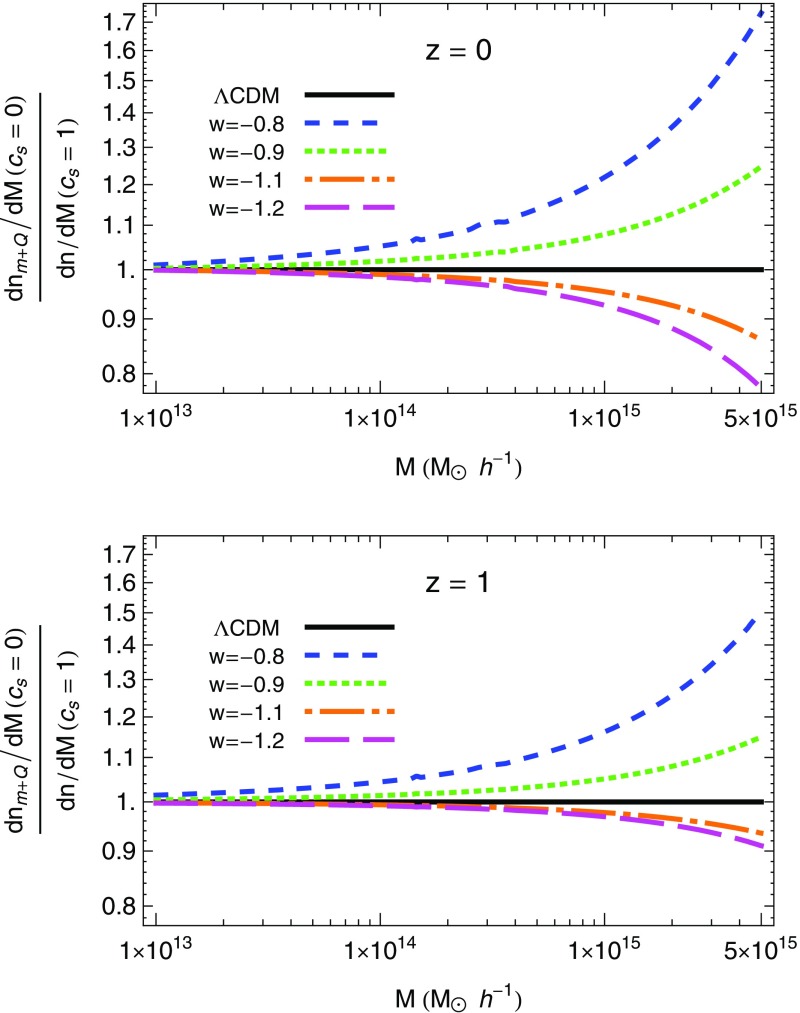
Ratio of the total mass functions, which include the quintessence contribution, for $c_s=0$ and $c_s=1$ at $z=0$ (above) and $z=1$ (below). Image reproduced by permission from Creminelli et al. (2010); copyright by IOP and SISSA

where the energy density of quintessence $\rho _Q$ has now a different value inside and outside the overdensity, while the pressure remains unperturbed. In this case the quintessence inside the overdensity evolves following the internal scale factor *R*, $\dot{\rho }_Q + 3 (\dot{R}/R) (\rho _Q + {\bar{p}}_Q) =0$ and the comoving regions behave as closed FLRW universes. *R* satisfies the Friedmann equation and the spherical collapse can be solved exactly (Creminelli et al. 2010).

Quintessence with zero speed of sound modifies dark matter clustering with respect to the smooth quintessence case through the linear growth function and the linear threshold for collapse. Indeed, for $w >-\,1$ ($w < -\,1$), it enhances (diminishes) the clustering of dark matter, the effect being proportional to $1+w$. The modifications to the critical threshold of collapse are small and the effects on the dark matter mass function are dominated by the modification on the linear dark matter growth function. Besides these conventional effects there is a more important and qualitatively new phenomenon: quintessence mass adds to the one of dark matter, contributing to the halo mass by a fraction of order $\sim (1 + w) \varOmega _Q/\varOmega _m$. Importantly, it is possible to show that the mass associated with quintessence stays constant inside the virialized object, independently of the details of virialization. Moreover,the ratio between the virialization and the turn-around radii is approximately the same as the one for $\varLambda$CDM computed by Lahav et al. (1991). In Fig. 5, we plot the ratio of the mass function including the quintessence mass contribution, for the $c_s=0$ case to the smooth $c_s=1$ case. The sum of the two effects is rather large: for values of *w* still compatible with the present data and for large masses the difference between the predictions of the $c_s = 0$ and the $c_s = 1$ cases is of order one.

*I.6.2.2 Coupled dark energy*

We now consider spherical collapse within coupled dark-energy cosmologies. The presence of an interaction that couples the cosmon dynamics to another species introduces a new force acting between particles (CDM or neutrinos in the examples mentioned in Sect. I.5.3) and mediated by dark-energy fluctuations. Whenever such a coupling is active, spherical collapse, whose concept is intrinsically based on gravitational attraction via the Friedmann equations, has to be suitably modified in order to account for other external forces. As shown in Wintergerst and Pettorino (2010), the inclusion of the fifth force within the spherical collapse picture deserves particular caution. Here we summarize the main results on this topic and we refer to Wintergerst and Pettorino (2010) for a detailed illustration of spherical collapse in presence of a fifth force.

If CDM is coupled to a quintessence scalar field as described in Sects. I.5.3 and II.10 of the present document, the full nonlinear evolution equations within the Newtonian limit read:

I.6.11 $$\begin{aligned} \dot{\delta }_m= & {} -\mathbf {v}_m\,\nabla \delta _m - \left( 1 + \delta _m\right) \,\nabla \cdot \mathbf {v}_m \end{aligned}$$

I.6.12 $$\begin{aligned} \dot{\mathbf {v}}_m= & {} -\left( 2{{\bar{H}}} - \beta \,\dot{{\bar{\phi }}}\right) \,\mathbf {v}_m - \left( \mathbf {v}_m\,\nabla \right) \mathbf {v}_m \nonumber \\&- a^{-2}\,\nabla \left( \varPhi - \beta \,\delta \phi \right) \end{aligned}$$

I.6.13 $$\begin{aligned} \varDelta \delta \phi= & {} -\beta \,a^2\,\delta \rho _m \end{aligned}$$

I.6.14 $$\begin{aligned} \varDelta \varPhi= & {} -\frac{a^2}{2}\,\sum _{\alpha } \delta \rho _{\alpha } \end{aligned}$$

These equations can be derived from the non-relativistic Navier–Stokes equations and from the Bianchi identities written in presence of an external source of the type:

I.6.15 $$\begin{aligned} \nabla _{\gamma }T_{\mu }^{\gamma } = Q_{\mu } = -\beta T_{\gamma }^{\gamma } \partial _{\mu }\phi , \end{aligned}$$

where $T^{\gamma }_{\mu }$ is the stress energy tensor of the dark matter fluid and we are using comoving spatial coordinates $\mathbf {x}$ and cosmic time *t*. Note that $\mathbf {v}_m$ is the comoving velocity, related to the peculiar velocities by $\mathbf {v}_m = \mathbf {v}_{pec}/a$. They are valid for arbitrary quintessence potentials as long as the scalar field is sufficiently light, i.e., $m_\phi ^2 \delta \phi = V''(\phi )\delta \phi \ll \varDelta \delta \phi$ for the scales under consideration. For a more detailed discussion see Wintergerst and Pettorino (2010). Combining the above equations yields to the following expression for the evolution of the matter perturbation $\delta _m$:

I.6.16 $$\begin{aligned} \ddot{\delta }_m = -\left( 2{\bar{H}}-\beta \,\dot{{\bar{\phi }}}\right) \,\dot{\delta }_m + \frac{4}{3}\frac{\dot{\delta }_m^2}{1 + \delta _m} + \frac{1 + \delta _m}{a^2} \,\varDelta \varPhi _{\text {eff}}, \end{aligned}$$

Linearization leads to:

I.6.17 $$\begin{aligned} \ddot{\delta }_{m,L} = -\left( 2{{\bar{H}}}-\beta \,\dot{{\bar{\phi }}}\right) \,\dot{\delta }_{m,L} + a^{-2}\,\varDelta \varPhi _{\text {eff}}. \end{aligned}$$

where the effective gravitational potential follows the modified Poisson equation:

I.6.18 $$\begin{aligned} \varDelta {\varPhi _{\text {eff}}} = -\frac{a^2}{2} {{\bar{\rho }}}_m \delta _m \left( 1+2 \beta ^2\right) . \end{aligned}$$

Equations (I.6.16) and (I.6.17) are the two main equations which correctly describe the nonlinear and linear evolution for a coupled dark-energy model. They can be used, among other things, for estimating the extrapolated linear density contrast at collapse $\delta _c$ in the presence of a fifth force. It is possible to reformulate Eqs. (I.6.16) and (I.6.17) into an effective spherical collapse:

I.6.19 $$\begin{aligned} \frac{\ddot{R}}{R} = -\beta \,\dot{\phi }\left( H - \frac{\dot{R}}{R}\right) - \frac{1}{6} \sum _{\alpha } \left[ {\rho }_{\alpha }\left( 1 + 3 { w}_{\alpha }\right) \right] - \frac{1}{3}\,\beta ^2\,\delta \rho _m. \end{aligned}$$

**Fig. 6:**
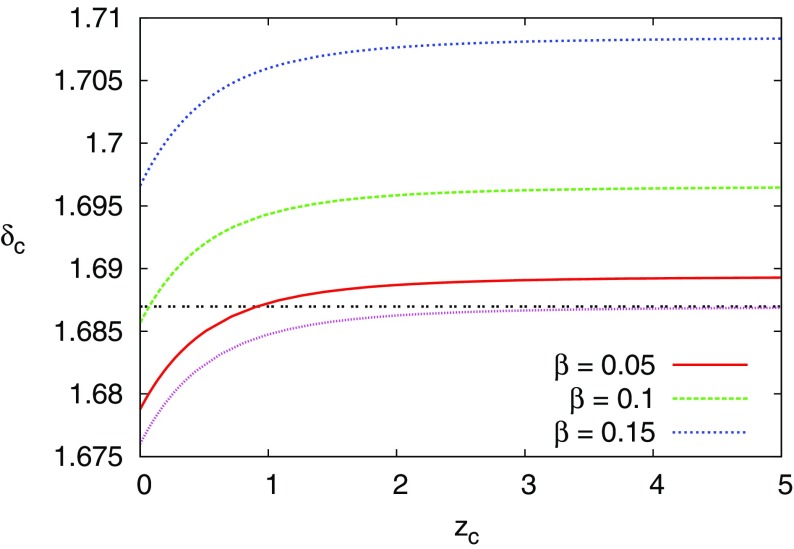
Extrapolated linear density contrast at collapse for coupled quintessence models with different coupling strength $\beta$. For all plots we use a constant $\alpha = 0.1$. We also depict $\delta _c$ for reference $\varLambda$CDM (dotted, pink) and EdS (double-dashed, black) models. Image reproduced by permission from Wintergerst and Pettorino (2010); copyright by APS

Equation (I.6.19) (Mainini and Bonometto 2006; Wintergerst and Pettorino 2010), describes the general evolution of the radius of a spherical overdense region within coupled quintessence. Comparing with the standard case (I.6.9) we notice the presence of two additional terms: a ‘friction’ term and the coupling term $\beta ^2\,\delta \rho _m$, the latter being responsible for the additional attractive fifth force. Note that the ‘friction’ term is actually velocity dependent and its effects on collapse depend, more realistically, on the direction of the velocity, information which is not contained within a *spherical* collapse picture and can be treated within simulations (Baldi and Pettorino 2011; Li et al. 2011; Baldi 2011a, b; Li and Barrow 2011b). We stress that it is crucial to include these additional terms in the equations, as derived from the nonlinear equations, in order to correctly account for the presence of a fifth force. The outlined procedure can easily be generalized to include uncoupled components, for example baryons. In this case, the corresponding evolution equation for $\delta _b$, will be fed by $\varPhi _{\text {eff}} = \varPhi$. This yields an evolution equation for the uncoupled scale factor $R_{uc}$ that is equivalent to the standard Friedmann equation. In Fig. 6, we show the linear density contrast at collapse $\delta _c(z_c)$ for three coupled quintessence models with $\alpha = 0.1$ and $\beta = 0.05$, 0.1, 0.15.

An increase of $\beta$ results in an increase of $\delta _c$. As shown in Wintergerst and Pettorino (2010), $\delta _c(\beta )$ is well described by a simple quadratic fitting formula,

I.6.20 $$\begin{aligned} \delta _c(\beta ) = 1.686\left( 1 + a\beta ^2\right) ,a = 0.556, \end{aligned}$$

valid for small $\beta \lesssim 0.4$ and $z_c \ge 5$. We recall that a nonlinear analysis beyond the spherical collapse method can be addressed by means of the time-renormalization-group method, extended to the case of couple quintessence in Saracco et al. (2010).

**Fig. 7:**
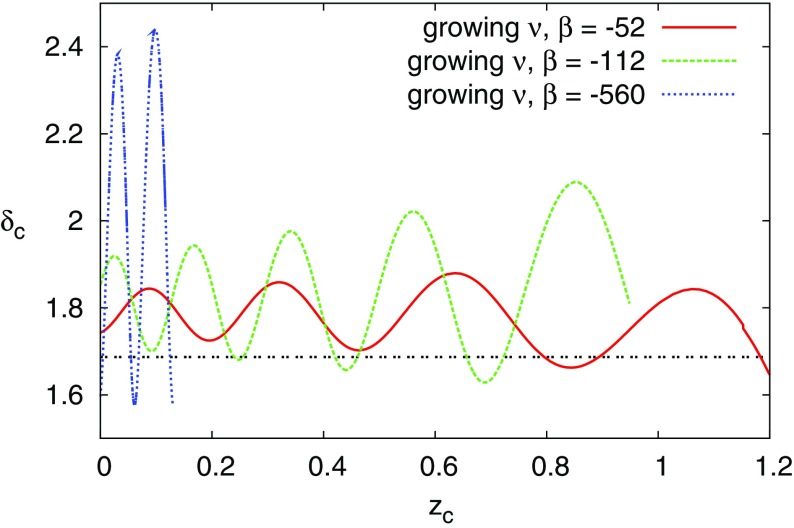
Extrapolated linear density contrast at collapse $\delta _c$ versus collapse redshift $z_c$ for growing neutrinos with $\beta = -\,52$ (solid, red), $\beta = -\,112$ (long-dashed, green) and $\beta = -\,560$ (short-dashed, blue). A reference EdS model (double-dashed. black) is also shown. Image reproduced by permission from Wintergerst and Pettorino (2010); copyright by APS

If a coupling between dark energy and neutrinos is present, as described in Sects. I.5.3 and II.8, bound neutrino structures may form within these models (Brouzakis et al. 2008). It was shown in Mota et al. (2008) that their formation will only start after neutrinos become non-relativistic. A nonlinear treatment of the evolution of neutrino densities is thus only required for very late times, and one may safely neglect neutrino pressure as compared to their density. The evolution Eqs. (I.6.16) and (I.6.17) can then also be applied for the nonlinear and linear neutrino density contrast. The extrapolated linear density at collapse $\delta _c$ for growing neutrino quintessence reflects in all respects the characteristic features of this model and results in a $\delta _c$ which looks quite different from standard dark-energy cosmologies. We have plotted the dependence of $\delta _c$ on the collapse redshift $z_c$ in Fig. 7 for three values of the coupling. The oscillations seen are the result of the oscillations of the neutrino mass caused by the coupling to the scalar field: the latter has characteristic oscillations as it approaches the minimum of the effective potential in which it rolls, given by a combination of the self-interaction potential $U(\phi )$ and the coupling contribution $\beta (1-3{ w}_\nu ){\rho }_\nu$. Furthermore, due to the strong coupling $\beta$, the average value of $\delta _c$ is found to be substantially higher than 1.686, corresponding to the Einstein de Sitter value, shown in black (double-dashed) in Fig. 7. Such an effect can have a strong impact on structure formation and on CMB (Pettorino et al. 2010). For the strongly coupled models, corresponding to a low present day neutrino mass $m_\nu (t_0)$, the critical density at collapse is only available for $z_c \lesssim 0.2$, 1 for $\beta = -\,560$, $-\,112$, respectively. This is again a reflection of the late transition to the non-relativistic regime. Nonlinear investigations of single lumps beyond the spherical collapse picture was performed in Wintergerst et al. (2010) and Brouzakis et al. (2011), the latter showing the influence of the gravitational potentials induced by the neutrino inhomogeneities on the acoustic oscillations in the baryonic and dark-matter spectra.

**Fig. 8:**
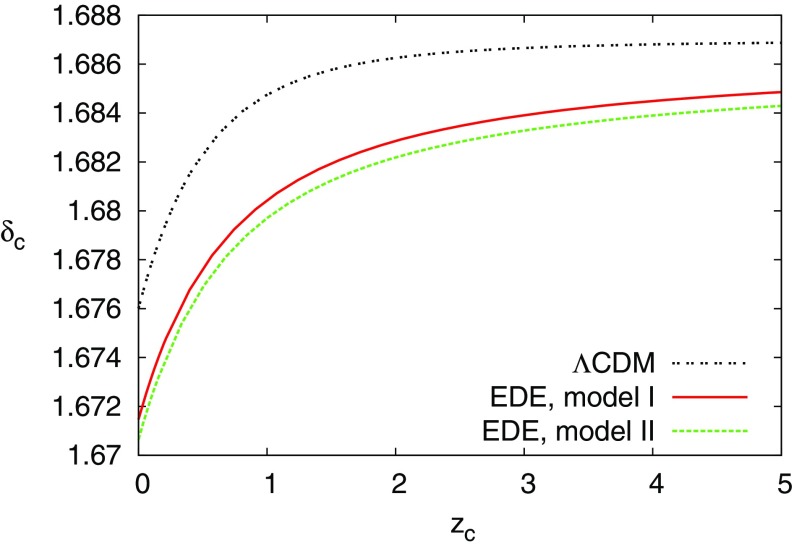
Extrapolated linear density contrast at collapse $\delta _c$ versus collapse redshift $z_c$ for EDE models I (solid, red) and II (long-dashed, green), as well as $\varLambda$CDM (double-dashed, black). Image reproduced by permission from Wintergerst and Pettorino (2010); copyright by APS

*I.6.2.3 Early dark energy*

A convenient way to parametrize the presence of a nonnegligible homogeneous dark-energy component at early times was presented in Wetterich (2004) and has been illustrated in Sect. I.2.1 of the present review. If we specify the spherical collapse equations for this case, the nonlinear evolution of the density contrast follows the evolution Eqs. (I.6.16) and (I.6.17) without the terms related to the coupling. As before, we assume relativistic components to remain homogeneous. In Fig. 8, we show $\delta _c$ for two models of early dark energy, namely models I and II, corresponding to the choices ($\varOmega _{m,0} = 0.332, w_0 = -\,0.93, \varOmega _{\text {DE},e} = 2\times 10^{-4}$) and ($\varOmega _{m,0} = 0.314, w_0 = -\,0.99, \varOmega _{\text {DE},e} = 8\times 10^{-4}$) respectively. Results show $\delta _c(z_c = 5) \sim \,1.685$ ($\sim 5\times 10^{-2}\%$) (Francis et al. 2008b; Wintergerst and Pettorino 2010).

*I.6.2.4 Universal couplings*

In Kopp et al. (2013), the authors compute the critical density of collapse for spherically symmetric overdensities in a class of *f*(*R*) modified gravity models. They evolve the Einstein, scalar field and non-linear fluid equations,under the assumptions that system remains quasi-static throughout the collapse. The result of this analysis is a fitting function for the spherical collapse $\delta _{c}$ as a function of collapse redshift, mass of the overdensity and $f_{\mathrm{R0}}$

I.6.21 $$\begin{aligned} \delta _c\left( z,M,f_{\mathrm{R0}}\right)&=\delta ^{\varLambda }_c(z) \Bigg \{ 1+ b_2 (1+z)^{-a_3} \left( m_{b} -\sqrt{m_{b}^2+1}\right) \nonumber \\&\qquad +\, b_3(\tanh m_{b}-1) \Bigg \}\nonumber \\ m_{b}(z,M,f_{\mathrm{R0}})&=(1+z)^{a_3}\left( \log _{10} \left[ M/\left( M_\odot h^{-1}\right) \right] -m_1(1+z)^{-a_4}\right) \nonumber \\ m_1\left( f_{\mathrm{R0}}\right)&= 1.99 \log _{10}f_{\mathrm{R0}}+26.21 \nonumber \\ b_2&= 0.0166\nonumber \\ b_3 \left( f_{\mathrm{R0}}\right)&=0.0027 \cdot \left( 2.41-\log _{10}f_{\mathrm{R0}}\right) \nonumber \\ a_3\left( f_{\mathrm{R0}}\right)&= 1 + 0.99 \exp \left[ -\,2.08 \left( \log _{10}f_{\mathrm{R0}} + 5.57\right) ^2\right] \nonumber \\ a_4\left( f_{\mathrm{R0}}\right)&= \left( \tanh \left[ 0.69\cdot \left( \log _{10}f_{\mathrm{R0}} + 6.65\right) \right] + 1\right) 0.11. \end{aligned}$$

**Fig. 9:**
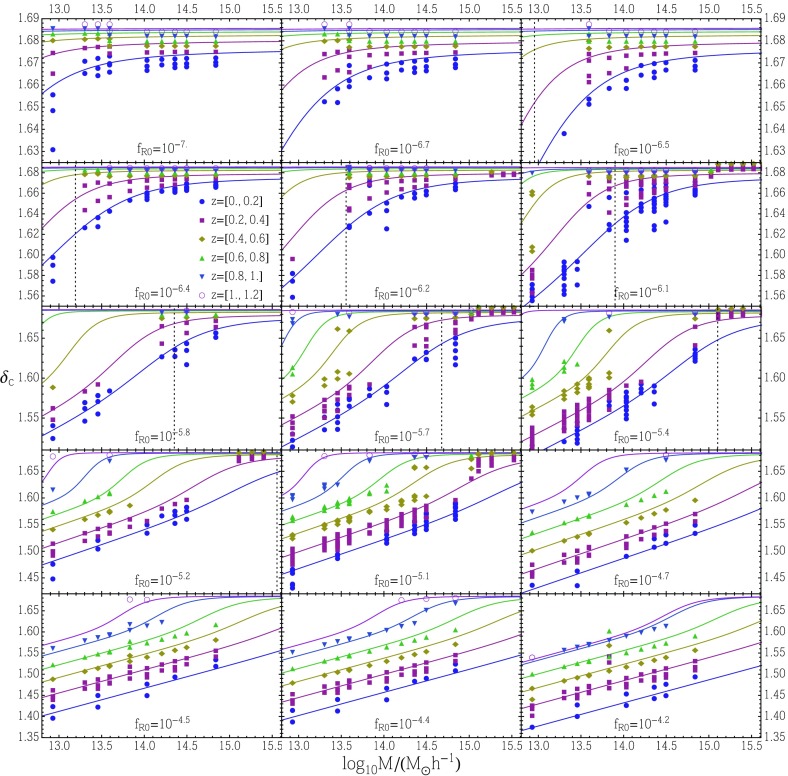
$\delta _{c}$ as a function of mass. In each panel we show the results from the numerical analysis (points) and from the fitting function (lines). Image reproduced with permission from Kopp et al. (2013), copyright by APS

The results of the numerical collapse simulation and the fitting function are shown in Fig. 9.

In Kopp et al. (2013), Achitouv et al. (2016), the authors extend $\delta _{c}$ into drifting and diffusing barrier within the context of excursion set theory. With this procedure they obtain an ‘analytical’ mass function for *f*(*R*) models. The analytic formula for the halo mass function is tested against Monte Carlo random walks for a wide class of moving barriers and can therefore be applied to other modified gravity theories. In addition the results are compared to the results from N-body simulations obtained by the method described in Puchwein et al. (2013)

**Fig. 10:**
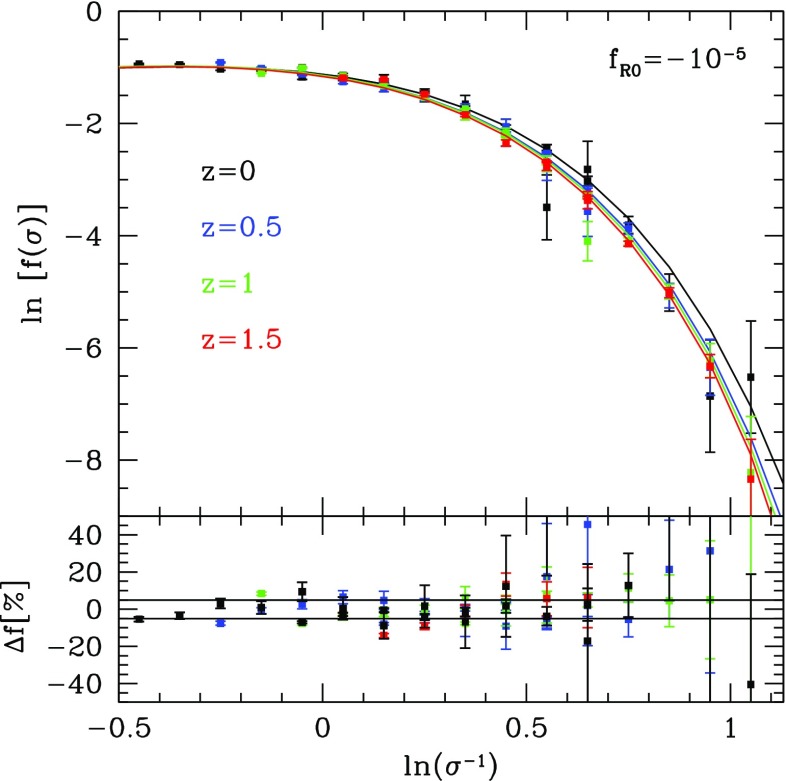
Mass function for $f_{\mathrm{R0}}=-\,10^{-5}$ at redshifts $z=0{-}1.5$. The solid lines are theoretical predictions, squares with errorbars are from simulations. The lower panel shows the relative difference. The black solid lines show $\pm \,5\%$ differences. Image reproduced with permission from Achitouv et al. (2016), copyright by APS

In Fig. 10, we show an example of the derived mass function computed for $f(R_0)=10^{-5}$ for different redshits (solid lines). The comparison to the simulations (points), shows good agreement, but not high-precision agreement as would be required for a detailed cosmological data analysis using the mass function.

### Observational properties of dark energy and modified gravity

Both scalar field dark-energy models and modifications of gravity can in principle lead to any desired expansion history *H*(*z*), or equivalently any evolution of the effective dark-energy equation of state parameter *w*(*z*). For canonical scalar fields, this can be achieved by selecting the appropriate potential $V(\varphi )$ along the evolution of the scalar field $\varphi (t)$, as was done, e.g., in Bassett et al. (2002). For modified gravity models, the same procedure can be followed for example for *f*(*R*) type models (e.g., Pogosian and Silvestri 2008). The evolution history on its own can thus not tell us very much about the physical nature of the mechanism behind the accelerated expansion (although of course a clear measurement showing that $w \ne -1$ would be a sensational discovery). A smoking gun for modifications of gravity can thus only appear at perturbation level.

In the next subsections, we explore how dark energy or modified gravity effects can be detected through weak lensing and redshift surveys.

#### General remarks

Quite generally, cosmological observations fall into two categories: geometrical probes and structure formation probes. While the former provide a measurement of the Hubble function, the latter are a test of the gravitational theory in an almost Newtonian limit on subhorizon scales. Furthermore, possible effects on the geodesics of test particles need to be derived: naturally, photons follow null-geodesics while massive particles, which constitute the cosmic large-scale structure, move along geodesics for non-relativistic particles.

In some special cases, modified gravity models predict a strong deviation from the standard Friedmann equation as in, e.g., DGP, (I.5.58). While the Friedmann equation is not known explicitly in more general models of massive gravity (cascading gravity or hard mass gravity), similar modifications are expected to arise and provide characteristic features, (see, e.g., Afshordi et al. 2009; Jain and Khoury 2010) that could distinguish these models from other scenarios of modified gravity or with additional dynamical degrees of freedom.

In general, however, the most interesting signatures of modified gravity models are to be found in the perturbation sector. For instance, in DGP, growth functions differ from those in dark-energy models by a few percent for identical Hubble functions, and for that reason, an observation of both the Hubble and the growth function gives a handle on constraining the gravitational theory (Lue et al. 2004). The growth function can be estimated both through weak lensing and through galaxy clustering and redshift distortions.

Concerning the interactions of light with the cosmic large-scale structure, one sees a modified coupling in general models and a difference between the metric potentials. These effects are present in the anisotropy pattern of the CMB, as shown in Sawicki and Carroll (2005), where smaller fluctuations were found on large angular scales, which can possibly alleviate the tension between the CMB and the $\varLambda$CDM model on small multipoles where the CMB spectrum acquires smaller amplitudes due to the ISW-effect on the last-scattering surface, but provides a worse fit to supernova data. An interesting effect inexplicable in GR is the anticorrelation between the CMB temperature and the density of galaxies at high redshift due to a sign change in the integrated Sachs–Wolfe effect. Interestingly, this behavior is very common in modified gravity theories.

A very powerful probe of structure growth is of course weak lensing, but to evaluate the lensing effect it is important to understand the nonlinear structure formation dynamics as a good part of the total signal is generated by small structures. Only recently has it been possible to perform structure formation simulations in modified gravity models, although still without a mechanism in which GR is recovered on very small scales, necessary to be in accordance with local tests of gravity.

In contrast, the number density of collapsed objects relies only little on nonlinear physics and can be used to investigate modified gravity cosmologies. One needs to solve the dynamical equations for a spherically symmetric matter distribution. Modified gravity theories show the feature of lowering the collapse threshold for density fluctuations in the large-scale structure, leading to a higher comoving number density of galaxies and clusters of galaxies. This probe is degenerate with respect to dark-energy cosmologies, which generically give the same trends.

Finally, supernova observations—able of accurately mapping the expansion history of the universe—are themselves lensed by foreground matter structures. This extra spread in the Hubble diagram caused by lensing contains precious clustering information, which is encoded in the one-point lensing PDF and can be used to constrain parameters such as the power spectrum normalization $\sigma _8$ or the growth index $\gamma$. Therefore, forthcoming supernova catalogs can be seen as both geometrical and structure formation probes. It is important to point out that the one-point statistics is independent of and complementary to the methods based on cosmic shear and cluster abundance observables. See Marra et al. (2013c) and Quartin et al. (2014) for more details and references therein.

#### Observing modified gravity with weak lensing

The magnification matrix is a $2\times 2$ matrix that relates the true shape of a galaxy to its image. It contains two distinct parts: the convergence, defined as the trace of the matrix, modifies the size of the image, whereas the shear, defined as the symmetric traceless part, distorts the shape of the image. At small scales the shear and the convergence are not independent. They satisfy a consistency relation, and they contain therefore the same information on matter density perturbations. More precisely, the shear and the convergence are both related to the sum of the two Bardeen potentials, $\varPhi +\varPsi$, integrated along the photon trajectory. At large scales however, this consistency relation does not hold anymore. Various relativistic effects contribute to the convergence, see Bonvin (2008). Some of these effects are generated along the photon trajectory, whereas others are due to the perturbations of the galaxies redshift. These relativistic effects provide independent information on the two Bardeen potentials, breaking their degeneracy. The convergence is therefore a useful quantity that can increase the discriminatory power of weak lensing.

The convergence can be measured through its effect on the galaxy number density, see e.g., Broadhurst et al. (1995). The standard method extracts the magnification from correlations of distant quasars with foreground clusters, see Scranton et al. (2005), Menard et al. (2010). Recently, Zhang and Pen (2005, 2006) designed a new method that permits to accurately measure auto-correlations of the magnification, as a function of the galaxies redshift. This method potentially allows measurements of the relativistic effects in the convergence.

*I.7.2.1 Magnification matrix*

We are interested in computing the magnification matrix $\mathcal {D}_{ab}$ in a perturbed Friedmann universe. The magnification matrix relates the true shape of a galaxy to its image, and describes therefore the deformations encountered by a light bundle along its trajectory. $\mathcal {D}_{ab}$ can be computed by solving Sachs equation, see Sachs (1961), that governs propagation of light in a generic geometry. The convergence $\kappa$ and the shear $\gamma \equiv \gamma _1+i \gamma _2$ are then defined respectively as the trace and the symmetric traceless part of $\mathcal {D}_{ab}$

I.7.1 $$\begin{aligned} \mathcal {D}_{ab}=\frac{\chi _S}{1+z_S}\left( \begin{array}{l@{\quad }l} 1-\kappa -\gamma _1&{}-\gamma _2\\ -\gamma _2&{}1-\kappa +\gamma _1 \end{array}\right) . \end{aligned}$$

Here $z_S$ is the redshift of the source and $\chi _S$ is a time coordinate related to conformal time $\eta _S$ through $\chi _S=\eta _O-\eta _S$.

In this section we consider a spatially flat ($K=0$) Friedmann universe with scalar perturbations. We start from the usual longitudinal (or Newtonian) gauge where the metric is given by

I.7.2 $$\begin{aligned} g_{\mu \nu } \, \mathrm {d}x^\mu \, \mathrm {d}x^\nu = a^2\left[ -(1+2\varPsi )d\eta ^2 + (1-2\varPhi )\delta _{ij} \, \mathrm {d}x^i \, \mathrm {d}x^j\right] . \end{aligned}$$

We compute $\mathcal {D}_{ab}$ at linear order in $\varPhi$ and $\varPsi$ and then we extract the shear and the convergence. We find, see Bonvin (2008) and Bernardeau et al. (2010) 
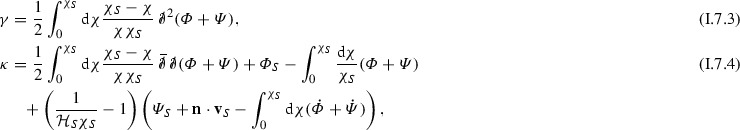


where $\mathbf {n}$ is the direction of observation and $\mathbf {v}_S$ is the peculiar velocity of the source. Here we are making use of the angular spin raising  and lowering  operators (see e.g., Lewis et al. 2002 for a review of the properties of these operators) defined as 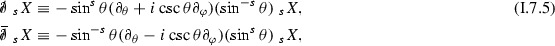


where ${}_s X$ is an arbitrary field of spin *s* and $\theta$ and $\varphi$ are spherical coordinates.

Eq. (I.7.3) and the first term in Eq. (I.7.4) are the standard contributions of the shear and the convergence, but expressed here with the full-sky transverse operators 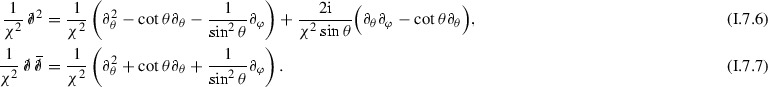 In the flat-sky approximation, where $\theta$ is very small,  reduces to the 2D Laplacian $\partial _x^2+\partial _y^2$ and one recovers the standard expression for the convergence. Similarly, the real part of  that corresponds to $\gamma _1$ reduces to $\partial _y^2-\partial _x^2$ and the imaginary part that corresponds to $\gamma _2$ becomes $\partial _x\partial _y$.

The other terms in Eq. (I.7.4) are relativistic corrections to the convergence, that are negligible at small scales but may become relevant at large scales. The terms in the first line are intrinsic corrections, generated respectively by the curvature perturbation at the source position and the Shapiro time-delay. The terms in the second line are due to the fact that we measure the convergence at a fixed redshift of the source $z_S$ rather that at a fixed conformal time $\eta _S$. Since in a perturbed universe, the observable redshift is itself a perturbed quantity, this transformation generates additional contribution to the convergence. Those are respectively the Sachs–Wolfe contribution, the Doppler contribution and the integrated Sachs–Wolfe contribution. Note that we have neglected the contributions at the observer position since they only give rise to a monopole or dipole term. The dominant correction to the convergence is due to the Doppler term. Therefore in the following we are interested in comparing its amplitude with the amplitude of the standard contribution. To that end we define $\kappa _{\mathrm {st}}$ and $\kappa _{\mathrm {vel}}$ as 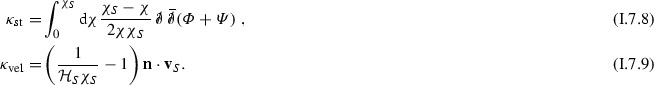


*I.7.2.2 Observable quantities*

The convergence is not directly observable. However it can be measured through the modifications that it induces on the galaxy number density. Let us introduce the magnification

I.7.10 $$\begin{aligned} \mu =\frac{1}{\det \mathcal {D}} \simeq 1+2 \kappa , \quad \text{ when } \quad |\kappa |, |\gamma | \ll 1. \end{aligned}$$

The magnification modifies the size of a source: $d\varOmega _O=\mu d\varOmega _S$, where $d\varOmega _S$ is the true angular size of the source and $d\varOmega _O$ is the solid angle measured by the observer, i.e. the size of the image. The magnification has therefore an impact on the observed galaxy number density. Let us call $\bar{n}(f)df$ the number of unlensed galaxies per unit solid angle, at a redshift $z_S$, and with a flux in the range $[f,f+df]$. The magnification $\mu$ modifies the flux measured by the observer, since it modifies the observed galaxy surface. It affects also the solid angle of observation and hence the number of galaxies per unit of solid angle. These two effects combine to give a galaxy number overdensity, see Broadhurst et al. (1995) and Scranton et al. (2005)

I.7.11 $$\begin{aligned} \delta ^\mu _g=\frac{n(f)-\bar{n}(f)}{\bar{n}(f)} \simeq 1+2\big (\alpha -1\big )(\kappa _{\mathrm {st}}+\kappa _{\mathrm {vel}}) . \end{aligned}$$

Here $\alpha \equiv -N'(>f_c)f_c/N(f_c)$, where $N(>f_c)$ is the number of galaxies brighter than $f_c$ and $f_c$ is the flux limit adopted. Hence $\alpha$ is an observable quantity, see e.g., Zhang and Pen (2005) and Scranton et al. (2005). Recent measurements of the galaxy number overdensity $\delta ^\mu _g$ are reported in Scranton et al. (2005) and Menard et al. (2010). The challenge in those measurements is to eliminate intrinsic clustering of galaxies, which induces an overdensity $\delta _g^{cl}$ much larger than $\delta _g^\mu$. One possibility to separate these two effects is to correlate galaxy number overdensities at widely separated redshifts. One can then measure $\langle \delta _g^\mu (z_S)\delta _g^{cl}(z_{S'})\rangle$, where $z_S$ is the redshift of the sources and $z_{S'}<z_S$ is the redshift of the lenses. Another possibility, proposed by Zhang and Pen (2005, 2006), is to use the unique dependence of $\delta ^\mu _g$ on galaxy flux (i.e., on $\alpha$) to disentangle $\delta ^\mu _g$ from $\delta _g^{cl}$. This method, combined with precise measurements of the galaxies redshift, allows to measure auto-correlations of $\delta ^\mu _g$, i.e., $\langle \delta _g^\mu (z_S)\delta _g^{\mu }(z_{S'})\rangle$, either for $z_S\ne z_{S'}$ or for $z_S=z_{S'}$. The velocity contribution, $\kappa _{\mathrm {vel}}$, has only an effect on $\langle \delta _g^\mu (z_S)\delta _g^{\mu }(z_{S'})\rangle$. The correlations between $\delta _g^{cl}(z_{S'})$ and $\mathbf {v}_S$ are indeed completely negligible and hence the source peculiar velocity does not affect $\langle \delta _g^\mu (z_S)\delta _g^{cl}(z_{S'})\rangle$. In the following we study in detail the contribution of peculiar motion to $\langle \delta _g^\mu (z_S)\delta _g^{\mu }(z_S)\rangle$.

The two components of the convergence $\kappa _{\mathrm {st}}$ and $\kappa _{\mathrm {vel}}$ (and consequently the galaxy number overdensity) are functions of redshift $z_S$ and direction of observation $\mathbf {n}$. We can therefore determine the angular power spectrum

I.7.12 $$\begin{aligned} \left\langle \delta ^\mu _g\left( z_S,\mathbf {n}\right) \delta ^\mu _g(z_S,\mathbf {n}')\right\rangle = \sum _{\ell }\frac{2\ell +1}{4\pi }C_\ell (z_S)P_\ell \left( \mathbf {n}\cdot \mathbf {n}'\right) . \end{aligned}$$

The angular power spectrum $C_\ell (z_S)$ contains two contributions, generated respectively by $\langle \kappa _{\mathrm {st}} \kappa _{\mathrm {st}}\rangle$ and $\langle \kappa _{\mathrm {vel}} \kappa _{\mathrm {vel}}\rangle$. The cross-term $\langle \kappa _{\mathrm {vel}} \kappa _{\mathrm {st}}\rangle$ is negligible since $\kappa _{\mathrm {st}}$ contains only Fourier modes with a wave vector $\mathbf {k}_\perp$ perpendicular to the line of sight [see Eq. (I.7.8)], whereas $\kappa _{\mathrm {vel}}$ selects modes with wave vector along the line of sight [Eq. (I.7.9)].

So far the derivation has been completely generic. Eqs. (I.7.3) and (I.7.4) are valid in any theory of gravity whose metric can be written as in Eq. (I.7.2). To evaluate the angular power spectrum we now have to be more specific. In the following we assume GR, with no anisotropic stress such that $\varPhi =\varPsi$. We use the Fourier transform convention

I.7.13 $$\begin{aligned} \mathbf {v}(\mathbf {x},\chi )=\frac{1}{(2\pi )^3}\int \mathrm {d}^3k \, \mathbf {v}(\mathbf {k},\chi )e^{i\mathbf {k}\mathbf {x}}. \end{aligned}$$

The continuity equation, see e.g., Dodelson (2003), allows us to express the peculiar velocity as

I.7.14 $$\begin{aligned} \mathbf {v}(\mathbf {k},\chi )=-i\frac{\dot{G}(a)}{G(a)}\frac{\mathbf {k}}{k^2} \delta (\mathbf {k},a), \end{aligned}$$

where $\delta (\mathbf {k},a)$ is the density contrast, *G*(*a*) is the growth function, and $\dot{G}(a)$ its derivative with respect to $\chi$. With this we can express the angular power spectrum as

I.7.15 $$\begin{aligned} C_\ell ^{\mathrm {vel}}(z_S)=\frac{16\pi \delta _H^2(\alpha _S-1)^2\dot{G}(a_S)^2}{H_0^4G^2(a=1)} \left( \frac{1}{\mathcal {H}_S \chi _S}-1\right) ^2 \int \mathrm {d}k \, k T^2(k)j_\ell '(k \chi _S)^2. \end{aligned}$$

Here $\delta _H$ is the density contrast at horizon and *T*(*k*) is the transfer function defined through, see e.g., Dodelson (2003)

I.7.16 $$\begin{aligned} \varPsi (\mathbf {k},a)=\frac{9}{10}\varPsi _p(\mathbf {k})T(k)\frac{G(a)}{a}. \end{aligned}$$

We assume a flat power spectrum, $n_s=1$, for the primordial potential $\varPsi _p(\mathbf {k})$. We want to compare this contribution with the standard contribution

I.7.17 $$\begin{aligned} C_\ell ^{\mathrm {st}}(z_S)= & {} \frac{36\pi \delta _H^2(\alpha _S-1)^2\varOmega _m^2\ell ^2(\ell +1)^2}{G^2(a=1)}\int \frac{\mathrm {d}k}{k}T^2(k)\nonumber \\&\times \left[ \int _0^{\chi _S}\mathrm {d}\chi \frac{\chi _S-\chi }{\chi \chi _S}\frac{G(a)}{a}j_\ell (k\chi )\right] ^2 . \end{aligned}$$

**Fig. 11:**
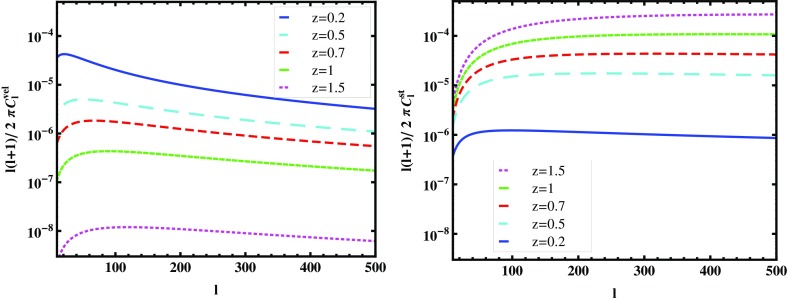
Left: The velocity contribution $C_\ell ^{\mathrm {vel}}$ as a function of $\ell$ for various redshifts. Right: The standard contribution $C_\ell ^{\mathrm {st}}$ as a function of $\ell$ for various redshifts

We evaluate $C_\ell ^{\mathrm {vel}}$ and $C_\ell ^{\mathrm {st}}$ in a $\varLambda$CDM universe with $\varOmega _m = 0.25$, $\varOmega _\varLambda = 0.75$ and $\delta _H=5.7\times 10^{-5}$. We approximate the transfer function with the BBKS formula, see Bardeen et al. (1986). In Fig. 11, we plot $C_\ell ^{\mathrm {vel}}$ and $C_\ell ^{\mathrm {st}}$ for various source redshifts. The amplitude of $C_\ell ^{\mathrm {vel}}$ and $C_\ell ^{\mathrm {st}}$ depends on $(\alpha -1)^2$, which varies with the redshift of the source, the flux threshold adopted, and the sky coverage of the experiment. Since $(\alpha -1)^2$ influences $C_\ell ^{\mathrm {vel}}$ and $C_\ell ^{\mathrm {st}}$ in the same way we do not include it in our plot. Generally, at small redshifts, $(\alpha -1)$ is smaller than 1 and consequently the amplitude of both $C_\ell ^{\mathrm {vel}}$ and $C_\ell ^{\mathrm {st}}$ is slightly reduced, whereas at large redshifts $(\alpha -1)$ tends to be larger than 1 and to amplify $C_\ell ^{\mathrm {vel}}$ and $C_\ell ^{\mathrm {st}}$, see e.g., Zhang and Pen (2006). However, the general features of the curves and more importantly the ratio between $C_\ell ^{\mathrm {vel}}$ and $C_\ell ^{\mathrm {st}}$ are not affected by $(\alpha -1)$.

Figure 11 shows that $C_\ell ^{\mathrm {vel}}$ peaks at rather small $\ell$, between 30 and 120 depending on the redshift. This corresponds to rather large angle $\theta \sim 90\textendash 360\mathrm {\ arcmin}$. This behavior differs from the standard term (Fig. 11) that peaks at large $\ell$. Therefore, it is important to have large sky surveys to detect the velocity contribution. The relative importance of $C_\ell ^{\mathrm {vel}}$ and $C_\ell ^{\mathrm {st}}$ depends strongly on the redshift of the source. At small redshift, $z_S=0.2$, the velocity contribution is about $4\times 10^{-5}$ and is hence larger than the standard contribution which reaches $10^{-6}$. At redshift $z_S=0.5$, $C_\ell ^{\mathrm {vel}}$ is about 20% of $C_\ell ^{\mathrm {st}}$, whereas at redshift $z_S=1$, it is about 1% of $C_\ell ^{\mathrm {st}}$. Then at redshift $z_S=1.5$ and above, $C_\ell ^{\mathrm {vel}}$ becomes very small with respect to $C_\ell ^{\mathrm {st}}$: $C_\ell ^{\mathrm {vel}} \le 10^{-4}\,C_\ell ^{\mathrm {st}}$. The enhancement of $C_\ell ^{\mathrm {vel}}$ at small redshift together with its fast decrease at large redshift are due to the prefactor $\left( \frac{1}{\mathcal {H}_S \chi _S}-1\right) ^2$ in Eq. (I.7.15). Thanks to this enhancement we see that if the magnification can be measured with an accuracy of 10%, then the velocity contribution is observable up to redshifts $z\le 0.6$. If the accuracy reaches 1% then the velocity contribution becomes interesting up to redshifts of order 1.

The shear and the standard contribution in the convergence are not independent. One can easily show that their angular power spectra satisfy the consistency relation, see Hu (2000)

I.7.18 $$\begin{aligned} C_\ell ^{\kappa \, {\mathrm {st}}}=\frac{\ell (\ell +1)}{(\ell +2)(\ell -1)}C_\ell ^\gamma . \end{aligned}$$

This relation is clearly modified by the velocity contribution. Using that the cross-correlation between the standard term and the velocity term is negligible, we can write a new consistency relation that relates the observed convergence $C_\ell ^{\kappa \, {\mathrm {tot}}}$ to the shear

I.7.19 $$\begin{aligned} \frac{\ell (\ell +1)}{(\ell +2)(\ell -1)}C_\ell ^\gamma =C_\ell ^{\kappa \, {\mathrm {tot}}}-C_\ell ^{\kappa \, {\mathrm {vel}}}. \end{aligned}$$

Consequently, if one measures both the shear $C_\ell ^\gamma$ and the magnification $C_\ell ^{\kappa \, {\mathrm {tot}}}$ as functions of the redshift, Eq. (I.7.19) allows to extract the peculiar velocity contribution $C_\ell ^{\kappa \, {\mathrm {vel}}}$. This provides a new way to measure peculiar velocities of galaxies.

Note that in practice, in weak lensing tomography, the angular power spectrum is computed in redshift bins and therefore the square bracket in Eq. (I.7.17) has to be integrated over the bin

I.7.20 $$\begin{aligned} \int _0^\infty \mathrm {d}\chi n_i(\chi )\int _0^{\chi }\mathrm {d}\chi ' \frac{\chi -\chi '}{\chi \chi '}\frac{G\left( \chi '\right) }{a\left( \chi '\right) }j_\ell \left( k\chi '\right) , \end{aligned}$$

where $n_i$ is the galaxy density for the *i*-th bin, convolved with a Gaussian around the mean redshift of the bin. The integral over $\chi '$ is then simplified using Limber approximation, i.e.,

I.7.21 $$\begin{aligned} \int _0^\chi \mathrm {d}\chi ' F\left( \chi '\right) J_\ell \left( k\chi '\right) \simeq \frac{1}{k}F\left( \frac{\ell }{k} \right) \theta (k\chi -\ell ), \end{aligned}$$

where $J_\ell$ is the Bessel function of order $\ell$. The accuracy of Limber approximation increases with $\ell$. Performing a change of coordinate such that $k=\ell /\chi$, Eq. (I.7.17) can be recast in the usual form used in weak lensing tomography, see e.g., Eq. (I.8.4).

#### Observing modified gravity with redshift surveys

Wide-deep galaxy redshift surveys have the power to yield information on both *H*(*z*) and $f_{g}(z)$ through measurements of baryon acoustic oscillations (BAO) and redshift-space distortions. In particular, if gravity is not modified and matter is not interacting other than gravitationally, then a detection of the expansion rate is directly linked to a unique prediction of the growth rate. Otherwise galaxy redshift surveys provide a unique and crucial way to make a combined analysis of *H*(*z*) and $f_{g}(z)$ to test gravity. As a wide-deep survey, Euclid allows us to measure *H*(*z*) directly from BAO, but also indirectly through the angular diameter distance $D_A(z)$ (and possibly distance ratios from weak lensing). Most importantly, Euclid survey enables us to measure the cosmic growth history using two independent methods: $f_g(z)$ from galaxy clustering, and *G*(*z*) from weak lensing. In the following we discuss the estimation of $[H(z), D_A(z)$ and $f_g(z)]$ from galaxy clustering.

From the measure of BAO in the matter power spectrum or in the 2-point correlation function one can infer information on the expansion rate of the universe. In fact, the sound waves imprinted in the CMB can be also detected in the clustering of galaxies, thereby completing an important test of our theory of gravitational structure formation.

The BAO in the radial and tangential directions offer a way to measure the Hubble parameter and angular diameter distance, respectively. In the simplest FLRW universe the basis to define distances is the dimensionless, radial, comoving distance:

I.7.22 $$\begin{aligned} \chi (z) \equiv \int _0^z \frac{\mathrm {d}z'}{E\left( z'\right) }. \end{aligned}$$

The dimensionless version of the Hubble parameter is:

I.7.23 $$\begin{aligned} E^2(z) = \varOmega _m^{(0)}(1+z)^3 +\varOmega _k(1+z)^2 + \left( \varOmega _k-\varOmega _m^{(0)}\right) \exp \left[ \int _0^z \frac{3(1+w(\tilde{z}))}{1+\tilde{z}}\,\mathrm {d}\tilde{z} \right] .\nonumber \\ \end{aligned}$$

The standard cosmological distances are related to $\chi (z)$ via

I.7.24 $$\begin{aligned} D_A(z) = \frac{c}{H_0 (1+z) \sqrt{-\varOmega _k}}\sin \left( \sqrt{-\varOmega _k}\chi (z)\right) \end{aligned}$$

where the luminosity distance, $D_L(z)$, is given by the distance duality:

I.7.25 $$\begin{aligned} D_L(z) = (1+z)^2 D_A(z). \end{aligned}$$

The coupling between $D_A(z)$ and $D_L(z)$ persists in any metric theory of gravity as long as photon number is conserved (see Sect. IV.2 for cases in which the duality relation is violated). BAO yield both $D_A(z)$ and *H*(*z*) making use of an almost completely linear physics (unlike for example SN Ia, demanding complex and poorly understood mechanisms of explosions). Furthermore, they provide the chance of constraining the growth rate through the change in the amplitude of the power spectrum.

The characteristic scale of the BAO is set by the sound horizon at decoupling. Consequently, one can attain the angular diameter distance and Hubble parameter separately. This scale along the line of sight ($s_{||}(z)$) measures *H*(*z*) through $H(z) = c\varDelta z/s_{||}(z)$, while the tangential mode measures the angular diameter distance $D_A(z) = s_{\perp }/\varDelta \theta (1+z)$.

One can then use the power spectrum to derive predictions on the parameter constraining power of the survey (see, e.g., Amendola et al. 2005a; Guzzo et al. 2008; Wang 2008a; Wang et al. 2010; Di Porto et al. 2012).

In order to explore the cosmological parameter constraints from a given redshift survey, one needs to specify the measurement uncertainties of the galaxy power spectrum. In general, the statistical error on the measurement of the galaxy power spectrum $P_{\mathrm {g}}(k)$ at a given wave-number bin is (Feldman et al. 1994)

I.7.26 $$\begin{aligned} \left[ \frac{\varDelta P_{\mathrm {g}}}{P_{\mathrm {g}}}\right] ^2= \frac{2(2\pi )^2 }{V_{\mathrm {survey}}k^2\varDelta k\varDelta \mu } \left[ 1+\frac{1}{n_{\mathrm {g}}P_{\mathrm {g}}}\right] ^2, \end{aligned}$$

where $n_{\mathrm {g}}$ is the mean number density of galaxies, $V_{\mathrm {survey}}$ is the comoving survey volume of the galaxy survey, and $\mu$ is the cosine of the angle between $\mathbf {k}$ and the line-of-sight direction $\mu = \mathbf {k}\cdot \hat{r}/k$.

In general, the *observed* galaxy power spectrum is different from the *true* spectrum, and it can be reconstructed approximately assuming a reference cosmology (which we consider to be our fiducial cosmology) as (e.g., Seo and Eisenstein 2003)

I.7.27 $$\begin{aligned} P_{\mathrm {obs}}\left( k_{{\mathrm {ref}}\perp },k_{{\mathrm {ref}}\parallel },z\right) =\frac{D\!_A(z)_{\mathrm {ref}} ^2 H(z)}{D\!_A(z)^2 H(z)_{\mathrm {ref}}} P_{\mathrm {g}}\left( k_{{\mathrm {ref}}\perp },k_{{\mathrm {ref}}\parallel },z\right) +P_{\mathrm {shot}}, \end{aligned}$$

where

I.7.28 $$\begin{aligned} P_{\mathrm {g}}\left( k_{{\mathrm {ref}}\perp },k_{{\mathrm {ref}}\parallel },z\right) =b(z)^2\left[ 1+\beta (z) \frac{k_{{\mathrm {ref}}\parallel }^2}{k_{{\mathrm {ref}}\perp }^2+k_{{\mathrm {ref}}\parallel }^2}\right] ^2\times P_{\mathrm {matter}}(k,z). \end{aligned}$$

In Eq. (I.7.27), *H*(*z*) and $D_A(z)$ are the Hubble parameter and the angular diameter distance, respectively, and the prefactor $(D\!_A(z)_{\mathrm {ref}} ^2 H(z))/(D\!_A(z)^2 H(z)_{\mathrm {ref}})$ encapsulates the geometrical distortions due to the Alcock–Paczyński effect (Seo and Eisenstein 2003; Ballinger et al. 1996). Their values in the reference cosmology are distinguished by the subscript ‘ref’, while those in the true cosmology have no subscript. $k_\perp$ and $k_\parallel$ are the wave-numbers across and along the line of sight in the true cosmology, and they are related to the wave-numbers calculated assuming the reference cosmology by $k_{{\mathrm {ref}}\perp } = k_\perp D_A(z)/D_A(z)_{\mathrm {ref}}$ and $k_{{\mathrm {ref}}\parallel } = k_\parallel H(z)_{\mathrm {ref}}/H(z)$. $P_{\mathrm {shot}}$ is the unknown white shot noise that remains even after the conventional shot noise of inverse number density has been subtracted (Seo and Eisenstein 2003). In Eq. (I.7.28), *b*(*z*) is the *linear bias* factor between galaxy and matter density distributions, $f_g(z)$ is the linear growth rate,^7^ and $\beta (z)=f_g(z)/b(z)$ is the linear redshift-space distortion parameter (Kaiser 1987). The linear matter power spectrum $P_{\mathrm {matter}}(k,z)$ in Eq. (I.7.27) takes the form

I.7.29 $$\begin{aligned} P_{\mathrm {matter}}(k,z)=\frac{8\pi ^2c^4k_0\varDelta ^2_\mathcal{R}(k_0)}{25 H_0^4\varOmega _{m}^2} T^2(k) \left[ \frac{G(z)}{G(z=0)}\right] ^2 \left( \frac{k}{k_0}\right) ^{n_s}e^{-k^2\mu ^2\sigma _r^2},\qquad \quad \end{aligned}$$

where *G*(*z*) is the usual *scale independent* linear growth-factor in the absence of massive neutrino free-streaming (see Eq. (25) in Eisenstein and Hu 1999), whose fiducial value in each redshift bin is computed through numerical integration of the differential equations governing the growth of linear perturbations in presence of dark energy (Linder and Jenkins 2003) or employing the approximation of Eq. (I.3.22). *T*(*k*) depends on matter and baryon densities^8^ (neglecting dark energy at early times), and is computed in each redshift bin using a Boltzmann code like camb^9^ (Lewis et al. 2000b) or cmbfast.

In Eq. (I.7.29) a damping factor $e^{-k^2\mu ^2\sigma _r^2}$ has been added, due to redshift uncertainties, where $\sigma _r=(\partial r/\partial z)\sigma _z$, *r*(*z*) being the comoving distance (Wang 2010; Seo and Eisenstein 2003), and assumed that the power spectrum of primordial curvature perturbations, $P_{{\mathcal {R}}}(k)$, is

I.7.30 $$\begin{aligned} \varDelta ^2_{{\mathcal {R}}}(k) \equiv \frac{k^3P_{{\mathcal {R}}}(k)}{2\pi ^2} = \varDelta ^2_{{\mathcal {R}}}(k_0)\left( \frac{k}{k_0}\right) ^{n_s}, \end{aligned}$$

where $k_0=0.002/\mathrm {Mpc}$, $\varDelta ^2_\mathcal{R}(k_0)|_{\mathrm {fid}}=2.45\times 10^{-9}$ is the dimensionless amplitude of the primordial curvature perturbations evaluated at a pivot scale $k_0$, and $n_s$ is the scalar spectral index (Larson et al. 2011).

In the limit where the survey volume is much larger than the scale of any features in $P_{\mathrm {obs}}(k)$, it has been shown that the redshift survey Fisher matrix for a given redshift bin can be approximated as (Tegmark 1997)

I.7.31 $$\begin{aligned} F_{ij}^{\mathrm {LSS}}= & {} \int _{-1}^{1} \int _{k_{\min }}^{k_{\max }}\frac{\partial \ln P_{\mathrm {obs}}(k,\mu )}{\partial p_i} \frac{\partial \ln P_{\mathrm {obs}}(k,\mu )}{\partial p_j} V_{\mathrm {eff}}(k,\mu ) \frac{2\pi k^2 \, \mathrm {d}k \, \mathrm {d}\mu }{2(2\pi )^3}, \qquad \quad \end{aligned}$$

where the derivatives are evaluated at the parameter values $p_i$ of the fiducial model, and $V_{\mathrm {eff}}$ is the effective volume of the survey:

I.7.32 $$\begin{aligned} V_{\mathrm {eff}}(k,\mu ) = \left[ \frac{{n_{\mathrm {g}}}P_{\mathrm {g}}(k,\mu )}{{n_{\mathrm {g}}}P_{\mathrm {g}}(k,\mu )+1} \right] ^2 V_{\mathrm {survey}}, \end{aligned}$$

where the comoving number density $n_{\mathrm {g}}(z)$ is assumed to be spatially constant. Due to azimuthal symmetry around the line of sight, the three-dimensional galaxy redshift power spectrum $P_{\mathrm {obs}}(\mathbf {k})$ depends only on *k* and $\mu$, i.e., is reduced to two dimensions by symmetry (Seo and Eisenstein 2003). The total Fisher matrix can be obtained by summing over the redshift bins.

To minimize nonlinear effects, one should restrict wave-numbers to the quasi-linear regime, e.g., imposing that $k_{\max }$ is given by requiring that the variance of matter fluctuations in a sphere of radius *R* is, for instance, $\sigma ^2(R)=0.25$ for $R=\pi /(2k_{\max })$. Or one could model the nonlinear distortions as in Eisenstein et al. (2007). On scales larger than ($\sim \,100\, h^{-1}\mathrm {\ Mpc}$) where we focus our analysis, nonlinear effects can be represented in fact as a displacement field in Lagrangian space modeled by an elliptical Gaussian function. Therefore, following Eisenstein et al. (2007) and Seo and Eisenstein (2007), to model nonlinear effect we multiply *P*(*k*) by the factor

I.7.33 $$\begin{aligned} \exp \left\{ -k^{2}\left[ \frac{\left( 1-\mu ^{2}\right) \varSigma _{\perp }^{\,2}}{2}+\frac{\mu ^{2}\varSigma _{ \parallel }^{\,2}}{2}\right] \right\} , \end{aligned}$$

where $\varSigma _{\perp }$ and $\varSigma _{\parallel }$ represent the displacement across and along the line of sight, respectively. They are related to the growth factor *G* and to the growth rate $f_g$ through $\varSigma _{\perp }=\varSigma _{0}G$ and $\varSigma _{\parallel }=\varSigma _{0}G(1+f_g)$. The value of $\varSigma _{0}$ is proportional to $\sigma _{8}$. For a reference cosmology where $\sigma _{8}=0.8$ (Komatsu et al. 2011), we have $\varSigma _{0}=11\, h^{-1}\mathrm {\ Mpc}$.

Finally, we note that when actual data are available, the usual way to measure $\beta =f_g/b$ is by fitting the measured galaxy redshift-space correlation function $\xi (\sigma ,\pi )$ to a model (Peebles 1980):

I.7.34 $$\begin{aligned} \xi (\sigma ,\pi )= \int _{-\infty }^{\infty }{\mathrm {d}}v\, f(v)\, \tilde{\xi }(\sigma ,\pi -v/H_0), \end{aligned}$$

where *f*(*v*) describes the small-scale random motion (usually modeled by a Gaussian that depends on the galaxy pairwise peculiar velocity dispersion), and $\tilde{\xi }(\sigma ,\pi )$ is the model accounting for coherent infall velocities^10^:

I.7.35 $$\begin{aligned} \tilde{\xi }(\sigma ,\pi )=\xi _0(s) P_0(\mu ) +\xi _2(s) P_2(\mu )+ \xi _4(s) P_4(\mu ). \end{aligned}$$

$P_l(\mu )$ are Legendre polynomials; $\mu =\cos \theta$, where $\theta$ denotes the angle between $\mathbf {r}$ and $\pi$; $\xi _0(s)$, $\xi _2(s)$, and $\xi _4(s)$ depend on $\beta$ and the real-space correlation function $\xi (r)$.

The bias between galaxy and matter distributions can be estimated from either galaxy clustering, or weak lensing. To determine bias, we can assume that the galaxy density perturbation $\delta _g$ is related to the matter density perturbation $\delta _m(\mathbf {x})$ as (Fry and Gaztanaga 1993):

I.7.36 $$\begin{aligned} \delta _g= b \delta _m(\mathbf {x})+ b_2 \delta _m^2(\mathbf {x})/2. \end{aligned}$$

Bias can be derived from galaxy clustering by measuring the galaxy bispectrum:I.7.37where *J* is a function that depends on the shape of the triangle formed by ($\mathbf {k}_1$, $\mathbf {k}_2$, $\mathbf {k}_3$) in $\mathbf {k}$ space, but only depends very weakly on cosmology (Matarrese et al. 1997; Verde et al. 2002).

In general, bias can be measured from weak lensing through the comparison of the shear-shear and shear-galaxy correlations functions. A combined constraint on bias and the growth factor *G*(*z*) can be derived from weak lensing by comparing the cross-correlations of multiple redshift slices.

Of course, if bias is assumed to be linear ($b_2=0$) and scale independent, or is parametrized in some simple way, e.g., with a power law scale dependence, then it is possible to estimate it even from linear galaxy clustering alone, as we will see in Sect. I.8.3.

#### Constraining modified gravity with galaxy–CMB correlations

Two of the above-mentioned observable signatures of dark energy and modified gravity are especially suitable to study the time evolution of dark energy at the perturbative level: the ISW effect and CMB lensing. Both effects produce sub-dominant secondary anisotropies imprinted on the CMB at late times, and can be measured as a function of redshift by cross-correlating CMB temperature and lensing maps with galaxy surveys, thus allowing a tomographic analysis of the dark energy properties.

*I.7.4.1 The ISW effect*

The CMB photons freely streaming in the late universe encounter over- and under-densities; their energy will thus change as a function of time as the photons climb in and out of potential wells, but the average net energy gain will be null as long as the potentials are globally constant in time. Since the potentials decay in the presence of cosmic acceleration, a non-zero ISW effect will be produced in this case, corresponding to a temperature anisotropy in the direction $\hat{{\mathbf {n}}}$

I.7.38 $$\begin{aligned} \frac{\varDelta T}{T} (\hat{{\mathbf {n}}}) = \int d \eta \, e^{-\tau (z)} \, \left( {\dot{\varPhi }} + {\dot{\varPsi }} \right) \left[ \eta , \hat{\mathbf n} (\eta _0 - \eta ) \right] , \end{aligned}$$

where dots indicate derivatives with respect to the conformal time $\eta$, $\tau$ is the optical depth, and $\varPhi , \varPsi$ are the Newtonian gauge potentials describing the time and space metric perturbations, respectively. This effect is subdominant with respect to the primary CMB temperature anisotropies produced at primordial times, from which it can however be extracted by cross-correlating the full CMB maps with galaxy catalogues, which are correlated with the ISW signal since the galaxy overdensities also trace the same gravitational potentials (Crittenden and Turok 1996).

The ISW has been detected, in agreement with the $\varLambda$CDM predictions, at the $\sim \,4\, \sigma$ significance level by cross-correlating *WMAP* and *Planck* CMB data with numerous galaxy catalogues: see Ho et al. (2008), Giannantonio et al. (2008), Giannantonio et al. (2012a) and references therein.

Future galaxy surveys including the *Euclid* satellite are expected to improve current ISW measurements by increasing redshift depth and survey volume, thus allowing a consistent tomographic study from one galaxy survey, as well as by improving the control of systematics; the total signal-to-noise is however not expected to exceed the $\sim \,8 \,\sigma$ level (Crittenden and Turok 1996) in the $\varLambda$CDM scenario, since the ISW signal peaks on the largest scales, which are dominated by cosmic variance. The measurement of ISW at high redshift has however a significant discovery potential, as in case exotic dark energy models are correct, the actual level of ISW may be significantly higher.

*I.7.4.2 CMB lensing*

An additional, complementary observable is provided by CMB lensing (Lewis and Challinor 2006). This is a special case of the weak gravitational lensing described above, where the sources are set to the CMB last-scattering surface at redshift $z_{\star } \simeq 1100$. In this case, the primary CMB lensing map is deflected by the intervening large-scale structure by small angles of the order $\sim \, 2.5$ arcmin, by the effect of a lensing potential $\varphi$ in a direction $\hat{{\mathbf {n}}}$ given by

I.7.39 $$\begin{aligned} \varphi (\hat{{\mathbf {n}}}) = - \int d \chi \, \frac{\chi _{*} - \chi }{\chi _{*} \chi } \left( \varPhi + \varPsi \right) \left[ \chi \hat{{\mathbf {n}}}, \eta _0 - \chi \right] , \end{aligned}$$

where $\chi$ is the conformal distance. This potential is simply related to the convergence $\kappa$ used above in multipole space by $\kappa _{lm} = l(l+1) \varphi _{lm}/2$. The effect of lensing on the CMB temperature anisotropies is a smoothing of the peaks an troughs in the angular power spectrum.

Maps of the CMB lensing potential have been reconstructed from higher-order statistics of the CMB temperature maps by the *Planck* (Planck Collaboration 2014b), south pole telescope (van Engelen et al. 2012) and atacama cosmology telescope (Das et al. 2011b) surveys; cross-correlations between these lensing maps and galaxy surveys have also been confirmed with these three data sets (see, e.g., Giannantonio and Percival 2014): such cross-correlations allow once again to study the redshift evolution of the gravitational potentials, and thus the physical properties of the dark sector.

Upcoming and future galaxy surveys leading up to the *Euclid* satellite mission, combined with rapidly improving CMB data, will increase the signal-to-noise of the CMB lensing cross-correlations well beyond the current levels, since the CMB lensing signal is maximum on smaller scales, which are currently dominated by statistical and systematic errors, but not by cosmic variance.

#### Cosmological bulk flows

As we have seen, the additional redshift induced by the galaxy peculiar velocity field generates the redshift distortion in the power spectrum. In this section we discuss a related effect on the luminosity of the galaxies and on its use to measure the peculiar velocity in large volumes, the so-called bulk flow.

In the gravitational instability framework, inhomogeneities in the matter distribution induce gravitational accelerations $\mathbf {g}$, which result in galaxies having peculiar velocities $\mathbf {v}$ that add to the Hubble flow. In linear theory the peculiar velocity field is proportional to the peculiar acceleration

I.7.40 $$\begin{aligned} \mathbf {v}(\mathbf {r})=\frac{2f_g}{3H_0\varOmega _m}\mathbf {g}(\mathbf {r})= \frac{H_0 f_g}{4\pi } \int \delta _m\left( \mathbf {r^\prime }\right) \frac{\left( \mathbf {r^\prime } - \mathbf {r}\right) }{|\mathbf {r^\prime }-\mathbf {r}|^3} \,\mathrm {d}^3\mathbf {r^\prime }, \end{aligned}$$

and the bulk flow of a spherical region is solely determined by the gravitational pull of the dipole of the external mass distribution. For this reason, bulk flows are reliable indicators to deviations from homogeneity and isotropy on large scale, should they exist.

Constraints on the power spectrum and growth rate can be obtained by comparing the bulk flow estimated from the volume-averaged motion of the sphere of radius *R*:

I.7.41 $$\begin{aligned} \mathbf {B}_{R}\equiv \frac{\int \mathbf {v}(\mathbf {x}) W(\mathbf {x}/R)\,\mathrm {d}^3\mathbf {x}}{\int W(\mathbf {x}/R)\,\mathrm {d}^3\mathbf {x}}, \end{aligned}$$

with expected variance:

I.7.42 $$\begin{aligned} \sigma ^2_{\mathbf {B},{R}}= \frac{H_0^2 f_g^2}{6\pi ^2}\int P(k)\mathcal{W}(kR)^2(k)\,\mathrm {d}k, \end{aligned}$$

where the window function $W(\mathbf {x}/R)$ and its Fourier transform ${{\mathcal {W}}}(kR)$ describe the spatial distribution of the dataset.

Over the years the bulk flows has been estimated from the measured peculiar velocities of a large variety of objects ranging from galaxies (Giovanelli et al. 1998a, b; Dekel et al. 1999; Courteau et al. 2000; da Costa et al. 2000; Sarkar et al. 2007) clusters of galaxies (Lauer and Postman 1994; Branchini et al. 1996; Hudson et al. 2004) and SN Ia (Riess et al. 1995). Conflicting results triggered by the use of error-prone distance indicators have fueled a long lasting controversy on the amplitude and convergence of the bulk flow, that is still ongoing. For example, the recent claim of a bulk flow of $407\pm 81\mathrm {\ km\ s}^{-1}$ within $R=50\,\hbox {h}^{-1}\mathrm {\ Mpc}$ (Watkins et al. 2009), inconsistent with expectation from the $\varLambda$CDM model, has been seriously challenged by the re-analysis of the same data by Nusser and Davis (2011) who found a bulk flow amplitude consistent with $\varLambda$CDM expectations and from which they were able to set the strongest constraints on modified gravity models so far. On larger scales, Kashlinsky et al. (2010) claimed the detection of a dipole anisotropy attributed to the kinetic SZ decrement in the WMAP temperature map at the position of X-ray galaxy clusters. When interpreted as a coherent motion, this signal would indicate a gigantic bulk flow of $1028\pm 265\mathrm {\ km\ s}^{-1}$ within $R=528\,\hbox {h}^{-1}\mathrm {\ Mpc}$. This highly debated result has been seriously questioned by independent analyses of WMAP data (see, e.g., Osborne et al. 2011)

The large, homogeneous dataset expected from Euclid has the potential to settle these issues. The idea is to measure bulk flows in large redshift surveys, based on the apparent, dimming or brightening of galaxies due to their peculiar motion. The method, originally proposed by Tammann et al. (1979), has been recently extended by Nusser et al. (2011) who propose to estimate the bulk flow by minimizing systematic variations in galaxy luminosities with respect to a reference luminosity function measured from the whole survey. It turns out that, if applied to the photo-*z* catalog expected from Euclid, this method would be able to detect at $\> 5 \sigma$ significance a bulk flow like the one of Watkins et al. (2009) over $\sim \,50$ independent spherical volumes at $z \ge 0.2$, provided that the systematic magnitude offset over the corresponding areas in the sky does not exceed the expected random magnitude errors of 0.02–0.04 mag. Additionally, photo-*z* or spectral-*z* could be used to validate or disproof with very large ($> \,7 \sigma$) significance the claimed bulk flow detection of Kashlinsky et al. (2010) at $z=0.5$.

Closely related to the bulk flow is the local group peculiar velocity inferred from the observed CMB dipole (Juszkiewicz et al. 1990)

I.7.43 $$\begin{aligned} \mathbf {v}_{\mathrm {CMB}}=\mathbf {v}_{\mathrm {LG},R}-\frac{H_0f_g}{3}\mathbf {x}_{c.m.}+\mathbf {B}_{R} , \end{aligned}$$

where $\mathbf {v}_{\mathrm {LG},R}$ is the local group velocity resulting from the gravitational pull of all objects in the sample within the radius *R*, $\mathbf {x}_{\mathrm {c.m.}}$ is the position of the center of mass of the sample and $\mathbf {v}_{\mathrm {CMB}}$ is the LG velocity inferred from the CMB dipole (Bennett et al. 2003). The convergence of $\mathbf {v}_{\mathrm {LG},R}$ with the radius and its alignment with the CMB dipole direction indicates a crossover to homogeneity (Scaramella et al. 1991) and allows to constrain the growth rate by comparing $\mathbf {v}_{\mathrm {CMB}}$ with $\mathbf {v}_{\mathrm {LG},R}$. The latter can be estimated from the dipole in the distribution of objects either using a number-weighting scheme if redshifts are available for all objects of the sample, or using a flux-weighting scheme. In this second case the fact that both gravitational acceleration and flux are inversely proportional to the distance allows to compute the dipole from photometric catalogs with no need to measure redshifts. The drawback is that the information on the convergence scale is lost.

As for the bulk flow case, despite the many measurements of cosmological dipoles using galaxies (Yahil et al. 1980; Davis and Huchra 1982; Meiksin and Davis 1986; Strauss et al. 1992; Schmoldt et al. 1999; Kocevski and Ebeling 2006), there is still no general consensus on the scale of convergence and even on the convergence itself. Even the recent analyses of measuring the acceleration of the local group from the 2MASS redshift catalogs provided conflicting results. Erdoğdu et al. (2006) found that the galaxy dipole seems to converge beyond $R=60\,h^{-1}\mathrm {\ Mpc}$, whereas (Lavaux et al. 2010) find no convergence within $R=120\,h^{-1}\mathrm {\ Mpc}$.

Once again, Euclid will be in the position to solve this controversy by measuring the galaxy and cluster dipoles not only at the LG position and out to very large radii, but also in several independent ad truly all-sky spherical samples carved out from the the observed areas with $|b|>20^{\circ }$. In particular, coupling photometry with photo-*z* one expects to be able to estimate the convergence scale of the flux-weighted dipole over about 100 independent spheres of radius $200\,h^{-1}\mathrm {\ Mpc}$ out to $z=0.5$ and, beyond that, to compare number-weighted and flux-weighted dipoles over a larger number of similar volumes using spectroscopic redshifts.

Similarly, the growth rate can be constrained by studying the possibility of a Hubble bubble, a local region of space with a (slightly) different Hubble rate. This study was triggered by the fact that global observables such as Planck and BAO (Planck Collaboration 2014a, Table 5) yield a present-day Hubble constant 9% lower than local measurements performed by considering recession velocities of objects around us (Riess et al. 2011). This $2.4\sigma$ tension could be relieved if the effect of a local Hubble bubble is taken into account, see Marra et al. (2013a) and references therein. With Euclid one will of course use the data the other way around, using observations to constrain the Hubble bubbles (velocity monopoles) at different radii, and so the growth rate of matter structures, similarly to what discussed regarding the bulk flow.

#### Model independent observations

**Fig. 12:**
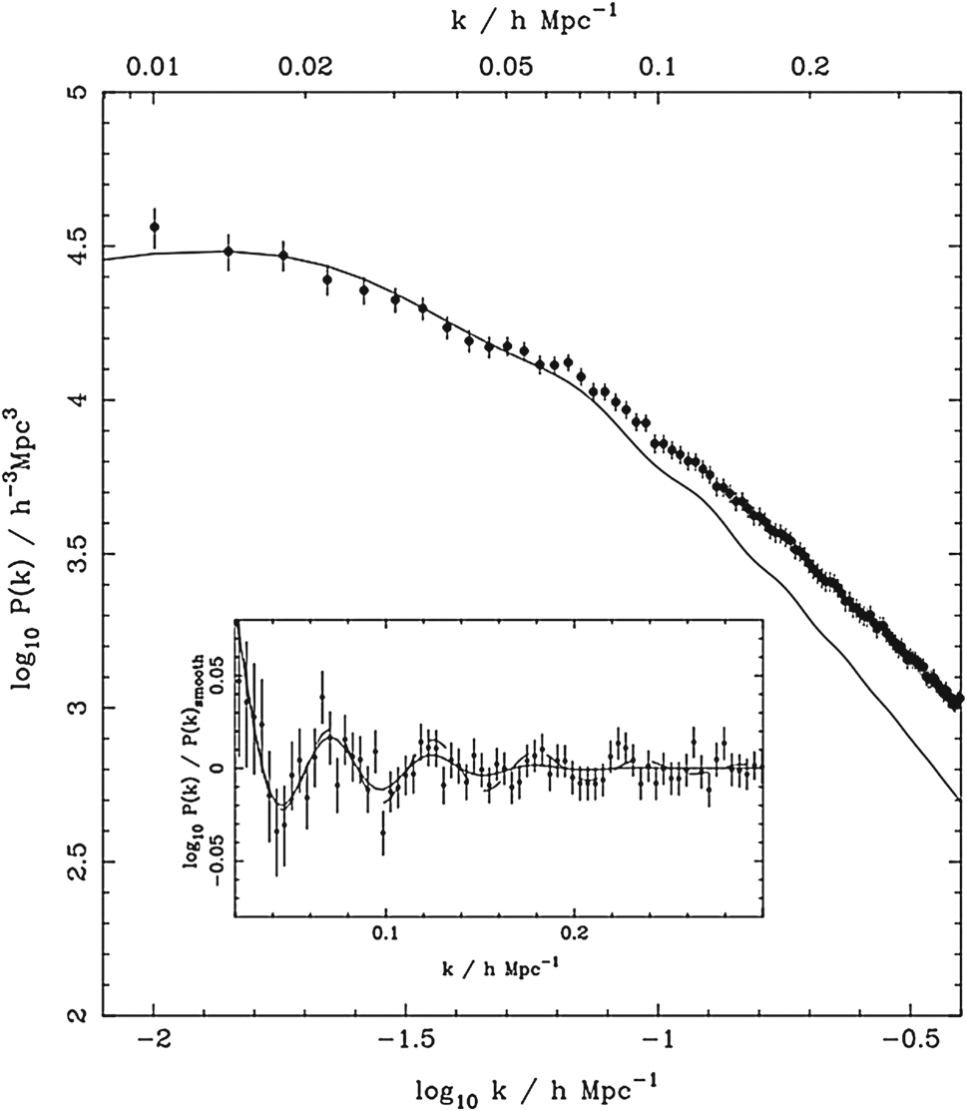
Matter power spectrum form measured from SDSS (Percival et al. 2007)

As discussed, one of the most powerful statistical tools that can be used to describe the distribution of matter in the Universe is the power spectrum *P*(*k*), or its Fourier transform $\xi (r)$, the two-point correlation function. However, comoving distances *r* and the corresponding wavenumbers *k* are not observationally accessible (Fig. 12).

When observing galaxies we measure their redshift and angular position. To convert this into a three-dimensional galaxy catalog we must make model assumptions in order to relate the observed redshift to a distance. For small redshift, $z\ll 1$, the simple relation $r(z)=z/H_0$ can be used. When expressing distances in units of $h^{-1}$ Mpc the uncertainty in the measurement of $H_0$ is then absorbed in $h=H_0/(100\,\hbox {Mpc/(km/s)}$. However, when $z\simeq 1$ the distance $r(z)=\chi (z)/H_0$ depends on the full cosmic expansion history, i.e., on the parameters $\varOmega _m$, $\varOmega _k$, $\varOmega _{DE}=1-\varOmega _m-\varOmega _k$ and $w_{DE}$, see Eq. (I.7.22), and wrong assumptions about the distance redshift relation will bias the entire catalog in a non-trivial way.

**Fig. 13:**
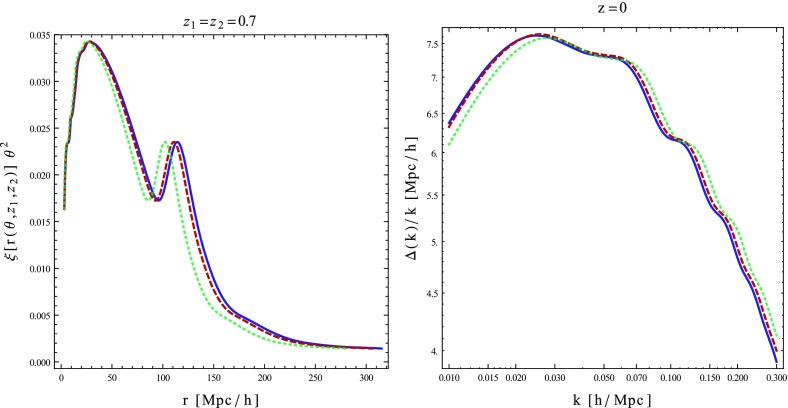
Effect of wrong cosmological parameters on the power spectrum. The true one (solid line) assumes the cosmological parameters of Komatsu et al. (2011) (in particular $\varOmega _\mathrm{m}=0.27$) and takes into account redshift space distortions. The wrong assumptions $\varOmega _{\mathrm{m}}=0.3, 0.5$ (dashed and dotted line, respectively) rescale the correlation function (on the left, multiplied by $\theta ^2$ to enhance the BAOs) and the dimensionless power spectrum (on the right, divided by *k* to enhance the BAOs)

Assuming an incorrect cosmology causes geometric redshift-distortions, in addition to the dynamical redshift distortions due to the peculiar velocities of galaxies. In Fig. 13 we show the effect of assuming a wrong cosmology. To illustrate this, we consider a $\varLambda$CDM universe with the cosmological parameters given in Komatsu et al. (2011). To reconstruct the comoving separation between two galaxies with redshift $z_1$ and $z_2$, respectively, and separated by the angle $\theta$, an observer must assume a model to reconstruct the relation $r(\theta ,z_1,z_2)$ which for vanishing curvature, is given by $r(z_1,z_2,\theta ) = \sqrt{r(z_1)^2 + r(z_2)^2 - 2r(z_1)r(z_2)\cos \theta }$, where the comoving distance $r(z) =\int _0^z dz'/H(z')$ depends on the cosmological model. Iterative methods are usually applied to converge to the correct cosmology. The inferred galaxy clustering in a different cosmological model can also be approximately obtained from the fiducial one by a rescaling of the transverse and parallel separations (Percival et al. 2010; Reid et al. 2012), so that an Alcock–Paczyński test (Alcock and Paczyński 1979) can be performed to select the best fit cosmological model. Nevertheless, these procedures rely on the assumption of a fiducial cosmology and are not very well suited to *measure* cosmological parameters, especially error estimates are not straight forward.

Together with the standard analysis, it is therefore important to determine the truly observed two-point statistics from observations, either in terms of the redshift dependent angular power spectra, $C_\ell (z_1,z_2)$, or in terms of the redshift dependent angular correlations functions $\xi (\theta ,z_1,z_2)$, which are related to the power spectra by Eq. (I.7.12), and to compare them directly with their theoretically obtained counterparts. The full expression for $C_\ell (z_1,z_2)$ are found in Bonvin and Durrer (2011), Asorey et al. (2012), Di Dio et al. (2013), see also Challinor and Lewis (2011), Yoo et al. (2009) and Yoo (2010). The angular correlation function has been studied in Montanari and Durrer (2012) and Bertacca et al. (2012), while the role of the lensing magnification, $\kappa$, which dominates the cross-correlations of different redshifts has been investigated in Montanari and Durrer (2015).

**Fig. 14:**
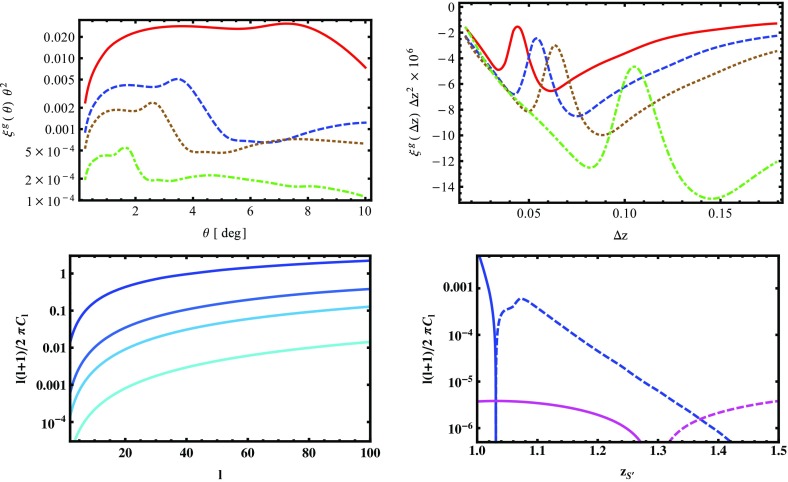
*Top panel:* transverse (on the left) and radial (on the right) correlation function at $z=0.3, 0.7, 1, 3$ from top to bottom, respectively. *Bottom panel:* the transverse power spectra at $z=0.1, 0.5, 1, 3$ from top to bottom, respectively (on the left), and the radial one for $\ell =20$ and $z_1=1$ as function of $z_2$ (on the right). The standard, non-relativistic terms in blue, the relativistic corrections from lensing in magenta. Images reproduced with permission from [top] (Montanari and Durrer 2012), and [bottom] from Bonvin and Durrer (2011), copyright by APS

The power spectra for $z_1=z_2 = 0.1,~0.5,~1$ and 3 are shown on Fig. 14, lower, left panel, and $C_{20}(1,z_2)$ is plotted as a function of $z_2$ in Fig. 14, lower, right panel.

These directly observed quantities are functions of three variables, $\theta , z_1$ and $z_2$. Therefore, they are harder to infer from observations than a function of only one variable. Especially, the shot noise problem is much more severe. However, they also contain more information. They combine in a non-trivial way clustering information given by $\xi (r)$ and geometrical information about the evolution of distances in the Universe via $r(\theta ,z_1,z_2)$. The Euclid galaxy survey will be sufficiently big to beat down the significant shot noise problem and profit maximally from this cleaner cut between observations and modeling, see Sect. I.8.11 for forecasts.

As an illustration of what can be done with this correlation function, we briefly consider the baryon acoustic oscillations (BAOs). The transverse BAOs at fixed redshift *z* in $\xi (\theta ,z,z)$ are shown in Fig. 14, top left panel.

The radial BAO, the correlation along the line of sight, $\xi (\theta =0,z-\varDelta z/2,z+\varDelta z/2)$ as a function of the redshift separation $\varDelta z$ of galaxy pairs around the reference value *z* are shown in Fig. 14, top right panel. To measure the radial correlation function, a spectroscopic determination of the redshift is required to resolve the BAO feature. The transverse and radial correlation functions can be used to determine the angular and redshift extension ($\theta _\mathrm{BAO}(z)$ and $\varDelta z_{\mathrm{BAO}}(z)$, respectively) of the BAOs as function of redshift, which determine

I.7.44 $$\begin{aligned} F(z)\equiv (1+z)H(z)D_A(z) = \frac{\varDelta z_\mathrm{BAO}(z)}{\theta _{\mathrm{BAO}}(z)} \equiv F^{AP}(z). \end{aligned}$$

where *H*(*z*) is the Hubble parameter and $D_A(z)$ is the angular diameter distance. Combining this with a measurement of the luminosity distance $D_L(z)=(1+z)^2D_A(z)$, e.g., from supernova type 1a data (Suzuki et al. 2012), we can break the degeneracy between *H*(*z*) and $D_A(z)$. This allows us to test the relation

$$\begin{aligned} D_A(z) = \frac{1}{z+1}\int _0^z\frac{dz'}{H\left( z'\right) } \quad \text{ or }\quad F(z) = \int _0^zdz'\frac{H(z)}{H\left( z'\right) } \end{aligned}$$

which must be valid, if the geometry of our Universe is close to a flat Friedmann–Lemaître metric.

### Forecasts for Euclid

Here^11^ we describe forecasts for the constraints on modified gravity parameters which Euclid observations should be able to achieve. We begin with reviewing the relevant works in literature. Then, after we define our “Euclid model”, i.e., the main specifics of the redshift and weak lensing survey, we illustrate a number of Euclid forecasts obtained through a Fisher matrix approach.

#### A review of forecasts for parametrized modified gravity with Euclid

Heavens et al. (2007) have used Bayesian evidence to distinguish between models, using the Fisher matrices for the parameters of interest. This study calculates the ratio of evidences *B* for a 3D weak lensing analysis of the full Euclid survey, for a dark-energy model with varying equation of state, and modified gravity with additionally varying growth parameter $\gamma$. They find that Euclid can decisively distinguish between, e.g., DGP and dark energy, with $|\ln B|\simeq 50$. In addition, they find that it will be possible to distinguish any departure from GR which has a difference in $\gamma$ greater than $\simeq 0.03$. A phenomenological extension of the DGP model (Dvali and Turner 2003; Afshordi et al. 2009) has also been tested with Euclid. Specifically, Camera et al. (2011a) found that it will be possible to discriminate between this modification to gravity from $\varLambda$CDM at the $3\sigma$ level in a wide range of angular scale, approximately $1000\lesssim \ell \lesssim 4000$.

**Fig. 15:**
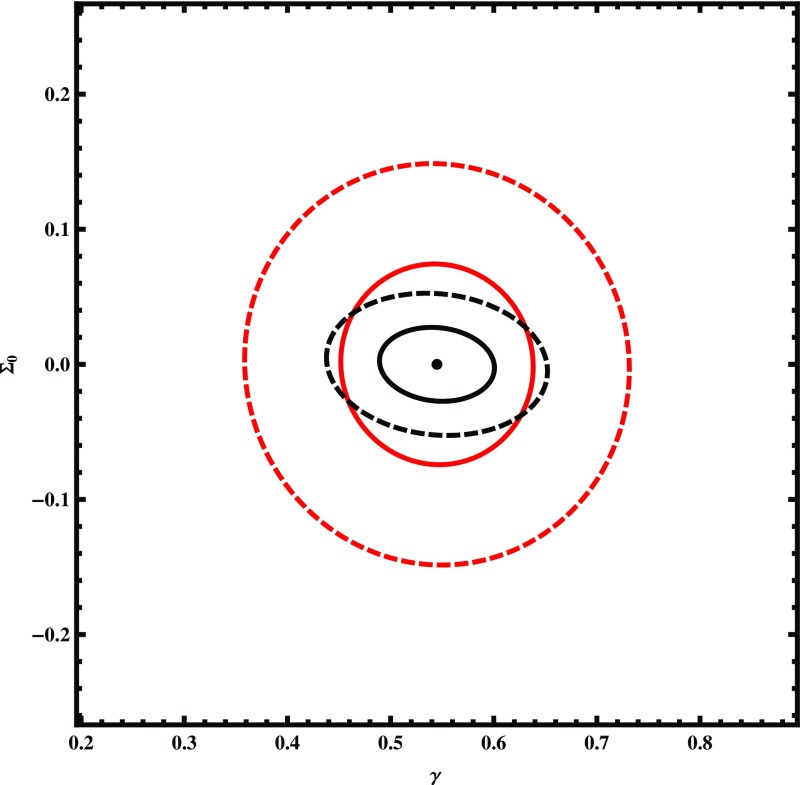
Marginalized $\gamma -\varSigma _0$ forecast for weak lensing only analysis with Euclid. Here, $\varSigma _0$ is defined from $\varSigma = 1+\varSigma _0 a$ and $\varSigma$, defined via Eq. (I.3.28), is related to the WL potential. Black contours correspond to $\ell _{\max }=5000$, demonstrating an error of 0.089$(1\sigma )$ on $\varSigma _0$, whereas the red contours correspond to $\ell _{\max }=500$ giving an error of 0.034. In both cases, the inner and outer contours are $1\sigma$ and $2\sigma$ respectively. GR resides at [0.55, 0], while DGP resides at [0.68, 0]

Thomas et al. (2009) construct Fisher matrix forecasts for the Euclid weak lensing survey, shown in Fig. 15. The constraints obtained depend on the maximum wavenumber which we are confident in using; $\ell _{\max }=500$ is relatively conservative as it probes the linear regime where we can hope to analytically track the growth of structure; $\ell _{\max }=10{,}000$ is more ambitious as it includes nonlinear power, using the Smith et al. (2003) fitting function. This will not be strictly correct, as the fitting function was determined in a GR context. Note that $\gamma$ is not very sensitive to $\ell _{\max }$, while $\varSigma _0$, defined in Amendola et al. (2008b) as $\varSigma = 1 + \varSigma _0 a$ [and where $\varSigma$ is defined in Eq. (I.3.28)] is measured much more accurately in the nonlinear regime.

Amendola et al. (2008b) find Euclid weak lensing constraints for a more general parameterization that includes evolution. In particular, $\varSigma (z)$ is investigated by dividing the Euclid weak lensing survey into three redshift bins with equal numbers of galaxies in each bin, and approximating that $\varSigma$ is constant within that bin. Since $\varSigma _1$, i.e., the value of $\varSigma$ in the $a=1$ bin (present-day) is degenerate with the amplitude of matter fluctuations, it is set to unity. The study finds that a deviation from unit $\varSigma$ (i.e., GR) of 3% can be detected in the second redshift bin, and a deviation of 10% is still detected in the furthest redshift bin.

Beynon et al. (2010) make forecasts for modified gravity with Euclid weak lensing including (Hu and Sawicki 2007b) in interpolating between the linear spectrum predicted by modified gravity, and GR on small scales as required by solar system tests. This requires parameters *A* (a measure of the abruptness of transitioning between these two regimes), $\alpha _1$ (controlling the *k*-dependence of the transition) and $\alpha _2$ (controlling the *z*-dependence of the transition).

**Fig. 16:**
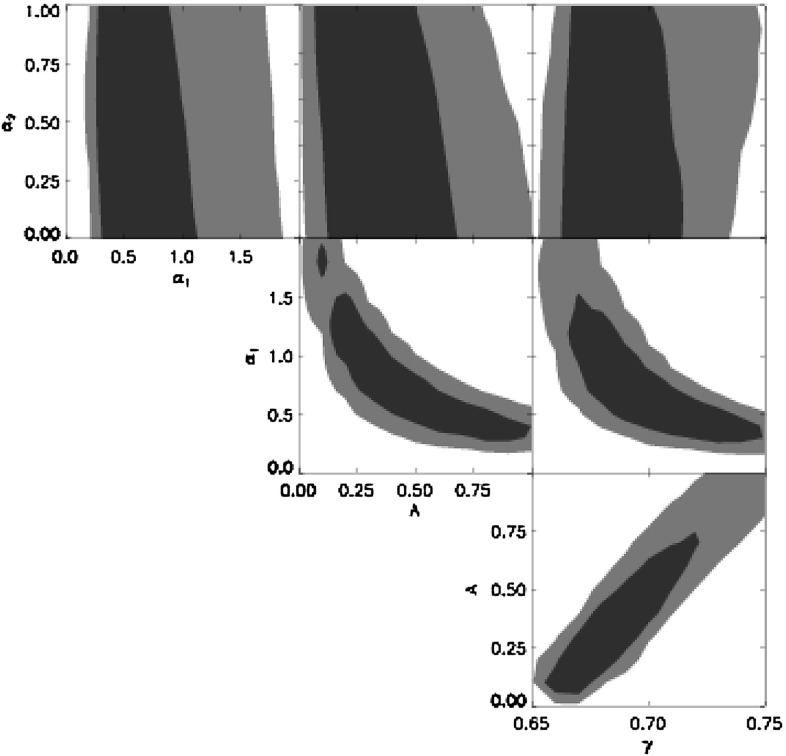
Constraints on $\gamma$, $\alpha _1$, $\alpha _2$ and *A* from Euclid, using a DGP fiducial model and 0.4 redshift bins between 0.3 and 1.5 for the central cosmological parameter values fitting $\hbox {WMAP} + \hbox {BAO} + \hbox {SNe}$

The forecasts for modified gravity parameters are shown in Fig. 16 for the Euclid lensing data. Even with this larger range of parameters to fit, Euclid provides a measurement of the growth factor $\gamma$ to within 10%, and also allows some constraint on the $\alpha _1$ parameter, probing the physics of nonlinear collapse in the modified gravity model.

Finally, Song et al. (2010) have shown forecasts for measuring $\varSigma$ and $\mu$ using both imaging and spectroscopic surveys. They combine 15,000 square-degree lensing data (corresponding to Laureijs et al. 2009 rather than to the updated Laureijs et al. 2011) with the peculiar velocity dispersion measured from redshift space distortions in the spectroscopic survey, together with stringent background expansion measurements from the CMB and supernovae. They find that for simple models for the redshift evolution of $\varSigma$ and $\mu$, both quantities can be measured to 20% accuracy.

#### Euclid surveys

The Euclid mission will produce a catalog of up to 30 million galaxy redshifts with $f_{H_\alpha } > 3 \times 10^{-16}$ and 50 million with $f_{H_\alpha } > 2 \times 10^{-16}$ and an imaging survey that should allow to estimate the galaxy ellipticity of up to 1.5 billion galaxy images with photometric redshifts. Here we discuss these surveys and fix their main properties into a “Euclid model”, i.e., an approximation to the real Euclid survey that will be used as reference mission in the following.

**Modeling the Redshift Survey.**

The main goals of next generation redshift surveys will be to constrain the dark-energy parameters and to explore models alternative to standard Einstein gravity. For these purposes they will need to consider very large volumes that encompass $z\sim 1$, i.e., the epoch at which dark energy started dominating the energy budget, spanning a range of epochs large enough to provide a sufficient leverage to discriminate among competing models at different redshifts.

Here we consider a survey covering a large fraction of the extragalactic corresponding to $\sim \,15{,}000\mathrm {\ deg}^2$ capable to measure a large number of galaxy redshifts out to $z\sim 2$. A promising observational strategy is to target H$\alpha$ emitters at near-infrared wavelengths (which implies $z>0.5$) since they guarantee both relatively dense sampling (the space density of this population is expected to increase out to $z\sim 2$) and an efficient method to measure the redshift of the object. The limiting flux of the survey should be the tradeoff between the requirement of minimizing the shot noise, the contamination by other lines (chiefly among them the [O ii] line), and that of maximizing the so-called efficiency $\varepsilon$, i.e., the fraction of successfully measured redshifts. To minimize shot noise one should obviously strive for a low flux. Indeed, Geach et al. (2010) found that a limiting flux $f_{\mathrm {H}\alpha } \ge 1\times 10^{-16}\mathrm {\ erg\ cm^{-2}\ s^{-1}}$ would be able to balance shot noise and cosmic variance out to $z=1.5$. However, simulated observations of mock H$\alpha$ galaxy spectra have shown that $\varepsilon$ ranges between 30 and 60% (depending on the redshift) for a limiting flux $f_{\mathrm {H}\alpha }\ge 3\times 10^{-16}\mathrm {\ erg\ cm^{-2}\ s^{-1}}$ (Laureijs et al. 2011). Moreover, contamination from [O ii] line drops from 12 to 1% when the limiting flux increases from $1\times 10^{-16}$ to $5\times 10^{-16}\mathrm {\ erg\ cm^{-2}\ s^{-1}}$ (Geach et al. 2010).

Taking all this into account, in order to reach the top-level science requirement on the number density of H$\alpha$ galaxies, the average effective H$\alpha$ line flux limit from a 1-arcsec diameter source shall be lower than or equal to $3\times 10^{-16}\mathrm {\ erg\ cm^{-2}\ s^{-1}}$. However, a slitless spectroscopic survey has a success rate in measuring redshifts that is a function of the emission line flux. As such, the Euclid survey cannot be characterized by a single flux limit, as in conventional slit spectroscopy.

We use the number density of H$\alpha$ galaxies at a given redshift, *n*(*z*), estimated using the latest empirical data (see Figure 3.2 of Laureijs et al. 2011), where the values account for redshift—and flux—success rate, to which we refer as our reference efficiency $\varepsilon _r$.

However, in an attempt to bracket current uncertainties in modeling galaxy surveys, we consider two further scenarios, one where the efficiency is only the half of $\varepsilon _r$ and one where it is increased by a factor of 40%. Then we define the following cases:

- *Reference case* (*ref.*). Galaxy number density *n*(*z*) which include efficiency $\varepsilon _r$ (column $n_2(z)$ in Table 3).
- *Pessimistic case* (*pess.*). Galaxy number density $n(z)\cdot 0.5$, i.e., efficiency is $\varepsilon _{r}\cdot 0.5$ (column $n_3(z)$ in Table 3).
- *Optimistic case* (*opt.*). Galaxy number density $n(z)\cdot 1.4$, i.e., efficiency is $\varepsilon _{r}\cdot 1.4$ (column $n_1(z)$ in Table 3).

The total number of observed galaxies ranges from $2\times 10^7$ (pess.) to $5\times 10^7$ (opt.) with a central value at $3\times 10^7$ . For all cases we assume that the error on the measured redshift is $\varDelta z=0.001(1+z)$, independent of the limiting flux of the survey.

**Table 3:** Expected galaxy number densities in units of $(h/\mathrm {Mpc})^{3}$ for Euclid survey

*z*	$n_{1}(z) \times 10^{-3}$	$n_{2}(z)\times 10^{-3}$	$n_{3}(z) \times 10^{-3}$
0.65$\textendash$0.75	1.75	1.25	0.63
0.75$\textendash$0.85	2.68	1.92	0.96
0.85$\textendash$0.95	2.56	1.83	0.91
0.95$\textendash$1.05	2.35	1.68	0.84
1.05$\textendash$1.15	2.12	1.51	0.76
1.15$\textendash$1.25	1.88	1.35	0.67
1.25$\textendash$1.35	1.68	1.20	0.60
1.35$\textendash$1.45	1.40	1.00	0.50
1.45$\textendash$1.55	1.12	0.80	0.40
1.55$\textendash$1.65	0.81	0.58	0.29
1.65$\textendash$1.75	0.53	0.38	0.19
1.75$\textendash$1.85	0.49	0.35	0.18
1.85$\textendash$1.95	0.29	0.21	0.10
1.95$\textendash$2.05	0.16	0.11	0.06

Let us notice that the galaxy number densities *n*(*z*) depend on the fiducial cosmology adopted in the computation of the survey volume, needed for the conversion from the galaxy numbers *dN* / *dz* to *n*(*z*)

**Modeling the weak lensing survey.** For the weak lensing survey, we assume again a sky coverage of 15,000 square degrees. For the number density we use the common parameterization

I.8.1 $$\begin{aligned} n(z) = z^2 \exp \left( -\left( z/z_0\right) ^{3/2}\right) , \end{aligned}$$

where $z_0 =z_{\mathrm {mean}}/1.412$ is the peak of *n*(*z*) and $z_{\mathrm {mean}}$ the median and typically we assume $z_{\mathrm {mean}}=0.9$ and a surface density of valid images of $n_g=30$ per arcmin$^2$ (Laureijs et al. 2011). We also assume that the photometric redshifts give an error of $\varDelta z=0.05(1+z)$. Other specifications will be presented in the relevant sections.

#### Forecasts for the growth rate from the redshift survey

In this section, we forecast the constraints that future observations can put on the growth rate and on a scale-independent bias, employing the Fisher matrix method presented in Sect. I.7.3. We use the representative Euclid survey presented in Sect. I.8.2. We assess how well one can constrain the bias function from the analysis of the power spectrum itself and evaluate the impact that treating bias as a free parameter has on the estimates of the growth factor. We estimate how errors depend on the parametrization of the growth factor and on the number and type of degrees of freedom in the analysis. Finally, we explicitly explore the case of coupling between dark energy and dark matter and assess the ability of measuring the coupling constant. Our parametrization is defined as follows. More details can be found in Di Porto et al. (2012).

Equation of state

In order to represent the evolution of the equation of state parameter *w*, we use the popular CPL parameterization (Chevallier and Polarski 2001; Linder 2003)

I.8.2 $$\begin{aligned} w(z)=w_{0}+w_{1}\frac{z}{1+z}. \end{aligned}$$

As a special case, we will also consider the case of a constant *w*. We refer to this as the *w*-parametrization.

Growth rate

Here, we assume that the growth rate, $f_g$, is a function of time but not of scale. As usual, we use the simple prescription (Peebles 1976; Lahav et al. 1991; Polarski and Gannouji 2008; Linder 2005; Wang and Steinhardt 1998)

I.8.3 $$\begin{aligned} f_g=\varOmega _{m}^{\gamma }, \end{aligned}$$

where $\varOmega _{m}(z)$ is the matter density in units of the critical density as a function of redshift. A value $\gamma \approx 0.545$ reproduces well the $\varLambda$CDM behavior while departures from this value characterize different models. Here we explore three different parameterizations of $f_g$:

- *f*-*parameterization*. This is in fact a non-parametric model in which the growth rate itself is modeled as a step-wise function $f_g(z)=f_{i}$, specified in different redshift bins. The errors are derived on $f_{i}$ in each *i*-th redshift bin of the survey.
- $\gamma$-*parameterization*. As a second case we assume
  I.8.4 $$\begin{aligned} f_g\equiv \varOmega _{m}(z)^{\gamma (z)}. \end{aligned}$$
  where the $\gamma (z)$ function is parametrized as
  I.8.5 $$\begin{aligned} \gamma (z)=\gamma _{0}+\gamma _{1}\frac{z}{1+z}. \end{aligned}$$
  As shown by Wu et al. (2009) and Fu et al. (2009), this parameterization is more accurate than that of Eq. (I.8.3) for both $\varLambda$CDM and DGP models. Furthermore, this parameterization is especially effective to distinguish between a *w*CDM model (i.e., a dark-energy model with a constant equation of state) that has a negative $\gamma _{1}$ ($-0.020\lesssim \gamma _{1}\lesssim -0.016$) and a DGP model that instead, has a positive $\gamma _{1}$ ($0.035<\gamma _{1}<0.042$). In addition, modified gravity models show a strongly evolving $\gamma (z)$ (Gannouji et al. 2009; Motohashi et al. 2010a; Fu et al. 2009), in contrast with conventional dark-energy models. As a special case we also consider $\gamma =$ constant (only when *w* also is assumed constant), to compare our results with those of previous works.
- $\eta$-*parameterization*. To explore models in which perturbations grow faster than in the $\varLambda$CDM case, like in the case of a coupling between dark energy and dark matter (Di Porto and Amendola 2008), we consider a model in which $\gamma$ is constant and the growth rate varies as
  I.8.6 $$\begin{aligned} f_g\equiv \varOmega _{m}(z)^{\gamma }(1+\eta ), \end{aligned}$$
  where $\eta$ quantifies the strength of the coupling. The example of the coupled quintessence model worked out by Di Porto and Amendola (2008) illustrates this point. In that model, the numerical solution for the growth rate can be fitted by the formula (I.8.6), with $\eta =c\beta _{c}^{2}$, where $\beta _{c}$ is the dark energy-dark matter coupling constant and best fit values $\gamma =0.56$ and $c=2.1$. In this simple case, observational constraints over $\eta$ can be readily transformed into constraints over $\beta _{c}$.

Reference cosmological models

We assume as reference model a “pseudo” $\varLambda$CDM, where the growth rate values are obtained from Eq. (I.8.3) with $\gamma =0.545$ and $\varOmega _m(z)$ is given by the standard evolution. Then $\varOmega _m(z)$ is completely specified by setting $\varOmega _{m,0}=0.271$, $\varOmega _k=0$, $w_0=-\,0.95$, $w_1=0$. When the corresponding parameterizations are employed, we choose as fiducial values $\gamma _{1}=0$ and $\eta =0$, We also assume a primordial slope $n_s=0.966$ and a present normalization $\sigma _8=0.809$.

One of the goals of this work is to assess whether the analysis of the power spectrum in redshift-space can distinguish the fiducial model from alternative cosmologies, characterized by their own set of parameters (apart from $\varOmega _{m,0}$ which is set equal to 0.27 for all of them). The alternative models that we consider in this work are:

- *DGP model*. We consider the flat space case studied in Maartens and Majerotto (2006). When we adopt this model then we set $\gamma _{0}=0.663$, $\gamma _{1}=0.041$ (Fu et al. 2009) or $\gamma =0.68$ (Linder and Cahn 2007) and $w=-\,0.8$ when $\gamma$ and *w* are assumed constant.
- *f*(*R*) *model*. Here we consider different classes of *f*(*R*) models: (i) the one proposed in Hu and Sawicki (2007a), depending on two parameters, *n* and $\mu$ in Eq. (I.5.36), which we fix to $n=0.5,1,2$ and $\mu =3$. For the model with $n=2$ we assume $\gamma _{0}=0.43$, $\gamma _{1}=-\,0.2$, values that apply quite generally in the limit of small scales [provided they are still linear, see Gannouji et al. (2009)] or $\gamma =0.4$ and $w=-\,0.99$. Unless differently specified, we will always refer to this specific model when we mention comparisons to a single *f*(*R*) model. (ii) The model of Eq. (I.5.37), proposed in Starobinsky (2007), where we fix $\mu =3$ and $n=2$, which shows a very similar behavior to the previous one. (iii) The one proposed in Tsujikawa (2008), Eq. (I.5.38), fixing $\mu =1$.
- *Coupled dark-energy (CDE) model*. This is the coupled model proposed by Amendola (2000a) and Wetterich (1995). In this case we assume $\gamma _{0}=0.56$, $\eta =0.056$ (this value comes from putting $\beta _{c}=0.16$ as coupling, which is of the order of the maximal value allowed by CMB constraints) (Amendola and Quercellini 2003). As already explained, this model cannot be reproduced by a constant $\gamma$. Forecasts on coupled quintessence based on Amendola et al. (2011), Amendola (2000a), Pettorino and Baccigalupi (2008) are discussed in more detail in Sect. I.8.8.

For the fiducial values of the bias parameters in every bin, we assume $b(z)=\sqrt{1+z}$ (already used in Rassat et al. 2008) since this function provides a good fit to H${\alpha }$ line galaxies with luminosity $L_{\mathrm {H}\alpha }=10^{42}\mathrm {\ erg^{-1}\ s^{-1}\ h^{-2}}$ modeled by Orsi et al. (2010) using the semi-analytic *GALFORM* models of Baugh et al. (2005). For the sake of comparison, we will also consider the case of constant $b=1$ corresponding to the rather unphysical case of a redshift-independent population of unbiased mass tracers.

The fiducial values for $\beta$ are computed through

I.8.7 $$\begin{aligned} \beta ^F(z) = \frac{\varOmega _m^F(z)^{\gamma ^F}}{b^F(z)}=\frac{f_g^F}{b^F}. \end{aligned}$$

Now we express the growth function *G*(*z*) and the redshift distortion parameter $\beta (z)$ in terms of the growth rate $f_g$ [see Eqs. (I.8.8), (I.8.7)]. When we compute the derivatives of the spectrum in the Fisher matrix *b*(*z*) and $f_g(z)$ are considered as independent parameters in each redshift bin. In this way we can compute the errors on *b* (and $f_g$) self consistently by marginalizing over all other parameters.

Now we are ready to present the main result of the Fisher matrix analysis. We note that in all tables below we always quote errors at 68% probability level and draw in the plots the probability regions at 68 and/or 95% (denoted for shortness as 1 and 2$\sigma$ values). Moreover, in all figures, all the parameters that are not shown have been marginalized over or fixed to a fiducial value when so indicated.

Results for the *f*-parameterization

The total number of parameters that enter in the Fisher matrix analysis is 45: 5 parameters that describe the background cosmology ($\varOmega _{m,0}h^{2},\varOmega _{b,0}h^{2},$ *h*, *n*, $\varOmega _{k}$) plus 5 *z*-dependent parameters specified in 8 redshift bins evenly spaced in the range $z=[0.5,2.1]$. They are $P_{\text {s}}(z)$, *D*(*z*), *H*(*z*), $f_g(z)$, *b*(*z*). However, since we are not interested in constraining *D*(*z*) and *H*(*z*), we always project them to the set of parameters they depend on (as explained in Seo and Eisenstein 2003) instead of marginalizing over, so extracting more information on the background parameters.

The fiducial growth function *G*(*z*) in the $(i+1)$-th redshift bin is evaluated from a step-wise, constant growth rate $f_g(z)$ as

I.8.8 $$\begin{aligned} G(z)=\exp \left\{ -\int _{0}^{z}f_g(z)\frac{dz}{1+z}\right\} = \prod _{i}\left( \frac{1+z_{i}}{1+z_{i-1}}\right) ^{-f_{i}}\left( \frac{1+z}{1+z_{i}}\right) ^{-f_{i+1}} . \end{aligned}$$

**Fig. 17:**
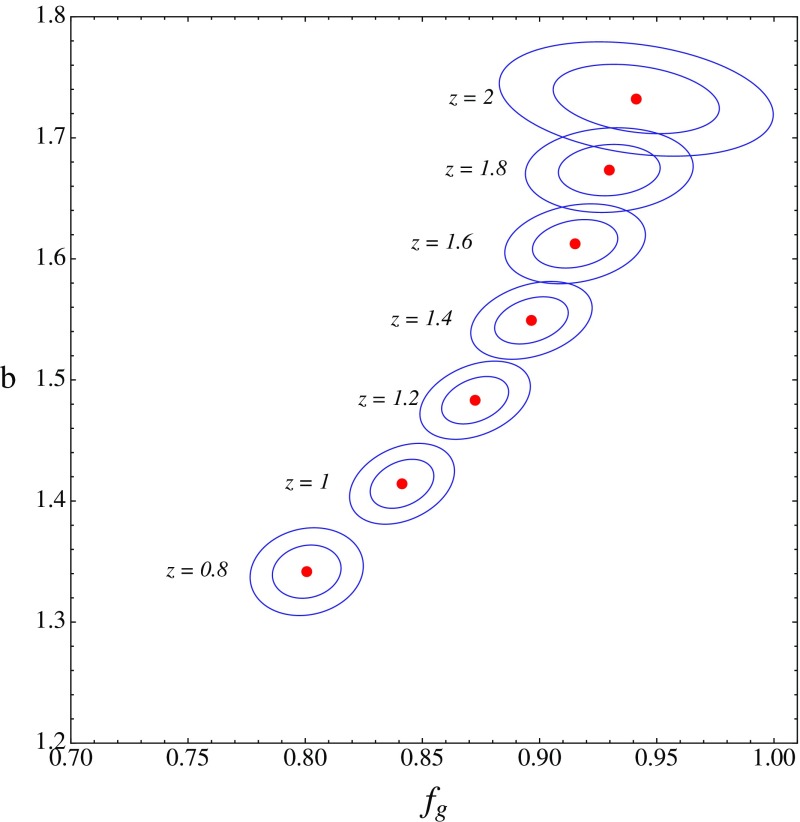
Contour plots at 68 and 98% of probability for the pairs $s(z_i)-b(z_i)$ in 7 redshift bins (with $b=\sqrt{1+z}$). The ellipses are centered on the fiducial values of the growth rate and bias parameters, computed in the central values of the bins, $z_{i}$

To obtain the errors on $s_{i}$ and $b_{i}$ we compute the elements of the Fisher matrix and marginalize over all other parameters. In this case one is able to obtain, self-consistently, the error on the bias and on the growth factor at different redshifts, as detailed in Table 4. In Fig. 17, we show the contour plots at 68 and 95% of probability for all the pairs $s(z_i)-b(z_i)$ in several redshift bins (with $b=\sqrt{1+z}$), where $z_{i}$’s are the central values of the bins. We do not show the ellipses for all the 14 bins to avoid overcrowding.

**Table 4:** $1\sigma$ marginalized errors for the bias and the growth rates in each redshift bin

z	$\sigma _{b}$			$b^F$	*z*	$f_g^F$	$\sigma _{f_g}$		
	Ref.	Opt.	Pess.				Ref.	Opt.	Pess.
0.7	0.016	0.015	0.019	1.30	0.7	0.76	0.011	0.010	0.012
0.8	0.014	0.014	0.017	1.34	0.8	0.80	0.010	0.009	0.011
0.9	0.014	0.013	0.017	1.38	0.9	0.82	0.009	0.009	0.011
1.0	0.013	0.012	0.016	1.41	1.0	0.84	0.009	0.008	0.011
1.1	0.013	0.012	0.016	1.45	1.1	0.86	0.009	0.008	0.011
1.2	0.013	0.012	0.016	1.48	1.2	0.87	0.009	0.009	0.011
1.3	0.013	0.012	0.016	1.52	1.3	0.88	0.010	0.009	0.012
1.4	0.013	0.012	0.016	1.55	1.4	0.89	0.010	0.009	0.013
1.5	0.013	0.012	0.016	1.58	1.5	0.91	0.011	0.010	0.014
1.6	0.013	0.012	0.016	1.61	1.6	0.91	0.012	0.011	0.016
1.7	0.014	0.013	0.017	1.64	1.7	0.92	0.014	0.012	0.018
1.8	0.014	0.013	0.018	1.67	1.8	0.93	0.014	0.013	0.019
1.9	0.016	0.014	0.021	1.70	1.9	0.93	0.017	0.015	0.025
2.0	0.019	0.016	0.028	1.73	2.0	0.94	0.023	0.019	0.037

Table 4 illustrates one important result: through the analysis of the redshift-space galaxy power spectrum in a next-generation Euclid-like survey, it will be possible to measure galaxy biasing in $\varDelta z=0.1$ redshift bins with less than 1.6% error, provided that the bias function is independent of scale. We also tested a different choice for the fiducial form of the bias: $b(z)=1$ finding that the precision in measuring the bias as well as the other parameters has a very little dependence on the *b*(*z*) form. Given the robustness of the results on the choice of *b*(*z*) in the following we only consider the $b(z)=\sqrt{1+z}$ case.

**Fig. 18:**
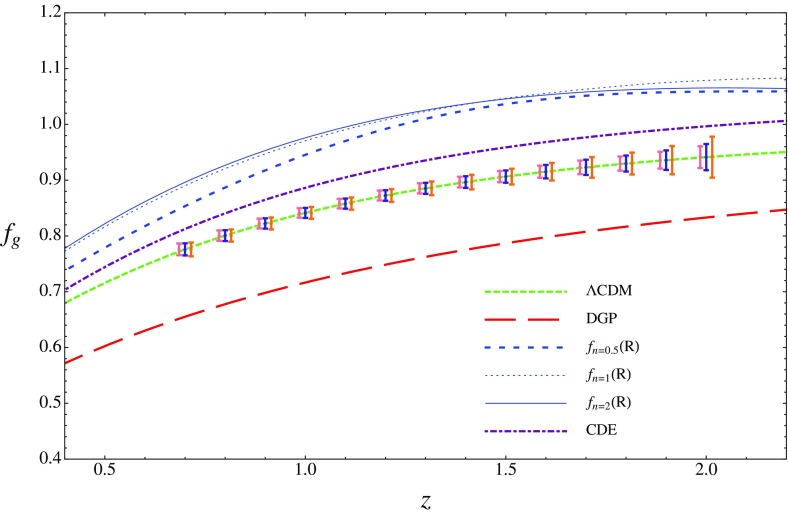
Expected constraints on the growth rates in each redshift bin. For each *z* the central error bars refer to the *Reference case* while those referring to the *Optimistic* and * Pessimistic* case have been shifted by $-0.015$ and $+0.015$ respectively. The growth rates for different models are also plotted: $\varLambda$CDM (green tight shortdashed curve), flat DGP (red longdashed curve) and a model with coupling between dark energy and dark matter (purple, dot-dashed curve). The blue curves (shortdashed, dotted and solid) represent the *f*(*R*) model by Hu and Sawicki (2007a), Eq. (I.5.36) with $n=0.5,1,2$ respectively and $\mu =3$. The plot shows that it will be possible to distinguish these models with next generation data

**Fig. 19:**
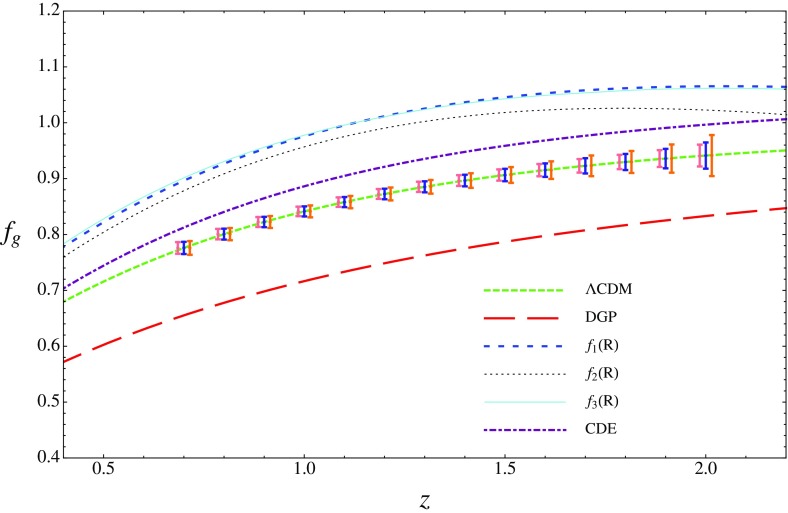
Expected constraints on the growth rates in each redshift bin. For each *z* the central error bars refer to the *Reference case* while those referring to the *Optimistic* and *Pessimistic* case have been shifted by $-\,0.015$ and $+\,0.015$ respectively. The growth rates for different models are also plotted: $\varLambda$CDM (green tight shortdashed curve), flat DGP (red longdashed curve) and a model with coupling between dark energy and dark matter (purple, dot-dashed curve). Here we plot again the *f*(*R*) model by Hu and Sawicki (2007a), Eq. (I.5.36), with $n=2$ and $\mu =3$ (blue shortdashed curve) together with the model by Starobinsky (2007), Eq. (I.5.37), with $n=2$ and $\mu =3$ (cyan solid curve) and the one by Tsujikawa (2008), Eq. (I.5.38), with $\mu =1$ (black dotted curve). Also in this case it will be possible to distinguish these models with next generation data

In Figs. 18 and 19, we show the errors on the growth rate $f_g$ as a function of redshift, overplotted to our fiducial $\varLambda$CDM (green solid curve). The three sets of error bars are plotted in correspondence of the 14 redshift bins and refer (from left to right) to the *Optimistic, Reference* and *Pessimistic* cases, respectively. The other curves show the expected growth rate in three alternative cosmological models: flat DGP (red, longdashed curve), CDE (purple, dot-dashed curve) and different *f*(*R*) models (see description in the figure captions). This plot clearly illustrates the ability of next generation surveys to distinguish between alternative models, even in the less favorable choice of survey parameters.

The main results can be summarized as follows.

1. The ability of measuring the biasing function is not too sensitive to the characteristic of the survey (*b*(*z*) can be constrained to within 1% in the *Optimistic* scenario and up to 1.6% in the *Pessimistic* one) provided that the bias function is independent of scale. Moreover, we checked that the precision in measuring the bias has a very little dependence on the *b*(*z*) form.
2. The growth rate $f_g$ can be estimated to within 1–2.5% in each bin for the *Reference case* survey with no need of estimating the bias function *b*(*z*) from some dedicated, independent analysis using higher order statistics (Verde et al. 2002) or full-PDF analysis (Sigad et al. 2000).
3. The estimated errors on $f_g$ depend weakly on the fiducial model of *b*(*z*).

Next, we focus on the ability of determining $\gamma _0$ and $\gamma _1$, in the context of the $\gamma$-*parameterization* and $\gamma$, $\eta$ in the $\eta$-*parameterization*. In both cases the Fisher matrix elements have been estimated by expressing the growth factor as

I.8.9 $$\begin{aligned} G(z)= & {} \delta _{0}\exp \left[ (1+\eta )\int _{0}^{z}\varOmega _{m} \left( z^{\prime }\right) ^{\gamma (z)}\frac{ dz^{\prime }}{1+z^{\prime }}\right] , \end{aligned}$$

where for the $\gamma$-*parameterization* we fix $\eta =0$.

**Fig. 20:**
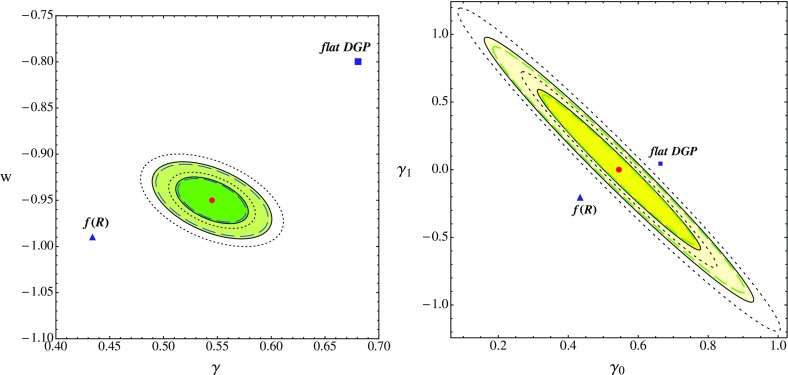
$\gamma$-parameterization. Left panel: 1 and 2$\sigma$ marginalized probability regions for constant $\gamma$ and *w*: the green (shaded) regions are relative to the *Reference case*, the blue long-dashed ellipses to the *Optimistic case*, while the black short-dashed ellipses are the probability regions for the *Pessimistic case*. The red dot marks the fiducial model; two alternative models are also indicated for comparison. Right panel: 1 and 2$\sigma$ marginalized probability regions for the parameters $\gamma _{0}$ and $\gamma _{1}$, relative to the *Reference case* (shaded yellow regions), to the *Optimistic case* (green long-dashed ellipses), and to the *Pessimistic case* (black dotted ellipses). Red dots represent the fiducial model, blue squares mark the DGP while triangles stand for the *f*(*R*) model. Then, in the case of $\gamma$-parameterization, one could distinguish these three models (at 95% probability)

**Fig. 21:**
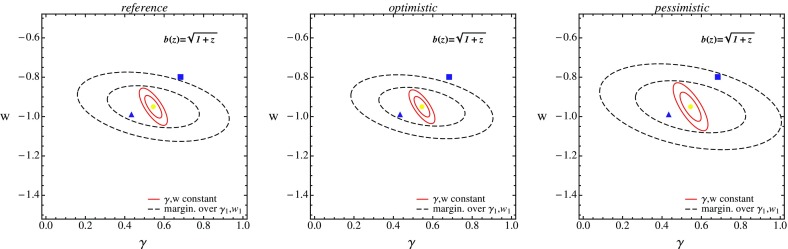
$\gamma$-parameterization. 1 and $2\sigma$ marginalized probability regions obtained assuming constant $\gamma$ and *w* (red solid curves) or assuming the parameterizations (I.8.5) and (I.8.2) and marginalizing over $\gamma _{1}$ and $w_{1}$ (black dashed curves); marginalized error values are in columns $\sigma _{{\gamma }_{\text {marg},1}}$, $\sigma _{{w}_{\text {marg},1}}$ of Table 8. Yellow dots represent the fiducial model, the triangles a *f*(*R*) model and the squares mark the flat DGP

**Fig. 22:**
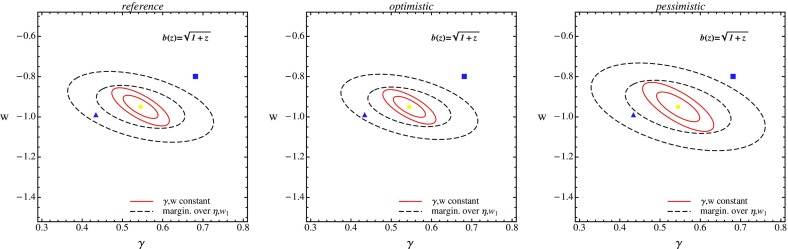
$\eta$-parameterization. 1 and 2$\sigma$ marginalized probability regions obtained assuming constant $\gamma$ and *w* (red solid curves) or assuming the parameterizations (I.8.6) and (I.8.2) and marginalizing over $\eta$ and $w_{1}$ (black dashed curves); marginalized error values are in columns $\sigma _{{\gamma }_{\text {marg},2}}$, $\sigma _{{w}_{\text {marg},2}}$ of Table 9. Yellow dots represent the fiducial model, the triangles stand for a *f*(*R*) model and the squares mark the flat DGP

**Fig. 23:**
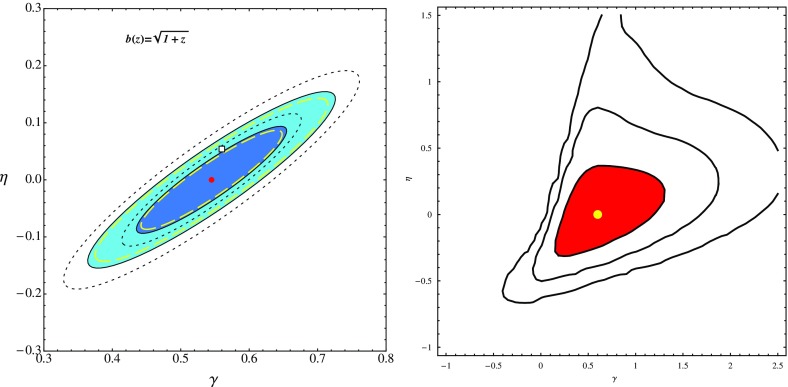
$\eta$-parameterization. Left panel: 1 and 2$\sigma$ marginalized probability regions for the parameters $\gamma$ and $\eta$ in Eq. (I.8.6) relative to the reference case (shaded blue regions), to the optimistic case (yellow long-dashed ellipses) and to the pessimistic case (black short-dashed ellipses). The red dot marks the fiducial model while the square represents the coupling model. Right panel: present constraints on $\gamma$ and $\eta$ computed through a full likelihood method (here the red dot marks the likelihood peak) (Di Porto and Amendola 2008)

- $\gamma$-*parameterization*. We start by considering the case of constant $\gamma$ and *w* in which we set $\gamma =\gamma ^F=0.545$ and $w=w^F=-\,0.95$. As we will discuss in the next Section, this simple case will allow us to cross-check our results with those in the literature. In Fig. 20, we show the marginalized probability regions, at 1 and $2\sigma$ levels, for $\gamma$ and *w*. The regions with different shades of green illustrate the *Reference case* for the survey whereas the blue long-dashed and the black short-dashed ellipses refer to the *Optimistic* and *Pessimistic* cases, respectively. Errors on $\gamma$ and *w* are listed in Table 5 together with the corresponding figures of merit [FoM] defined to be the squared inverse of the Fisher matrix determinant and therefore equal to the inverse of the product of the errors in the pivot point, see Albrecht et al. (2006). Contours are centered on the fiducial model. The blue triangle and the blue square represent the flat DGP and the *f*(*R*) models’ predictions, respectively. It is clear that, in the case of constant $\gamma$ and *w*, the measurement of the growth rate in a Euclid-like survey will allow us to discriminate among these models. These results have been obtained by fixing the curvature to its fiducial value $\varOmega _k=0$. If instead, we consider curvature as a free parameter and marginalize over, the errors on $\gamma$ and *w* increase significantly, as shown in Table 6, and yet the precision is good enough to distinguish the different models. For completeness, we also computed the fully marginalized errors over the other cosmological parameters for the reference survey, given in Table 7.
  As a second step we considered the case in which $\gamma$ and *w* evolve with redshift according to Eqs. (I.8.5) and (I.8.2) and then we marginalized over the parameters $\gamma _{1}$, $w_{1}$ and $\varOmega _k$. The marginalized probability contours are shown in Fig. 21 in which we have shown the three survey setups in three different panels to avoid overcrowding. Dashed contours refer to the *z*-dependent parameterizations while red, continuous contours refer to the case of constant $\gamma$ and *w* obtained after marginalizing over $\varOmega _k$. Allowing for time dependency increases the size of the confidence ellipses since the Fisher matrix analysis now accounts for the additional uncertainties in the extra-parameters $\gamma _{1}$ and $w_{1}$; marginalized error values are in columns $\sigma _{{\gamma }_{\text {marg},1}}$, $\sigma _{{w}_{\text {marg},1}}$ of Table 8. The uncertainty ellipses are now larger and show that DGP and fiducial models could be distinguished at $>\,2\sigma$ level only if the redshift survey parameter will be more favorable than in the *Reference case*.
  We have also projected the marginalized ellipses for the parameters $\gamma _{0}$ and $\gamma _{1}$ and calculated their marginalized errors and figures of merit, which are reported in Table 9. The corresponding uncertainties contours are shown in the right panel of Fig. 20. Once again we overplot the expected values in the *f*(*R*) and DGP scenarios to stress the fact that one is expected to be able to distinguish among competing models, irrespective on the survey’s precise characteristics.
- $$\eta $$-*parameterization*.
  We have repeated the same analysis as for the $\gamma$-*parameterization* taking into account the possibility of coupling between DE and DM, i.e., we have modeled the growth factor according to Eq. (I.8.6) and the dark-energy equation of state as in Eq. (I.8.2) and marginalized over all parameters, including $\varOmega _k$. The marginalized errors are shown in columns $\sigma _{{\gamma }_{\text {marg},2}}$, $\sigma _{{w}_{\text {marg},2}}$ of Table 8 and the significance contours are shown in the three panels of Fig. 22 which is analogous to Fig. 21. Even if the ellipses are now larger we note that errors are still small enough to distinguish the fiducial model from the *f*(*R*) and DGP scenarios at $>\,1\sigma$ and $>\,2\sigma$ level respectively.
  Marginalizing over all other parameters we can compute the uncertainties in the $\gamma$ and $\eta$ parameters, as listed in Table 10. The relative confidence ellipses are shown in the left panel of Fig. 23. This plot shows that next generation Euclid-like surveys will be able to distinguish the reference model with no coupling (central, red dot) to the CDE model proposed by Amendola and Quercellini (2003) (white square) only at the $1\textendash 1.5\sigma$ level.

**Table 5:** Numerical values for $1\sigma$ constraints on parameters in Fig. 20 and figures of merit

	Case	$\sigma _{\gamma }$	$\sigma _{w}$	FoM
$b=\sqrt{1+z}$	Ref.	0.02	0.017	3052
With	Opt.	0.02	0.016	3509
$\varOmega _{k}$ fixed	Pess.	0.026	0.02	2106

Here we have fixed $\varOmega _{k}$ to its fiducial value, $\varOmega _k = 0$

**Table 6:** Numerical values for $1\sigma$ constraints on parameters $\gamma$ and *w* (assumed constant), relative to the red ellipses in Figs. 21, 22 and figures of merit

Bias	Case	$\sigma _{\gamma }$	FoM	
$b=\sqrt{1+z}$	Ref.	0.03	0.04	1342
	Opt.	0.03	0.03	1589
	Pess.	0.04	0.05	864

Here we have marginalized over $\varOmega _{k}$

**Table 7:** Numerical values for marginalized $1\sigma$ constraints on cosmological parameters using constant $\gamma$ and *w*

	Case	$\sigma _{h}$	$\sigma _{\varOmega _m h^2}$	$\sigma _{\varOmega _b h^2}$	$\sigma _{\varOmega _k}$	$\sigma _{n_s}$	$\sigma _{\sigma _8}$
$b=\sqrt{1+z}$	Ref.	0.007	0.002	0.0004	0.008	0.03	0.006

**Table 8:** $1\sigma$ marginalized errors for parameters $\gamma$ and *w* expressed through $\gamma$ and $\eta$ parameterizations

Bias	Case	$\sigma _{\gamma _{\mathrm {marg},1}}$	$\sigma _{w_{\mathrm {marg},1}}$	FoM	$\sigma _{\gamma _{\mathrm {marg},2}}$	$\sigma _{w_{\mathrm {marg},2}}$	FoM
$b=\sqrt{1+z}$	Ref.	0.15	0.07	97	0.07	0.07	216
	Opt.	0.14	0.06	112	0.07	0.06	249
	Pess.	0.18	0.09	66	0.09	0.09	147

Columns $\gamma _{0,\mathrm {marg}1},w_{0,\mathrm {marg}1}$ refer to marginalization over $\gamma _{1},w_{1}$ (Fig. 21) while columns $\gamma _{0,\mathrm {marg}2},w_{0,\mathrm {marg}2}$ refer to marginalization over $\eta ,w_{1}$ (Fig. 22)

**Table 9:** Numerical values for $1\sigma$ constraints on parameters in right panel of Fig. 20 and figures of merit

Bias	Case	$\sigma _{\gamma _{0}}$	$\sigma _{\gamma _{1}}$	FoM
$b=\sqrt{1+z}$	Ref.	0.15	0.4	87
	Opt.	0.14	0.36	102
	Pess.	0.18	0.48	58

**Table 10:** Numerical values for $1\sigma$ constraints on parameters in Fig. 23 and figures of merit

Bias	Case	$\sigma _{\gamma }$	$\sigma _{\eta }$	FoM
$b=\sqrt{1+z}$	Ref.	0.07	0.06	554
	Opt.	0.07	0.06	650
	Pess.	0.09	0.08	362

**Fig. 24:**
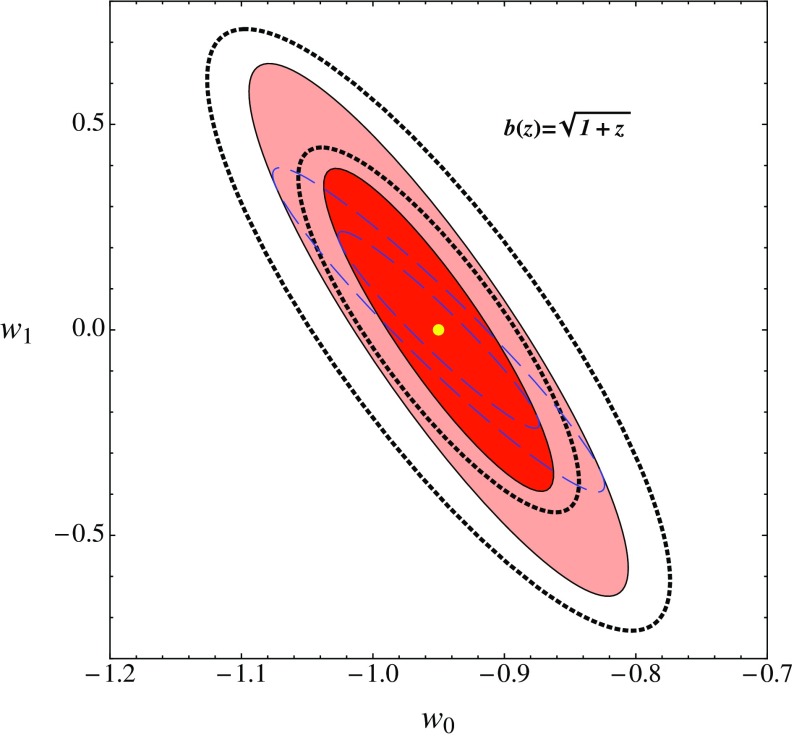
Errors on the equation of state. 1 and $2\sigma$ marginalized probability regions for the parameters $w_{0}$ and $w_{1}$, relative to the reference case and bias $b=\sqrt{(}1+z)$. The blue dashed ellipses are obtained fixing $\gamma _{0}, \gamma _{1}$ and $\varOmega _{k}=0$ to their fiducial values and marginalizing over all the other parameters; for the red shaded ellipses instead, we also marginalize over $\varOmega _{k}=0$ but we fix $\gamma _{0}, \gamma _{1}$. Finally, the black dotted ellipses are obtained marginalizing over all parameters but $w_{0}$ and $w_{1}$. The progressive increase in the number of parameters reflects in a widening of the ellipses with a consequent decrease in the figures of merit (see Table 11)

Finally, in order to explore the dependence on the number of parameters and to compare our results to previous works, we also draw the confidence ellipses for $w_0$, $w_1$ with three different methods: (i) fixing $\gamma _{0}, \gamma _{1}$ and $\varOmega _k$ to their fiducial values and marginalizing over all the other parameters; (ii) fixing only $\gamma _{0}$ and $\gamma _{1}$; (iii) marginalizing over all parameters but $w_0$, $w_1$. As one can see in Fig. 24 and Table 11 this progressive increase in the number of marginalized parameters reflects in a widening of the ellipses with a consequent decrease in the figures of merit. These results are in agreement with those of other authors (e.g., Wang et al. 2010).

The results obtained in this section can be summarized as follows.

1. If both $\gamma$ and *w* are assumed to be constant and setting $\varOmega _k=0$, then a redshift survey described by our *Reference case* will be able to constrain these parameters to within 4 and 2%, respectively.
2. Marginalizing over $\varOmega _{k}$ degrades these constraints to 5.3 and 4% respectively.
3. If *w* and $\gamma$ are considered redshift-dependent and parametrized according to Eqs. (I.8.5) and (I.8.2) then the errors on $\gamma _{0}$ and $w_{0}$ obtained after marginalizing over $\gamma _{1}$ and $w_{1}$ increase by a factor $\sim$ 7, 5. However, with this precision we will be able to distinguish the fiducial model from the DGP and *f*(*R*) scenarios with more than $2\sigma$ and $1\sigma$ significance, respectively.
4. The ability to discriminate these models with a significance above $2\sigma$ is confirmed by the confidence contours drawn in the $\gamma _{0}$–$\gamma _{1}$ plane, obtained after marginalizing over all other parameters.
5. If we allow for a coupling between dark matter and dark energy, and we marginalize over $\eta$ rather than over $\gamma _{1}$, then the errors on $w_{0}$ are almost identical to those obtained in the case of the $\gamma$-*parameterization*, while the errors on $\gamma _{0}$ decrease significantly.

**Table 11:** $1\sigma$ marginalized errors for the parameters $w_{0}$ and $w_{1}$, obtained with three different methods (reference case, see Fig. 24)

	$\sigma _{w_{0}}$	$\sigma _{w_{1}}$	FoM
$\gamma _{0}, \gamma _{1}$, $\varOmega _{k}$ fixed	0.05	0.16	430
$\gamma _{0},\gamma _{1}$ fixed	0.06	0.26	148
Marginalization over all other parameters	0.07	0.3	87

However, our ability in separating the fiducial model from the CDE model is significantly hampered: the confidence contours plotted in the $\gamma$–$\eta$ plane show that discrimination can only be performed wit $1 \textendash 1.5\sigma$ significance. Yet, this is still a remarkable improvement over the present situation, as can be appreciated from Fig. 23 where we compare the constraints expected by next generation data to the present ones. Moreover, the *Reference* survey will be able to constrain the parameter $\eta$ to within 0.06. Reminding that we can write $\eta =2.1 \beta _c^2$ (Di Porto and Amendola 2008), this means that the coupling parameter $\beta _c$ between dark energy and dark matter can be constrained to within 0.14, solely employing the growth rate information. This is comparable to existing constraints from the CMB but is complementary since obviously it is obtained at much smaller redshifts. A variable coupling could therefore be detected by comparing the redshift survey results with the CMB ones.

It is worth pointing out that, whenever we have performed statistical tests similar to those already discussed by other authors in the context of a Euclid-like survey, we did find consistent results. Examples of this are the values of FoM and errors for $w_0$, $w_1$, similar to those in Wang et al. (2010), Majerotto et al. (2012) and the errors on constant $\gamma$ and *w* (Majerotto et al. 2012). However, let us notice that all these values strictly depend on the parametrizations adopted and on the numbers of parameters fixed or marginalized over (see, e.g., Rassat et al. 2008).

#### Weak lensing non-parametric measurement of expansion and growth rate

In this section, we apply power-spectrum tomography (Hu 1999) to the Euclid weak lensing survey without using any parameterization of the Hubble parameter *H*(*z*) as well as the growth function *G*(*z*). Instead, we add the fiducial values of those functions at the center of some redshift bins of our choice to the list of cosmological parameters. Using the Fisher matrix formalism, we can forecast the constraints that future surveys can put on *H*(*z*) and *G*(*z*). Although such a non-parametric approach is quite common for as concerns the equation-of-state ratio *w*(*z*) in supernovae surveys (see, e.g., Albrecht et al. 2009) and also in redshift surveys (Seo and Eisenstein 2003), it has not been investigated for weak lensing surveys.

The Fisher matrix is given by (Hu and Tegmark 1999)

I.8.10 $$\begin{aligned} F_{\alpha \beta } = f_\mathrm {sky} \sum _\ell \frac{(2\ell +1)\varDelta \ell }{2}\frac{\partial P_{ij}(\ell )}{\partial p_\alpha }C^{-1}_{jk}\frac{\partial P_{km}(\ell )}{\partial p_\beta }C^{-1}_{mi}, \end{aligned}$$

where $f_\mathrm {sky}$ is the observed fraction of the sky, *C* is the covariance matrix, $P(\ell )$ is the convergence power spectrum and ${\mathbf {p}}$ is the vector of the parameters defining our cosmological model. Repeated indices are being summed over from 1 to *N*, the number of redshift bins. The covariance matrix is defined as (no summation over *j*)

I.8.11 $$\begin{aligned} C_{jk}=P_{jk} + \delta _{jk}\gamma _\mathrm {int}^2 n^{-1}_j, \end{aligned}$$

where $\gamma _\mathrm {int}$ is the intrinsic galaxy shear and $n_j$ is the fraction of galaxies per steradian belonging to the *j*-th redshift bin:

I.8.12 $$\begin{aligned} n_j = 3600 \left( \frac{180}{\pi } \right) ^2 n_\theta \int _{0}^\infty n_j(z){\mathrm {d}} z \end{aligned}$$

where $n_\theta$ is the galaxy density per arc minute and $n_j(z)$ the galaxy density for the *j*-th bin, convolved with a gaussian around ${\hat{z}}_j$, the center of that bin, with a width of $\sigma _z(1+{\hat{z}}_j)$ in order to account for errors in the redshift measurement.

For the matter power spectrum we use the fitting formulae from Eisenstein and Hu (1999) and for its nonlinear corrections the results from Smith et al. (2003). Note that this is where the growth function enters. The convergence power spectrum for the *i*-th and *j*-th bin can then be written as

I.8.13 $$\begin{aligned} P_{ij}(\ell ) = \frac{9H_0^3}{4}\int _0^\infty \frac{W_i(z)W_j(z)E^3(z)\varOmega _m^2(z)}{(1+z)^4} P_{\delta _m}\left( \frac{\ell }{\pi r(z)}\right) {\mathrm {d}} z. \end{aligned}$$

Here we make use of the window function

I.8.14 $$\begin{aligned} W_i(z) = \int _z^\infty \frac{\mathrm {d}{\tilde{z}}}{H({\tilde{z}})}\left[ 1-\frac{r(z)}{r({\tilde{z}})} \right] n_i[r({\tilde{z}})] \end{aligned}$$

(with *r*(*z*) being the comoving distance) and the dimensionless Hubble parameter

I.8.15 $$\begin{aligned} E^2(z) = \varOmega _m^{(0)}(1+z)^3 + (1-\varOmega _m^{(0)}) \exp \left[ \int _0^z \frac{3(1+w({\tilde{z}}))}{1+{\tilde{z}}}{\mathrm {d}}{\tilde{z}} \right] . \end{aligned}$$

For the equation-of-state ratio, finally, we use the usual CPL parameterization.

We determine *N* intervals in redshift space such that each interval contains the same amount of galaxies. For this we use the common parameterization

I.8.16 $$\begin{aligned} n(z) = z^2 \exp \left( -\left( z/z_0\right) ^{3/2}\right) , \end{aligned}$$

where $z_0 =z_\mathrm {mean}/1.412$ is the peak of *n*(*z*) and $z_\mathrm {mean}$ the median. Now we can define ${\hat{z}}_i$ as the center of the *i*-th redshift bin and add $h_i\equiv \log \left( {H({\hat{z}}_i)/H_0}\right)$ as well as $g_i\equiv \log G({\hat{z}}_i)$ to the list of cosmological parameters. The Hubble parameter and the growth function now become functions of the $h_i$ and $g_i$ respectively:

I.8.17 $$\begin{aligned} H(z;\varOmega _m^{(0)},w_0,w_1)&\rightarrow H(z;h_1,\ldots ,h_N) \end{aligned}$$

I.8.18 $$\begin{aligned} G(z;\varOmega _m^{(0)},\gamma )&\rightarrow G(z;g_1,\ldots ,g_N) \end{aligned}$$

This is being done by linearly interpolating the functions through their supporting points, e.g., $({\hat{z}}_i,\exp (h_i))$ for *H*(*z*). Any function that depends on either *H*(*z*) or *G*(*z*) hence becomes a function of the $h_i$ and $g_i$ as well.

**Table 12:** Values used in our computation

$\omega _m$	0.1341	$f_\mathrm {sky}$	0.375
$\omega _b$	0.02258	$z_\mathrm {mean}$	0.9
$\tau$	0.088	$\sigma _z$	0.05
$n_s$	0.963	$n_\theta$	30
$\varOmega _m$	0.266	$\gamma _\mathrm {int}$	0.22
$w_0$	– 1	$\ell _{\max }$	$5\times 10^3$
$w_1$	0	$\varDelta \log _{10}\ell$	0.02
$\gamma$	0.547		
$\gamma _{\mathrm {ppn}}$	0		
$\sigma _8$	0.801		

The values of the fiducial model (WMAP7, on the left) and the survey parameters (on the right)

The values for our fiducial model (taken from WMAP 7-year data, Komatsu et al. 2011) and the survey parameters that we chose for our computation can be found in Table 12.

As for the sum in Eq. (I.8.10), we generally found that with a realistic upper limit of $\ell _\mathrm {max} = 5\times 10^3$ and a step size of $\varDelta \lg \ell = 0.2$ we get the best result in terms of a figure of merit (FoM), that we defined as

I.8.19 $$\begin{aligned} \mathrm {FoM} = \sum \sigma _i^{-2}. \end{aligned}$$

Note that this is a fundamentally different FoM than the one defined by the Dark Energy Task Force. Our definition allows for a single large error without influencing the FoM significantly and should stay almost constant after dividing a bin arbitrarily in two bins, assuming the error scales roughly as the inverse of the root of the number of galaxies in a given bin.

**Fig. 25:**
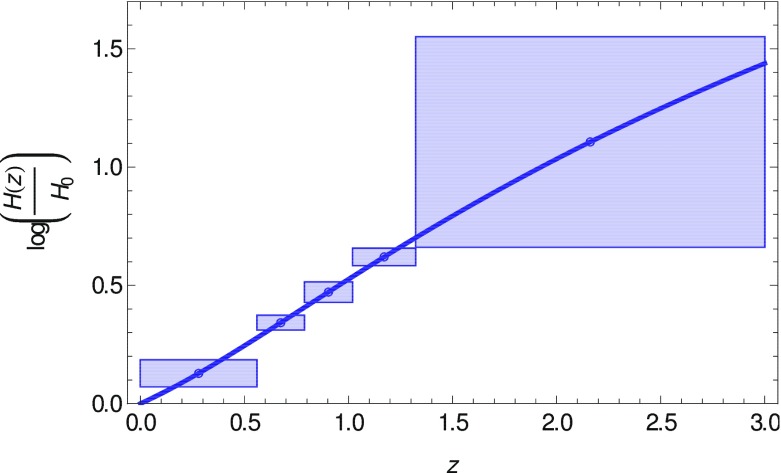
Error bars on the Hubble parameter *H*(*z*) with five redshift bins. The exact height of the error bars respectively are (0.23, 0.072, 0.089, 0.064, 0.76)

We first did the computation with just binning *H*(*z*) and using the common fit for the growth function slope (Wang and Steinhardt 1998)

**Fig. 26:**
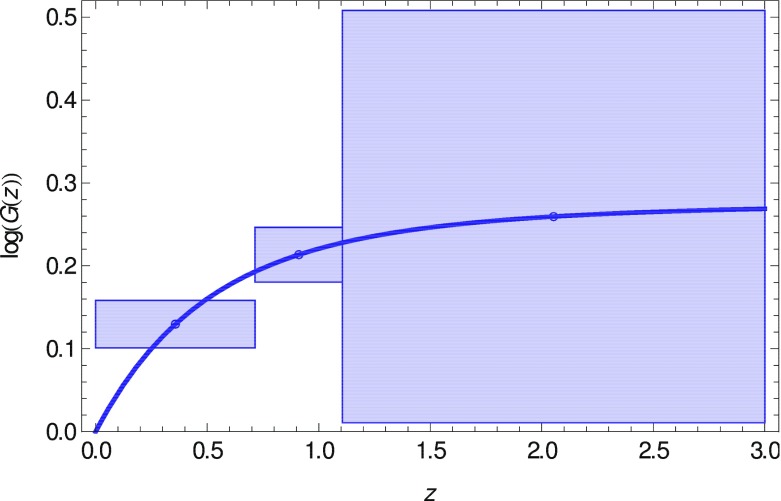
Error bars on the growth function *G*(*z*) with three redshift bins while marginalizing over the $h_i$s. The exact height of the error bars respectively are (0.029, 0.033, 0.25)

I.8.20 $$\begin{aligned} \frac{d \log G(z)}{d\log a} = \varOmega _m(z)^\gamma , \end{aligned}$$

yielding the result in Fig. 25. Binning both *H*(*z*) and *G*(*z*) and marginalizing over the $h_i$s yields the plot for *G*(*z*) seen in Fig. 26.

Notice that here we assumed no prior information. Of course one could improve the FoM by taking into account some external constraints due to other experiments.

#### Testing the non-linear corrections for weak lensing forecasts

In order to fully exploit the scientific potential of the next generation of weak lensing surveys, accurate predictions of the matter power spectrum are required. The signal-to-noise ratio of the cosmic shear signal is highest on angular scales of 5–10 arcminutes, which correspond to physical scales of $\sim \,1$ Mpc. Restricting the analysis to larger scales does not necessarily solve the problem, because the observed two-point ellipticity correlation functions are still sensitive to small scale structures projected along the line-of-sight. This may be avoided using a full 3D shear analysis (see Castro et al. 2005; Kitching et al. 2011, for details), but using only the larger scales increases the statistical uncertainties due to cosmic variance.

Currently only N-body simulations allow us to capture the non-linear structure formation, but for a survey such as *Euclid* an accuracy of $\sim \,1\%$ is needed (Huterer 2002; Huterer and Takada 2005). This accuracy goes beyond the claimed $\pm \, 3\%$ uncertainty of the popular halofit code (Smith et al. 2003). However, the accuracy can be improved provided the simulations are started with adequate initial conditions, with a large volume, sufficient time stepping and high mass resolution. For instance (Heitmann et al. 2010) obtained an accuracy of $\sim \,1\%$ out to $k\sim 1\,\hbox {h Mpc}^{-1}$ for a gravity-only simulation.

It is important to distinguish between gravity-only simulations, which are used to make the forecasts, and hydrodynamical simulations that attempt to capture the modifications to the matter power spectrum due to baryon physics. Although most of the matter in the Universe is indeed believed to be in the form of collissionless cold dark matter, baryons represent a non-negligible fraction of the total matter content. The distribution of baryons traces that of the underlying dark matter density field and thus gravity-only simulations should capture most of the structure formation. Nonetheless, differences in the spatial distribution of baryons with respect to the dark matter is expected to lead to changes that exceed the required accuracy of 1 per cent.

Various processes, which include radiative cooling, star formation and energy injection from supernovae and active galactic nuclei, affect the distribution of baryons. Implementing these processes correctly is difficult, and as a consequence the accuracy of hydrodynamic simulations is under discussion. That baryon physics cannot be ignored was perhaps most clearly shown in van Daalen et al. (2011) who looked at the changes in the matter power spectra when different processes are included. This was used by Semboloni et al. (2011) to examine the impact on cosmic shear studies. The results suggest that AGN feedback may lead to a suppression of the power by as much as $10\%$ at $k\sim \,1\,\hbox {h Mpc}^{-1}$.

Semboloni et al. (2011) showed that ignoring the baryonic physics leads to biases in the cosmological parameter estimates that are much larger than the precision of *Euclid*. In the case of the AGN model, the bias in *w* is as much as $40\%$. Unfortunately our knowledge of the various feedback processes is still incomplete and we cannot use the simulations to interpret cosmic shear signal. Furthermore, hydrodynamic simulations are too expensive to simulate large volumes for a range of cosmological parameters. To circumvent this problem several approaches have been suggested. For instance, Bernstein (2009) proposed to describe the changes in the power spectrum by Legendre polynomials, and to marginalise over the nuisance parameters (also see Kitching and Taylor 2011, for a similar approach). Although this leads to unbiased estimates for cosmological parameters, the precision decreases significantly, by as much as 30% (Zentner et al. 2008).

Instead Semboloni et al. (2011) and Semboloni et al. (2013) examined whether it is possible to model the effects of baryon physics using a halo model approach, in which the baryons and stars are treated separately from the dark matter distribution. The model parameters, rather than being mere nuisance parameters, correspond to physical quantities that can be constrained observationally. These works showed that even with this still rather simple approach it is possible to reduce the biases in the cosmological parameters to acceptable levels, without a large loss in precision.

The forecasts do not include the uncertainty due to baryon physics, hence the results implicitly assume that this can be understood sufficiently well that no loss in precision occurs. This may be somewhat optimistic, as more work is needed in the coming years to accurately quantify the impact of baryon physics on the modelling of the matter power spectrum, but we note that the initial results are very encouraging. In particular, Semboloni et al. (2013) found that requiring consistency between the two- and three-point statistics can be used to self-calibrate feedback models.

Another complication for the forecasts is the performance of the prescriptions for the non-linear power spectrum for non-$\varLambda$CDM models. For instance, McDonald et al. (2006) showed that, using halofit for non-$\varLambda$CDM models, requires suitable corrections. In spite of that, halofit has been often used to calculate the spectra of models with non-constant DE state parameter *w*(*z*). This procedure was dictated by the lack of appropriate extensions of halofit to non-$\varLambda$CDM cosmologies.

In this paragraph we quantify the effects of using the halofit code instead of *N*-body outputs for nonlinear corrections for DE spectra, when the nature of DE is investigated through weak lensing surveys. Using a Fisher-matrix approach, we evaluate the discrepancies in error forecasts for $w_{0}$, $w_{a}$ and $\varOmega _m$ and compare the related confidence ellipses. See Casarini et al. (2011) for further details.

The weak lensing survey is as specified in Sect. I.8.2. Tests are performed assuming three different fiducial cosmologies: $\varLambda$CDM model ($w_0 = -\,1$, $w_a = 0$) and two dynamical DE models, still consistent with the WMAP+BAO+SN combination (Komatsu et al. 2011) at 95% C.L. They will be dubbed M1 ($w_0 = -\,0.67$, $w_a = 2.28$) and M3 ($w_0 = -\,1.18$, $w_a = 0.89$). In this way we explore the dependence of our results on the assumed fiducial model. For the other parameters we adopt the fiducial cosmology of Sect. I.8.2.

The derivatives needed to calculate the Fisher matrix are evaluated by extracting the power spectra from the *N*-body simulations of models close to the fiducial ones, obtained by considering parameter increments $\pm \, 5\%$. For the $\varLambda$CDM case, two different initial seeds were also considered, to test the dependence on initial conditions, finding that Fisher matrix results are almost insensitive to it. For the other fiducial models, only one seed is used.

*N*-body simulations are performed by using a modified version of pkdgrav (Stadel 2001) able to handle any DE state equation *w*(*a*), with $N^3 = 256^{3}$ particles in a box with side $L = 256\, h^{-1}\mathrm {\ Mpc}$. Transfer functions generated using the camb package are employed to create initial conditions, with a modified version of the PM software by Klypin and Holtzman (1997), also able to handle suitable parameterizations of DE.

Matter power spectra are obtained by performing a fast Fourier transform (FFT) of the matter density fields, computed from the particles distribution through a Cloud-in-Cell algorithm, by using a regular grid with $N_{g}=2048$. This allows us to obtain nonlinear spectra in a large *k*-interval. In particular, our resolution allows to work out spectra up to $k \simeq 10\, h\mathrm {\ Mpc}^{-1}$. However, for $k > 2\textendash 3\, h\mathrm {\ Mpc}^{-1}$ neglecting baryon physics is no longer accurate (Jing et al. 2006; Rudd et al. 2008; Bonometto et al. 2010; Zentner et al. 2008; Hearin and Zentner 2009). For this reason, we consider WL spectra only up to $\ell _{\max } = 2000$.

Particular attention has to be paid to matter power spectra normalizations. In fact, we found that, normalizing all models to the same linear $\sigma _8 (z=0)$, the shear derivatives with respect to $w_0$, $w_a$ or $\varOmega _m$ were largely dominated by the normalization shift at $z=0$, $\sigma _{8}$ and $\sigma _{8,nl}$ values being quite different and the shift itself depending on $w_0$, $w_a$ and $\varOmega _m$. This would confuse the *z* dependence of the growth factor, through the observational *z*-range. This normalization problem was not previously met in analogous tests with the Fisher matrix, as halofit does not directly depend on the DE state equation.

As a matter of fact, one should keep in mind that, observing the galaxy distribution with future surveys, one can effectively measure $\sigma _{8,nl}$, and not its linear counterpart. For these reasons, we choose to normalize matter power spectra to $\sigma _{8,nl}$, assuming to know it with high precision.

**Fig. 27:**
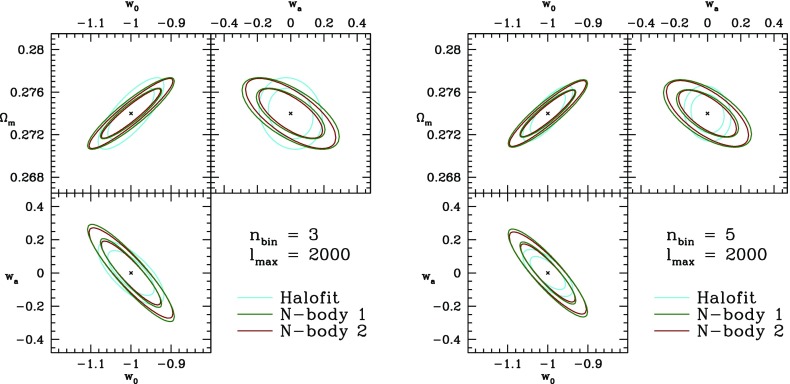
Likelihood contours, for 65 and 95% C.L., calculated including signals up to $\ell \simeq 2000$ for the $\varLambda$CDM fiducial. Here simulations and halofit yield significantly different outputs

In Fig. 27 we show the confidence ellipses, when the fiducial model is $\varLambda$CDM, in the cases of 3 or 5 bins and with $\ell _{\max } = 2000$. Since the discrepancy between different seeds are small, discrepancies between halofit and simulations are truly indicating an underestimate of errors in the halofit case.

As expected, the error on $\varOmega _m$ estimate is not affected by the passage from simulations to halofit, since we are dealing with $\varLambda$CDM models only. On the contrary, using halofit leads to underestimates of the errors on $w_0$ and $w_a$, by a substantial 30–40% (see Casarini et al. (2011) for further details).

This confirms that, when considering models different from $\varLambda$CDM, nonlinear correction obtained through halofit may be misleading. This is true even when the fiducial model is $\varLambda$CDM itself and we just consider mild deviations of *w* from $-\,1$.

**Fig. 28:**
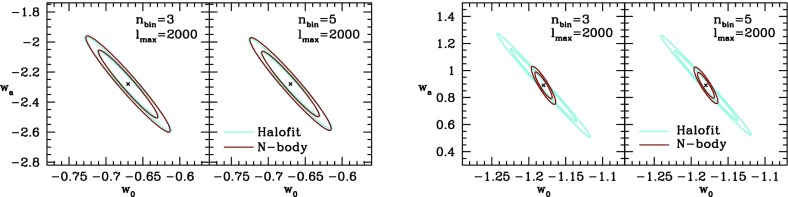
On the left (right) panel, 1- and 2-$\sigma$ contours for the M1 (M3) model. The two fiducial models exhibit quite different behaviors

Figure 28 then show the results in the $w_0$–$w_a$ plane, when the fiducial models are M1 or M3. It is evident that the two cases are quite different. In the M1 case, we see just quite a mild shift, even if they are ${\mathcal {O}}$ (10%) on error predictions. In the M3 case, errors estimated through halofit exceed simulation errors by a substantial factor. Altogether, this is a case when estimates based on halofit are not trustworthy.

The effect of baryon physics is another nonlinear correction to be considered. We note that the details of a study on the impact of baryon physics on the power spectrum and the parameter estimation can be found in Semboloni et al. (2011)

#### Forecasts for the dark-energy sound speed

As we have seen in Sect. I.3.1, when dark energy clusters, the standard sub-horizon Poisson equation that links matter fluctuations to the gravitational potential is modified and $Q\ne 1$. The deviation from unity will depend on the degree of DE clustering and therefore on the sound speed $c_s$. In this subsection, we try to forecast the constraints that Euclid can put on a constant $c_s$ by measuring *Q* both via weak lensing and via redshift clustering. Here we assume standard Einstein gravity and zero anisotropic stress (and therefore we have $\varPsi =\varPhi$) and we allow $c_{s}$ to assume different values in the range 0–1.

Generically, while dealing with a non-zero sound speed, we have to worry about the sound horizon $k_{sh}=aH/c_{s}$, which characterizes the growth of the perturbations; then we have at least three regimes with different behavior of the perturbations:

1. perturbations larger than the causal horizon (where perturbations are not causally connected and their growth is suppressed),
2. perturbations smaller than the causal horizon but larger than the sound horizon, $k\ll aH/c_{s}$ (this is the only regime where perturbations are free to grow because the velocity dispersion, or equivalently the pressure perturbation, is smaller than the gravitational attraction),
3. perturbations smaller than the sound horizon, $k\gg aH/c_{s}$ (here perturbations stop growing because the pressure perturbation is larger than the gravitational attraction).

As we have set the anisotropic stress to zero, the perturbations are fully described by *Q*. The main problem is therefore to find an explicit expression that shows how *Q* depends on $c_s$. Sapone and Kunz (2009) have provided the following explicit approximate expression for $Q\left( k,a\right)$ which captures the behavior for both super- and sub-horizon scales:

I.8.21 $$\begin{aligned} Q(k,a)=1+\frac{1-\varOmega _{M,0}}{\varOmega _{M,0}}\frac{(1+w)a^{-3w}}{1-3w+\frac{2}{3}\nu (a)^{2}}. \end{aligned}$$

Here, $\nu (a)^{2}=k^{2}c_{s}^2 a/\left( \varOmega _{M,0}H_{0}^{2}\right)$, which it is defined through $c_{s}k\equiv \nu aH$ so that $\nu$ counts how deep a mode is inside the sound horizon.

Eq. (I.8.21) depends substantially on the value of the sound speed or, to put it differently, on the scale considered. For scales larger than the sound horizon ($\nu \approx 0$), Eq. (I.8.21) scales as $a^{-3w}$ and for $\varOmega _{m,0}=0.25$ and $w=-\,0.8$ we have that

I.8.22 $$\begin{aligned} Q-1\approx \frac{3}{17}a^{2.4}\simeq 0.18a^{2.4}. \end{aligned}$$

This is not a negligible deviation today, but it decreases rapidly as we move into the past, as the dark energy becomes less important.^12^ As a scale enters the sound horizon, $Q-1$ grows with one power of the scale factor slower (since $\delta _{\mathrm {DE}}$ stops growing), suppressing the final deviation roughly by the ratio of horizon size to the scale of interest (as now $\nu ^2\gg 1$). In the observable range, $(k/H_{0})^{2}\approx 10^{2}\textendash 10^{4}$. Therefore, if $c_{s}\approx 1$, $Q\rightarrow 1$ and the dependence on $c_{s}$ is lost. This shows that *Q* is sensitive to $c_{s}$ only for small values, $c_{s}^{2}\lesssim 10^{-2}$.

We can characterize the dependence of *Q* on the main perturbation parameter $c_{s}^2$ by looking at its derivative, a key quantity for Fisher matrix forecasts:

I.8.23 $$\begin{aligned} \frac{\partial \log Q}{\partial \log c_s^2}=-\frac{x}{\left( 1+x\right) }\frac{Q-1}{Q}, \end{aligned}$$

where $x=\frac{2}{3}\nu (a)^{2}/(1-3w)\simeq 0.2\nu (a)^{2}$ (with the last expression being for $w=-\,0.8$). For the values we are interested in here, this derivative has a peak at the present epoch at the sound horizon, i.e., for $c_{s}\approx H_{0}/k$, which in the observable range of *k* is $c_{s}\approx .01-.001$, and declines rapidly for larger $c_{s}$. This means that the sensitivity of *Q* to the sound speed can be boosted by several orders of magnitude as the sound speed is decreased.

There are several observables that depend on *Q*:

- The growth of matter perturbations
  There are two ways to influence the growth factor: firstly at background level, with a different Hubble expansion. Secondly at perturbation level: if dark energy clusters then the gravitational potential changes because of the Poisson equation, and this will also affect the growth rate of dark matter. All these effects can be included in the growth index $\gamma$ and we therefore expect that $\gamma$ is a function of *w* and $c_s^2$ (or equivalently of *w* and *Q*).
  The growth index depends on dark-energy perturbations (through *Q*) as (Sapone and Kunz 2009)
  I.8.24 $$\begin{aligned} \gamma =\frac{3\left( 1-w-A\left( Q\right) \right) }{5-6w} \end{aligned}$$
  where
  I.8.25 $$\begin{aligned} A\left( Q\right) =\frac{Q-1}{1-\varOmega _{M}\left( a\right) }. \end{aligned}$$
  Clearly here, the key quantity is the derivative of the growth factor with respect to the sound speed:
  I.8.26 $$\begin{aligned} \frac{\partial \log G}{\partial \ln c_s^2}\propto \int _{a_0}^{a_1}{\frac{\partial \gamma }{\partial c_{s}^2}{\mathrm {d}}a}\propto \int _{a_0}^{a_1}{\frac{\partial Q}{\partial c_{s}^2}{\mathrm {d}}a} \propto \int _{a_0}^{a_1}{\left( Q-1\right) {\mathrm {d}}a}. \end{aligned}$$
  From the above equation we also notice that the derivative of the growth factor does not depend on $Q-1$ like the derivative *Q*, but on $Q-Q_{0}$ as it is an integral (being $Q_0$ the value of *Q* today). The growth factor is thus not directly probing the deviation of *Q* from unity, but rather how *Q* evolves over time, see Sapone et al. (2010) for more details.
- Redshift space distortions
  The distortion induced by redshift can be expressed in linear theory by the $\beta$ factor, related to the bias factor and the growth rate via:
  I.8.27 $$\begin{aligned} \beta (z,k)=\frac{\varOmega _{m}\left( z\right) ^{\gamma (k,z)}}{b(z)}. \end{aligned}$$
  The derivative of the redshift distortion parameter with respect to the sound speed is:
  I.8.28 $$\begin{aligned} \frac{\partial \log \left( 1+\beta \mu ^{2}\right) }{\partial \log c_s^2}= -\frac{3}{5-6w}\frac{\beta \mu ^{2}}{1+\beta \mu ^{2}}\frac{x}{1+x}\left( Q-1\right) . \end{aligned}$$
  We see that the behavior versus $c_{s}^{2}$ is similar to the one for the *Q* derivative, so the same discussion applies. Once again, the effect is maximized for small $c_{s}$. The $\beta$ derivative is comparable to that of *G* at $z=0$ but becomes more important at low redshifts.
- Shape of the dark matter power spectrum
  Quantifying the impact of the sound speed on the matter power spectrum is quite hard as we need to run Boltzmann codes (such as camb, Lewis et al. 2000b) in order to get the full impact of dark-energy perturbations into the matter power spectrum. Sapone et al. (2010) proceeded in two ways: first using the camb output and then considering the analytic expression from Eisenstein and Hu (1999) (which does not include dark energy perturbations, i.e., does not include $c_{s}$).
  They find that the impact of the derivative of the matter power spectrum with respect the sound speed on the final errors is only relevant if high values of $c_s^2$ are considered; by decreasing the sound speed, the results are less and less affected. The reason is that for low values of the sound speed other parameters, like the growth factor, start to be the dominant source of information on $c_{s}^{2}$.

Impact on weak lensing

Now it is possible to investigate the response of weak lensing (WL) to the dark-energy parameters. Proceeding with a Fisher matrix as in Amendola et al. (2008b), the main difference here being that the parameter *Q* has an explicit form. Since *Q* depends on *w* and $c_s^2$, we can forecast the precision with which those parameters can be extracted. We can also try to trace where the constraints come from. For a vanishing anisotropic stress the WL potential becomes:

I.8.29 $$\begin{aligned} k^{2}\left( \varPhi +\varPsi \right) = -2Q\frac{3H_{0}^{2}\varOmega _{M,0}}{2a}\varDelta _{M} \end{aligned}$$

which can be written, in linear perturbation theory as:

I.8.30 $$\begin{aligned} k^{2}\left( \varPhi +\varPsi \right) =-\,3H\left( a\right) ^{2}a^{3}Q\left( a,k\right) \varOmega _{M}\left( a\right) G\left( a,k\right) \varDelta _{M}\left( k\right) . \end{aligned}$$

Hence, the lensing potential contains three conceptually different contributions from the dark-energy perturbations:

- The direct contribution of the perturbations to the gravitational potential through the factor *Q*.
- The impact of the dark-energy perturbations on the growth rate of the dark matter perturbations, affecting the time dependence of $\varDelta _{M}$, through $G\left( a,k\right)$.
- A change in the shape of the matter power spectrum *P*(*k*), corresponding to the dark energy induced *k* dependence of $\varDelta _{M}$.

We use the representative Euclid survey presented in Sect. I.8.2 and we extend our survey up to three different redshifts: $z_{\max }=2,3,4$. We choose different values of $c_s^2$ and $w_0 = -\,0.8$ in order to maximize the impact on *Q*: values closer to $-\,1$ reduce the effect and therefore increase the errors on $c_{s}$.

**Fig. 29:**
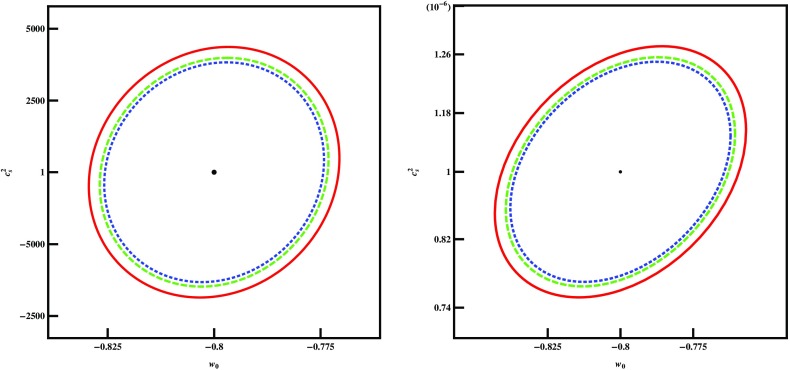
Confidence region at 68% for three different values of $z_{\max }=2.5,3.5,4$, red solid, green long-dashed and blue dashed contour, respectively. The left panel shows the confidence region when the sound speed is $c_s^2=1$; the right panel with the sound speed $c_s^2=10^{-6}$. The equation of state parameter is for both cases $w_{0}=-\,0.8$

In Fig. 29, we report the $1-\sigma$ confidence region for $w_{0},c_s^2$ for two different values of the sound speed and $z_{\max }$. For high value of the sound speed ($c_s^2=1$) we find $\sigma (w_{0})=0.0195$ and the relative error for the sound speed is $\sigma (c_s^2)/c_s^2=2615$. As expected, WL is totally insensitive to the clustering properties of quintessence dark-energy models when the sound speed is equal to 1. The presence of dark-energy perturbations leaves a *w* and $c_s^2$ dependent signature in the evolution of the gravitational potentials through $\varDelta _{\mathrm {DE}}/\varDelta _{m}$ and, as already mentioned, the increase of the $c_s^2$ enhances the suppression of dark-energy perturbations which brings $Q \rightarrow 1$.

Once we decrease the sound speed then dark-energy perturbations are free to grow at smaller scales. In Fig. 29, the confidence region for $w_{0},c_s^2$ for $c_s^2=10^{-6}$ is shown; we find $\sigma (w_{0})=0.0286$, $\sigma (c_s^2)/c_s^2=0.132$; in the last case the error on the measurement of the sound speed is reduced to the 70% of the total signal.

Impact on galaxy power spectrum.

We now explore a second probe of clustering, the galaxy power spectrum. The procedure is the same outlined in Sect. I.7.3. We use the representative Euclid survey presented in Sect. I.8.2. Here too we also consider in addition possible extended surveys to $z_{\max }=2.5$ and $z_{\max }=4$.

**Fig. 30:**
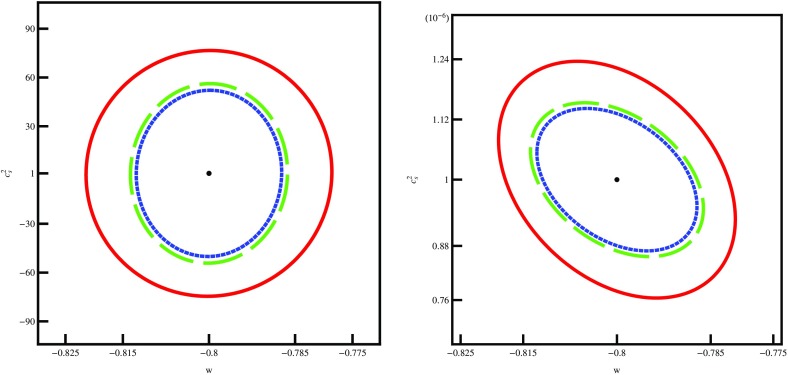
Confidence region at 68% for three different values of $z_{\max }=2.5,3.5,4$, red solid, green long-dashed and blue dashed contour, respectively. The left panel shows the confidence region when the sound speed is $c_s^2=1$; the right panel with the sound speed $c_s^2=10^{-6}$. The equation of state parameter is for both cases $w_{0}=-\,0.8$

In Fig. 30, we report the confidence region for $w_{0},c_s^2$ for two different values of the sound speed and $z_{\max }$. For high values of the sound speed ($c_s^2=1$) we find, for our benchmark survey: $\sigma (w_{0})=0.0133$, and $\sigma (c_s^2)/c_s^2=50.05$. Here again we find that galaxy power spectrum is not sensitive to the clustering properties of dark energy when the sound speed is of order unity. If we decrease the sound speed down to $c_s^2=10^{-6}$ then the errors are $\sigma (w_{0})=0.0125$, $\sigma (c_s^2)/c_s^2=0.118$.

In conclusion, as perhaps expected, we find that dark-energy perturbations have a very small effect on dark matter clustering unless the sound speed is extremely small, $c_{s}\le 0.01$. Let us remind that in order to boost the observable effect, we always assumed $w=-\,0.8$; for values closer to $-\,1$ the sensitivity to $c_s^2$ is further reduced. As a test, Sapone et al. (2010) performed the calculation for $w=-\,0.9$ and $c_s^2=10^{-5}$ and found $\sigma _{c_s^2}/c_s^2=2.6$ and $\sigma _{c_s^2}/c_s^2=1.09$ for WL and galaxy power spectrum experiments, respectively.

Such small sound speeds are not in contrast with the fundamental expectation of dark energy being much smoother that dark matter: even with $c_{s}\approx 0.01$, dark-energy perturbations are more than one order of magnitude weaker than dark matter ones (at least for the class of models investigated here) and safely below nonlinearity at the present time at all scales. Models of “cold” dark energy are interesting because they can cross the phantom divide (Kunz and Sapone 2006) and contribute to the cluster masses (Creminelli et al. 2010) (see also Sect. I.6.2 of this review). Small $c_{s}$ could be constructed for instance with scalar fields with non-standard kinetic energy terms.

#### Weak lensing constraints on *f(R)* gravity

In this section, we present the Euclid weak lensing forecasts of a specific, but very popular, class of models, the so-called *f*(*R*) models of gravity. As we have already seen in Sect. I.5.4 these models are described by the action

I.8.31 $$\begin{aligned} S_{\mathrm {grav}} = \int \sqrt{-g} \, \mathrm {d}^{4}x \left[ \frac{f(R)}{16\pi G} - {{\mathcal {L}}}_{\mathrm {m}} \right] , \end{aligned}$$

where *f*(*R*) is an arbitrary function of the Ricci scalar and $\mathcal{L}_{\mathrm {m}}$ is the Lagrange density of standard matter and radiation.

In principle one has complete freedom to specify the function *f*(*R*), and so any expansion history can be reproduced. However, as discussed in Sect. I.5.4, those that remain viable are the subset that very closely mimic the standard $\varLambda$CDM background expansion, as this restricted subclass of models can evade solar system constraints (Chiba 2003; Tsujikawa et al. 2008a; Gu 2011), have a standard matter era in which the scale factor evolves according to $a(t) \propto t^{2/3}$ (Amendola et al. 2007b) and can also be free of ghost and tachyon instabilities (Nariai 1973; Gurovich and Starobinsky 1979).

**Fig. 31:**
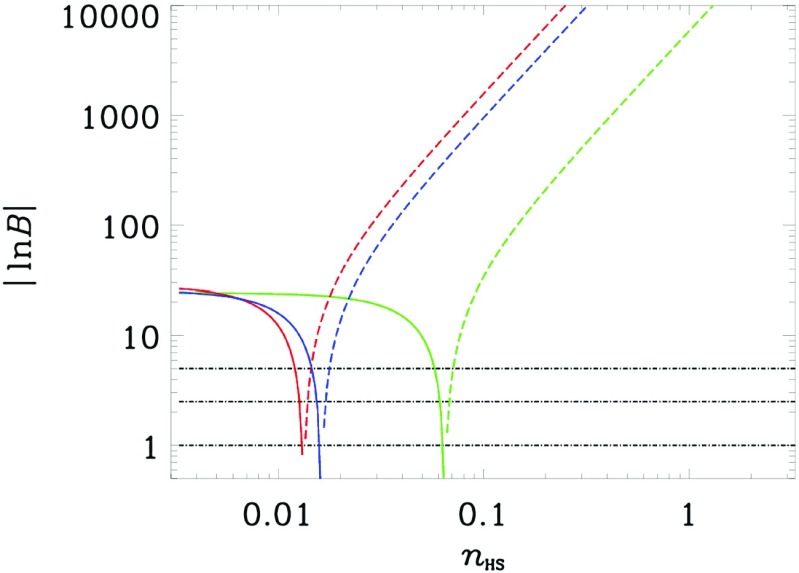
The Bayes factor $\ln B$ for the *f*(*R*) model of Eq. (I.5.36) over standard $\varLambda$CDM as a function of the extra parameter *n*. The green, red and blue curves refer to the conservative, bin-dependent and optimistic $\ell _{\max }$, respectively. The horizontal lines denote the Jeffreys’ scale levels of significance

To this subclass belongs the popular *f*(*R*) model proposed by Hu and Sawicki (2007a) (I.5.36). Camera et al. (2011b) demonstrated that Euclid will have the power of distinguishing between it and $\varLambda$CDM with a good accuracy. They performed a tomographic analysis using several values of the maximum allowed wavenumber of the Fisher matrices; specifically, a conservative value of 1000, an optimistic value of 5000 and a bin-dependent setting, which increases the maximum angular wavenumber for distant shells and reduces it for nearby shells. Moreover, they computed the Bayesian expected evidence for the model of Eq. (I.5.36) over the $\varLambda$CDM model as a function of the extra parameter *n*. This can be done because the $\varLambda$CDM model is formally nested in this *f*(*R*) model, and the latter is equivalent to the former when $n=0$. Their results are shown in Fig. 31. For another Bayesian evidence analysis of *f*(*R*) models and the added value of probing the growth of structure with galaxy surveys see also Song et al. (2007b).

This subclass of *f*(*R*) models can be parameterized solely in terms of the mass of the scalar field, which as we have seen in Eq. (I.5.55) is related to the *f*(*R*) functional form via the relation

I.8.32 $$\begin{aligned} M^{2}(a) = {\frac{1}{3 f_{,RR}[R_{\mathrm {back}}(a)]}} \end{aligned}$$

where *R* subscripts denote differentiation with respect to *R*. The function $f_{,RR}$ can be approximated by its standard $\varLambda$CDM form,

I.8.33 $$\begin{aligned} {\frac{R_{\mathrm {back}}}{H_{0}^{2}}} \simeq {\frac{3\varOmega _\mathrm{m0}}{a^{3}}} + 12\varOmega _{\varLambda }, \end{aligned}$$

valid for $z \lesssim 1000$. The mass *M*(*a*) is typically a function of redshift which decays from a large value in the early universe to its present day value $M_{0}$.

Whilst these models are practically indistinguishable from $\varLambda$CDM at the level of background expansion, there is a significant difference in the evolution of perturbations relative to the standard GR behavior.

The evolution of linear density perturbations in the context of *f*(*R*) gravity is markedly different than in the standard $\varLambda$CDM scenario; $\delta _{\mathrm {m}} \equiv \delta \rho _{\mathrm {m}}/\rho _{\mathrm {m}}$ acquires a nontrivial scale dependence at late times. This is due to the presence of an additional scale *M*(*a*) in the equations; as any given mode crosses the modified gravity ‘horizon’ $k = aM(a)$, said mode will feel an enhanced gravitational force due to the scalar field. This will have the effect of increasing the power of small scale modes.

Perturbations on sub-horizon scales in the Newtonian gauge evolve approximately according to

I.8.34 $$\begin{aligned}&\varPsi = \left( 1 + {\frac{2\bar{K}^{2}}{3 + 2\bar{K}^{2}}}\right) \varPhi , \end{aligned}$$

I.8.35 $$\begin{aligned}&k^{2}\varPhi = -4\pi G \left( {\frac{3 + 2\bar{K}^{2}}{3 + 3\bar{K}^{2}}}\right) a^{2}\rho _{\mathrm {m}} \delta _{\mathrm {m}}, \end{aligned}$$

I.8.36 $$\begin{aligned}&\ddot{\delta }_{\mathrm {m}} + 2H \dot{\delta }_{\mathrm {m}} - 4\pi G \left( {\frac{3 + 4\bar{K}^{2}}{ 3 + 3\bar{K}^{2}}}\right) \rho _{\mathrm {m}}\delta _{\mathrm {m}} = 0, \end{aligned}$$

where $\bar{K} = k/(aM(a))$. These equations represent a particular example of a general parameterization introduced in Martinelli et al. (2010a), Bertschinger and Zukin (2008) and Zhao et al. (2009b). To solve them one should first parameterize the scalaron mass *M*(*a*), choosing a form that broadly describes the behavior of viable *f*(*R*) models. A suitable functional form, which takes into account the evolution of *M*(*a*) in both the matter era and the late-time accelerating epoch, is given by Thomas et al. (2011)

I.8.37 $$\begin{aligned} M^{2} = M_{0}^{2} \left( {\frac{ a^{-3} + 4 a_{*}^{-3}}{ 1 + 4 a_{*}^{-3}}}\right) ^{2\nu }, \end{aligned}$$

where $a_{*}$ is the scale factor at matter-$\varLambda$ equality; $a_{*} = (\varOmega _{\mathrm{m0}}/\varOmega _{\varLambda })^{1/3}$. There are two modified gravity parameters; $M_{0}$ is the mass of the scalaron at the present time and $\nu$ is the rate of increase of *M*(*a*) to the past.

**Fig. 32:**
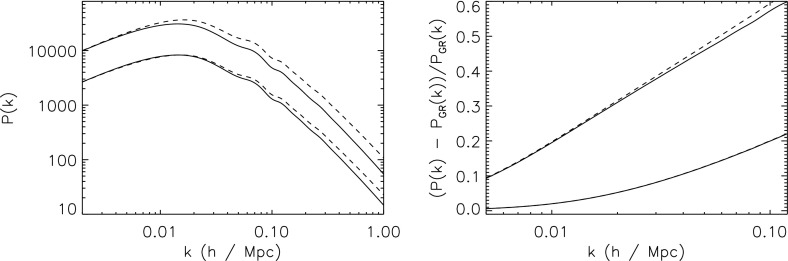
Left panel: Linear matter power spectra for $\varLambda$CDM (solid line; $M_{0}^{-1}=0$, $\nu =1.5$) and scalaron (dashed line; $M^{-1}_{0}=375\,[10^{28}\mathrm {\ h^{-1}\ eV^{-1}}]$, $\nu =1.5$) cosmologies. The modification to gravity causes a sizeable scale dependent effect in the growth of perturbations. The redshift dependence of the scalaron can be seen by comparing the top and bottom pairs of power spectra evaluated at redshifts $z=0.0$ and $z=1.5$, respectively. Right panel: The environmental dependent chameleon mechanism can be seen in the mildly nonlinear regime. We exhibit the fractional difference $(P(k) - P_{\mathrm {GR}}(k))/P_{\mathrm {GR}}(k)$ between the *f*(*R*) and GR power spectra for the model (I.8.37) with parameters $M^{-1}_{0}=375\,[10^{28}\mathrm {\ h^{-1}\ eV^{-1}}]$ and $\nu =1.5$. The dashed lines represent linear power spectra (*P*(*k*) and $P_{\mathrm {GR}}(k)$ calculated with no higher order effects) and the solid lines are the power spectra calculated to second order. We see that the nonlinearities decrease the modified gravity signal. This is a result of the chameleon mechanism. The top set of lines correspond to $z=0$ and the bottom to $z=0.9$; demonstrating that the modified gravity signal dramatically decreases for larger *z*. This is due to the scalaron mass being much larger at higher redshifts. Furthermore, nonlinear effects are less significant for increasing *z*

In Fig. 32, the linear matter power spectrum is exhibited for this parameterization (dashed line), along with the standard $\varLambda$CDM power spectrum (solid line). The observed, redshift dependent tilt is due to the scalaron’s influence on small scale modes, and represents a clear modified gravity signal. Since weak lensing is sensitive to the underlying matter power spectrum, we expect Euclid to provide direct constraints on the mass of the scalar field.

**Fig. 33:**
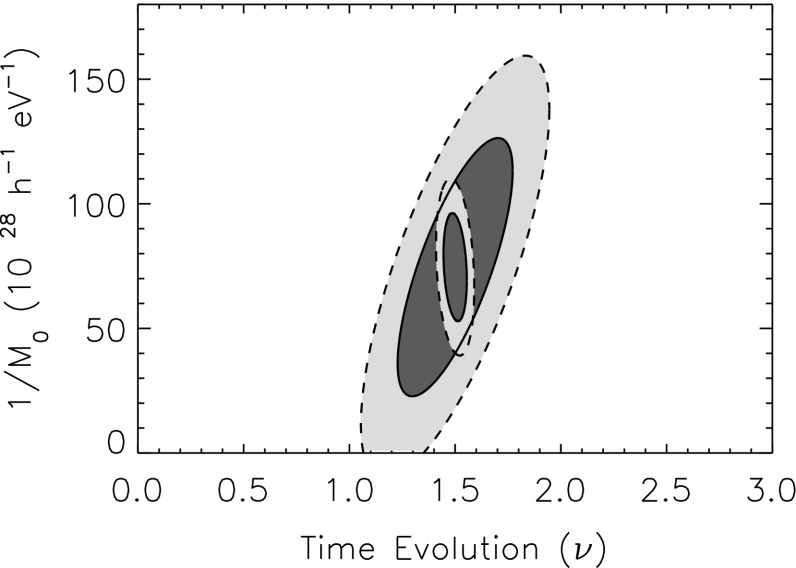
68% (dark grey) and 95% (light grey) projected bounds on the modified gravity parameters $M_{0}^{-1}$ and $\nu$ for the combined Euclid weak lensing and Planck CMB surveys. The larger (smaller) contours correspond to including modes $l = 400\,(10{,}000)$ in the weak lensing analysis

By performing a Fisher analysis, using the standard Euclid specifications, Thomas et al. (2011) calculates the expected *f*(*R*) parameter sensitivity of the weak lensing survey. By combining Euclid weak lensing and Planck Fisher matrices, both modified gravity parameters $M_{0}$ and $\nu$ are shown to be strongly constrained by the growth data in Fig. 33. The expected $1\sigma$ bounds on $M_{0}$ and $\nu$ are quoted as $M_{0} = 1.34 \pm 0.62 \times 10^{-30}\, [\mathrm {h\ eV}]$, $\nu = 1.5 \pm 0.18$ when using linear data $l < 400$ only and $M_{0} = 1.34 \pm 0.25 \times 10^{-30}\, [\mathrm {h\ eV}]$, $\nu = 1.5 \pm 0.04$ when utilizing the full set of nonlinear modes $l < 10{,}000$.

**Fig. 34:**
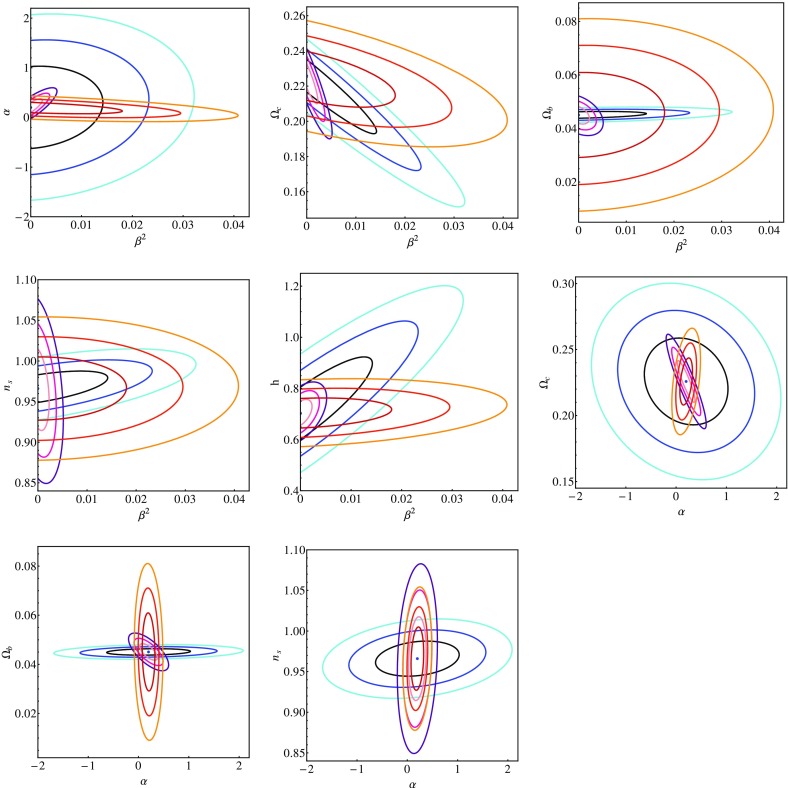
Comparison among predicted confidence contours for the cosmological parameter set $\varTheta \equiv \{\beta ^{2},\alpha ,\varOmega _{c},h,\varOmega _{b},n_{s},\sigma _{8},\log (A)\}$ using CMB (Planck, blue contours), *P*(*k*) (pink-violet contours) and weak lensing (orange-red contours) with Euclid-like specifications. Image reproduced by permission from Amendola et al. (2011), copyright by APS

#### Forecast constraints on coupled quintessence cosmologies

In this section, we present forecasts for coupled quintessence cosmologies (Amendola 2000a; Wetterich 1995; Pettorino and Baccigalupi 2008), obtained when combining Euclid weak lensing, Euclid redshift survey (baryon acoustic oscillations, redshift distortions and full *P*(*k*) shape) and CMB as obtained in Planck (see also the next section for CMB priors). Results reported here were obtained in Amendola et al. (2011) and we refer to it for details on the analysis and Planck specifications (for weak lensing and CMB constraints on coupled quintessence with a different coupling see also Martinelli et al. 2010b; De Bernardis et al. 2011). In Amendola et al. (2011), the coupling is the one described in Sect. I.5.3.4, as induced by a scalar–tensor model. The slope $\alpha$ of the Ratra–Peebles potential is included as an additional parameter and Euclid specifications refer to the Euclid Definition phase (Laureijs et al. 2011).

The combined Fisher confidence regions are plotted in Fig. 34 and the results are in Table 13. The main result is that future surveys can constrain the coupling of dark energy to dark matter $\beta ^{2}$ to less than $3\times 10^{-4}$. Interestingly, some combinations of parameters (e.g., $\varOmega _b$ vs. $\alpha$) seem to profit the most from the combination of the three probes.

**Table 13:** 1-$\sigma$ errors for the set $\varTheta \equiv \{\beta ^{2},\alpha ,\varOmega _{c},h,\varOmega _{b},n_{s}\,\sigma _{8},\log (A)\}$ of cosmological parameters, combining $\hbox {CMB}+P(k)$ (left column) and $\hbox {CMB}+P(k)+\hbox {WL}$ (right column)

Parameter	$\sigma _{i}\hbox {CMB}+P(k)$	$\sigma _{i}\,\hbox {CMB}+\hbox {P}(k)+\hbox {WL}$
$\beta ^{2}$	0.00051	0.00032
$\alpha$	0.055	0.032
$\varOmega _{c}$	0.0037	0.0010
*h*	0.0080	0.0048
$\varOmega _{b}$	0.00047	0.00041
$n_{s}$	0.0057	0.0049
$\sigma _{8}$	0.0049	0.0036
$\log (A)$	0.0051	0.0027

We can also ask whether a better knowledge of the parameters $\{\alpha ,\varOmega _{c},h,\varOmega _{b},n_{s},\sigma _{8},\log (A)\}$, obtained by independent future observations, can give us better constraints on the coupling $\beta ^{2}$. In Table 14 we show the errors on $\beta ^{2}$ when we have a better knowledge of only one other parameter, which is here fixed to the reference value. All remaining parameters are marginalized over.

It is remarkable to notice that the combination of CMB, power spectrum and weak lensing is already a powerful tool and a better knowledge of one parameter does not improve much the constraints on $\beta ^{2}$. CMB alone, instead, improves by a factor 3 when $\varOmega _{c}$ is known and by a factor 2 when *h* is known. The power spectrum is mostly influenced by $\varOmega _{c}$, which allows to improve constraints on the coupling by more than a factor 2. Weak lensing gains the most by a better knowledge of $\sigma _{8}$.

**Table 14:** 1-$\sigma$ errors for $\beta ^{2}$, for CMB, *P*(*k*), WL and $\hbox {CMB}+P(k)+\hbox {WL}$

Fixed parameter	CMB	*P*(*k*)	WL	$\hbox {CMB}+P(k)+\hbox {WL}$
(Marginalized on all params)	0.0094	0.0015	0.012	0.00032
$\alpha$	0.0093	0.00085	0.0098	0.00030
$\varOmega _{c}$	0.0026	0.00066	0.0093	0.00032
*h*	0.0044	0.0013	0.011	0.00032
$\varOmega _{b}$	0.0087	0.0014	0.012	0.00030
$n_{s}$	0.0074	0.0014	0.012	0.00028
$\sigma _{8}$	0.0094	0.00084	0.0053	0.00030
$\log (A)$	0.0090	0.0015	0.012	0.00032

For each line, only the parameter in the left column has been fixed to the reference value. The first line corresponds to the case in which we have marginalized over all parameters. Table reproduced by permission from Amendola et al. (2011), copyright by APS

#### Forecasts for the anisotropic stress parameter $\eta$

One problem, encountered in trying to constrain the MG theoretical parameters *Q*(*a*, *k*) and $\eta (a,k)$ introduced earlier, is that one has to assume, or parametrize, the initial fluctuation power spectrum and its evolution until the epoch at which observations are made. This is of course fine if one assumes a standard cosmology until dark energy begins driving the expansion, but not necessarily so if dark energy, or any other non-standard process, is active also in the past. In this section, we examine briefly how can one perform a test of the anisotropic stress parameter $\eta$ that is independent of the shape and the volution of the power spectrum.

In Amendola et al. (2013a) it has been shown that lensing, clustering, and redshift distortion measurements at linear scales can determine, in addition to the expansion rate *H*(*z*), only three additional variables *R*, *A* and *L*, generally function of space and time, that are independent of the power spectrum shape. They are given by

I.8.38 $$\begin{aligned} A&=Gb\delta _{\text {m,0}},\qquad R=Gf\delta _{\text {m,0}},\nonumber \\ L&=\varOmega _{\text {m,0}}GQ(1+1/\eta )\delta _{\text {m,0}}. \end{aligned}$$

where the subscript 0 denotes present time and, as usual, *G* is the growth factor normalized to unity today, *f* is the growth rate and *b* is the bias: all these functions can in principle freely depend on time and space. The factor $\delta _{\text {t},0}$ represents the square root of the variance of todays’ fluctuations. As anticipated, its shape depends on processes that are known only in standard cosmologies.

In order to reconstruct $\eta$ from *A*, *R* and *L* it is necessary therefore to remove the dependence on $\delta _{\text {t},0}$. This can be done by considering ratios like $P_{1}=R/A$, $P_{2}=L/R$ and $P_{3}=R'/R$ (prime is derivative with respect to *e*-folding time). All other possible combinations can be obtained from these quantities. In terms of these model-independent ratios, and assuming that beside dark energy one has only pressureless matter (this assumption can easily generalized) the gravitational slip becomes (Amendola et al. 2013a; Motta et al. 2013)

I.8.39 $$\begin{aligned} 1+\eta =\frac{3P_{2}(1+z)^{3}}{2E^{2}\left( P_{3}+2+\frac{E'}{E}\right) } \end{aligned}$$

where we also set $E(z)\equiv H(z)/H_{0}$. This expression gives then the theoretical quantity $\eta (a,k)$ as a direct function of the observables, without the need of additional assumptions on initial conditions beyond the region effectively observed.

The function $\eta$ can assume in principle any form, but if one confine themselves to single scalar fields with second-order equation of motion or to bimetric gravity, then the relatively simple form (I.4.8) holds true. Then, Eq. (I.8.39) can test a vast class of models at once. In Amendola et al. (2014), the forecasts for $\eta$ have been performed for a Euclid-like survey and assuming a LSST-like amount of supernovae Ia. The result is that $\eta$ can be measured to within 1–2% when assumed constant in redshift and space and to within 10%, roughly, when varying only in redshift, while the error rapidly degrades when assuming a more general form like Eq. (I.4.8).

#### Extra-Euclidean data and priors

In addition to the baseline Euclid surveys, a possibility may exist for an auxiliary Euclid survey, for example focused on Type Ia supernovae. Type Ia supernovae used as standardized candles (luminosity distance indicators) led to the discovery of cosmic acceleration and they retain significant leverage for revealing the nature of dark energy. Their observed flux over the months after explosion (the light curve) is calibrated by an empirical brightness–light curve width relation into a luminosity distance multiplied by a factor involving the unknown absolute brightness and Hubble constant. This nuisance factor cancels when supernovae at different redshifts are used as a relative distance measure. The relative distance is highly sensitive to cosmic acceleration and provides strong complementarity with other cosmological probes of dark energy, at the same or different redshifts.

Another advantageous property of supernovae is their immunity to systematics from cosmology theory—they are purely geometric measures and do not care about the matter power spectrum, coupling to dark matter, cosmologically modified gravity, dark energy clustering, etc. Their astrophysical systematics are independent of other probes, giving important crosschecks. The cosmological parameter likelihood function arising from supernovae constraints can to a good approximation simply be multiplied with the likelihood from other probes. Current supernovae likelihoods are in user friendly form from the joint lightcurve analysis (JLA) of the supernova legacy survey (SNLS) and sloan digital sky survey (SDSS) of Betoule et al. (2014) or the Union2.1 compilation of Suzuki et al. (2012). In the near future the Union3 compilation should merge these sets and all other current supernova data, within an improved Bayesian framework.

The Euclid Supernovae Science Working Group proposed a six month auxiliary survey with Euclid, the Dark Energy Supernova InfraRed Experiment (DESIRE) (Astier et al. 2014). This delivers substantial improvements on dark energy equation of state constraints relative to ground-based supernova surveys, with a 50% higher figure of merit, as shown in Table 15.

**Table 15:** Cosmological performance of the simulated surveys

	${\varvec{\sigma }}({\varvec{w}}_{\varvec{a}})$	${\varvec{z}}_{\varvec{p}}$	${\varvec{\sigma }}({\varvec{w}}_{\varvec{p}})$	FoM
$\hbox {low-z} + \hbox {LSST-DDF} + \hbox {DESIRE}$	0.22	0.25	0.022	203.2
$\hbox {low-z} + \hbox {LSST-DDF}$	0.28	0.22	0.026	137.1
$\hbox {LSST-DDF} + \hbox {DESIRE}$	0.40	0.35	0.031	81.4

The FoMs assume a 1-D geometrical *Planck* prior and flatness. $z_p$ is the redshift at which the equation of state uncertainty reaches its minimum $\sigma (w_p)$. The FoM is defined as $[Det(Cov(w_0, w_a))]^{-1/2}= [\sigma (w_a)\sigma (w_p)]^{-1}$ and accounts for a suite of systematic uncertainties (see Astier et al. 2014)

Other dark-energy projects will enable the cross-check of the dark-energy constraints from Euclid. These include Planck, BOSS, WiggleZ, HETDEX, DES, Panstarrs, LSST, BigBOSS and SKA.

Planck will provide exquisite constraints on cosmological parameters, but not tight constraints on dark energy by itself, as CMB data are not sensitive to the nature of dark energy (which has to be probed at $z<2$, where dark energy becomes increasingly important in the cosmic expansion history and the growth history of cosmic large scale structure). Planck data in combination with Euclid data provide powerful constraints on dark energy and tests of gravity. In the next Sect. I.8.10.1, we will discuss how to create a Gaussian approximation to the Planck parameter constraints that can be combined with Euclid forecasts in order to model the expected sensitivity.

The galaxy redshift surveys BOSS, WiggleZ, HETDEX, and BigBOSS are complementary to Euclid, since the overlap in redshift ranges of different galaxy redshift surveys, both space and ground-based, is critical for understanding systematic effects such as bias through the use of multiple tracers of cosmic large scale structure. Euclid will survey H$\alpha$ emission line galaxies at $0.5< z < 2.0$ over 15,000 square degrees. The use of multiple tracers of cosmic large scale structure can reduce systematic effects and ultimately increase the precision of dark-energy measurements from galaxy redshift surveys (see, e.g., Seljak et al. 2009).

Currently on-going or recently completed surveys which cover a sufficiently large volume to measure BAO at several redshifts and thus have science goals common to Euclid, are the Sloan Digital Sky Survey III Baryon Oscillations Spectroscopic Survey (BOSS for short) and the WiggleZ survey.

BOSS^13^ maps the redshifts of 1.5 million Luminous Red Galaxies (LRGs) out to $z\sim 0.7$ over 10,000 square degrees, measuring the BAO signal, the large-scale galaxy correlations and extracting information of the growth from redshift space distortions. A simultaneous survey of $2.2< z < 3.5$ quasars measures the acoustic oscillations in the correlations of the Lyman-$\alpha$ forest. LRGs were chosen for their high bias, their approximately constant number density and, of course, the fact that they are bright. Their spectra and redshift can be measured with relatively short exposures in a 2.4 m ground-based telescope. The data-taking of BOSS will end in 2014.

The WiggleZ^14^ survey is now completed, it measured redshifts for almost 240,000 galaxies over 1000 square degrees at $0.2<z<1$. The target are luminous blue star-forming galaxies with spectra dominated by patterns of strong atomic emission lines. This choice is motivated by the fact that these emission lines can be used to measure a galaxy redshift in relatively short exposures of a 4-m class ground-based telescope.

Red quiescent galaxies inhabit dense clusters environments, while blue star-forming galaxies trace better lower density regions such as sheets and filaments. It is believed that on large cosmological scales these details are unimportant and that galaxies are simply tracers of the underlying dark matter: different galaxy type will only have a different ‘bias factor’. The fact that so far results from BOSS and WiggleZ agree well confirms this assumption.

Between now and the availability of Euclid data other wide-field spectroscopic galaxy redshift surveys will take place. Among them, eBOSS will extend BOSS operations focusing on 3100 square degrees using a variety of tracers. Emission line galaxies will be targeted in the redshift window $0.6<z<1$. This will extend to higher redshift and extend the sky coverage of the WiggleZ survey. Quasars in the redshift range $1<z<2.2$ will be used as tracers of the BAO feature instead of galaxies. The BAO LRG measurement will be extended to $z \sim 0.8$, and the quasar number density at $z>2.2$ of BOSS will be tripled, thus improving the BAO Lyman-$\alpha$ forest measure.

HETDEX aims at surveying 1 million Lyman-$\alpha$ emitting galaxies at $1.9< z < 3.5$ over 420 square degrees. The main science goal is to map the BAO feature over this redshift range.

Further in the future, we highlight here the proposed BigBOSS survey and SuMIRe survey with HyperSupremeCam on the Subaru telescope. The BigBOSS survey will target [OII] emission line galaxies at $0.6< z < 1.5$ (and LRGs at $z < 0.6$) over 14,000 square degrees. The SuMIRe wide survey proposes to survey $\sim \, 2000$ square degrees in the redshift range $0.6<z<1.6$ targeting LRGs and [OII] emission-line galaxies. Both these surveys will likely reach full science operations roughly at the same time as the Euclid launch.

Wide field photometric surveys are also being carried out and planned. The ongoing Dark Energy Survey (DES)^15^ will cover 5000 square degrees out to $z\sim 1.3$ and is expected to complete observations in 2017; the Panoramic Survey Telescope and Rapid Response System (Pan-STARRS), on-going at the single-mirror stage, The PanSTARSS survey, which first phase is already on-going, will cover 30,000 square degrees with 5 photometry bands for redshifts up to $z\sim 1.5$. The second pause of the survey is expected to be competed by the time Euclid launches. More in the future the Large Synoptic Survey Telescope (LSST) will cover redshifts $0.3<z<3.6$ over 15,000 square degrees, but is expected to begin operations in 2021, after Euclid’s planned launch date. The galaxy imaging surveys DES, Panstarrs, and LSST will complement Euclid imaging survey in both the choice of band passes, and the sky coverage.

SKA (which is expected to begin operations in 2020 and reach full operational capability in 2024) will survey neutral atomic hydrogen (HI) through the radio 21 cm line, over a very wide area of the sky. It is expected to detect HI emitting galaxies out to $z\sim 1.5$ making it nicely complementary to Euclid. Such galaxy redshift survey will of course offer the opportunity to measure the galaxy power spectrum (and therefore the BAO feature) out to $z \sim 1.5$. The well behaved point spread function of a synthesis array like the SKA should ensure superb image quality enabling cosmic shear to be accurately measured and tomographic weak lensing used to constrain cosmology and in particular dark energy. This weak lensing capability also makes SKA and Euclid very complementary. For more information see, e.g., Rawlings et al. (2004) and Blake et al. (2004).

**Fig. 35:**
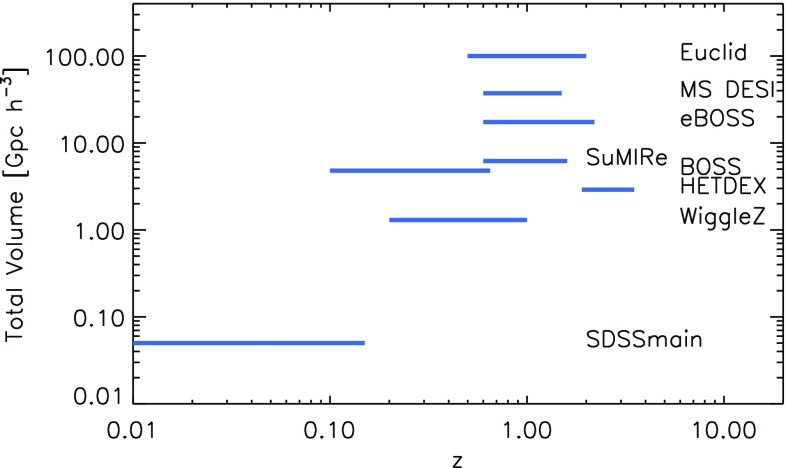
Redshift coverage and volume for the surveys mentioned in the text. Spectroscopic surveys only are shown. Recall that while future and forthcoming photometric surveys focus on weak gravitational lensing, spectroscopic surveys can extract the three dimensional galaxy clustering information and therefore measure radial and tangential BAO signal, the power spectrum shape and the growth of structure via redshift space distortions. The three-dimensional clustering information is crucial for BAO. For example to obtain the same figure of merit for dark-energy properties a photometric survey must cover a volume roughly ten times bigger than a spectroscopic one

Figure 35 puts Euclid into context. Euclid will survey H$\alpha$ emission line galaxies at $0.5< z < 2.0$ over 15,000 square degrees. Clearly, Euclid with both spectroscopic and photometric capabilities and wide field coverage surpasses all surveys that will be carried out by the time it is launched. The large volume surveyed is crucial as the number of modes to sample for example the power spectrum and the BAO feature scales with the volume. The redshift coverage is also important especially at $z<2$ where the dark-energy contribution to the density of the universe is non-negligible (at $z>2$ for most cosmologies the universe is effectively Einstein–de Sitter, therefore, high redshifts do not contribute much to constraints on dark energy). Having a single instrument, a uniform target selection and calibration is also crucial to perform precision tests of cosmology without having to build a ‘ladder’ from different surveys selecting different targets. On the other hand it is also easy to see the synergy between these ground-based surveys and Euclid: by mapping different targets (over the same sky area and ofter the same redshift range) one can gain better control over issues such as bias. The use of multiple tracers of cosmic large scale structure can reduce systematic effects and ultimately increase the precision of dark-energy measurements from galaxy redshift surveys (see, e.g., Seljak et al. 2009).

Moreover, having both spectroscopic and imaging capabilities Euclid is uniquely poised to explore the clustering with both the three dimensional distribution of galaxies and weak gravitational lensing.

*I.8.10.1 The Planck prior*

Planck will provide highly accurate constraints on many cosmological parameters, which makes the construction of a Planck Fisher matrix somewhat non-trivial as it is very sensitive to the detailed assumptions. A relatively robust approach was used by Mukherjee et al. (2008) to construct a Gaussian approximation to the WMAP data by introducing two extra parameters,

I.8.40 $$\begin{aligned} R \equiv \sqrt{\varOmega _m H_0^2} \,r(z_{\mathrm {CMB}}), \qquad l_a \equiv \pi r(z_{\mathrm {CMB}})/r_s(z_{\mathrm {CMB}}), \end{aligned}$$

where *r*(*z*) is the comoving distance from the observer to redshift *z*, and $r_s(z_{\mathrm {CMB}})$ is the comoving size of the sound-horizon at decoupling.

In this scheme, $l_a$ describes the peak location through the angular diameter distance to decoupling and the size of the sound horizon at that time. If the geometry changes, either due to non-zero curvature or due to a different equation of state of dark energy, $l_a$ changes in the same way as the peak structure. *R* encodes similar information, but in addition contains the matter density which is connected with the peak height. In a given class of models (for example, quintessence dark energy), these parameters are “observables” related to the shape of the observed CMB spectrum, and constraints on them remain the same independent of (the prescription for) the equation of state of the dark energy.

As a caveat we note that if some assumptions regarding the evolution of perturbations are changed, then the corresponding *R* and $l_a$ constraints and covariance matrix will need to be recalculated under each such hypothesis, for instance, if massive neutrinos were to be included, or even if tensors were included in the analysis (Corasaniti and Melchiorri 2008). Further, *R* as defined in Eq. (I.8.40) can be badly constrained and is quite useless if the dark energy clusters as well, e.g., if it has a low sound speed, as in the model discussed in Kunz (2009).

In order to derive a Planck fisher matrix, Mukherjee et al. (2008) simulated Planck data as described in Pahud et al. (2006) and derived constraints on our base parameter set $\{R,l_a,\varOmega _b h^2,n_s\}$ with a MCMC based likelihood analysis. In addition to *R* and $l_a$ they used the baryon density $\varOmega _bh^2$, and optionally the spectral index of the scalar perturbations $n_s$, as these are strongly correlated with *R* and $l_a$, which means that we will lose information if we do not include these correlations. As shown in Mukherjee et al. (2008), the resulting Fisher matrix loses some information relative to the full likelihood when only considering Planck data, but it is very close to the full analysis as soon as extra data is used. Since this is the intended application here, it is perfectly sufficient for our purposes.

The following tables, from Mukherjee et al. (2008), give the covariance matrix for quintessence-like dark energy (high sound speed, no anisotropic stress) on the base parameters and the Fisher matrix derived from it. Please consult the appendix of that paper for the precise method used to compute *R* and $l_a$ as the results are sensitive to small variations.

**Table 16:** *R*, $l_a$, $\varOmega _bh^2$ and $n_s$ estimated from Planck simulated data

Parameter	mean	rms variance
$$\varOmega _k\ne 0$$		
*R*	1.7016	0.0055
$l_a$	302.108	0.098
$\varOmega _b h^2$	0.02199	0.00017
$n_s$	0.9602	0.0038

Table reproduced by permission from Mukherjee et al. (2008), copyright by APS

**Table 17:** Covariance matrix for $(R, l_a, \varOmega _b h^2, n_s)$ from Planck

	*R*	$l_a$	$\varOmega _b h^2$	$n_s$
$$\varOmega _k\ne 0$$				
*R*	0.303492E–04	0.297688E–03	$-$0.545532E–06	$-$0.175976E–04
$l_a$	0.297688E–03	0.951881E–02	$-$0.759752E–05	$-$0.183814E–03
$\varOmega _b h^2$	$-$0.545532E–06	$-$0.759752E-05	0.279464E–07	0.238882E–06
$n_s$	$-$0.175976E–04	$-$0.183814E-03	0.238882E–06	0.147219E–04

Table reproduced by permission from Mukherjee et al. (2008), copyright by APS

**Table 18:** Fisher matrix for ($w_0$, $w_a$, $\varOmega _{\mathrm {DE}}$, $\varOmega _k$, $\omega _m$, $\omega _b$, $n_S$) derived from the covariance matrix for $(R, l_a, \varOmega _b h^2, n_s)$ from Planck

	$w_0$	$w_a$	$\varOmega _{\mathrm {DE}}$	$\varOmega _k$	$\omega _m$	$\omega _b$	$n_S$
$w_0$	.172276E$+$06	.490320E$+$05	.674392E$+$06	$-$.208974E$+$07	.325219E$+$07	$-$.790504E$+$07	$-$.549427E$+$05
$w_a$	.490320E$+$05	.139551E$+$05	.191940E$+$06	$-$.594767E$+$06	.925615E$+$06	$-$.224987E$+$07	$-$.156374E$+$05
$\varOmega _{\mathrm {DE}}$	.674392E$+$06	.191940E$+$06	.263997E$+$07	$-$.818048E$+$07	.127310E$+$08	$-$.309450E$+$08	$-$.215078E$+$06
$\varOmega _k$	$-$.208974E$+$07	$-$.594767E$+$06	$-$.818048E$+$07	.253489E$+$08	$-$.394501E$+$08	.958892E$+$08	.666335E$+$06
$\omega _m$	.325219E$+$07	.925615E$+$06	.127310E$+$08	$-$.394501E$+$08	.633564E$+$08	$-$.147973E$+$09	$-$.501247E$+$06
$\omega _b$	$-$.790504E$+$07	$-$.224987E$+$07	$-$.309450E$+$08	.958892E$+$08	$-$.147973E$+$09	.405079E$+$09	.219009E$+$07
$n_S$	$-$.549427E$+$05	$-$.156374E$+$05	$-$.215078E$+$06	.666335E$+$06	$-$.501247E$+$06	.219009E$+$07	.242767E$+$06

Table reproduced by permission from Mukherjee et al. (2008), copyright by APS

#### Forecasts for model independent observations

As discussed in Sect. I.7.6, it is worth to complement the standard *P*(*k*) analysis, see Eq. (I.7.31), with the $C_{\ell }(z_1,z_2)$ method, which involves the directly observable redshift and angular separations instead of reconstructed model-dependent comoving distances. The full relativistic expression of the redshift dependent angular power spectra of galaxy number counts, $C_{\ell }(z_1,z_2)$, which holds for any theory of gravity whose metric can be written as in Eq. (I.7.2) and in which photons and dark matter particles move along geodesics, is given in Bonvin and Durrer (2011) and Di Dio et al. (2013). In particular, it includes the lensing contribution [see Eq. (I.7.17)] and redshift-space distortions due to peculiar velocities, see Kaiser (1987), as well as other terms depending on the gravitational potentials.

The Fisher matrix is discussed, e.g., in Di Dio et al. (2013) and Eq. (I.7.31) is replaced by:

I.8.41 $$\begin{aligned} F_{\alpha \beta } = \sum _{\ell ,(ij),(pq)} \frac{\partial C_\ell ^{ij} }{\partial p_\alpha } \frac{\partial C_\ell ^{pq}}{\partial p_\beta } \text {Cov}^{-1}_{\ell , (ij), (pq)}, \end{aligned}$$

where $C_\ell ^{ij}$ is the correlation between redshift bin $z_i$ and $z_j$, and the covariance matrix between different power spectra can be approximated as:

I.8.42 $$\begin{aligned} \text {Cov}_{[\ell ,\ell '] [(ij), (pq)]}=\delta _{\ell ,\ell '}\frac{C_\ell ^{\text {obs},i p} C_\ell ^{\text {obs},jq} + C_\ell ^{\text {obs},i q} C_\ell ^{\text {obs},jp}}{f_\text {sky} \left( 2 \ell + 1 \right) }. \end{aligned}$$

The observable power spectrum $C_\ell ^{\text {obs},ij}$ takes into account the fact that we observe a finite number of galaxies instead of a smooth field. This leads to a shot noise term

I.8.43 $$\begin{aligned} C_\ell ^{\text {obs},ij}= C_\ell ^{ij} + \frac{\delta _{ij}}{ N(i)}, \end{aligned}$$

where *N*(*i*) denotes the number of galaxies in the bin around $z_i$. The power spectra, using $\varDelta _{{\mathcal {R}}}^2 =k^3P_\mathcal{R}/(2\pi ^2)$, see Eq. (I.7.30), given by

I.8.44 $$\begin{aligned} C_\ell ^{ij} = 4 \pi \int \frac{dk}{k} \varDelta _{{\mathcal {R}}}^2(k) \varDelta ^i_\ell (k)\varDelta ^j_\ell (k)~, \end{aligned}$$

are computed in terms of integrals of transfer functions $\varDelta _\ell (z,k)$:

I.8.45 $$\begin{aligned} \varDelta _\ell ^i(k) = \int dz \frac{dN}{dz} W_i(z) \varDelta _\ell (z,k)~, \end{aligned}$$

which account for the tracer distribution $\frac{dN}{dz}$ and the bin selection function $W_i(z)$. This can be approximated by a top hat if good redshift estimates are available, or by a Gaussian with standard deviation determined by the photometric redshift errors. The power spectra are computed, e.g., by the publicly available code classgal described in Di Dio et al. (2013).

**Fig. 36:**
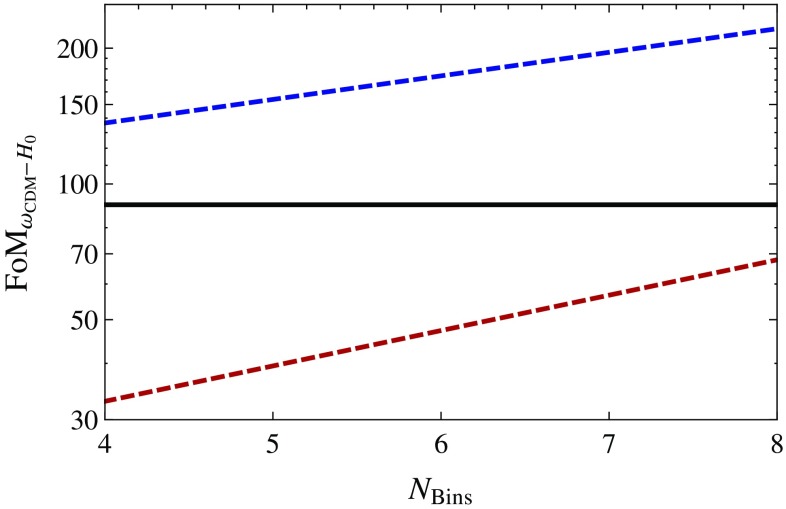
We show the figure of merit for $\omega _{CDM}=h^2\varOmega _{CDM}$ and $H_0$ as a function of the number of bins for the photometric survey of Euclid. The black line is the *P*(*k*) result, the red dashed line is the $C_l(z_1,z_2)$ result for bin auto-correlations only, while the blue line also includes cross-correlations

For photometric redshifts, the model independent $C_{\ell }(z_1,z_2)$ method performs significantly better then the standard *P*(*k*) analysis (Di Dio et al. 2014) as can be seen in Fig. 36.

This is due to the fact that this analysis makes optimal use of the redshift information and does not average over directions. For spectroscopic redshifts, however, the large number of redshift bins which would be needed to fully profit from the redshift information, is severely limited by shot noise. In fact when using redshift bins that are significantly thicker than the redshift resolution of the survey, the *P*(*k*) analysis, in principle, has an advantage since it makes use of the full redshift resolution in determining distances of galaxies, while in the $C_{\ell }(z_1,z_2)$ analysis we do not distinguish redshifts of galaxies in the same bin. However, for spectroscopic surveys we can in principle allow for very slim bins with a thickness significantly smaller than the nonlinearity scale, and the maximal number of useful bins is decided by the shot noise, as well as by numerical limitations related to Markov Chain Monte Carlo data analysis.

The cross correlations from different redshift bins provide an alternative measure of the lensing potential (Montanari and Durrer 2015), which is complementary to the analysis of shear with completely different systematic errors. This will allow the measurement of $\langle \delta (z_1)\kappa (z_2)\rangle$ for $z_2>z_1$.

### Summary and outlook

This section introduced the main features of the most popular dark energy/modified gravity models. Here we summarize the performance of Euclid with respect to these models. Unless otherwise indicated, we always assume Euclid with no external priors and all errors fully marginalized over the standard cosmological parameters. Here RS denotes the redshift survey, WLS the weak lensing one (Tables 16, 17, 18).

1. Euclid (RS) should be able to measure the main standard cosmological parameters to percent or sub-percent level as detailed in Table 7 (all marginalized errors, including constant equation of state and constant growth rate, see Table 11 and Fig. 24).
2. The two CPL parameters $w_0,w_1$ should be measured with errors 0.06 and 0.26, respectively (fixing the growth rate to fiducial), see Table 11 and Fig. 24.
3. The equation of state *w* and the growth rate parameter $\gamma$, both assumed constant, should be simultaneously constrained to within 0.04 and 0.03, respectively.
4. The growth function should be constrained to within 0.01–0.02 for each redshift bin from $z=0.7$ to $z=2$ (see Table 4).
5. A scale-independent bias function *b*(*z*) should be constrained to within 0.02 for each redshift bin (see Table 4).
6. The growth rate parameters $\gamma _0,\gamma _1$ defined in Eq. (I.8.5) should be measured to within 0.08, 0.17, respectively.
7. Euclid will achieve an accuracy on measurements of the dark energy sound speed of $\sigma (c_s^2)/c_s^2=2615$ (WLS) and $\sigma (c_s^2)/c_s^2=50.05$ (RS), if $c_s^2=1$, or $\sigma (c_s^2)/c_s^2=0.132$ (WLS) and $\sigma (c_s^2)/c_s^2=0.118$ (RS), if $c_s^2=10^{-6}$.
8. The coupling $\beta ^{2}$ between dark energy and dark matter can be constrained by Euclid (with Planck) to less than $3\times 10^{-4}$ (see Fig. 34 and Table 13).
9. Any departure from GR greater than $\simeq 0.03$ in the growth index $\gamma$ will be distinguished by the WLS (Heavens et al. 2007).
10. Euclid WLS can detect deviations between 3 and 10% from the GR value of the modified-gravity parameter $\varSigma$ (Eq. I.3.28), whilst with the RS there will be a 20% accuracy on both $\varSigma$ and $\mu$ (Eq. I.3.27).
11. With the WLS, Euclid should provide an upper limit to the present dimensionless scalaron inverse mass $\mu \equiv H_{0}/M_{0}$ of the *f*(*R*) scalar [where the time dependent scalar field mass is defined in Eq. (I.8.37)] as $\mu = 0.00 \pm 1.10\times 10^{-3}$ for $l < 400$ and $\mu = 0.0 \pm 2.10 \times 10^{-4}$ for $l < 10{,}000$
12. The WLS will be able to rule out the DGP model growth index with a Bayes factor $|\ln B|\simeq 50$ (Heavens et al. 2007), and viable phenomenological extensions could be detected at the $3\sigma$ level for $1000\lesssim \ell \lesssim 4000$ (Camera et al. 2011a).
13. The photometric survey of Euclid, i.e., the WLS, is very promising in measuring directly observable angular and redshift dependent power spectra $C_{\ell }(z_1,z_2)$ (and correlation function) as discussed in Di Dio et al. (2014). This spectra are truly model independent and especially well suited to estimate cosmological parameter or test models of modified gravity.

At the same time, there are several areas of research that we feel are important for the future of Euclid, both to improve the current analyses and to maximize its science return. Here we provide a preliminary, partial list.

1. The results of the redshift survey and weak lensing surveys should be combined in a statistically coherent way
2. The set of possible priors to be combined with Euclid data should be better defined
3. The forecasts for the parameters of the modified gravity and clustered dark-energy models should be extended to include more general cases
4. We should estimate the errors on a general reconstruction of the modified gravity functions $\varSigma ,\mu$ or of the metric potentials $\varPsi ,\varPhi$ as a function of both scale and time.
5. We should use the $C_\ell (z_1,z_2)$-method to constrain modified gravity models.

## Part II Dark matter and neutrinos

### Introduction

The identification of dark matter is one of the most important open problems in particle physics and cosmology. In standard cosmology, dark matter contributes 85% of all the matter in the universe, but we do not know what it is made of, as we have never observed dark matter particles in our laboratories. The foundations of the modern dark matter paradigm were laid in the 1970s and 1980s, after decades of slow accumulation of evidence. Back in the 1930s, it was noticed that the Coma cluster seemed to contain much more mass than what could be inferred from visible galaxies (Zwicky 1933, 1937), and a few years later, it became clear that the Andromeda galaxy M31 rotates anomalously fast at large radii, as if most of its mass resides in its outer regions. Several other pieces of evidence provided further support to the dark matter hypothesis, including the so called timing-argument. In the 1970s, rotation curves were extended to larger radii and to many other spiral galaxies, proving the presence of large amounts of mass on scales much larger than the size of galactic disks (Peacock 1999).

We are now in the position of determining the total abundance of dark matter relative to normal, baryonic matter, in the universe with exquisite accuracy; we have a much better understanding of how dark matter is distributed in structures ranging from dwarf galaxies to clusters of galaxies, thanks to gravitational lensing observations (see Massey et al. 2010, for a review) and theoretically from high-resolution numerical simulations made possible by modern supercomputers (such as, for example, the Millennium or Marenostrum simulations).

Originally, Zwicky thought of dark matter as most likely baryonic—missing cold gas, or low mass stars. Rotation curve observation could be explained by dark matter in the form of MAssive Compact Halo Objects (MACHOs, e.g., a halo of black holes or brown dwarfs). However, the MACHO and EROS experiments have shown that dark matter cannot be in the mass range $0.6\times 10^{-7}\,M_{\odot }<M<15\,M_{\odot }$ if it comprises massive compact objects (Alcock et al. 2000; Tisserand et al. 2007). Gas measurements are now extremely sensitive, ruling out dark matter as undetected gas (Bi and Davidsen 1997; Choudhury et al. 2001; Richter et al. 2006; but see Pfenniger et al. 1994). And the CMB and Big Bang Nucleosynthesis require the total mass in baryons in the universe to be significantly less that the total matter density (Rebolo 2002; Coc et al. 2002; Turner 2002).

This is one of the most spectacular results in cosmology obtained at the end of the 20th century: dark matter has to be non-baryonic. As a result, our expectation of the nature of dark matter shifted from an astrophysical explanation to particle physics, linking the smallest and largest scales that we can probe.

During the seventies the possibility of the neutrino to be the dark matter particle with a mass of tenth of eV was explored, but it was realized that such light particle would erase the primordial fluctuations on small scales, leading to a lack of structure formation on galactic scales and below. It was therefore postulated that the dark matter particle must be cold (low thermal energy, to allow structures on small scale to form), collisionless (or have a very low interaction cross section, because dark matter is observed to be pressureless) and stable over a long period of time: such a candidate is referred to as a weakly interacting massive particle (WIMP). This is the standard cold dark matter (CDM) picture (see Frenk et al. 1990; Peebles et al. 1991).

Particle physicists have proposed several possible dark matter candidates. Supersymmetry (SUSY) is an attractive extension of the Standard Model of particle physics. The lightest SUSY particle (the LSP) is stable, uncharged, and weakly interacting, providing a perfect WIMP candidate known as a neutralino. Specific realizations of SUSY each provide slightly different dark matter candidates (for a review see Jungman et al. 1996). Another distinct dark matter candidate arising from extensions of the Standard Model is the axion, a hypothetical pseudo-Goldstone boson whose existence was postulated to solve the so called strong *CP* problem in quantum chromodynamics (Peccei and Quinn 1977), also arising generically in string theory (Witten 1984; Svrcek and Witten 2006). They are known to be very well motivated dark matter candidates (for a review of axions in cosmology see Sikivie 2008). Other well-known candidates are sterile neutrinos, which interact only gravitationally with ordinary matter, apart from a small mixing with the familiar neutrinos of the Standard Model (which should make them ultimately unstable), and candidates arising from technicolor (see, e.g., Gudnason et al. 2006). A wide array of other possibilities have been discussed in the literature, and they are currently being searched for with a variety of experimental strategies (for a complete review of dark matter in particle physics see Amsler et al. 2008).

There remain some possible discrepancies in the standard cold dark matter model, such as the missing satellites problem, and the cusp-core controversy (see below for details and references) that have led some authors to question the CDM model and to propose alternative solutions. The physical mechanism by which one may reconcile the observations with the standard theory of structure formation is the suppression of the matter power spectrum at small scales. This can be achieved with dark matter particles with a strong self-scattering cross section, or with particles with a non-negligible velocity dispersion at the epoch of structure formation, also referred to as warm dark matter (WDM) particles.

Another possibility is that the extra gravitational degrees of freedom arising in modified theories of gravity play the role of dark matter. In particular this happens for the Einstein-Aether, TeVeS and bigravity models. These theories were developed following the idea that the presence of unknown dark components in the universe may be indicating us that it is not the matter component that is exotic but rather that gravity is not described by standard GR.

Finally, we note that only from astrophysical probes can any dark matter candidate found in either direct detection experiments or accelerators, such as the LHC, be confirmed. Any direct dark matter candidate discovery will give Euclid a clear goal to verify the existence of this particle on astrophysical scales. Within this context, Euclid can provide precious information on the nature of dark matter. In this part, we discuss the most relevant results that can be obtained with Euclid, and that can be summarized as follows:

- The discovery of an exponential suppression in the power spectrum at small scales, that would rule out CDM and favor WDM candidates, or, in absence of it, the determination of a lower limit on the mass of the WDM particle, $m_{\mathrm {WDM}}$, of 2 keV;
- the determination of an upper limit on the dark matter self-interaction cross section $\sigma /m\sim 10^{-27}\mathrm {\ cm^2\ GeV^{-1}}$ at 68% CL, which represents an improvement of three orders of magnitude compared to the best constraint available today, which arises from the analysis of the dynamics of the bullet cluster;
- the measurement of the slope of the dark matter distribution within galaxies and clusters of galaxies with unprecedented accuracy;
- the determination of the properties of the only known—though certainly subdominant—non-baryonic dark matter particle: the standard neutrino, for which Euclid can provide information on the absolute mass scale, its normal or inverted hierarchy, as well as its Dirac or Majorana nature;
- the test of unified dark matter (UDM, or quartessence) models, through the detection of characteristic oscillatory features predicted by these theories on the matter power spectrum, detectable through weak lensing or baryonic acoustic oscillations studies;
- a probe of the axiverse, i.e., of the legacy of string theory through the presence of ultra-light scalar fields that can affect the growth of structure, introducing features in the matter power spectrum and modifying the growth rate of structures.

Finally, Euclid will provide, through gravitational lensing measurement, a map of the dark matter distribution over the entire extragalactic sky, allowing us to study the effect of the dark matter environment on galaxy evolution and structure formation as a function of time. This map will pinpoint our place within the dark universe.

### Dark matter halo properties

Dark matter was first proposed by Zwicky (1937) to explain the anomalously high velocity of galaxies in galaxy clusters. Since then, evidence for dark matter has been accumulating on all scales. The velocities of individual stars in dwarf galaxies suggest that these are the most dark matter dominated systems in the universe (e.g., Mateo 1998; Kleyna et al. 2001; Simon and Geha 2007; Martin et al. 2007; Walker et al. 2007). Low surface brightness (LSB) and giant spiral galaxies rotate too fast to be supported by their stars and gas alone, indicating the presence of dark matter (de Blok et al. 2001; Simon et al. 2005; Borriello and Salucci 2001; Klypin et al. 2002). Gravitationally lensed giant elliptical galaxies and galaxy clusters require dark matter to explain their observed image distributions (e.g., Refsdal 1964; Bourassa and Kantowski 1975; Walsh et al. 1979; Soucail et al. 1987; Clowe et al. 2006a). Finally, the temperature fluctuations in the cosmic microwave background (CMB) radiation indicate the need for dark matter in about the same amount as that required in galaxy clusters (e.g., Smoot et al. 1992; Wright et al. 1992; Spergel et al. 2007).

While the case for particle dark matter is compelling, until we find direct evidence for such a particle, astrophysics remains a unique dark matter probe. Many varieties of dark matter candidates produce a noticeable change in the growth of structure in the universe (Jungman et al. 1996; Steffen 2009). Warm dark matter (WDM) suppresses the growth of structure in the early universe producing a measurable effect on the small-scale matter power spectrum (Bode et al. 2001; Avila-Reese et al. 2001; Barkana et al. 2001). Self-interacting dark matter (SIDM) changes the expected density distribution *within* bound dark matter structures (Dalcanton and Hogan 2001; Hogan and Dalcanton 2000). In both cases, the key information about dark matter is contained on very small scales. In this section, we discuss previous work that has attempted to measure the small scale matter distribution in the universe, and discuss how Euclid will revolutionize the field. We divide efforts into three main areas: measuring the halo mass function on large scales, but at high redshift; measuring the halo mass function on small scales through lens *substructures*; measuring the dark matter density profile within galaxies and galaxy clusters.

**Fig. 37:**
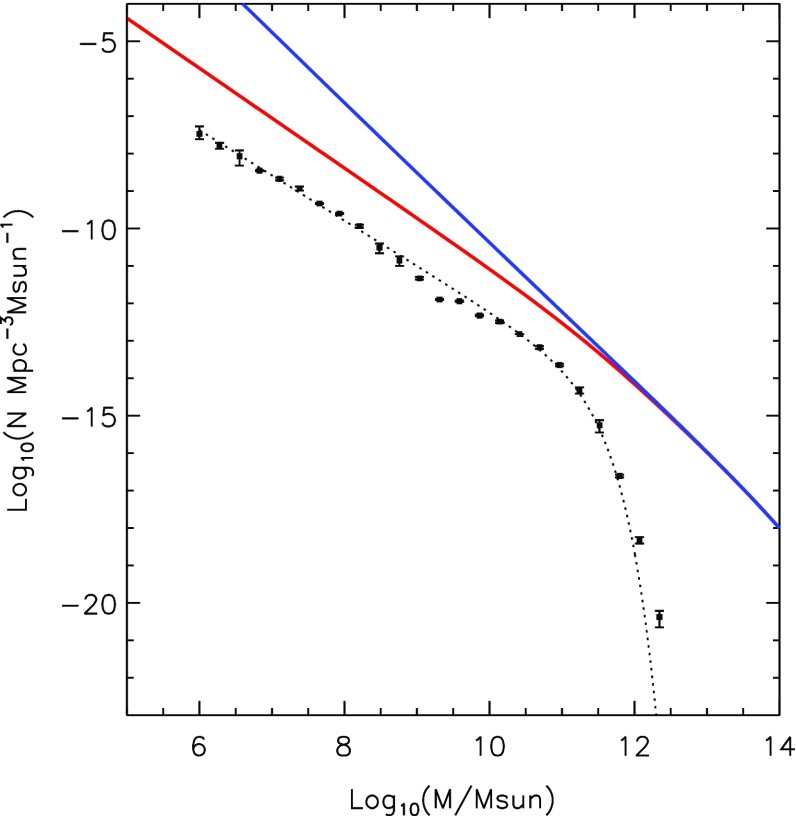
The baryonic mass function of galaxies (data points). The dotted line shows a Schechter function fit to the data. The blue line shows the predicted mass function of dark matter haloes, assuming that dark matter is cold. The red line shows the same assuming that dark matter is warm with a (thermal relic) mass of $m_{\mathrm {WDM}}=1\mathrm {\ keV}$

#### The halo mass function as a function of redshift

Attempts have already been made to probe the small scale power in the universe through galaxy counts. Figure 37 shows the best measurement of the ‘baryonic mass function’ of galaxies to date (Read and Trentham 2005). This is the number of galaxies with a given total mass in baryons normalized to a volume of 1 Mpc. To achieve this measurement, Read and Trentham (2005) sewed together results from a wide range of surveys reaching a baryonic mass of just $\sim \,10^6\,M_{\odot }$—some of the smallest galaxies observed to date.

The baryonic mass function already turns up an interesting result. Over-plotted in blue on Fig. 37 is the *dark matter* mass function expected assuming that dark matter is ‘cold’—i.e., that it has no preferred scale. Notice that this has a different shape. On large scales, there should be bound dark matter structures with masses as large as $10^{14}\,M_{\odot }$, yet the number of observed galaxies drops off exponentially above a baryonic mass of $\sim \,10^{12}\,M_{\odot }$. This discrepancy is well-understood. Such large dark matter haloes have been observed, but they no longer host a single galaxy; rather they are bound collections of galaxies—galaxy clusters (see e.g. Zwicky 1937). However, there is also a discrepancy at low masses that is not so well understood. There should be far more bound dark matter haloes than observed small galaxies. This is the well-known ‘missing satellite’ problem (Moore et al. 1999; Klypin et al. 1999).

The missing satellite problem could be telling us that dark matter is not cold. The red line on Fig. 37 shows the expected dark matter mass function for WDM with a (thermal relic) mass of $m_{\mathrm {WDM}}=1\mathrm {\ keV}$. Notice that this gives an excellent match to the observed slope of the baryonic mass function on small scales. However, there may be a less exotic solution. It is likely that star formation becomes inefficient in galaxies on small scales. A combination of supernovae feedback, reionization and ram-pressure stripping is sufficient to fully explain the observed distribution assuming pure CDM (Kravtsov et al. 2004; Read et al. 2006; Macciò et al. 2010). Such ‘baryon feedback’ solutions to the missing satellite problem are also supported by recent measurements of the orbits of the Milky Way’s dwarf galaxies (Lux et al. 2010).

*II.2.1.1. Weak and strong lensing measurements of the halo mass function*

To make further progress on WDM constraints from astrophysics, we must avoid the issue of baryonic physics by probing the halo mass function *directly*. The only tool for achieving this is gravitational lensing. In weak lensing this means stacking data for a very large number of galaxies to obtain an averaged mass function. In strong lensing, this means simply finding enough systems with ‘good data’. Good data ideally means multiple sources with wide redshift separation (Saha and Read 2009); combining independent data from dynamics with lensing may also prove a promising route (see e.g. Treu and Koopmans 2002).

Euclid will measure the halo mass function down to $\sim \, 10^{13}\,M_{\odot }$ using weak lensing. It will simultaneously find 1000s of strong lensing systems. However, in both cases, the lowest mass scale is limited by the lensing critical density. This limits us to probing down to a halo mass of $\sim \,10^{11}\,M_{\odot }$ which gives poor constraints on the nature of dark matter. However, if such measurements can be made as a *function of redshift*, the constraints improve dramatically. We discuss this in the next Section.

*II.2.1.2 The advantage of going to high redshift*

**Fig. 38:**
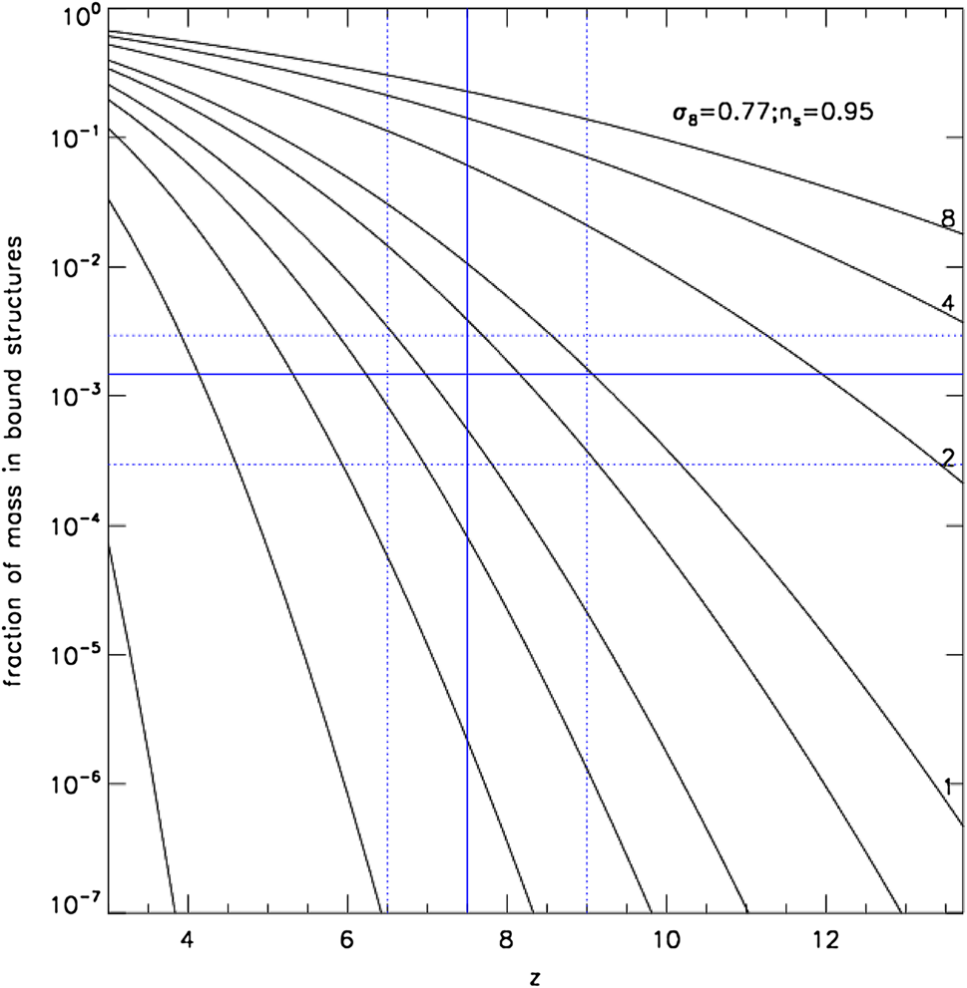
The fraction of mass in bound structures as a function of redshift, normalized to a halo of Milky Way’s mass at redshift $z=0$. Marked are different masses of thermal-relic WDM particles in keV (black solid lines). Notice that the differences between different WDM models increases towards higher redshift

Dark matter constraints from the halo mass function become much stronger if the halo mass function is measured as a function of redshift. This is because warm dark matter *delays* the growth of structure formation as well as suppressing small scale power. This is illustrated in Fig. 38, which shows the fraction of mass in bound structures as a function of redshift, normalized to a halo of Milky Way’s mass at redshift $z=0$. Marked are different thermal relic WDM particle masses in keV (black solid lines). Notice that the differences between WDM models increase significantly towards higher redshift at a given mass scale. Thus we can obtain strong constraints on the nature of dark matter by moving to higher *z*’s, rather than lower halo mass.

The utility of redshift information was illustrated recently by observations of the Lyman-$\alpha$ absorption spectra from Quasars (Viel et al. 2008; Seljak et al. 2006). Quasars act as cosmic ‘flashlights’ shining light from the very distant universe. Some of this light is absorbed by intervening neutral gas leading to absorption features in the Quasar spectra. Such features contain rich information about the matter distribution in the universe at high redshift. Thus, the Lyman-$\alpha$ forest measurements have been able to place a lower bound of $m_{\mathrm {WDM}}>4\mathrm {\ keV}$ probing scales of $\sim \,1\mathrm {\ Mpc}$. Key to the success of this measurement is that much of the neutral gas lies in-between galaxies in filaments. Thus, linear approximations for the growth of structures in WDM versus CDM remain acceptable, while assuming that the baryons are a good tracer of the underlying matter field is also a good approximation. However, improving on these early results means probing smaller scales where nonlinearities and baryon physics will creep in. For this reason, tighter bounds must come from techniques that either probe even higher redshifts, or even smaller scales. Lensing from Euclid is an excellent candidate since it will achieve both while measuring the halo mass function directly rather than through the visible baryons.

#### The dark matter density profile

**Fig. 39:**
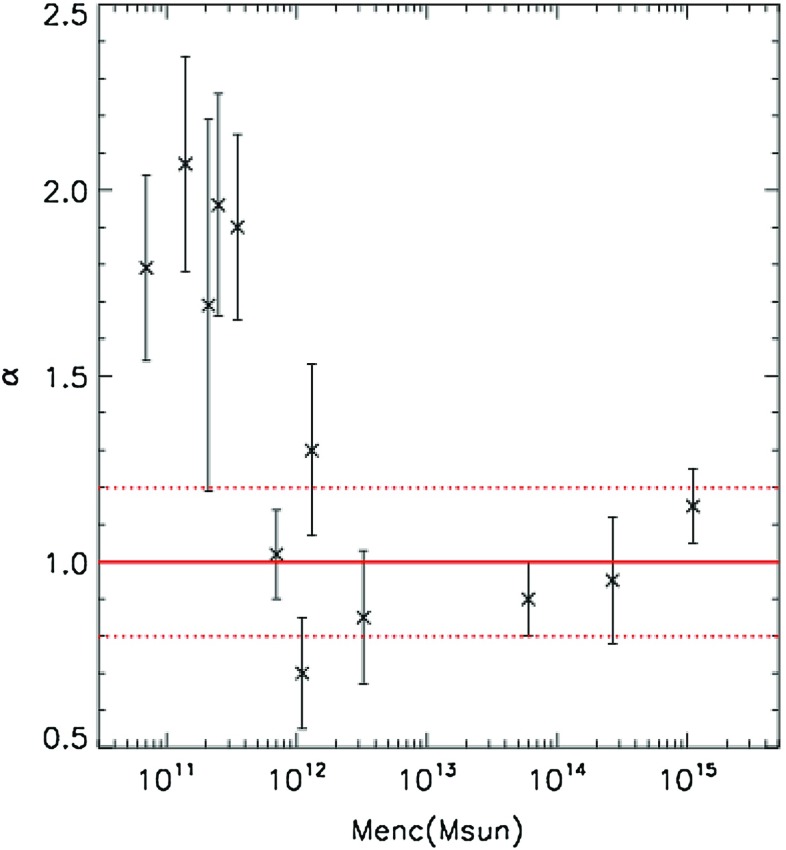
The central log-slope $\alpha$ of the density distribution $\rho \propto r^\alpha$ for 9 galaxies/groups and 3 lensing clusters as a function of the enclosed lensing mass. Marked in red is the prediction from structure formation simulations of the standard cosmological model, that assume non-relativistic CDM, and that do not include any baryonic matter

An alternative approach to constraining dark matter models is to measure the distribution of dark matter *within* galaxies. Figure 39 shows the central log-slope of the density distribution for 9 galaxies/groups and 3 lensing clusters as a function of the enclosed lensing mass (Saha et al. 2006; Read et al. 2007; Saha and Read 2009). Over the visible region of galaxies, the dark matter distribution tends towards a single power law: $\rho \propto r^\alpha$. Marked in red is the prediction from structure-formation simulations of the standard cosmological model, that assume non-relativistic CDM, and that do not include any baryonic matter. Notice that above an enclosed lensing mass of $\sim \, 10^{12}\,M_{\odot }$, the agreement between theory and observations is very good. This lends support to the idea that dark matter is cold and not strongly self-interacting. However, this result is based on only a handful of galaxy clusters with excellent data. Furthermore, lower mass galaxies and groups can, in principle, give tighter constraints. In these mass ranges, however ($M_{\mathrm {enc}}<10^{12}\,M_{\odot }$), the lensing mass is dominated by the visible stars. Determining the underlying dark matter distribution is then much more difficult. It is likely that the dark matter distribution is also altered from simple predictions by the dynamical interplay between the stars, gas and dark matter during galaxy formation (e.g., Debattista et al. 2008).

### Euclid dark matter studies: wide-field X-ray complementarity

The predominant extragalactic X-ray sources are AGNs and galaxy clusters. For dark matter studies the latter are the more interesting targets. X-rays from clusters are emitted as thermal bremsstrahlung by the hot intracluster medium (ICM) which contains most of the baryons in the cluster. The thermal pressure of the ICM supports it against gravitational collapse so that measuring the temperature through X-ray observations provides information about the mass of the cluster and its distribution. Hence, X-rays form a complementary probe of the dark matter in clusters to Euclid weak lensing measurements.

The ongoing X-ray missions XMM-Newton and Chandra have good enough angular resolution to measure the temperature and mass profiles in $\sim \,10$ radial bins for clusters at reasonable redshifts, although this requires long exposures. Many planned X-ray missions aim to improve the spectral coverage, spectral resolution, and/or collection area of the present mission, but they are nonetheless mostly suited for targeted observations of individual objects. Two notable exceptions are eROSITA^16^ (Cappelluti et al. 2011) and the Wide Field X-ray Telescope^17^ (WFXT Giacconi et al. 2009; Vikhlinin et al. 2009; Sartoris et al. 2010; Rosati et al. 2011; Borgani et al. 2011; Sartoris et al. 2012, proposed) which will both conduct full sky surveys and, in the case of WFXT, also smaller but deeper surveys of large fractions of the sky.

A sample of high-angular resolution X-ray cluster observations can be used to test the prediction from *N*-body simulations of structure formation that dark matter haloes are described by the NFW profile (Navarro et al. 1996) with a concentration parameter *c*. This describes the steepness of the profile, which is related to the mass of the halo (Neto et al. 2007). Weak or strong lensing measurements of the mass profile, such as those that will be provided from Euclid, can supplement the X-ray measurement and have different systematics. Euclid could provide wide field weak lensing data for such a purpose with very good point spread function (PSF) properties, but it is likely that the depth of the Euclid survey will make dedicated deep field observations a better choice for a lensing counterpart to the X-ray observations. However, if the WFXT mission becomes a reality, the sheer number of detected clusters with mass profiles would mean Euclid could play a much more important rôle.

X-ray observations of galaxy clusters can constrain cosmology by measuring the geometry of the universe through the baryon fraction $f_{\mathrm {gas}}$ (Allen et al. 2008) or by measuring the growth of structures by determining the high-mass tail of the mass function (Mantz et al. 2010). The latter method would make the most of the large number of clusters detected in full-sky surveys and there would be several benefits by combining an X-ray and a lensing survey. It is not immediately clear which type of survey would be able to better detect clusters at various redshifts and masses, and the combination of the two probes could improve understanding of the sample completeness. An X-ray survey alone cannot measure cluster masses with the required precision for cosmology. Instead, it requires a calibrated relation between the X-ray temperature and the cluster mass. Such a calibration, derived from a large sample of clusters, could be provided by Euclid. In any case, it is not clear yet whether the large size of a Euclid sample would be more beneficial than deeper observations of fewer clusters.

Finally, X-ray observations can also confirm the nature of possible ‘bullet-like’ merging clusters. In such systems the shock of the collision has displaced the ICM from the dark matter mass, which is identified through gravitational lensing. This offers the opportunity to study dark matter haloes with very few baryons and, e.g., search for signatures of decaying or annihilating dark matter.

### Dark matter mapping

Gravitational lensing offers a unique way to chart dark matter structures in the universe as it is sensitive to all forms of matter. Weak lensing has been used to map the dark matter in galaxy clusters (see for example Clowe et al. 2006b) with high resolution reconstructions recovered for the most massive strong lensing clusters (see for example Bradač et al. 2006). Several lensing studies have also mapped the projected surface mass density over degree scale-fields (Gavazzi and Soucail 2007; Schirmer et al. 2007; Kubo et al. 2009) to identify shear-selected groups and clusters. The minimum mass scale that can be identified is limited only by the intrinsic ellipticity noise in the lensing analysis and projection effects. Using a higher number density of galaxies in the shear measurement reduces this noise, and for this reason the Deep Field Euclid Survey will be truly unique for this area of research, permitting high resolution reconstructions of dark matter in the field (Massey et al. 2007; Heymans et al. 2008) and the study of lenses at higher redshift.

There are several non-parametric methods to reconstruct dark matter in 2D which can be broadly split into two categories: convergence techniques (Kaiser and Squires 1993) and potential techniques (Bartelmann et al. 1996). In the former one measures the projected surface mass density (or convergence) $\kappa$ directly by applying a convolution to the measured shear under the assumption that $\kappa \ll 1$. Potential techniques perform a $\chi ^2$ minimization and are better suited to the cluster regime and can also incorporate strong lensing information (Bradač et al. 2005). In the majority of methods, choices need to be made about smoothing scales to optimize signal-to-noise whilst preserving reconstruction resolution. Using a wavelet method circumvents this choice (Starck et al. 2006; Khiabanian and Dell’Antonio 2008) but makes the resulting significance of the reconstruction difficult to measure.

In Van Waerbeke et al. (2013) the techniques of weak lensing mass mapping were applied to a wide-field survey for the first time, using the CFHTLenS data set. These mass maps were used to generate higher order statistics beyond the two-point correlation function.

#### Charting the universe in 3D

The lensing distortion depends on the total projected surface mass density along the line of sight with a geometrical weighting that peaks between a given source and observer, while increasing with source distance. This redshift dependence can be used to recover the full 3D gravitational potential of the matter density as described in Hu and Keeton (2002), Bacon and Taylor (2003) and applied to the COMBO-17 survey in Taylor et al. (2004) and the COSMOS survey in Massey et al. (2007). This work has been extended in Simon et al. (2009) to reconstruct the full 3D mass density field and applied to the STAGES survey in Simon et al. (2012).

All 3D mass reconstruction methods require the use of a prior based on the expected mean growth of matter density fluctuations. Without the inclusion of such a prior, Hu and Keeton (2002) have shown that one is unable to reasonably constrain the radial matter distribution, even for densely sampled space-based quality lensing data. Therefore 3D maps cannot be directly used to infer cosmological parameters.

The driving motivation behind the development of 3D reconstruction techniques was to enable an unbiased 3D comparison of mass and light. Dark haloes for example would only be detected in this manner. However the detailed analysis of noise and the radial PSF in the 3D lensing reconstructions presented for the first time in Simon et al. (2012) show how inherently noisy the process is. Given the limitations of the method to resolve only the most massive structures in 3D the future direction of the application of this method for the Euclid Wide survey should be to reconstruct large scale structures in the 3D density field. Using more heavily spatially smoothed data we can expect higher quality 3D resolution reconstructions as on degree scales the significance of modes in a 3D mass density reconstruction are increased (Simon et al. 2009). Adding additional information from flexion may also improve mass reconstruction, although using flexion information alone is much less sensitive than shear (Pires and Amara 2010).

#### Mapping large-scale structure filaments

Structure formation theory robustly predicts that matter in the Universe is concentrated in sheets and filaments and that galaxy clusters live at the intersection of these filaments. The most comprehensive analytical framework for describing the emergence of these structure from anisotropic gravitational collapse is the work of Bond et al. (1996), which coined the term “cosmic web” for them. It combines the linear evolution of density fluctuations in the Zeldovich approximation (Zel’dovich 1970) with the statistics of peaks in the primordial density field (Bardeen et al. 1986) using the the peak-patch formalism (Bond and Myers 1996a, b, c).

Numerically, filaments have been seen since the early days of *N*-body simulations (e.g., Klypin and Shandarin 1983). Increasing mass and spatial resolution of these simulations have refined our understanding of them and a detailed inventory of the mass distribution over the different kinds of large-scale structures (galaxy clusters, filaments, sheets, voids) indicates that a plurality of all mass in the Universe and thus probably of all galaxies is in filaments (Aragón-Calvo et al. 2010).

Observationally, filaments have been traced by galaxy redshift surveys from early indications (Joeveer et al. 1978) of their existence to conclusive evidence in the CfA redshift survey (Geller and Huchra 1989) to modern day redshift surveys like 2dF, SDSS, BOSS and VIPERS (Colless et al. 2001; Ahn et al. 2012; Dawson et al. 2013; Guzzo et al. 2014). In X-rays the tenuous warm-hot intergalactic medium expected to reside in filaments (Davé et al. 2001) has been seen in emission (Werner et al. 2008) and absorption (Buote et al. 2009; Fang et al. 2010). Observing the underlying dark matter skeleton has been much more challenging and early weak-lensing candidates for direct detections (Kaiser et al. 1998; Gray et al. 2002) could not be confirmed by higher quality follow-up observations (Gavazzi et al. 2004; Heymans et al. 2008).

The most significant weak-lensing detection of a large-scale structure filament yet was presented by Dietrich et al. (2012), who found a mass bridge connecting the galaxy clusters Abell 222 and Abell 223 at $4\sigma$ in a mass reconstruction. This dark matter filament is spatially coincident with an overdensity of galaxies (Dietrich et al. 2005) and extended soft X-ray emission (Werner et al. 2008). This study, like the others mentioned before, makes use of the fact that filaments have a higher density closer to galaxy clusters and are expected to be particular massive between close pairs of galaxy clusters (Bond et al. 1996). Jauzac et al. (2012), reported another weak-lensing filament candidate at $3\sigma$ significance coming out of a galaxy cluster but not obviously connecting to another overdensity of galaxies or dark matter. The works of Dietrich et al. (2012) and Jauzac et al. (2012) also provide the first direct mass measurements of filaments. These are in agreement with the prediction that massive filaments can be as heavy as small galaxy clusters (Aragón-Calvo et al. 2010).

The relative dearth of weak lensing filament observations compared to galaxy cluster measurements is of course due to their much lower density contrast. Numerical simulations of weak-lensing observations accurately predict that filaments will generally be below their detection threshold (Dietrich et al. 2005; Mead et al. 2010). A statistical analysis of large set of ray-tracing simulations indicates that even with a survey slightly deeper than the Euclid wide-survey, the vast majority of filaments will not be individually detectable (Higuchi et al. 2014). Maturi and Merten (2013) propose a matched filter tuned to the shape of filaments to overcome the obstacles in filament detections in weak lensing data.

An alternative to lensing by individual filaments is to average (or “stack”) the lensing signal of many filaments. These filaments could either be identified in the Euclid spectroscopic survey or one could use the high probability that neighbouring massive dark matter halos are often connected by filaments. Zhang et al. (2013) pioneered this technique of blindly stacking the area between galaxy cluster pairs to boost the overdensity of filament galaxies with respect to the field. Their selection of cluster pairs was based on statistical studies of the abundance and properties of filaments between cluster pairs (Pimbblet et al. 2004; Colberg et al. 2005). This stacking approach was extended to weak lensing by Clampitt et al. (2016). They developed a method to measure the lensing signal of extended structures while at the same time nulling the contribution of the halo pairs at the endpoints of filaments. Stacking the lensing signal in the regions between luminous red galaxies in SDSS, Clampitt et al. (2016) were able to put first constraints on the density profiles of filaments.

### Constraints on dark matter interaction cross sections

We now move towards discussing the particulate aspects of dark matter, starting with a discussion on the scattering cross-sections of dark matter. At present, many physical properties of the dark matter particle remain highly uncertain. Prospects for studying the scattering of dark matter with each of the three major constituents of the universe—itself, baryons, and dark energy—are outlined below.

#### Dark matter–dark matter interactions

Self-interacting dark matter (SIDM) was first postulated by Spergel and Steinhardt (2000), in an attempt to explain the apparent paucity of low-mass haloes within the Local Group. The required cross-section $\sigma /m\sim 1\,\hbox {cm}^2$/g was initially shown to be infeasible (Meneghetti et al. 2001; Gnedin and Ostriker 2001), but recent high-resolution simulations have revised the expected impact of self-interaction, which now remains consistent with observations of cluster halo shapes and profiles. Indeed, self-interaction within a hidden dark sector is a generic consequence of some extensions to the Standard Model. For example, atomic, glueballino, and mirror dark matter models predict a cross-section $\sigma /m\approx 0.6\,\hbox {cm}^2/\hbox {g}=1$ barn/GeV (similar to nuclear cross-sections in the Standard Model). Note that couplings within the dark sector can be many orders of magnitude larger than those between dark matter and Standard Model particles, which is of order picobarns. Interactions entirely within the dark sector are unprobed by direct detection or collider experiments, but leads to several phyical effects that can potentially be observed by Euclid.

Clusters of galaxies present an interesting environment in which the dark matter density is sufficiently high for collisions to play a significant role. If dark matter particles possess even a small cross-section for elastic scattering, small-scale structure can be erased, and cuspy cores can be smoothed. In particular, collisions between galaxy clusters act as astronomical-scale particle colliders. Since dark matter and baryonic matter are subject to different forces, they follow different trajectories out of the collision. If dark matter’s particle interactions are rare but exchange a lot of momentum (often corresponding to short-ranged forces), dark matter will tend to be scattered away and lost. If the interactions are rare but exchange little momentum (often corresponding to long-ranged forces),the dark matter will be decelerated by an additional drag force and become spatially offset (Kahlhoefer et al. 2014).

How do these cosmological constraints relate to the values anticipated by particle physics? WIMPs are expected to fall in the range of 10 GeV to a few TeV. The aforementioned values would then correspond to around $\sigma _p\lesssim 10^{-24}\mathrm {\ cm}^2$, at least twenty order of magnitudes greater than what one might expect to achieve from neutral current interactions. Therefore in a cosmological context WIMPs are essentially collisionless, as are axions, since they exhibit an even smaller cross section. Any cosmological detection of SIDM would thus point towards the more exotic candidates postulated by particle physicists, particularly those which are not point particles but instead comprise of extended objects such as Q-balls. A measurement of the scattering cross-section would also place an upper bound on the mass of the dark matter particle, since unitarity of the scattering matrix forbids extremely large cross sections (Hui 2001), i.e.,

II.5.1 $$\begin{aligned} \sigma _\mathrm {tot}\le 1.76\times 10^{-17}\,\mathrm {cm^2}\left( \frac{\mathrm {GeV}}{m_\chi }\right) ^2\left( \frac{10\,\mathrm {km\ s^{-1}}}{v_\mathrm {rel}}\right) ^2. \end{aligned}$$

*II.5.1.1 Dark matter evaporation*

As highlighted by Gnedin and Ostriker (2001), cross-sections large enough to alleviate the structure formation issues would also allow significant heat transfer from particles within a large halo to the cooler sub-haloes. This effect is most prominent close to the centers of clusters. As the sub-halo evaporates, the galaxy residing within the halo would be disrupted. Limiting this rate of evaporation to exceed the Hubble time allows an upper bound to be placed on the scattering cross-section of approximately $\sigma _p/m_p\lesssim 0.3\mathrm {\ cm^{2}\ g^{-1}}$ (neglecting any velocity dependence). Note the dependence on particle mass—a more massive CDM particle would be associated with a lower number density, thereby reducing the frequency of collisions.

*II.5.1.2 Dark matter deceleration*

Particulate dark matter and baryonic matter may be temporarily separated during collisions between galaxy clusters, such as 1E 0657-56 (Clowe et al. 2006a; Bradač et al. 2006) and MACS J0025.4-1222 (Bradač et al. 2008). These ‘bullet clusters’ have provided astrophysical constraints on the interaction cross-section of hypothesized dark matter particles (Randall et al. 2008), and may ultimately prove the most useful laboratory in which to test for any velocity dependence of the cross-section. Unfortunately, high-speed collisions between two massive progenitors are rare (Shan et al. 2010a, b), and constraints from individual systems are limited by uncertainties in their collision velocity, impact parameter and angle with respect to the plane of the sky.

However, all galaxy clusters grow through almost continual minor merger accretion. In Massey et al. (2011) and Harvey et al. (2014), a statistical ‘bulleticity’ method has been proposed to exploit every individual infalling substructure in every cluster. For each piece of infalling substructure, a local vector from the dark matter peak (identified using weak lensing analysis) and the baryonic mass peak (from X-rays). An average bulleticity signal of zero would imply an equal cross sections for the dark matter and baryonic matter. By measuring any observed, finite amplitude of bulleticity, one can empirically measure the ratio between the dark matter self-interaction and baryonic self-interaction cross sections. Since we know the baryonic cross-section relatively well, we can infer the dark matter-dark matter cross-section.

**Fig. 40:**
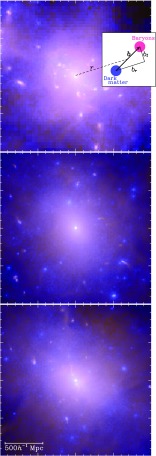
Full hydrodynamical simulations of massive clusters at redshift $z=0.6$. Total projected mass is shown in blue, while X-ray emission from baryonic gas is in red. The preferential trailing of gas due to pressure from the ICM, and its consequent separation from the non interacting dark matter, is apparent in much of the infalling substructure

In Fig. 40, a result from hydrodynamical simulations of dark and baryonic matter within clusters in shown. Massey et al. (2011) and Harvey et al. (2014) have used these simulations to show that the measurement of a net bulleticity consistent with the cold dark matter used in the simulations will be possible with Euclid.

Finally, a Fisher matrix calculation has shown that, under the assumption that systematic effects can be controlled, Euclid could use such a technique to constrain the relative particulate cross-sections to $6\times 10^{-27}\mathrm {\ cm^{2}\ GeV^{-1}}$.

The dark matter-dark matter interaction probed by Euclid using this technique will be complementary to the interactions constrained by direct detection and accelerator experiments where the primary constraints will be on the dark matter-baryon interaction.

*II.5.1.3 Dark matter halo shapes*

Self-interacting dark matter circularises the centres of dark matter halos, removing triaxiality (Feng et al. 2010; Peter et al. 2013), and smooths out cuspy cores. These profiles can be measeured directly using strong gravitational lensing (e.g., Sand et al. 2008; Newman et al. 2013).

Meneghetti et al. (2001) have performed ray-tracing through *N*-body simulations, and have discovered that the ability for galaxy clusters to generate giant arcs from strong lensing is compromized if the dark matter is subject to just a few collisions per particle. This constraint translates to an upper bound $\sigma _p/m_p\lesssim 0.1\mathrm {\ cm^{2}\ g^{-1}}$. Furthermore, more recent analyses of SIDM models (Markevitch et al. 2004; Randall et al. 2008) utilize data from the Bullet Cluster to provide another independent limit on the scattering cross section, though the upper bound remains unchanged. Massey et al. (2011) have proposed that the tendency for baryonic and dark matter to become separated within dynamical systems, as seen in the Bullet Cluster, could be studied in greater detail if the analysis were to be extended over the full sky in Euclid.

#### Dark matter–baryonic interactions

Currently, a number of efforts are underway to directly detect WIMPs via the recoil of atomic nuclei. The underground experiments such as CDMS, CRESST, XENON, EDELWEISS and ZEPLIN have pushed observational limits for the spin-independent WIMP-nucleon cross-section down to the $\sigma \lesssim 10^{-43}\mathrm {cm}^2$ régime.^18^ A collection of the latest constraints can be found at http://dmtools.brown.edu.

Another opportunity to unearth the dark matter particle lies in accelerators such as the LHC. By 2018 it is possible these experiments will have yielded mass estimates for dark matter candidates, provided its mass is lighter than a few hundred GeV. However, the discovery of more detailed properties of the particle, which are essential to confirm the link to cosmological dark matter, would have to wait until the International Linear Collider is constructed.

#### Dark matter–dark energy interactions

Interactions in the dark sector have provided a popular topic for exploration, with a view to building models which alleviate the coincidence and fine-tuning issues associated with dark energy (see Sect. I.5.3). The great uncertainty surrounding the physical nature of dark energy leaves plenty of scope for non-gravitational physics to play a rôle. These models are discussed at length in other sections of this reviews (I.5 and II.10). Here, we only mention that Simpson (2010) have explored the phenomenology associated with dark matter scattering elastically with dark energy. The growth rate of large-scale structures is artificially slowed, allowing a modest constraint of

II.5.2 $$\begin{aligned} \sigma _p/m_p\lesssim \frac{10}{1+w}\mathrm {\ cm^{2}\ g^{-1}}. \end{aligned}$$

It is clear that such dark sector interactions do not arise in the simplest models of dark matter and dark energy. However a rigorous refutation of GR will require not only a robust measure of the growth of cosmic structures, but confirmation that the anomalous dynamics are not simply due to physics within the dark sector.

### Constraints on warm dark matter

*N*-body simulations of large-scale structures that assume a $\varLambda$CDM cosmology appear to over-predict the power on small scales when compared to observations (Primack 2009): ‘the missing-satellite problem’ (Kauffmann et al. 1993; Klypin et al. 1999; Strigari et al. 2007; Bullock 2010), the ‘cusp-core problem’ (Li and Chen 2009; Simon et al. 2005; Zavala et al. 2009) and sizes of mini-voids (Tikhonov et al. 2009). These problems may be more or less solved by several different phenomena (e.g. Diemand and Moore 2011), however one which could explain all of the above is warm dark matter (WDM) (Bode et al. 2001; Colin et al. 2000; Boyanovsky et al. 2008). If the dark matter particle is very light, it can cause a suppression of the growth of structures on small scales via free-streaming of the dark matter particles whilst relativistic in the early universe.

#### Warm dark matter particle candidates

Numerous WDM particle models can be constructed, but there are two that occur most commonly in literature, because they are most plausible from particle physics theory as well as from cosmological observations:

- Sterile neutrinos may be constructed to extend the standard model of particle physics. The standard model active (left-handed) neutrinos can then receive the observed small masses through, e.g., a see-saw mechanism. This implies that right-handed sterile neutrinos must be rather heavy, but the lightest of them naturally has a mass in the keV region, which makes it a suitable WDM candidate. The simplest model of sterile neutrinos as WDM candidate assumes that these particles were produced at the same time as active neutrinos, but they never thermalized and were thus produced with a much reduced abundance due to their weak coupling (see Biermann and Munyaneza 2008, and references therein).
- The gravitino appears as the supersymmetric partner of the graviton in supergravity models. If it has a mass in the keV range, it will be a suitable WDM candidate. It belongs to a more general class of *thermalized* WDM candidates. It is assumed that this class of particles achieved a full thermal equilibrium, but at an earlier stage, when the number of degrees of freedom was much higher and hence their relative temperature with respect to the CMB is much reduced. Note that in order for the gravitino to be a good dark matter particle in general, it must be very stable, which in most models corresponds to it being the LSP (e.g. Borgani and Masiero 1997; Cembranos et al. 2005).

Other possible WDM candidates exist, for example a non-thermal neutralino (Hisano et al. 2001) or a non-thermal gravitino (Baltz and Murayama 2003) etc.

#### Dark matter free-streaming

The modification of the shape of the linear-theory power spectrum of CDM due to WDM can be calculated by multiplication by a transfer function (Bode et al. 2001)

II.6.1 $$\begin{aligned} T(k)\equiv \sqrt{\frac{P_{\mathrm {WDM}}(k)}{P_{\mathrm {CDM}}(k)}}=\left[ 1+(\alpha k)^{2\mu }\right] ^{-5/\mu }, \end{aligned}$$

with suitable parameter $\mu =1.12$ (Viel et al. 2005) and with the scale break parameter, $\alpha$, in the case of thermal relic DM

II.6.2 $$\begin{aligned} \alpha =0.049\left( \frac{m_{\mathrm {WDM}}}{\mathrm {keV}}\right) ^{-1.11}\left( \frac{\varOmega _{\mathrm {WDM}}}{0.25} \right) ^{0.11}\left( \frac{h}{0.7}\right) ^{1.22}\,h^{-1}\,\mathrm {Mpc}. \end{aligned}$$

This is a fit to the solution of the full Boltzman equations.

There is a one-to-one relation between the mass of the thermalized WDM particle $m_\mathrm {WDM}$ (e.g., gravitino), and the mass of the simplest sterile neutrino $m_\mathrm {\nu s}$, such that the two models have an identical impact on cosmology (Viel et al. 2005)

II.6.3 $$\begin{aligned} m_\mathrm {\nu s}=4.43\left( \frac{m_{\mathrm {WDM}}}{\mathrm {keV}}\right) ^{4/3}\left( \frac{\omega _{\mathrm {WDM}}}{0.1225}\right) ^{-1/3}\mathrm {\ keV}, \end{aligned}$$

where $\omega =\varOmega h^2$. The difference comes from the fact that in the gravitino case the particle is fully thermalized, the number of effective degrees of freedom being determined by mass and energy density of dark matter, while in the simplest sterile neutrino case the number of degrees of freedom is fixed, while abundance is determined by mass and energy density of dark matter.

#### Current constraints on the WDM particle from large-scale structure

Measurements in the particle-physics energy domain can only reach masses uninteresting in the WDM context, since direct detectors look mainly for a WIMP, whose mass should be in the GeV–TeV range. However, as described above, cosmological observations are able to place constraints on light dark matter particles. Observation of the flux power spectrum of the Lyman-$\alpha$ forest, which can indirectly measure the fluctuations in the dark matter density on scales between $\sim \,100\mathrm {\ kpc}$ and $\sim \,10\mathrm {\ Mpc}$ gives the limits of $m_{\mathrm {WDM}}>4\mathrm {\ keV}$ or equivalently $m_{\mathrm {\nu s}}>28\mathrm {\ keV}$ at 95% confidence level (Viel et al. 2008, 2005; Seljak et al. 2006). For the simplest sterile neutrino model, these lower limits are at odds with the upper limits derived from X-ray observations, which come from the lack of observed diffuse X-ray background from sterile neutrino annihilation and set the limit $m_{\mathrm {\nu s}}<1.8\mathrm {\ keV}$ at the 95% confidence limit (Boyarsky et al. 2006). However, these results do not rule the simplest sterile neutrino models out. There exist theoretical means of evading small-scale power constraints (see e.g. Boyarsky et al. 2009, and references therein). The weak lensing power spectrum from Euclid will be able to constrain the dark matter particle mass to about $m_{\mathrm {WDM}}>2\mathrm {\ keV}$ (Markovič et al. 2011).

#### Nonlinear structure in WDM

In order to extrapolate the matter power spectrum to later times one must take into account the nonlinear evolution of the matter density field.

Several fitting functions have been found to calculate the nonlinear power on the small scales of the present-day matter power spectrum in the scenario where all dark matter is warm. The most basic approach is simply to modify the linear matter power spectrum from Eq. II.6.1, which is based on the output of Bolzmann codes like camb or class (Lewis et al. 2000b; Blas et al. 2011). One can then either (i) run simulations (Boehm et al. 2005; Boyanovsky et al. 2008; Zavala et al. 2009; Wang and White 2007; Colombi et al. 2009; Viel et al. 2012; Schneider et al. 2012; Benson et al. 2013; Angulo et al. 2013; Semenov et al. 2013), (ii) use the halo model (Smith and Markovic 2011; Schneider et al. 2012; Dunstan et al. 2011) or (iii) a fit analogous to Eq. II.6.1, where the $\varLambda$CDM nonlinear power spectrum is modified by a transfer function (Viel et al. 2012) to calculate the present-day power on the small scales:

II.6.4 $$\begin{aligned} T_{\mathrm {nl}}(k) \equiv \sqrt{\frac{P^\mathrm {nl}_{\mathrm {WDM}}(k)}{P^\mathrm {nl}_{\mathrm {CDM}}(k)}} = \left[ 1+(\alpha \,k)^{\mu l}\right] ^{-s/(2\mu )}, \end{aligned}$$

where

II.6.5 $$\begin{aligned} \alpha (m_{\mathrm {WDM}},z) = 0.0476 \left( \frac{\mathrm {keV}}{m_{\mathrm {WDM}}}\right) ^{1.85}\left( \frac{1+z}{2}\right) ^{1.3} , \end{aligned}$$

and $\mu =3$, $l=0.6$, $s=0.4$ are the fitting parameters.

Such fits can be used to calculate further constraints on WDM from the weak lensing power spectrum or galaxy clustering (Markovič et al. 2011; Markovič and Viel 2014)

It should be noted that in order to use the present day clustering of structure as a probe for WDM it is crucial to take into account baryonic physics as well as neutrino effect, which are described in the following section.

### Neutrino properties

The first significant evidence for a finite neutrino mass (Fukuda et al. 1998) indicated the incompleteness of the standard model of particle physics. Subsequent experiments have further strengthened this evidence and improved the determination of the neutrino mass splitting required to explain observations of neutrino oscillations.

As a summary of the last decade of neutrino experiments, two hierarchical neutrino mass splittings and three mixing angles have been measured. Furthermore, the standard model has three neutrinos: the motivation for considering deviations from the standard model in the form of extra sterile neutrinos has disappeared (Melchiorri et al. 2009; Aguilar-Arevalo et al. 2007). Of course, deviations from the standard effective numbers of neutrino species could still indicate exotic physics which we will discuss below (Sect. II.7.4).

**Fig. 41:**
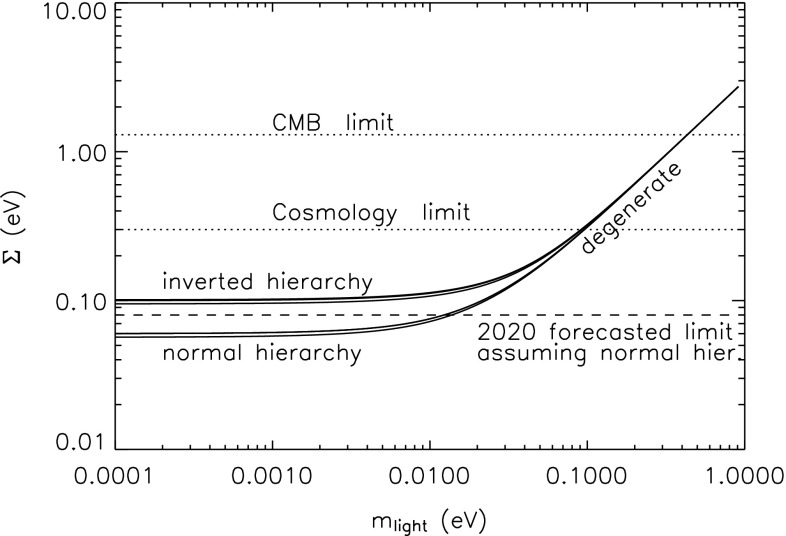
Constraints from neutrino oscillations and from cosmology in the *m*–$\varSigma$ plane. Image reproduced by permission from Jiménez et al. (2010); copyright by IOP and SISSA

New and future neutrino experiments aim to determine the remaining parameters of the neutrino mass matrix and the nature of the neutrino mass. Within three families of neutrinos, and given all neutrino oscillation data, there are three possible mass spectra: (a) degenerate, with mass splitting smaller than the neutrino masses, and two non-degenerate cases, (b) normal hierarchy (NH), with the larger mass splitting between the two more massive neutrinos and (c) inverted hierarchy (IH), with the smaller spitting between the two higher mass neutrinos. Figure 41 (Jiménez et al. 2010) illustrates the currently allowed regions in the plane of total neutrino mass, $\varSigma$, versus mass of the lightest neutrino, *m*. Note that a determination of $\varSigma <0.1\mathrm {\ eV}$ would indicate normal hierarchy and that there is an expected minimum mass $\varSigma >0.054\mathrm {\ eV}$. The cosmological constraint is from Reid et al. (2010).

Cosmological constraints on neutrino properties are highly complementary to particle physics experiments for several reasons:

- **Relic neutrinos** produced in the early universe are hardly detectable by weak interactions, making it impossible with foreseeable technology to detect them directly. But new cosmological probes such as Euclid offer the opportunity to detect (albeit indirectly) relic neutrinos, through the effect of their mass on the growth of cosmological perturbations.
- **Cosmology remains a key avenue to determine the absolute neutrino mass scale** Particle physics experiments will be able to place lower limits on the *effective* neutrino mass, which depends on the hierarchy, with no rigorous limit achievable in the case of normal hierarchy (Murayama and Peña-Garay 2004). Contrarily, neutrino free streaming suppresses the small-scale clustering of large-scale cosmological structures by an amount that depends on neutrino mass.
- **“What is the hierarchy (normal, inverted or degenerate)?”** Neutrino oscillation data are unable to resolve whether the mass spectrum consists in two light states with mass *m* and a heavy one with mass *M*—normal hierarchy—or two heavy states with mass *M* and a light one with mass *m*—inverted hierarchy—in a model-independent way. Cosmological observations, such as the data provided by Euclid, can determine the hierarchy, complementarily to data from particle physics experiments.
- **“Are neutrinos their own anti-particle?”** If the answer is yes, then neutrinos are Majorana fermions; if not, they are Dirac. If neutrinos and anti-neutrinos are identical, there could have been a process in the early universe that affected the balance between particles and anti-particles, leading to the matter anti-matter asymmetry we need to exist (Fukugita and Yanagida 1986). This question can, in principle, be resolved if neutrino-less double-$\beta$ decay is observed (see Murayama and Peña-Garay 2004, and references therein). However, if such experiments (ongoing and planned, e.g., Cremonesi 2010) lead to a negative result, the implications for the nature of neutrinos depend on the hierarchy. As shown in Jiménez et al. (2010), in this case cosmology can offer complementary information by helping determine the hierarchy.

#### Evidence of relic neutrinos

The hot big bang model predicts a background of relic neutrinos in the universe with an average number density of $\sim \,100\,N_{\nu }\mathrm {\ cm}^{-3}$, where $N_{\nu }$ is the number of neutrino species. These neutrinos decouple from the CMB at redshift $z\sim 10^{10}$ when the temperature was $T\sim o(\mathrm {MeV})$, but remain relativistic down to much lower redshifts depending on their mass. A detection of such a neutrino background would be an important confirmation of our understanding of the physics of the early universe.

Massive neutrinos affect cosmological observations in different ways. Primary CMB data alone can constrain the total neutrino mass $\varSigma$, if it is above $\sim \,1\mathrm {\ eV}$ (Komatsu et al. 2011, finds $\varSigma <1.3\mathrm {\ eV}$ at 95% confidence) because these neutrinos become non-relativistic before recombination leaving an imprint in the CMB. Neutrinos with masses $\varSigma <1\mathrm {\ eV}$ become non-relativistic after recombination altering matter-radiation equality for fixed $\varOmega _mh^2$; this effect is degenerate with other cosmological parameters from primary CMB data alone. After neutrinos become non-relativistic, their free streaming damps the small-scale power and modifies the shape of the matter power spectrum below the free-streaming length. The free-streaming length of each neutrino family depends on its mass.

Current cosmological observations do not detect any small-scale power suppression and break many of the degeneracies of the primary CMB, yielding constraints of $\varSigma <0.3\mathrm {\ eV}$ (Reid et al. 2010) if we assume the neutrino mass to be a constant. A detection of such an effect, however, would provide a detection, although indirect, of the cosmic neutrino background. As shown in the next section, the fact that oscillations predict a minimum total mass $\varSigma \sim 0.054\mathrm {\ eV}$ implies that Euclid has the statistical power to detect the cosmic neutrino background. We finally remark that the neutrino mass may also very well vary in time (Wetterich 2007); this might be tested by comparing (and not combining) measurements from CMB at decoupling with low-*z* measurements. An inconsistency would point out a direct measurement of a time varying neutrino mass (Wetterich and Pettorino 2009).

#### Neutrino mass

Particle physics experiments are sensitive to neutrino flavours making a determination of the neutrino absolute-mass scales very model dependent. On the other hand, cosmology is not sensitive to neutrino flavour, but is sensitive to the total neutrino mass.

The small-scale power-suppression caused by neutrinos leaves imprints on CMB lensing and prior to the experiment forecasts indicated that Planck should be able to constrain the sum of neutrino masses $\varSigma$, with a $1\sigma$ error of 0.13 eV (Kaplinghat et al. 2003; Lesgourgues et al. 2006; de Putter et al. 2009). In Planck Collaboration (2014a) reported constraints on the $N_{\mathrm{eff}}=3.30{+/-}0.27$ for the effective number of relativistic degrees of freedom, and an upper limit of 0.23 eV for the summed neutrino mass. However the Planck cosmological constraints also reported a relatively low value of the Hubble parameter with respect to previous measurements, that resulted in several papers, for example (Wyman et al. 2014), that investigated the possibility that this tension could possibly be resolved by introducing an eV-scale (possibly sterile) neutrino. Combining the Planck results with large scale structure measurements or weak lensing measurements has resulted in reported claims of even stronger constraints on the sum of neutrino masses, for example (Riemer-Sørensen et al. 2014) found an upper limit on the sum of neutrino masses of $<\, 0.18$ eV (95% confidence) by combining with WiggleZ data (Battye and Moss 2014) and (Hamann and Hasenkamp 2013) combined Planck data with weak lensing data from CFHTLenS and found higher values for the sum of neutrino masses, as a result of tension in the measured and inferred values of $\sigma _8$ between lensing and the CMB where the lensing prefers a lower value, however (Kitching et al. 2014) find that such a lower value of $\sigma _8$ is consistent with Baryon feedback models impacting the small-scale distribution of dark matter.

Euclid’s measurement of the galaxy power spectrum, combined with Planck (primary CMB only) priors should yield an error on $\varSigma$ of 0.04 eV (for details see Carbone et al. 2011b) which is in qualitative agreement with previous work (e.g., Saito et al. 2009), assuming a minimal value for $\varSigma$ and constant neutrino mass. Euclid’s weak lensing should also yield an error on $\varSigma$ of 0.05 eV (Kitching et al. 2008a). While these two determinations are not fully independent (the cosmic variance part of the error is in common given that the lensing survey and the galaxy survey cover the same volume of the universe) the size of the error-bars implies more than $1\sigma$ detection of even the minimum $\varSigma$ allowed by oscillations. Moreover, the two independent techniques will offer cross-checks and robustness to systematics. The error on $\varSigma$ depends on the fiducial model assumed, decreasing for fiducial models with larger $\varSigma$. Euclid will enable us not only to detect the effect of massive neutrinos on clustering but also to determine the absolute neutrino mass scale. However, recent numerical investigations found severe observational degeneracies between the cosmological effects of massive neutrinos and of some modified gravity models (Baldi et al. 2014). This may indicate an intrinsic theoretical limit to the effective power of astronomical data in discriminating between alternative cosmological scenarios, and in constraining the neutrino mass as well. Further investigations with higher resolution simulations are needed to clarify this issue and to search for possible ways to break these cosmic degeneracies (see also La Vacca et al. 2009; Kristiansen et al. 2010; Marulli et al. 2012).

#### Hierarchy and the nature of neutrinos

Since cosmology is insensitive to flavour, one might expect that cosmology may not help in determining the neutrino mass hierarchy. However, for $\varSigma <0.1\mathrm {\ eV}$, only normal hierarchy is allowed, thus a mass determination can help disentangle the hierarchy. There is however another effect: neutrinos of different masses become non-relativistic at slightly different epochs; the free streaming length is sightly different for the different species and thus the detailed shape of the small scale power suppression depends on the individual neutrino masses and not just on their sum. As discussed in Jiménez et al. (2010), in cosmology one can safely neglect the impact of the solar mass splitting. Thus, two masses characterize the neutrino mass spectrum: the lightest *m*, and the heaviest *M*. The mass splitting can be parameterized by $\varDelta =(M-m)/\varSigma$ for normal hierarchy and $\varDelta =(m-M)/\varSigma$ for inverted hierarchy. The absolute value of $\varDelta$ determines the mass splitting, whilst the sign of $\varDelta$ gives the hierarchy. Cosmological data are very sensitive to $|\varDelta |$; the direction of the splitting—i.e., the sign of $\varDelta$—introduces a sub-dominant correction to the main effect. Nonetheless, Jiménez et al. (2010) show that weak gravitational lensing from Euclid data will be able to determine the hierarchy (i.e., the mass splitting and its sign) if far enough away from the degenerate hierarchy (i.e., if $\varSigma <0.13$).

**Fig. 42:**
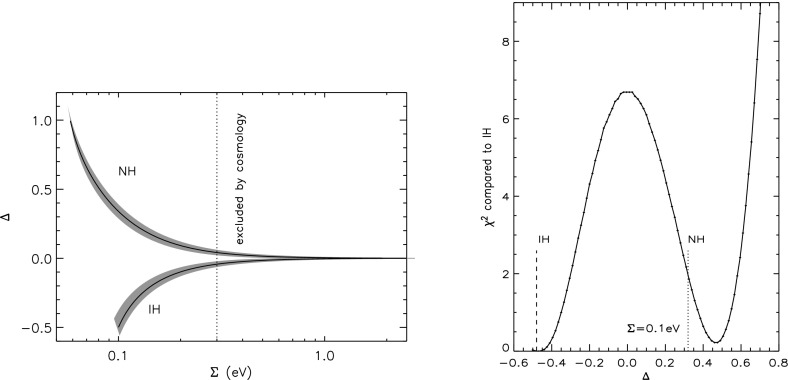
Left: region in the $\varDelta$–$\varSigma$ parameter space allowed by oscillations data. Right: Weak lensing forecasts. The dashed and dotted vertical lines correspond to the central value for $\varDelta$ given by oscillations data. In this case Euclid could discriminate NI from IH with a $\varDelta \chi ^2=2$. Image reproduced by permission from Jiménez et al. (2010); copyright by IOP and SISSA

A detection of neutrino-less double-$\beta$ decay from the next generation experiments would indicate that neutrinos are Majorana particles. A null result of such double-$\beta$ decay experiments would lead to a result pointing to the Dirac nature of the neutrino only for degenerate or inverted mass spectrum. Even in this case, however, there are ways to suppress the double-$\beta$ decay signal, without the neutrinos being Dirac particles. For instance, the pseudo-Dirac scenario, which arises from the same Lagrangian that describes the see-saw mechanism (see, e.g., Rodejohann 2012). This information can be obtained from large-scale structure cosmological data, improved data on the tritium beta decay, or the long-baseline neutrino oscillation experiments. If the small mixing in the neutrino mixing matrix is negligible, cosmology might be the most promising arena to help in this puzzle.

#### Number of neutrino species

Neutrinos decouple early in cosmic history and contribute to a relativistic energy density with an effective number of species $N_{\nu ,\mathrm {eff}}=3.046$. Cosmology is sensitive to the physical energy density in relativistic particles in the early universe, which in the standard cosmological model includes only photons and neutrinos: $\omega _{\mathrm {rel}}=\omega _{\gamma }+N_{\nu ,\mathrm {eff}}\omega _{\nu }$, where $\omega _{\gamma }$ denotes the energy density in photons and is exquisitely constrained from the CMB, and $\omega _{\nu }$ is the energy density in one neutrino. Deviations from the standard value for $N_{\nu ,\mathrm {eff}}$ would signal non-standard neutrino features or additional relativistic species. $N_{\nu ,\mathrm {eff}}$ impacts the big bang nucleosynthesis epoch through its effect on the expansion rate; measurements of primordial light element abundances can constrain $N_{\nu ,\mathrm {eff}}$ and rely on physics at $T\sim \mathrm {MeV}$ (Bowen et al. 2002). In several non-standard models—e.g., decay of dark matter particles, axions, quintessence—the energy density in relativistic species can change at some later time. The energy density of free-streaming relativistic particles alters the epoch of matter-radiation equality and leaves therefore a signature in the CMB and in the matter-transfer function. However, there is a degeneracy between $N_{\nu ,\mathrm {eff}}$ and $\varOmega _m h^2$ from CMB data alone (given by the combination of these two parameters that leave matter-radiation equality unchanged) and between $N_{\nu ,\mathrm {eff}}$ and $\sigma _8$ and/or $n_s$. Large-scale structure surveys measuring the shape of the power spectrum at large scale can constrain independently the combination $\varOmega _m h$ and $n_s$, thus breaking the CMB degeneracy. Furthermore, anisotropies in the neutrino background affect the CMB anisotropy angular power spectrum at a level of $\sim \,20\%$ through the gravitational feedback of their free streaming damping and anisotropic stress contributions. Detection of this effect is now possible by combining CMB and large-scale structure observations. This yields an indication at more than $2\sigma$ level that there exists a neutrino background with characteristics compatible with what is expected under the cosmological standard model (Trotta and Melchiorri 2005; De Bernardis et al. 2008).

The forecasted errors on $N_{\nu ,\mathrm {eff}}$ for Euclid (with a Planck prior) are $\pm \,0.1$ at $1\sigma$ level (Kitching et al. 2008a), which is a factor $\sim \,5$ better than current constraints from CMB and LSS and about a factor $\sim \,2$ better than constraints from light element abundance and nucleosynthesis.

#### Model dependence

A recurring question is how much model dependent will the neutrino constraints be. It is important to recall that usually parameter-fitting is done within the context of a $\varLambda$CDM model and that the neutrino effects are seen indirectly in the clustering. Considering more general cosmological models, might degrade neutrino constraints, and vice versa, including neutrinos in the model might degrade dark-energy constraints. Here below we discuss the two cases of varying the total neutrino mass $\varSigma$ and the number of relativistic species $N_{\mathrm {eff}}$, separately. Possible effects of modified gravity models that could further degrade the neutrino mass constraints will not be discussed in this section.

#### $\varSigma$ forecasted error bars and degeneracies

In Carbone et al. (2011b) it is shown that, for a general model which allows for a non-flat universe, and a redshift dependent dark-energy equation of state, the $1\sigma$ spectroscopic errors on the neutrino mass $\varSigma$ are in the range 0.036–0.056 eV, depending on the fiducial total neutrino mass $\varSigma$, for the combination Euclid+Planck.

On the other hand, looking at the effect that massive neutrinos have on the dark-energy parameter constraints, it is shown that the total CMB + LSS dark-energy FoM decreases only by $\sim$ 15–25% with respect to the value obtained if neutrinos are supposed to be massless, when the forecasts are computed using the so-called “*P*(*k*)-method marginalized over growth-information” (see Methodology section), which therefore results to be quite robust in constraining the dark-energy equation of state.

For what concerns the parameter correlations, at the LSS level, the total neutrino mass $\varSigma$ is correlated with all the cosmological parameters affecting the galaxy power spectrum shape and BAO positions. When Planck priors are added to the Euclid constraints, all degeneracies are either resolved or reduced, and the remaining dominant correlations among $\varSigma$ and the other cosmological parameters are $\varSigma$–$\varOmega _{\mathrm {de}}$, $\varSigma$–$\varOmega _m$, and $\varSigma$–$w_a$, with the $\varSigma$–$\varOmega _{\mathrm {de}}$ degeneracy being the largest one.

*II.7.6.1 Hierarchy dependence*

In addition, the neutrino mass spectroscopic constraints depend also on the neutrino hierarchy. In fact, the $1\sigma$ errors on total neutrino mass for normal hierarchy are $\sim$ 17–20% larger than for the inverted one. It appears that the matter power spectrum is less able to give information on the total neutrino mass when the normal hierarchy is assumed as fiducial neutrino mass spectrum. This is similar to what found in Jiménez et al. (2010) for the constraints on the neutrino mass hierarchy itself, when a normal hierarchy is assumed as the fiducial one. On the other hand, when CMB information are included, the $\varSigma$-errors decrease by $\sim$ 35% in favor of the normal hierarchy, at a given fiducial value $\varSigma |_{\mathrm {fid}}$. This difference arises from the changes in the free-streaming effect due to the assumed mass hierarchy, and is in agreement with the results of Lesgourgues et al. (2004), which confirms that the expected errors on the neutrino masses depend not only on the sum of neutrino masses, but also on the order of the mass splitting between the neutrino mass states.

*II.7.6.2 Growth and incoherent peculiar velocity dependence*

$\varSigma$ spectroscopic errors stay mostly unchanged whether growth-information are included or marginalised over, and decrease only by 10%–20% when adding $f_g\sigma _8$ measurements. This result is expected, if we consider that, unlike dark-energy parameters, $\varSigma$ affects the shape of the power spectrum via a redshift-dependent transfer function *T*(*k*, *z*), which is sampled on a very large range of scales including the *P*(*k*) turnover scale, therefore this effect dominates over the information extracted from measurements of $f_g\sigma _8$. This quantity, in turn, generates new correlations with $\varSigma$ via the $\sigma _8$-term, which actually is anti-correlated with $M_\nu$ (Marulli et al. 2011). On the other hand, if we suppose that early dark-energy is negligible, the dark-energy parameters $\varOmega _{\mathrm {de}}$, $w_0$ and $w_a$ do not enter the transfer function, and consequently growth information have relatively more weight when added to constraints from *H*(*z*) and $D_A(z)$ alone. Therefore, the value of the dark-energy FoM does increase when growth-information are included, even if it decreases by a factor $\sim$ 50–60% with respect to cosmologies where neutrinos are assumed to be massless, due to the correlation among $\varSigma$ and the dark-energy parameters. As confirmation of this degeneracy, when growth-information are added and if the dark-energy parameters $\varOmega _{\mathrm {de}}$, $w_0$, $w_a$ are held fixed to their fiducial values, the errors $\sigma ({\varSigma })$ decrease from 0.056 to 0.028 eV, for Euclid combined with Planck.

We expect that dark-energy parameter errors are somewhat sensitive also to the effect of incoherent peculiar velocities, the so-called “Fingers of God” (FoG). This can be understood in terms of correlation functions in the redshift-space; the stretching effect due to random peculiar velocities contrasts the flattening effect due to large-scale bulk velocities. Consequently, these two competing effects act along opposite directions on the dark-energy parameter constraints (see methodology Sect. V).

On the other hand, the neutrino mass errors are found to be stable again at $\sigma ({\varSigma })=0.056$, also when FoG effects are taken into account by marginalising over $\sigma _v(z)$; in fact, they increase only by 10%–14% with respect to the case where FoG are not taken into account.

Finally, in Table 19 we summarize the dependence of the $\varSigma$-errors on the model cosmology, for Euclid combined with Planck.^19^ We conclude that, if $\varSigma$ is $>\,0.1\,\hbox {eV}$, spectroscopy with Euclid will be able to determine the neutrino mass scale independently of the model cosmology assumed, provided GR is correct and dark energy does not interact with other species (Baldi et al. 2014). If $\varSigma$ is $<\,0.1\,\hbox {eV}$, the sum of neutrino masses, and in particular the minimum neutrino mass required by neutrino oscillations, can be measured in the context of a $\varLambda$CDM model.

**Table 19:** $\sigma (M_\nu )$ and $\sigma (N_{\mathrm {eff}})$ marginalized errors from LSS + CMB

General cosmology						
Fiducial $\rightarrow$	$\varSigma =0.3\mathrm {\, eV}^a$	$\varSigma =0.2\mathrm {\, eV}^a$	$\varSigma =0.125\mathrm {\, eV}^b$	$\varSigma =0.125\mathrm {\, eV}^c$	$\varSigma =0.05\mathrm {\, eV}^b$	$N_{\mathrm {eff}}=3.04^d$
$\hbox {Euclid}+\hbox {Planck}$	0.0361	0.0458	0.0322	0.0466	0.0563	0.0862
$\varLambda$CDM cosmology						
$\hbox {Euclid}+\hbox {Planck}$	0.0176	0.0198	0.0173	0.0218	0.0217	0.0224

${}^a$ For degenerate spectrum: $m_1\approx m_2\approx m_3$

${}^b$ For normal hierarchy: $m_3\ne 0$, $m_1\approx m_2\approx 0$

${}^c$ For inverted hierarchy: $m_1\approx m_2$, $m_3\approx 0$

${}^d$ Fiducial cosmology with massless neutrinos

#### $N_{\mathrm {eff}}$ forecasted errors and degeneracies

Regarding the $N_{\mathrm {eff}}$ spectroscopic errors, Carbone et al. (2011b) finds $\sigma (N_{\mathrm {eff}})\sim \,0.56$ from Euclid, and $\sigma (N_{\mathrm {eff}})\sim \,0.086$, for Euclid+Planck. Concerning the effect of $N_{\mathrm {eff}}$ uncertainties on the dark-energy parameter errors, the CMB + LSS dark-energy FoM decreases only by $\sim \,5\%$ with respect to the value obtained holding $N_{\mathrm {eff}}$ fixed at its fiducial value, meaning that also in this case the “*P*(*k*)-method marginalized over growth–information” is not too sensitive to assumptions about model cosmology when constraining the dark-energy equation of state.

About the degeneracies between $N_{\mathrm {eff}}$ and the other cosmological parameters, it is necessary to say that the number of relativistic species gives two opposite contributions to the observed power spectrum $P_{\mathrm {obs}}$ (see methodology Sect. V), and the total sign of the correlation depends on the dominant one, for each single cosmological parameter. In fact, a larger $N_{\mathrm {eff}}$ value suppresses the transfer function *T*(*k*) on scales $k\le k_{\max }$. On the other hand, a larger $N_{\mathrm {eff}}$ value also increases the Alcock–Paczyński prefactor in $P_{\mathrm {obs}}$. For what concerns the dark-energy parameters $\varOmega _{\mathrm {de}}$, $w_0$, $w_a$, and the dark-matter density $\varOmega _m$, the Alcock–Paczynski prefactor dominates, so that $N_{\mathrm {eff}}$ is positively correlated to $\varOmega _{\mathrm {de}}$ and $w_a$, and anti-correlated to $\varOmega _m$ and $w_0$. In contrast, for the other parameters, the *T*(*k*) suppression produces the larger effect and $N_{\mathrm {eff}}$ results to be anti-correlated to $\varOmega _b$, and positively correlated to *h* and $n_s$. The degree of the correlation is very large in the $n_s$-$N_{\mathrm {eff}}$ case, being of the order $\sim \, 0.8$ with and without Planck priors. For the remaining cosmological parameters, all the correlations are reduced when CMB information are added, except for the covariance $N_{\mathrm {eff}}$-$\varOmega _{\mathrm {de}}$, as happens also for the $M_\nu$-correlations. To summarize, after the inclusion of Planck priors, the remaining dominant degeneracies among $N_{\mathrm {eff}}$ and the other cosmological parameters are $N_{\mathrm {eff}}$-$n_s$, $N_{\mathrm {eff}}$-$\varOmega _{\mathrm {de}}$, and $N_{\mathrm {eff}}$-*h*, and the forecasted error is $\sigma (N_{\mathrm {eff}})\sim \,0.086$, from Euclid+Planck. Finally, if we fix to their fiducial values the dark-energy parameters $\varOmega _{\mathrm {de}}$, $w_0$ and $w_a$, $\sigma (N_{\mathrm {eff}})$ decreases from 0.086 to 0.048, for the combination Euclid+Planck. However, it has to be noticed that if $N_{\mathrm {eff}}$ is allowed to vary, then the shape of the matter power spectrum in itself cannot constrain $\varOmega _m h$. Indeed, in $\varLambda$CDM models, the power spectrum constrains $\varOmega _m h$ because the turning point $k_\mathrm {eq}$ corresponds to the comoving Hubble rate at equality. If the radiation content is known, then $k_\mathrm {eq}$ depends only on $\varOmega _m h$. However, if the radiation content is unknown, then $k_\mathrm {eq}$ is not linked to a unique value of $\varOmega _m h$ (Abazajian et al. 2012b). The fact that one can use a combination of CMB (excluding the damping tail) and matter power spectrum data to break the $N_{\mathrm {eff}}$–$\varOmega _m h^2$ degeneracy is due to a decreasing baryon fraction $f_b = \varOmega _b h^2/\varOmega _m h^2$ when $N_{\mathrm {eff}}$ is increased (while keeping $z_\mathrm {eq}$ fixed) (e.g., Bashinsky and Seljak 2004).

#### Nonlinear effects of massive cosmological neutrinos on bias, P(k) and RSD

In general, forecasted errors are obtained using techniques, like the Fisher-matrix approach, that are not particularly well suited to quantifying systematic effects. These techniques forecast only statistical errors, which are meaningful as long as they dominate over systematic errors. Possible sources of systematic errors of major concern are the effects of nonlinearities and galaxy bias.

The description of nonlinearities in the matter power spectrum in the presence of massive neutrinos has been addressed in several different ways: Wong (2008), Saito et al. (2008, 2009, 2011) have used perturbation theory, Lesgourgues et al. (2009) the time-RG flow approach and Brandbyge et al. (2008), Brandbyge and Hannestad (2009), Brandbyge et al. (2010), Viel et al. (2010) different schemes of *N*-body simulations. Another nonlinear scheme that has been examined in the literature is the halo model. This has been applied to massive neutrino cosmologies in Abazajian et al. (2005a) and Hannestad et al. (2005, 2006).

On the other hand, galaxy/halo bias is known to be almost scale-independent only on large, linear scales, but to become nonlinear and scale-dependent for small scales and/or for very massive haloes. From the above discussion and references, it is clear that the effect of massive neutrinos on the galaxy power spectrum in the nonlinear regime must be explored via *N*-body simulations to encompass all the relevant effects.

Here below we focus on the behavior of the DM halo mass function (MF), the DM halo bias, and the redshift-space distortions (RSD), in the presence of a cosmological background of massive neutrinos. To this aim, Brandbyge et al. (2010) and Marulli et al. (2011) have analysed a set of large *N*-body hydrodynamical simulations, developed with an extended version of the code Gadget-3 (Viel et al. 2010), which take into account the effect of massive free-streaming neutrinos on the evolution of cosmic structures (Fig. 42).

**Fig. 43:**
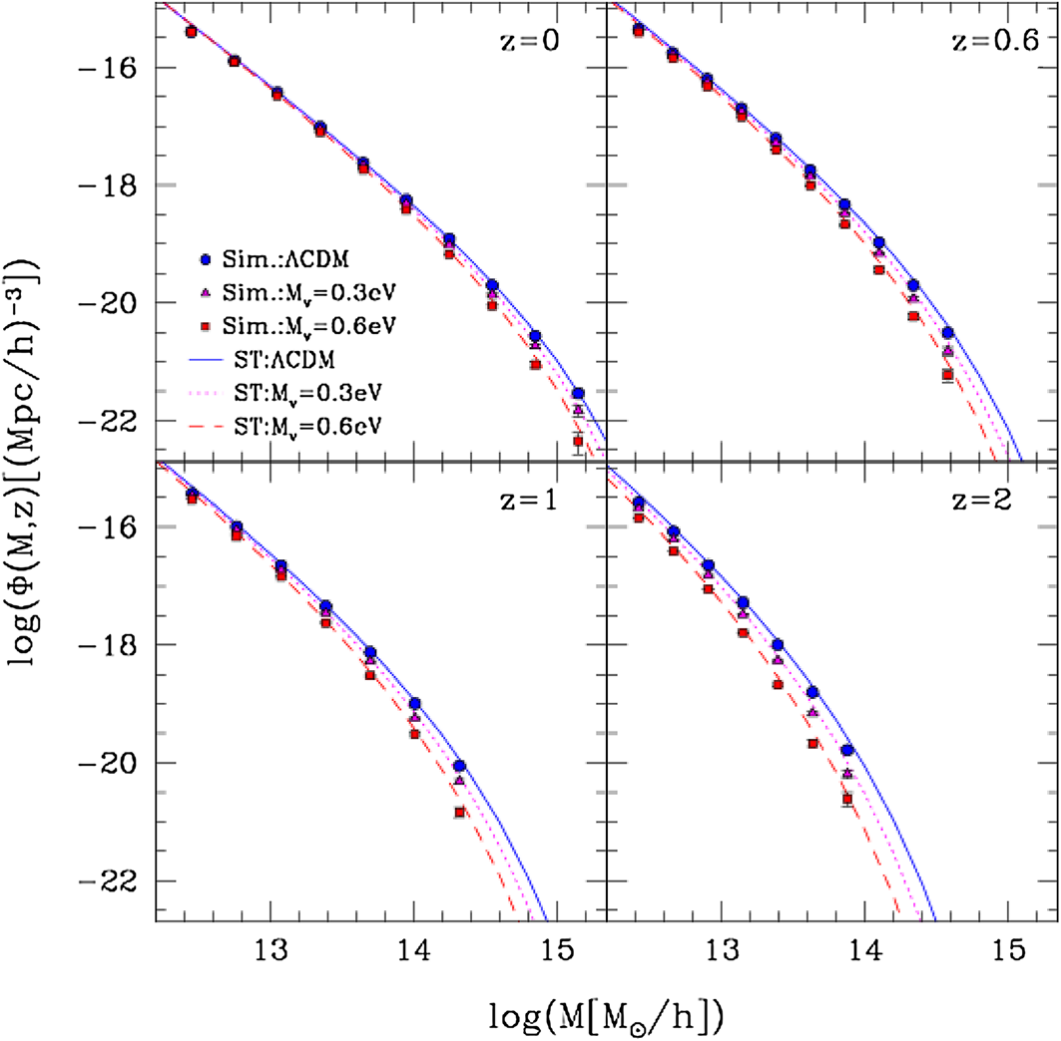
DM halo mass function (MF) as a function of $\varSigma$ and redshift. MF of the SUBFIND haloes in the $\varLambda$CDM *N*-body simulation (blue circles) and in the two simulations with $\varSigma =0.3\mathrm {\ eV}$ (magenta triangles) and $\varSigma =0.6\,\mathrm {\ eV}$ (red squares). The blue, magenta and red lines show the halo MF predicted by Sheth and Tormen (2002), where the variance in the density fluctuation field, $\sigma (M)$, at the three cosmologies, $\varSigma =0,0.3,0.6\mathrm {\ eV}$, has been computed with the software camb (Lewis et al. 2000b)

**Fig. 44:**
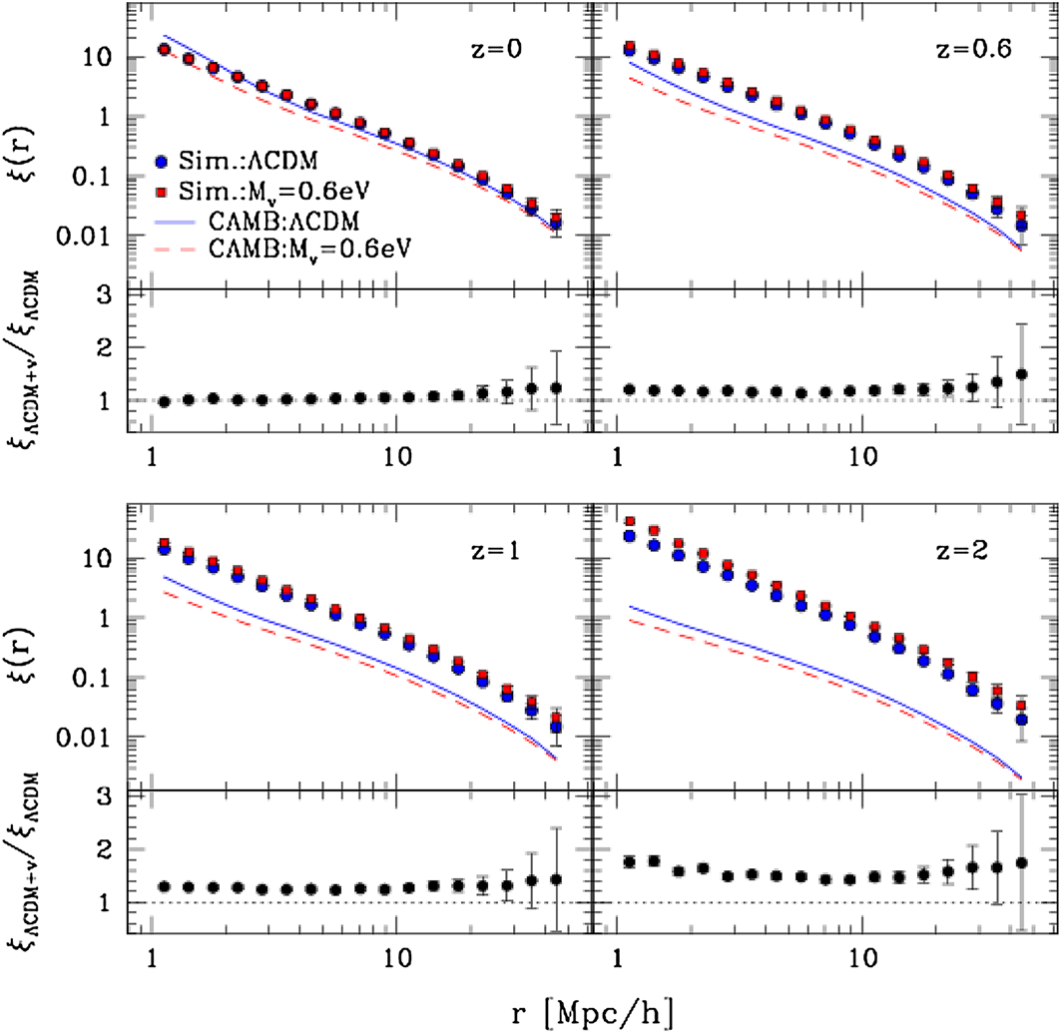
Real space two-point auto-correlation function of the DM haloes in the $\varLambda$CDM *N*-body simulation (blue circles) and in the simulation with $\varSigma =0.6\mathrm {\ eV}$ (red squares). The blue and red lines show the DM correlation function computed using the camb matter power spectrum with $\varSigma =0$ and $\varSigma =0.6\mathrm {\ eV}$, respectively. The bottom panels show the ratio between the halo correlation function extracted from the simulations with and without massive neutrinos

**Fig. 45:**
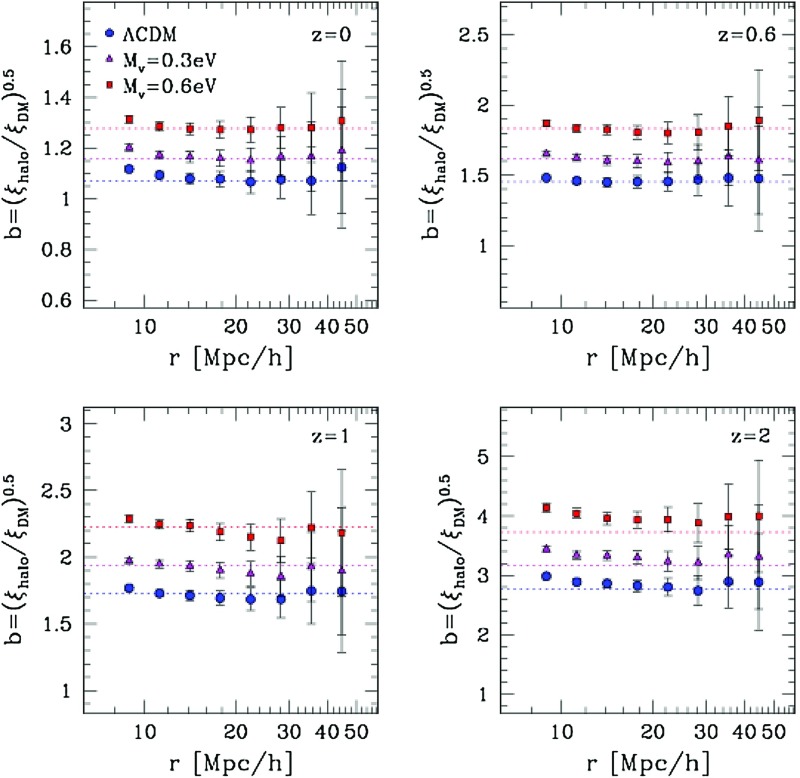
Bias of the DM haloes in the $\varLambda$CDM *N*-body simulation (blue circles) and in the two simulations with $\varSigma =0.3\mathrm {\ eV}$ (magenta triangles) and $\varSigma =0.6\,\mathrm {\ eV}$ (red squares). Dotted lines are the theoretical predictions of Sheth et al. (2001)

**Fig. 46:**
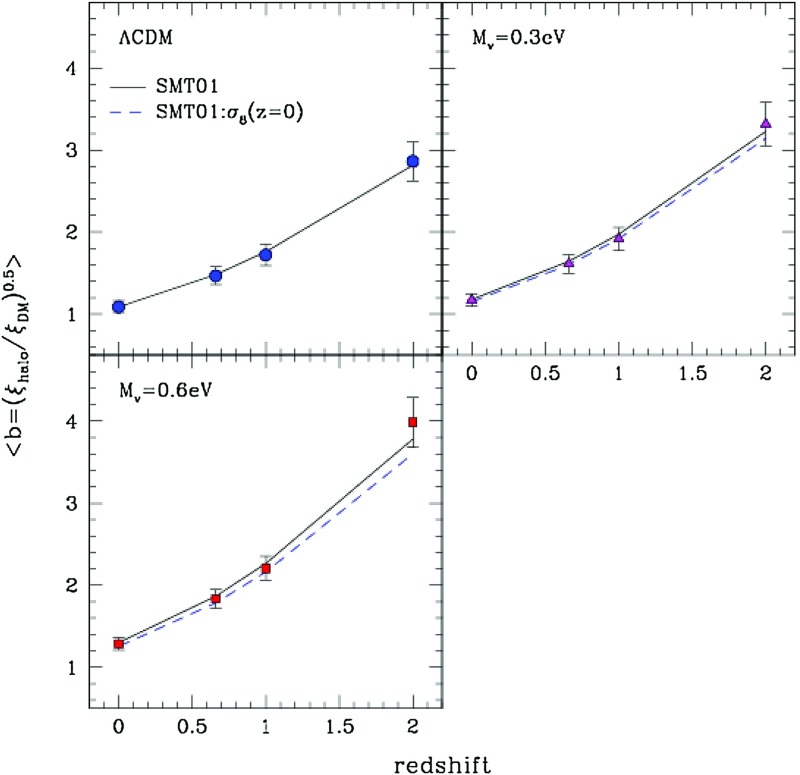
Mean bias (averaged in $10<r\,[\mathrm {Mpc}/h]<50$) as a function of redshift compared with the theoretical predictions of Sheth and Tormen (2002). Here the dashed lines represent the theoretical expectations for a $\varLambda$CDM cosmology renormalized with the $\sigma _8$ value of the simulations with a massive neutrino component

**Fig. 47:**
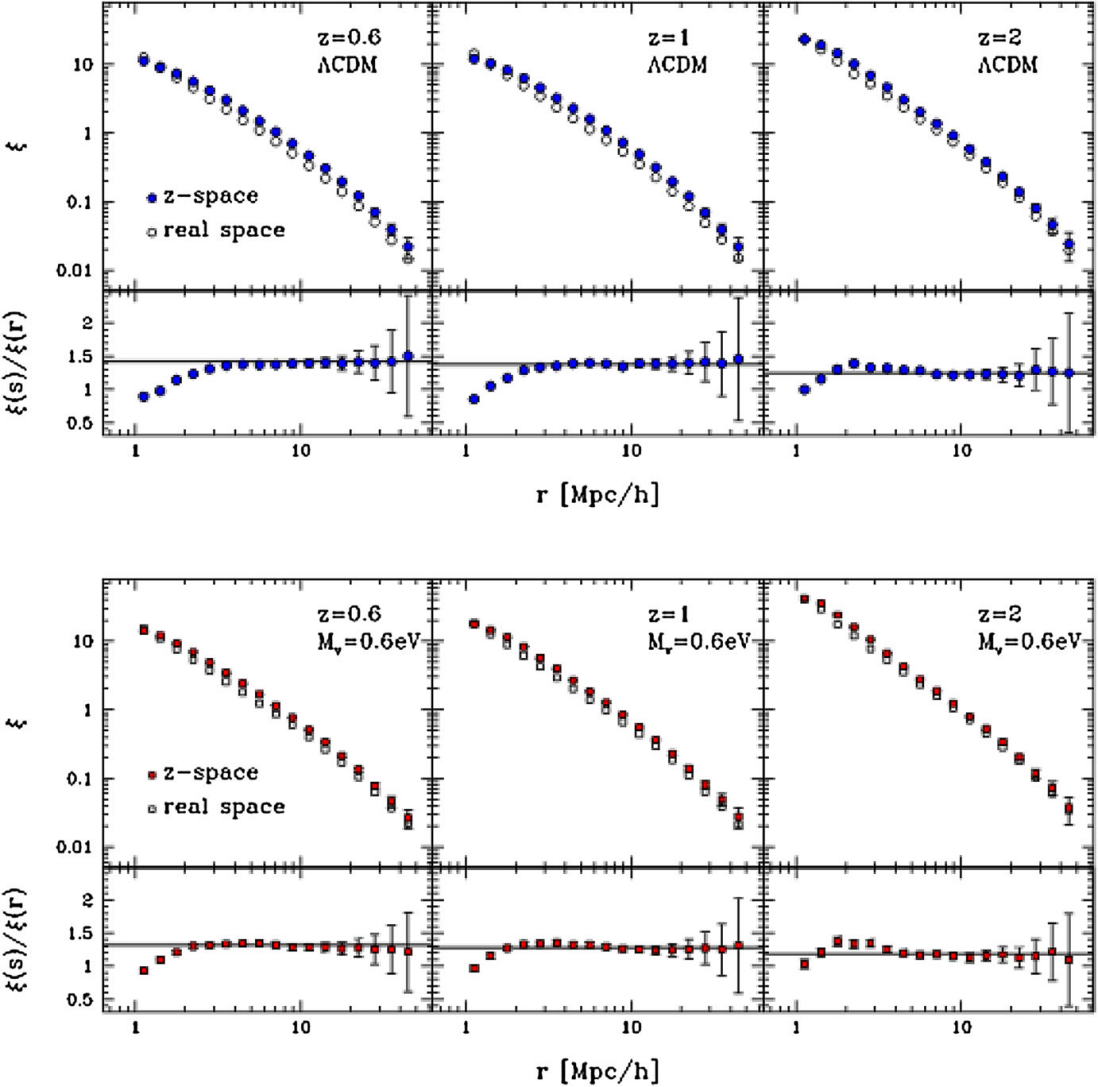
Two-point auto-correlation function in real and redshift space of the DM haloes in the $\varLambda$CDM *N*-body simulation (blue circles) and in the simulation with $\varSigma =0.6\mathrm {\ eV}$ (red squares). The bottom panels show the ratio between them, compared with the theoretical expectation

**Fig. 48:**
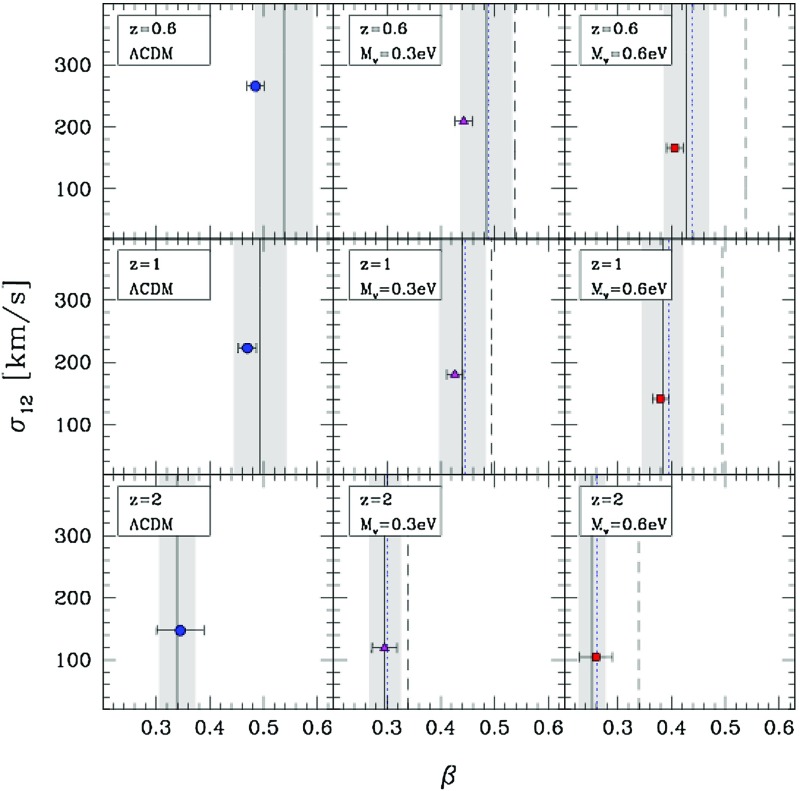
Best-fit values of $\beta$-$\sigma _{12}$, as a function of $\varSigma$ and redshift (points), compared with the theoretical prediction (grey shaded area). The blue dotted lines show the theoretical prediction for a $\varLambda$CDM cosmology normalised to the $\sigma _8$ value of the simulation with a massive neutrino component

The pressure produced by massive neutrino free-streaming contrasts the gravitational collapse which is the basis of cosmic structure formation, causing a significant suppression in the average number density of massive structures. This effect can be observed in the high mass tail of the halo MF in Fig. 43, as compared with the analytic predictions of Sheth and Tormen (2002) (ST), where the variance in the density fluctuation field, $\sigma (M)$, has been computed via camb (Lewis et al. 2000b), using the same cosmological parameters of the simulations. In particular, here the MF of sub-structures is shown, identified using the subfind package (Springel et al. 2001), while the normalization of the matter power spectrum is fixed by the dimensionless amplitude of the primordial curvature perturbations $\varDelta ^2_\mathcal{R}(k_0)|_{\mathrm {fid}}=2.3\times 10^{-9}$, evaluated at a pivot scale $k_0=0.002/\mathrm {Mpc}$ (Larson et al. 2011), which has been chosen to have the same value both in the $\varLambda$CDM$\nu$ and in the $\varLambda$CDM cosmologies.

In Fig. 43, two fiducial neutrino masses have been considered, $\varSigma =0.3$ and $\varSigma =0.6\mathrm {\ eV}$. From the comparison of the corresponding MFs, we confirm the theoretical predictions, i.e., that the higher the neutrino mass is, the larger the suppression in the comoving number density of DM haloes becomes. These results have been overall confirmed by recent numerical investigations (Villaescusa-Navarro et al. 2014; Castorina et al. 2014; Costanzi et al. 2013). Moreover, it was shown that an even better agreement with numerical simulations can be obtained by using the linear CDM power spectrum, instead of the total matter one (see also Ichiki and Takada 2012).

Massive neutrinos also strongly affect the spatial clustering of cosmic structures. A standard statistic generally used to quantify the degree of clustering of a population of sources is the two-point auto-correlation function. Although the free-streaming of massive neutrinos causes a suppression of the matter power spectrum on scales *k* larger than the neutrino free-streaming scale, the halo bias is significantly enhanced. This effect can be physically explained thinking that, due to neutrino structure suppression, the same halo bias would correspond, in a $\varLambda$CDM cosmology, to more massive haloes (than in a $\varLambda$CDM$\nu$ cosmology), which as known are typically more clustered.

This effect is evident in Fig. 44 which shows the two-point DM halo correlation function measured with the Landy and Szalay (1993) estimator, compared to the matter correlation function. In particular, the clustering difference between he $\varLambda$CDM and $\varLambda$CDM$\nu$ cosmologies increases at higher redshifts, as it can be observed from Figs. 44, 45, and 46. Note also the effect of nonlinearities on the bias, which clearly starts to become scale-dependent for separations $r<20\mathrm {\ Mpc}/h$ (see also Villaescusa-Navarro et al. 2014; Castorina et al. 2014; Costanzi et al. 2013).

There are indications from 3D weak lensing in the CFHTLenS survey (Kitching et al. 2014) that the matter power suppressed is suppressed with respect to the $\varLambda$CDM expectation in the wavenumber range 1–10 *h* Mpc$^{-1}$, which may be a hint of either massive neutrinos, or feedback from AGN, or both. Euclid will be able to probe this regime with much greater precision, and potentially disentangle the two effects.

RSD are also strongly affected by massive neutrinos. Figure 47 shows the real and redshift space correlation functions of DM haloes as a function of neutrino mass. The effect of massive neutrinos is particularly evident when the correlation function is measured as a function of the two directions perpendicular and parallel to the line of sight. The value of the linear growth rate that can be derived by modelling galaxy clustering anisotropies can be greatly suppressed with respect to the value expected in a $\varLambda$CDM cosmology. Indeed, neglecting the relic massive neutrino background in data analysis might induce a bias in the inferred growth rate, from which a potentially fake signature of modified gravity might be inferred. Figure 48 demonstrates this point, showing the best-fit values of $\beta$ and $\sigma _{12}$, as a function of $\varSigma$ and redshift, where $\beta = {\frac{f(\varOmega _{\mathrm {M}})}{b_\mathrm {eff}}}$, $b_{\mathrm {eff}}$ being the halo effective linear bias factor, $f(\varOmega _{\mathrm {M}})$ the linear growth rate and $\sigma _{12}$ the pairwise velocity dispersion.

### Coupling between dark energy and neutrinos

As we have seen in Sect. I.5.3, it is interesting to consider the possibility that dark energy, seen as a dynamical scalar field (quintessence), may interact with other components in the universe. In this section we focus on the possibility that a coupling may exist between dark energy and neutrinos.

The idea of such a coupling has been addressed and developed by several authors within MaVaNs theories first (Fardon et al. 2004; Peccei 2005; Bi et al. 2005; Afshordi et al. 2005; Weiner and Zurek 2006; Das and Weiner 2011; Takahashi and Tanimoto 2006; Spitzer 2006; Bjælde et al. 2008; Brookfield et al. 2006b, a) and more recently within growing neutrino cosmologies (Amendola et al. 2008a; Wetterich 2007; Mota et al. 2008; Wintergerst et al. 2010; Wintergerst and Pettorino 2010; Pettorino et al. 2010; Brouzakis et al. 2011). It has been shown that neutrinos can play a crucial role in cosmology, setting naturally the desired scale for dark energy. Interestingly, a coupling between neutrinos and dark energy may help solving the ‘why now’ problem, explaining why dark energy dominates only in recent epochs. The coupling follows the description illustrated in Sect. I.5.3 for a general interacting dark-energy cosmology, where now $m_\nu =m_\nu (\phi )$.

Typically, in growing neutrino cosmologies, the function $m_\nu (\phi )$ is such that the neutrino mass grows with time from low, nearly massless values (when neutrinos are non-relativistic) up to present masses in a range in agreement with current observations (see the previous section of this review for latest bounds on neutrino masses). The key feature of growing neutrino models is that the amount of dark energy today is triggered by a cosmological event, corresponding to the transition from relativistic to non-relativistic neutrinos at redshift $z_\mathrm {NR}\sim \,5{-}10$. As long as neutrinos are relativistic, the coupling plays no role on the dynamics of the scalar field, which follows attractor solutions of the type described in Sect. I.5.3. From there on, the evolution of dark energy resembles that of a cosmological constant, plus small oscillations of the coupled dark energy-neutrino fluid. As a consequence, when a coupling between dark energy and neutrinos is active, the amount of dark energy and its equation of state today are strictly connected to the present value of the neutrino mass.

The interaction between neutrinos and dark energy is a nice and concrete example of the significant imprint that dynamical coupled dark energy can leave on observables and in particular on structure formation and on the cosmic microwave background. This is due to the fact that the coupling, playing a role only after neutrinos become non-relativistic, can reach relatively high values as compared to gravitational attraction. Typical values of $\beta$ are order 50–100 or even more such that even the small fraction of cosmic energy density in neutrinos can have a substantial influence on the time evolution of the quintessence field. During this time the fifth force can be of order $10^2{-}10^4$ times stronger than gravity. The neutrino contribution to the gravitational potential influences indirectly also dark matter and structure formation, as well as CMB, via the Integrated Sachs–Wolfe effect and the nonlinear Rees–Sciama effect, which is non-negligible at the scales where neutrinos form stable lumps. Furthermore, backreaction effects can substantially modify the growth of large scale neutrino lumps, with effects which are much larger than in the dark matter case. The presence of a fifth force due to an interaction between neutrinos and dark energy can lead to remarkably peculiar differences with respect to a cosmological constant scenario.

Here, we just recall some of the typical features that can arise when such an interaction is active:

- existence of very large structures, order $10{-}500\mathrm {\ Mpc}$ (Afshordi et al. 2005; Mota et al. 2008; Wintergerst et al. 2010; Wintergerst and Pettorino 2010; Pettorino et al. 2010);
- enhanced ISW effect, drastically reduced when taking into account nonlinearities (Pettorino et al. 2010): information on the gravitational potential is a good mean to constrain the range of allowed values for the coupling $\beta$;
- large-scale anisotropies and enhanced peculiar velocities (Watkins et al. 2009; Ayaita et al. 2009);
- the influence of the gravitational potential induced by the neutrino inhomogeneities can affect BAO in the dark-matter spectra (Brouzakis et al. 2011).

Investigation of structure formation at large scales (order $1{-}100\mathrm {\ Mpc}$) as well as cross correlation with CMB are crucial in order to disentangle coupled neutrino-quintessence cosmologies from a cosmological constant scenario. Detection of a population of very large-scale structures could pose serious difficulties to the standard framework and open the way to the existence of a new cosmological interaction stronger than gravity.

### Unified dark matter

The appearance of two unknown components in the standard cosmological model, dark matter and dark energy, has prompted discussion of whether they are two facets of a single underlying dark component. This concept goes under the name of quartessence (Makler et al. 2003), or unified dark matter (UDM). *A priori* this is attractive, replacing two unknown components with one, and in principle it might explain the ‘why now?’ problem of why the energy densities of the two components are similar (also referred to as the coincidence problem). Many UDM models are characterized by a sound speed, whose value and evolution imprints oscillatory features on the matter power spectrum, which may be detectable through weak lensing or BAO signatures with Euclid.

The field is rich in UDM models (see Bertacca et al. 2010, for a review and for references to the literature). The models can grow structure, as well as providing acceleration of the universe at late times. In many cases, these models have a non-canonical kinetic term in the Lagrangian, e.g., an arbitrary function of the square of the time derivative of the field in a homogeneous and isotropic background. Early models with acceleration driven by kinetic energy (*k*-inflation Armendariz-Picon et al. 1999; Garriga and Mukhanov 1999; Bose and Majumdar 2009) were generalized to more general Lagrangians (*k*-essence; e.g., Armendariz-Picon et al. 2000, 2001; Scherrer 2004). For UDM, several models have been investigated, such as the generalized Chaplygin gas (Kamenshchik et al. 2001; Bento et al. 2002; Bilić et al. 2002; Zhang et al. 2006; Popov 2010), although these may be tightly constrained due to the finite sound speed (e.g. Amendola et al. 2003a; Bento et al. 2003; Sandvik et al. 2004; Zhu 2004). Vanishing sound speed models however evade these constraints (e.g., the silent Chaplygin gas of Amendola et al. 2005b). Other models consider a single fluid with a two-parameter equation of state (e.g., Balbi et al. 2007), models with canonical Lagrangians but a complex scalar field (Arbey 2006), models with a kinetic term in the energy–momentum tensor (Gao et al. 2010; Chimento and Forte 2008), models based on a DBI action (Chimento et al. 2010), models which violate the weak equivalence principle (Füzfa and Alimi 2007) and models with viscosity (Dou and Meng 2011). Finally, there are some models which try to unify inflation as well as dark matter and dark energy (Capozziello et al. 2006; Nojiri and Odintsov 2008; Liddle et al. 2008; Lin 2009; Henriques et al. 2009).

A requirement for UDM models to be viable is that they must be able to cluster to allow structure to form. A generic feature of the UDM models is an effective sound speed, which may become significantly non-zero during the evolution of the universe, and the resulting Jeans length may then be large enough to inhibit structure formation. The appearance of this sound speed leads to observable consequences in the CMB as well, and generally speaking the speed needs to be small enough to allow structure formation and for agreement with CMB measurements. In the limit of zero sound speed, the standard cosmological model is recovered in many models. Generally the models require fine-tuning, although some models have a fast transition between a dark matter only behavior and $\varLambda$CDM. Such models (Piattella et al. 2010) can have acceptable Jeans lengths even if the sound speed is not negligible.

#### Theoretical background

An action which is applicable for most UDM models, with a single scalar field $\varphi$, is

II.9.1 $$\begin{aligned} S=\int \mathrm {d}^4x\sqrt{-g}\left[ \frac{R}{2}+\mathcal{L}(\varphi ,X)\right] , \end{aligned}$$

where

II.9.2 $$\begin{aligned} X\equiv -\frac{1}{2}\nabla _\mu \varphi \nabla ^\mu \varphi \end{aligned}$$

and $\nabla$ indicates covariant differentiation. This leads to an energy density which is $\rho =2X\,\partial p/\partial X-p$, and hence an equation-of-state parameter $w \equiv p/\rho$ (in units of $c=1$) given by

II.9.3 $$\begin{aligned} w=\frac{p}{2X\,\partial p/\partial X-p}, \end{aligned}$$

and $p={{\mathcal {L}}}$. A full description of the models investigated and Lagrangians considered is beyond the scope of this work; the reader is directed to the review by Bertacca et al. (2010) for more details. Lagrangians of the form

**Fig. 49:**
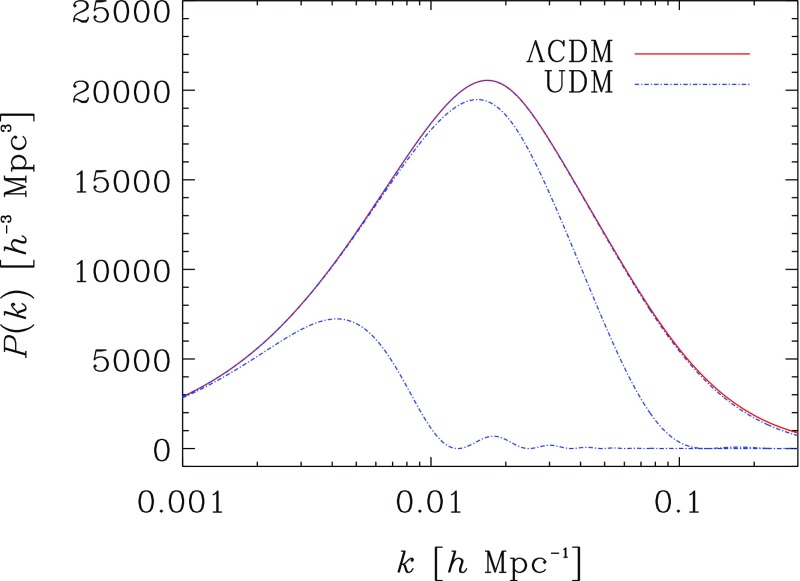
The $z=0$ matter power spectrum arising in UDM models with a Lagrangian given by Eq. (II.9.4). $\varLambda$CDM is solid, and UDM models with $c_\infty =10^{-1}, 10^{-2}, 10^{-3}$ are shown from bottom to top. Image reproduced by permission from Camera et al. (2011c), copyright by the authors

II.9.4 $$\begin{aligned} {{\mathcal {L}}}(\varphi ,X) = f(\varphi )g(X)-V(\varphi ), \end{aligned}$$

where *g*(*X*) is a Born–Infeld kinetic term, were considered in a Euclid-like context by Camera et al. (2011c), and models of this form can avoid a strong ISW effect which is often a problem for UDM models (see Bertacca et al. 2008, and references therein). This model is parameterized by a late-time sound speed, $c_\infty$, and its influence on the matter power spectrum is illustrated in Fig. 49. For zero sound speed $\varLambda$CDM is recovered.

#### Euclid observables

Of interest for Euclid are the weak lensing and BAO signatures of these models, although the supernova Hubble diagram can also be used (Thakur et al. 2009). The observable effects come from the power spectrum and the evolution of the equation-of-state parameter of the unified fluid, which affects distance measurements. The observational constraints of the generalized Chaplygin gas have been investigated (Park et al. 2010), with the model already constrained to be close to $\varLambda$CDM with SDSS data and the CMB. The effect on BAO measurements for Euclid has been calculated by (Camera et al. 2012), whereas the weak lensing effect has been considered for non-canonical UDM models (Camera et al. 2011c). The change in shape and oscillatory features introduced in the power spectrum allow the sound speed parameter to be constrained very well by Euclid, using 3D weak lensing (Heavens 2003; Kitching et al. 2007) with errors $\sim \, 10^{-5}$ (see also Camera et al. 2009, 2012).

### Dark energy and dark matter

In Sect. I.5, we have illustrated the possibility that dark energy, seen as a dynamical scalar field (quintessence), may interact with other components in the universe. When starting from an action such as Eq. (I.5.20), the species which interact with quintessence are characterized by a mass function that changes in time (Kodama and Sasaki 1984; Amendola 2000a, 2004; Pettorino and Baccigalupi 2008). Here, we consider the case in which the evolution of cold dark matter (CDM) particles depends on the evolution of the dark-energy scalar field. In this case the general framework seen in Sect. I.5 is specified by the choice of the function $m_c=m_c(\phi )$. The coupling is not constrained by tests of the equivalence principle and solar system constraints, and can therefore be stronger than the coupling with baryons. Typical values of $\beta$ presently allowed by observations (within current CMB data) are within the range $0< \beta < 0.06$ at 95% CL for a constant coupling and an exponential potential, (Bean et al. 2008b; Amendola et al. 2003b; Amendola 2004; Amendola and Quercellini 2003), or possibly more if neutrinos are taken into account or more realistic time-dependent choices of the coupling are used (La Vacca et al. 2009; Kristiansen et al. 2010). As mentioned in Sect. I.5.3, this framework is generally referred to as ‘coupled quintessence’ (CQ). Various choices of couplings have been investigated in the literature, including constant $\beta$ (Amendola 2000a, 2004; Mangano et al. 2003; Koivisto 2005; Guo et al. 2007; Quartin et al. 2008; Quercellini et al. 2008; Pettorino and Baccigalupi 2008) and varying couplings (Baldi 2011b), with effects on Supernovæ, CMB and cross-correlation of the CMB and LSS (Bean et al. 2008b; Amendola et al. 2003b; Amendola 2004; Amendola and Quercellini 2003; La Vacca et al. 2009; Kristiansen et al. 2010; Mainini and Mota 2012).

The presence of a coupling (and therefore, of a fifth force acting among dark matter particles) modifies the expansion of the universe, linear perturbations and most relevantly, structure formation. Coupled quintessence is a concrete model in which a non-negligible amount of dark energy is present at early times. The presence of such an early dark-energy component is accompanied specific features, as illustrated in Sect. I.5 for a general framework:

1. a fifth force $\varvec{\nabla } \left[ \varPhi _\alpha + \beta \phi \right]$ with an effective $\tilde{G}_{\alpha } = G_{N}[1+2\beta ^2(\phi )]$;
2. a velocity-dependent term $\tilde{H}\mathbf {v}_{\alpha } \equiv H \left( 1 - {\beta (\phi )} \frac{\dot{\phi }}{H}\right) \mathbf {v}_{\alpha }$;
3. a time-dependent mass for each particle $\alpha$, evolving according to Eq. (I.5.25).

All these effects, and in particular the first two, contribute significantly to structure formation. Note that the second and third terms are not independent of each other as they are a direct consequence of momentum conservation. Depending on the function $m_c(\phi )$, and therefore $\beta (\phi )$, the first two terms can partially balance: the fifth force increases gravitational attraction whilst the velocity-dependent term, if the CDM mass decreases with time, tries to dilute the concentration of the virialized haloes. In particular, a striking difference between constant and variable-coupling models concerning the interplay of all these three effects has been highlighted in Baldi (2011b): whilst for constant couplings only the latter two effects can alter the virial equilibrium of an already-collapsed object, for the case of a variable coupling the time evolution of the effective gravitational constant can also modify the virial status of a halo, and can either enhance or counteract the effect of reducing halo concentrations (for decreasing and increasing couplings, respectively). Nonlinear evolution within coupled quintessence cosmologies has been addressed using various methods of investigation, such as spherical collapse (Mainini and Bonometto 2006; Wintergerst and Pettorino 2010; Manera and Mota 2006; Koivisto 2005; Sutter and Ricker 2008; Abdalla et al. 2009; Bertolami et al. 2009) and alternative semi-analytic methods (Saracco et al. 2010; Amendola and Quercellini 2004). *N*-body and hydro-simulations have also been done (Macciò et al. 2004; Baldi et al. 2010; Baldi 2011b; Baldi and Pettorino 2011; Baldi and Viel 2010; Li et al. 2011; Baldi 2011a; Li and Barrow 2011b; Zhao et al. 2010; Marulli et al. 2012; Giocoli et al. 2013; Moresco et al. 2014).

We list here briefly the main observable features typical of this class of models:

- enhanced ISW effect (Amendola 2000a, 2004; Mainini and Mota 2012); such effects may be partially reduced when taking into account nonlinearities, as described in Pettorino et al. (2010);
- increase in the number counts of massive clusters at high redshift (Baldi and Pettorino 2011);
- scale-dependent bias between baryons and dark matter, which behave differently if only dark matter is coupled to dark energy (Baldi et al. 2010; Baldi 2011a);
- less steep inner core halo profiles (depending on the interplay between fifth force and velocity-dependent terms) (Baldi et al. 2010; Baldi 2011a, b; Li et al. 2011; Li and Barrow 2011b);
- lower concentration of the halos (Baldi et al. 2010; Baldi 2011b; Li and Barrow 2011b);
- voids are emptier when a coupling is active (Baldi and Viel 2010).

As discussed in Sect. I.6.1, when a variable coupling $\beta (\phi )$ is active the relative balance of the fifth-force and other dynamical effects depends on the specific time evolution of the coupling strength. Under such conditions, certain cases may also lead to the opposite effect of larger halo inner overdensities and higher concentrations, as in the case of a steeply growing coupling function (see Baldi 2011b). Alternatively, the coupling can be introduced by choosing directly a covariant stress–energy tensor, treating dark energy as a fluid in the absence of a starting action (Mangano et al. 2003; Väliviita et al. 2008; Caldera-Cabral et al. 2009a, b; Schaefer et al. 2008; Väliviita et al. 2010; Majerotto et al. 2010; Gavela et al. 2009, 2010). For an illustration of nonlinear effects in the presence of a coupling see Sect. I.6.

### Ultra-light scalar fields

Ultra-light scalar fields arise generically in high energy physics, most commonly as axions or other axion-like particles (ALPs). They are the pseudo-goldstone bosons (PGBs) of spontaneously broken symmetries. Their mass remains protected to all loop orders by a shift symmetry, which is only weakly broken to give the fields a mass and potential, through non perturbative effects. Commonly these effects are presumed to be caused by instantons, as in the case of the QCD axion, but the potential can also be generated in other ways, for example, in the study of quintessence (Panda et al. 2011). Here, we will be considering a general scenario, motivated by the suggestions of Arvanitaki et al. (2010) and Hu et al. (2000), where an ultralight scalar field constitutes some fraction of the dark matter, and we make no detailed assumptions about its origin.

#### Phenomenology and motivation

If the DM is light then either thermal free-streaming (as for massive neutrinos or WDM) or non-thermal quantum pressure (as is the case for ultra-light scalars) can lead to potentially observable effects in the large scale structure. The mass range of interest for ultra-light scalars is:

II.11.1 $$\begin{aligned} 10^{-33}\mathrm {\ eV} \lesssim m \lesssim 10^{-18}\mathrm {\ eV}, \end{aligned}$$

where the lower end corresponds to the crossover scale to DE, and the upper end is empirically determined as where the ALPs become indistinguishable from pressureless CDM. DM in this mass range has been dubbed “fuzzy cold dark matter”, or FCDM (Hu et al. 2000).

There may be a small model-dependent thermal population of ALPs, but the majority of the cosmological population will be cold and non-thermally produced. Production of cosmological ALPs proceeds by the vacuum realignment mechanism. When the Peccei–Quinn-like *U*(1) symmetry is spontaneously broken at the scale $f_a$ the ALP acquires a vacuum expectation value, the misalignment angle $\theta _i$, uncorrelated across different causal horizons. However, provided that inflation occurs after symmetry breaking, $f_a>H_I/2pi$, then the field is homogenized over our entire causal volume. This is the scenario we will consider, since large $f_a\gtrsim 10^{16}\mathrm {\ GeV}$ is required for ultra-light scalars to make up any significant fraction of the DM^20^.

Due to high occupation numbers ALPs can be modelled as a classical scalar field (see Sikivie and Yang 2009, and references therein), $\phi$, rolling in a potential, $V(\phi )$. For simplicity we will take $V(\phi )=m^2\phi ^2/2$, and we discuss other possibilities in Sect. II.11.2. The field evolves according to the Klein–Gordon (KG) equation, which for the homogeneous component, $\phi _0(t)$, is:

II.11.2 $$\begin{aligned} \ddot{\phi _0}+3 H \dot{\phi _0}+m^2\phi _0 = 0, \end{aligned}$$

where overdots denote derivatives with respect to physical time. The energy density in axions is given by $\rho _a=\frac{1}{2}(\dot{\phi }^2+m^2\phi ^2)$. When $H\gtrsim m$ the axion field is frozen at its initial displacement, the energy density remains constant, $w=-\,1$, and the density perturbations do not cluster. When $H\lesssim m$ the field begins to oscillate about the potential minimum, and the time averaged energy density on time scales $\gg 1/m$ has equation of state $w=0$. The horizon size when oscillations began imprints a characteristic scale on axion density perturbations. In addition, a WKB approximation leads to a non-zero time averaged sound speed (in, e.g., the synchronous or Newtonian gauge) for the density perturbations, even in the $w=0$ phase (e.g., Hu et al. 2000):

II.11.3 $$\begin{aligned} c_s^2\equiv \frac{\delta P}{\delta \rho }=\frac{k^2/4m^2a^2}{1+k^2/4m^2a^2}. \end{aligned}$$

This sound speed leads to a Jeans scale for axion density perturbations, suppressing structure formation. The Jeans scale is given by

II.11.4 $$\begin{aligned} k_J\approx a^{1/4}\left( \frac{\varOmega _mh^2}{0.12}\right) ^{1/4}\left( \frac{0.7}{h}\right) \left( \frac{\rho }{\bar{\rho }}\right) ^{1/4}\left( \frac{m}{10^{-22}\mathrm {\ eV}}\right) ^{1/2}\times 10^2\, h\mathrm {Mpc}^{-1}. \end{aligned}$$

The Jeans scale can also be equated to the de-Broglie wavelength, e.g., in a halo with virial velocity *v*, $r_J \sim \lambda _{\mathrm dB} \sim 1/mv$ (Hu et al. 2000).

In the range $m\sim 10^{-22}\mathrm {\ eV}$, due to the existence of the Jeans scale, FCDM may play a role in the resolution of small-scale problems of CDM. Marsh and Silk (2014) showed that in this regard it may out-perform WDM and avoid the so-called “Catch 22” of WDM whereby one cannot form dwarf galaxies and simultaneously give large core radii to their density profiles. In the mass range $m\sim 10^{-32}\mathrm {\ eV}$ ALPs/FCDM have affects on structure not dissimilar from massive neutrinos (Amendola and Barbieri 2006; Marsh et al. 2012), however there is no degeneracy when the CMB is used in conjunction with large scale structure, due to the different effects on the expansion rate during the radiation era.

The large phase space density of ultralight scalar fields may cause them to form Bose–Einstein condensates (see Sikivie and Yang 2009, and references therein). This could lead to many interesting, and potentially observable phenomena, such as formation of vortices in the condensate, which may effect halo mass profiles (Silverman and Mallett 2002; Kain and Ling 2010), and black hole super radiance (Arvanitaki et al. 2010; Arvanitaki and Dubovsky 2011; Rosa 2010). Sikivie has argued (Sikivie 2010) that axion dark matter fits the observed caustics in dark matter profiles of galaxies, which cannot be explained by ordinary dust CDM.

Regardless of the specifics of the model, Tegmark et al. (2006) and others have argued that on general statistical grounds we should expect a scenario where ALPs make up an order one fraction of the CDM, alongside the standard WIMP candidate of the lightest supersymmetric particle. However, it must be noted that there are objections when we consider a population of light fields in the context of inflation (Mack 2011; Mack and Steinhardt 2011). The problem with these objections is that they make some assumptions about what we mean by “fine tuning” of fundamental physical theories, which is also related to the problem of finding a measure on the landscape of string theory and inflation models (see, e.g., Linde and Noorbala 2010), the so-called “Goldilocks problem”.

#### Particle physics and string theory models

Axions and ALPs arise generically in string theory (Svrcek and Witten 2006). They are similar to the well known QCD axion (Peccei and Quinn 1977; Weinberg 1978; Wilczek 1978; ’t Hooft 1976b, a; Dine 1981; Preskill et al. 1983; Steinhardt and Turner 1983; Turner et al. 1983; Abbott and Sikivie 1983; Dine and Fischler 1983; Turner 1986; Visinelli and Gondolo 2009), and their cosmology has been extensively studied (see, for example, Banks and Dine 1997). Axions arise from the spontaneous breaking of a global *U*(1) symmetry at some high scale, $f_a$, where $f_a$ is known as the axions decay constant. Axion masses are protected by a shift symmetry to all orders in perturbation theory, but they acquire a potential due to non-perturbative effects which preserves a discrete shift symmetry, making the axion field periodic. The canonically normalised field is $\phi =f_a\theta$ and the potential is:

II.11.5 $$\begin{aligned} V(\phi )=\varLambda _a^4 U(\theta ), \end{aligned}$$

where $U(\theta )$ can be any periodic function of $\theta$, but is commonly taken to be $U(\theta )=(1-\cos \theta )$. The energy scale of non-perturbative physics is $\varLambda _a$. For the QCD axion, QCD instantons provide the potential and we have $\varLambda _a=\varLambda _\mathrm{QCD}\sim 200\text { MeV}$. Expanding the potential around the minimum at $\theta =0$, the mass is given by:

II.11.6 $$\begin{aligned} m=\frac{\varLambda ^2}{f_a}. \end{aligned}$$

Since in the expansion of the potential all self-couplings will be suppressed by the high scale $f_a$, and the specific form of *U* is model-dependent, this justifies the assumption above to consider only the quadratic mass term as relevant in the cosmological setting. In addition non-periodic potentials have been constructed in string theory via so-called ‘axion monodromy’.

In a string theory setting, the scale $\varLambda _a$ is unknown, but is expected to scale as $\varLambda _a=\mu e^{-S}$, where $\mu$ is some hard scale possibly related to SUSY breaking, and the action, *S* scales linearly with the value of some modulus field that fixes the size of a closed cycle in the compact space. The number of axions in string theory models is fixed by the topology of the compact space. Since many known Calabi–Yau manifolds have large Hodge numbers in the range 10’s–100’s or more, the number of axions is expected to be large. The observation of that there are many fields, with masses both protected by a shift symmetry and exponentially sensitive to the details of moduli stabilisation leads naturally to the conclusion that the existence of ultra-light axions is a plausibly general feature of string theory. The resulting scenario has been called the ‘String Axiverse’ (Arvanitaki et al. 2010).

In a field theory model, due to the shift symmetry, additional (to the QCD axion) light axions can also be introduced in a natural way. The non-perturbative effects leading to the potential and a scale $\varLambda _a\ne \varLambda _{\mathrm{QCD}}$ require the introduction, e.g., of a new strongly coupled sector.

#### Constraints from large scale structure

Cosmological constraints on ALPs can be obtained across the range of Eq. II.11.1. The combined CMB-large scale structure likelihood analysis of Amendola and Barbieri (2006) has shown that ultra-light fields with mass around $10^{-32}\textendash 10^{-24}\mathrm {\ eV}$ might account for up to 10% of the dark matter abundance. Outside of this range, the abundance can be larger.^21^ Euclid will be able to place tighter constraints across this entire range, and extend the mass range covered.

Ultra-light fields with $m\lesssim 10^{-29}\mathrm {\ eV}$ are similar in many ways to massive neutrinos (Amendola and Barbieri 2006), the major difference being that their non-thermal production breaks the link between the scale of suppression, $k_m$, and the fraction of dark matter, $f_{ax}$, through the dependence of $f_{ax}$ on the initial field value $\phi _i$. Therefore an accurate measurement of the matter power spectrum in the low-*k* region where massive neutrinos corresponding to the WMAP limits on $\varOmega _\nu$ are expected to suppress structure will determine whether the expected relationship between $\varOmega _\nu$ and $k_m$ holds. These measurements will limit the abundance of ultra-light fields that begin oscillations in the matter-dominated era.

Another powerful test of the possible abundance of ultralight fields beginning oscillations in the matter era will be an accurate measure of the position of the turn over in the matter power spectrum, since this gives a handle on the species present at equality. Ultra-light fields with masses in the regime such that they begin oscillations in the radiation-dominated era may suppress structure at scales where the BAO are relevant, and thus distort them. Improving the accuracy of the BAO measurement would place severe limits on ultralight fields in this mass regime.

There has been considerable progress in the last few years, placing constraints on ultra-light scalar fields with cosmological data, specifically using latest CMB measurements. In Hložek et al. (2015), the authors found that in the mass range $10^{-32}~\mathrm{eV}\le m_{a}\le 10^{-25.5}~\mathrm{eV}$, the axion relic-density $\varOmega _{a}$ (relative to the total dark-matter relic density $\varOmega _{d}$) obeys $\varOmega _{a}/\varOmega _{d} \le 0.05$ and $\varOmega _{a}h^{2}\le 0.006$ at $95\%$-confidence. For $m_{a}\sim 10^{-24}~\mathrm{eV}$, the ultra slight scale fields are indistinguishable from dark matter while for $m_{a}< 10^{-32}~\mathrm{eV}$,they can make up a large fraction of the dark energy. More recently, in Hložek et al. (2017b), the authors have a identified a “window of co-existence” of $10^{-25}~\mathrm{eV}\le m_{a}\le 10^{-24}~\mathrm{eV}$ where the data allow a $\sim \,10\%$ contribution of ultra-light fields to the dark matter budget while at the same time leading to a $\sim \,1\%$ contribution of isocurvature and tensors modes to the CMB powe spectrum.

**Fig. 50:**
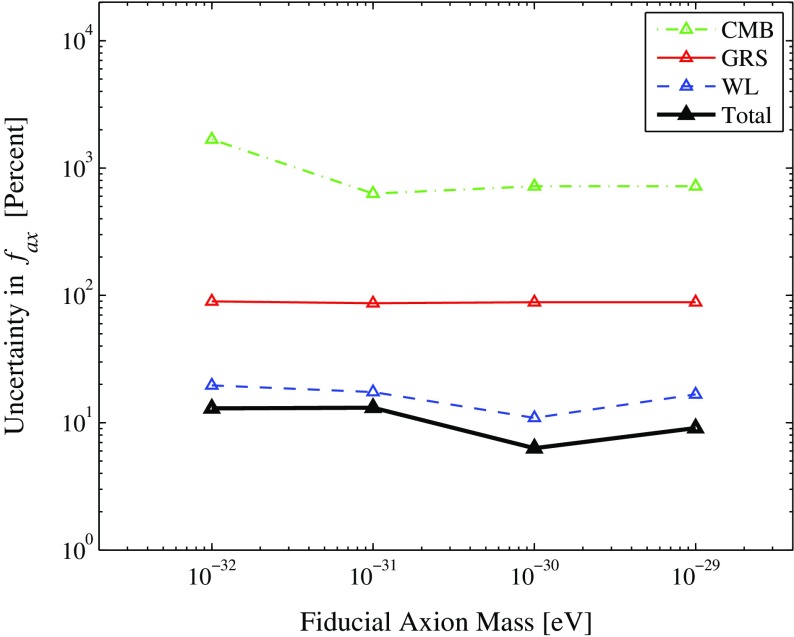
Marginalized uncertainty in $f_{ax}$ for CMB (green), a galaxy redshift survey (red), weak lensing (blue) and the total (black) evaluated for four different fiducial axion masses, for the cosmology $\varLambda \hbox {CDM}+f_{ax}+\nu$. Image reproduced by permission from Marsh et al. (2012), copyright by APS

Future surveys will greatly improve constraints on ultra-light scalar fields. For a start, Stage IV CMB experiments, as shown in Hložek et al. (2017a) while improve constraints on the ultra-light scalar field energy density by a factor of 10 in the mass range $10^{-32}~\mathrm{eV}\le m_{a}\le 10^{-23}~\mathrm{eV}$ and will, furthermore be able to break the degeneracies between the scalar fields and massive neutrinos. As shown in Marsh et al. (2012), these constraints will be matched and improved next generation galaxy surveys alone (such as Euclid) it should be possible to unambiguously detect a fraction of dark matter in axions with $10^{-33}\mathrm {\ eV}<m< 10^{-29}\mathrm {\ eV}$ of the order of 1% of the total. Furthermore, the authors in Marsh et al. (2012) demonstrated that the tightest constraints on the axion fraction $f_{ax}$ come from a Euclid-like weak lensing survey; when combined with a galaxy redshift survey, constraining $f_{ax}$ to 0.1% should be possible, see Fig. 50. The strength of the weak lensing constraint depends on the photometric redshift measurement, i.e., on tomography. Therefore, lensing tomography will allow Euclid—through the measurement of the growth rate—to resolve the redshift evolution of the axion suppression of small scale convergence power. Further details can be found in Marsh et al. (2012).

**Fig. 51:**
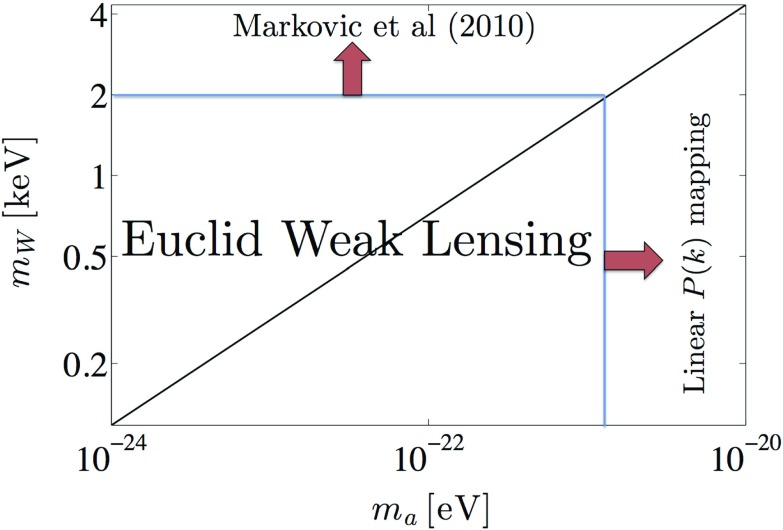
Mapping between axion and WDM mass that suppress power by a factor of two at the same scale. This can be used to approximately the axion mass constraint possible with Euclid on based on WDM forecasts

At the heavier end with $m> 10^{-24}\mathrm {\ eV}$ axions affect structure formation on scales comparable to WDM with $m_W\gtrsim 0.2\mathrm {\ keV}$ Marsh and Silk (2014). By matching the scale at which the transfer function is suppressed by factor of 2, one can make a map between axion and WDM masses. This map, adapted from Marsh and Silk (2014), is shown in Fig. 51. Such a map serves as a guide to the constraining power of Euclid on axions based on WDM forecasts and constraints. However it should be noted that the Jeans scale is dynamical for axions, leading to scale dependent growth, and different non-linear behaviour to WDM, so dedicated studies are needed. For example, constraining $m_\mathrm{WDM}>2\mathrm {\ keV}$ (Markovič et al. 2011) (and Sect. II.6 of this review) can constrain axions to $m\gtrsim 10^{-21}\mathrm {\ eV}$ as the main component of the DM. Reaching this level is significant as it approaches the exclusion limits from black hole super radiance (Arvanitaki and Dubovsky 2011).

The expected suppression of structure caused by ultralight fields should be properly taken into account in *N*-body simulations. The nonlinear regime of *P*(*k*) needs to be explored further both analytically and numerically for cosmologies containing ultra-light fields, especially to constrain those fields which are heavy enough such that $k_m$ occurs around the scale where nonlinearities become significant, i.e., those that begin oscillation deep inside the radiation-dominated regime and are naively degenerate with WDM. For lighter fields the effects in the nonlinear regime should be well-modelled by using the linear *P*(*k*) for *N*-body input, and shifting the other variables such as $\varOmega _c$ accordingly.

In conclusion, with proper numerical modelling, it should be possible for Euclid to put powerful, percent-level constraints on an ultra-light scalar field contribution to the DM over 12–13 orders of magnitude in mass in the range $10^{-33}\mathrm {\ eV}\lesssim m\lesssim 10^{-20}\mathrm {\ eV}$. Euclid will also be able to place complementary constraints on ALPs through their generic coupling to photons. This ALP-photon coupling allows photons to decay into unobserved ALPs, thus affecting infered luminosity distances to SN sources. Euclid distance measurements will place strong constraints on this effect, at an unprecedented level. See Sect. IV.2 for details, including forecasts.

### Dark-matter surrogates in theories of modified gravity

#### Extra fields in modified gravity

The idea that the dark universe may be a signal of modified gravity has led to the development of a plethora of theories. From polynomials in curvature invariants, preferred reference frames, UV and IR modifications and extra dimensions, all lead to significant modifications to the gravitational sector. A universal feature that seems to emerge in such theories is the existence of fields that may serve as a proxy to dark matter. This should not be unexpected. On a case by case basis, one can see that modifications to gravity generically lead to extra degrees of freedom.

For example, polynomials in curvature invariants lead to higher-derivative theories which inevitably imply extra (often unstable) solutions that can play the role of dark matter. This can be made patently obvious when mapping such theories onto the Einstein frame with an addition scalar field (scalar–tensor theories). Einstein-Aether theories (Zlosnik et al. 2008) explicitly introduce an extra time-like vector field. The time-like constraint locks the background, leading to modifications to the background expansion; perturbations in the vector field can, under certain conditions, lead to growth of structure, mimicking the effect of pressureless dark matter. The vector field plays the same role in TeVeS (Bekenstein 2004), where two extra fields are introduced to modify the gravitational dynamics. And the same effects come into play in bigravity models (Bañados et al. 2009) where two metrics are explicitly introduced—the scalar modes of the second metric can play the role of dark matter.

In what follows we briefly focus on three of the above cases where extra gravitational degrees of freedom play the role of dark matter: Einstein-Aether models, TeVeS models and bigravity models. We will look at the Einstein-Aether model more carefully and then briefly discuss the other two cases.

#### Vector dark matter in Einstein-Aether models

As we have seen in a previous section, Einstein-Aether models introduce a time-like vector field $A^{a}$ into gravitational dynamics. The four vector $A^{a}$ can be expanded as $A^{\mu }=(1+\epsilon X,\epsilon \partial ^{j}Z)= (1+\epsilon X,\frac{\epsilon }{a^{2}}\partial _{j}Z)$ (Zlosnik et al. 2008). In Fourier space we have $A^{\mu }=(1-\epsilon \varPsi ,i\frac{\epsilon }{a} k_{j}V)$, where, for computational convenience, we have defined $V\equiv Z/a$ and have used the fact that the constraint fixes $X=-\,\varPsi$.

The evolution equation for the perturbation in the vector field becomes (where primes denote derivatives with respect to conformal time)

II.12.1 $$\begin{aligned} 0&= c_{1}[V''+k^{2}V+2{{\mathcal {H}}}V'+2\mathcal{H}^{2}V+\varPsi '+\varPhi '+2{{\mathcal {H}}}\varPsi ] \nonumber \\&\quad +c_2[k^{2}V+6{{\mathcal {H}}}^{2}V-3\frac{a''}{a}V+3\varPhi '+3{{\mathcal {H}}}\varPsi ] \nonumber \\&\quad +c_3[k^{2}V+2{{\mathcal {H}}}^{2}V-\frac{a''}{a}V+\varPhi '+{{\mathcal {H}}}\varPsi ] \nonumber \\&\quad +\frac{F_{KK}}{F_{K}}[-K^{\epsilon }\alpha \mathcal{H}-K^{0'}(-c_{1}(V'+\varPsi )+3c_{2}{{\mathcal {H}}} V+c_{3}{{\mathcal {H}}} V)]. \end{aligned}$$

The perturbation in the vector field is sourced by the two gravitational potentials $\varPhi$ and $\varPsi$ and will in turn source them through Einstein’s equations. The Poisson equation takes the form

II.12.2 $$\begin{aligned} k^{2}\varPhi&=-\frac{1}{2}F_{K}c_{1}k^{2}[V'+\varPsi +(3+2\tilde{c}_{3})\mathcal{H}V] \nonumber \\&\qquad -4\pi Ga^{2}\sum _{a}\left( \bar{\rho }_{a}\delta _{a}+3(\bar{\rho }_{a}+\bar{P}_{a})\mathcal{H}\frac{\theta _{a}}{k^{2}}\right) . \end{aligned}$$

To understand why the vector field can play the role of dark matter it is instructive to study the effect of the vector field during matter domination. It should give us a sense of how in the generalized Einstein-Aether case, the growth of structure is affected. Let us consider the simplest case in which the the dominant remaining contribution to the energy density is baryonic, treated as a pressureless perfect fluid with energy–momentum tensor $\mathbf {T}$ and let us introduce the variable $V'\equiv E$. For ease of illustration we will initially consider only the case where *V* is described by a growing monomial, i.e. $V=V_{0}(\eta /\eta _0)^p$. During the matter era we have

II.12.3 $$\begin{aligned} a^{2}\delta T^{0}_{\phantom {0}0}&\simeq -l_{E}\xi (k)k^{2}\eta ^{5+p-6n} \end{aligned}$$

II.12.4 $$\begin{aligned} k^{2}(\varPsi -\varPhi )&\simeq -l_{S}\xi (k)k^{2}\eta ^{5+p-6n} \end{aligned}$$

with $$l_{E} \equiv -(c_{1}(2+p)n+2\alpha (1-2n)n)$$, $$l_{S} \equiv -(c_{1}+c_{3})n(6n-p-10)$$, and

II.12.5 $$\begin{aligned} \xi (k) \sim \gamma V_{0}(k)\eta _0^{-p}k_\mathrm {hub}^{6-6n}\left[ 3\alpha \varOmega _{m}\left( \frac{H_{0}}{M}\right) ^{2}\right] ^{n-1} , \end{aligned}$$

where $k_\mathrm {hub}\equiv 1/\eta _\mathrm {today}$. Hence, the vector field affects our evolution equations for the matter and metric perturbations only through its contribution to the energy density and its anisotropic stress. On large scales, $k \eta \ll 1$, and assuming adiabatic initial conditions for the fields $\delta$, $\varPhi$ and $\theta$, this leads to

II.12.6 $$\begin{aligned} \delta = C_{1}(k)+\frac{6l_{S}\xi (k)}{(10+p-6n)}\eta ^{5+p-6n}, \end{aligned}$$

where $C_{1}$ is a constant of integration and we have omitted the decaying mode. Therefore, even before horizon crossing, the anisotropic stress term due to the vector field can influence the time evolution of the baryon density contrast.

On small scales ($k\eta \gg 1$), we find

II.12.7 $$\begin{aligned} \delta (k,\eta ) = C_{2}(k)\eta ^{2} +\frac{(\frac{1}{2}l_{E}+l_{S})}{(5+p-6n)(10+p-6n)}\xi (k)(k\eta )^{2}\eta ^{5+p-6n} , \end{aligned}$$

where $C_{2}(k)$ is a constant of integration. Hence, for sub-horizon modes, the influence of the vector field on the evolution of $\delta$ is a combination of the effect of the energy density and anisotropic stress contributions though both, in this limit, result in the same contributions to the scale dependence and time evolution of the density contrast. The net effect is that, for particular choices of parameters in the action, the perturbations in the vector field can enhance the growth of the baryon density contrast, very much along the lines of dark matter in the dark matter dominated scenario.

#### Scalar and tensors in TeVeS

We have already come across the effect of the extra fields of TeVeS. Recall that, in TeVeS, as well as a metric (tensor) field, there is a time-like vector field and a scalar field both of which map the two frames on to each other. While at the background level the extra fields contribute to modifying the overall dynamics, they do not contribute significantly to the overall energy density. This is not so at the perturbative level. The field equations for the scalar modes of all three fields can be found in the conformal Newtonian gauge in Skordis et al. (2006). While the perturbations in the scalar field will have a negligible effect, the space-like perturbation in the vector field has an intriguing property: it leads to growth. Dodelson and Liguori (2006) have shown that the growing vector field feeds into the Einstein equations and gives rise to a growing mode in the gravitational potentials and in the baryon density. Thus, baryons will be aided by the vector field leading to an effect akin to that of pressureless dark matter. The effect is very much akin to that of the vector field in Einstein-Aether models—in fact it is possible to map TeVeS models onto a specific subclass of Einstein-Aether models. Hence the discussion above for Einstein-Aether scenarios can be used in the case of TeVeS.

#### Tensor dark matter in models of bigravity

In bigravity theories (Bañados et al. 2009), one considers two metrics: a dynamical metric $g_{\mu \nu }$ and a background metric, ${{\tilde{g}}}_{\alpha \beta }$. As in TeVeS, the dynamical metric is used to construct the energy–momentum tensor of the non-gravitational fields and is what is used to define the geodesic equations of test particles. The equations that define its evolution are usually not the Einstein field equations but may be defined in terms of the background metric.

Typically ${{\tilde{g}}}_{\alpha \beta }$ is dynamical, with a corresponding term in the gravitational action. It then becomes necessary to link ${{\tilde{g}}}_{\alpha \beta }$ to $g_{\mu \nu }$ with the background metric determining the field equations of the dynamical metric through a set of interlinked field equations. In bigravity models both metrics are used to build the Einstein–Hilbert action even though only one of them couples to the matter content. A complete action is of the form

II.12.8 $$\begin{aligned} S=\frac{1}{16 \pi G}\int \mathrm {d}^4 x\left[ {\sqrt{-g}}(R-2\varLambda )+{\sqrt{-\tilde{g}}} \left( {\tilde{R}}-2{\tilde{\varLambda }}\right) -\sqrt{-{{\tilde{g}}}}\frac{1}{\ell ^2}({\tilde{g}}^{-1})^{\alpha \beta }g_{\alpha \beta }\right] ,\nonumber \\ \end{aligned}$$

where $\varLambda$ and ${{\tilde{\varLambda }}}$ are two cosmological constant terms and $\ell ^2$ defines the strength of the linking term between the two actions. The cosmological evolution of perturbations in these theories has been worked out in some detail. It turns out that perturbations in the auxiliary field can be rewritten in the form of a generalized dark matter fluid (Hu et al. 1998) with fluid density, momentum, pressure and shear that obey evolution equations which are tied to the background evolution. As a result, it is possible to work out cosmological observables such as perturbations in the CMB and large scale structure. If we restrict ourselves to a regime in which ${{\tilde{\rho }}}$ simply behaves as dark matter, then the best-fit bimetric model will be entirely indistinguishable from the standard CDM scenario.

### Outlook

Dark matter dominates the matter content of the universe, and only through astrophysical and cosmological observations can the nature of dark matter on large scales be determined. In this review, we have discussed a number of observational techniques available to Euclid: dark matter mapping, complementarity with other astronomical observations (e.g., X-ray and CMB experiments); cluster and galaxy scale dark matter halo mapping; and power spectrum analyses. The techniques described will allow Euclid to constrain a variety of dark matter candidates and their microphysical properties. We have discussed Warm Dark Matter scenarios, axion-like dark matter, scalar field dark matter models (as well as the possible interactions between dark energy and scattering with ordinary matter) and massive neutrinos (the only known component of dark matter).

Here, we briefly list the main dark matter constraints so far forecasted for Euclid:

- The weak lensing power spectrum from Euclid will be able to constrain warm dark matter particle mass to about $m_\mathrm {WDM}>2\mathrm {\ keV}$ (Markovič et al. 2011);
- The galaxy power spectrum, with priors from Planck (primary CMB only), will yield an error on the sum of neutrino masses $\varSigma$ of 0.03–0.06 eV, depending on the fiducial value of $\varSigma$, and assuming GR and non-interacting dark energy (see Table 19; Carbone et al. 2011b);
- Euclid’s weak lensing should also yield an error on $\varSigma$ of 0.05 eV (Kitching et al. 2008a);
- Jiménez et al. (2010) have shown that weak gravitational lensing from Euclid data will be able to determine neutrino hierarchy (if $\varSigma <0.13$);
- The forecasted errors on the effective number of neutrino species $N_{\nu ,\mathrm {eff}}$ for Euclid (with a Planck prior) are $\pm \,0.1$ (for weak lensing Kitching et al. 2008a) and $\pm \,0.086$ (for galaxy clustering Carbone et al. 2011b);
- The sound speed of unified dark energy-dark matter can be constrained with errors $\sim \,10^{-5}$ by using 3D weak lensing (Camera et al. 2011c);
- Recently, Marsh et al. (2012) showed that with current and next generation galaxy surveys alone it should be possible to unambiguously detect a fraction of dark matter in axions of the order of 1% of the total.

We envisage a number of future scenarios, all of which give Euclid an imperative to confirm or identify the nature of dark matter. In the event that a dark matter candidate is discovered in direct detection experiments or an accelerator (e.g., LHC) a primary goal for Euclid will be to confirm, or refute, the existence of this particle on large scales. In the event that no discovery is made directly, then astronomical observations will remain our only way to determine the nature of dark matter.

## Part III Initial conditions

### Introduction

The exact origin of the primordial perturbations that seeded the formation of the large-scale structure in the universe is still unknown. Our current understanding of the initial conditions is based on inflation, a phase of accelerated expansion preceding the standard evolution of the universe (Guth 1981; Starobinsky 1979, 1982; Sato 1981). In particular, inflation explains why the universe is so precisely flat, homogeneous and isotropic. During this phase, scales much smaller than the Hubble radius are inflated to super-horizon sizes, so that regions appearing today as causally disconnected were in fact very close in the past. This mechanism is also at the origin of the cosmic large-scale structure. Vacuum quantum fluctuations of any light field present during inflation are amplified by the accelerated expansion and *freeze-out* on super-Hubble scales acquiring a quasi-scale invariant spectrum (Mukhanov and Chibisov 1981; Hawking 1982; Starobinsky 1982; Guth and Pi 1982; Bardeen et al. 1983).

From the early development of inflation, the simplest proposal based on a weakly-coupled single scalar field slow-rolling along its potential (Linde 1982; Albrecht and Steinhardt 1982) has gained strength and many models have been built based on this picture (see, e.g., Linde 2008 for a review). The simplest inflationary models based on a weakly-coupled single scalar field are compatible with Planck 2015 cosmological results. The geometry is compatible with being flat ($\varOmega _K = -\,0.005^{+0.016}_{-0.017}$ at 95% CL by combining Planck 2015 temperature and lensing information, Planck Collaboration 2014a, b). The most recent Planck 2015 determination of the scalar spectral index is $n_\mathrm {s}=0.968 \pm 0.06$ at 68% CL, disfavouring exact scale invariance at 6$\sigma$. There is no statistical evidence of a running of the spectral index, i.e. $\mathrm {d} n_{\mathrm s}/\mathrm {d}\ln k = -\,0.003 \pm 0.007$ at 68%CL. Although many inflationary potentials are now excluded by Planck 2015 data (Planck Collaboration 2014c), the simplest inflationary predictions have been extremely successful in passing many observational tests: it predicts perfectly adiabatic and almost Gaussian fluctuations with a quasi scale-invariant spectrum and a small amount of gravitational waves.

While current data have ruled out some classes of inflationary models, the next qualitative step forward is investigating the physics responsible for inflation: we still lack a complete understanding of the high energy physics describing it. In fact, most likely the physics of inflation is far out of reach of terrestrial experiments, many orders of magnitude larger than the center-of-mass energy at the Large Hadron Collider (LHC). Thus, cosmological tests of inflation offer a unique opportunity to learn about ultra-high energy physics. We can do this by targeting observations which directly probe the dynamics of inflation. One route is to accurately measure the shape of the primordial power spectrum of scalar perturbations produced during the phase of accelerated expansion, which is directly related to the shape of the inflaton potential, and to constrain the amplitude of the corresponding stochastic gravitational-wave background, which is related instead to the energy-scale of inflation. Planck, in combination with the BICEP2/Keck Array data, has constrained the tensor-to-scalar ratio to $r_{0.05} < 0.08$ at 95%CL (BICEP2/Keck Array and PlanckCollaborations 2015). With the most recent addition of the Keck Array 95 GHz channel, the constraint has been further tightened to $r_{0.05} < 0.07$ at 95%CL (BICEP2 Collaboration 2016).

A complementary approach is offered by constraining—or exploring—how much the distribution of primordial density perturbations departs from Gaussian statistics and purely adiabatic fluctuations. Non-Gaussianity is a very sensitive probe of self-couplings and interactions between the fields generating the primordial perturbations, whereas the presence of isocurvature modes can teach us about the number of fields present during inflation and their role in reheating and generating the matter in the universe. At present, Planck establish that the statistics of primordial perturbations is compatible with a Gaussian one (Planck Collaboration 2016a, b) and that the preference of isocurvature initial conditions is not statistically significant. Nevertheless, future large-scale structure surveys like Euclid can probe these features with an unprecedented accuracy, thus providing a way to test aspects of inflationary physics that are not easily accessible otherwise.

Non-minimal scenarios or proposals even radically different from single-field inflation are still compatible with the data. In order to learn something about the physics of the early universe we need to rule out or confirm the conventional slow-roll scenario and possibly discriminate between non-conventional models. Non-Gaussianities and isocurvature perturbations currently represent the best tools that we have to accomplish this task. Any deviation from the conventional Gaussian and adiabatic initial perturbations would represent important breakthroughs in our understanding of the early universe. In this section we are going to review what we can learn by constraining the initial conditions with a large-scale structure survey like Euclid.

### Constraining inflation

The spectrum of cosmological perturbations represents an important source of information on the early universe. During inflation scalar (compressional) and tensor (purely gravitational) fluctuations are produced. The shape and the amplitude of the power spectrum of scalar fluctuations can be related to the dynamics of the inflationary phase, providing a window on the inflaton potential. Inflation generically predicts a deviation from a purely scale-invariant spectrum. Together with future CMB experiments such as Planck, Euclid will improve our constraints on the scalar spectral index and its running, helping to pin down the model of inflation.

#### Primordial perturbations from inflation

It is convenient to describe primordial perturbations using the curvature perturbation on uniform density hypersurfaces $\zeta$ introduced in Bardeen et al. (1983). An important property of this quantity is that for adiabatic perturbations—i.e., in absence of isocurvature perturbations, discussed in Sect. III.5—it remains constant on super-Hubble scales, allowing us to connect the early inflationary phase to the late-time universe observations, regardless of the details of reheating. In a gauge where the energy density of the inflaton vanishes, we can define $\zeta$ from the spatial part of the metric (assuming a flat FRW universe), as (Salopek and Bond 1990; Maldacena 2003)

III.2.1 $$\begin{aligned} g_{ij} = a^2(t) \exp \left( 2 \zeta \right) \delta _{ij}. \end{aligned}$$

This definition, where $\zeta$ enters the metric in the exponential form, has the advantage that it is valid also beyond linear order and can be consistently used when discussing non-Gaussian fluctuations, such as in Sect. III.3.

The power spectrum of primordial perturbations is given by

III.2.2 $$\begin{aligned} \langle \zeta _{\mathbf {k}} \zeta _{\mathbf {k}'} \rangle = (2 \pi )^3 \delta \left( \mathbf {k}+\mathbf {k}'\right) P_{\zeta }(k), \end{aligned}$$

where $\langle \cdots \rangle$ denotes the average over an ensemble of realizations. It is useful to define a dimensionless spectrum as ${{\mathcal {P}}}_s(k) \equiv \frac{k^3}{2\pi ^2} P_\zeta (k)\;$, where the index *s* stands for scalar, to distinguish it from the spectrum of tensor perturbations, defined below. The deviation from scale-invariance of the scalar spectrum is characterized by the spectral index $n_s$, defined by (see, e.g., Liddle and Lyth 2000)

III.2.3 $$\begin{aligned} n_s \equiv 1 + \frac{d \ln {{\mathcal {P}}}_s}{d \ln k} , \end{aligned}$$

where $n_s=1$ denotes a purely scale-invariant spectrum. We also define the running of the spectral index $\alpha _s$ as

III.2.4 $$\begin{aligned} \alpha _s \equiv \frac{d n_s}{d \ln k}. \end{aligned}$$

These quantities are taken at a particular pivot scale. For our analysis we chose it to be $k_* \equiv 0.05\mathrm {\ Mpc}^{-1}$. Thus, with these definitions the power spectrum can be written as

III.2.5 $$\begin{aligned} P_\zeta (k) = \frac{2 \pi ^2}{k^3} A_s(k_*) (k/k_*)^{n_s(k_*) -1 + \frac{1}{2} \alpha _s(k_*) \ln (k/k_*) }, \end{aligned}$$

where $A_s$ is the normalization parameterising the amplitude of the fluctuations.

During inflation tensor modes are also generated. They are described by the gauge invariant metric perturbation $h_{ij}$, defined from the spatial part of the metric as

III.2.6 $$\begin{aligned} g_{ij} = a^2(t) \left( \delta _{ij} + h_{ij} \right) , \quad h^j_{i,j} = 0 = h_{i}^i. \end{aligned}$$

Each mode has 2 polarizations, $h_+$ and $h_\times$, each with power spectrum given by

III.2.7 $$\begin{aligned} \langle h_{\mathbf {k}} h_{\mathbf {k}'} \rangle = (2 \pi )^3 \delta \left( \mathbf {k}+\mathbf {k}'\right) P_h(k). \end{aligned}$$

Defining the dimensionless power spectrum of tensor fluctuations as ${{\mathcal {P}}}_t(k) \equiv 2 \frac{k^3}{2\pi ^2} P_h (k)\;$, where the factor of 2 comes from the two polarizations, it is convenient to define the ratio of tensor to scalar fluctuations as

III.2.8 $$\begin{aligned} r\equiv {{{\mathcal {P}}}_t (k_*)}/{{{\mathcal {P}}}_s (k_*)}. \end{aligned}$$

The form of the power spectrum given in Eq. (III.2.5) approximates very well power spectra of perturbations generated by slow-roll models. In particular, the spectrum of scalar fluctuations is given in terms of the Hubble rate *H* and the first slow-roll parameter $\epsilon \equiv - \dot{H}{/}H^2$, both evaluated at the time when the comoving scale *k* crosses the Hubble radius during inflation,

III.2.9 $$\begin{aligned} {{\mathcal {P}}}_s(k) = \left. \frac{1}{8 \pi ^2 \epsilon }\frac{H^2}{M_{\mathrm {Pl}}^2} \right| _{k=aH}. \end{aligned}$$

During slow-roll, $\epsilon$ is related to the first derivative of the inflaton potential $V(\phi )$, $\epsilon \approx \frac{M_{\mathrm {Pl}}^2}{2} \left( \frac{V'}{V} \right) ^2$, where the prime denotes differentiation with respect to $\phi$. As *H* and $\epsilon$ vary slowly during inflation, this spectrum is almost scale-invariant. Indeed, the scalar spectral index $n_s$ in Eq. (III.2.3) reads

III.2.10 $$\begin{aligned} n_s=1 - 6 \epsilon + 2 \eta _V, \end{aligned}$$

where the second slow-roll parameter $\eta _V \equiv M_{\mathrm {Pl}}^2 \frac{V''}{V}$ must be small for inflation to yield a sufficient number of *e*-foldings. The running of the spectral index defined in Eq. (III.2.4) is even smaller, being second-order in the slow-roll parameters. It is given by $\alpha _s = 16\epsilon \eta _V - 24 \epsilon ^2- 2\xi _V$ where we have introduced the third slow-roll parameter $\xi _V \equiv M_{\mathrm {Pl}}^4 \frac{V' V''' }{V^2}$.

The spectrum of tensor fluctuations is given by

III.2.11 $$\begin{aligned} {{\mathcal {P}}}_t(k) = \left. \frac{2}{\pi ^2 }\frac{H^2}{M_{\mathrm {Pl}}^2} \right| _{k=aH}, \end{aligned}$$

which shows that the ratio of tensor to scalar fluctuations in Eq. (III.2.8) is simply related to the first slow-roll parameter by $r = 16 \epsilon$.

As a fiducial model, in the next section we will consider chaotic inflation (Linde 1983), based on the quadratic inflaton potential $V = \frac{1}{2} m^2 \phi ^2$. In this case, the first two slow-roll parameters are both given in terms of the value of the inflaton field at Hubble crossing $\phi$ or, equivalently, in terms of number of *e*-folds from Hubble crossing to the end of inflation *N*, as $\epsilon =\eta _V = 2 M_{\mathrm {Pl}}^2/\phi ^2 = 1/2 N$, while $\xi _V =0$. This implies

III.2.12 $$\begin{aligned} n_s = 1 - 2/N_*, \quad r = 8/N_*, \quad \alpha _s = -2/N_*^2 , \end{aligned}$$

where the star denotes Hubble crossing of the pivot scale $k_*$. Choosing $N_*=62.5$, this yields $n_s= 0.968$, $r=0.128$ and $\alpha _s =0$ as our fiducial model.

#### Forecast constraints on the power spectrum

We will now study how much Euclid will help in improving the already very tight constraints on the power spectrum given by the Planck satellite. Let us start discussing the forecast for Planck. We assume 2.5 years (5 sky surveys) of multiple CMB channel data, with instrument characteristics for the different channels listed in Table 20. We take the detector sensitivities and the values of the full width half maximum from the Planck “Blue Book” (Planck Science Team 2009). In this analysis we use three channels for Planck mock data and we assume that the other channels are used for foreground removal and thus do not provide cosmological information.

**Table 20:** Instrument specifics for the Planck satellite with 30 months of integration

Channel frequency (GHz)	70	100	143
Resolution (arcmin)	14	10	7.1
Sensitivity-intensity ($\mu K$)	8.8	4.7	4.1
Sensitivity-polarization ($\mu K$)	12.5	7.5	7.8

For a nearly full-sky CMB experiment (we use $f_{\mathrm {sky}}=0.75$), the likelihood ${{\mathcal {L}}}$ can be approximated by (Verde et al. 2006)

III.2.13 $$\begin{aligned} \begin{aligned} -2 \ln {{{\mathcal {L}}}}&= \sum _{\ell =\ell _{\min }}^{\ell _{\max }} (2\ell +1)f_{\mathrm {sky}}\, \left[ -3 + \frac{\hat{C}_\ell ^{BB}}{C_\ell ^{BB}} + \ln \left( \frac{C_\ell ^{BB}}{\hat{C}_\ell ^{BB}}\right) \right. \\&\quad \left. + \,\frac{\hat{C}_\ell ^{TT}C_\ell ^{EE} + \hat{C}_\ell ^{EE}C_\ell ^{TT} - 2\hat{C}_\ell ^{TE}C_\ell ^{TE}}{C_\ell ^{TT}C_\ell ^{EE}-\left( C_\ell ^{TE}\right) ^2} +\ln {\left( \frac{C_\ell ^{TT}C_\ell ^{EE}- \left( C_\ell ^{TE}\right) ^2}{\hat{C}_\ell ^{TT}\hat{C}_\ell ^{EE}- \left( \hat{C}_\ell ^{TE}\right) ^2}\right) }\right] , \end{aligned}\nonumber \\ \end{aligned}$$

where we assume $l_{\min }=3$ and $l_{\max }=2500$. Here, $C_\ell$ is the sum of the model-dependent theoretical power spectrum $C_\ell ^{\mathrm {theory}}$ and of the noise spectrum $N_\ell$, which we assume perfectly known. The mock data $\hat{C}_\ell$ is $C_\ell$ for the fiducial model, with $C_\ell ^{\mathrm {theory}}$ calculated using the publicly available code camb (Lewis et al. 2000b) and $N_\ell$ calculated assuming a Gaussian beam. We use the model described in Verde et al. (2006) and Baumann et al. (2009) to propagate the effect of polarization foreground residuals into the estimated uncertainties on the cosmological parameters. For simplicity, in our simulation we consider only the dominating components in the frequency bands that we are using, i.e., the synchrotron and dust signals. The fraction of the residual power spectra are all assumed to be 5%.

Let us turn now to the Euclid forecast based on the spectroscopic redshift survey. We will model the galaxy power spectrum in redshift space as (Kaiser 1987; Peacock 1992; Peacock and Dodds 1994; see also discussion in Sect. I.7.3)

III.2.14 $$\begin{aligned} P_g(k,z,\mu ) = \left( b+ f_g \mu ^2\right) ^2 G^2(z)P_{\mathrm {matter}}(k;z=0) e^{-k^2\mu ^2\sigma _r^2}, \end{aligned}$$

where $\mu$ is the cosine of the angle between the wavenumber $\mathbf {k}$ and the line of sight, *G*(*z*) is the linear growth factor defined in Eq. (I.3.21), $f_g \equiv {d\ln G}/{d\ln a}$ is the linear growth rate [see Eq. (I.3.22)] and $P_{\mathrm {matter}}(k;z=0)$ is the matter power spectrum at redshift 0. The term $f_g \mu ^2$ comes for the redshift distortions due to the large-scale peculiar velocity field (Kaiser 1987), which is correlated with the matter density field. The factor $e^{-k^2\mu ^2\sigma _r^2}$ accounts for the radial smearing due to the redshift distortions that are uncorrelated with the large-scale structure. We consider two contributions. The first is due to the redshift uncertainty of the spectroscopic galaxy samples. Assuming a typical redshift uncertainty $\sigma _z=0.001(1+z)$, this turns into a contribution to $\sigma _r$ given by $\partial r/\partial z \, \sigma _z = H^{-1} \, \sigma _z$, where $r(z)=\int _0^z cdz'/H(z')$ is the comoving distance of a flat FRW universe and *H* is the Hubble parameter as a function of the redshift. The second contribution comes from the Doppler shift due to the virialized motion of galaxies within clusters, which typically have a pairwise velocity dispersion $v_p$ of the order of few hundred kilometers per second. This term can be parameterized as $\frac{v_p}{\sqrt{2}}H^{-1} (1+z)$ (Peacock and Dodds 1994). Taking the geometric mean of the two contributions, we obtain

III.2.15 $$\begin{aligned} \sigma _r^2 = \frac{(1+z)^2}{H^2} \left( 10^{-6} + {v_p^2}/{2} \right) , \end{aligned}$$

where the two velocities in the parenthesis contribute roughly the same. Practically neither the redshift measurement nor the virialized virialized motion of galaxies can be precisely quantified. In particular, the radial smearing due to peculiar velocity is not necessarily close to Gaussian. Thus, Eq. (III.2.14) should not be used for wavenumbers $k>\frac{H(z)}{v_p(1+z)}$, where the radial smearing effect is important.

On large scales the matter density field has, to a very good approximation, Gaussian statistics and uncorrelated Fourier modes. Under the assumption that the positions of observed galaxies are generated by a random Poissonian point process, the band-power uncertainty is given by (Tegmark et al. 1998; see also Eq. (I.7.26) in Sect. I.7.3)

III.2.16 $$\begin{aligned} \varDelta P_g = \left[ \frac{2 (2\pi )^3}{(2\pi k^2 \,dk \,d\mu ) \left( 4\pi r^2f_{\mathrm {sky}} \, dr\right) }\right] ^{1/2}\left( P_g+\frac{1}{\bar{n}}\right) . \end{aligned}$$

Here $f_{\mathrm {sky}}$ is the observed fraction of sky, *r* the comoving distance defined above, and $\bar{n}$ is the expected number density of galaxies that can be used.

Finally, we ignore the band-band correlations and write the likelihood as

III.2.17 $$\begin{aligned} -2 \ln {{\mathcal {L}}} = \sum _{k,\mu ,z\ \mathrm {bins}} \left( \frac{ P_g^{\mathrm {model}} - P_g^{\mathrm {fiducial}} }{\varDelta P_g^{\mathrm {fiducial}} }\right) ^2. \end{aligned}$$

To produce the mock data we use a fiducial $\varLambda$CDM model with $\varOmega _ch^2=0.1128$, $\varOmega _bh^2 = 0.022$, $h=0.72$, $\sigma _8 = 0.8$ and $\tau =0.09$, where $\tau$ is the reionization optical depth. As mentioned above, we take the fiducial value for the spectral index, running and tensor to scalar ratio, defined at the pivot scale $k_*=0.05\,{\mathrm {Mpc}}^{-1}$, as given by chaotic inflation with quadratic potential, i.e., $n_s = 0.968$, $\alpha _s=0$ and $r=0.128$. We have checked that for Planck data *r* is almost orthogonal to $n_s$ and $\alpha _s$. Therefore our result is not sensitive to the fiducial value of *r*.

The fiducial Euclid spectroscopically selected galaxies are split into 14 redshift bins. The redshift ranges and expected numbers of observed galaxies per unit volume $\bar{n}_{\mathrm {obs}}$ are taken from Laureijs et al. (2011) and shown in the third column of Table 3 in Sect. I.8.2 ($n_2(z)$). The number density of galaxies that can be used is $\bar{n}=\varepsilon \bar{n}_{\mathrm {obs}}$, where $\varepsilon$ is the fraction of galaxies with measured redshift. The boundaries of the wavenumber range used in the analysis, labeled $k_{\min }$ and $k_{\max }$, vary in the ranges $(0.00435\textendash 0.00334)h\mathrm {\ Mpc}^{-1}$ and $(0.16004\textendash 0.23644)h\mathrm {\ Mpc}^{-1}$ respectively, for $0.7\le z\le 2$. The IR cutoff $k_{\min }$ is chosen such that $k_{\min }r = 2\pi$, where *r* is the comoving distance of the redshift slice. The UV cutoff is the smallest between $\frac{H}{v_p(1+z)}$ and $\frac{\pi }{2R}$. Here *R* is chosen such that the r.m.s. linear density fluctuation of the matter field in a sphere with radius *R* is 0.5. In each redshift bin we use 30 *k*-bins uniformly in $\ln k$ and 20 uniform $\mu$-bins.

For the fiducial value of the bias, in each of the 14 redshift bins of width $\varDelta z=0.1$ in the range (0.7–2), we use those derived from Orsi et al. (2010), i.e. (1.083, 1.125, 1.104, 1.126, 1.208, 1.243, 1.282, 1.292, 1.363, 1.497, 1.486, 1.491, 1.573, 1.568), and we assume that $v_p$ is redshift dependent choosing $v_p=400\mathrm {\ km/s}$ as the fiducial value in each redshift bin. Then we marginalize over *b* and $v_p$ in the 14 redshift bins, for a total of 28 nuisance parameters.

**Table 21:** Cosmological parameters

Parameter	Planck 2015 constraint	EUCLID fiducial cosmology	$\hbox {Planck} + \hbox {Euclid}$ constraint
$\varOmega _bh^2$	$0.02238 \pm 0.00027$	0.022	$0.02227^{+0.00008}_{-0.00008}$
$\varOmega _ch^2$	$0.1180\pm 0.0021$	0.12	$0.1116^{+0.0002}_{-0.0002}$
$100 \theta$	$1.04111 \pm 0.00047$	1.041	$1.0392^{+0.0002}_{-0.0002}$
$\tau _{\mathrm {re}}$	$0.071\pm 0.018$	0.09	$0.085^{+0.003}_{-0.003}$
$n_s$	$0.9690\pm 0.0063$	0.96	$0.966^{+0.002}_{-0.002}$
$\alpha _s$	$-0.008^{+0.009}_{-0.008}$	0	$-0.000^{+0.003}_{-0.003}$
$\ln (10^{10}A_s)$	$3.073\pm 0.033$	3.098	$3.077^{+0.006}_{-0.006}$
$r_{0.05}$	$<\, 0.16 (95\% {\mathrm CL})$	0	$0.127^{+0.019}_{-0.018}$
$\varOmega _m$	$0.304\pm 0.013$	0.32	$0.271^{+0.001}_{-0.001}$
$\sigma _8$	$0.816 \pm 0.010$	0.83	$0.808^{+0.003}_{-0.003}$
*h*	$0.682 \pm 0.010$	0.67	$0.703^{+0.001}_{-0.001}$

In these two cases, we consider the forecast constraints on eight cosmological parameters, i.e., $\varOmega _bh^2$, $\varOmega _ch^2$, $\theta$, $\tau$, $\ln A_s$, $n_s$, $\alpha _s$, and *r*. Here $\theta$ is the angle subtended by the sound horizon on the last scattering surface, rescaled by a factor 100. We use the publicly available code CosmoMC (Lewis and Bridle 2002) to perform Markov Chain Monte Carlo calculation. The nuisance parameters are marginalized over in the final result. The marginalized 68.3% confidence level (CL) constraints on cosmological parameters for Planck only ($\hbox {Planck TT} + \hbox {lowP} + \hbox {lensing}$, for LCDM with r and running of the spectral index, Planck Collaboration 2015), and Planck and Euclid forecast are listed in the second and third columns of Table 21, respectively.

**Fig. 52:**
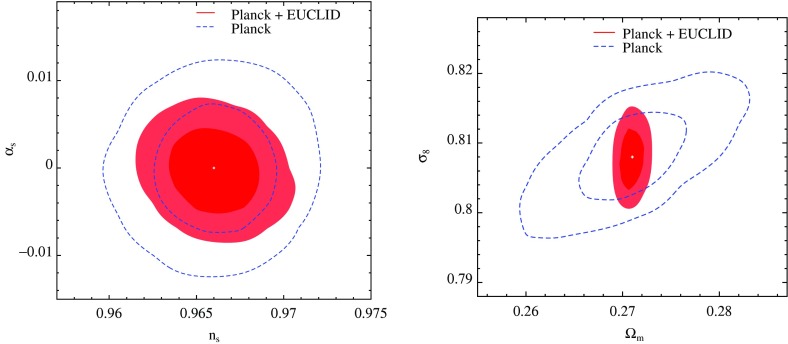
The marginalized likelihood contours (68.3 and 95.4% CL) for Planck forecast only (blue dashed lines) and Planck plus Euclid pessimistic (red filled contours). The white points correspond to the fiducial model

Euclid can improve the ‘figure of merit’ on the $n_s$-$\alpha _s$ plane by a factor of 2.2, as shown in the left panel of Fig. 52. Because the bias is unknown, the LSS data do not directly measure $A_s$ or $\sigma _8$. However, Euclid can measure $\varOmega _m$ to a much better accuracy, which can break the degeneracy between $\varOmega _m$ and $\sigma _8$ that one typically finds using CMB data alone. This is shown in the right panel of Fig. 52.

A more extensive and in depth analysis of what constraints on inflationary models a survey like Euclid can provide is presented in Huang et al. (2012b). In particular they find that for models where the primordial power spectrum is not featureless (i.e., close to a power law with small running) a survey like Euclid will be crucial to detect and measure features. Indeed, what we measure with the CMB is the angular power spectrum of the anisotropies in the 2-D multipole space, which is a projection of the power spectrum in the 3-D momentum space. Features at large $\ell$’s and for small width in momentum space get smoothed during this projection but this does not happen for large-scale structure surveys. The main limitation on the width of features measured using large-scale structure comes from the size of the volume of the survey: the smallest detectable feature being of the order of the inverse cubic root of this volume and the error being determined by number of modes contained in this volume. Euclid, with the large volume surveyed and the sheer number of modes that are sampled and cosmic variance dominated offers a unique opportunity to probe inflationary models where the potential is not featureless. In addition the increased statistical power would enable us to perform a Bayesian model selection on the space of inflationary models (e.g., Easther and Peiris 2012; Noreña et al. 2012b and references therein).

### Probing the early universe with non-Gaussianities

The workhorse for primordial non-Gaussianity has been so far the “local model” (Salopek and Bond 1990; Gangui et al. 1994; Verde et al. 2000b; Komatsu and Spergel 2001; Bartolo et al. 2004):

III.3.1 $$\begin{aligned} \varPhi =\phi +f_{\mathrm {NL}}\left( \phi ^2-\left\langle \phi ^2\right\rangle \right) . \end{aligned}$$

Here, $\phi$ is a Gaussian random field while $\varPhi$ denotes Bardeen’s gauge-invariant potential, which, on sub-Hubble scales reduces to the usual Newtonian peculiar gravitational potential, up to a minus sign. On large scales it is related to the conserved variable $\zeta$ by

III.3.2 $$\begin{aligned} \zeta = \frac{5 + 3 w}{3 + 3w} \varPhi , \end{aligned}$$

where *w* is the equation of state of the dominant component in the universe. The amount of primordial non-Gaussianity is quantified by the nonlinearity parameter $f_{\mathrm {NL}}$. Note that, since $\varPhi \simeq \phi \simeq 10^{-5}$, $f_{\mathrm {NL}}\sim 100$ corresponds to relative non-Gaussian corrections of order $10^{-3}$. While $\zeta$ is constant on large scales, $\varPhi$ is not. For this reason, in the literature there are two conventions for Eq. (III.3.1): the large-scale structure (LSS) and the cosmic microwave background (CMB) one. In the LSS convention, $\varPhi$ is linearly extrapolated at $z=0$; in the CMB convention $\varPhi$ is instead primordial: thus $f^{\mathrm {LSS}}_{\mathrm {NL}}=g(z=\infty )/g(0) f_{\mathrm {NL}}^{\mathrm {CMB}}\sim 1.3 \,f^{\mathrm {CMB}}_{\mathrm {NL}}$, where *g*(*z*) denotes the linear growth suppression factor relative to an Einstein–de Sitter universe. In the past few years it has become customary to always report $f_{\mathrm {NL}}^{\mathrm {CMB}}$ values even though, for simplicity as it will be clear below, one carries out the calculations with $f_{\mathrm {NL}}^{\mathrm {LSS}}$.

In this section we review the theoretical motivations and implications for looking into primordial non-Gaussianity; the readers less theoretically oriented can go directly to Sect. III.4.

#### Local non-Gaussianity

The non-Gaussianities generated in the conventional scenario of inflation (single-field with standard kinetic term, in slow-roll, initially in the Bunch–Davies vacuum) are predicted to be extremely small. Earlier calculations showed that $f_{\mathrm {NL}}$ would be of the order of the slow-roll parameters (Salopek and Bond 1990; Falk et al. 1993; Gangui et al. 1994). More recently, with an exact calculation, Maldacena (2003) confirmed this result and showed that the dominant contribution to non-Gaussianity comes from gravitational interaction and it is thus independent of the inflaton potential. More precisely, in the squeezed limit, i.e., when one of the modes is much smaller than the other two, the bispectrum of the primordial perturbation $\zeta$ is given by

III.3.3 $$\begin{aligned} B_\zeta (k_1 \ll k_2,k_3) = 4 f_{\mathrm {NL}}^{\mathrm {local}} P_\zeta (k_2) P_\zeta (k_3), \end{aligned}$$

where $f_{\mathrm {NL}}^{\mathrm {local}}$ is proportional to the tilt of scalar fluctuations, $f_{\mathrm {NL}}^{\mathrm {local}} = -\,(5/12) (n_s-1)$, a value much too small to be observable. Thus, any deviation from this prediction would rule out a large class of models based on the simplest scenario.

Furthermore, Creminelli and Zaldarriaga (2004) showed that irrespective of slow-roll and of the particular inflaton Lagrangian or dynamics, in single-field inflation, or more generally when only adiabatic fluctuations are present, there exists a consistency relation involving the 3-point function of scalar perturbations in the squeezed limit (see also Seery and Lidsey 2005; Chen et al. 2007b; Cheung et al. 2008b). In this limit, when the short wavelength modes are inside the Hubble radius during inflation, the long mode is far out of the horizon and its only effect on the short modes is to rescale the unperturbed history of the universe. This implies that the 3-point function is simply proportional to the 2-point function of the long wavelength modes times the 2-point function of the short wavelength mode times its deviation from scale invariance. In terms of local non-Gaussianity this translates into the same $f_{\mathrm {NL}}^{\mathrm {local}}$ found in Maldacena (2003). Thus, a convincing detection of local non-Gaussianity would rule out all classes of inflationary single-field models.

To overcome the consistency relation and produce large local non-Gaussianity one can go beyond the single-field case and consider scenarios where a second field plays a role in generating perturbations. In this case, because of non-adiabatic fluctuations, scalar perturbations can evolve outside the horizon invalidating the argument of the consistency relation and possibly generating a large $f_{\mathrm {NL}}^{\mathrm {local}}$ as in Linde and Mukhanov (1997). The curvaton scenario is one of such mechanisms. The curvaton is a light scalar field that acquires scale-invariant fluctuations during inflation and decays after inflation but well before nucleosynthesis (Mollerach 1990; Moroi and Takahashi 2001; Lyth and Wands 2002; Enqvist and Sloth 2002). During the decay it dominates the universe affecting its expansion history thus imprints its perturbations on super-horizon scales. The way the expansion history depends on the value of the curvaton field at the end of the decay can be highly nonlinear, leading to large non-Gaussianity. Indeed, the nonlinear parameter $f_{\mathrm {NL}}^{\mathrm {local}}$ is inversely proportional to the curvaton abundance before the decay (Lyth et al. 2003).

Models exists where both curvaton and inflaton fluctuations contribute to cosmological perturbations (Langlois and Vernizzi 2004). Interestingly, curvaton fluctuations could be negligible in the 2-point function but detectable through their non-Gaussian signature in the 3-point function, as studied in Boubekeur and Lyth (2006). We shall come back on this point when discussing isocurvature perturbations. Other models generating local non-Gaussianities are the so called modulated reheating models, in which one light field modulates the decay of the inflaton field (Dvali et al. 2004; Kofman 2003). Indeed, non-Gaussianity could be a powerful window into the physics of reheating and preheating, the phase of transition from inflation to the standard radiation dominated era (see, e.g., Bond et al. 2009; Chambers et al. 2010).

In the examples above only one field is responsible for the dynamics of inflation, while the others are spectators. When the inflationary dynamics is dominated by several fields along the $\sim \,60$ e-foldings of expansion from Hubble crossing to the end of inflation we are truly in the multi-field case. For instance, a well-studied model is double inflation with two massive non-interacting scalar fields (Polarski and Starobinsky 1992). In this case, the overall expansion of the universe is affected by each of the field while it is in slow-roll; thus, the final non-Gaussianity is slow-roll suppressed, as in single field inflation (Rigopoulos et al. 2006; Alabidi and Lyth 2006; Vernizzi and Wands 2006).

Because the slow-roll conditions are enforced on the fields while they dominate the inflationary dynamics, it seems difficult to produce large non-Gaussianity in multi-field inflation; however, by tuning the initial conditions it is possible to construct models leading to an observable signal (see Byrnes et al. 2008; Tanaka et al. 2010). Non-Gaussianity can be also generated at the end of inflation, where large-scale perturbations may have a nonlinear dependence on the non-adiabatic modes, especially if there is an abrupt change in the equation of state (see, e.g., Bernardeau and Uzan 2002; Lyth 2005). Hybrid models (Linde 1994), where inflation is ended by a tachyonic instability triggered by a waterfall field decaying in the true vacuum, are natural realizations of this mechanism (Enqvist and Väihkönen 2004; Barnaby and Cline 2006).

#### Shapes: what do they tell us?

As explained above, local non-Gaussianity is expected for models where nonlinearities develop outside the Hubble radius. However, this is not the only type of non-Gaussianity. Single-field models with derivative interactions yield a negligible 3-point function in the squeezed limit, yet leading to possibly observable non-Gaussianities. Indeed, as the interactions contain time derivatives and gradients, they vanish outside the horizon and are unable to produce a signal in the squeezed limit. Correlations will be larger for other configurations, for instance between modes of comparable wavelength. In order to study the observational signatures of these models we need to go beyond the local case and study the *shape* of non-Gaussianity (Babich et al. 2004).

Because of translational and rotational invariance, the 3-point function is characterized by a function of the modulus of the three wave-vectors, also called the bispectrum $B_{\zeta }(k_1,k_2,k_3)$, defined as

III.3.4 $$\begin{aligned} \langle \zeta _{\mathbf {k_1}} \zeta _{\mathbf {k_2}} \zeta _{\mathbf {k_3}} \rangle = (2 \pi )^3 \delta (\mathbf {k_1}+\mathbf {k_2}+\mathbf {k_3}) B_{\zeta }(k_1,k_2,k_3). \end{aligned}$$

Relaxing the assumption of a local $f_{\mathrm {NL}}$, this function is a rich object which can contain a wealth of information, depending on the size and shape of the triangle formed by $k_1$, $k_2$ and $k_3$. Indeed, the dependence of the bispectrum on configuration in momentum space is related to the particular inflationary model generating it. Namely, each third-order operator present in the field action gives rise to a particular shape of the bispectrum.

An example of models containing large derivative interactions has been proposed by Silverstein and Tong (2004) and Alishahiha et al. (2004). Based on the Dirac–Born–Infeld Lagrangian, ${{\mathcal {L}}} = f(\phi )^{-1} \sqrt{1- f(\phi ) X} +V(\phi )$, with $X= -\,g^{\mu \nu } \partial _\mu \phi \partial _\nu \phi$, it is called DBI inflation. This Lagrangian is string theory-motivated and $\phi$ describes the low-energy radial dynamics of a D3-brane in a warped throat: $f(\phi )^{-1}$ is the warped brane tension and $V(\phi )$ the interaction field potential. In this model the non-Gaussianity is dominated by derivative interactions of the field perturbations so that we do not need to take into account mixing with gravity. An estimate of the non-Gaussianity is given by the ratio between the third-order and the second order Lagrangians, respectively ${{\mathcal {L}}}_3$ and $\mathcal{L}_2$, divided by the amplitude of scalar fluctuations. This gives $f_{\mathrm {NL}} \sim ({{{\mathcal {L}}}_3}/{{{\mathcal {L}}}_2}) \varPhi ^{-1} \sim -1/{c_s^2}$, where $c_s^2 = [1+ 2 X (\partial ^2 {{\mathcal {L}}}/\partial X^2)/(\partial {{\mathcal {L}}}/\partial X)]^{-1} <1$ is the speed of sound of linear fluctuations and we have assumed that this is small, as it is the case for DBI inflation. Thus, the non-Gaussianity can be quite large if $c_s \ll 1$.

However, this signal vanishes in the squeezed limit due to the derivative interactions. More precisely, the particular momentum configuration of the bispectrum is very well described byIII.3.5where, up to numerical factors of order unity, $f_{\mathrm {NL}}^{\mathrm {equil}} \simeq -1/c_s^2$. The function of momenta inside the parenthesis is the *equilateral* shape (Creminelli et al. 2006), a *template* used to approximate a large class of inflationary models. It is defined in such a way as to be factorisable, maximized for equilateral configurations and vanishing in the squeezed limit faster than the local shape, see Eq. (III.3.3).

To compare two shapes $F_1$ and $F_2$, it is useful to define a 3-dimensional scalar product between them as (Babich et al. 2004)

III.3.6 $$\begin{aligned} F_1 \cdot F_2 = \sum F_1 (k_1,k_2,k_3) F_2 (k_1,k_2,k_3)/(P_\zeta (k_1)P_\zeta (k_2)P_\zeta (k_3)), \end{aligned}$$

where the sum is over all configurations forming a triangle. Then, $\cos \theta = F_1 \cdot F_2/\sqrt{(F_1 \cdot F_1)(F_2 \cdot F_2)}$ defines a quantitative measure of how much two shapes “overlap” and their signal is correlated. The cosine is small between the local and equilateral shapes. Two shapes with almost vanishing cosine are said to be orthogonal and any estimator developed to be sensitive to a particular shape will be completely blind to its orthogonal one. Note that the observable signal could actually be a combination of different shapes. For instance, multi-field models base on the DBI action (Langlois et al. 2008a) can generate a linear combination of local and equilateral non-Gaussianities (Renaux-Petel 2009).

The interplay between theory and observations, reflected in the relation between derivative interactions and the shape of non-Gaussianity, has motivated the study of inflation according to a new approach, the *effective field theory of inflation* (Cheung et al. 2008c; see also Weinberg 2008). Inflationary models can be viewed as effective field theories in presence of symmetries. Once symmetries are defined, the Lagrangian will contain each possible operator respecting such symmetries. As each operator leads to a particular non-Gaussian signal, constraining non-Gaussianity directly constrains the coefficients in front of these operators, similarly to what is done in high-energy physics with particle accelerators. For instance, the operator ${{\mathcal {L}}}_3$ discussed in the context of DBI inflation leads to non-Gaussianity controlled by the speed of sound of linear perturbations. This operator can be quite generic in single field models. Current constraints on non-Gaussianity allow to constrain the speed of sound of the inflaton field during inflation to be $c_s \ge 0.01$ (Cheung et al. 2008c; Senatore et al. 2010). Another well-studied example is ghost inflation (Arkani-Hamed et al. 2004a), based on the ghost condensation, a model proposed by Arkani-Hamed et al. (2004b) to modify gravity in the infrared. This model is motivated by shift symmetry and exploits the fact that in the limit where this symmetry is exact, higher-derivative operators play an important role in the dynamics, generating large non-Gaussianity with approximately equilateral shape.

Following this approach has allowed to construct operators or combination of operators leading to new shapes, orthogonal to the equilateral one. An example of such a shape is the *orthogonal* shape proposed in Senatore et al. (2010). This shape is generated by a particular combination of two operators already present in DBI inflation. It is peaked both on equilateral-triangle configurations and on flattened-triangle configurations (where the two lowest-*k* sides are equal exactly to half of the highest-*k* side)—the sign in this two limits being opposite. The orthogonal and equilateral are not an exhaustive list. For instance, Creminelli et al. (2010) have shown that the presence in the inflationary theory of an approximate Galilean symmetry (proposed by Nicolis et al. (2009) in the context of modified gravity) generates third-order operators with two derivatives on each field. A particular combination of these operators produces a shape that is approximately orthogonal to the three shapes discussed above.

Non-Gaussianity is also sensitive to deviations from the initial adiabatic Bunch–Davies vacuum of inflaton fluctuations. Indeed, considering excited states over it, as done in Chen et al. (2007b), Holman and Tolley (2008) and Meerburg et al. (2009), leads to a shape which is maximized in the collinear limit, corresponding to enfolded or squashed triangles in momentum space, although one can show that this shape can be written as a combination of the equilateral and orthogonal ones (Senatore et al. 2010).

At present, Planck 2015 data are compatible with Gaussian statistics and set tight constraints on primordial non-Gaussian shapes. The constraints at 68% CL are: $f_\text {NL}^\text {local}=2.5 \pm 5.7$ (after subtraction of the ISW-lensing contribution), $f_\text {NL}^\text {equil}= -\, 16 \pm 70$, and $f_\text {NL}^\text {ortho}= -\, 34 \pm 33$ (Planck Collaboration 2016a).

#### Beyond shapes: scale dependence and the squeezed limit

There is a way out to generate large non-Gaussianity in single-field inflation. Indeed, one can temporarily break scale-invariance, for instance by introducing features in the potential as in Chen et al. (2007a). This can lead to large non-Gaussianity typically associated with scale-dependence. These signatures could even teach us something about string theory. Indeed, in axion monodromy, a model recently proposed by Silverstein and Westphal (2008) based on a particular string compactification mechanism, the inflaton potential is approximately linear, but periodically modulated. These modulations lead to tiny oscillations in the power spectrum of cosmological fluctuations and to large non-Gaussianity (see, e.g., Flauger et al. 2010).

This is not the only example of scale dependence. While in general the amplitude of the non-Gaussianity signal is considered constant, there are several models, beside the above example, which predict a scale-dependence. For example models like the Dirac–Born–Infeld (DBI) inflation, e.g., Alishahiha et al. (2004), Chen (2005a, b), Bean et al. (2008a) can be characterized by a primordial bispectrum whose amplitude varies significantly over the range of scales accessible by cosmological probes.

In view of measurements from observations it is also worth considering the so-called squeezed limit of non-Gaussianity that is the limit in which one of the momenta is much smaller than the other two. Observationally this is because some probes (like, for example, the halo bias Sect. III.4.2, accessible by large-scale structure surveys like Euclid) are sensitive to this limit. Most importantly, from the theoretical point of view, there are consistency relations valid in this limit that identify different classes of inflation, e.g., Creminelli et al. (2011, 2012) and references therein.

Similar relationships can also be derived for multi-field models (e.g. Byrnes et al. 2012; Elliston et al. 2012). Particularly interesting is the so-called Suyama–Yamaguchi inequality (e.g., Suyama and Yamaguchi 2008; Smith et al. 2011; Assassi et al. 2012; Kehagias and Riotto 2012; Beltrán Almeida et al. 2013) which connects the amplitudes of the bi- and tri-spectra in models where non-Gaussianity is seeded by light fields different from the inflaton. Observational tests of the inequality could then provide information regarding the multi-field nature of the inflationary process. Forecasts for the Euclid weak-lensing survey have been presented by Grassi et al. (2014).

The scale dependence of non-Gaussianity, the shapes of non-gaussianity and the behavior of the squeezed limit are all promising avenues, where the combination of CMB data and large-scale structure surveys such as Euclid can provide powerful constraints as illustrated, e.g., in Sefusatti et al. (2009), Noreña et al. (2012a) and Sefusatti et al. (2012).

#### Beyond inflation

As explained above, the search of non-Gaussianity could represent a unique way to rule out the simplest of the inflationary models and distinguish between different scenarios of inflation. Interestingly, it could also open up a window on new scenarios, alternative to inflation. There have been numerous attempts to construct models alternative to inflation able to explain the initial conditions of our universe. In order to solve the cosmological problems and generate large-scale primordial fluctuations, most of them require a phase during which observable scales today have exited the Hubble size. This can happen in bouncing cosmologies, in which the present era of expansion is preceded by a contracting phase. Examples are the pre-Big Bang (Gasperini and Veneziano 1993) and the ekpyrotic scenario (Khoury et al. 2001).

In the latter, the 4-d effective dynamics corresponds to a cosmology driven by a scalar field with a steep exponential potential $V(\phi ) = \exp (-c \phi )$, with $c \gg 1$. Leaving aside the problem of the realization of the bounce, it has been shown that the adiabatic mode in this model generically leads to a steep blue spectrum for the curvature perturbations (Lyth 2002; Brandenberger and Finelli 2001; Creminelli et al. 2005). Thus, at least a second field is required to generate an almost scale-invariant spectrum of perturbations (Finelli 2002; Creminelli and Senatore 2007; Buchbinder et al. 2007; Koyama and Wands 2007; Fertig et al. 2014). If two fields are present, both with exponential potentials and steepness coefficients $c_1$ and $c_2$, the non-adiabatic component has negative mass and acquires a quasi invariant spectrum of fluctuations with tilt $n_s-1 = 4(c_1^{-2} + c_2^{-2})$, with $c_1,c_2 \gg 1$. Then one needs to convert the non-adiabatic fluctuation into curvature perturbation, similarly to what the curvaton mechanism does. If the two fields $\phi$ and $\chi$ are coupled through the kinetic term $\exp (-b \phi ) g^{\mu \nu } \partial _\mu \partial _\nu \chi$ (Di Marco et al. 2003) then nearly scale invariant perturbations are generated when $b \sim c$ and $\chi$ has no potential (Fertig et al. 2014).

As the Hubble rate increases during the collapse, one expects nonlinearities in the fields to become more and more important, leading to non-Gaussianity in the produced perturbations. As nonlinearities grow larger on super-Hubble scales, one expects the signal to be of local type. The particular amplitude of the non-Gaussianity in the observable curvature perturbations depends on the conversion mechanism from the non-adiabatic mode to the observable perturbations. The tachyonic instability itself can lead to a phase transition to an ekpyrotic phase dominated by just one field $\phi _1$. If the two fields have both an exponential potential, Koyama et al. (2007) have found that $f_{\mathrm {NL}}^{\mathrm {local}} = -\,(5/12)c_1^2$. Current constraints on $f_{\mathrm {NL}}^{\mathrm {local}}$ (Planck 2015 data imposes $f_{\mathrm {NL}}^{\mathrm {local}} = 2.5 \pm 5.7$ at 68% CL after subtraction of the ISW-lensing contribution, Planck Collaboration 2014e) gives a value at odd with Planck 2015 measurements of the spectral index. In fact in this model, even for $f_{\mathrm {NL}}=-\,10$, $c_2\simeq 5$ which implies a too large value of the scalar spectral index $n_s-1 > 0.17$) which is excluded by Planck measurements. Thus, one needs to modify the potential to accommodate a red spectrum or consider alternative conversion mechanisms to change the value of the generated non-Gaussianity (Buchbinder et al. 2008; Lehners and Steinhardt 2008). If the kinetic term of the second field is coupled to the first one as in Fertig et al. (2014) and Ijjas et al. (2014), then the generated non-Gaussianity is compatible with Planck 2015 constraints on $f_{\mathrm {NL}}$.

### Primordial non-Gaussianity and large-scale structure

As we have seen, even the simplest inflationary models predict deviations from Gaussian initial conditions. Confirming or ruling out the simplest inflationary model is an important goal and in this section we will show how Euclid can help achieving this. Moreover, Euclid data (alone or in combination with CMB experiments like Planck) can be used to explore the primordial bispectrum and thus explore the interaction of the fields during inflation.

#### Constraining primordial non-Gaussianity and gravity from 3-point statistics

Contrary to CMB research which mainly probes the high-redshift universe, current studies of the LSS focus on data at much lower redshifts and are more heavily influenced by cosmic evolution. Even if the initial conditions were Gaussian, nonlinear evolution due to gravitational instability generates a non-zero bispectrum for the matter distribution. The first non-vanishing term in perturbation theory (e.g., Catelan et al. 1995) gives

III.4.1 $$\begin{aligned} B(\mathbf {k}_1,\mathbf {k}_2,\mathbf {k}_3)=2(P(k_1) P(k_2)J(\mathbf {k}_1,\mathbf {k}_2)+ {\mathrm {cyclic\,\, permutations}}) \, \end{aligned}$$

where $J(\mathbf {k}_1,\mathbf {k}_2)$ is the gravitational instability “kernel” which depends very weakly on cosmology and for an Einstein-de-Sitter universe assumes the form:

III.4.2 $$\begin{aligned} J(\mathbf {k}_1,\mathbf {k}_2)=\frac{5}{7}+\frac{\mathbf {k}_1\cdot \mathbf {k}_2}{2k_1k_2}\left( \frac{k_1}{k_2}+\frac{k_2}{k_1}\right) +\frac{2}{7}\left( \frac{\mathbf {k}_1\cdot \mathbf {k}_2}{k_1 k_2}\right) ^2. \end{aligned}$$

This kernel represents the “signature” of gravity as we know it on the large-scale structure of the universe. Either a modification of the gravitational law or the introduction of a coupling between dark matter and another component (say dark energy) would alter the bispectrum shape from the standard form. The volume covered by Euclid will enable us to exploit this.

It was recognized a decade ago (Verde et al. 2000b) that the contribution to the matter bispectrum generated by gravitational instability is large compared to the fossil signal due to primordial non-Gaussianity and that the primordial signal “redshifts away” compared to the gravitational signal. In fact, primordial non-Gaussianity of the local type would affect the late-time dark matter density bispectrum with a contribution of the form

III.4.3 $$\begin{aligned} B^{f_{\mathrm {NL}}\, {\mathrm {local}}}(\mathbf {k}_1,\mathbf {k}_2,\mathbf {k}_3,z)= 2(f_{\mathrm {NL}}P(k_1)P(k_2)\frac{{{\mathcal {F}}}(\mathbf {k}_1,\mathbf {k}_2)}{D(z)/D(z=0)}\,+\, {\mathrm {cyclic\,permutations}}).\end{aligned}$$

where *D*(*z*) is the linear growth function which in an Einstein–de Sitter universe goes like $(1+z)^{-1}$ and

III.4.4 $$\begin{aligned} {{\mathcal {F}}}= \frac{{{\mathcal {M}}}(k_3)}{{{\mathcal {M}}}(k_1){{\mathcal {M}}}(k_2)}\,; \,\,\,{{\mathcal {M}}}(k)=\frac{2}{3}\frac{k^2 T(k)}{H_0^2\varOmega _{m,0}}, \end{aligned}$$

*T*(*k*) denoting the matter transfer function, $H_0$ the Hubble constant and $\varOmega _{m,0}$ the matter density parameter. Clearly the contributions due to primordial non-Gaussianity and gravitational instability have different scale and redshift dependence and the two kernel shapes in configuration space are different, thus, making the two components, at least in principle and for high signal-to-noise, separable. This is particularly promising for high-redshift probes of the matter distribution like the 21-cm background which should simultaneously provide competing measures of $f_{\mathrm {NL}}$ and a test of the gravitational law (Pillepich et al. 2007). Regrettably, these studies require using a long-wavelength radio telescope above the atmosphere (e.g., on the Moon) and will certainly come well after Euclid.

Galaxy surveys do not observe the dark matter distribution directly. However, dark matter halos are believed to host galaxy formation, and different galaxy types at different redshifts are expected to populate halos in disparate ways (Magliocchetti and Porciani 2003; Zehavi et al. 2005). A simple (and approximate) way to account for galaxy biasing is to assume that the overdensity in galaxy counts can be written as a truncated power expansion in terms of the mass overdensity (smoothed on some scale): $\delta _g(x)=b_1\delta _{\mathrm {DM}}(x)+b_2(\delta _{\mathrm {DM}}^2-\langle \delta _{\mathrm {DM}}^2 \rangle )$. The linear and quadratic bias coefficient $b_1$ and $b_2$ are assumed to be scale-independent (although this assumption must break down at some point) but they can vary with redshift and galaxy type. Obviously, a quadratic bias will introduce non-Gaussianity even on an initially Gaussian field. In summary, for local non-Gaussianity and scale-independent quadratic bias we have (Verde et al. 2000b):

III.4.5 $$\begin{aligned} B(\mathbf {k}_1,\mathbf {k}_2,\mathbf {k}_3,z)= & {} 2 P(k_1)P(k_2) b_1(z)^3\nonumber \\&\times \left[ f_{\mathrm {NL}}\frac{{{\mathcal {F}}}(\mathbf {k}_1,\mathbf {k}_2)}{D(z)} + J(\mathbf {k}_1,\mathbf {k}_2) +\frac{b_2(z)}{2 b_1(z)}\right] + \mathrm {cyc.}\qquad \qquad \end{aligned}$$

Before the above expression can be compared against observations, it needs to be further complicated to account for redshift-space distortions and shot noise. Realistic surveys use galaxy redshifts as a proxy for distance, but gravitationally-induced peculiar velocities distort the redshift-space galaxy distribution. At the same time, the discrete nature of galaxies gives rise to corrections that should be added to the bispectrum computed in the continuous limit. We will not discuss these details here as including redshift space distortions and shot noise will not change the gist of the message.

From the observational point of view, it is important to note that photometric surveys are not well suited for extracting a primordial signal out of the galaxy bispectrum. Although in general they can cover larger volumes than spectroscopic surveys, the projection effects due to the photo-z smearing along the line-of-sight is expected to suppress significantly the sensitivity of the measured bispectrum to the shape of the primordial one (see, e.g., Verde et al. 2000a). Sefusatti and Komatsu (2007) have shown that, if the evolution of the bias parameters is known a priori, spectroscopic surveys like Euclid would be able to give constraints on the $f_{\mathrm {NL}}$ parameter that are competitive with CMB studies. While the gravitationally-induced non-Gaussian signal in the bispectrum has been detected to high statistical significance (see, e.g., Verde et al. 2002; Kulkarni et al. 2007 and references therein), the identification of nonlinear biasing (i.e., $b_2\ne 0$) is still controversial, and there has been so far no detection of any extra (primordial) bispectrum contributions.

Of course, one could also consider higher-order correlations. One of the advantages of considering, e.g., the trispectrum is that, contrary to the bispectrum, it has very weak nonlinear growth (Verde and Heavens 2001), but it has the disadvantage that the signal is de-localized: the number of possible configurations grows fast with the dimensionality *n* of the *n*-point function!

Finally, it has been proposed to measure the level of primordial non-Gaussianity using Minkowski functionals applied either to the galaxy distribution or the weak lensing maps (see, e.g., Hikage et al. 2008; Munshi et al. 2011 and references therein). The potentiality of this approach compared to more traditional methods needs to be further explored in the near future.

#### Non-Gaussian halo bias

The discussion above neglects an important fact which went unnoticed until year 2008: the presence of small non-Gaussianity can have a large effect on the clustering of dark matter halos (Dalal et al. 2008; Matarrese and Verde 2008). The argument goes as follows. The clustering of the peaks in a Gaussian random field is completely specified by the field power spectrum. Thus, assuming that halos form out of linear density peaks, for Gaussian initial conditions the clustering of the dark matter halos is completely specified by the linear matter power spectrum. On the other hand, for a non-Gaussian field, the clustering of the peaks depends on all higher-order correlations, not just on the power spectrum. Therefore, for non-Gaussian initial conditions, the clustering of dark matter halos depends on the linear bispectrum (and higher-order moments).

One can also understand the effect in the peak-background-split framework: overdense patches of the (linear) universe collapse to form dark matter halos if their overdensity lies above a critical collapse threshold. Short-wavelength modes define the overdense patches while the long-wavelength modes determine the spatial distribution of the collapsing ones by modulating their height above and below the critical threshold. In the Gaussian case, long- and short-wavelength modes are uncorrelated, yielding the well known linear, scale-independent peak bias. In the non-Gaussian case, however, long and short wavelength modes are coupled, yielding a different spatial pattern of regions that cross the collapse threshold.

In particular, for primordial non-Gaussianity of the local type, the net effect is that the halo distribution on very large scales relates to the underlying dark matter in a strongly scale-dependent fashion. For $k\lesssim 0.02\,h\mathrm {\ Mpc}^{-1}$, the effective linear bias parameter scales as $k^{-2}$ (Dalal et al. 2008; Matarrese and Verde 2008; Giannantonio and Porciani 2010). This is because the halo overdensity depends not only on the underlying matter density but also on the value of the auxiliary Gaussian potential $\phi$ (Giannantonio and Porciani 2010).

**Fig. 53:**
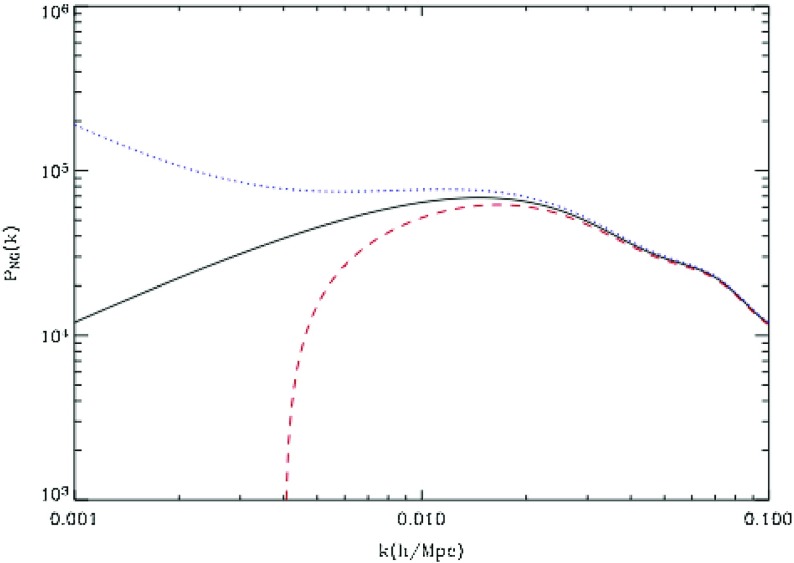
For illustration purposes this is the effect of a local $f_{\mathrm {NL}}$ of $\pm \, 50$ on the $z=0$ power spectrum of halos with mass above $10^{13}\,M_{\odot }$

The presence of this effect is extremely important for observational studies as it allows to detect primordial non-Gaussianity from 2-point statistics of the galaxy distribution like the power spectrum. Combining current LSS data gives constraints on $f_{\mathrm {NL}}$ which are comparable to the pre-Planck CMB ones (Slosar et al. 2008; Xia et al. 2010; Giannantonio et al. 2014). Similarly, planned galaxy surveys are expected to progressively improve upon existing limits (Carbone et al. 2008, 2010; Giannantonio et al. 2012b). For example, Euclid could reach an error on $f_{\mathrm {NL}}$ of $\sim \,5$ (see below for further details) which is comparable with the BPol forecast errors.

The scale dependence of the halo bias changes considering different shapes of primordial non-Gaussianity (Schmidt and Kamionkowski 2010; Wagner et al. 2010). For instance, orthogonal and folded models produce an effective bias that scales as $k^{-1}$ while the scale dependence becomes extremely weak for equilateral models.^22^ Therefore, measurements of the galaxy power spectrum on the largest possible scales have the possibility to constrain the shape and the amplitude of primordial non-Gaussianity and thus shed new light on the dynamics of inflation.

On scales comparable with the Hubble radius, matter and halo clustering are affected by general-relativity effects: the Poisson equation gets a quadratic correction that acts effectively as a non-zero local $f_{\mathrm {NL}}$ (Bartolo et al. 2005; Pillepich et al. 2007). This contribution is peculiar to the inflationary initial conditions because it requires perturbations on super-horizon scales and it is mimicked in the halo bias by a local $f_{\mathrm {NL}}=-\,1.6$ (Verde and Matarrese 2009). This is at the level of detectability by a survey like Euclid.

#### Number counts of nonlinear structures

Even a small deviation from Gaussianity in the initial conditions can have a strong impact on those statistics which probe the tails of the linear density distribution. This is the case for the abundance of the most extreme nonlinear objects existing at a given cosmic epoch, massive dark matter halos and voids, as they correspond to the highest and lowest density peaks (the rarest events) in the underlying linear density field.

Thus small values of $f_{\mathrm {NL}}$ are potentially detectable by measuring the abundance of massive dark matter halos as traced by galaxies and galaxy clusters at $z \gtrsim 1$ (Matarrese et al. 2000). This approach has recently received renewed attention (e.g., LoVerde et al. 2008; Grossi et al. 2009; Pillepich et al. 2010; Maggiore and Riotto 2010; D’Amico et al. 2011a; Verde 2010a; Pillepich et al. 2012 and references therein) and might represent a promising tool for Euclid science. In Euclid, galaxy clusters at high redshift can be identified either by lensing studies or by building group catalogs based on the spectroscopic and photometric galaxy data. The main challenge here is to determine the corresponding halo mass with sufficient accuracy to allow comparison with the theoretical models.

While galaxy clusters form at the highest overdensities of the primordial density field and probe the high-density tail of the PDF, voids form in the low-density regions and thus probe the low-density tail of the PDF. Most of the volume of the evolved universe is underdense, so it seems interesting to pay attention to the distribution of underdense regions. For the derivation of the non-Gaussian void probability function one proceeds in parallel to the treatment for halos with the only subtlety that the critical threshold is not negative and that its numerical value depends on the precise definition of a void (and may depend on the observables used to find voids), e.g., Kamionkowski et al. (2009). Note that while a positive skewness ($f_{\mathrm {NL}}>0$) boosts the number of halos at the high mass end (and slightly suppress the number of low-mass halos), it is a negative skewness that will increase the voids size distribution at the largest voids end (and slightly decrease it for small void sizes). In addition voids may probe slightly larger scales than halos, making the two approaches highly complementary.

Even though a number of observational techniques to detect voids in galaxy surveys have been proposed (see, e.g., Colberg et al. 2008 and references therein), the challenge here is to match the theoretical predictions to a particular void-identification criterion based on a specific galaxy sample. We envision that mock galaxy catalogs based on numerical simulations will be employed to calibrate these studies for Euclid.

#### Forecasts for Euclid

A number of authors have used the Fisher-matrix formalism to explore the potentiality of Euclid in determining the level and the shape of primordial non-Gaussianity (Carbone et al. 2008, 2010; Giannantonio et al. 2012b). In what follows, unless specifically mentioned, we will focus on the local type of non-Gaussianity which has been more widely studied so far.

The most promising avenue is exploiting the scale-dependent bias on very large scales in studies of galaxy clustering at the two-point level. Early Fisher forecasts for the Euclid redshift survey found that, for a fiducial model with $f_{\mathrm {NL}}=0$, this gives a marginalized $1\sigma$ error on the nonlinearity parameter of $\varDelta f_{\mathrm {NL}}\simeq 2$ (Carbone et al. 2008, 2010). Forecasts based on the most recent specifics for the Euclid surveys (see Table 22) are presented in Giannantonio et al. (2012b) and summarized in Table 23 below. Updated values of the galaxy number counts and of the efficiency in measuring spectroscopic redshifts correspond to a marginalized $1\sigma$ error of $f_{\mathrm {NL}} \simeq 4\textendash 5$ (depending a little on the detailed assumptions of the Fisher matrix calculation), with a slightly better result obtained using the Euclid spectroscopic sample rather than the photometric one (complemented with multi-band ground-based photometry), at least for a fiducial value of $f_{\mathrm {NL}}=0$ (Giannantonio et al. 2012b). The forecast errors further improve by nearly a few per cent using Planck priors on the cosmological parameters determined with the power spectrum of CMB temperature anisotropies.

**Table 22:** Specifications of the surveys used in the Euclid forecasts given in Table 23

	Photometric survey	Spectroscopic survey
Surveyed area (deg$^2$)	15,000	15,000
Galaxy density (arcmin$^{-2}$)	30	0.56
Median redshift	0.8	1.0
Number of redshift bins	12	12
Redshift uncertainty $\sigma _z/(1+z)$	0.05	0.001
Intrinsic ellipticity noise $\gamma$	–	0.247
Gaussian linear bias param.	$\sqrt{1+z}$	$\sqrt{1+z}$

The redshift distributions of the different galaxy samples are as in Sect. I.8.2 (see also Giannantonio et al. 2012b)

**Table 23:** Forecast $1\sigma$ errors for the nonlinearity parameter $f_{\mathrm {NL}}$ based on two-point statistics (power spectra) of the Euclid redshift and weak-lensing surveys

Bispectrum shape	Local	Orthogonal	Equilateral
Fiducial $f_{\mathrm {NL}}$	0	0	0
Galaxy clustering (spectr. *z*)	4.1 (4.0)	54 (11)	220 (35)
Galaxy clustering (photom. *z*)	5.8 (5.5)	38 (9.6)	140 (37)
Weak lensing	73 (27)	9.6 (3.5)	34 (13)
Combined	4.7 (4.5)	4.0 (2.2)	16 (7.5)

Results are obtained using the Fisher-matrix formalism and marginalizing over eight cosmological parameters ($\varOmega _\varLambda$, $\varOmega _m$, $\varOmega _b$, *h*, $n_s$, $\sigma _8$, $w_0$, $w_a$) plus a large number of nuisance parameters to account for galaxy biasing, nonlinear redshift-space distortions and shot noise (see Giannantonio et al. 2012b for details). Results within parentheses include the forecast priors for the cosmological parameters from the power spectrum of CMB temperature anisotropies measured with the Planck satellite (note that no prior is assumed on $f_{\mathrm {NL}}$). The label “Galaxy clustering” refers to the anisotropic power spectrum $P(k_\parallel ,k_\perp )$ for spectroscopic data and to the angular power spectrum $C_\ell$ for photometric data. The combined analysis of clustering and lensing data is based on angular power spectra and includes all possible cross-correlations between different redshift bins and probes. nonlinear power spectra are computed using the halo model. This introduces possible inaccuracies in the forecasts for weak lensing data in the equilateral and orthogonal shapes (see main text for details)

The amplitude and shape of the matter power spectrum in the mildly nonlinear regime depend (at a level of a few per cent) on the level of primordial non-Gaussianity (Taruya et al. 2008; Pillepich et al. 2010; Giannantonio and Porciani 2010). Measuring this signal with the Euclid weak-lensing survey gives $\varDelta f_{\mathrm {NL}}\simeq 70$ (30 with Planck priors) (Giannantonio et al. 2012b). On the other hand, counting nonlinear structures in terms of peaks in the weak-lensing maps (convergence or shear) should give limits in the same ballpark (Marian et al. 2010 find $\varDelta f_{\mathrm {NL}}=13$ assuming perfect knowledge of all the cosmological parameters).

Finally, by combining lensing and angular power spectra (and accounting for all possible cross-correlations) one should achieve $\varDelta f_{\mathrm {NL}}\simeq 5$ (4.5 with Planck priors) (Giannantonio et al. 2012b). This matches what is expected from both the Planck mission and the proposed BPol satellite.

Note that the forecast errors on $f_{\mathrm {NL}}$ are somewhat sensitive to the assumed fiducial values of the galaxy bias. In our study we have adopted the approximation $b(z)=\sqrt{1+z}$ (Rassat et al. 2008). On the other hand, using semi-analytic models of galaxy formation, Orsi et al. (2010) found bias values which are nearly 10–15% lower at all redshifts. Adopting this slightly different bias, the constraint on $f_{\mathrm {NL}}$ already degrades by 50% with respect to our fiducial case.

Euclid data can also be used to constrain the scale dependence of the nonlinearity parameter (see Table 24). To this purpose, we consider a local model of primordial non-Gaussianity where

III.4.6 $$\begin{aligned} f_{\mathrm {NL}}=f_{\mathrm {NL}}^{\mathrm {(piv)}}\cdot \left( \frac{k}{k_{\mathrm {piv}}}\right) ^{n_{f_{\mathrm {NL}}}}, \end{aligned}$$

with fiducial values $k_{\mathrm {piv}}=0.02\, h\mathrm {\ Mpc}^{-1}$, $f_{\mathrm {NL}}^{\mathrm {(piv)}}=30$, and $n_{f_{\mathrm {NL}}}=0$. In this case, the combination of lensing and clustering data gives $\varDelta \alpha _{\mathrm {s,m}}=0.18$ (0.14 with Planck priors) and $\varDelta f_{\mathrm {NL}}^{\mathrm {(piv)}}\simeq 9$ (7 with Planck priors) (Giannantonio et al. 2012b). These constraints are similar to what is expected from future studies of the CMB bispectrum with Planck (Sefusatti et al. 2009).

**Table 24:** Forecast $1\sigma$ errors for a scale-dependent local model of primordial non-Gaussianity (Giannantonio et al. 2012b). Details of the forecasts are as in the previous Table 23

	$\varDelta f_{\mathrm {NL}}^{\mathrm {(piv)}}$	$\varDelta n_{f_{\mathrm {NL}}}$
Galaxy clustering (spectr. *z*)	9.3 (7.2)	0.28 (0.21)
Galaxy clustering (photom. *z*)	25 (18)	0.38 (0.26)
Weak lensing	134 (82)	0.66 (0.59)
Combined	8.9 (7.4)	0.18 (0.14)

In the end, we briefly comment on how well Euclid data could constrain the amplitude of alternative forms of primordial non-Gaussianity than the local one. In particular, we consider the equilateral and orthogonal shapes introduced in Sect. III.3.2. Table 23 summarizes the resulting constraints on the amplitude of the primordial bispectrum, $f_{\mathrm {NL}}$. The forecast errors from galaxy clustering grow larger and larger when one moves from the local to the orthogonal and finally to the equilateral model. This reflects the fact that the scale-dependent part of the galaxy bias for $k\rightarrow 0$ approximately scales as $k^{-2}$, $k^{-1}$, and $k^0$ for the local, orthogonal, and equilateral shapes, respectively (Schmidt and Kamionkowski 2010; Wagner et al. 2010; Scoccimarro et al. 2012; Desjacques et al. 2011a, b). On the other hand, the lensing constraints (that, in this case, come from the very nonlinear scales) appear to get much stronger for the non-local shapes. A note of caution is in order here. In Giannantonio et al. (2012b), the nonlinear matter power spectrum is computed using a halo model which has been tested against *N*-body simulations only for non-Gaussianity of the local type.^23^ In consequence, the weak-lensing forecasts might be less reliable than in the local case (see the detailed discussion in Giannantonio et al. 2012b). This does not apply for the forecasts based on galaxy clustering which are always robust as they are based on the scale dependence of the galaxy bias on very large scales.

#### Complementarity

The CMB bispectrum is very sensitive to the shape of non-Gaussianity; halo bias and mass function, the most promising approaches to constrain $f_{\mathrm {NL}}$ with a survey like Euclid, are much less sensitive. However, it is the complementarity between CMB and LSS that matters. One could envision different scenarios. If non-Gaussianity is local with negative $f_{\mathrm {NL}}$ and CMB obtains a detection, then the halo bias approach should also give a high-significance detection (GR correction and primordial contributions add up), while if it is local but with positive $f_{\mathrm {NL}}$, the halo-bias approach could give a lower statistical significance as the GR correction contribution has the opposite sign. If CMB detects $f_{\mathrm {NL}}$ at the level of 10 and a form that is close to local, but halo bias does not detect it, then the CMB bispectrum is given by secondary effects (e.g., Mangilli and Verde 2009). If CMB detects non-Gaussianity that is not of the local type, then halo bias can help discriminate between equilateral and enfolded shapes: if halo bias sees a signal, it indicates the enfolded type, and if halo bias does not see a signal, it indicates the equilateral type. Thus even a non-detection of the halo-bias effect, in combination with CMB constraints, can have an important discriminative power.

### Isocurvature modes

At some time well after inflation but deep into the radiation era the universe is filled with several components. For instance, in the standard picture right before recombination there are four components: baryons, cold dark matter, photons and neutrinos. One can study the distribution of *super-Hubble* fluctuations between different species, which represent the initial conditions for the subsequent evolution. So far we have investigated mostly the adiabatic initial conditions; in this section we explore more generally the possibility of isocurvature initial conditions. Although CMB data are the most sensitive to constrain isocurvature perturbations, we discuss here the impact on Euclid results.

#### The origin of isocurvature perturbations

Let us denote by $\rho _\alpha$ the energy density of the component $\alpha$. Perturbations are purely adiabatic when for each component $\alpha$ the quantity $\zeta _\alpha \equiv - 3 H \delta \rho _\alpha /{\dot{\rho }}_\alpha$ is the same (Weinberg 2003; Malik et al. 2003). Let us consider for instance cold dark matter and photons. When fluctuations are adiabatic it follows that $\zeta _{\mathrm {cdm}} = \zeta _\gamma$. Using the energy conservation equation, ${\dot{\rho }}_\alpha = -\, 3 H (\rho _\alpha + p_\alpha )$ with $p_{\mathrm {cdm}}=0$ and $p_\gamma = \rho _\gamma /3$, one finds that the density contrasts of these species are related by

III.5.1 $$\begin{aligned} \frac{\delta \rho _{\mathrm {cdm}}}{\rho _{\mathrm {cdm}}} = \frac{3}{4} \frac{\delta \rho _\gamma }{\rho _\gamma }. \end{aligned}$$

Using that $n_{\mathrm {cdm}} \propto \rho _{\mathrm {cdm}}$ and $n_\gamma \propto \rho _\gamma ^{3/4}$, this also implies that particle number ratios between these species is fixed, i.e., $\delta (n_{\mathrm {cdm}}/n_\gamma ) = 0$.

When isocurvature perturbations are present, the condition described above is not satisfied.^24^ In this case one can define a non-adiabatic or entropic perturbation between two components $\alpha$ and $\beta$ as ${{\mathcal {S}}}_{\alpha , \beta } \equiv \zeta _\alpha -\zeta _\beta$, so that, for the example above one has

III.5.2 $$\begin{aligned} {{\mathcal {S}}}_{{\mathrm {cdm}},r } = \frac{\delta \rho _{\mathrm {cdm}}}{ \rho _{\mathrm {cdm}}} - \frac{3}{4} \frac{\delta \rho _\gamma }{ \rho _\gamma } = \frac{\delta (n_{\mathrm {cdm}}/n_\gamma )}{n_{\mathrm {cdm}}-n_\gamma }. \end{aligned}$$

A sufficient condition for having purely adiabatic perturbations is that all the components in the universe were created by a single degree of freedom, such as during reheating after single field inflation.^25^ Even if inflation has been driven by several fields, thermal equilibrium may erase isocurvature perturbations if it is established before any non-zero conserving quantum number was created (see Weinberg 2004). Thus, a detection of non-adiabatic fluctuations would imply that several scalar fields where present during inflation *and* that either some of the species were not in thermal equilibrium afterwards or that some non-zero conserving quantum number was created before thermal equilibrium.

The presence of many fields is not unexpected. Indeed, in all the extension of the Standard Model scalar fields are rather ubiquitous. In particular, in String Theory dimensionless couplings are functions of moduli, i.e., scalar fields describing the compactification. Another reason to consider the relevant role of a second field other than the inflaton is that this can allow to circumvent the necessity of slow-roll (see, e.g., Dvali and Kachru 2003) enlarging the possibility of inflationary models.

Departure from thermal equilibrium is one of the necessary conditions for the generation of baryon asymmetry and thus of the matter in the universe. Interestingly, the oscillations and decay of a scalar field requires departure from thermal equilibrium. Thus, baryon asymmetry can be generated by this process; examples are the decay of a right-handed sneutrino (Hamaguchi et al. 2002) or the Affleck and Dine (1985) scenario. If the source of the baryon-number asymmetry in the universe is the condensation of a scalar field after inflation, one expects generation of baryon isocurvature perturbations (Moroi and Takahashi 2001). This scalar field can also totally or partially generate adiabatic density perturbations through the curvaton mechanism.

In summary, given our ignorance about inflation, reheating, and the generation of matter in the universe, a discovery of the presence of isocurvature initial conditions would have radical implications on both the inflationary process and on the mechanisms of generation of matter in the universe.

Let us concentrate on the non-adiabatic perturbation between cold dark matter (or baryons, which are also non-relativistic) and radiation ${{\mathcal {S}}} = {{\mathcal {S}}}_{{\mathrm {cdm}}, \gamma }$. Constraints on the amplitude of the non-adiabatic component are given in terms of the parameter $\alpha$, defined at a given scale $k_0$, by $P_{{\mathcal {S}}}-P_\zeta \equiv \alpha -(1-\alpha )$, see e.g., Beltrán et al. (2004), Bean et al. (2006) and Komatsu et al. (2009). As discussed in Langlois (1999), adiabatic and entropy perturbations may be correlated. To measure the amplitude of the correlation one defines a cross-correlation coefficient, $\beta \equiv - P_{{{\mathcal {S}}}, \zeta }/\sqrt{P_{{\mathcal {S}}} P_\zeta }$. Here $P_{{{\mathcal {S}}}, \zeta }$ is the cross-correlation power-spectrum between ${{\mathcal {S}}}$ and $\zeta$ and for the definition of $\beta$ we have adopted the sign convention of Komatsu et al. (2009). Observables, such as for instance the CMB anisotropies, depend on linear combinations of $\zeta$ and ${{\mathcal {S}}}$. Thus, constraints on $\alpha$ will considerably depend on the cross-correlation coefficient $\beta$ (see, e.g, discussion in Gordon and Lewis 2003).

If part of the cold dark matter is created out of equilibrium from a field other than the inflaton, totally uncorrelated isocurvature perturbations, with $\beta =0$, are produced, as discussed for instance in Efstathiou and Bond (1986) and Linde and Mukhanov (1997). The axion is a well-known example of such a field. The axion is the Nambu–Goldstone boson associated with the Peccei and Quinn (1977) mechanism to solve the strong-CP problem in QCD. As it acquires a mass through QCD non-perturbative effects, when the Hubble rate drops below its mass the axion starts oscillating coherently, behaving as cold dark matter (Preskill et al. 1983; Abbott and Sikivie 1983; Dine and Fischler 1983). During inflation, the axion is practically massless and acquires fluctuations which are totally uncorrelated from photons, produced by the inflaton decay (Seckel and Turner 1985; Linde 1985, 1991; Turner and Wilczek 1991). Planck data 2015 set a tight constrain on CDM uncorrelated isocurvature fluctuations: $\alpha _{\beta =0} < 0.037$ at 95% CL (Planck Collaboration 2016b).

Totally uncorrelated isocurvature perturbations can also be produced in the curvaton mechanism, if the dark matter or baryons are created from inflation, before the curvaton decay, and remain decoupled from the product of curvaton reheating (Langlois et al. 2008b). This scenario is ruled out if the curvaton is entirely responsible for the curvature perturbations. However, in models when the final curvature perturbation is a mix of the inflaton and curvaton perturbations (Langlois and Vernizzi 2004), such an entropy contribution is still allowed.

When dark matter or baryons are produced solely from the curvaton decay, such as discussed by Lyth et al. (2003), the isocurvature perturbations are totally anti-correlated, with $\beta =-\,1$.^26^ The Planck 2015 constrains the fraction of this isocurvature CDM modes to $\alpha _{\beta =0} < 0.0018$ at 95% CL (Planck Collaboration 2016b), which implies that the curvaton has decayed in CDM when it contributed to most of the energy content of the Universe.

For instance, some fraction of the curvaton decays to produce CDM particles or the out-of-equilibrium curvaton decay generates the primordial baryon asymmetry (Hamaguchi et al. 2002; Affleck and Dine 1985).

If present, isocurvature fields are not constrained by the slow-roll conditions imposed on the inflaton field to drive inflation. Thus, they can be highly non-Gaussian (Linde and Mukhanov 1997; Bernardeau and Uzan 2002). Even though negligible in the two-point function, their presence could be detected in the three-point function of the primordial curvature and isocurvature perturbations and their cross-correlations, as studied in Kawasaki et al. (2008) and Langlois et al. (2008b).

#### Constraining isocurvature perturbations

Even if pure isocurvature models have been ruled out, current observations allow for mixed adiabatic and isocurvature contributions (e.g., Crotty et al. 2003; Trotta 2007c; Komatsu et al. 2009; Väliviita and Giannantonio 2009). As shown in Trotta et al. (2001), Amendola et al. (2002), Väliviita and Giannantonio (2009), Langlois and Riazuelo (2000), Bucher et al. (2001) and Sollom et al. (2009), the initial conditions issue is a very delicate problem: in fact, for current cosmological data, relaxing the assumption of adiabaticity reduces our ability to do precision cosmology since it compromises the accuracy of parameter constraints. Generally, allowing for isocurvature modes introduces new degeneracies in the parameter space which weaken constraints considerably.

The cosmic microwave background radiation (CMB), being our window on the early universe, is the preferred data set to learn about initial conditions. Up to now, however, the CMB temperature power spectrum alone, which is the CMB observable better constrained so far, has not been able to break the degeneracy between the nature of initial perturbations (i.e., the amount and properties of an isocurvature component) and cosmological parameters, e.g., Kurki-Suonio et al. (2005) and Trotta et al. (2001). Even if the precision measurement of the CMB first acoustic peak at $\ell \simeq 220$ ruled out the possibility of a dominant isocurvature mode, allowing for isocurvature perturbations together with the adiabatic ones introduce additional degeneracies in the interpretation of the CMB data that current experiments could not break. Adding external data sets somewhat alleviates the issue for some degeneracy directions, e.g., Trotta et al. (2003), Beltrán et al. (2004) and Dunkley et al. (2005). As shown in Bucher et al. (2001), the precision polarization measurement of the next CMB experiments like Planck will be crucial to lift such degeneracies, i.e., to distinguish the effect of the isocurvature modes from those due to the variations of the cosmological parameters.

It is important to keep in mind that analyzing the CMB data with the prior assumption of purely adiabatic initial conditions when the real universe contains even a small isocurvature contribution, could lead to an incorrect determination of the cosmological parameters and on the inferred value of the sound horizon at radiation drag. The sound horizon at radiation drag is the standard ruler that is used to extract information about the expansion history of the universe from measurements of the baryon acoustic oscillations. Even for a CMB experiment like Planck, a small but non-zero isocurvature contribution, still allowed by Planck data—see Planck Collaboration (2014c)—, if ignored, can introduce a systematic error in the interpretation of the BAO signal that is comparable if not larger than the statistical errors. In fact, Mangilli et al. (2010) show that even a tiny amount of isocurvature perturbation, if not accounted for, could affect standard rulers calibration from CMB observations such as those provided by the Planck mission, affect BAO interpretation, and introduce biases in the recovered dark energy properties that are larger than forecast statistical errors from future surveys. In addition it will introduce a mismatch of the expansion history as inferred from CMB and as measured by BAO surveys. The mismatch between CMB predicted and the measured expansion histories has been proposed as a signature for deviations from a DM cosmology in the form of deviations from Einstein’s gravity (e.g., Acquaviva and Verde 2007; Ishak et al. 2006), couplings in the dark sector (e.g., Honorez et al. 2010) or time-evolving dark energy.

For the above reasons, extending on the work of Mangilli et al. (2010) and Carbone et al. (2011a) adopted a general fiducial cosmology which includes a varying dark energy equation of state parameter and curvature. In addition to BAO measurements, in this case the information from the shape of the galaxy power spectrum are included and a joint analysis of a Planck-like CMB probe and a Euclid-type survey is considered. This allows one to break the degeneracies that affect the CMB and BAO combination. As a result, most of the cosmological parameter systematic biases arising from an incorrect assumption on the isocurvature fraction parameter $f_{\mathrm {iso}}$, become negligible with respect to the statistical errors. The combination of CMB and LSS gives a statistical error $\sigma (f_{\mathrm {iso}}) \sim 0.008$, even when curvature and a varying dark energy equation of state are included, which is smaller than the error obtained from CMB alone when flatness and cosmological constant are assumed. These results confirm the synergy and complementarity between CMB and LSS, and the great potential of future and planned galaxy surveys.

### Summary and outlook

We have summarized aspects of the initial conditions for the growth of cosmological perturbations that Euclid will enable us to probe. In particular we have considered the shape of the primordial power spectrum and its connection to inflationary models, primordial non-Gaussianity and isocurvature perturbations.

A survey like Euclid will greatly improve our knowledge of the initial conditions for the growth of perturbations and will help shed light on the mechanism for the generation of primordial perturbations. The addition of Euclid data will improve the Planck satellite’s cosmic microwave background constraints on parameters describing the shape of the primordial power spectrum by a factor of 2–3.

Primordial non-Gaussianity can be tested by Euclid in three different and complementary ways: via the galaxy bispectrum, number counts of nonlinear structures and the non-Gaussian halo bias. These approaches are also highly competitive with and complementary to CMB constraints. In combination with Planck, Euclid will not only test a possible scale-dependence of non-Gaussianity but also its shape. The shape of non-Gaussianity is the key to constrain and classify possible deviations for the simplest single-field slow roll inflation.

Isocurvature modes affect the interpretation of large-scale structure clustering in two ways. The power spectrum shape is modified on small scales due to the extra perturbations although this effect however can be mimicked by scale-dependent bias. More importantly isocurvature modes can lead to an incorrect inferred value for the sound horizon at radiation drag from CMB data. This then predicts an incorrect location of the baryon acoustic feature. It is through this effect that Euclid BAO measurements improve constraints on isocurvature modes.

## Part IV Testing the basic cosmological hypotheses

### Introduction

The standard cosmological analyses implicitly make several assumptions, none of which are seriously challenged by current data. Nevertheless, Euclid offers the possibility of testing some of these basic hypotheses. Examples of the standard assumptions are that photon number is conserved, that the Copernican principle holds (i.e., we are not at a special place in the universe) and that the universe is homogeneous and isotropic, at least on large enough scales. These are the pillars on which standard cosmology is built, so it is important to take the opportunity offered by Euclid observations to test these basic hypotheses.

### Photon number conservation, transparency and the etherington relation

There are several examples of non-standard—but nevertheless theoretically well-motivated—physical processes in which photon number is not conserved. A non-exhaustive list includes a non perfectly transparent Universe, decaying vacuum cosmologies/photon injection mechanisms, models in which the fine-structure constant varies, physically motivated scenarios where photons mix with other particles (such as axions), modified gravity scenarios, and so on. There are two basic ways to observationally constrain these scenarios: the temperature-redshift relation and the distance duality (or Etherington) relation.

The Etherington relation (Etherington 1933) implies that, in a cosmology based on a metric theory of gravity, distance measures are unique: the luminosity distance is $(1 + z)^2$ times the angular diameter distance. This is valid in any cosmological background where photons travel on null geodesics and where, crucially, photon number is conserved.

On the other hand, if the expansion of the Universe is adiabatic and the CMB spectrum was a black-body at the time it originated, this shape will be preserved with its temperature evolving as $T(z) = T_0 (1 + z)$. This is a robust prediction of standard cosmology, but it is violated in many non-standard models, including scenarios involving photon mixing/violation of photon number conservation.

An optical/IR survey like Euclid will only be able to test the distance duality relation. However, we argue here, complementary information from the CMB temperature-redshift relation, which will be available by the year 2020, can greatly enhance the interpretation of Euclid results.

Specific scenarios in which the Etherington relation would be violated include deviations from a metric theory of gravity, photons not travelling along unique null geodesics, variations of fundamental constants, etc. Following Avgoustidis et al. (2009, 2010) generic deviations from the Etherington relation can be parametrized as:

IV.2.1 $$\begin{aligned} D_L(z) = D_A(z)(1 + z)^{2+\epsilon }, \end{aligned}$$

with current constraints being at the few percent level (Avgoustidis et al. 2010). Similarly, deviations from the standard evolution of the CMB temperature with redshift can be parametrized phenomenologically by Avgoustidis et al. (2012)

IV.2.2 $$\begin{aligned} T(z) = T_0 (1 + z)^{1-\beta }. \end{aligned}$$

At low redshifts (say $z < 1$), the CMB temperature can be measured via the Sunyaev–Zel’dovich effect towards galaxy clusters (Luzzi et al. 2009), while at higher redshifts ($z > 1$) it can be obtained from high-resolution absorption spectroscopy of atomic, ionic or molecular levels excited by the photon absorption of the CMB radiation (Noterdaeme et al. 2011). Up to redshifts $z\sim 3$, current direct constraints on the parameter $\beta$ are at the few percent level (Noterdaeme et al. 2011), while combining all currently available direct and indirect *T*(*z*) measurements yields a sub-percent constraint on $\beta$ (Avgoustidis et al. 2016).

Therefore, in models where photon number is not conserved, both the temperature-redshift relation and the Etherington relation will be violated, providing two independent observables. Crucially, in a broad range of models the two violations are not independent: for example assuming that deviations from the standard behaviour only depend on redshift (but not on frequency) one can show (Avgoustidis et al. 2012) that if

IV.2.3 $$\begin{aligned} T(z) = T_0 (1 + z) f(z) \end{aligned}$$

then

IV.2.4 $$\begin{aligned} D_L(z) = D_A(z)(1 + z)^2 f(z)^{3/2}. \end{aligned}$$

In particular, for the two simple parametrizations above, $\epsilon = -\, 3\beta /2$.

Therefore, distance duality data can be used to constrain $\beta$ (i.e., deviations from the standard CMB temperature evolution), leading to a 40% improvement in existing constraints, as discussed in Avgoustidis et al. (2012).

#### Transparency

A change in the photon flux during propagation towards the Earth will affect the supernovae (SNe) luminosity distance measures $D_L(z)$ but not the determinations of the angular diameter distance. BAO will not be affected so $D_A(z)$ and *H*(*z*) measurements from BAO could be combined with supernovae measurements of $D_L(z)$ to constrain deviations from photon number conservation. Photon conservation can be violated by simple astrophysical effects or by exotic physics. Amongst the former we find, for instance, attenuation due to interstellar dust, gas and/or plasmas. Most known sources of attenuation are expected to be clustered and can be typically constrained down to the 0.1% level (Ménard et al. 2008; More et al. 2009).

Unclustered sources of attenuation are however much more difficult to constrain. For example, grey dust (Aguirre 1999) has been invoked to explain the observed dimming of Type Ia supernovae without resorting to cosmic acceleration. While the latter scenario has been ruled out by observations (Aguirre and Haiman 2000; Bassett and Kunz 2004b), it has been shown (Corasaniti 2006) that the effect of grey dust could cause an extinction as large as 0.08 mag at $z = 1.7$, thus potentially affecting dark energy parameter inference from future supernova surveys. More exotic sources of photon conservation violation involve a coupling of photons to particles beyond the standard model of particle physics. Such couplings would mean that, while passing through the intergalactic medium, a photon could disappear—or even (re)appear—while interacting with such exotic particles, modifying the apparent luminosity of sources. Recently, Avgoustidis et al. (2010) considered the mixing of photons with scalars, known as axion-like particles, chameleons, and the possibility of mini-charged particles which have a tiny, and unquantized electric charge. In particular, the implications of these particles on the SN luminosity have been described in a number of publications (Csáki et al. 2002; Mörtsell et al. 2002; Burrage 2008; Ahlers 2009) and a detailed discussion of the proposed approach can be found in Bassett and Kunz (2004a, b), Avgoustidis et al. (2009, 2010).

Any systematic violations in photon conservation can then be interpreted as an opacity effect in the observed luminosity distance, parametrized through a generic opacity parameter, $\tau (z)$, as:

IV.2.5 $$\begin{aligned} D_{L,\mathrm {obs}}^2=D^2_{L,\mathrm {true}}\exp [\tau (z)]. \end{aligned}$$

Note that a negative $\tau (z)$ allows for apparent brightening of light sources, as would be the case, for example, if exotic particles were also emitted from the source and converted into photons along the line of sight (Burrage 2008). For specific models of exotic matter-photon coupling, such as axion-like particles (ALPs), chameleons, and mini-charged particles (MCPs), the appropriate parametrization of $\tau (z)$ can be used (Avgoustidis et al. 2010). In order to discuss generic forecasts for Euclid, we return to the parametrization of Avgoustidis et al. (2009), Eq. (IV.2.1) above (Fig. 53).

**Fig. 54:**
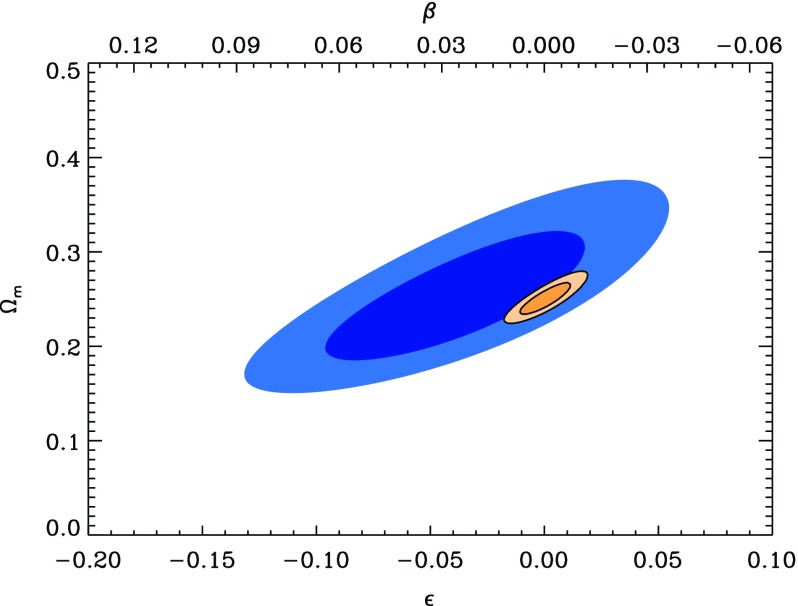
Constraints on possible violation of the Etherington relation in the form of deviations from a perfectly transparent universe ($\epsilon =0$). The corresponding constraint on the parameter $\beta$, quantifying violations from the standard temperature-redshift relation, can be read in the upper x-axis. Blue regions represent current constraints while orange are forecast Euclid constraints assuming it is accompanied by a Dark Energy Task Force stage IV supernovae sample. Image reproduced with permission from Avgoustidis et al. (2010), copyright by IOP

Forecast Euclid constraints are shown in Fig. 54, taken from Avgoustidis et al. (2010), and adapted to also show the corresponding constraint on the temperature-redshift violation discussed in Avgoustidis et al. (2012). The lower x-axis displays the parameter $\epsilon$ of Eq. (IV.2.1), while the upper x-axis shows parameter $\beta$ of Eq. (IV.2.2), assuming the linear relation $\epsilon = -\, 3\beta /2$. Current constraints are shown in blue, while the orange contours represent the expected improvement from Euclid, assuming it is accompanied by a supernova sample with the characteristic of a Dark Energy Task Force stage IV survey.

**Fig. 55:**
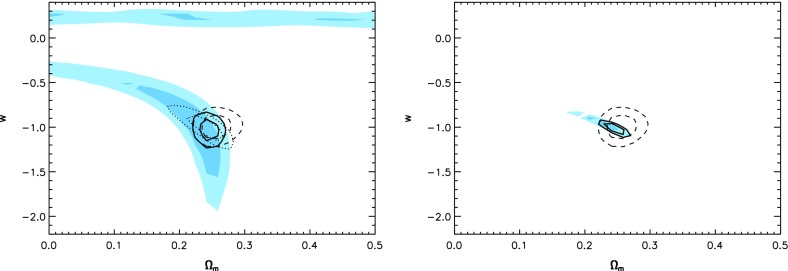
Forecasted 68 and 95% likelihood contours for SN (filled blue), H(z) (dashed line transparent) and combined SN+H(z) (solid line transparent), assuming Euclid BAO is accompanied by a SNAP-like (or Dark Energy Task Force stage IV) supernova sample. We show constraints on the $\varOmega _m-w$ plane, having marginalised over all other parameters in the context of flat wCDM models. On the left, we have allowed a coupling between photons and a putative dark energy scalar at the level allowed by current data, while on the right we have set this coupling to zero. The dotted contours on the left show SN contours assuming constraints on this coupling can be improved by an order of magnitude. Note how the joint contours become dominated by the SN data if this coupling is strongly constrained

If considering Euclid data in combination with SN brightness measurements, allowing for the coupling of scalar fields to photons will drastically weaken constraints on cosmological parameters. A remedy is to independently constrain photon number non-conservation arising from the possible coupling of photons to a putative dark energy/modified gravity scalar field. As shown in Avgoustidis et al. (2014) and illustrated in Fig. 55, improved direct measurements of the CMB temperature at different redshifts (such as those expected in the coming years from ALMA and ESPRESSO, and ultimately ELT-HIRES) can be used in combination with distance measures and SN data to break parameter degeneracies and significantly improve constraints on physical processes in the early universe. Note in particular that the combined BAO+SN constraint is dominated by the BAO data if the coupling between photons and dark energy is included at the currently allowed level, but gets dominated by SN (and the combined constraint improves dramatically) if constraints on this coupling improve by more than one order of magnitude.

In these forecasts we are assuming that the SN data will come from a SNAP-like or other DETF IV dataset. However, the need for this external dataset may be alleviated if Euclid carries out its own SN survey; this scenario is also discussed in Avgoustidis et al. (2014).

#### Axion-like particles

Axion-like particles (ALP) can arise from field theoretic extensions of the standard model as Goldstone bosons when a global shift symmetry, present in the high energy sector, is spontaneously broken. Interestingly, these fields also arise naturally in string theory (for a review see Svrcek and Witten 2006). Chameleon scalar fields are another very interesting type of ALPs (Brax et al. 2010b). They were originally invoked to explain the current accelerated expansion of the universe with a quintessence field which can couple to matter without giving rise to large fifth forces or unacceptable violations of the weak equivalence principle. A chameleon model with only matter couplings will induce a coupling to photons.

**Fig. 56:**
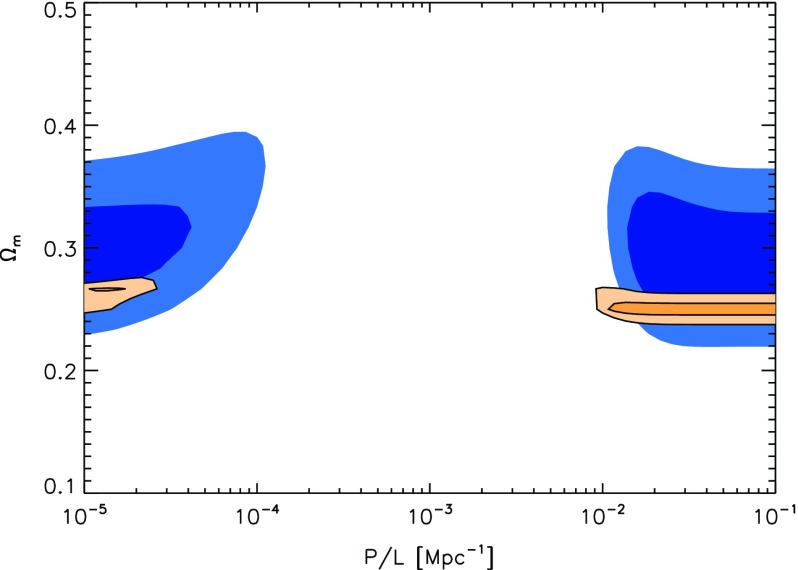
Constraints on the simplest Axion-like particles models. Blue regions represent current constraints while orange are forecast Euclid constraints assuming it is accompanied by a Dark Energy Task Force stage IV supernovae sample. Here *P* / *L* is the conversion probability per unit length and is the relevant parameter for $\tau (z)$. Image reproduced with permission from Avgoustidis et al. (2010), copyright by IOP

The presence of ALPs will have an impact on observations of SNe if their observed light passes through (intergalactic) magnetic fields. The net effect depends on the ratio of the transition probability to the length travelled through a magnetic field, and a parameter *A* describing the degree of thermalization of the initial flux ($A=1$ means thermalized flux where the photon to ALP transition is compensated by the inverse ALP to photon, making the photon number constant). For the simplest ALP model $A=2/3$, the present and forecast constraints are shown in Fig. 56, reproduced from Avgoustidis et al. (2010).

#### Mini-charged particles

New particles with a small unquantized charge have been investigated in several extensions of the standard model (Holdom 1986; Batell and Gherghetta 2006). In particular, they arise naturally in extensions of the standard model which contain at least one additional U(1) hidden sector gauge group (Holdom 1986; Brümmer et al. 2009). The gauge boson of this additional U(1) is known as a hidden photon, and hidden sector particles, charged under the hidden U(1), get an induced electric charge proportional to the small mixing angle between the kinetic terms of the two photons. In string theory, such hidden U(1)s and the required kinetic mixing are a generic feature (Abel et al. 2008b, a; Dienes et al. 1997; Abel and Schofield 2004; Goodsell et al. 2009). Hidden photons are not necessary however to explain mini-charged particles, and explicit brane-world scenarios have been constructed (Batell and Gherghetta 2006) where MCPs arise without the need for hidden photons.

**Fig. 57:**
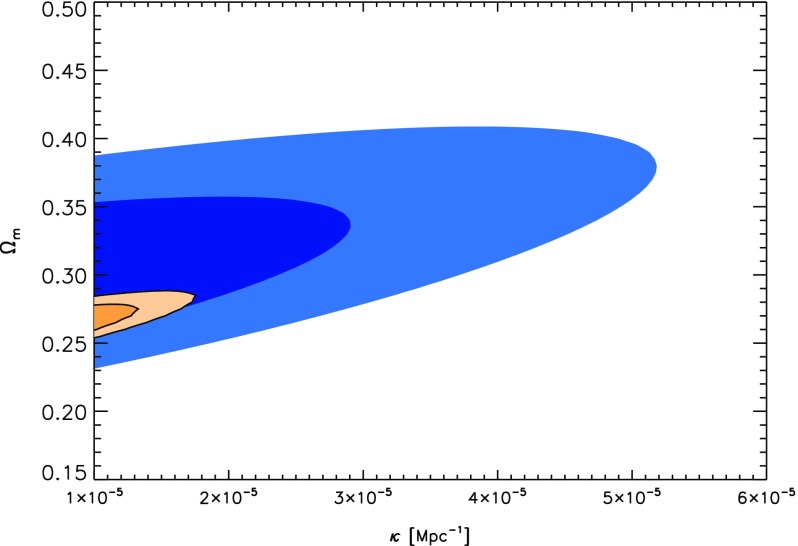
Constraints on MCP models. Blue regions represent current constraints while orange are forecast Euclid constraints assuming it is accompanied by a Dark Energy Task Force stage IV supernovae sample. Image reproduced with permission from Avgoustidis et al. (2010), copyright by IOP

More interestingly, Ahlers (2009), Gies et al. (2006) and Ahlers et al. (2008) pointed out that photons propagating in a background magnetic field can actually pair-produce MCPs without the need for a second photon in the initial state. The opacity in this case is parametrized by $\kappa y(z)$ where *y* is the comoving distance to the source and $\kappa$ encloses information on the MCP electric charge and the intervening magnetic field strength. Figure 57 shows current and forecast Euclid’s constraints, taken from Avgoustidis et al. (2010) assuming Euclid is accompanied by a supernova sample with the characteristic of a Dark Energy Task Force stage IV survey.

### Beyond homogeneity and isotropy

The crucial ingredient that kickstarted dark energy research was the interpretation in 1998 of standard candle observations in terms of cosmic acceleration required to explain the data in the context of the FLRW metric. What we observe is however merely that distant sources ($z>0.3$) are dimmer than we would predict in a matter-only universe calibrated through “nearby” sources. That is, we observe a different evolution of luminosity rather than directly an increase in the expansion rate. Can this be caused by a strong inhomogeneity rather than by an accelerating universe?

In addition, cosmic acceleration seems to be a recent phenomenon at least for standard dark-energy models, which gives rise to the coincidence problem. The epoch in which dark energy begins to play a role is close to the epoch in which most of the cosmic structures formed out of the slow linear gravitational growth. We are led to ask again: can the acceleration be caused by strong inhomogeneities rather than by a dark energy component?

Finally, one must notice that in all the standard treatment of dark energy one always assumes a perfectly isotropic expansion. Could it be that some of the properties of acceleration depend critically on this assumption?

In order to investigate these issues, in this section we explore radical deviations from homogeneity and isotropy and see how Euclid can test them.

#### Anisotropic models

In recent times, there has been a resurgent interest towards anisotropic cosmologies, classified in terms of Bianchi solutions to general relativity. This has been mainly motivated by hints of anomalies in the cosmic microwave background (CMB) distribution observed on the full sky by the WMAP satellite (de Oliveira-Costa et al. 2004; Vielva et al. 2004; Cruz et al. 2005; Eriksen et al. 2004a) and Planck satellite (Planck Collaboration 2014d, e, f, g).

While the CMB is very well described as a highly isotropic (in a statistical sense) Gaussian random field, and the anomalies are a posteriori statistics and therefore their statistical significance should be corrected at least for the so-called *look elsewhere effect* (see, e.g., Pontzen and Peiris 2010; Bennett et al. 2011 and references therein), recent analyses have shown that local deviations from Gaussianity in some directions (the so called cold spots, see Cruz et al. 2005; Planck Collaboration 2014d) cannot be excluded at high confidence levels. Furthermore, the CMB angular power spectrum extracted from the WMAP and Planck maps has shown in the past a quadrupole power lower than expected from the best-fit cosmological model (Efstathiou 2004; Planck Collaboration 2014a). Several explanations for this anomaly have been proposed (see, e.g., Tsujikawa et al. 2003; Cline et al. 2003; DeDeo et al. 2003; Campanelli et al. 2007; Gruppuso 2007) including the fact that the universe is expanding with different velocities along different directions. While deviations from homogeneity and isotropy are constrained to be very small from cosmological observations, these usually assume the non-existence of anisotropic sources in the late universe. Conversely, as suggested in Koivisto and Mota (2008a, b), Battye and Moss (2006), Chimento and Forte (2006) and Cooray et al. (2010), dark energy with anisotropic pressure acts as a late-time source of anisotropy. Even if one considers no anisotropic pressure fields, small departures from isotropy cannot be excluded, and it is interesting to devise possible strategies to detect them.

The effect of assuming an anisotropic cosmological model on the CMB pattern has been studied by Collins and Hawking (1973), Barrow et al. (1985), Martinez-González and Sanz (1995), Maartens et al. (1996), Bunn et al. (1996) and Kogut et al. (1997). The Bianchi solutions describing the anisotropic line element were treated as small perturbations to a Friedmann–Robertson–Walker (FRW) background. Such early studies did not consider the possible presence of a non-null cosmological constant or dark energy and were upgraded updated recently by McEwen et al. (2006) and Jaffe et al. (2006).

**Table 25:** Bianchi models containing FRW limit and their structure constants

Type	*a*	$n_1$	$n_2$	$n_3$
I	0	0	0	0
V	1	0	0	0
$\hbox {VII}_{0}$	0	0	1	1
$\hbox {VII}_h$	$\sqrt{h}$	0	1	1
IX	0	1	1	1

One difficulty with the anisotropic models that have been shown to fit the large-scale CMB pattern, is that they have to be produced according to very unrealistic choices of the cosmological parameters. For example, the Bianchi VIIh template used in Jaffe et al. (2006) requires an open universe, a hypothesis which is excluded by most cosmological observations.

Bianchi models are described by homogeneous and anisotropic metrics. If anisotropy is slight, the dynamics of any Bianchi model can be decomposed into an isotropic FRW background linearly perturbed to break isotropy; on the other side, homogeneity is maintained with respect to three Killing vector fields. Anisotropy can also be studied by means of inhomogeneous models such as the Szekeres metric. In particular, the quasi-spherical Szekeres models feature a mass-density distribution which can be interpreted as a superposition of a mass monopole and a mass dipole (see Bolejko et al. 2011 and references therein), thus generalizing the case of the isotropic LTB metric (see Sect. IV.3.2 below).

The geometry of Bianchi models is set up by the structure constants $C^k_{ij}$, defined by the commutators of (these) three Killing fields $\varvec{\xi }_i$:

IV.3.1 $$\begin{aligned} \left[ \varvec{\xi }_i, \varvec{\xi }_j \right] = C^k_{ij} \varvec{\xi }_k. \end{aligned}$$

The structure constants are subject to the antisymmetry relation $C^k_{ij} = -\, C^k_{ji}$ and the Jacobi identities $C^a_{[bc} C^d_{e]a}=0$. As a consequence, their attainable values are restricted to only four of the initial 27 necessary to describe a given space. In Ellis and MacCallum (1969), these four values are dubbed as $n_1, n_2, n_3$ and $a_1$ $n_1, n_2, n_3$ and *a*. The categorization of Bianchi models into different types relies on classifying the inequivalent sets of these four constants. In Table 25 the subclass of interest containing the FRW limit is shown. Bianchi types VIIh and IX contain the open and closed FRW model, respectively. Type $\hbox {VII}_{0}$ contains the flat FRW; types I and V are just particular subcases of the $\hbox {VII}_{0}$ and VIIh. In type I no vertical motions are allowed and the only extension with respect to the FRW case is that there are three different scale factors. The metric in general can be written as

IV.3.2 $$\begin{aligned} g_{\mu \nu }=-n_{\mu }n_{\nu }+g_{ab}\xi _{\mu }^{a}\xi _{\nu }^{b}, \end{aligned}$$

where $g_{ab}$ is a $3\times 3$ metric depending on *t*. It can be decomposed as $g_{ab}=e^{2\alpha } [e^{2\beta }]_{ab}$, where the first term represents the volumetric expansion and the second term includes the anisotropy.

*IV.3.1.1 Late-time anisotropy*

While deviations from homogeneity and isotropy are constrained to be very small from cosmological observations, these usually assume the non-existence of anisotropic sources in the late universe. The CMB provides very tight constraints on Bianchi models at the time of recombination (Bunn et al. 1996; Kogut et al. 1997; Martinez-González and Sanz 1995) of order of the quadrupole value, i.e., $\sim \,10^{-5}$. Usually, in standard cosmologies with a cosmological constant the anisotropy parameters scale as the inverse of the comoving volume. This implies an isotropization of the expansion from the recombination up to the present, leading to the typically derived constraints on the shear today, namely $\sim \,10^{-9}\div 10^{-10}$. However, this is only true if the anisotropic expansion is not generated by any anisotropic source arising after decoupling, e.g., vector fields representing anisotropic dark energy (Koivisto and Mota 2008a).

An additional problem is that an inflationary phase—required to explain a number of features of the standard cosmological model—isotropizes the universe very efficiently, leaving a residual anisotropy that is negligible for any practical application. These difficulties vanish if an anisotropic expansion takes place only well after the decoupling between matter and radiation, for example at the time of dark energy domination (Koivisto and Mota 2008a, b; Battye and Moss 2006; Chimento and Forte 2006; Cooray et al. 2010). In these references, it is proposed that dark energy with anisotropic pressure acts as a late-time source of anisotropy.

It would be great if one could measure deviations from isotropic expansion. For example, the effect of cosmic parallax (Quercellini et al. 2009) has been recently proposed as a tool to assess the presence of an anisotropic expansion of the universe. It is essentially the change in angular separation in the sky between far-off sources, due to an anisotropic expansion.

A common parameterization of an anisotropically distributed dark energy component is studied in a class of Bianchi I type, where the line element is

IV.3.3 $$\begin{aligned} \mathrm {d}s^{2} = -\,\mathrm {d}t^{2}+a^{2}(t) \, \mathrm {d}x^{2}+b^{2}(t) \, \mathrm {d}y^{2}+c^{2}(t) \, \mathrm {d}z^{2}. \end{aligned}$$

The expansion rates in the three Cartesian directions *x*, *y* and *z* are defined as $H_{X}=\dot{a}/a$, $H_{Y}=\dot{b}/b$ and $H_{Z}=\dot{c}/c$, where the dot denotes the derivative with respect to coordinate time. In these models they differ from each other, but in the limit of $H_{X}=H_{Y}=H_{Z}$ the flat FRW isotropic expansion is recovered. Among the Bianchi classification models the type I exhibits flat geometry and no overall vorticity; conversely, shear components $\varSigma _{X,Y,Z}=H_{X,Y,Z}/H-1$ are naturally generated, where *H* is the expansion rate of the average scale factor, related to the volume expansion as $H=\dot{A}/A$ with $A=(abc)^{1/3}$.

The anisotropic expansion is caused by the anisotropically stressed dark energy fluid whenever its energy density contributes to the global energy budget. If the major contributions to the overall budget come from matter and dark energy, as after recombination, their energy–momentum tensor can be parametrized as:

IV.3.4 $$\begin{aligned} T_{(m)\nu }^{\mu }= & {} \text{ diag }\left( -\,1,w_{m},w_{m},w_{m}\right) \rho _{m} \end{aligned}$$

IV.3.5 $$\begin{aligned} T_{(\mathrm {DE})\nu }^{\mu }= & {} \text{ diag }\left( -\,1,w,w+3\delta ,w+3\gamma \right) \rho _{\mathrm {DE}}, \end{aligned}$$

respectively, where $w_{m}$ and *w* are the equation of state parameters of matter and dark energy and the skewness parameters $\delta$ and $\gamma$ can be interpreted as the difference of pressure along the *x* and *y* and *z* axis. Note that the energy–momentum tensor (IV.3.5) is the most general one compatible with the metric (IV.3.3) (Koivisto and Mota 2008a). Two quantities are introduced to define the degree of anisotropic expansion:

IV.3.6 $$\begin{aligned} \begin{aligned} R&\,\equiv \,(\dot{a}/a-\dot{b}/b)/H\;=\;\varSigma _{x}-\varSigma _{y},\\ S&\,\equiv \,(\dot{a}/a-\dot{c}/c)/H\;=\;2\varSigma _{x}+\varSigma _{y}. \end{aligned} \end{aligned}$$

Considering the generalized Friedmann equation, the continuity equations for matter and dark energy and no coupling between the two fluids, the derived autonomous system reads (Koivisto and Mota 2008a, b):

IV.3.7 $$\begin{aligned} \begin{aligned} U'&= U(U-1)[\gamma (3+R-2S)\;+\,\delta (3-2R+S)\,+\,3(w-w_{m})]\\ S'&= \frac{1}{6}(9-R^{2}+RS-S^{2})\big \{S[U(\delta +\gamma +w-w_{m})+w_{m}-1]-6\, \gamma \, U\big \}\\ R'&= \frac{1}{6}(9-R^{2}+RS-S^{2})\big \{R[U(\delta +\gamma +w-w_{m})+w_{m}-1]-6\, \delta \, U\big \}, \end{aligned} \end{aligned}$$

where $U\equiv \rho _{\mathrm {DE}}/(\rho _{\mathrm {DE}}+\rho _{m})$ and the derivatives are taken with respect to $\log (A)/3$. System (IV.3.7) exhibits many different fixed points, defined as the solutions of the system $S'=R'=U'=0$. Beside the Einstein–de Sitter case ($R_{*}=S_{*}=U_{*}=0$), the most physically interesting for our purposes are the dark energy dominated solution

IV.3.8 $$\begin{aligned} R_{*} = \frac{6\delta }{\delta +\gamma +w-1}, \quad S_{*} = \frac{6\gamma }{\delta +\gamma +w-1}, \quad U_{*} = 1, \end{aligned}$$

and the scaling solution

IV.3.9 $$\begin{aligned} \begin{aligned} R_{*}&= \frac{3\delta (\delta +\gamma +w)}{2(\delta ^{2}-\delta \gamma +\gamma ^{2})} ,\quad S_{*} = \frac{3\gamma (\delta +\gamma +w)}{2(\delta ^{2}-\delta \gamma +\gamma ^{2})},\\ U_{*}&= \frac{w+\gamma +\delta }{w^{2}-3(\gamma -\delta )^{2}+2w(\gamma +\delta )} , \end{aligned} \end{aligned}$$

in which $\rho _{\mathrm {DE}}/\rho _{m}={\mathrm {const.}}$, i.e., the fractional dark energy contribution to the total energy density is constant.

Anisotropic distribution of sources in Euclid survey might constrain the anisotropy at present, when the dark energy density is of order 74%, hence not yet in the final dark energy dominant attractor phase (IV.3.8).

*IV.3.1.2 Early-time anisotropy*

An alternative (and, arguably, more speculative) possibility is that anisotropy may be generated by the presence of anisotropic fields at inflation. Such fields could be spinors, vectors or higher order forms which modify the properties of fluctuations in a direction-dependent way, either directly through perturbation dynamics or by causing the background to inflate slightly anisotropically. The most common alternative is vector fields (which will be further discussed in Sect. IV.4.2).

Whereas a canonical scalar field easily inflates the universe if suitable initial conditions are chosen, it turns out that it is much less straightforward to construct vector field alternatives. In particular, one must maintain a sufficient level of isotropy of the universe, achieve slow roll and keep perturbations stable. Approaches to deal with the anisotropy have been based on a “triad” of three identical vectors aligned with the three axis (Armendariz-Picon 2004), a large number of randomly oriented fields averaging to isotropy (Golovnev et al. 2008), time-like (Koivisto and Mota 2008c) or sub-dominant (Dimopoulos and Karčiauskas 2008) fields. There are many variations of inflationary scenarios involving vector fields, and in several cases the predictions of the primordial spectra of perturbations have been worked out in detail, see e.g., Watanabe et al. (2010). The generic prediction is that the primordial perturbation spectra become statistically anisotropic, see e.g., Ackerman et al. (2007).

Anisotropy could be also regarded simply as a trace of the initial conditions set before inflation. One then assumes that inflation has lasted just about the 60 e-folds so that the largest observable scales were not yet smoothed out, or isotropized, by the early inflationary expansion (Pitrou et al. 2008). Such a scenario can also be linked to various speculative ideas of pre-inflationary physics such as gravitational tunnelling into an anisotropic universe, see e.g., Adamek et al. (2010).

Also in this case the interest in such possibilities has been stimulated by several anomalies observed in the temperature WMAP maps, see Copi et al. (2010) for a recent review (some of them were also present in the COBE maps). Their statistical evidence is quite robust w.r.t. the increase of the signal-to-noise ratio over the years of the WMAP mission and to independent tests by the international scientific community, although the a posteriori choice of statistics could make their interpretation difficult, see Bennett et al. (2011). Apart from those already mentioned in Sect. IV.3.1, these anomalies include an alignment between the harmonic quadrupole and octupole modes in the temperature anisotropies (de Oliveira-Costa et al. 2004), an asymmetric distribution of CMB power between two hemispheres, or dipole asymmetry (Eriksen et al. 2004b), the lack of power of the temperature two-point correlation function on large angular scales ($>\,60^{\circ }$), asymmetries in the even versus odd multipoles of the CMB power spectra (parity symmetry breaking), both at large (Kim and Naselsky 2010; Gruppuso et al. 2011) and intermediate angular scales (Bennett et al. 2011). Some of the anomalies could be connected among each other, e.g., the CMB parity breaking has been recently linked to the lack of large-scale power (Maris et al. 2011; Copi et al. 2007; Kim and Naselsky 2011).

#### Inhomogeneous models

Nonlinear inhomogeneous models are traditionally studied either with higher-order perturbation theory or with *N*-body LSS simulation codes. Both approaches have their limits. A perturbation expansion obviously breaks down when the perturbations are deeply in the nonlinear regime. *N*-body codes, on the other hand, are intrinsically Newtonian and, at the moment, are unable to take into account full relativistic effects, although there are recent attempts to address these issues, see Adamek et al. (2013).

Nevertheless, *N*-body codes can still account for the general relativistic behavior of gravitational collapse in the specific case of inhomogeneous spherically symmetric models, as shown recently in Alonso et al. (2010), where the growth of a void follows the full nonlinear GR solution down to large density contrasts (of order one). A possibility to make progress is, therefore, to proceed with the most extreme simplification: spherical symmetry. By assuming that the inhomogeneity is radial the general relativistic equations can be solved exactly and the suite of available *N*-body techniques can be applied, and one can make definite observable predictions both at the background and perturbation level.

It is important to stress that the fact that the model is spherically symmetric does not necessarily mean that the observer is at the center of a large void or super structure, in gross violation of the Copernican principle. The universe is indeed inhomogeneous and a spherically symmetric description of the density field is legitimate if the observer averages over angles. Clearly, which picture is valid is determined by the parameters used to model the inhomogeneity. If the inhomogeneity is standard—i.e. stemming from standard perturbations—then the latter Copernican view is applicable. However, if the inhomogeneity is unconstrained, then the non-Copernican view is the only viable.

Historically, large-void models have been investigated as an alternative to the dark-energy scenario. Indeed, a void creates an apparent acceleration field that can in principle match any supernova observations (see the review article by Marra and Notari 2011 and references therein). This effect is easy to understand: standard candles are confined to the light cone and hence temporal changes can be replaced by spatial changes along photon geodesics. In this case, “faster expansion now than before” is simply replaced by “faster expansion here than there”. This is why a void model can mimic the effect of dark energy if it extends to the point in spacetime where the dark energy becomes subdominant: a typical scenario that can mimic the late-time acceleration of the concordance $\varLambda$CDM model consists of a deep void extending for 1–3 Gpc, corresponding to a transition redshift $z_{e}$ (i.e., the void edge) of about 0.3–1. Of course, these models strongly violate the Copernican principle (CP) as the observer needs to be confined within few tens of Mpc from the center of a Gpc-scale void in order not to predict a dipole anisotropy that it is not observed in the CM (Marra and Notari 2011).

The consistent way to realize such a spherical inhomogeneity has been studied since the 1930s in the relativistic literature: the Lemaître–Tolman–Bondi (LTB) metric. This is the generalization of an FLRW metric in which the expansion factor along the radial coordinate *r* is different relative to the surface line element $\mathrm {d}\varOmega ^{2}=\mathrm {d}\theta ^{2}+\sin ^{2}\theta \,\mathrm {d}\phi ^{2}$. If we assume the inhomogeneous metric (this subsection follows closely the treatment in Enqvist and Mattsson (2007) and Amendola and Tsujikawa (2010), see (Marra and Paakkonen 2012, “Appendix B” section) for an alternative approach)

IV.3.10 $$\begin{aligned} \mathrm {d}s^{2}=-\mathrm {d}t^{2}+X^{2}(t,r)\,\mathrm {d}r^{2} +R^{2}(t,r)\,\mathrm {d}\varOmega ^{2}, \end{aligned}$$

and solve the (0, 1) Einstein equation for a fluid at rest we find that the LTB metric is given by

IV.3.11 $$\begin{aligned} \mathrm {d}s^{2}=-\mathrm {d}t^{2}+\frac{\left[ R'(t,r)\right] ^{2}}{1+\beta (r)}\mathrm {d}r^{2}+R^{2}(t,r)\mathrm {d}\varOmega ^{2}, \end{aligned}$$

where $R(t,r),\beta (r)$ are arbitrary functions. Here primes and dots refer to partial space and time derivatives, respectively. The function $\beta (r)$ can be thought of as a position-dependent spatial curvature. If *R* is factorized so that $R(t,r)=a(t)f(r)$ and $\beta (r)=-Kf^{2}(r)$, then we recover the FLRW metric (up to a redefinition of *r*: from now on when we seek the FLRW limit we put $R=a(t)r$ and $\beta =-Kr^{2}$). Otherwise, we have a metric representing a spherical inhomogeneity centered on the origin. An observer located at the origin will observe an isotropic universe. We can always redefine *r* at the present time to be $R_{0}\equiv R(t_{0},r)=r$, so that the metric is very similar to an FLRW today.

Considering the infinitesimal radial proper length $D_{||}=R'\mathrm {d}r/\sqrt{1+\beta }$, we can define the *radial* *Hubble function* as

IV.3.12 $$\begin{aligned} H_{||}\equiv \dot{D}_{||}/D_{||}=\dot{R}'/R', \end{aligned}$$

and similarly the *transverse Hubble function*:

IV.3.13 $$\begin{aligned} H_{\perp }=\dot{R}/R. \end{aligned}$$

Of course the two definitions coincide for the FLRW metric. The non-vanishing components of the Ricci tensor for the LTB metric are

IV.3.14 $$\begin{aligned}&R_{0}^{0}=\frac{2\ddot{R}}{R}+\frac{\ddot{R}'}{R'}, \end{aligned}$$

IV.3.15 $$\begin{aligned}&R_{1}^{1}= \frac{2\dot{R}\dot{R'}+R\ddot{R}'-\beta '}{RR'}, \end{aligned}$$

IV.3.16 $$\begin{aligned}&R_{2}^{2}=R_{3}^{3}= \frac{\dot{R}^{2}-\beta }{R^{2}}+ \frac{\dot{R}\dot{R}'+R'\ddot{R}-\beta '/2}{RR'}. \end{aligned}$$

In terms of the two Hubble functions, we find that the Friedmann equations are given by

IV.3.17 $$\begin{aligned} H_{\perp }^{2}+2H_{||}H_{\perp }-\frac{\beta }{R^{2}}-\frac{\beta '}{RR'}= & {} 8\pi G(\rho _{m}+\rho _{\varLambda }), \end{aligned}$$

IV.3.18 $$\begin{aligned} 6\frac{\ddot{R}}{R}+2H_{\perp }^{2}-2\frac{\beta }{R^{2}}-2H_{||}H_{\perp }+\frac{ \beta '}{RR'}= & {} -8\pi G (\rho _{m} - 2 \rho _{\varLambda }), \end{aligned}$$

where $\rho _{m}(t,r)$ is the pressureless matter density and $\rho _{\varLambda }= \varLambda /8\pi G$ is the energy density associated with a cosmological constant. Adding Eqs. (IV.3.17) and (IV.3.18), it follows that $2R\ddot{R}+\dot{R}^{2}=\beta +\varLambda R^2$. Integrating this equation, we obtain a Friedmann-like equation

IV.3.19 $$\begin{aligned} H_{\perp }^{2}=\frac{\alpha (r)}{R^{3}} + \frac{8\pi G}{3}\rho _{\varLambda } +\frac{\beta (r)}{R^{2}}, \end{aligned}$$

where $\alpha (r)$ is a free function that we can use along with $\beta (r)$ to describe the inhomogeneity. Using Eq. (IV.3.19) we can define the effective density parameters $\varOmega _{i}^{(0)}(r)=\varOmega _{i}(r,t_{0})$ today:

IV.3.20 $$\begin{aligned} \varOmega _{m}^{(0)}(r)\equiv & {} \frac{\alpha (r)}{R_{0}^{3}H_{\perp 0}^{2}}, \end{aligned}$$

IV.3.21 $$\begin{aligned} \varOmega _{\varLambda }^{(0)}(r)\equiv & {} \frac{8\pi G}{3 H_{\perp 0}^{2}}\rho _{\varLambda }, \end{aligned}$$

IV.3.22 $$\begin{aligned} \varOmega _{K}^{(0)}(r)= & {} 1-\varOmega _{m}^{(0)}(r)-\varOmega _{\varLambda }^{(0)}(r)=\frac{\beta (r)}{R_{0}^{2}H_{\perp 0}^{2}}. \end{aligned}$$

where $R_{0}\equiv R(r,t_{0})=r$ and $H_{\perp 0}\equiv H_{\perp }(r,t_{0})$ (the superscript (0) denotes the present value). Hence, we see that the initial condition at some time $t_{0}$ (which here we take as the present time) must specify two free functions of *r*, for instance $\alpha (r),\beta (r)$ or $\varOmega _{m}^{(0)}(r),H_{\perp 0}^{}(r)$. The latter choice shows that the inhomogeneity can be in the matter distribution or in the expansion rate or in both. This freedom can be used to fit simultaneously for any expansion rate (and therefore luminosity and angular diameter distances and for any source number density (Mustapha et al. 1997).

If one imposes the additional constraint that the age of the universe is the same for every observer, then only one free function is left (García-Bellido and Haugbølle 2008a). The same occurs if one chooses $\varOmega _{m}^{(0)}(r)= {\mathrm {constant}}$ (notice that this is different from $\rho _{m}^{(0)}(r)={\mathrm {constant}}$, which is another possible choice), i.e., if the matter density fraction is assumed homogeneous today (and only today) (Enqvist 2008). The choice of a homogeneous universe age guarantees against the existence of diverging inhomogeneities in the past. In particular, a simultaneous big bang excludes decaying modes which would be strongly in contradiction with the inflationary paradigm (Zibin 2008).

In the case of zero cosmological constant, Eq. (IV.3.19) is a classical cycloid equation whose solution for $\beta >0$ is given parametrically by

IV.3.23 $$\begin{aligned} R(r,\eta )=\,\frac{\alpha (r)}{2\beta (r)}&(\cosh \eta -1)=\frac{R_{0}\varOmega _{m}^{(0)}(r)}{2[1-\varOmega _{m}^{(0)}(r)]} (\cosh \eta -1), \end{aligned}$$

IV.3.24 $$\begin{aligned} t(r,\eta )-t_{B}(r)=\,&\frac{\alpha (r)}{2\beta ^{3/2}(r)}(\sinh \eta -\eta )=\frac{\varOmega _{m}^{(0)}(r)}{2[ 1-\varOmega _{m}^{(0)}(r)]^{3/2}H_{\perp 0}}(\sinh \eta -\eta ), \end{aligned}$$

where $t_{B}(r)=t(r,\eta =0)$ is the inhomogeneous “big-bang” time, i.e., the time for which $\eta =0$ and $R=0$ for a point at comoving distance *r*. This can be put to zero in all generality by a redefinition of time. The “time” variable $\eta$ is defined by the relation

IV.3.25 $$\begin{aligned} \eta =\int ^{t}_0\frac{\beta (r)^{1/2}}{R(\tilde{t},r)}\mathrm {d}\tilde{t}. \end{aligned}$$

Notice that the “time” $\eta$ that corresponds to a given *t* depends on *r*; so *R*(*r*, *t*) is found by solving numerically $\eta (t,r)$ from Eq. (IV.3.24) and then substituting $R[r,\eta (r,t)]$. The present epoch $\eta _{0}(r)$ is defined by the condition $R=R_{0}$. One can derive the age of the universe $t_{\mathrm {age}}(r)=t(r,\eta _{0})-t_{B}(r)$in terms of $\varOmega _{m}^{(0)},H_{\perp 0}$. For $\beta <0$ the $\eta$ functions in Eqs. (IV.3.23, IV.3.24) become $(1-\cos \eta )$ and $(\eta -\sin \eta )$ for *R* and *t*, respectively, while for $\beta =0$ they are $\eta ^{2}/2$ and $\eta ^{3}/6$. In the case of a nonzero cosmological constant, the differential equation of (IV.3.19) can be integrated numerically or approached semi-analytically with the method presented in Valkenburg (2012).

As anticipated, if one wants to have faster expansion inside some radius in order to mimic cosmic acceleration, one needs to impose to the solution the structure of a void. An example of the choice of $\varOmega _{m}^{(0)}(r)$ and $h^{(0)}(r)\equiv H_{\perp 0}/(100\mathrm {\ km\ s}^{-1}\mathrm {\ Mpc}^{-1}$) is (Alnes et al. 2006; García-Bellido and Haugbølle 2008a)

IV.3.26 $$\begin{aligned} \varOmega _{m}^{(0)}(r)= & {} \varOmega _{\mathrm {out}}+(\varOmega _{\mathrm {in}}-\varOmega _{\mathrm {out}})f(r,r_{0},\varDelta ), \end{aligned}$$

IV.3.27 $$\begin{aligned} h^{(0)}(r)= & {} h_{\mathrm {out}}+(h_{\mathrm {in}}-h_{\mathrm {out}})f(r,r_{0},\varDelta ), \end{aligned}$$

with

IV.3.28 $$\begin{aligned} f(r,r_{0},\varDelta )=\frac{1-\tanh [(r-r_{0})/2\varDelta ]}{1+\tanh (r_{0}/2\varDelta )}, \end{aligned}$$

representing the transition function of a shell of radius $r_{0}$ and thickness $\varDelta$. The six constants $\varOmega _{\mathrm {in}},\varOmega _{\mathrm {out}},h_{\mathrm {in}},h_{\mathrm {out}},r_{0},\varDelta$ completely fix the model. If $h_{\mathrm {in}}>h_{\mathrm {out}}$ one can mimic the accelerated expansion.

In order to compare the LTB model to observations we need to generalize two familiar concepts: redshift and luminosity distance. The redshift can be calculated through the equation

IV.3.29 $$\begin{aligned} \frac{\mathrm {d}z}{\mathrm {d}r}=(1+z)\frac{\dot{R}'}{\sqrt{1+\beta }}, \end{aligned}$$

where we must impose $z(r=0)=0$ and *R*(*t*, *r*) must be calculated on the radial geodesic $t_{p}(r)$:

IV.3.30 $$\begin{aligned} \frac{\mathrm {d}t_p}{\mathrm {d}r}=-\frac{R'(t_p(r),r)}{\sqrt{1+\beta (r)}}. \end{aligned}$$

Every LTB function, e.g., $H_{\perp }(t,r),R(t,r)$ etc., can be converted into a line-of-sight function of redshift by evaluating the arguments $r_{p}(z),t_{p}(z)$ along the past light cone.

The proper area of an infinitesimal surface at $r,t={\mathrm {constant}}$ is given by $A=R^{2}(r,t)\sin \theta \, \mathrm {d}\theta \,\mathrm {d}\phi$. The angular diameter distance is the square root of $A/(\sin \theta \,\mathrm {d}\theta \,\mathrm {d}\phi )$ so that $d_{A}(z)=R(t_{p}(z),r_{p}(z))$. Since the Etherington duality relation $d_{L}=(1+z)^{2}d_{A}$ remains valid in inhomogeneous models, we have (Kristian and Sachs 1966)

IV.3.31 $$\begin{aligned} d_{L}(z)=(1+z)^{2}R(t_{p}(z),r_{p}(z)). \end{aligned}$$

This clearly reduces to $d_{L}=(1+z)r(z)$ in the FLRW background. Armed with these observational tools, we can compare any LTB model to the observations.

Predictions of the $\varLambda$LTB model can be obtained using the packages ColLambda^27^ (Valkenburg 2012) and VoidDistancesII^28^ (Marra et al. 2013b). The latter acts as a wrapper around camb (Lewis et al. 2000a), necessitating no changes to camb’s source code and minimal changes to CosmoMC’s source code.

*IV.3.2.1 Void models as alternative to dark energy*

As said earlier, the LTB model can potentially fit distance-redshift relations such as the SN Ia Hubble diagram, the CMB power spectrum and BAO scale (Moss et al. 2011; Biswas et al. 2010). However, LTB models as alternative to dark energy (without a cosmological constant) have been ruled out by basically two effects that involve the velocity field caused by the inhomogeneity: Compton y-distortion and kSZ effect.

Off-center observers see a large dipole in the CMB in an inhomogeneous universe with simultaneous big bang, which affects the observed CMB through the kinetic Sunyaev–Zel’dovich (kSZ) effect (García-Bellido and Haugbølle 2008b): hot electrons inside an overdensity distort the CMB spectrum through inverse Compton scattering, in which low energy CMB photons receive energy boosts during collisions with the high-energy electrons. By considering the ‘linear kSZ effect’ (Zhang and Stebbins 2011; Zibin and Moss 2011), in which the effect due to all free electrons in the reionized universe is taken into account, it has been shown that void models able to mimic acceleration predict a kSZ signal by far larger than the upper limits from SPT (Shirokoff et al. 2011) and ACT (Das et al. 2011a) at $\ell =3000$. Consequently, void models with homogeneous big bang are ruled out.

On the other hand, if the big bang is not homogeneous (hypothesis which is, as said earlier, strongly in contradiction with the inflationary paradigm), it may be possible to reduce the kSZ signal so as to satisfy the experimental limits. However, in this case, the CMB undergoes large distortions because CMB photons are scattered from inside our past light-cone into our line-of-sight by off-centre reionized structures which act as mirrors. The spectrum observed by the central observer is then a mixture of blackbody spectra with different temperatures, producing a distorted blackbody spectrum. It has been shown (Caldwell and Stebbins 2008; Zibin 2011) that such models generate a Compton y-distortion which exceeds the limits placed by the COBE satellite (Fixsen et al. 1996).

*IV.3.2.2 Late-time inhomogeneity*

**Fig. 58:**
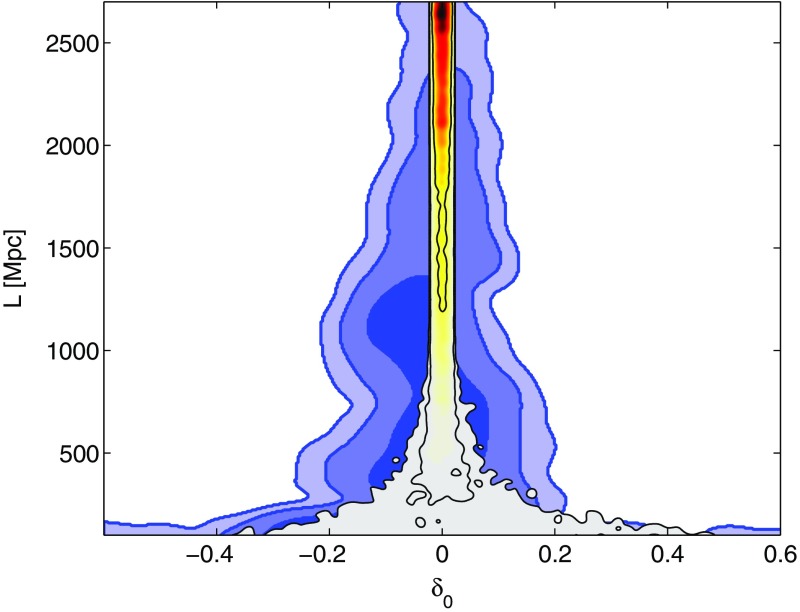
Marginalized constraints on the amount of radial inhomogeneity $\delta _0$ at a given scale *L* from local Hubble parameter measurements, supernova Ia data, CMB anisotropies, BAO observations, Compton y-distortion and kSZ constraints and age data (as lower bounds only) at 68, 95 and 99% confidence level (blue contours), compared to the expected level of inhomogeneity within a $\varLambda$CDM model that satisfies the Copernican principle (red-to-gray contours). Image reproduced with permission from Valkenburg et al. (2013b), copyright by the authors

The $\varLambda$CDM model is a limiting case of the $\varLambda$LTB metric. This makes the latter a useful tool to constrain departure from the homogeneous FLRW paradigm. This program is similar in spirit to one aimed at constraining departures from isotropy using the Bianchi models of Sect. IV.3.1. In Valkenburg et al. (2013b), available observations were used to constrain the amount of radial inhomogeneity $\delta _0$ at a given scale *L*. As stressed earlier, in this context, spherical symmetry is interpreted as the result of averaging the density field around us with a spherically symmetric filter. It is not assumed that the universe is spherically symmetric, rather that the observer averages over all angles, which is equivalent to expanding the full matter field in spherical harmonics, and throwing away all other information than the radially dependent monopole. The result of this analysis is shown in Fig. 58, blue contours. The red-to-gray contours shows the expected level of inhomogeneity within a $\varLambda$CDM model that satisfies the Copernican principle. Forthcoming data from Euclid should be able to help improving these constraints.

One can improve upon these constraints by including observables sensitive to the interior of our past light cone such as the fossil record of a large number of galaxies (Heavens et al. 2011). One can use the average star formation rate at a fixed lookback time as a diagnostic test for homogeneity, which is valid also outside the LTB framework. The lookback time has two elements to it—the lookback time of the emission of the light, plus the time along the past world line. The last of these can be probed using the integrated stellar spectra of the galaxies, using a code such as vespa (Tojeiro et al. 2007), and this is evidently dependent only on atomic and nuclear physics, independent of homogeneity. The lookback time can also be computed, surprisingly simply, without assuming homogeneity from (Heavens et al. 2011)

IV.3.32 $$\begin{aligned} \varDelta t = \int _0^z \frac{dz'}{(1+z')H_r(z')}, \end{aligned}$$

where $H_r$ is the radial Hubble constant. In principle, this can be obtained from radial BAOs, assuming early-time homogeneity so that the physical BAO scale is fixed. The spectroscopic part of Euclid could estimate both the star formation histories from stacked spectra, and the radial expansion rate.

Inhomogeneous models such as LTB are very useful in order to test various consistency relations which are valid only within the homogenous FLRW paradigm, see Sect. IV.3.2.4 and Clarkson (2012) for a review. For example, while in FLRW the function *H*(*z*) fixes the comoving distance $\chi (z)$ up to a constant curvature (and consequently also the luminosity and angular diameter distances), in the LTB model the relation between $\chi (z)$ and $H_{\perp }(z)$ or $H_{\Vert }(z)$ can be arbitrary. That is, one can choose the two spatial free functions to be for instance $H_{\perp }(r,0)$ and *R*(*r*, 0), from which the line-of-sight values $H_{\perp }(z)$ and $\chi (z)$ would also be arbitrarily fixed. This shows that the “consistency” FLRW relation between $\chi (z)$ and *H*(*z*) is violated in the LTB model, and in general in any strongly inhomogeneous universe. Further below we discuss how this consistency test can be exploited by Euclid to test for large-scale inhomogeneities. Recently, there has been an implementation of LTB models in large-scale structure *N*-body simulations (Alonso et al. 2010), where inhomogeneities grow in the presence of a large-scale void and seem to follow the predictions of linear perturbation theory. Since the LSS simulations capture the full relativistic behavior of matter as it falls into potential wells in the presence of a large void, one can use such simulations to test the non-linear dynamics of LTB models. In particular, one can follow the clustering of galaxies, the shear induced by the inhomogeneous background and the gravitational lensing induced by the whole web of cosmic structures.

An interesting class of tests on large-scale inhomogeneities involve probes of the growth of structure. However, progress in making theoretical predictions has been hampered by the increased complexity of cosmological perturbation theory in the LTB spacetime, where scalar and tensor perturbations couple, see e.g., Zibin (2008), Clarkson et al. (2009), Nishikawa et al. (2012), February et al. (2013) for recent progress. Nevertheless, a number of promising tests of large-scale inhomogeneity using the growth of structure have been proposed. Alonso et al. (2012) used *N*-body simulations to modify the Press–Schechter halo mass function, introducing a sensitive dependence on the background shear. The shear vanishes in spatially-homogeneous models, and so a direct measurement of this quantity would put stringent constraints on the level of background inhomogeneity, independent of cosmological model assumptions.

Purely geometric tests involving large-scale structure have been proposed, which neatly side-step the perturbation theory issue. The Baryon Acoustic Oscillations (BAO) measure a preferred length scale, *d*(*z*), which is a combination of the acoustic length scale, $l_s$, set at matter-radiation decoupling, and projection effects due to the geometry of the universe, characterized by the volume distance, $D_V(z)$. In general, the volume distance in an LTB model will differ significantly from that in the standard model, even if the two predict the same SN Ia Hubble diagram and CMB power spectrum. Assuming that the LTB model is almost homogeneous at the decoupling epoch, $l_s$ may be inferred from CMB observations, allowing the purely geometric volume distance to be reconstructed from BAO measurements. It has been shown by Zumalacárregui et al. (2012) that, based on these considerations, recent BAO measurements effectively rule out giant void models, independent of other observational constraints.

Another important consequence of late-time inhomogeneity is the impact of cosmic variance on cosmological parameters. Indeed, we can only observe the Universe from our own position which is fixed, in terms of cosmological scales. If we could move around in the Universe, we would measure the variation of local parameters, a variation caused by observing from locations with different values of the gravitational potential. However, as we cannot probe this unavoidable variation, there is a cosmic variance on the parameters inferred from observations which is systematic in nature. Particularly important for the dark-energy quest are the cosmic-variance errors on the dark-energy equation of state (Valkenburg et al. 2013a) and the Hubble parameter (Marra et al. 2013a; Ben-Dayan et al. 2014). Forthcoming probes such as Euclid will have to deal with this issue as the presence of unaccounted for inhomogeneity (either within the FLRW paradigm or not) could affect forecasts (see Sect. I.8) and cosmological inference.

Finally, the “cold spot” in the CMB sky could be attributed to a very large underdensity (Cruz et al. 2008; Masina and Notari 2009) allowing to directly test the assumption of local homogeneity. The recent discovery (Finelli et al. 2016) of a large underdensity ($\delta _0\simeq -0.2,\ r_0\simeq 200$ Mpc) in WISE-2MASS, at a median redshift of $z\simeq 0.2$, with an LTB profile that matches both the local galaxy density and the CMB cold spot anisotropy, opens the possibility to explore the local inhomogeneous Universe by cross-correlating the CMB secondary anisotropies and the local voids (and other structures) on large scales. We are thus entering the realm of the inhomogeneous universe beyond FRW, where a full understanding of local structures is necessary for a comprehensive picture of the universe.

*IV.3.2.3 Measuring the transition to homogeneity at different redshifts*

Large-scale homogeneity is usually assumed without proof when analyzing certain cosmological probes (Bonvin and Durrer 2011). This is often a reasonable approach, since it would not be possible to obtain many observational constraints without doing so. However, in order to be able to rely on these constraints, we must verify the validity of the Cosmological Principle independently in an unbiased way. Along these lines, different groups have argued that the Universe might in fact not reach a homogeneous regime on large scales, and that instead it behaves like a fractal (Coleman and Pietronero 1992; Pietronero et al. 1997; Montuori et al. 1997; Sylos Labini et al. 1998; Joyce et al. 1999; Sylos Labini et al. 2009; Sylos Labini 2011), while other groups claim the opposite result: the predictions of the standard $\varLambda$CDM model (Martinez and Coles 1994; Guzzo 1997; Sylos Labini and Amendola 1997; Martinez et al. 1998; Scaramella et al. 1998; Amendola and Palladino 1999; Pan and Coles 2000; Kurokawa et al. 2001; Yadav et al. 2005; Sarkar et al. 2009; Scrimgeour et al. 2012; Nadathur 2013).

The disparity between these two results seems to stem from the differences in the analysis methods. On the one hand it is desirable to use methods that are, as far as possible, free of assumptions, especially regarding the property you want to measure. However, in the case of the validity of the Cosmological Principle, this is not an easy task, since homogeneity must som etimes be assumed in order to cope with certain observational effects. At the end of the day, we must ensure that the method used is able to distinguish homogeneous from non-homogeneous models to a reasonable level of precision. A robust and popular method to study the transition to homogeneity in the matter density field at late times is to analyze the fractality of the galaxy distribution in a redshift survey. Furthermore, fractal dimensions can be used to quantify clustering, since they depend on the scaling of the different moments of galaxy counts in spheres, which in turn are related to the n-point correlation functions. As has been said, the homogeneous regime is reached, within the standard $\varLambda$CDM model, at very large scales, and therefore a large survey volume is necessary in order to safely claim a detection of this transition. In this sense, photometric galaxy redshift surveys such as DES (The Dark Energy Survey Collaboration 2017) provide a unique opportunity for this study, since they are able to observe large numbers of objects distributed across wide areas and to further redshifts than their spectroscopic counterparts. The main caveat of these surveys is that, due to the limited precision in the redshift determination, much of the radial information is lost, and we are only able to study angular clustering in different thick redshift slices. Hence, in order to study the fractality of the galaxy distribution with a photometric survey, the methods and estimators used in previous analyses must be adapted to draw results from angular information alone. One advantage of this approach is that, since angular positions are pure observables (unlike three-dimensional distances, which can only be calculated assuming a fiducial cosmology), the results obtained are completely model independent.

In Alonso et al. (2013), we the authors proposed an observable, the angular homogeneity index H2, which could be used by photometric surveys in the near-future to study the fractal structure of the galaxy distribution.

*IV.3.2.4 Reconstructing the global curvature at different redshifts*

Clarkson et al. (2008) presented an observational test for the Copernican principle which relies on the consistency relation between expansion rate and angular diameter distance. This test is valid also outside the LTB framework discussed earlier. Here we discuss the implications for Euclid.

Let us recall that the angular diameter distance in a FLRW model can be written as [see Eq. (I.7.24) and the surrounding discussion]:

IV.3.33 $$\begin{aligned} D_{A}(z)=\frac{1}{1+z}\frac{1}{H_{0}\sqrt{-\varOmega ^{(0)}_{K}}}\sin \left( \sqrt{-\varOmega ^{(0)}_{K}}\int _{0}^{z}{dz'\frac{H_{0}}{H(z')}}\right) . \end{aligned}$$

where $\varOmega ^{(0)}_{K}$ is the curvature parameter *today*. We can invert the last equation to obtain an expression for the curvature parameter that depends on the Hubble parameter *H* and comoving angular diameter distance $D\left( z\right) =\left( 1+z\right) D_{A}\left( z\right)$ only, see Clarkson et al. (2008):

IV.3.34 $$\begin{aligned} \varOmega ^{(0)}_{K}=\frac{\left[ H\left( z\right) D'\left( z\right) \right] ^{2}-1}{\left[ H_{0}D\left( z\right) \right] ^{2}}, \end{aligned}$$

where here the prime refers to the derivative with respect the redshift. Then Eq. (IV.3.34) tells us how the curvature parameter can be measured from the distance and the Hubble rate observations, in a model-independent way. The idea is then to measure the curvature parameter $\varOmega ^{(0)}_{K}$ at different redshifts. Let us consider again Eq. (IV.3.34); if we are in a FLRW universe then $\varOmega ^{(0)}_K$ should be independent of redshift, i.e., its derivative with respect to *z* should be zero

IV.3.35 $$\begin{aligned} \mathcal {C}(z) = \frac{{\mathrm {d}}\varOmega ^{(0)}_{K}}{{\mathrm {d}}z}=0. \end{aligned}$$

If it happens that $\mathcal {C}(z)\ne 0$ even at a single redshift then this means the large-scale universe is not homogeneous.

A possible test to measure $\varOmega ^{(0)}_K$ at various redshifts is provided by baryon acoustic oscillations. Observing the features of BAO in the galaxy power spectrum in both angular (orthogonal to the line of sight $L_{\perp }$) and radial direction (along the line of sight $L_{\parallel }$) allows us to measure with a great accuracy both $D_{A}(z)$ and *H*(*z*), respectively. If the geometry is not FLRW, then the standard BAO will be deformed in three different ways:

1. The sound horizon scale, which is the characteristic ruler, will be different in the $\perp$ and $\parallel$ directions and it will be also different from that for the FLRW universe.
2. Even if the sound horizon were isotropic at decoupling, the subsequent expansion in the $\perp$ and $\parallel$ directions will be different just because they will be governed by two distinct Hubble parameters: $H_{\perp }$ and $H_{\parallel }$.
3. The redshift distortion parameter will be different because it will depend on the background expansion.

Also the growth factor will be modified, perhaps in a scale dependent way. If the true underlying model is radically inhomogeneous, but we assume a FLRW in interpreting the observations, the derived cosmological parameters will be biased (or unphysical) and the parameters derived from BAO data will be different from those measured by SN Ia and/or lensing. As argued also in different contexts, a mismatch on the value of one of more parameters may indicate that we are assuming a wrong model.

**Fig. 59:**
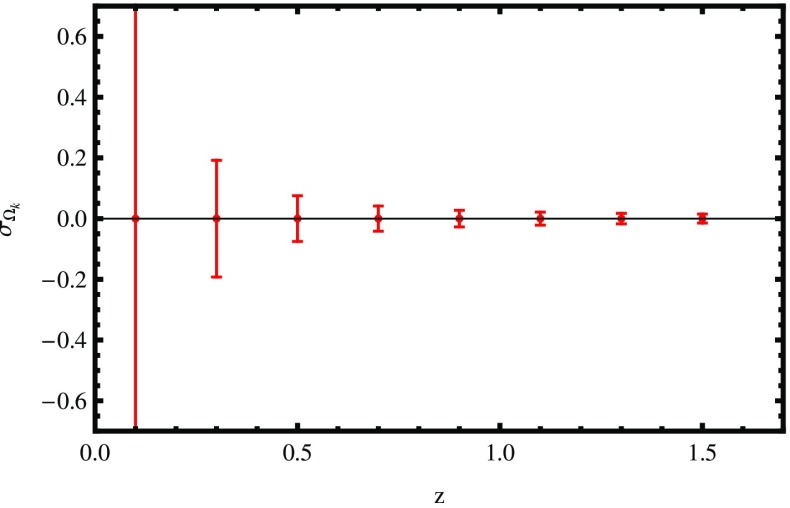
Relative errors on $\varOmega _{K}$ for our benchmark survey for different redshifts

**Fig. 60:**
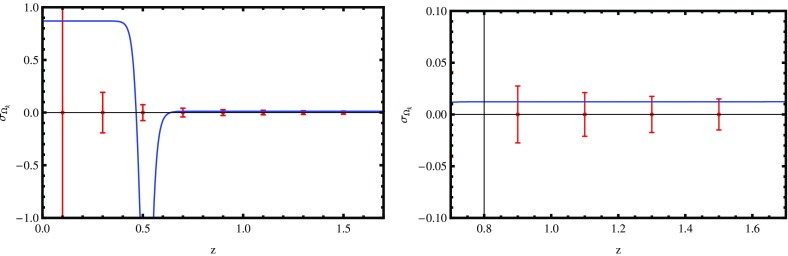
Left: same as Fig. 59 but now with superimposed the prediction for the Lemaître–Tolman–Bondi model considered by García-Bellido and Haugbølle (2008a). Right: zoom in the high-redshift range

We show here the sensitivity that can be reached with an experiment like Euclid for the curvature parameter $\varOmega ^{(0)}_{K}$. We choose a redshift survey with a depth of $z=1.6$ and consider different redshift bins. In Fig. 59 we show the first $1\sigma$ absolute errors on the curvature parameter for different redshift bins that can be obtained measuring the Hubble parameter and the angular diameter distance. In obtaining these errors we used Fisher-based forecasts for the radial and angular BAO signal following Seo and Eisenstein (2003), Eisenstein et al. (2007), as discussed in Sect. I.7.3. The sensitivity that can be reached with an experiment like Euclid is extremely high; we can measure the curvature parameter better than 0.02 at redshift of $z\simeq 1$. This will allow us to discriminate between FLRW and averaged cosmology as for example illustrated in Fig. 60.

An alternative to measuring the global curvature is to measure the shear of the background geometry. If there is a large inhomogeneous void then a congruence of geodesics will not only expand but also suffer shear (García-Bellido and Haugbølle 2009). The amount of shear will depend on the width and magnitude of the transition between the interior of the void and the asymptotic Einstein–de Sitter universe. Normalizing the shear w.r.t. the overall expansion, one finds (García-Bellido and Haugbølle 2009)

IV.3.36 $$\begin{aligned} \varepsilon = \frac{H_\perp (z)-H_{||}(z)}{2H_\perp +H_{||}} \simeq \frac{1 - H_{||}(z)\,\partial _z\Big [(1+z)\,D_A(z)\Big ]}{3H_{||}(z)D_A(z) + 2\Big (1 - H_{||}(z)\,\partial _z\Big [(1+z)\,D_A(z)\Big ]\Big )}.\nonumber \\ \end{aligned}$$

Clearly, in homogeneous FRW universes the shear vanishes identically since $H_\perp = H_{||} = H$. Also note that the function $H_{||}(z)D_A(z)$ is nothing but the Alcock–Paczynski factor, which is normally used as a geometric test for the existence of vacuum energy in $\varLambda$CDM models.

#### Backreaction of inhomogeneities on overall expansion

Besides the observational effects of inhomogeneities on the propagation of photons, it has been argued (Räsänen 2004; Kolb et al. 2005; Ellis and Buchert 2005) that large-scale structures could—due to the nonlinear nature of gravity—affect the way the homogeneous and isotropic background metric itself evolves. In other words, there could be a backreaction of inhomogeneities on the background which would evolve according to modified Friedmann equations. The motivation—as said earlier—stems from the coincidence that large-scale structures became nonlinear recently, exactly when a primary dark energy is supposed to start dominating the energy content of the universe and cause acceleration.

In general, we would like to compute directly the impact of the inhomogeneities, without requiring an exact and highly symmetric solution of Einstein’s equations like FLRW or even LTB. Unfortunately there is no easy way to approach this problem. One ansatz tries to construct average quantities that follow equations similar to those of the traditional FLRW model, see Buchert (2000, 2008). In this framework, it is possible to obtain a set of equations, often called the Buchert equations, that look surprisingly similar to the Friedmann equations for the averaged scale factor $a_\mathcal {D}$, with extra contributions:

IV.3.37 $$\begin{aligned} 3\left( \frac{{\dot{a}}_\mathcal {D}}{a_\mathcal {D}}\right) ^2 - 8\pi G \left\langle \varrho \right\rangle _\mathcal {D}-\varLambda= & {} - \frac{\left\langle \mathcal {R} \right\rangle _\mathcal {D}+{\mathcal {Q}}_\mathcal {D}}{2} \;, \end{aligned}$$

IV.3.38 $$\begin{aligned} 3\frac{{\ddot{a}}_\mathcal {D}}{a_\mathcal {D}} + 4\pi G \left\langle \varrho \right\rangle _\mathcal {D} -\varLambda= & {} {\mathcal {Q}}_\mathcal {D}. \end{aligned}$$

Here $\mathcal {R}$ is the 3-Ricci scalar of the spatial hypersurfaces and the kinematical backreaction $\mathcal {Q}$ is given by

IV.3.39 $$\begin{aligned} {{\mathcal {Q}}}_\mathcal {D}= \frac{2}{3}\left\langle \left( \theta - \left\langle \theta \right\rangle _\mathcal {D}\right) ^2 \right\rangle _\mathcal {D} - 2\left\langle \sigma ^2 \right\rangle _\mathcal {D}, \end{aligned}$$

i.e., it is a measure of the variance of the expansion rate $\theta$ and of the shear $\sigma _{ij}$. We see that this quantity, if positive, can induce an accelerated growth of $a_\mathcal {D}$, which suggests that observers would conclude that the universe is undergoing accelerated expansion.

Unfortunately, the Buchert equations are not closed and further input is needed. There has been extensive work trying to determinate if the backreaction effect is negligible or not. For example, Clifton et al. (2013), Yoo and Okawa (2014) and Bruneton and Larena (2012) considered approaches which model the universe as a lattice of Schwarzschild patches. However, after more than 10 years of research it is not yet clear if backreaction can account for dark energy or not, see e.g., Kolb (2011), Rasanen (2011), Green and Wald (2013) and references therein.

An alternative route is to avoid the fact that the Buchert equations are not closed by imposing by hand an effective, average geometry with the help of a template metric that only holds on average. The probably simplest first choice is to impose on each spatial hypersurface a spatial metric with constant curvature, by imagining that the inhomogeneities have been smoothed out. But in general the degrees of freedom of this metric (scale factor and spatial curvature) will not evolve as in the FLRW case, since the evolution is given by the full, inhomogeneous universe, and we would not expect that the smoothing of the inhomogeneous universe follows exactly the evolution that we would get for a smooth (homogeneous) universe. For example, the average curvature could grow over time, due to the collapse of overdense structure and the growth (in volume) of the voids. Thus, unlike in the FRLW case, the average curvature in the template metric should be allowed to evolve. This is the case that was studied in Larena et al. (2009).

**Fig. 61:**
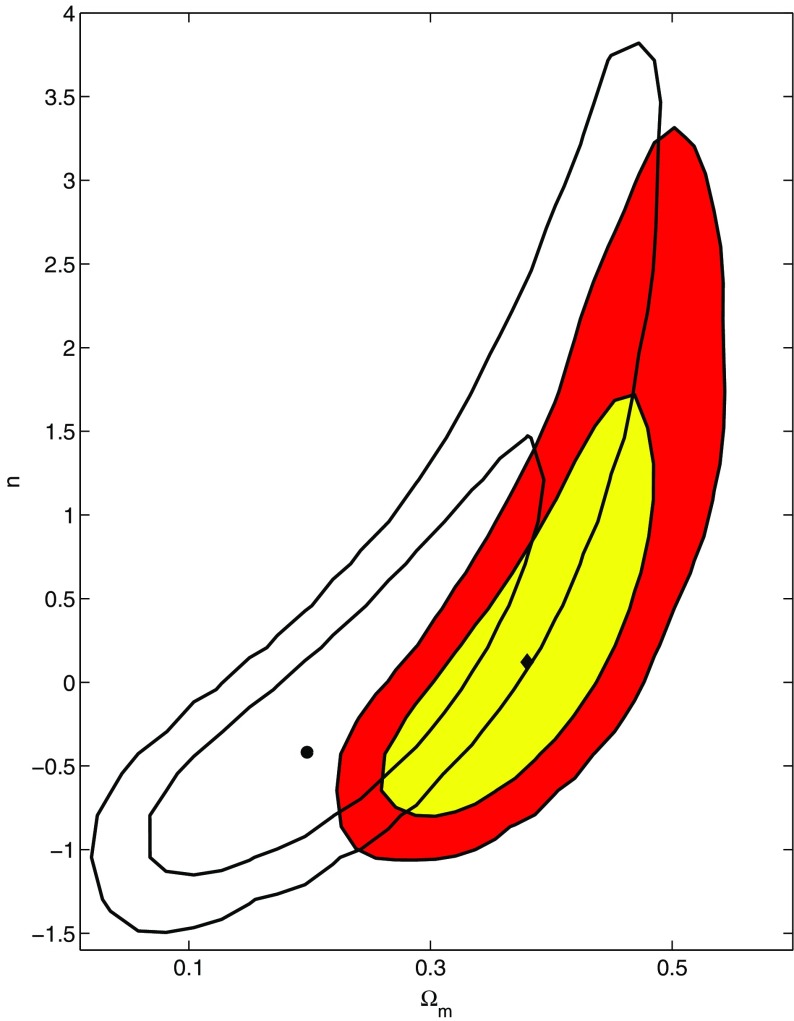
Supernova and CMB constraints in the $(\varOmega ^{\mathcal {D}_0}_{m},n)$ plane for the averaged effective model with zero Friedmannian curvature (filled ellipses) and for a standard flat FLRW model with a quintessence field with constant equation of state $w=-\,(n+3)/3$ (black ellipses). The disk and diamond represent the absolute best-fit models respectively for the standard FLRW model and the averaged effective model

However, imposing a template metric is not enough. Firstly, although there is an integrability condition linking the evolution of $\left\langle \mathcal {R} \right\rangle _\mathcal {D}$ and $\mathcal {Q}_\mathcal {D}$ and in addition a consistency requirement that the effective curvature $\kappa (t)$ in the metric is related to $\left\langle \mathcal {R} \right\rangle _\mathcal {D}$, we still need to impose an overall evolution by hand as it was not yet possible to compute this from first principles. Larena et al. (2009) assumed a scaling solution $\left\langle \mathcal {R} \right\rangle _\mathcal {D}\propto a_\mathcal {D}^n$, with *n* a free exponent. In a dark energy context, this scaling exponent *n* corresponds to an effective dark energy with $w_\mathcal {D}= -\,(n+3)/3$, but in the backreaction case with the template metric the geometry is different from the usual dark energy case. A perturbative analysis (Li and Schwarz 2007) found $n=-\,1$, but of course this is only an indication of the possible behavior as the situation is essentially non-perturbative. The second choice concerns the computation of observables. Larena et al. (2009) studied distances to supernovae and the CMB peak position, effectively another distance. The assumption taken was that distances could be computed within the averaged geometry as if this was the true geometry, by integrating the equation of radial null geodesics. In other words, the effective metric was taken to be the one that describes distances correctly. The resulting constraints are shown in Fig. 61. We see that the leading perturbative mode ($n=1$) is marginally consistent with the constraints. These contours should be regarded as an indication of what kind of backreaction is needed if it is to explain the observed distance data.

One interesting point, and maybe the main point in light of the discussion of Sect. IV.3.2.4, is that the averaged curvature needs to become necessarily large at late times due to the link between it and the backreaction term $\mathcal {Q}$, in order to explain the data. Just as in the case of a large deviation from homogeneity, this effective curvature makes the backreaction scenario testable to some degree with future large surveys like Euclid.

### Speculative avenues: non-standard models of primordial fluctuations

In this section we explore other non-conventional scenarios that challenge our understanding of the universe. Here we present models that include mechanisms for primordial anisotropy in the fluctuation spectrum, due to spacetime non-commutativity, to inflationary vector fields or to super-horizon fluctuations. Since inflation can occur at high energies for which we lack robust direct experimental probes, it is reasonable to pay attention on possible deviations from some standard properties of low energy physics. We review these here and point out possible observables for the Euclid project.

#### Probing the quantum origin of primordial fluctuations

Conventionally, the 2-point correlation function of a random variable $X(\mathbf {k},t)$ is regarded as a classical object, related to the power spectrum $P_{X}$ via the relation

IV.4.1 $$\begin{aligned} \langle X(\mathbf {k},t)X\left( \mathbf {k}',t\right) \rangle = (2\pi )^{3}\delta \left( \mathbf {k}-\mathbf {k}'\right) P_{X}(k), \end{aligned}$$

where $$k=|\mathbf {k}|$$.

When we look at $X(\mathbf {k},t)$ in terms of a *quantum field* in momentum space, we need to reinterpret the average $\langle \cdots \rangle$ as the expectation value of the 2-point function over a determined quantum state. This raises several issues that are usually ignored in a classical analysis. For instance, the value of the expectation value depends in the algebra of the annihilation and creation operators that compose the field operator. Any non-trivial algebra such as a non-commutative one, leads to non-trivial power spectra. Also, the quantum expectation value depends on the state of the field, and different choices can lead to radically different results.

Suppose that $\varphi (\mathbf {x},t)$ represents a perturbation propagating on an inflationary background. Upon quantization, we have

IV.4.2 $$\begin{aligned} {\hat{\varphi }}(\mathbf {x},t)=(2\pi )^{-3/2}\int \mathrm {d}^{3}k\left[ \varphi _{k}(t){\hat{a}}_{\mathbf {k}}\,e^{i\mathbf {k}\cdot t}+\varphi _{k}^{*}(t){\hat{a}}_{\mathbf {k}}^{\dagger }\,e^{-i\mathbf {k}\cdot t}\right] , \end{aligned}$$

where ${\hat{a}}_{\mathbf {k}}$ is the usual annihilation operator. When calculated in the limit $\mathbf {k}\rightarrow \mathbf {k}'$, the expectation value of the two-point function in coordinate space diverges, signalling the breakdown of the theory at short distances. From the quantum field theory perspective, this means that the expectation value needs to be regularized in the ultraviolet (UV). It has been argued that this has in specific scenarios sizeable effects on the observable spectrum—see e.g., Agulló et al. (2010), Marozzi et al. (2011), see however, e.g., Durrer et al. (2009) for a contrary viewpoint.

In addition to UV divergences, there are infrared (IR) ones in long-range correlations. Usually, one tames these by putting the universe in a box and cutting off super-horizon correlations. However, several authors have recently proposed more sensible IR regulating techniques, see e.g., Giddings and Sloth (2011), Koivisto and Prokopec (2011). Very natural ways to obtain IR finite results are to take into account the presence of tiny spatial curvature or a pre-inflationary phase which alters the initial conditions (Janssen and Prokopec 2011; Koivisto and Prokopec 2011). In principle these regularizations will leave an imprint in the large-scale structure data, in the case that regularization scale is not too far beyond the present horizon scale. If this pre-inflationary phase is characterized by modified field theory, such as modified dispersion relations or lower dimensional effective gravity, the scalar and tensor power spectra show a modification whose magnitude is model-dependent, see e.g., Rinaldi (2012).

The two-point function of a scalar field is constructed from basic quantum field theory, according to a set of rules determined in the context of relativistic quantum mechanics. In particular, the usual commutation rules between position and momentum are promoted to commutation rules between the field and its canonical conjugate. A modification of the fundamental quantum mechanical commutation rules can easily be generalized to field theory. The most popular case is represented by non-commutative geometry, which implies that coordinate operators do not commute, i.e.,

IV.4.3 $$\begin{aligned} \left[ {\hat{x}}^{\mu },{\hat{x}}^{\nu }\right] =i\theta ^{\mu \nu }, \end{aligned}$$

where $\theta ^{\mu \nu }$ is an anti-symmetric matrix, usually taken to be constant, see e.g., Snyder (1947) and Connes (1990). There are many fundamental theories that phenomenologically reduce to an ordinary field theory over a non-commutative manifold, from string theory to quantum gravity. It is therefore important to consider the possibility that non-commutative effects took place during the inflationary era and try to extract some prediction.

One can construct models where the inflationary expansion of the universe is driven by non-commutative effects, as in Alexander et al. (2003) and Rinaldi (2011). In this kind of models, there is no need for an inflaton field and non-commutativity modifies the equation of state in the radiation-dominated universe in a way that it generates a quasi-exponential expansion. The initial conditions are thermal and not determined by a quantum vacuum. For the model proposed in Alexander et al. (2003), the predictions for the power spectra have been worked out in Koh and Brandenberger (2007). Here, Brandenberger and Koh find that the spectrum of fluctuations is nearly scale invariant, and shows a small red tilt, the magnitude of which is different from what is obtained in a usual inflationary model with the same expansion rate.

On the other hand, non-commutativity could introduce corrections to standard inflation. Such a, perhaps less radical approach, consists in assuming the usual inflaton-driven background, where scalar and tensor perturbations propagate with a Bunch and Davies vacuum as initial condition, but are subjected to non-commutativity at short distance. It turns out that the power spectrum is modified according to (see e.g., Koivisto and Mota 2011, and references therein)

IV.4.4 $$\begin{aligned} P=P_{0}\,e^{H\mathbf {\theta }\cdot \mathbf {k}}, \end{aligned}$$

where *H* is the Hubble parameter, $P_{0}$ is the usual commutative spectrum, and $\mathbf {\theta }$ is the vector formed by the $\theta ^{0i}$ components of $\theta ^{\mu \nu }$. This prediction can be obtained by using a deformation of statistics in non-commutative spacetime on the usual inflationary computation. It can be also derived in an alternative way beginning from an effective deformation of the Heisenberg algebra of the inflaton field. The most important aspect of the result is that the spectrum becomes direction-dependent. The perturbations thus distinguish a preferred direction given by the vector $\mathbf {\theta }$ that specifies the non-commutativity between space and time.

Furthermore, it is interesting that the violation of isotropy can also violate parity. This could provide what seems a quite unique property of possible signatures in the CMB and large-scale structure. However, there is also an ambiguity with the predictions of the simplest models, which is related to interpretations of non-commuting quantum observables at the classical limit. This is evident from the fact that one has to consider an effectively imaginary $\varvec{\theta }$ in the above formula (IV.4.4). Reality of physical observables requires the odd parity part of the spectrum (IV.4.4) to be imaginary. The appearance of this imaginary parameter $\mathbf {\theta }$ into the theory may signal the unitary violation that has been reported in theories of time–space non-commutativity. It is known that the Seiberg–Witten map to string theory applies only for space–space non-commutativity (Seiberg and Witten 1999). Nevertheless, the phenomenological consequence that the primordial fluctuations can distinguish handedness, seems in principle a physically perfectly plausible—though speculative—possibility, and what ultimately renders it very interesting is that we can test by cosmological observations. Thus, while lacking the completely consistent and unique non-commutative field theory, we can parametrize the ambiguity by a phenomenological parameter whose correct value is left to be determined observationally. The parameter $\alpha \in [0,1]$ can be introduced (Koivisto and Mota 2011) to quantify the relative amplitude of odd and even contributions in such a way that $P = \alpha P^+ + i(1-\alpha )P^-$, where $P^\pm = (P(\mathbf {k})\pm P(-\mathbf {k}))/2$.

The implications of the anisotropic power spectra, such as (IV.4.4), for the large-scale structure measurements, is discussed below in Sect. IV.4.3. Here we proceed to analyse some consequences of the non-commutativity relation (IV.4.3) to the higher order correlations of cosmological perturbations. We find that they can violate both isotropy and parity symmetry of the FRW background. In particular, the latter effect persists also in the case $\alpha =1$. This case corresponds to the prescription in Akofor et al. (2008) and in the remainder of this subsection we restrict to this case for simplicity. Thus, even when we choose this special prescription where the power spectrum is even, higher order correlations will violate parity. This realizes the possibility of an odd bispectrum that was recently contemplated upon in Kamionkowski and Souradeep (2011).

More precisely, the functions *B* defined in Eq. (III.3.4) for the three-point function of the curvature perturbation can be shown to have the form

IV.4.5 $$\begin{aligned} B_\varPhi (\mathbf {k}_1,\mathbf {k}_2,\mathbf {k}_3)= & {} 2\cos {\left( \mathbf {k}_1\wedge \mathbf {k}_2 \right) }\Big (\cosh (2H\varvec{\theta }\cdot \mathbf {k}_3)P_0(\mathbf {k}_1)P_0(\mathbf {k}_2)f_s(\mathbf {k}_3) + 2\,{\mathrm {perm.}}\Big ) \nonumber \\&-\, 2i\sin {\left( \mathbf {k}_1\wedge \mathbf {k}_2 \right) }\Big (\sinh (2H\varvec{\theta }\cdot \mathbf {k}_3)P_0(\mathbf {k}_1)P_0(\mathbf {k}_2)f_s(\mathbf {k}_3) + 2\,{\mathrm {perm.}}\Big ),\nonumber \\ \end{aligned}$$

where the function $f_s(k)$ is

IV.4.6 $$\begin{aligned} f_s(k)=\frac{N''}{2N'^2}\left( 1+{n_{f_{{\mathrm {NL}},0}}}\,\ln \frac{k}{k_p}\right) , \end{aligned}$$

$k_p$ being a pivot scale and primes denoting derivatives with respect to the inflaton field. The quantity ${n_{f_{{\mathrm {NL}},0}}}$ is the scale dependence in the commutative case explicitly given by

IV.4.7 $$\begin{aligned} {n_{f_{{\mathrm {NL}},0}}} =\frac{N'}{N''}\left( -3\eta + \frac{V'''}{3H^2}\right) . \end{aligned}$$

The spatial components of the non-commutativity matrix $\theta _{ij}$ enter the bispectrum through the phase $\mathbf {k}_1\wedge \mathbf {k}_2= k_1^i k_2^j\,\theta _{ij}$. They do not appear in the results for the spectrum and therefore affect only the non-Gaussian statistics of primordial perturbations.

We now focus on this part in the following only and set all components of $\varvec{\theta }$ equal to zero. This gives

IV.4.8 $$\begin{aligned} f_{{\mathrm {NL}},\theta }= & {} \frac{5}{3}\cos {\left( \mathbf {k}_1\wedge \mathbf {k}_2 \right) }\frac{P_{0}(k_1)P_{0}(k_2)f_s(k_3)+2\,{\mathrm {perm.}}}{P_{0}(k_1)P_{0}(k_2)+2\,{\mathrm {perm.}}}, \end{aligned}$$

where the only contribution from the non-commutativity is the pre-factor involving the wedge product. This affects the scale dependence of ${n_{f_{{\mathrm {NL}},\theta }}}$ and can hence be constrained observationally. For example, computing the scale-dependence for shape preserving variations of the momentum space triangle, $\mathbf {k}_i\rightarrow \lambda \mathbf {k}_i$, defined as

IV.4.9 $$\begin{aligned} {n_{f_{{\mathrm {NL}},\theta }}} = \frac{\partial \ln |f_{{\mathrm {NL}},\theta }\left( \lambda \mathbf {k}_1,\lambda \mathbf {k}_2,\lambda \mathbf {k}_3\right) |}{\partial \ln \lambda }\Big |_{\lambda =1}, \end{aligned}$$

we find, in the present case

IV.4.10 $$\begin{aligned} {n_{f_{{\mathrm {NL}},\theta }}} = -2k_1^ik_2^j\theta _{ij}\tan \left( k_1^ik_2^j\theta _{ij}\right) +{n_{f_{{\mathrm {NL}},0}}}, \end{aligned}$$

where ${n_{f_{{\mathrm {NL}},0}}}$ given by (IV.4.7) is the result in the commuting case. The part dependent on $\theta _{ij}$ arises purely from non-commutative features. The Euclid data can be used to constrain the scale dependence of the nonlinearity parameter $f_{{\mathrm {NL}},\theta }$, and the scale dependence could therefore place interesting bounds on $\theta _{ij}$. We note however that the amplitude of the nonlinearity is not enhanced by the purely spatial non-commutativity, but is given by the underlying inflationary model. The amplitude on the other hand is exponentially enhanced by the possible timespace non-commutativity.

Moreover, it is worth noting that the result (IV.4.10) depends on the wave vectors $\mathbf {k}_1$ and $\mathbf {k}_2$ and hence on the shape of the momentum space triangle. This is in contrast with the commutative case, where the scale dependence is given by the same result (IV.4.7) for all shape preserving variations, $\mathbf {k}_i\rightarrow \lambda \mathbf {k}_i$, regardless of triangle shape. This allows, in principle, to distinguish between the contributions arising from the non-commutative properties of the theory and from the standard classical inflationary physics or gravitational clustering.

To recapitulate, parity violations in the statistics of large-scale structures would be a smoking gun signature of timespace non-commutativity at work during inflation. Moreover, purely spatial non-commutativity predicts peculiar features in the higher order correlations of the perturbations, and in particular these can be most efficiently detected by combining information of the scale- and shape-dependence of non-Gaussianity. As discussed earlier in this document, this information is extractable from the Euclid data.

#### Vector field models and modulated perturbations

Various inflationary models populated by vector fields can be described with a Lagrangian of the following form

IV.4.11 $$\begin{aligned} L_{\mathrm {vector}}=-\frac{1}{4} f(\varphi ) F_{\mu \nu }F^{\mu \nu }+\frac{1}{2}m^2 B_{\mu }B^{\mu }, \end{aligned}$$

where $F_{\mu \nu }\equiv \partial _{\mu }B_{\nu }-\partial _{\nu }B_{\mu }$, and $f(\varphi )$ is a suitable function of the inflaton field. A Lagrangian containing just the standard kinetic term $F_{\mu \nu }F^{\mu \nu }$ would be conformally invariant thus preventing fluctuations of the vector field $B_{\mu }$ to be excited on super-horizon scales. Contrary to the case of a light scalar field, large-scale primordial perturbations of the vector field can be generated during inflation if the vector field is sufficiently massive (with $m^2\approx -2H^2$). This Lagrangian includes the case of a massive (curvaton) vector field (when $f\equiv 1$) studied by Dimopoulos (2006) and Dimopoulos and Karčiauskas (2008) and where the mass of the vector field is acquired via a non-minimal coupling to gravity to break conformal invariance. For some of these models there are actually some instability issues about the evolution of the primordial longitudinal perturbation modes of the vector field (Himmetoglu et al. 2009b, a). The models with varying kinetic function (when $f(\varphi )$ is switched on) allows to overcome these difficulties, since in this case the longitudinal mode is gauged away. They have been studied in various contexts (e.g., Yokoyama and Soda 2008; Dimopoulos et al. 2010). The Ackerman–Carroll–Wise models (Ackerman et al. 2007) employ a different Lagrangian of the form $L_{\mathrm {vector}}=-\frac{1}{4} F_{\mu \nu }F^{\mu \nu }+\lambda (B^\mu B_\mu -m^2)$, so that the norm of the vector field is fixed by the Lagrangian multiplier $\lambda$. In these models (where inflation is driven by an inflaton field) the main effect of the vector field is a slightly anisotropic background evolution described by a metric, with $c(t)=b(t)$) with a backreaction on the inflaton field fluctuations, rather than the vector field perturbations themselves. Another possibility that has been explored is based on a non-Abelian gauge *SU*(2) vector multiplet (Bartolo et al. 2009a, b), providing a realistic model of gauge interactions neglected so far.

A general prediction from all these scenarios is that the power spectrum of primordial perturbations can be written as

IV.4.12 $$\begin{aligned} P(\mathbf {k})=P(k)\left[ 1+g(k) \left( \hat{\mathbf {k}} \cdot \hat{\mathbf {n}}\right) ^2 \right] , \end{aligned}$$

where *g*(*k*) is the amplitude of the rotational invariance breaking (statistical isotropy breaking) induced by a preferred direction $\mathbf {n}$. Thus, the power spectrum is not just a function of *k* but it depends on the wave vector $\mathbf {k}$. Usually the preferred direction is related to the vector fields $n^i \propto B^i$ while the amplitude is related to the contribution of the vector field perturbations to the total curvature perturbation $g \sim P_{\zeta _B}/P_\zeta$.

However, beyond the various concrete realizations, the expression (IV.4.12), first introduced in Ackerman et al. (2007), provides a robust and useful way to study observable consequences of a preferred direction during inflation and also a practical template for comparison with observations (see below) (see also Ashoorioon et al. 2016). Usually the amplitude *g*(*k*) is set to a constant $g_*$. A generalization of the above parametrization is $P(\mathbf {k})=P(k)\left[ 1+\sum _{LM} g_{\mathrm {LM}}(k) Y_{LM}(\hat{\mathbf {k}}) \right]$, where $Y_{\mathrm {LM}}(\hat{\mathbf {k}})$ are spherical harmonics with only even multipoles $L \ge 2$ (Pullen and Kamionkowski 2007). Throughout, we use upper-case indices *LM* for power anisotropies, and lower-case indices *lm* for temperature/polarization anisotropies. Interestingly enough, inflationary models with vector fields can also generate higher-order correlators, such as bispetrum and trispectrum, which display anisotropic features as well (e.g., Yokoyama and Soda 2008; Karčiauskas et al. 2009; Bartolo et al. 2009a, b).

The alignment of low CMB multipoles and the hemispherical power asymmetry observed in the CMB anisotropies can find an explanation in some models where the primordial gravitational perturbation is the result of fluctuations within our Hubble volume, modulated by super-horizon fluctuations. The primordial gravitational perturbation can thus be thought of as a product of two fields $\varPhi _1(\mathbf {x})$ and $\varPhi _2(\mathbf {x})$ (Dvorkin et al. 2008, and references therein)

IV.4.13 $$\begin{aligned} \varPhi (\mathbf {x})=\varPhi _1(\mathbf {x})\left[ 1+\varPhi _2(\mathbf {x}) \right] , \end{aligned}$$

where $\varPhi _2(\mathbf {x})$ has only super-horizon fluctuations, so that within a given Hubble volume it takes a fixed value, while $\varPhi _1(\mathbf {x})$ has sub-horizon stochastic fluctuations within that volume. The result is that an observer within our Hubble volume would see broken statistical homogeneity from the modulation on large scales of $\varPhi _1(\mathbf {x})$, and also broken statistical isotropy from the gradient of the modulating field $\varPhi _2(\mathbf {x})$. The dipole modulation $\delta T (\hat{\mathbf {p}})/T = S(\hat{\mathbf {p}})\left[ 1+A(\hat{\mathbf {p}} \cdot \hat{\mathbf {n}}) \right]$ used for CMB by, e.g., Eriksen et al. (2007) and Hanson and Lewis (2009) (or for LSS Hirata 2009) to explain the hemispherical asymmetry falls within the parametrization of Eq. (IV.4.13). A scenario with a dipole modulation has been realized in some concrete and detailed models, such as those involving adiabatic and isocurvature modulating perturbations from a curvaton field (Erickcek et al. 2008, 2009).

#### Current and future constraints from CMB and LSS on an anisotropic power spectrum

Groeneboom and Eriksen (2009), using WMAP5 year data (up to multipoles $\ell =400$), claimed a detection of a quadrupolar power spectrum of the form of Eq. (IV.4.12) at more than $3 \sigma$ ($g_*=0.15 \pm 0.039$) with preferred direction $(l,b)=(110^{\circ }, 10^{\circ })$. Subsequently this result has been put under further check. Hanson and Lewis (2009) confirmed this effect at high statistical significance, pointing out however that beam asymmetries could be a strong contaminant (see also Hanson et al. 2010). The importance of this systematic effect is somewhat debated: Groeneboom et al. (2010), including polarization and beam asymmetries analysis excluded that the latter can be responsible for the observed effect. Their claim is a $9 \sigma$ detection with $g_*=0.29 \pm 0.031$. However, the preferred direction shifted much closer to the ecliptic poles, which is probably an indication that some unknown systematic is involved and must be corrected in order to obtain true constraints on any primordial modulation. Foregrounds and noise are disfavored as possible systematic effects (Bennett et al. 2011; Groeneboom and Eriksen 2009). Thus the cause of this kind of asymmetry is not definitely known. Planck should be able to detect a power quadrupole as small as 2% (at $3 \sigma$) (Pullen and Kamionkowski 2007; Groeneboom and Eriksen 2009; Groeneboom et al. 2010). It is of course desirable to test this (and other anisotropic effects) with other techniques.

What about large-scale structure surveys? Up to now there are just a few analyses testing anisotropies in large-scale structure surveys, but all of them have been crucial, indicating that large-scale structure surveys such as Euclid offer a promising avenue to constrain these features.

Hirata (2009) used high-redshift quasars from the Sloan Digital Sky Survey to rule out the simplest version of dipole modulation of the primordial power spectrum. In comparison the Planck mission using the CMB hemispherical asymmetry would only marginally distinguish it from the standard case (Eriksen et al. 2007). The constraints obtained by high-redshift quasars require an amplitude for the dipole modulation 6 times smaller than the one required by CMB. This would disfavor the simple curvaton spatial gradient scenario (Erickcek et al. 2008) proposed to generate this dipole modulation. Only a curvaton scenario with a non-negligible fraction of isocurvature perturbations at late times could avoid this constraint from current high-redshift quasars (Erickcek et al. 2009).

Pullen and Hirata (2010) considered a sample of photometric luminous red galaxies from the SDSS survey to assess the quadrupole anisotropy in the primordial power spectrum of the type described by Eq. (IV.4.12). The sample is divided into eight redshift slices (from $z=0.2$ up to $z=0.6$), and within each slice the galaxy angular power spectrum is analysed. They also accounted for possible systematic effects (such as a modulation of the signal and noise due to a slow variation of the photometric calibration errors across the survey) and redshift-space distortion effects. In this case (Pullen and Hirata 2010)

IV.4.14 $$\begin{aligned} C_g\left( \mathbf {n},\mathbf {n'}\right) = \langle \delta _g(\mathbf {n}) \delta _g(\mathbf {n'}) \rangle= & {} \sum _l \frac{2l+1}{4\pi } C_{g,l}P_l\left( \mathbf {n} \cdot \mathbf {n'}\right) \nonumber \\&+\sum _{LM}\sum _{lml'm'}D_{g,ll'}^{LM}X_{lml'm'}^{LM}R_{lm}(\mathbf {n})R_{l'm'}\left( \mathbf {n'}\right) .\nonumber \\ \end{aligned}$$

Here, the set of $C_{g,l}$s are given by the usual galaxy angular power spectrum for the case of statistical isotropy. Statistical anisotropy produces the second term

IV.4.15 $$\begin{aligned} D_{g,ll'}^{LM}={\mathrm {i}}^{l-l'}\frac{2}{\pi }\int _0^\infty {\mathrm {d}}k\,k^2 P_g(k)g_{LM}W_l(k)W_{l'}(k), \end{aligned}$$

where $X_{lml'm'}^{LM}$ are geometric coefficients related to Wigner $3-j$ symbols, *R* denotes the real spherical harmonics (see Eqs. (3) and (13) of Pullen and Kamionkowski (2007) for more details), $P_g(k)=b^2_g P(k)$ is the isotropic galaxy power spectrum and $W_l(k) = \int \mathrm {d}\chi f(\chi )j_l(k\chi )$ is the window function ($\chi$ is the comoving distance, and $f(\chi )$ is the selection function, i.e., the normalized redshift distribution for a redshift slice).

Assuming the same preferred direction singled out by Groeneboom and Eriksen (2009), they derive a constraint on the anisotropy amplitude $g_*=0.006 \pm 0.036$ ($1 \sigma$), thus finding no evidence for anisotropy. Marginalizing over $\mathbf {n}$ with a uniform prior they find $-0.41< g_* < 0.38$ at 95% C.L. These results could confirm that the signal seen in CMB data is of systematic nature. However, it must be stressed that CMB and LSS analyses probe different scales, and in general the amplitude of the anisotropy is scale dependent $g=g(k)$, as in the model proposed in Erickcek et al. (2009). An estimate for what an experiment like Euclid can achieve is to consider how the uncertainty in $g_*$ scale in terms of number of modes measured and the number of redshift slices. Following the arguments of Pullen and Hirata (2010), the uncertainty will scale roughly as $\ell _{\max }^{-1} N_z^{-1/2}$, where $\ell _{\max }$ is the maximum multipole at which the galaxy angular power spectrum is probed, and $N_z$ is the number of redshift slices. Considering that the redshift survey of Euclid will cover redshifts $0.4< z <2$, there is an increase by a factor of 3 in distance of the survey and hence a factor 3 increase in $l_{\max }$ [$l_{\max } \sim k_{\max } \chi (z)$, see the expression for the selection function after Eq. (IV.4.15)]. Taking $k_{\max }= 0.2h\, {\mathrm {Mpc}}^{-1}$ the effective number of redshift slices is also increased of a factor of $\sim 3$ ($N_z \sim k_{\max } \ \varDelta \chi /\pi$, with $\varDelta \chi$ the radial width of the survey). Therefore, one could expect that for a mission like Euclid one can achieve an uncertainty (at $1\sigma$) $\sigma _{g_*} \sim 10^{-3}\textendash 10^{-2}$ or $\sigma _{g_*} \sim 10^{-2}$, for a fixed anisotropy axis or marginalizing over $\mathbf {n}$, respectively. This will be competitive with Planck measurements and highly complementary to it (Paci et al. 2010; Gruppuso et al. 2011). Notice that these constraints apply to an analysis of the galaxy angular power spectrum. An analysis of the 3-dimensional power spectrum $P(\mathbf {k})$ could improve the sensitivity further. In this case the uncertainty would scale as $\varDelta g_* \sim N^{-1/2}_{\mathrm {modes}}$, where $N_{\mathrm {modes}}$ is the number of independent Fourier modes.

## Part V Statistical methods for performance forecasts

### Introduction

As cosmology becomes increasingly dominated by results emerging from large-scale observational programmes, it is imperative to be able to justify that resources are being deployed as effectively as possible. In recent years it has become standard to quantify the expected outcome of cosmological surveys to enable comparison, a procedure exemplified by the Figure of Merit (FoM) introduced by Huterer and Turner (2001) and later used in the influential Dark Energy Task Force (DETF) report about dark-energy surveys (Albrecht et al. 2006, 2009).

The idea is to be able to capture in one single number the scientific return of a future mission, in order to be able to rank competing proposals and to forecast their ability to answer relevant scientific questions, such as: is dark energy a cosmological constant or does it evolve with time? Is it an expression of modified gravity? How well can a time-evolution of dark energy be constrained?

Encapsulating the entire value of a proposed cosmological survey in one single number is of course highly reductive, and the ensuing conclusions should therefore be taken with a large grain of salt. Having said that, work in recent years has focused on attempts to devise Figures of Merit (FoMs) that represent in an increasingly realistic way future missions. It is perhaps obvious that, to a certain extent, the assessment of a future probe will depend on the scientific question one is most interested in: parameter constraints, model selection, robustness to systematics are but a few examples of the different levels on which a proposed mission can be evaluated and optimized.

This gives an overview of some of the approaches recently adopted in the field, and used elsewhere in this document to produce forecasts for Euclid. Useful references and background material to some of the concepts discussed below are: Trotta (2008), Hobson et al. (2010) for an introduction to Bayesian methods in cosmology, Sivia (1996), MacKay (2003) for introductions to the Bayesian approach in data analysis, Gilks et al. (1996) for an introduction to Markov Chain Monte Carlo (MCMC) methods.

### Predicting the science return of a future experiment

**Fig. 62:**
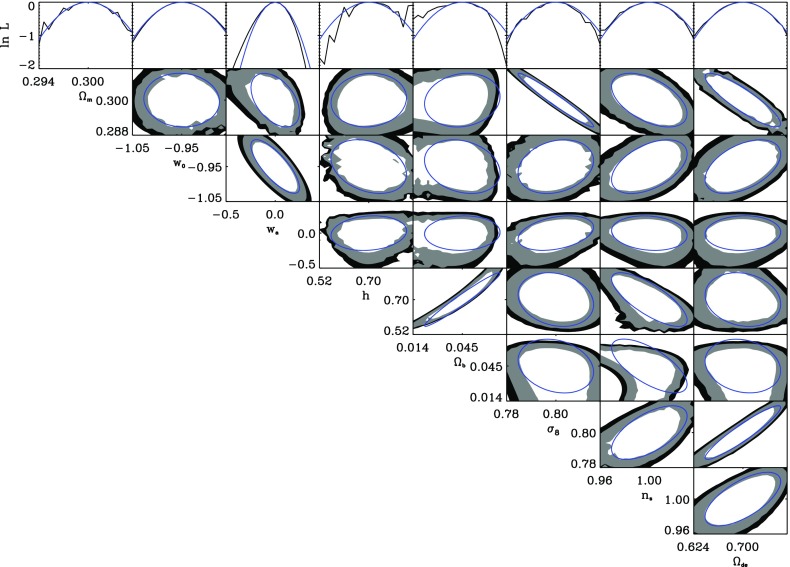
Projected cosmological 8-parameter space for a 15,000 square degrees, median redshift of $z=0.8$, 10 bin tomographic cosmic shear survey. Specifications are based on Euclid Yellow book (Laureijs et al. 2009) as this figure is representative of a method, rather than on forecast analysis; the discussion is still valid with more updated (Laureijs et al. 2011) Euclid specifications. The upper panel shows the 1D parameter constraints using analytic marginalization (black) and the Gaussian approximation (Fisher matrix, blue, dark grey). The other panels show the 2D parameter constraints. Grey contours are 1- 2- and 3-$\sigma$ levels using analytic marginalization over the extra parameters, solid blue ellipses are the 1-$\sigma$ contours using the Fisher-matrix approximation to the projected likelihood surface, solid red ellipses are the 1-$\sigma$ fully marginalized. Image reproduced by permission from Taylor and Kitching (2010)

**Fig. 63:**
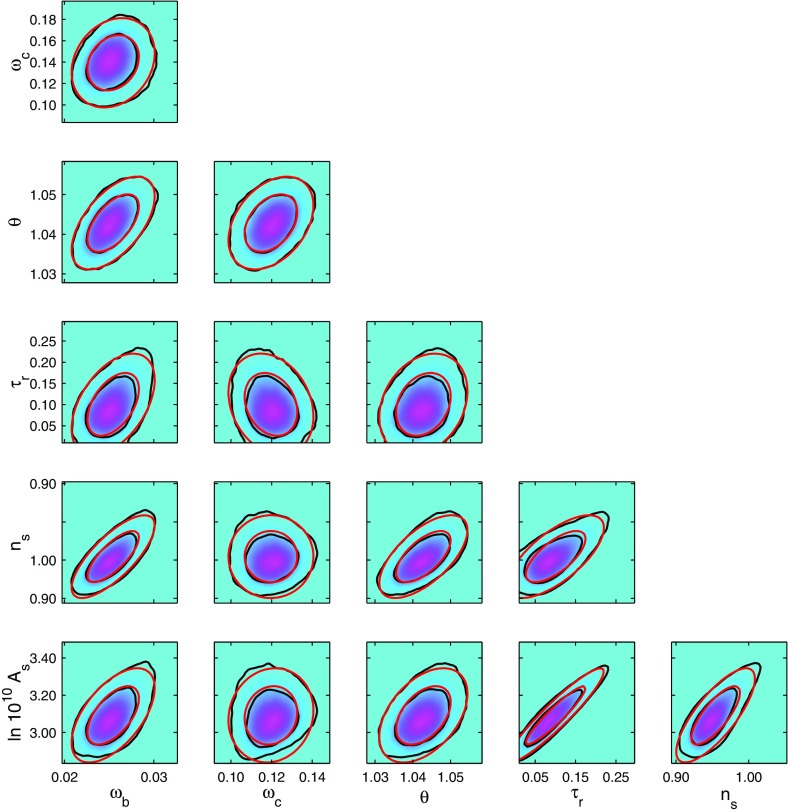
Gaussian approximation (Laplace approximation) to a 6-dimensional posterior distribution for cosmological parameters, from WMAP1 and SDSS data. For all couples of parameters, panels show contours enclosing 68 and 95% of joint probability from $2\times 10^5$ MC samples (black contours), along with the Laplace approximation (red ellipses). It is clear that the Laplace approximation captures the bulk of the posterior volume in parameter space in this case where there is little non-Gaussianity in the posterior PDF. Image reproduced from 2005 preprint of Trotta (2007a)

We consider a toy Gaussian linear model in order to illustrate the different approaches to performance forecast. We notice that, although motivated by computational simplicity and the ability to obtain analytical results, a Gaussian model is actually a fairly close representation of many cases of interest. In Fig. 62 we illustrate this point by plotting the parameter constraints expected from a Euclid-like survey and the corresponding Gaussian approximation in the Fisher-matrix approach to the likelihood (described below). In these cases, it seem clear that the Gaussian model captures fairly well the full probability distribution. Another example shown in Fig. 63 are cosmological constraints from WMAP and SDSS data, where a Gaussian approximation to the likelihood (the so-called Laplace approximation) is seen to give an excellent description of the full distribution obtained numerically via MCMC.

#### The Gaussian linear model

Suppose we have *N* cosmological probes, whose likelihood function is assumed to be a multi-dimensional Gaussian, given by:

V.2.1 $$\begin{aligned} \mathcal {L}_i ({\varTheta }) \equiv p(D_i |\varvec{\Theta })= \mathcal {L}_{0}^{i}\exp \left( -\frac{1}{2}(\varvec{\mu }_{i}-{\varTheta })^{t}C^{-1}_{i}(\varvec{\mu }_{i}-\varvec{\Theta })\right) . \end{aligned}$$

where $\mathbf \varTheta$ are the parameters one is interested in constraining, $D_i$ are the available data from probe *i* and $\mathbf \mu _i$ is the location of the maximum likelihood value in parameter space. The matrix is the covariance matrix of the parameters.

The posterior distribution for the parameters from each probe, $p({\varTheta }|D_i)$, is obtained by Bayes’ theorem as

V.2.2 $$\begin{aligned} p({\varTheta }|D_i)=\frac{p(\varvec{\Theta })p \left( D_i|\varvec{\Theta }\right) }{p(D_i)}, \end{aligned}$$

where and $p({\varTheta })$ is the prior and $p(D_i)$ is a normalizing constant (the Bayesian evidence). If we assume a Gaussian prior centered around the maximum likelihood value with inverse covariance matrix $\varSigma$, the posterior from each probe is also a Gaussian, with inverse covariance matrix

V.2.3 $$\begin{aligned} F_i = C^{-1}_i + \varSigma \end{aligned}$$

and posterior mean

V.2.4 $$\begin{aligned} \overline{\mu }_{i} = F_{i}^{-1}\left( C^{-1}_{i}\mu _{i}\right) . \end{aligned}$$

Tighter constraints on the parameters can be usually obtained by combining all available probes together (provided there are no systematics, see below). If we combine all probes together (assuming they are independent), we obtain a Gaussian posterior with inverse covariance matrix

V.2.5 $$\begin{aligned} F = \sum _{i=1}^N C^{-1}_i + \varSigma \end{aligned}$$

and mean

V.2.6 $$\begin{aligned} \overline{\mu } =F^{-1}\sum _{i=1}^N C^{-1}_{i}\mu _{i}. \end{aligned}$$

Notice that the precision of the posterior (i.e., the inverse covariance matrix) does not depend on the degree of overlap of the likelihoods from the individual probes. This is a property of the Gaussian linear model.

For future reference, it is also useful to write down the general expression for the Bayesian evidence. For a normal prior $p({\varTheta }) \propto {\mathcal {N}}(\theta _{\pi },\varSigma )$ and a likelihood

V.2.7 $$\begin{aligned} \mathcal {L}({\varTheta })=\mathcal {L}_{0} \exp \left( -\frac{1}{2}(\theta _{0}-{\varTheta })^{t}L(\theta _{0}-\varvec{\Theta })\right) ,\end{aligned}$$

the evidence for data *d* is given by

V.2.8 $$\begin{aligned} p(d) \equiv \int {\mathrm {d}}{\varTheta }p(d|\varvec{\Theta })p(\varvec{\Theta })= \mathcal {L}_{0}\frac{|\varSigma |^{1/2}}{|F|^{1/2}} \exp \left[ -\frac{1}{2}\left( \theta _{0}^{t}L\theta _{0}+ \theta _{\pi }^{t}\varSigma \theta _{\pi }-\overline{\theta }^{t}F\overline{\theta }\right) \right] ,\nonumber \\ \end{aligned}$$

where *F* is given by Eq. (V.2.5) with $N=1$ and $\overline{\theta } = F^{-1}L\theta _0$.

#### Fisher-matrix error forecast

A general likelihood function for a future experiment (subscript *i*) can be Taylor-expanded around its maximum-likelihood value, $\mu _i$. By definition, at the maximum the first derivatives vanish, and the shape of the log-likelihood in parameter space is approximated by the Hessian matrix $H_i$,

V.2.9 $$\begin{aligned} \ln \mathcal {L}_i({\varTheta }) \approx \ln \mathcal {L}_i(\mu _i) + \frac{1}{2}({\varTheta }-\mu _i)^t H_i ({\varTheta }-\mu _i), \end{aligned}$$

where $H_i$ is given by

V.2.10 $$\begin{aligned} \left( H_i\right) _{\alpha \beta } \equiv \frac{\partial ^2 \ln \mathcal {L}_i}{\partial {\varTheta }_\alpha \partial {\varTheta }_\beta } {{\Big \arrowvert }_{\mu _i}}, \end{aligned}$$

and the derivatives are evaluated at the maximum-likelihood point. By taking the expectation of equation (V.2.9) with respect to many data realizations, we can replace the maximum-likelihood value $\mu _i$ with the true value, ${\varTheta }_*$, as the maximum-likelihood estimate is unbiased (in the absence of systematics), i.e., $\langle \mu _i \rangle = {\varTheta }_*$. We then define the Fisher information matrix as the expectation value of the Hessian,

V.2.11 $$\begin{aligned} F_i \equiv \langle H_i \rangle . \end{aligned}$$

The inverse of the Fisher matrix, $F^{-1}$, is an estimate of the covariance matrix for the parameters, and it describes how fast the log-likelihood falls (on average) around the maximum likelihood value, and we recover the Gaussian expression for the likelihood, Eq. (V.2.1), with the maximum likelihood value replaced by the true value of the parameters and the inverse covariance matrix given by the Fisher matrix, $L_i = F_i^{-1}$ (Kendall and Stuart 1977). In general, the derivatives depend on where in parameter space we take them (except for the simple case of linear models), hence it is clear that $F_i$ is a function of the fiducial parameters.

Once we have the Fisher matrix, we can give estimates for the accuracy on the parameters from a future measurement, by computing the posterior as in Eq. (V.2.2). If we are only interested in a subset of the parameters, then we can marginalize easily over the others: computing the Gaussian integral over the unwanted parameters is the same as inverting the Fisher matrix, dropping the rows and columns corresponding to those parameters (keeping only the rows and columns containing the parameters of interest) and inverting the smaller matrix back. The result is the marginalized Fisher matrix $\mathcal {F}_i$. For example, the $1\sigma$ error for parameter $\alpha$ from experiment *i*, marginalized over all other parameters, is simply given by $\sigma _\alpha = \sqrt{\left( F_i^{-1}\right) _{\alpha \alpha }}$.

It remains to compute the Fisher matrix for the future experiment. This can be done analytically for the case where the likelihood function is approximately Gaussian in the data, which is a good approximation for many applications of interest. We can write for the log-likelihood (in the following, we drop the subscript *i* denoting the experiment under consideration for simplicity of notation)

V.2.12 $$\begin{aligned} -2 \ln \mathcal {L}= \ln |C| + (D-\mu )^t C^{-1} (D-\mu ), \end{aligned}$$

where *D* are the (simulated) data that would be observed by the experiment and in general both the mean $\mu$ and covariance matrix *C* may depend on the parameters ${\varTheta }$ we are trying to estimate. The expectation value of the data corresponds to the true mean, $\langle D \rangle = \mu$, and similarly the expectation value of the data matrix $\varDelta \equiv (D-\mu )^t(D-\mu )$ is equal to the true covariance, $\langle \varDelta \rangle = C$. Then it can be shown (see, e.g., Tegmark et al. 1997) that the Fisher matrix is given by

V.2.13 $$\begin{aligned} F_{\alpha \beta } = \frac{1}{2} {\mathrm {tr}}\left[ A_{\alpha } A_{\beta } + C^{-1} \langle \varDelta _{,\alpha \beta } \rangle \right] , \end{aligned}$$

where $A_\alpha \equiv C^{-1} C_{,\alpha }$ and the comma denotes a derivative with respect to the parameters, for example $C_{,\alpha } \equiv \partial C/\partial {\varTheta }_\alpha$. The fact that this expression depends only on *expectation values* and not on the particular data realization means that the Fisher matrix can be computed from knowledge of the noise properties of the experiment without having to go through the step of actually generating any simulated data. The specific form of the Fisher matrix then becomes a function of the type of observable being considered and of the experimental parameters.

Explicit expressions for the Fisher matrix for cosmological observables can be found in Tegmark et al. (1997) for cosmic microwave background data, in Tegmark (1997) for the matter power spectrum from galaxy redshift surveys (applied to baryonic acoustic oscillations in Seo and Eisenstein (2003) and Hu and Jain (2004) for weak lensing. These approaches have been discussed in Sect. I.7. A useful summary of Fisher matrix technology is given in the Dark Energy Task Force report (Albrecht et al. 2006) and in Verde (2010b). A useful numerical package which includes several of the above calculations is the publicly available Matlab code^29^ Fisher4Cast (Bassett et al. 2011, 2009). Attempts to include systematic errors modelling in this framework can be found in Kitching and Taylor (2011), Taylor and Kitching (2010) and Kitching et al. (2009, 2008b).

For Gaussian data, the Fisher matrix can be obtained also by expanding the mean $\mu$ and the correlation matrix *C* to first order in a Taylor series around the posterior peak in parameter space. The credible regions obtained from the Fisher matrix approach are a good approximation to the exact results only when this linear expansion is acceptable. A way to test for this assumption and to correct it when it fails is obtained by expanding the mean and correlation to higher orders. If the parameters are only in the mean, one can show that the posterior remains normalizable at all orders; additional conditions are needed in the more general case. Expanding to second order in the derivatives one obtains (Sellentin et al. 2014)

V.2.14 $$\begin{aligned} \begin{aligned} \mathrm {\mathcal {L}}({\varTheta })&=N\exp \bigg [ \left. -\frac{1}{2}\varvec{\mu }_{,\alpha }C^{-1}\varvec{\mu }_{,\beta }\varDelta \theta _{\alpha }\varDelta \theta _{\beta }-\frac{1}{2}\varvec{\mu }_{,\alpha \beta }C^{-1}\varvec{\mu }_{,\gamma }\varDelta \theta _{\alpha }\varDelta \theta _{\beta }\varDelta \theta _{\gamma }\right. \\&\qquad -\frac{1}{8}\varvec{\mu }_{,\delta \gamma }C^{-1}\varvec{\mu }_{,\beta \alpha }\varDelta \theta _{\alpha }\varDelta \theta _{\beta }\varDelta \theta _{\gamma }\varDelta \theta _{\delta }\,\bigg ], \end{aligned} \end{aligned}$$

where $\varDelta \theta _{\alpha }={\varTheta }_{\alpha }-\theta _{\alpha }$. The first term is the standard Fisher term (when the parameters are only in the mean), the other terms express the deviations from Gaussianity in parameter space. As long as the deviation from Gaussianity is not too large, the additional terms describe the common “banana” shaped posteriors often encountered in the data analysis. Clearly, if these terms dominate over the Fisher term then a full exploration of the posterior, for instance through Monte Carlo methods, is advisable.

#### Figure of merits

It has become customary to describe the statistical power of a future dark energy probe by the inverse area enclosed by the 68% covariance ellipse marginalized down to the dark-energy parameter space. This measure of statistical performance for probe *i* (widely known as the DETF FoM, Albrecht et al. 2006; Huterer and Turner 2001) is usually defined (up to multiplicative constants) as

V.2.15 $$\begin{aligned} \text {FoM} = |F_i|^{1/2}, \end{aligned}$$

where the Fisher matrix $F_i$ is given in Eq. (V.2.11). Albrecht et al. (2006) suggested to use the inverse area of the 95% error ellipse of $w_0-w_a$ (where $w_0$ and $w_a$ are defined in Linder 2003; Chevallier and Polarski 2001). This definition was inspired by Huterer and Turner (2001). In Albrecht et al. (2009), it is suggested to model *w*(*a*) as piecewise constant values of *w*(*a*) defined in many small redshift bins ($\varDelta a = 0.025$). The suggestion is then to apply a principal component approach (Huterer and Starkman 2003) in order to understand the redshifts at which each experiment has the power to constrain *w*.

A closely related but more statistically motivated measure of the information gain is the Kullback–Leibler divergence (KL) between the posterior and the prior, representing the information gain obtained when upgrading the prior to the posterior via Bayes’ theorem:

V.2.16 $$\begin{aligned} D_{\mathrm {KL}} \equiv \int p({\varTheta }|D)\ln \frac{p(\varvec{\Theta }|D)}{p(\varvec{\Theta })}d\varvec{\Theta }. \end{aligned}$$

The KL divergence measures the relative entropy between the two distributions: it is a dimensionless quantity which expresses the information gain obtained via the likelihood. For the Gaussian likelihood and prior introduced above, the information gain (w.r.t. the prior $\varSigma$) from the combination of all probes is given by Trotta et al. (2010)

V.2.17 $$\begin{aligned} D_{\mathrm {KL}}=\frac{1}{2}\left( \ln |F|-\ln |\varSigma |-{{\mathrm {tr}}[1-\varSigma F^{-1}]}\right) . \end{aligned}$$

A discussion of other, alternative FoMs (D-optimality, A-optimality) can be found in Bassett (2005). In Wang (2008b), a different FoM for dark energy is suggested. For a set of DE parameters ${\varTheta }$, the FoM is defined as $\mathrm {FoM} = 1/\sqrt{\det C({\varTheta })}$, where $C({\varTheta })$ is the covariance matrix of ${\varTheta }$. This definition is more flexible since one can use it for any DE parametrization (Wang et al. 2010).

Given that Euclid can constrain both the expansion history and the growth of structure, it is also useful to introduce a new FoM for the growth of perturbations. Similarly to the DETF FoM, one can define this new FoM as the inverse area of the 95% error ellipse of $\varOmega _m-\gamma$, where $\gamma$ is the growth index, defined starting from the growth rate $f_G(z) \equiv \frac{d\ln G(z)}{d\ln a} = \varOmega _m^\gamma$, or as $1/\sqrt{\det C(w_0,w_a,\gamma )}$ or similar variants (Majerotto et al. 2012; Di Porto et al. 2012). Instead of $\gamma$, other parameters describing the growth can also be employed.

A FoM targeted at evaluating the robustness of a future probe to potential systematic errors has been introduced in March et al. (2011b). The robustness of a future probe is defined via the degree of overlap between the posterior distribution from that probe and the posterior from other, existing probes. The fundamental notion is that maximising statistical power (e.g., by designing a future probe to deliver orthogonal constraints w.r.t. current probes) will in general reduce its robustness (by increasing the probability of an incompatible results, for example because of systematic bias). Thus in evaluating the strength of a probe, both its statistical power and its resilience to plausible systematics ought to be considered.

#### The Bayesian approach

When considering the capabilities of future experiments, it is common stance to predict their performance in terms of constraints on relevant parameters, assuming a fiducial point in parameter space as the true model (often, the current best-fit model), as explained above. While this is a useful indicator for parameter inference tasks, many questions in cosmology fall rather in the model comparison category. Dark energy is a case in point, where the science driver for many future probes (including Euclid) is to detect possible departures from a cosmological constant, hence to gather evidence in favor of an evolving dark-energy model. Therefore, it is preferable to assess the capabilities of future experiments by their ability to answer model selection questions.

The procedure is as follows (see Mukherjee et al. 2006 for details and the application to dark-energy scenarios). At every point in parameter space, mock data from the future observation are generated and the Bayes factor between the competing models is computed, for example between an evolving dark energy and a cosmological constant. Then one delimits in parameter space the region where the future data would *not* be able to deliver a clear model comparison verdict, for example $\vert \ln B_{01} \vert < 5$ (evidence falling short of the “strong” threshold). Here, $B_{01}$ is the Bayes factor, which is formed from the ratio of the Bayesian evidences of the two models being considered:

V.2.18 $$\begin{aligned} B_{01} = \frac{p(d| \mathcal {M}_0)}{p(d| \mathcal {M}_1)}, \end{aligned}$$

where the Bayesian evidence is the average of the likelihood under the prior in each model (denoted by a subscript *m*):

V.2.19 $$\begin{aligned} p(d| \mathcal {M}_m) = \int \mathrm {d}{\varTheta }_m p(d| {\varTheta }_m, \mathcal {M}_m)p({\varTheta }_m | \mathcal {M}_m). \end{aligned}$$

The Bayes factor updates the prior probability ratio of the models to the posterior one, indicating the extent to which the data have modified one’s original view on the relative probabilities of the two models. The experiment with the smallest “model-confusion” volume in parameter space is to be preferred, since it achieves the highest discriminative power between models. An application of a related technique to the spectral index from the Planck satellite is presented in Pahud et al. (2006, 2007).

Alternatively, we can investigate the full probability distribution for the Bayes factor from a future observation. This allows to make probabilistic statements regarding the outcome of a future model comparison, and in particular to quantify the probability that a new observation will be able to achieve a certain level of evidence for one of the models, given current knowledge. This technique is based on the *predictive distribution* for a future observation, which gives the expected posterior for an observation with a certain set of experimental capabilities (further details are given in Trotta 2007b). This method is called PPOD, for *predictive posterior odds distribution* and can be useful in the context of experiment design and optimization

Hybrid approaches have also been attempted, i.e., to defined model-selection oriented FoMs while working in the Fisher-matrix framework, such as the Bayes factor (Heavens et al. 2007; Amara and Kitching 2010).

The most general approach to performance forecasting involves the use of a suitably defined utility function, and it has recently been presented in Trotta et al. (2011). Consider the different levels of uncertainty that are relevant when predicting the probability of a certain model selection outcome from a future probe, which can be summarized as follows:

- **Level 1:** current uncertainty about the correct model (e.g., is it a cosmological constant or a dark-energy model?).
- **Level 2:** present-day uncertainty in the value of the cosmological parameters for a given model (e.g., present error on the dark-energy equation of state parameters assuming an evolving dark-energy model).
- **Level 3:** realization noise, which will be present in future data even when assuming a model and a fiducial choice for its parameters.

The commonly-used Fisher matrix forecast ignores the uncertainty arising from Levels 1 and 2, as it assumes a fiducial model (Level 1) and fiducial parameter values (Level 2). It averages over realization noise (Level 3) in the limit of an infinite number of realizations. Clearly, the Fisher matrix procedure provides a very limited assessment of what we can expect for the scientific return of a future probe, as it ignores the uncertainty associated with the choice of model and parameter values.

The Bayesian framework allows improvement on the usual Fisher matrix error forecast thanks to a general procedure which fully accounts for all three levels of uncertainty given above. Following Loredo (2004), we think of future data ${D}_f$ as *outcomes*, which arise as consequence of our choice of experimental parameters *e* (*actions*). For each action and each outcome, we define a utility function $\mathcal {U}({D}_f, e)$. Formally, the utility only depends on the future data realization ${D}_f$. However, as will become clear below, the data ${D}_f$ are realized from a fiducial model and model parameter values. Therefore, the utility function implicitly depends on the assumed model and parameters from which the data ${D}_f$ are generated. The best action is the one that maximizes the expected utility, i.e., the utility averaged over possible outcomes:

V.2.20 $$\begin{aligned} \mathcal {EU}(e) \equiv \int \mathrm {d}{D}_fp({D}_f| e, d) \mathcal {U}({D}_f, e). \end{aligned}$$

Here, $$p({D}_f| e, d) $$ is the predictive distribution for the future data, conditional on the experimental setup (*e*) and on current data ($$d$$). For a single fixed model the predictive distribution is given by

V.2.21 $$\begin{aligned} p({D}_f| e, d)&= \int \mathrm {d}{\varTheta }\, p({D}_f, {\varTheta }| e, d) \nonumber \\&= \int \mathrm {d}{\varTheta }\, p({D}_f| {\varTheta }, e, d) p({\varTheta }|e,d) \nonumber \\&= \int \mathrm {d}{\varTheta }\, p({D}_f| {\varTheta }, e) p({\varTheta }|d), \end{aligned}$$

where the last line follows because $p({D}_f| {\varTheta }, e, d) = p({D}_f| {\varTheta }, e)$ (conditioning on current data is irrelevant once the parameters are given) and $p({\varTheta }|e,d) = p({\varTheta }| d)$ (conditioning on future experimental parameters is irrelevant for the present-day posterior). So we can predict the probability distribution for future data ${D}_f$ by averaging the likelihood function for the future measurement (Level 3 uncertainty) over the current posterior on the parameters (Level 2 uncertainty). The expected utility then becomes

V.2.22 $$\begin{aligned} \mathcal {EU}(e) = \int \mathrm {d}{\varTheta }p({\varTheta }| o, d) \int \mathrm {d}{D}_fp({D}_f| {\varTheta }, e) \mathcal {U}({D}_f, e). \end{aligned}$$

So far, we have tacitly assumed that only one model was being considered for the data. In practice, there will be several models that one is interested in testing (Level 1 uncertainty), and typically there is uncertainty over which one is best. This is in fact one of the main motivations for designing a new dark energy probe. If *M* models $\{ \mathcal {M}_1, \ldots , \mathcal {M}_M \}$ are being considered, each one with parameter vector ${\varTheta }_m$ ($m=1,\ldots , M$), the current posterior can be further extended in terms of model averaging (Level 1), weighting each model by its current model posterior probability, $p(\mathcal {M}_m | d)$, obtaining from Eq. (V.2.22) the model-averaged expected utility

V.2.23 $$\begin{aligned} \mathcal {EU}(e) =&\sum _{m=1}^M p(\mathcal {M}_m| d) \int \mathrm {d}{\varTheta }_m p({\varTheta }_m| d,\mathcal {M}_m) \nonumber \\&\times \int \mathrm {d}{D}_fp({D}_f| {\varTheta }_m, e,\mathcal {M}_m) \mathcal {U}({D}_f, e,\mathcal {M}_m). \end{aligned}$$

This expected utility is the most general definition of a FoM for a future experiment characterized by experimental parameters *e*. The usual Fisher matrix forecast is recovered as a special case of Eq. (V.2.23), as are other ad hoc FoMs that have been defined in the literature. Therefore Eq. (V.2.23) gives us a formalism to define in all generality the scientific return of a future experiment. This result clearly accounts for all three levels of uncertainty in making our predictions: the utility function $\mathcal {U}({D}_f, e,\mathcal {M}_m)$ (to be specified below) depends on the future data realization, ${D}_f$, (Level 3), which in turn is a function of the fiducial parameters value, ${\varTheta }_m$, (Level 2), and is averaged over present-day model probabilities (Level 1).

This approach is used in Trotta et al. (2011) to define two model-selection oriented Figures of Merit: the decisiveness $\mathcal {D}$, which quantifies the probability that a probe will deliver a decisive result in favor or against the cosmological constant, and the expected strength of evidence, $\mathcal {E}$, that returns a measure of the expected power of a probe for model selection.

### Survey design and optimization

Although the topic of survey design is still in its infancy, the basic idea is to carry out an optimization of survey parameters (such as for example choice of targets, depth of field, number of spectroscopic fibers, etc.) in order to identify the configuration that is more likely to return a high FoM for the scientific question being considered. Example of this approach applied to dark-energy parameters can be found in Bassett (2005), Parkinson et al. (2007, 2010), Bassett et al. (2005), while Loredo (2004) discussed a more general methodology. In Bassett (2005), a method is defined to optimize future surveys, in the framework of Bayesian statistics and without necessarily assuming a dark-energy model. In Bassett et al. (2005), Parkinson et al. (2007), and Parkinson et al. (2010) this method is used to produce forecasts for future weak lensing and galaxy redshift surveys.

The optimization process is carried out subject to constraints, such as for example design parameter ranges and/or cost constraints. This is generally a numerically complex and computationally expensive procedure. It typically requires to explore the design parameters space (e.g., via MCMC), generating at each point a set of pseudo-data that are analysed as real data would, in order to compute their FoM. Then the search algorithm moves on to maximize the FoM.

In order to carry out the optimization procedure, it might be useful to adopt a principal component analysis (PCA) to determine a suitable parametrization of *w*(*z*) (Huterer and Starkman 2003; Simpson and Bridle 2006). The redshift range of the survey can be split into *N* bins, with the equation of state taking on a value $w_i$ in the *i*-th bin:

V.3.1 $$\begin{aligned} w(z) = \sum _{i = 1}^N w_i b_i(z). \end{aligned}$$

where the basis functions $b_i$ are top-hats of value 1 inside the bin, and 0 elsewhere. If *F* is the Fisher matrix for the *N* parameters $w_i$, one can diagonalize it by writing $F = W^T \varLambda W$, where $\varLambda$ is a diagonal matrix, and the rows of *W* are the eigenvectors $e_i(z)$ or the so-called principal components. These define a new basis (in which the new coefficients $\alpha _i$ are uncorrelated) so the equation of state can be written as

V.3.2 $$\begin{aligned} w(z) = \sum _{i = 1}^N \alpha _i e_i(z). \end{aligned}$$

The diagonal elements of $\varLambda$ are the eigenvalues $\lambda _i$ and define the variance of the new parameters, $\sigma ^2(\alpha _i) = 1/\lambda _i$.

One can now reconstruct *w*(*z*) by keeping only a certain number of the most accurately determined modes, i.e., the ones with largest eigenvalues. The optimal number of modes to retain can be estimated by minimizing the risk, defined as the sum of the bias squared (how much the reconstructed equation of state departs from the true one by neglecting the more noisy modes) plus the variance of the estimate (Huterer and Starkman 2003).

### Future activities and open challenges

As outlined in the previous sections, several approaches are available to capture the expected scientific performance of Euclid. As part of future theoretical activities, it will be necessary to build on the above concepts in order to obtain a realistic assessment of the science return of Euclid. Operationally, this means that the following tasks will need to be carried out:

- Estimation of likelihood contours around the maximum likelihood peak beyond the Fisher matrix approach. We envisage here a programme where simulated mock data will be generated and then used to blindly reconstruct the likelihood surface to sufficient accuracy.
- Estimation of Bayesian posterior distributions and assessment of impact of various priors. Bayesian inference is a mature field in cosmology and we now have at our disposal a number of efficient and reliable numerical algorithms based on Markov Chain Monte Carlo or nested sampling methods.
- Comparison of Bayesian inferences with frequentist inferences based on profile likelihoods. Discrepancies might occur in the presence of large “volume and sampling effects” arising from insufficiently constraining data sets and highly multi-modal likelihoods (Trotta et al. 2008). Based on our experience so far, this is unlikely to be a problem for most of the statistical quantities of interest here but we recommend to check this explicitly for the more complicated distributions.
- Investigation of the coverage properties of Bayesian credible and frequentist confidence intervals. Coverage of intervals is a fundamental property in particle physics, but rarely discussed in the cosmological setting. We recommend a careful investigation of coverage from realistically simulated data sets (as done recently in March et al. 2011c). Fast neural networks techniques might be required to speed up the inference step by several orders of magnitude in order to make this kind of studies computationally feasible (Shaw et al. 2007; Bridges et al. 2011).
- Computation of the Bayesian evidence to carry out Bayesian model selection (Trotta 2008; Mukherjee et al. 2006). Algorithms based on nested sampling, and in particular, MultiNest (Feroz and Hobson 2008), seem to be ideally suited to this task, but other approaches are available, as well, such as population Monte Carlo (Kilbinger et al. 2011) and semi-analytical ones (Trotta 2007a; Heavens et al. 2007). A robust Bayesian model selection will require a careful assessment of the impact of priors. Furthermore, the outcome of Bayesian model selection is dependent on the chosen parametrization if different nonlinearly related reparametrizations can equally plausibly be chosen from physical consideration. Relevant examples include parametrizations of the isocurvature fraction (Beltrán et al. 2005), the tensor-to-scalar ratio (Parkinson et al. 2006), and the inflaton potential (Martin et al. 2011). It will be important to cross check results with frequentist hypothesis testing, as well. The notion of Bayesian doubt, introduced in March et al. (2011a), can also be used to extend the power of Bayesian model selection to the space of unknown models in order to test our paradigm of a $\varLambda$CDM cosmological model.
- Bayesian model averaging (Liddle et al. 2006; Parkinson and Liddle 2010) can also be used to obtain final inferences parameters which take into account the residual model uncertainty. Due to the concentration of probability mass onto simpler models (as a consequence of Occam’s razor), Bayesian model averaging can lead to tighter parameter constraints than non-averaged procedures, for example on the curvature parameter (Vardanyan et al. 2011).
- In cosmology and astronomy it is intrinsically difficult to deal with systematic errors that can bias an experiment. Particularly important can be the issue of dataset contamination. For instance, spurious transients such as core-collapse supernovae could end up in SNIa catalogs with the effect of biasing the best fit (i.e. affecting the accuracy of an experiment) rather than increasing the errors (i.e. affecting the precision) (see Amendola et al. 2013b, Fig. 3). The way out is to look for deviating subpopulations in data in order to purge the dataset of possible contaminations. A possible tool for this task is the *internal robustness* introduced in Amendola et al. (2013b), which uses Bayesian evidences to assess if the dataset at hand is better described by a single cosmological model or rather by two models, a cosmological one for unbiased data and a systematical one for contaminated data. It will be necessary to develop fast algorithms in order to scan the vast datasets that will be generated by Euclid in order to find partitions that minimize the value of internal robustness.

## Authors’ contributions

### Credits version 2 (2016)

#### Euclid Theory Working Group Editorial Board (2016)

Valeria Pettorino (editor in chief)

Tessa Baker

Stefano Camera

Adrian Vollmer

Elisabetta Majerotto

Martin Kunz (Euclid Theory Working Group Coordinator)

Luca Amendola (Euclid Theory Working Group Coordinator)

### Corresponding authors (2016):

Luca Amendola

Tessa Baker

Marco Baldi

Stefano Camera

Thomas D. Kitching

Martin Kunz

Elisabetta Majerotto

Valerio Marra

Valeria Pettorino

Ignacy Sawicki

Licia Verde

### Contributing authors (2016):

Luca Amendola, Stephen Appleby, Anastasios Avgoustidis, David Bacon, Tessa Baker, Marco Baldi, Nicola Bartolo, Alain Blanchard, Camille Bonvin, Stefano Borgani, Enzo Branchini, Clare Burrage, Stefano Camera, Carmelita Carbone, Luciano Casarini, Mark Cropper, Claudia de Rham, Jörg P. Dietrich, Cinzia Di Porto, Ruth Durrer, Anne Ealet, Pedro G. Ferreira, Fabio Finelli, Juan García-Bellido, Tommaso Giannantonio, Luigi Guzzo, Alan Heavens, Lavinia Heisenberg, Catherine Heymans, Henk Hoekstra, Lukas Hollenstein, Rory Holmes, Zhiqi Hwang, Knud Jahnke, Thomas D. Kitching, Tomi Koivisto, Martin Kunz, Giuseppe La Vacca, Eric Linder, Marisa March, Valerio Marra, Carlos Martins, Elisabetta Majerotto, Dida Markovic, David Marsh, Federico Marulli, Richard Massey, Yannick Mellier, Francesco Montanari, David F. Mota, Nelson J. Nunes, Will Percival, Valeria Pettorino, Cristiano Porciani, Claudia Quercellini, Justin Read, Massimiliano Rinaldi, Domenico Sapone, Ignacy Sawicki, Roberto Scaramella, Constantinos Skordis, Fergus Simpson, Andy Taylor, Shaun Thomas, Roberto Trotta, Licia Verde, Filippo Vernizzi, Adrian Vollmer, Yun Wang, Jochen Weller, Tom Zlosnik.

### Credits version 1 (2012)

#### Euclid Theory Working Group Editorial Board (2012)

Valeria Pettorino (editor in chief)

Tessa Baker

Stefano Camera

Elisabetta Majerotto

Marisa March

Cinzia Di Porto

Martin Kunz (Euclid Theory Working Group Coordinator)

Luca Amendola (Euclid Theory Working Group Coordinator)

### Corresponding authors (2012):

Luca Amendola

Stefano Camera

Cinzia Di Porto

Pedro G. Ferreira

Juan García-Bellido

Thomas D. Kitching

Martin Kunz

Valeria Pettorino

Cristiano Porciani

Roberto Trotta

Licia Verde

Yun Wang

### Contributing authors (2012):

Luca Amendola, Stephen Appleby, David Bacon, Tessa Baker, Marco Baldi, Nicola Bartolo, Alain Blanchard, Camille Bonvin, Stefano Borgani, Enzo Branchini, Clare Burrage, Stefano Camera, Carmelita Carbone, Luciano Casarini, Mark Cropper, Claudia de Rham, Cinzia Di Porto, Anne Ealet, Pedro G. Ferreira, Fabio Finelli, Juan Garía-Bellido, Tommaso Giannantonio, Luigi Guzzo, Alan Heavens, Lavinia Heisenberg, Catherine Heymans, Henk Hoekstra, Lukas Hollenstein, Rory Holmes, Ole Horst, Zhiqi Hwang, Knud Jahnke, Thomas D. Kitching, Tomi Koivisto, Martin Kunz, Giuseppe La Vacca, Marisa March, Elisabetta Majerotto, Dida Markovic, David Marsh, Federico Marulli, Richard Massey, Yannick Mellier, David F. Mota, Nelson J. Nunes, Will Percival, Valeria Pettorino, Cristiano Porciani, Claudia Quercellini, Justin Read, Massimiliano Rinaldi, Domenico Sapone, Roberto Scaramella, Constantinos Skordis, Fergus Simpson, Andy Taylor, Shaun Thomas, Roberto Trotta, Licia Verde, Filippo Vernizzi, Adrian Vollmer, Yun Wang, Jochen Weller, Tom Zlosnik.

## Abbreviations

AGN: Active galactic nucleus
ALP: Axio-like particle
BAO: Baryonic acoustic oscillations
BBKS: Bardeen–Bond–Kaiser–Szalay
BOSS: Baryon oscillation spectroscopic survey
BPol: B-polarization satellite
BigBOSS: Baryon oscillation spectroskopic survey
CAMB: Code for anisotropies in the microwave background
CDE: Coupled dark energy
CDM: Cold dark matter
CDMS: Cryogenic dark matter search
CL: Confidence level
CLASS: Cosmic linear anisotropy solving system
CMB: Cosmic microwave background
COMBO-17: Classifying objects by medium-band observations
COSMOS: Cosmological evolution survey
CPL: Chevallier–Polarski–Linder
CQ: Coupled quintessence
CRESST: Cryogenic rare event search with superconducting thermometers
DE: Dark energy
DES: Dark energy survey
DETF: Dark energy task force
DGP: Dvali–Gabadadze–Porrati
DM: Dark matter
EBI: Eddington–Born–Infeld
EDE: Early dark energy
EMT: Energy–momentum tensor
EROS: Expérience pour la recherche d’objets Sombres
eROSITA: Extended ROentgen survey with an imaging telescope array
FCDM: Fuzzy cold dark matter
FFT: Fast Fourier transform
FLRW: Friedmann–Lemaître–Robertson–Walker
FoM: Figure of merit
FoG: Fingers of god
GEA: Generalized Einstein-aether
GR: General relativity
HETDEX: Hobby–Eberly telescope dark energy experiment
ICM: Intracluster medium
IH: Inverted hierarchy
IR: Infrared
ISW: Integrated Sachs–Wolfe
KL: Kullback–Leibler divergence
LCDM: Lambda cold dark matter
LHC: Large hadron collider
LRG: Luminous red galaxy
LSB: Low surface brightness
LSS: Large scale structure
LSST: Large synoptic survey telescope
LTB: Lemaître–Tolman–Bondi
MACHO: Massive compact halo object
MCMC: Markov Chain Monte Carlo
MCP: Mini-charged particles
MF: Mass function
MG: Modified gravity
MOND: Modified Newtonian dynamics
MaVaNs: Mass varying neutrinos
NFW: Navarro–Frenk–White
NH: Normal hierarchy
PCA: Principal component analysis
PDF: Probability distribution function
PGB: Pseudo-Goldstein Boson
PKDGRAV: Parallel K–D tree GRAVity code
PPF: Parameterized post-Friedmann
PPN: Parameterized post-Newtonian
PPOD: Predictive posterior odds distribution
PSF: Point spread function
QCD: Quantum chromodynamics
RDS: Redshift space distortions
RG: Renormalization group
SD: Savage–Dickey
SDSS: Sloan digital sky survey
SIDM: Self interacting dark matter
SN: Supernova
TeVeS: Tensor vector scalar
UDM: Unified dark matter
UV: Ultra Violett
WDM: Warm dark matter
WFXT: Wide-field X-ray telescope
WIMP: Weakly interacting massive particle
WKB: Wentzel–Kramers–Brillouin
WL: Weak lensing
WLS: Weak lensing survey
WMAP: Wilkinson microwave anisotropy probe
XMM-Newton: X-ray multi-mirror mission
vDVZ: van Dam–Veltman–Zakharov

## List of symbols

$c_a$: Adiabatic sound speed
$D_A(z)$: Angular diameter distance
Angular spin raising operator
$\varPi ^i_j$: Anisotropic stress perturbation tensor
$$\sigma $$: Uncertainty
*Bo*: Bayes factor
*b*: Bias (ratio of galaxy to total matter perturbations)
$B_\varPhi (k_1,k_2,k_3)$: Bispectrum of the Bardeen’s potential
*g*(*X*): Born–Infeld kinetic term
$\zeta$: Comoving curvature perturbation
*r*(*z*): Comoving distance
$\mathcal {H}$: Conformal Hubble parameter, $\mathcal {H} = aH$
$$\eta ,\tau $$: Conformal time
$$\kappa $$: Convergence
*t*: Cosmic time
$$\varLambda $$: Cosmological constant
$$\varvec{\Theta }$$: Cosmological parameters
$r_c$: Cross over scale
$$\square $$: d’Alembertian, $$\square =\varvec{\nabla }^2$$
*F*: Derivative of *f*(*R*)
$\theta$: Divergence of velocity field
$$\mu $$: Direction cosine
$\pi$: Effective anisotropic stress
$\eta (a,k)$: Effective anisotropic stress parameterization
$$\rho $$: Energy density
$T_{\mu \nu }$: Energy momentum tensor
*w*: Equation of state
$F_{\alpha \beta }$: Fisher information matrix
$\sigma _8$: Fluctuation amplitude at 8 km/s/Mpc
$$u^\mu $$: Four-velocity
$\varOmega _m$: Fractional matter density
$f_{\mathrm {sky}}$: Fraction of sky observed
$\varDelta _M$: Gauge invariant comoving density contrast
$\tau (z)$: Generic opacity parameter
$\varpi$: Gravitational slip parameter
*G*(*a*): Growth function/growth factor
$\gamma$: Growth index/shear
$$f_g$$: Growth rate
$b_{\mathrm {eff}}$: Halo effective linear bias factor
*h*: Hubble constant in units of 100 km/s/Mpc
*H*(*z*): Hubble parameter
$$\xi _i$$: Killing field
$$\delta _{ij}$$: Kronecker delta
*f*(*R*): Lagrangian in modified gravity
$$P_l(\mu )$$: Legendre polynomials
$$\mathcal {L}(\varvec{\Theta })$$: Likelihood function
$\beta (z)$: Linear redshift-space distortion parameter
$$D_L(z)$$: Luminosity distance
*Q*(*a*, *k*): Mass screening effect
$\delta _m$: Matter density perturbation
$$g_{\mu \nu }$$: Metric tensor
$\mu$: Modified gravity function: $\mu =Q/\eta$
$C_\ell$: Multipole power spectrum
*G*: Newton’s gravitational constant
*N*: Number of e-folds, $N=\ln a$
*P*(*k*): Matter power spectrum
*p*: Pressure
$$\delta p$$: Pressure perturbation
$\chi (z)$: Radial, dimensionless comoving distance
*z*: Redshift
*R*: Ricci scalar
$$\phi $$: Scalar field
*A*: Scalar potential
$$\varPsi ,\varPhi $$: Scalar potentials
$n_s$: Scalar spectral index
*a*: Scale factor
$f_a$: Scale of Peccei–Quinn symmetry breaking
$\ell$: Spherical harmonic multipoles
$$c_s$$: Sound speed
$\varSigma$: Total neutrino mass/inverse covariance matrix/PPN parameter
$H_T^{ij}$: Trace-free distortion
*T*(*k*): Transfer function
$$B_i$$: Vector shift
$$\mathbf {k}$$: Wavenumber

## Footnotes

1 Continuously updated information on Euclid is available on http://www.euclid-ec.org.

2 As pointed out in Vikman (2005), barotropic fluids where the energy conservation equation defines the evolution can in any case not cross $w=-\,1$ as this is a fixed point of the evolution.

3 While the scalar field with a constant $w<-\,1$ does not exhibit instabilities at the level of classical perturbation theory, it is generally a ghost and unstable at the quantum level. It is however possible to construct stable models without ghosts that can cross the phantom barrier, for example, based on kinetic gravity braiding (Pujolas et al. 2011; Deffayet et al. 2010). As mentioned below, also the effective dark energy in coupled dark energy—dark matter models can cross the phantom barrier.

4 All *f*(*G*) models with *G* the Gauss–Bonnet term as in fact a particular choice of non-minimally coupled scalar–tensor theories, a subclass of the Horndeski lagrangian, see Kobayashi et al. (2011).

5 Not to be confused with a different formalism of the same name by other authors (Hu and Sawicki 2007b).

6 This subsection is based on Amendola and Tsujikawa (2010).

7 In presence of massive neutrinos $f_g$ depends also on the scale *k* (Kiakotou et al. 2008).

8 If we assume that neutrinos have a non-vanishing mass, then the transfer function is also redshift-dependent.

9 http://camb.info/.

10 See Hamilton (1992). $\tilde{\xi }(\sigma ,\pi )$ is the Fourier transform of $P_s(k)=(1+\beta \mu ^2)^2 P_r(k)$ (Kaiser 1987).

11 Forecasts are not updated in this version with respect to 2012.

12 For this reason, early dark-energy models can have a much stronger impact.

13 http://www.sdss3.org/surveys/boss.php.

14 http://wigglez.swin.edu.au/site/index.html.

15 http://www.darkenergysurvey.org.

16 http://www.mpe.mpg.de/erosita/.

17 http://www.wfxt.eu/home/Overview.html.

18 It is anyway worth noticing the controversial results of DAMA/LIBRA, and more recently of CoGeNT.

19 In this case we have added the contribution from BOSS at redshifts $0.1<z<z_{\min }$, where $z_{\min }=0.5$ is the minimum redshift of the Euclid spectroscopic survey.

20 In this scenario, isocurvature perturbations in the ALP energy density are produced, and can provide additional constraints.

21 Lyman-alpha forest power spectra can push constraints further at the high mass end, although the results in Amendola and Barbieri (2006) were not calibrated with simulations.

22 A clarification is in order here regarding the usage of the term “orthogonal”. Senatore et al. (2010) introduced two different bispectrum shapes with the same denomination: their Eq. (53) generates the $k^{-1}$ dependence in the linear bias parameter while their Eq. (113), which provides the correct large-scale behavior, causes a much weaker effect. Albeit unrelated to the orthogonal template, quasi-single-field models (Chen and Wang 2010) also give a $k^{-1}$ scaling of the bias.

23 Very few *N*-body simulations of the non-local models are currently available and none of them has very high spatial resolution.

24 Strictly speaking, isocurvature perturbations are defined by the condition that their total energy density in the total comoving gauge vanishes, i.e., $\sum _\alpha \delta \rho ^{({\mathrm {com.}})}_\alpha =0$. Using the relativistic Poisson equation, one can verify that this implies that they do not contribute to the “curvature” potential.

25 In this case in the flat gauge one finds, for each species $\alpha$, $\zeta _\alpha = \zeta$, where $\zeta$ is the Bardeen curvature perturbation conserved on super-Hubble scales.

26 Note that this convention is the opposite of the one used in Planck Collaboration (2014c), where “fully correlated” means that the cross-correlation term contributes constructively to the Sachs–Wolfe component of the total temperature spectrum.

27 http://www.wesselvalkenburg.nl/software/collambda/.

28 http://www.wesselvalkenburg.nl/software/voiddistances/.

29 Available from http://www.cosmology.org.za.
